# Inter‐and Intramolecular (4+3) Cycloadditions With Epoxy Allylsilanes as Dienophiles

**DOI:** 10.1002/asia.70708

**Published:** 2026-03-31

**Authors:** Qin Han Teo, Yuchen Zhou, Elizabeth H. Krenske, Pauline Chiu

**Affiliations:** ^1^ Department of Chemistry and State Key Laboratory of Synthetic Chemistry The University of Hong Kong Hong Kong P.R. China; ^2^ School of Chemistry and Molecular Biosciences The University of Queensland Queensland Australia

**Keywords:** (4+3) cycloaddition, allylsilanes, seven‐membered ring, methylenecycloheptane

## Abstract

Methylenated cycloheptanoids are synthesized by formal (4+3) cycloadditions of dienes with epoxy allylsilanes as dienophiles in the presence of catalytic amounts of TESOTf. Intermolecular and intramolecular (4+3) cycloadditions with dienes including furans, cyclopentadiene, pyrroles, thiophenes, and benzenes proceeded to provide polycyclic adducts in moderate to good yields.

## Graphical abstract

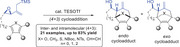

## 1 Introduction

**FIGURE 1:**
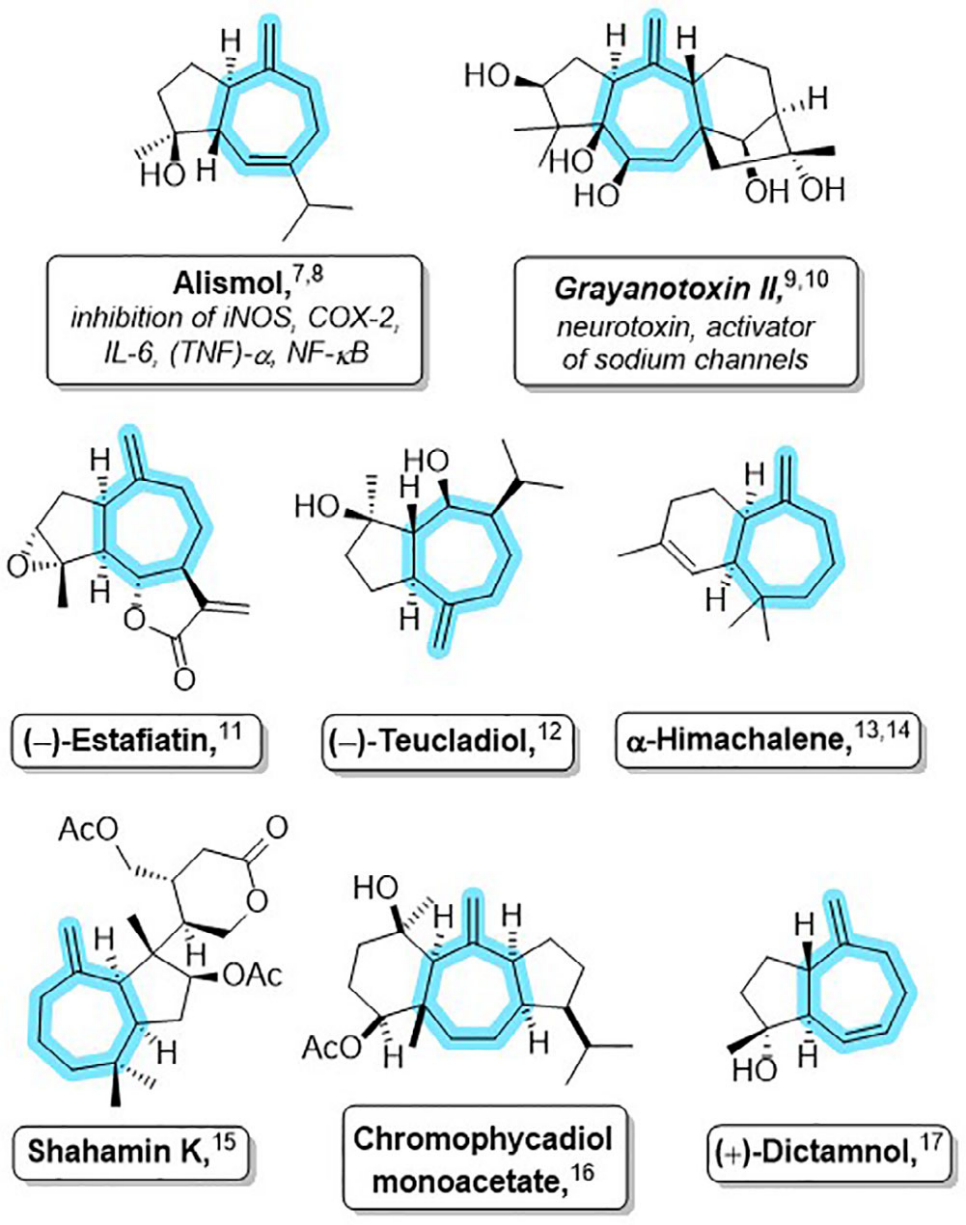
Natural products with methylenecycloheptane frameworks.

The synthesis of complex, functionalized seven‐membered carbocycles remains a pursuit in the synthetic organic chemistry community [1, 2, 3, 4, 5, 6], given the prevalence of seven‐membered ring core structures in many bioactive natural products with potential applications in pharmaceutical and agricultural industries, examples of which are shown in Figure 1 [7, 8, 9, 10, 11, 12, 13, 14, 15, 16, 17].

**SCHEME 1:**
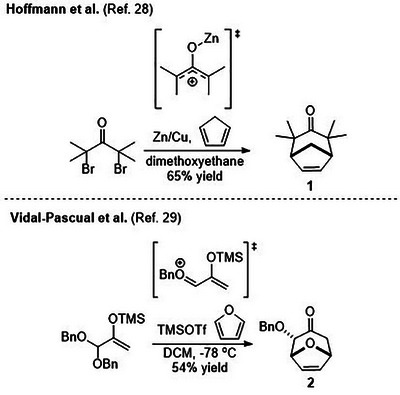
Examples of classical (4+3) cycloadditions involving oxyallylic cation intermediates.

Various annulation strategies have been developed to address the challenges associated with the synthesis of seven‐membered carbocycles [18, 19, 20, 21], and the (4+3) cycloaddition reaction is one of the most widely employed for constructing cycloheptanoids convergently [22, 23, 24, 25, 26, 27]. This cycloaddition reaction enables the rapid assembly of functional group‐endowed seven‐membered ring frameworks through the union of various dienes with a three‐carbon electrophilic dienophile, the most common type being oxyallylic cations. Therefore, classical (4+3) cycloadducts are typically substituted cycloheptenone derivatives, exemplified by cycloadducts 1 and 2. (Scheme 1) [28, 29].

**SCHEME 2:**
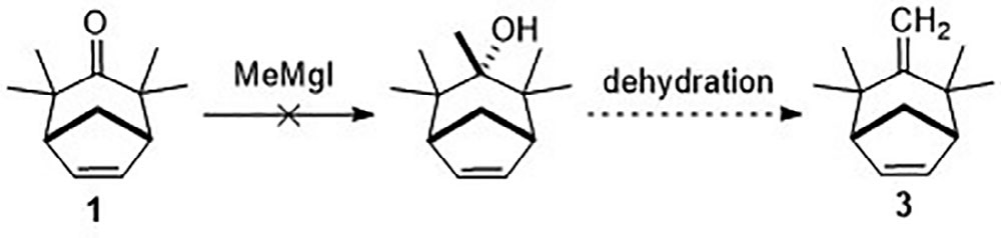
Attempted methylenation of cycloheptenone of 1.

The natural products shown in Figure 1 all share the common structural motif of a methylenated cycloheptane. Conceivably, methylenation of classical (4+3) cycloadducts such as 1 or 2 should yield the required frameworks toward the synthesis of these natural products. However, in practice, these transformations do not always proceed efficiently, particularly for more substituted or for polycyclic adducts. For example, bicycloheptadiene 3, which was desired as a model compound for studying the synthesis of didehydrozizaene, could not be secured by methylenation of cycloheptenone 1 by various means (Scheme 2) [30].

**SCHEME 3:**
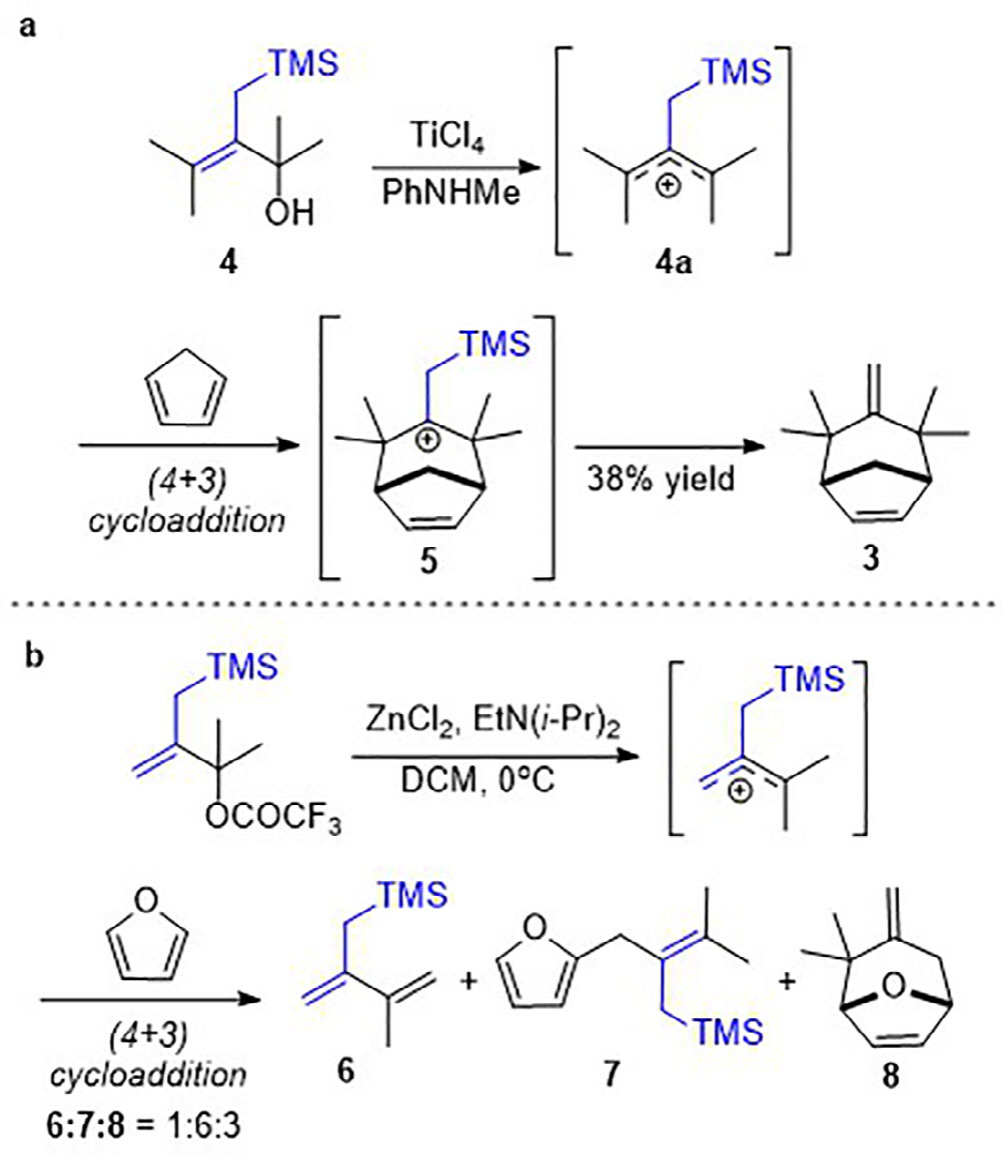
Allylsilanes as electrophiles in (4+3) cycloadditions.

Since methylenation of ketone 1 failed to yield 3, Hoffmann experimented with cycloadditions using an alternative, three‐carbon electrophile, allylic silane 4 (Scheme 3a). The reactive intermediate in this case is allylic cation 4a. After cycloaddition, a β‐silicon stabilized intermediate 5 is formed, and desilylation completed the reaction. While this reaction was able to generate the desired cycloheptenoid 3, the yield was not high (Scheme 3a) [31]. Furthermore, cycloadditions with aromatizable dienes like furan are often arrested to produce a Friedel‐Crafts type of product like 7, instead of proceeding to full cycloaddition to yield 8 (Scheme 3b) [32].

**SCHEME 4:**
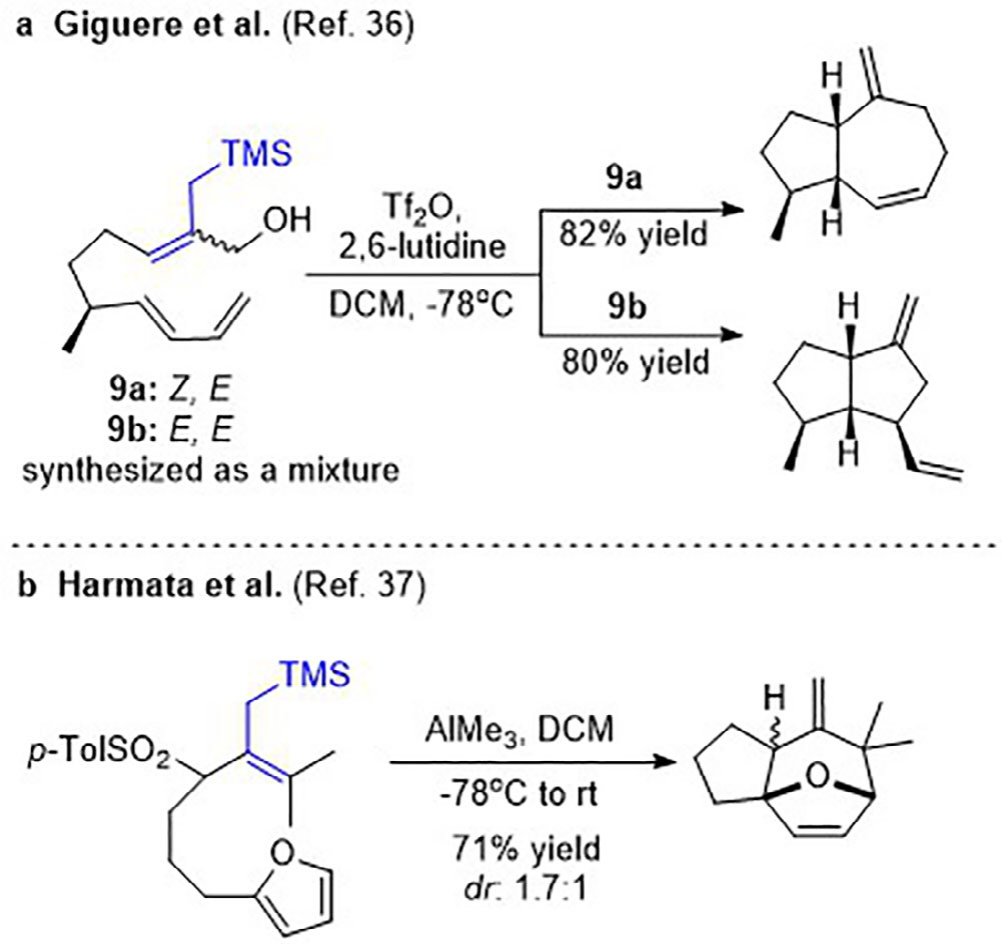
Examples of (4+3) cycloadditions involving allylsilanes.

Reports of (4+3) cycloadditions using allylic silanes as dienophiles are comparatively sparse [32, 33, 34, 35]. Apart from the sometimes poor yields of cycloadditions, other challenges include difficulties in the synthesis of the allylic silane cycloaddition precursors (Scheme 4a) [36] and low diastereoselectivities (Scheme 4b) [37].

**SCHEME 5:**
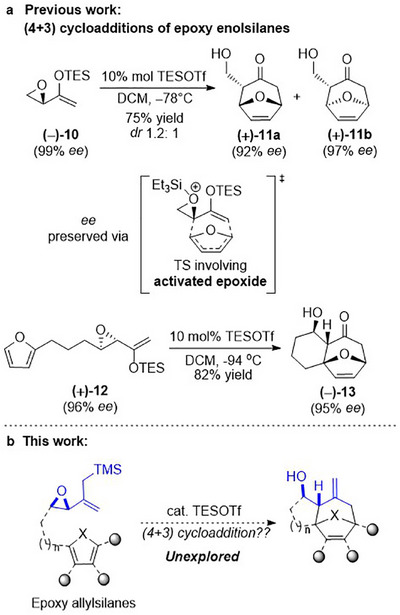
a) Previous work: Inter‐ and intramolecular (4+3) cycloadditions of epoxy enolsilanes as dienophiles. b) This work: Epoxy allylsilane as dienophile in (4+3) cycloaddition reaction.

Our group has been working on (4+3) cycloaddition reactions that utilize epoxy enolsilanes as the dienophile [38, 39, 40, 41]. Under silyl triflate catalysis, β‐hydroxylated cycloheptanones like 11 and 13 are obtained as cycloadducts (Scheme 5a). We have demonstrated that, in contrast to the classical (4+3) cycloadditions, the intermediate reacting with the diene is not an oxyallylic cation but a silyl‐activated epoxide [42]. This allows the stereochemistry of the epoxide to direct the cycloaddition. We have examined the intramolecular (4+3) cycloaddition with many dienes and found that even traditionally unreactive dienes like thiophenes [43] and benzenoids also underwent cycloaddition [44]. For the dienophile, we have examined the variations of the basic epoxy enolsilane structure on the reaction outcome. We have studied the effect of substituents at each position [45, 46, 47]. and the use of other strained heterocycles such as aziridines [48], oxetanes, and azetidines [49]. We have also applied this cycloaddition to the synthesis of natural products, including (+) cortistatin A [50] and (−)‐pseudolaric acid B [51].

A final parameter in this (4+3) cycloaddition that remains unexamined thus far is the enolsilane itself–how the reaction would proceed with alternatives to the enolsilane moiety, for example, an allylsilane (Scheme 5b). Structures such as those found in the natural products in Figure 1 sparked our interest in exploring whether an epoxy allylsilane could also be effective in this (4+3) cycloaddition reaction (Scheme 5b).

**SCHEME 6:**
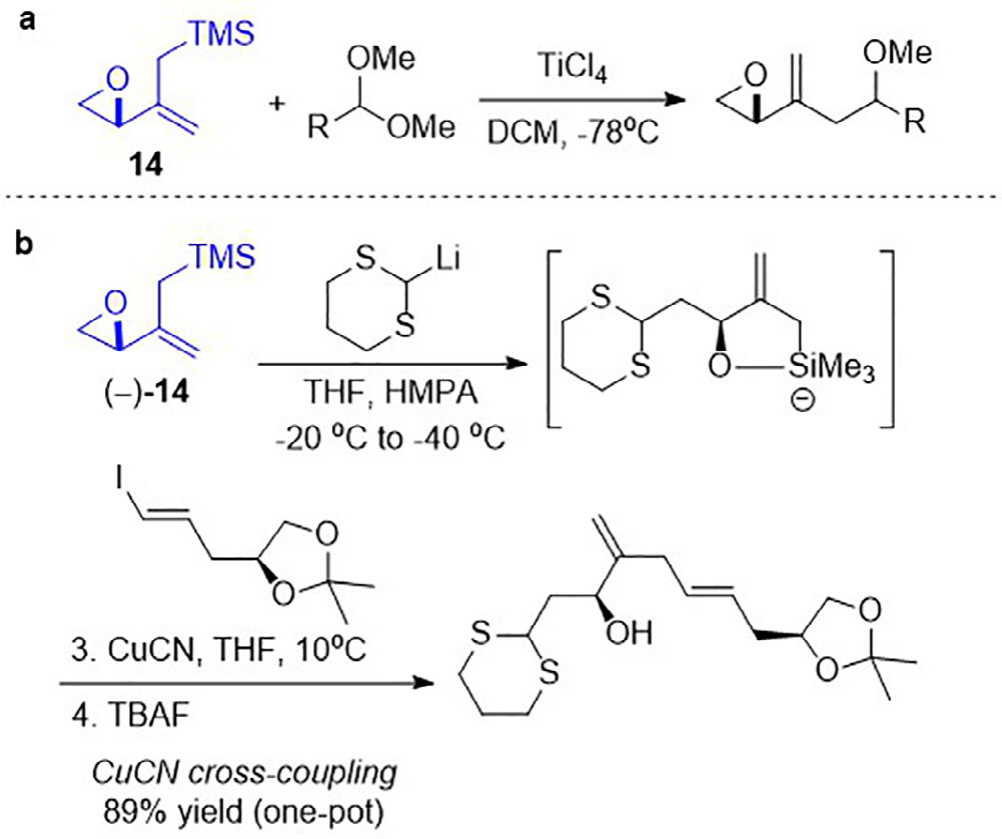
Documented reactions of epoxy allylsilane 14.

Interestingly, while epoxy allylsilane 14 has been reported in the literature, studies employing 14 thus far engaged the functional groups individually, that is, as an epoxide (Scheme 6a) [52] or as an allylsilane (Scheme 6b) [53], without new reactivity conferred through the juxtapositioning of the two functional groups. To the best of our knowledge, epoxy allylsilanes have not yet been reported to undergo any cycloaddition reactions.

Both to complete the investigations on the scope and variations of the (4+3) cycloaddition, as well as interest in the methylenated cycloheptanoid products that this reaction could potentially afford, we embarked on a study of (4+3) cycloadditions employing epoxy allylsilanes as the dienophile [54].

## 2 Results and Discussion

### 2.1 Studies on the Intermolecular (4+3) Cycloadditions of Epoxy Allylsilane 14

**SCHEME 7:**
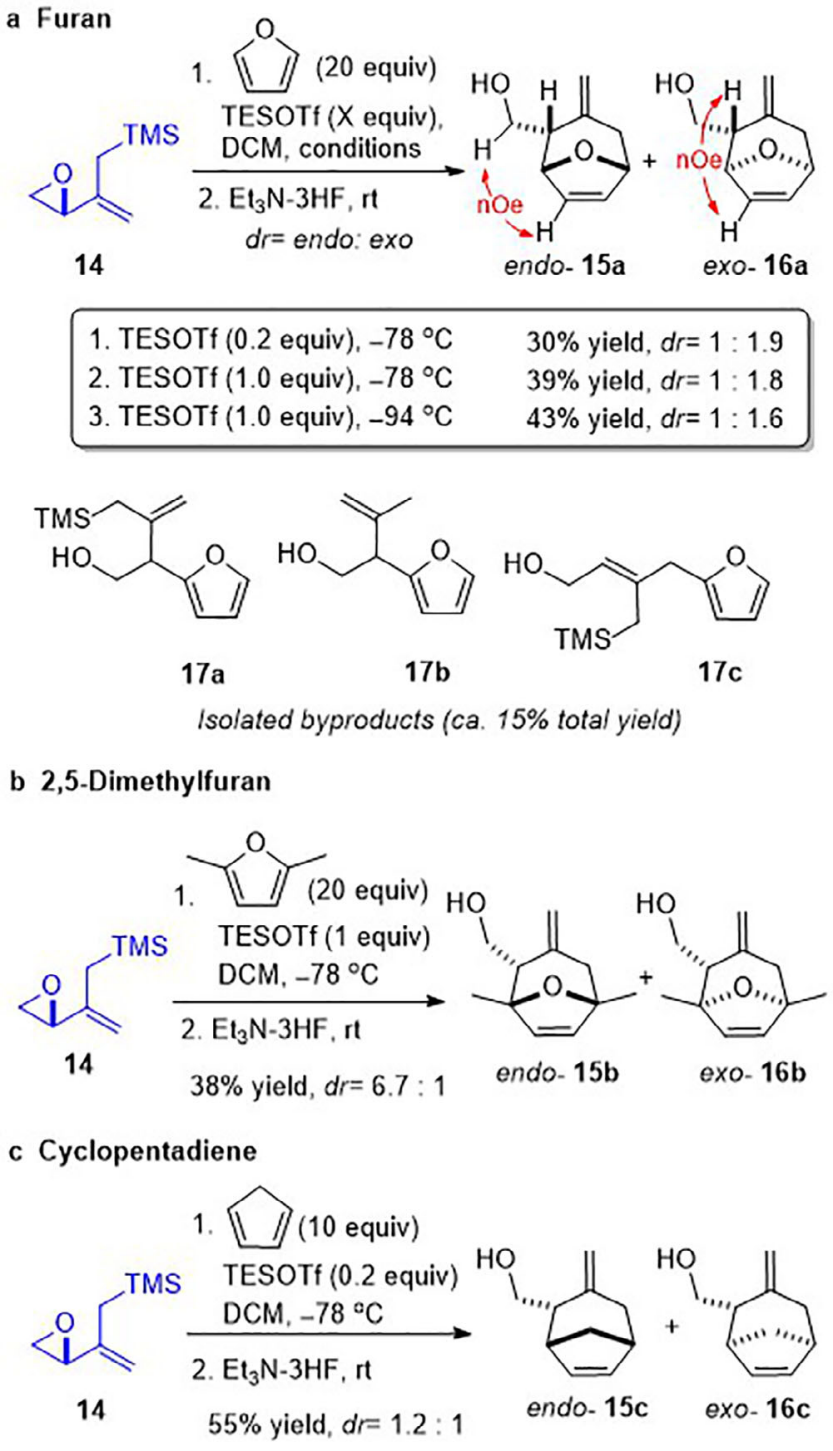
Intermolecular (4+3) cycloadditions of epoxy allylsilane **14** with cyclic dienes; diastereomeric ratios (*dr*) determined from ^1^H NMR analysis of the crude product mixture.

**SCHEME 8:**
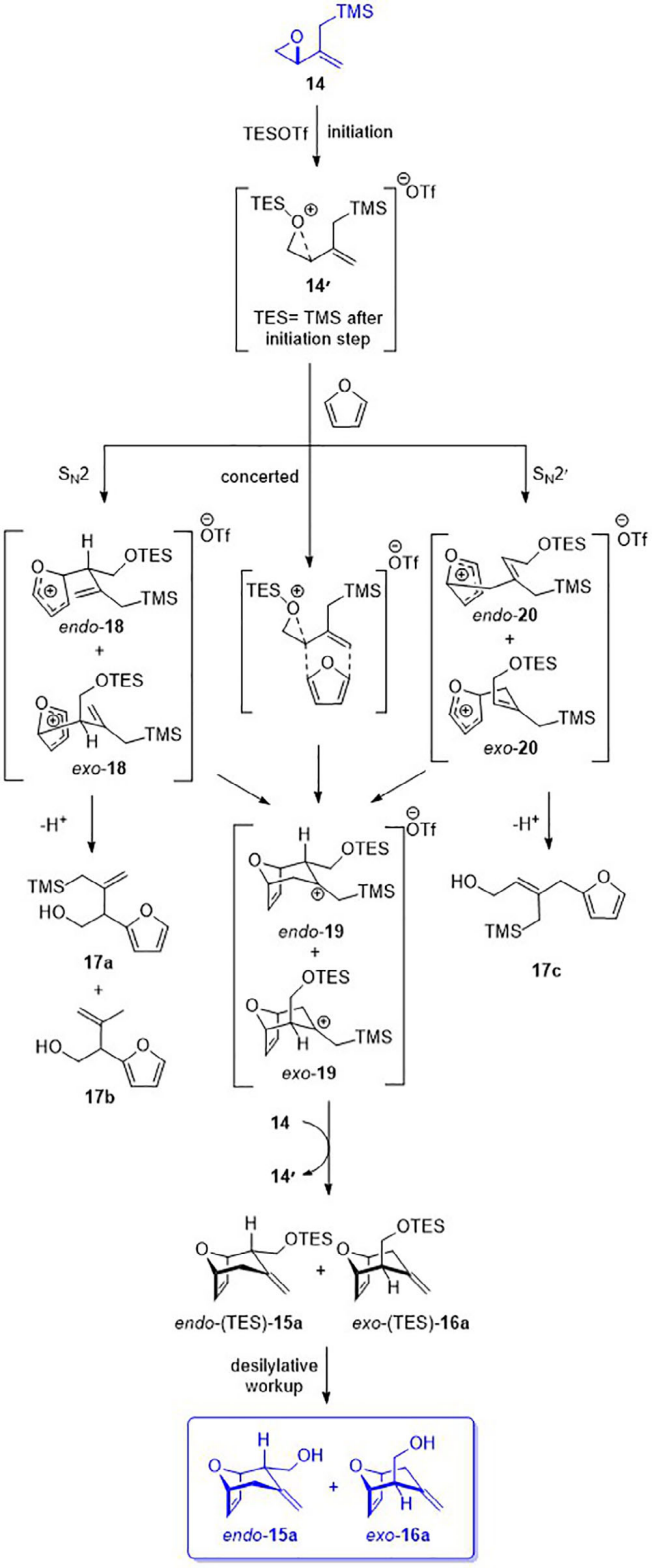
Proposed catalytic cycle for the formation of intermolecular (4+3) cycloadducts **15a, 16a** and side products **17a‐c**.

Epoxy allylsilane **14,** prepared according to the literature [52, 55, 56], was treated with a catalytic amount of TESOTf in the presence of 20 equivalents of furan (0.2 equiv. TESOTf, −78°C, Scheme 7a). The (4+3) cycloaddition proceeded in 30% yield to afford *endo*‐**15a** and *exo*‐**16a** with a slight preference for the *exo* cycloadduct. The relative stereochemistry of the cycloadducts was established by 2D‐NOESY experiments. Optimizations of the equivalents of silyl triflate and the temperature increased the cycloaddition yield to 43%. These cycloadditions were accompanied by the formation of 14–19% of Friedel‐Crafts products **17a**‐**c**, consistent with previous reports by Hoffmann [32] (Scheme 3b, compound **7**), suggesting that the cycloaddition involving an allylsilane dienophile proceeds via a more stepwise mechanistic pathway. Notably, **17a** and **17b** are products of a derailed cycloaddition where aromatization outcompeted the second C─C bond formation. On the other hand, **17c** is derived from an S_N_2ʹ attack on the activated epoxy allylsilane (vide infra, Scheme 8).

2,5‐Dimethylfuran also underwent (4+3) cycloaddition with **14** in moderate yields (Scheme 7b). The computational studies (vide infra) suggest that the apparently higher dr in this case stems from the higher energy barrier for the *exo*‐cycloaddition intermediate to complete ring closure, which results in some of the intermediate to be diverted to decomposition instead (see the Supporting Information for details). When the more reactive diene, cyclopentadiene, was employed, the intermolecular cycloaddition proceeded to afford cycloadducts **15c** and **16c** in higher yield (Scheme 7c). The low endo:exo selectivities observed in these cycloadditions are not unlike those observed for the intermolecular (4+3) cycloadditions of epoxy enolsilanes (Scheme 5a).

### 2.2 Computational Studies of the Cycloaddition Mechanism

**FIGURE 2:**
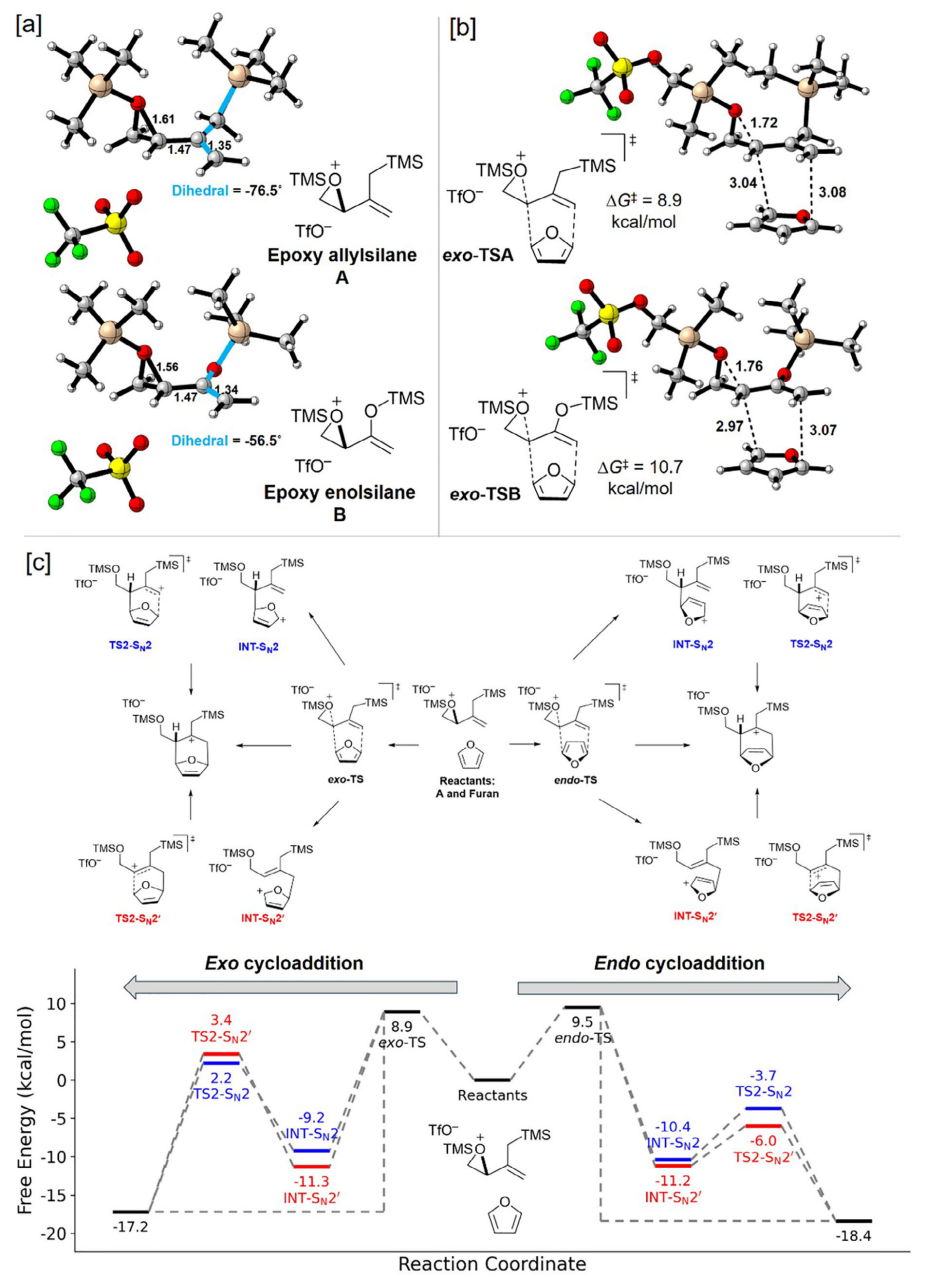
a,b) Comparisons of the structures of the epoxy enol‐ and allylsilanes **A** and **B** and the transition states and barriers for their *exo* (4+3) cycloadditions with furan. c) Free energy profile showing various possible pathways for the (4+3) cycloaddition of epoxy allylsilane **A** with furan. The calculations were performed with M06‐2X/def2‐TZVPP/SMD(CH_2_Cl_2_)//B3LYP‐D3(BJ)/6‐31G(d,p)/CPCM(CH_2_Cl_2_) level of theory.

Density functional theory computations were carried out to elucidate the reaction mechanism of the epoxy allylsilane cycloaddition (Figure 2). Our computations used the M06‐2X/def2‐TZVPP/SMD (CH_2_Cl_2_)//B3LYP‐D3(BJ)/6‐31G(d,p)/CPCM(CH_2_Cl_2_) level of theory [57, 58, 59, 60, 61, 62, 63, 64, 65, 66]. We began by comparing the activated forms of a model epoxy allylsilane (**A**) and epoxy enolsilane (**B**). The calculated bond lengths indicate that the epoxide in allylsilane **A** is more predisposed toward ring‐opening, with a C─O bond length of 1.61 Å, in comparison with 1.56 Å in enolsilane **B**. Analysis of the molecular geometries suggests that the weaker C─O bond may be related to the less σ‐withdrawing character of the allyl CH_2_ carbon of **A**, as compared to the enol oxygen of **B**. In enolsilane **B**, the strong σ‐withdrawing enol oxygen reduces the ability of the epoxide carbon to take on a developing positive charge, whereas in allylsilane **A**, the CH_2_ carbon is not σ‐withdrawing and the epoxide carbon has more positive charge. The importance of σ‐effects rather than π‐effects is due to the fact that the enolsilane unit in **B** is twisted rather than planar, which prevents full conjugation between the enol oxygen lone pairs and the C═C π system (See the Supporting Information for details). Consistent with the weaker epoxide C─O bond in allylsilane **A**, a greater propensity of the allylsilane to react with a diene is evident in the barriers for the reactions of **A** and **B** with furan: the ∆*G* ^‡^ for the reaction of allylsilane **A** (8.9 kcal/mol) is 1.8 kcal/mol lower than the reaction barrier for enolsilane **B** (10.7 kcal/mol).

Exploring the cycloaddition pathways of epoxy allylsilane **A** in detail, a 2D relaxed scan of the forming C–C bond distances suggested a plausible trifurcation of the pathway (see the Supporting Information for details). That is, for a given stereochemistry, after the transition state for the initial attack by the diene is reached (i.e., ***exo*‐TSA** or ***endo*‐TSA**), the two C–C bonds may form in either a concerted or stepwise manner, with two stepwise alternatives existing that differ with respect to which of the C–C bonds forms first. The stepwise pathways correspond to S_N_2 and S_N_2′ attack on the activated epoxy allylsilane, giving rise to structures **18** and **20**, respectively (Scheme 8). The concerted pathway leads directly to the cycloadduct **19**. A free energy profile showing the transition states and intermediates for the *endo* and *exo* cycloadditions of **A** with furan is shown in Figure 2(c). The energy profile reveals that the difference in energy between the initial attack by the diene in the *endo* and *exo* modes favors *exo* by 0.6 kcal/mol, which corresponds to a theoretical *dr* of 1:5 at –78°C. However, due to the trifurcating shape of the energy landscape, the relative contributions of the different reaction pathways cannot be predicted solely on the basis of the energies of the transition states and intermediates; post‐transition state dynamics would also play a role. One consideration is that the *endo* and *exo* cycloadditions differ with respect to the sizes of the barriers for the ring closure transition states. In the *endo* cycloaddition, the barriers for ring closure are smaller than the barrier for the initial attack. In contrast, in the *exo* cycloaddition, the barriers for the ring closures are 3–6 kcal/mol higher than the barrier for the initial attack. This suggests that for the reaction pathways that involve intermediates, the *exo* intermediates may have longer lifetimes than the *endo* intermediates, allowing the *exo* intermediates to undergo competitive side reactions leading to byproducts **17a**, **17b**, and **17c** and reducing the overall amount of *exo* cycloadduct formed. Factoring the formation of these byproducts into the possible fates of the *exo* intermediates would lead to a closer agreement with the small level of *exo* diastereoselectivity observed experimentally (*ca*. 1:2).

Scheme 8 shows a plausible mechanism, informed from the computational study, for the TESOTf‐catalyzed reactions of epoxy allylsilane **14** and furan. The reaction of **14** with TESOTf by silylation of the oxirane leads to key activated epoxide intermediate **14′**. Concerted cycloaddition of **14′** with furan results in **19**, which is stabilized by the β‐silicon effect [67]. A stepwise mechanism also operates and is evidenced by the observation and isolation of side products. Stepwise C‐C bond formations via S_N_2 or S_N_2′ attack of **14′** lead to intermediates **18** and **20,** respectively. From these intermediates, subsequent C‐C bond formations generate **19**, but they also undergo rearomatizing side reactions to produce side products **17a‐c**. Intermediate **19**, formed from all three pathways, undergoes desilylation, which induces the formation of **14ʹ** from **14** and also delivers the intermolecular cycloadducts **15a** and **16a**.

### 2.3 Synthesis of Intramolecular Cycloaddition Substrates

**SCHEME 9:**
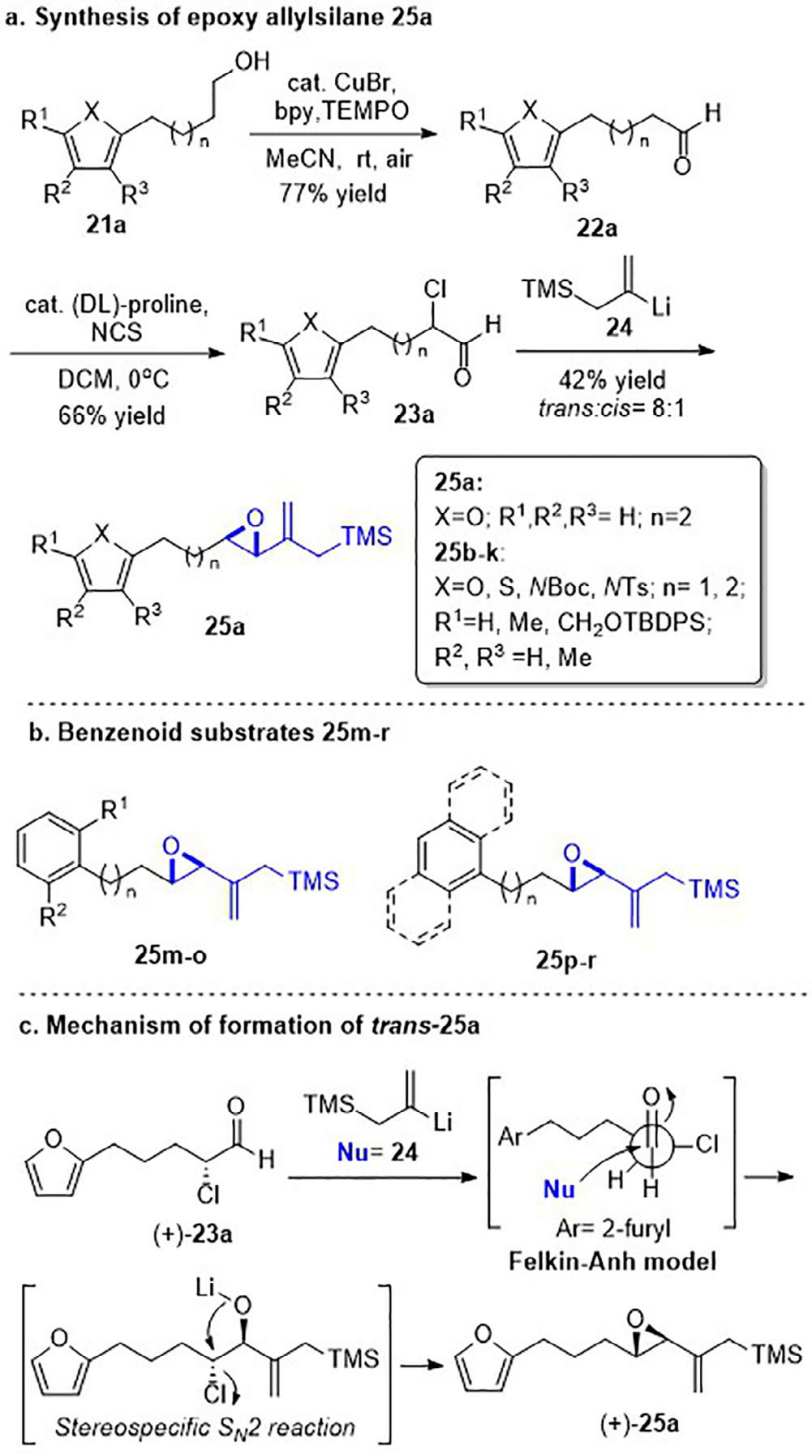
a) Representative route for the synthesis of epoxy allylsilanes **25** for the intramolecular (4+3) cycloaddition reaction. b) Benzenoid‐tethered substrates for the intramolecular (4+3) cycloaddition. c) The Felkin‐Anh model rationalizes the stereoselectivity of the vinyllithium addition reaction to generate the major chloroalcoholate, which then undergoes a stereospecific intramolecular S_N_2 reaction to yield the *trans*‐epoxide.

**SCHEME 10:**
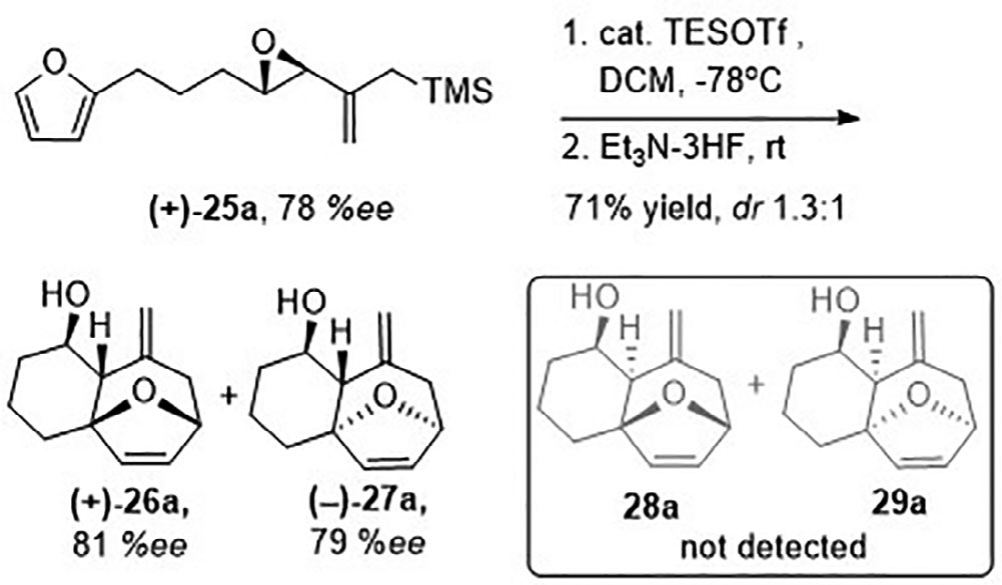
Intramolecular (4+3) cycloaddition of **(+)‐25a**.

To embark on the investigation of the intramolecular version of this cycloaddition, substrates that tether the epoxy allylsilane moiety to the diene need to be synthesized. The required *trans*‐epoxide was retroanalyzed to a chloroaldehyde, which could undergo propenylsilane addition‐intramolecular S_N_2 reaction to prepare the epoxy allylsilane moiety in one step. Scheme 9a shows the synthesis of **25a**, which is typical of the preparation of these intramolecular cycloaddition substrates. Furylated alkanol **21a** is oxidized to aldehyde **22a**, which was then subjected to amine‐catalyzed chlorination to generate chloroaldehyde **23a**. This strategy permits the synthesis of enantiomerically‐enriched chloroaldehyde **(+)‐23a** by employing (*L*)‐prolinamide as the chiral catalyst, albeit in just 78% *ee* [68]. The addition of the vinyllithium **24** follows Felkin‐Anh selectivity as illustrated in Scheme 10c to provide *trans*‐epoxide **25a** as the major diastereomer from **23a**. Pyrrole, thiophene, and arene‐tethered epoxy allylsilanes **25b‐k**, **25m‐r** were synthesized by similar routes (Scheme 9a,b; for details, see Supporting Information). Fortunately, the *trans*‐epoxy allylsilanes could be obtained in pure forms by separating them from the minor *cis*‐diastereomers using flash column chromatography.

### 2.4 Studies on the Intramolecular (4+3) Cycloadditions of Epoxy Allylsilanes 25

We first investigated the intramolecular (4+3) cycloadditions of the epoxy allylsilanes tethered to furans (Table 1). Treatment of epoxy allylsilanes **25a‐c** with catalytic amounts of TESOTf at low temperatures resulted in the formation of cycloadducts in moderate to good yields. However, the *endo*/*exo* selectivities were low (**26a‐c**:**27a‐c** ∼1:1) (Table 1, entries 1–2), quite a departure from the high diastereoselectivities observed in the cycloadditions of the corresponding enolsilanes (Scheme 5a). However, for **25d**, which has a 3‐methyl substituent on the furan, the cycloaddition in the presence of TESOTf furnished *exo*‐cycloadduct **27d** exclusively (Table 1, entry 3). A substituent at the 3‐position exerts a steric effect that hinders and disfavors an *endo* cycloaddition, a trend that has been observed previously in our studies [43]. The low yield in this particular cycloaddition is attributed to the easily decomposed 2,3‐disubstituted furan in **25d**, which also made it challenging to synthesize. Epoxy allylsilanes **25e** and **25f** with shorter tethers were also subjected to the typical cycloaddition conditions and were found to generate 5,7‐fused bicyclic cycloadducts in moderate yields (Table 1, entries 4–5).

**TABLE 1:** Intramolecular (4+3) cycloadditions of epoxy allylsilanes **25** with furans.^a^

Entry	Substrate	Cycloadduct^b^
1	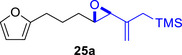	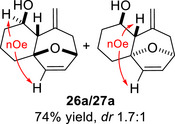
2	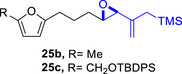	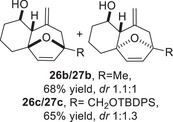
3	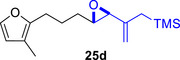	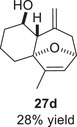
4	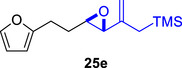	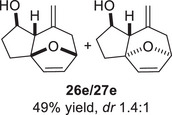
5	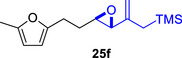	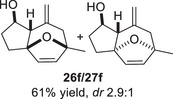

^a^ Reaction conditions: TESOTf (0.2 equiv., 0.2 M in DCM), DCM, ‐78°C; then Et_3_N‐3HF (3 equiv.), rt.

^b^ Isolated yield; *dr* was determined by NMR analysis of the crude product.

We also examined the (4+3) cycloaddition reaction of optically enriched epoxy allylsilane **(+)‐25a** and found that the *endo* and *exo* cycloadducts **(+)‐26a** and **(‒)‐27a** were obtained in good yields and with retention of *ee*. Importantly, diastereomeric cycloadducts **28a** and **29a**, products that would have arisen from reaction with an oxyallylic cation, were not observed (Scheme 10). The results are consistent with a cycloaddition mechanism in which the furan reacts with a TES‐activated epoxide through an S_N_2‐like, backside attack [42].

As epoxy enolsilanes successfully engaged in dearomative intramolecular (4+3) cycloadditions with many heterocycles [41, 43, 44], we also examined the reactivity of epoxy allylic silanes with various heterocyclic dienes. Epoxy allylsilanes **25g‐h** tethered to pyrroles underwent (4+3) cycloaddition reactions smoothly (Table 2, entries 1–2). Both Boc‐and tosyl‐protected pyrroles reacted to deliver cycloadducts **26 g/27 g** and **26 h/27 h** in good yields, with moderate *dr*. Similarly, the intramolecular (4+3) cycloadditions of epoxy allylsilanes **25i‐k** with thiophenes were successful, with higher yields of cycloadducts obtained with the more electron‐rich thiophenes (Table 2, entries 3–5). Introducing an electron‐donating alkyl substituent at the 3‐position of the thiophene improved both the reaction yield and *dr* of the cycloaddition reaction. For example, the reactions of **25j** and **25k** in the presence of TESOTf afforded **27j** and **27k** both exclusively as *exo*‐diastereomers, in 54% and 59% yields, respectively (Table 2, entries 4–5). Compared with 3‐substituted furans like **25d**, the polyalkylated thiophenes were much less sensitive to decomposition under the reaction conditions, resulting in to better cycloaddition yields.

**TABLE 2:** Intramolecular (4+3) cycloadditions of epoxy allylsilane **25** with pyrroles and thiophenes.^a^

Entry	Substrate	Cycloadduct^b^
1	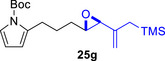	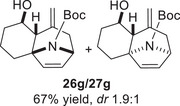
2	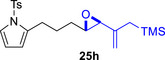	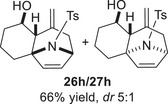
3	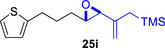	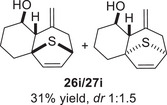
4	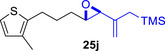	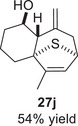
5	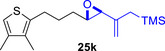	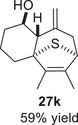

^a^ Reaction conditions: TESOTf (0.2‐1 equiv., 0.2 M in DCM), DCM, ‐78°C; then Et_3_N‐3HF (3 equiv.), rt.

^b^ Isolated yield; *dr* was determined by NMR analysis of the crude product.

**FIGURE 3:**
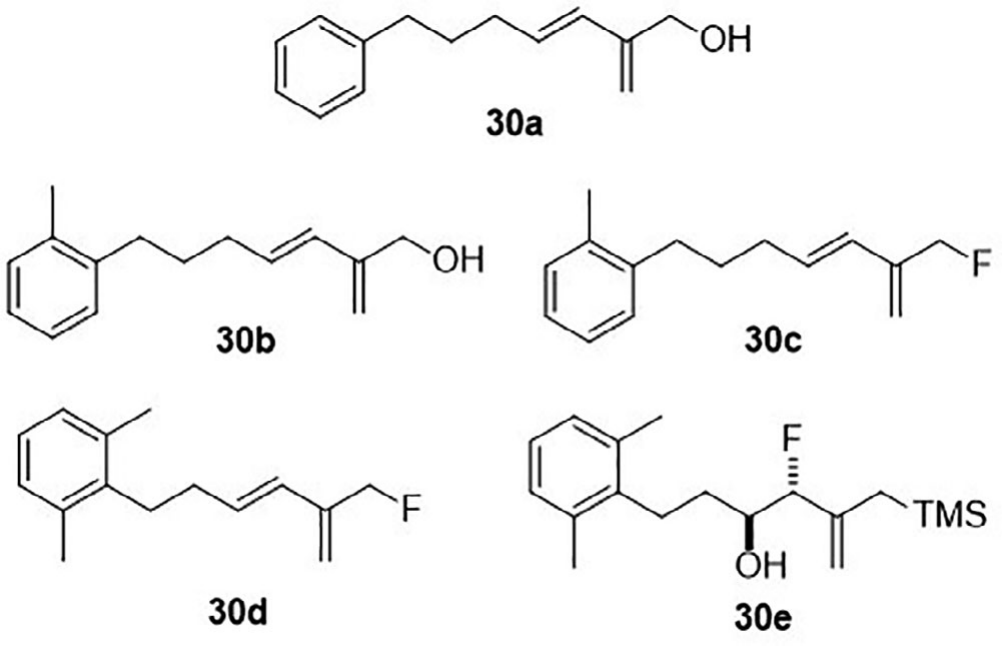
Side products identified from the reactions of **25m‐o**.

Besides heterocycles, our previous work showed that even benzenes underwent dearomatization to participate as dienes in the (4+3) cycloaddition with epoxy and aziridinyl enolsilanes [44]. We thus prepared **25m‐r** to study whether intramolecular (4+3) cycloadditions would occur (Table 3). In the event, no cycloadducts were found when **25m** was subjected to the reaction conditions, and only diene **30a** was isolated (Table 3, entry 1; Figure 3). The reaction of **25n** also did not produce cycloadducts, and only dienes **30b** and **30c** were obtained (Table 3, entry 2; Figure 3). It should be noted that the enolsilane analogues corresponding to **25m** and **25n** underwent intramolecular (4+3) cycloadditions successfully in 43% and 62% yields, respectively [44]. This indicated that under silyl triflate catalysis, the epoxide was sufficiently activated for reaction; however, the arenes were not reactive enough to engage in bond formation, giving way to other reactions that led to the observed side products (for a proposed mechanism, see the Supporting Information).

**TABLE 3:** Intramolecular (4+3) cycloadditions of epoxy allylsilane **25** with arenes.^a^

Entry	Substrate	Cycloadduct ^b^
1	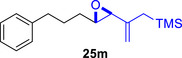	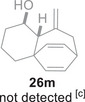 ^c^
2	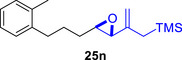	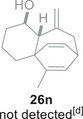 ^d^
3	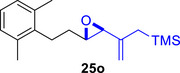	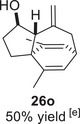 ^e^
4	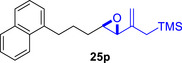	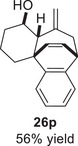
5	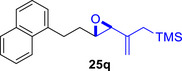	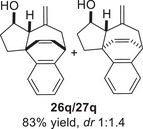
6	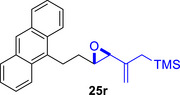	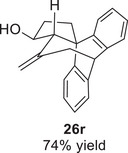

^a^ Reaction conditions: TESOTf (0.5‐1 equiv., 0.2 M in DCM), DCM, ‐78°C; then Et_3_N‐3HF (3 equiv.), rt.

^b^ Isolated yield; *dr* was determined by NMR analysis of the crude product.

^c^ **30a** (14% yield) was obtained.

^d^ **30b** (60% yield) and **30c** (23% yield) were obtained.

^e^ **30d** (20% yield) and **30e** (15% yield) were also obtained.

However, the cycloaddition was successful for dimethylbenzene **25o**, which proceeded to deliver cycloadduct **26o** in 50% yield (Table 3, entry 3), along with some side products **30d** and **30e** (Figure 3). This demonstrated that the dearomative (4+3) cycloaddition of benzene with activated epoxy allylsilanes is possible, except that more electron‐rich arenes are needed. The decrease in the tether chain length may be an additional factor favoring cycloaddition.

Epoxy allylic silanes **25p‐r** tethered to polyaromatic arenes such as naphthalene and anthracene also underwent dearomative (4+3) cycloaddition successfully to generate the corresponding cycloadducts in moderate to good yields, without the formation of diene side products (Table 3, entries 4–6). Naphthalene **25p** with a three‐carbon tether to the epoxy allylsilane reacted to afford **26p** as a single diastereomer (Table 3, entry 4). The cycloaddition reaction of naphthalene **25q** with a two‐carbon tether also proceeded successfully to afford both *endo* **‐26q** and *exo* **‐27q** in excellent overall yield (Table 3, entry 5). Lastly, anthracene **25r** also underwent cycloaddition to produce cycloadduct **26r** in good yield (Table 3, entry 6).

**FIGURE 4:**
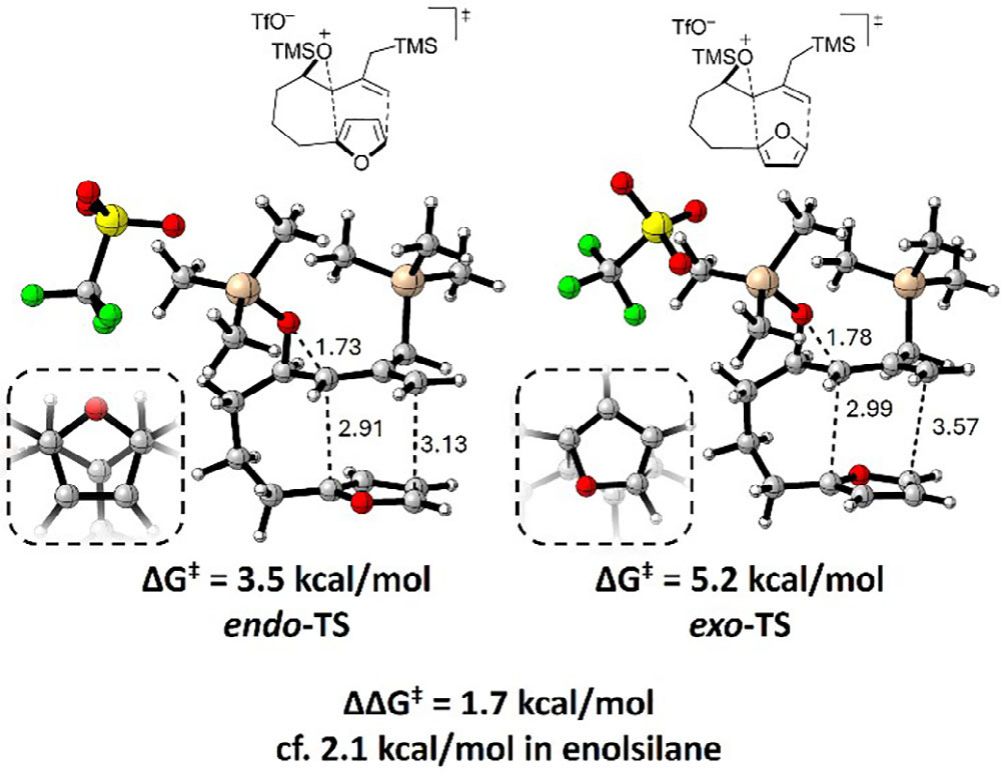
Transition states for intramolecular (4+3) cycloadditions of a model of allylsilane **25a**. The insets at the bottom left of each structure show a bottom‐up view of the reacting moieties.

Compared to the results obtained from our previous work [38, 39, 43, 44] on the (4+3) cycloadditions of epoxy enolsilanes with various dienes, the overall yields of the cycloadducts obtained from the inter‐and intramolecular (4+3) cycloadditions with epoxy allylsilanes as dienophiles are generally lower, with more side products formed in the reactions. Moreover, intramolecular cycloadditions with epoxy allylic silanes also tend to be less diastereoselective. This is evident when comparing the cycloadditions of **12** and **25a**, which are otherwise structurally the same: the cycloaddition of enolsilane derivative **12** produced the *endo* cycloadduct **13** exclusively in 83% yield (Scheme 5a), whereas the *dr* of the cycloaddition of the allylsilane **25a** was 1.7:1 (Table 1, entry 1). Our previous calculations on the intramolecular enolsilane cycloaddition showed that the *endo*/*exo* selectivity was controlled by the more favorable alignment of the reacting centers of the furan and three‐carbon moieties [43]. A diagram illustrating the application of this same concept in the context of the allylsilane is shown in Figure 4: the reacting carbons align better in the *endo* TS than the *exo* TS. On going from enolsilane to allylsilane, the difference in energy between the transition states for initial attack in the *endo* and *exo* modes decreases by a small amount (ca. 0.4 kcal/mol). While this agrees qualitatively with experiment, we believe that an understanding of the diastereoselectivity must also take into account the fact that the fate of the system after the initial attack by the diene (i.e., concerted or stepwise bond formation) is determined not only by the energies of the relevant intermediates but also by the shape of the energy surface, which would require a treatment of post‐transition state dynamic effects to evaluate fully (See the Supporting Information for the discussion).

### 2.5 Intramolecular (3+2) Cycloaddition

**SCHEME 11:**
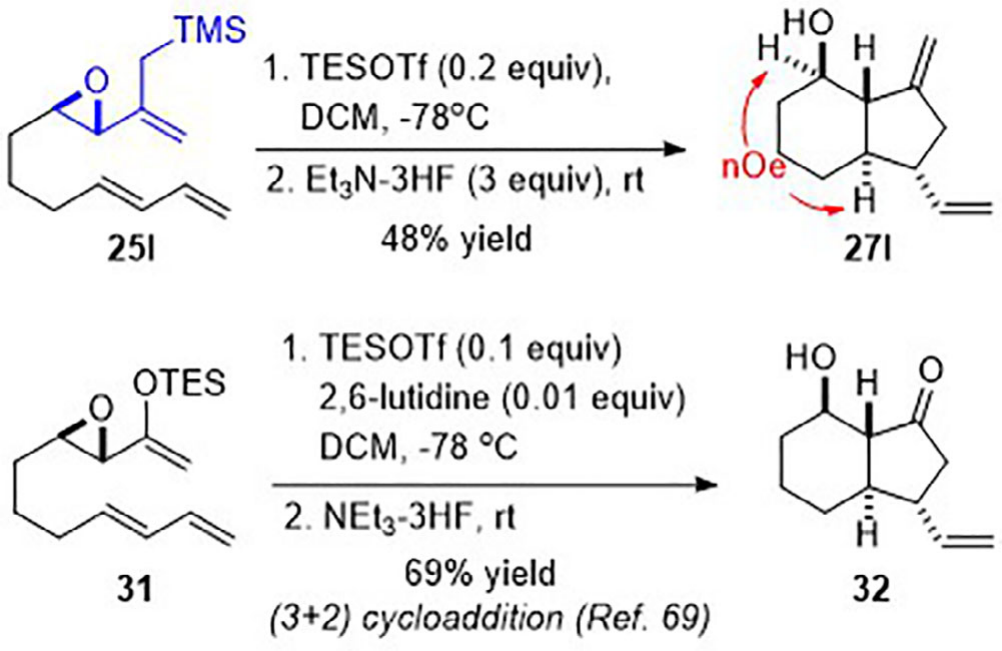
(3+2) cycloadditions of epoxy allylsilane **25l** compared with enolsilane **31**.

Finally, we also prepared epoxy allylsilane **25l** tethered to an acyclic diene. Subjecting it to the cycloaddition conditions produced a (3+2) cycloadduct, indene **27l**, as a single diastereomer (Scheme 11), an outcome that is analogous to the reaction of the epoxy enolsilane analog **31** [69].

## 3 Conclusion

In this report, we present the first study on the inter‐ and intramolecular (4+3) cycloadditions with epoxy allylsilanes as the dienophiles. We have demonstrated that epoxy allylsilanes is a viable dienophile to engage in cycloaddition reactions. Treatment of epoxy allylsilanes and dienes with a catalytic amount of silyl triflate leads to the formation of both *endo* and *exo* cycloadducts with an *exo*‐methylene cycloheptane framework in moderate to good yields. Computations suggest that, compared to epoxy enolsilanes, the epoxy allylsilanes are more reactive dienophiles, but the stepwise intermediates that form during their cycloadditions sometimes encounter higher barriers to undergo ring closure, which lead both to lower diastereoselectivities and to a greater propensity toward side reactions. The cycloaddition reaction is diastereoselective when reacting with dienes bearing a substituent at the 3‐position. The (4+3) cycloaddition of an enantio‐enriched epoxy allylsilane resulted in optically enriched cycloadducts with retention of *ee*. These cycloadducts are formed via the mechanism where the diene undergoes S_N_2‐like backside attack on the activated epoxy allylsilanes.

## 4 Experimental Section

### 4.1 General Experimental

All anhydrous reactions were performed in oven‐dried round‐bottomed flasks under a positive pressure of dry argon. Solvents and chemicals were purified according to standard procedures. Dichloromethane (DCM), furan, triethylamine (Et_3_N) were distilled from CaH_2_ under argon, then dried over 4 Å MS. Cyclopentadiene was obtained from cracking commercially available dicyclopentadiene. Other reagents were used as received.

^1^H and ^13^C NMR nuclear magnetic resonance spectra were recorded in deuterochloroform (CDCl_3_) with tetramethylsilane (TMS) as an internal standard at ambient temperature on a Bruker Avance 600 spectrometer operating at 600 MHz for ^1^H; and 150 MHz for ^13^C. All spectra were calibrated using the solvent resonance internal standard at δ 7.26 ppm for ^1^H spectra (residual CHCl_3_), and δ 77.16 ppm for ^13^C spectra. IR absorption spectra were recorded on a PerkinElmer‐Spectrum Two FT‐IR Spectrometer from 4000 cm^−1^ to 400 cm^−1^. Electron impact mass spectrometry was recorded on a Finnigan MAT 95 mass spectrometer. Analytical HPLC was carried out on an Agilent 1260 Infinity II LC system equipped with a G7111A quaternary pump, a G7129A vial sampler with integrated column compartment G7130A, and a variable wavelength G7114B detector operating with Agilent OpenLab CDS ChemStation Edition Rev. 2.4 software.

### 4.2 Typical Procedures for the (4+3) Cycloaddition Reactions of Epoxy Allylsilanes

#### 4.2.1 Intermolecular (4+3) Cycloaddition of Epoxy Allylsilane 14 With Cyclopentadiene 

To a solution of epoxy allylsilane 14 (0.10 g, 0.64 mmol, 1.0 equiv) in dry DCM (6.4 mL, 0.10 M) was added freshly cracked cyclopentadiene (0.54 mL, 6.4 mmol, 10 equiv) and **0.2** **M** TESOTf in DCM (0.64 mL, 0.13 mmol, 0.20 equiv) at ‒78°C. After **1** **h,** Et_3_N‐3HF (0.31 mL, 1.9 mmol, 3.0 equiv) was added. The mixture was stirred and allowed to warm to room temperature over 1 h. An aqueous solution of saturated NaHCO_3_ was added with stirring until the effervescence ceased. The resulting mixture was extracted with EtOAc three times. The combined organics were washed with brine and dried over anhydrous MgSO_4_. After concentrating *in vacuo*, the residue was purified by flash chromatography using 15% EtOAc in hexane to afford a mixture of 15c + 16c in a ratio of 1.2:1 as determined by ^1^H NMR spectroscopy, in the form of a colorless oil (52.4 mg, 55% yield). Analytically pure samples of 15c and 16c were obtained by further separation using preparative TLC (eluent: 60% DCM/ hexane), by developing the TLC plates **4** times.

15c: yellow oil; R_f _(60% DCM/ hexane) 0.63;  IR (neat) 3326, 2930, 1636, 1353, 1033, 887, 836, 787, 726 cm^−1^; ^1^H NMR (600 MHz, CDCl_3_): δ 5.90 (d, *J* = 1.9 Hz, 2H), 4.78 (d, *J* = 1.9 Hz, 1H), 4.73 (d, *J* = 1.7 Hz, 1H), 3.85 (dd, *J* = 10.8, 5.0 Hz, 1H), 3.66 (dd, *J* = 10.8, 6.5 Hz, 1H), 2.79 – 2.74 (m, 1H), 2.66 – 2.61 (m, 1H), 2.46 – 2.40 (m, 1H), 2.39 – 2.27 (m, 1H), 2.11 – 2.02 (m, 2H), 1.57 (d, *J* = 10.1 Hz, 1H) ppm; ^13^C{^1^H} NMR (150 MHz, CDCl_3_): δ 146.4, 134.8, 132.0, 110.8, 64.2, 47.8, 44.7, 41.9, 39.6, 37.0 ppm; EI‐MS (20 eV) m/z 150 (M^+^, 9), 119 (67), 117 (96), 91 (100); HRMS (EI, 40 eV) m/z [M]^+^ calcd for C_10_H_14_O 150.1039, found 150.1035.

16c: colorless oil; R_f _(60% DCM/ hexane) 0.56;  IR (neat) 3326, 2930, 1636, 1353, 1033, 887, 836, 787, 726 cm^−1^; ^1^H NMR (600 MHz, CDCl_3_): δ 5.98 (dd, 1H), 5.93 (dd, *J* = 5.7, 2.8 Hz, 1H), 4.82 (t, *J* = 2.3 Hz, 1H), 4.79 (t, *J* = 2.4 Hz, 1H), 3.69 – 3.59 (m, 2H), 2.65 – 2.59 (m, 3H), 2.40 – 2.31 (m, 2H), 2.16 – 2.10 (m, 1H), 1.76 – 1.69 (m, 1H), 1.68 (d, 1H) ppm; ^13^C{^1^H} NMR (150 MHz, CDCl_3_): δ 145.8, 134.9, 134.7, 115.5, 64.1, 48.1, 40.9, 39.2, 37.3, 35.1 ppm; EI‐MS (20 eV) m/z 150 (M^+^, 17), 119 (69), 117 (100), 91 (85);  HRMS (EI, 40 eV) m/z [M]^+^ calcd for C_10_H_14_O 150.1039, found 150.1034.

#### 4.2.2 Intramolecular (4+3) Cycloaddition of Epoxy Allylsilane 25a 

To a solution of epoxy allylsilane 25a (62.4 mg, 0.236 mmol, 1.00 equiv) in dry DCM (4.8 mL, 0.05 M) was added TESOTf (0.2 M solution in DCM, 0.24 mL, 0.05 mmol, 0.2 equiv) at ‒78°C. After **1** **h,** Et_3_N‐3HF (0.12 mL, 0.7 mmol, 3.0 equiv) was added. The mixture was stirred and allowed to warm to room temperature over 1 h. An aqueous solution of saturated NaHCO_3_ was added with stirring until the effervescence ceased. The resulting mixture was extracted with EtOAc three times. The combined organics were washed with brine and dried over anhydrous MgSO_4_. After concentrating *in vacuo*, the residue (1.7:1 mixture by ^1^H NMR spectroscopy) was purified by flash column chromatography (eluent: 20% EtOAc in hexane), to provide 26a (23.8 mg, 52% yield) and 27a (9.5 mg, 21% yield).

26a: white solid; R_f _(30% EtOAc in hexane) 0.33; IR (neat) 3393, 2922, 2851, 1643, 1450, 1351, 1225, 1160, 1064, 1030, 1000, 969, 952, 904, 883, 866, 828, 803, 739, 673, 643, 532, 452, 407 cm^−1^; ^1^H NMR (600 MHz, CDCl_3_): δ 6.11 (d, *J* = 6.0, 1.8, 0.9 Hz, 1H), 5.99 (d, *J* = 6.0 Hz, 1H), 5.16 (d, *J* = 1.7 Hz, 1H), 4.85 (d, *J* = 1.7 Hz, 1H), 4.78 (dt, *J* = 3.6, 1.7 Hz, 1H), 3.63 – 3.56 (m, 1H), 2.60 (d, *J* = 13.7, 3.4, 1.6 Hz, 1H), 2.24 (d, *J* = 10.2, 1.7 Hz, 1H), 2.14 – 2.05 (m, 1H), 2.04 (dd, *J* = 13.7, 1.6 Hz, 1H), 1.88 – 1.80 (m, 2H), 1.69 – 1.63 (m, 1H), 1.48 – 1.37 (m, 3H) ppm; ^13^C{^1^H} NMR (150 MHz, CDCl_3_): δ 143.4, 132.4, 131.3, 112.1, 87.3, 79.3, 69.3, 57.1, 39.0, 35.3, 33.2, 20.5 ppm; EI‐MS (20 eV) *m/z* 192 (M^+^, 13), 145 (100), 91 (44);  HRMS (EI, 40 eV) *m/z* [M]^+^ calcd for C_12_H_16_O_2_ 192.1145, found 192.1141; m.p. 66.5‐69.1°C.

27a: colorless oil; R_f _(30% EtOAc in hexane) 0.38; IR (neat) 3401, 2926, 2853, 1644, 1450, 1161, 1072, 1023, 986, 944, 906, 861, 817, 722, 649 cm^−1^; ^1^H NMR (600 MHz, CDCl_3_): δ 6.09 (dd, *J* = 5.9, 1.8 Hz, 1H), 5.93 (d, *J* = 5.9 Hz, 1H), 4.91 (t, *J* = 2.2 Hz, 1H), 4.89 (t, *J* = 2.3 Hz, 1H), 4.86 (dt, *J* = 3.8, 1.6 Hz, 1H), 3.84 – 3.77 (m, 1H), 2.70 – 2.64 (m, 1H), 2.15 – 2.06 (m, 1H), 2.04 (dd, *J* = 14.6, 1.4 Hz, 1H), 1.89 (d, *J* = 1.8 Hz, 1H), 1.87 – 1.81 (m, 2H), 1.75 – 1.68 (m, 3H), 1.65 – 1.60 (m, 1H) ppm; ^13^C{^1^H} NMR (150 MHz, CDCl_3_): δ 142.7, 135.9, 132.3, 116.0, 86.0, 79.8, 67.7, 55.4, 34.5, 32.6, 32.5, 19.7 ppm; EI‐MS (20 eV) *m/z* 192 (M^+^, 9), 162 (45), 133 (48), 119 (100), 91 (87);  HRMS (EI, 40 eV) *m/z* [M]^+^ calcd for C_12_H_16_O_2_ 192.1145, found 192.1147.

## Conflicts of Interest

The authors declare no conflicts of interest.

## Supporting information



Additional experimental procedures, characterizations, ^1^H and ^13^C{^1^H} NMR spectra for compounds, HPLC chromatograms, computational methods and data. **Supporting File**: asia70708‐sup‐0001‐SuppMat.pdf.

## Data Availability

The data that supports the findings of this study are available in the supplementary material of this article.
