# Supplementary material for: Inter‐and Intramolecular (4+3) Cycloadditions With Epoxy Allylsilanes as Dienophiles
Source: Chem Asian J. 2026 Mar 31;21(7):e70708. doi: 10.1002/asia.70708 (PMC13038243; doi:10.1002/asia.70708)
Supplement: Supplementary file 1 — Additional experimental procedures, characterizations, 1H and 13C{1H} NMR spectra for compounds, HPLC chromatograms, computational methods and data. Supporting File: asia70708‐sup‐0001‐SuppMat.pdf. [file ASIA-21-e70708-s001.pdf]

Supporting Information for:

Inter- and Intramolecular (4+3) Cycloadditions with Epoxy Allylsilanes as Dienophiles

Qin Han Teo,[a] Yuchen Zhou,[b] Elizabeth H. Krenske,*,[b] and Pauline Chiu*,[a]

[a] Q. H. Teo, P. Chiu
Department of Chemistry and State Key Laboratory of Synthetic Chemistry, The University of Hong Kong, Hong Kong, P.R. China.
E-mail: pchiu@hku.hk

[b] Y. Zhou, E. H. Krenske
School of Chemistry and Molecular Biosciences, The University of Queensland, St Lucia, QLD 4072, Australia
E-mail: e.krenske@uq.edu.au

Table of Contents

No	Contents	Page
1.	General Experimental	4
2.	Attempts to Induce Methylenation of (4+3) Cycloadducts	4
3.	Synthesis of epoxy allylsilane **14**	6
4.	General Procedure A: Intermolecular (4+3) Cycloadditions of **14**	7
	4.1 Reaction of epoxy allylsilane **14** with furan	8
	4.2 Reaction of epoxy allylsilane **14** with 2,5-dimethylfuran	9
	4.3 Reaction of epoxy allylsilane **14** with cyclopentadiene	9
5.	Computed TS Geometries and Energies/ Computational Methods	10
6.	Computed reaction energy profiles for intermolecular (4+3) cycloadditions	11
	6.1 Intermolecular (4+3) Cycloaddition of Activated Epoxy Enolsilane **B** with Furan	11
	6.2 Intermolecular (4+3) Cycloaddition of Epoxy Allylsilane **A** with 2,5-Dimethylfuran	14
7.	Experimental Procedures: Preparation of Epoxy Allylsilanes **(±)-25a-k**	16
	7.1 Preparation of alcohols **21a-k**	16
	7.2 Preparation of aldehydes **22a-k**	20
	7.3 Preparation of chloroaldehydes **23a-k**	24
	7.4 Preparation of epoxy allylic silanes **25a-k**	28
8.	Experimental Procedures: Preparation of Epoxy Allylsilanes **(±)-25m-r**	33
	8.1 Preparation of alkynols **40n-r**	33
	8.2 Preparation of alcohols **41n-r**	35
	8.3 Preparation of aldehydes **42m-r**	37
	8.4 Preparation of chloroaldehydes **43m-r**	39
	8.5 Preparation of epoxy allylic silanes **25m-r**	42
9.	General Procedure B for the intramolecular (4+3) cycloadditions of **(±)-25a-r**	44
	9.1 Reaction of epoxy allylsilane **25a**	45
	9.2 Reaction of epoxy allylsilane **25b**	45
	9.3 Reaction of epoxy allylsilane **25c**	46
	9.4 Reaction of epoxy allylsilane **25d**	47
	9.5 Reaction of epoxy allylsilane **25e**	47
	9.6 Reaction of epoxy allylsilane **25f**	48
	9.7 Reaction of epoxy allylsilane **25g**	48

	9.8 Reaction of epoxy allylsilane **25h**	49
	9.9 Reaction of epoxy allylsilane **25i**	50
	9.10 Reaction of epoxy allylsilane **25j**	50
	9.11 Reaction of epoxy allylsilane **25k**	51
	9.12 Reaction of epoxy allylsilane **25m**	51
	9.13 Reaction of epoxy allylsilane **25n**	52
	9.14 Reaction of epoxy allylsilane **25o**	52
	9.15 Reaction of epoxy allylsilane **25p**	53
	9.16 Reaction of epoxy allylsilane **25q**	53
	9.17 Reaction of epoxy allylsilane **25r**	54
10.	Proposed mechanism accounting for the formation of **30d** and **30e**	55
11.	Computed TS Geometries and Energies/ Computational Methods	56
	11.1 Intramolecular (4+3) Cycloaddition of Epoxy Allylsilane **C**	56
	11.2 Intramolecular (4+3) Cycloaddition of Epoxy Enolsilane **D**	58
12.	Asymmetric (4+3) cycloaddition of epoxy allylsilane **(+)-25a**	60
	12.1 Preparation of chloroaldehyde **(+)-23a**	60
	12.2 Preparation of epoxy allylsilane **(+)-25a**	60
	12.3 Reaction of epoxy allylsilane **(+)-25a**	61
13.	Studies on the (3+2) cycloaddition of epoxy allylsilane **25l**	62
	13.1 Synthetic route for the synthesis of epoxy allylsilane **25l**	62
	13.2 Preparation of chloroaldehyde **48**	62
	13.3 Preparation of epoxy allylsilane **25l**	63
	13.4 Reaction of epoxy allylsilane **25l**	63
14.	References	64
15.	^{1}H and ^{13}C-NMR spectra of isolated compounds	69
16.	HPLC Chromatograms of **(+)-23a**, **(+)-26a**, and **(–)-27a**	166
17.	Computations: Molecular Coordinates Summary	169

1. General Experimental

Preparative:

All anhydrous reactions were performed in oven-dried round-bottomed flasks under a positive pressure of dry argon. Air and moisture-sensitive compounds were introduced via syringes or cannula using standard inert atmosphere techniques. Reactions were monitored by thin layer chromatography (TLC) using E. Merck silica gel plates, Kieselgel 60 F_{254} with 0.2 mm thickness. Components were visualized by illumination with short-wavelength ultra-violet light and/or staining with $KMnO_4$, vanillin, or phosphomolybdic acid (PMA). Flash column chromatography was performed with E. Merck silica gel 60 (230-400 mesh ASTM).

Solvents and chemicals were purified according to standard procedures. All anhydrous solvents used for reactions were distilled or dried by passing through drying columns. Dichloromethane (DCM), dimethylformamide (DMF), furan, triethylamine (Et_3N), and tetrahydrofuran (THF) were distilled from CaH_2 under argon. Cyclopentadiene was obtained from cracking commercially available dicyclopentadiene. In particular, the solvents used in all (4+3) cycloadditions were distilled from CaH_2 under argon, then dried over 4 Å MS. Other reagents were used as received.

Analytical:

^1H and ^{13}C NMR nuclear magnetic resonance spectra were recorded in deuterochloroform ($CDCl_3$) with tetramethylsilane (TMS) as an internal standard at ambient temperature (unless otherwise specified) on a Bruker Avance 400 spectrometer, Bruker DX 500 spectrometer, or Bruker Avance 600 operating at 400 MHz, 500 MHz or 600 MHz respectively for ^1H; 100 MHz, 125 MHz or 150 MHz respectively for ^{13}C, and at 376 MHz, 470 MHz or 564 MHz for ^{19}F. All spectra were calibrated using the solvent resonance internal standard at δ 7.26 ppm for ^1H spectra (residual $CHCl_3$), and δ 77.16 ppm for ^{13}C spectra. Splitting patterns are designated as follows: s = singlet, d = doublet, t = triplet, q = quartet, m = multiplet, br = broad. IR absorption spectra were recorded on a PerkinElmer-Spectrum Two FT-IR Spectrometer from 4000 cm^{-1} to 400 cm^{-1}. Electron impact mass spectrometry was recorded on a Finnigan MAT 95 mass spectrometer or API QSTAR PULSAR iLC/MS/TOF System for both low resolution and high resolution, with accurate mass reported for the molecular ion (M^+) or next largest fragment thereof. Analytical HPLC was carried out on an Agilent 1260 Infinity II LC system equipped with a G7111A quaternary pump, a G7129A vial sampler with integrated column compartment G7130A, and a variable wavelength G7114B detector operating with Agilent OpenLab CDS ChemStation Edition Rev. 2.4 software. Optical rotations were recorded as solutions in $CHCl_3$ on a Bellingham & Stanley ADP440+ polarimeter.

2. Attempts to Induce Methylenation of (4+3) Cycloadducts

The conversion of several cycloheptenones obtained from the (4+3) cycloadditions of epoxy enolsilanes to their methylenated congeners was examined.

Using a typical protected (4+3) cycloadduct, cycloheptenone **33**, the Wittig reaction generated trace amounts of methylenated **34** (**Scheme S1, a**). This is presumably because of the steric demands of **33**, as well as the bulkiness of the Wittig reagent. More success was found by

subjecting **33** to Peterson olefination conditions (**Scheme S1, b**), which generated **34**, albeit in just 33% overall yield. The problematic step is the addition to the carbonyl group which proceeded sluggishly. Similarly, addition of $TMSCH_2Li$ to intramolecular (4+3) cycloadduct **35** and its protected derivative **36**, also proceeded in low yields. These experimental findings reinforced that direct methylenation of the products after cycloaddition is not a smooth or general process, as these polycyclic ketones are quite sterically demanding.

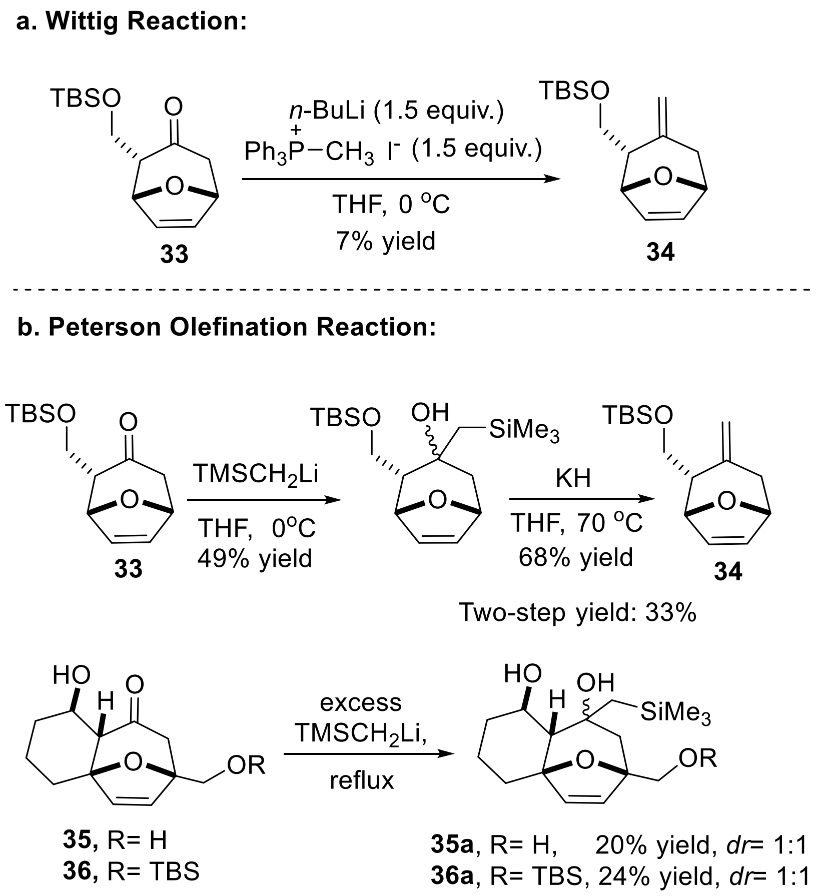

Scheme S1. Methylenation attempts on the epoxy enolsilane derived cycloadducts **33, 35,** and **36**.

3. Synthesis of epoxy allylsilane 14:

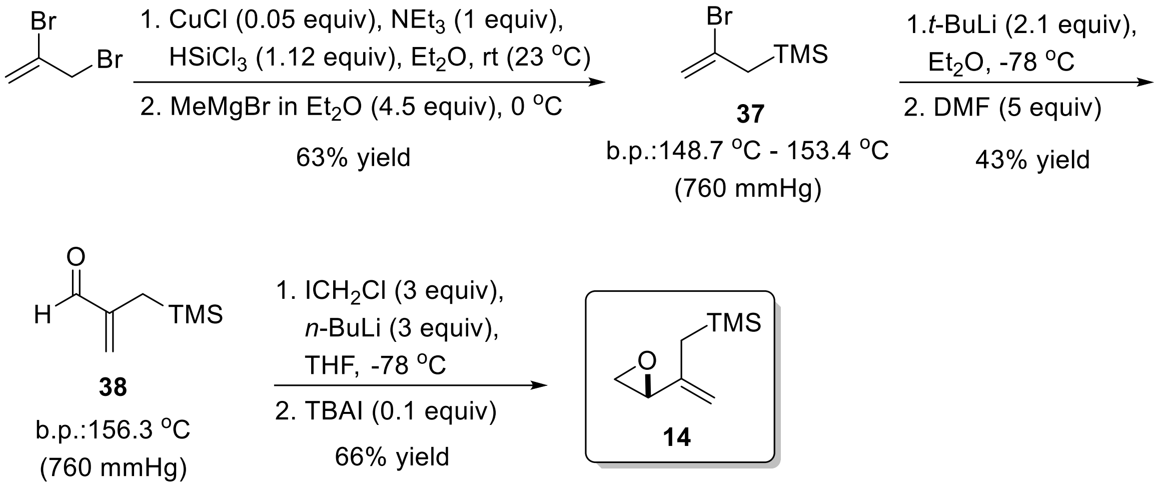

(2-Bromoallyl)trimethylsilane (37)

Compound **37** was synthesized following a literature procedure,[1] using 2,3-dibromopropene (6.5 mL, 67 mmol, 1.0 equiv), CuCl (0.330 g, 3.33 mmol, 0.05 equiv), NEt_3 (9.4 mL, 67 mmol, 1.0 equiv), $HSiCl_3$ (7.6 mL, 75 mmol, 1.1 equiv), MeMgBr (3M in Et_2O, 100 mL, 300 mmol, 4.5 equiv), and Et_2O (55 mL). Compound **37** was obtained as a colorless oil (7.98 g, 41.3 mmol, 62% yield).

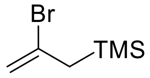

37

37: R_f (10% EtOAc in hexane) 0.93; ^{1}H NMR (500 MHz, $CDCl_3$): δ 5.31 (s, 1H), 5.22 (s, 1H), 2.11 (s, 2H), 0.11 (s, 9H) ppm; ^{13}C{^{1}H}NMR (125 MHz, $CDCl_3$): δ 131.3, 114.1, 33.5, -1.4 ppm. The spectral characteristics are consistent with those of **37** reported in the literature.[1]

2-((Trimethylsilyl)methyl)acrylaldehyde (38)

To a solution of **37** (3.00 g, 15.5 mmol, 1.00 equiv) in anhydrous Et_2O (50 mL, 0.3 M) at -78 °C was added *tert*-butyllithium (26 mL, 33 mmol, 2.1 equiv) in a dropwise manner. The resulting mixture was stirred for 30 min at -78 °C, and neat DMF (6.0 mL, 78 mmol, 5.0 equiv) was introduced at a rate of 0.5 mL/min. The resultant solution was allowed to warm to room temperature. The reaction mixture was quenched with saturated NH_4Cl, and the organic layer was separated. The aqueous layer was back-extracted with Et_2O three times. The combined organics were washed with saturated $NaHCO_3$, water, brine, and dried over anhydrous $MgSO_4$. The volatiles were removed *in vacuo*, and the residue was distilled under reduced pressure (18 mmHg, 50 °C) to give **38** (0.95 g, 6.7 mmol, 43% yield) as a colorless oil.

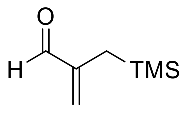

38

38: R_f (1% EtOAc in hexane) 0.28; ^{1}H NMR (500 MHz, $CDCl_3$): δ 9.47 (s, 1H), 6.08 (d, J = 1.1 Hz, 1H), 5.84 (d, J = 0.9 Hz, 1H), 1.76 (s, 2H), -0.02 (s, 9H) ppm; ^{13}C{^{1}H} NMR (100 MHz, $CDCl_3$): δ 194.7, 148.5, 131.6, 18.1, -1.7 ppm. The spectral characteristics are consistent with those of **38** reported in the literature. [2]

Trimethyl(2-(oxiran-2-yl)allyl)silane (14)

Compound **14** was synthesized according to a literature procedure,[3] using **38** (0.63 g, 4.4 mmol, 1.0 equiv), THF (14 mL, 0.32 M), ICH_2Cl (0.97 mL, 13 mmol, 3.0 equiv), *n*-BuLi (1.7 M in hexanes, 7.9 mL, 13 mmol, 3.0 equiv), and TBAI (0.164 g, 0.440 mmol, 0.100 equiv). Compound **14** was obtained as a pale-yellow oil (0.470 g, 3.01 mmol, 68% yield).

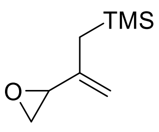

14

14: R_f (2% Et_2O in hexane) 0.36; ^1H NMR (500 MHz, $CDCl_3$): δ 5.00 (s, 1H), 4.75 (s, 1H), 3.26 (t, 1H), 2.86 (dd, J = 5.6, 4.0 Hz, 1H), 2.59 (dd, J = 5.7, 2.7 Hz, 1H), 1.51 – 1.39 (m, 2H), 0.04 (s, 9H) ppm; $^{13}C\{^1H\}$ NMR (100 MHz, $CDCl_3$): δ 143.5, 109.8, 54.3, 48.2, 21.3, -1.3 ppm. The spectral characteristics are consistent with those of **14** in the literature.[3]

4. General Procedure A: Intermolecular (4+3) cycloadditions of 14

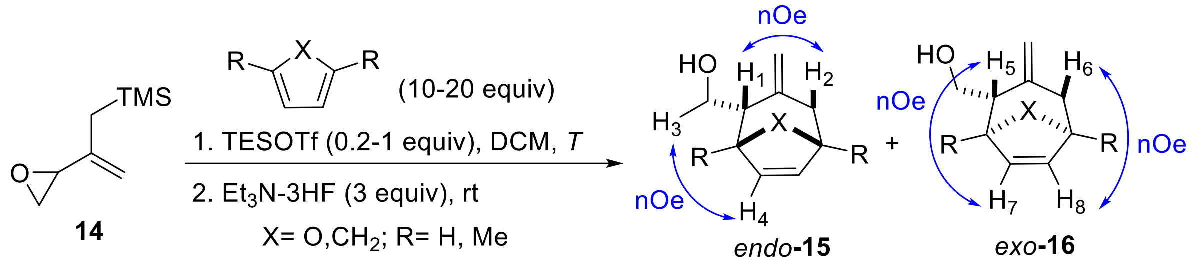

To a solution of epoxy allylsilane **14** (1 equiv) in dry DCM (0.2 M) was added dienes (10-20 equiv) and TESOTf (0.2 M solution in DCM, 0.2-1 equiv) at –94°C or –78 °C. The reaction progress was monitored by TLC. When the reaction is complete, Et_3N-3HF (3 equiv) was added. The mixture was allowed to warm to room temperature with stirring for 1 h. An aqueous solution of saturated $NaHCO_3$ was added with stirring until the effervescence ceased. The resulting mixture was extracted with EtOAc three times. The combined organics were washed with brine and dried over anhydrous $MgSO_4$. The crude product was concentrated *in vacuo* and then purified by flash chromatography to afford the products.

4.1 Reaction of epoxy allylsilane 14 with furan

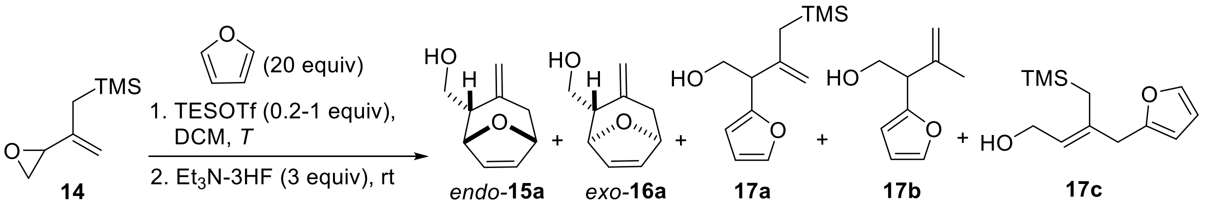

According to general procedure A, treatment of **14** (45 mg, 0.29 mmol, 1.0 equiv) in DCM (1.5 mL, 0.20 M) with furan (0.42 mL, 5.8 mmol, 20 equiv) and 0.2 M TESOTf in DCM (1.5 mL, 0.29 mmol, 1.0 equiv) at –94°C afforded, after flash column chromatography using 20% EtOAc in hexane, a mixture of **15a+16a** in a ratio of 1:1.6 (18.9 mg, 0.124 mmol, 43% yield) as determined by ^1H NMR spectroscopy, and **17b** (6.2 mg, 0.040 mmol, 14% yield). Analytically pure samples of **15a** and **16a** obtained by further separation using preparative TLC (eluent: 5% Et_2O/ DCM) by developing the TLC plates 4 times.

According to general procedure A, treatment of **14** (53 mg, 0.34 mmol, 1.0 equiv) in DCM (1.4 mL, 0.20 M) with furan (0.49 mL, 6.8 mmol, 20 equiv) and 0.2 M TESOTf in DCM (0.3 mL, 0.07 mmol, 0.2 equiv) at –78°C afforded, after flash column chromatography using

20% EtOAc in hexane, a mixture of **15a+16a** in a ratio of 1:1.9 (15.4 mg, 0.101 mmol, 30% yield) as determined by ^{1}H NMR spectroscopy, **17a** (9.3 mg, 0.041 mmol, 12% yield), and **17c** (5.6 mg, 0.024 mmol, 7% yield). Analytically pure samples of **15a** and **16a** obtained by further separation using preparative TLC (eluent: 5% Et_2O/ DCM) by developing the TLC plates 4 times.

endo-**15a**: Pale yellow oil; R_f (5% Et_2O/ DCM) 0.26; IR (neat) 3385, 2931, 1643, 1425, 1340, 1313, 1236, 1170, 1057, 1030, 1000, 975, 957, 896, 877, 860, 842, 827, 809, 771, 662, 554 cm^{-1}; ^{1}H NMR (600 MHz, $CDCl_3$): δ 6.20 (dd, *J* = 6.1, 1.8 Hz, 1H), 6.15 (dd, *J* = 6.1, 1.7 Hz, 1H), 4.88 (dd, *J* = 3.9, 1.8 Hz, 1H), 4.84 (s, 1H), 4.82 – 4.76 (m, 2H), 3.88 (dd, *J* = 11.2, 4.7 Hz, 1H), 3.70 (dd, *J* = 11.1, 6.9 Hz, 1H), 2.77 (dt, *J* = 6.9, 2.4 Hz, 1H), 2.61 (ddt, *J* = 14.4, 3.9, 1.9 Hz, 1H), 2.02 (d, *J* = 14.4 Hz, 1H) ppm; ^{13}C{^{1}H} NMR (150 MHz, $CDCl_3$): δ 142.4, 132.3, 130.7, 111.8, 79.9, 78.9, 61.3, 47.7, 36.2 ppm; EI-MS (20 eV) m/z 152 (M^+, 2), 134 (M^+-H_2O, 100), 133 (40); HRMS (EI, 40eV) m/z $[M]^+$ calcd for $C_9H_{12}O_2$ 152.0832, found 152.0834.

exo-**16a:** Colorless oil; R_f (5% Et_2O/ DCM) 0.57; IR (neat) 3389, 2921, 1639, 1341, 1029, 970, 881, 806, 706 cm^{-1}; ^{1}H NMR (600 MHz, $CDCl_3$): δ 6.20 (dd, *J* = 6.1, 1.8 Hz, 1H), 6.15 (dd, *J* = 6.1, 1.7 Hz, 1H), 4.89 (d, *J* = 2.2 Hz, 1H), 4.87 (d, *J* = 2.5 Hz, 1H), 4.81 (d, *J* = 4.2 Hz, 1H), 4.78 (s, 1H), 3.88 – 3.79 (m, 2H), 2.72 – 2.65 (m, 1H), 2.26 (t, *J* = 6.8 Hz, 1H), 2.07 (d, *J* = 14.8 Hz, 1H) ppm; ^{13}C{^{1}H} NMR (150 MHz, $CDCl_3$): δ 141.6, 132.4, 132.2, 116.3, 79.7, 78.8, 64.2, 48.4, 35.2 ppm; EI-MS (20 eV) m/z 152 (M^+,2), 134 (M^+-H_2O, 99), 119 (58); HRMS (EI, 40eV) m/z $[M]^+$ calcd for $C_9H_{12}O_2$ 152.0832, found 152.0834.

2-(Furan-2-yl)-3-((trimethylsilyl)methyl)but-3-en-1-ol (17a): colorless oil; R_f (30% EtOAc / hexane) 0.67; IR (neat) 3418, 2953, 2897, 1726, 1716, 1163, 853 cm^{-1}; ^{1}H NMR (500 MHz, $CDCl_3$): δ 7.35 (dd, *J* = 1.9, 0.8 Hz, 1H), 6.32 (dd, *J* = 3.2, 1.9 Hz, 1H), 6.17 (d, *J* = 3.2 Hz, 1H), 4.81 (d, *J* = 1.1 Hz, 1H), 4.74 (d, *J* = 1.1 Hz, 1H), 3.93 – 3.86 (m, 2H), 3.49 (t, *J* = 6.6 Hz, 1H), 1.57 (s, 2H), 0.03 (s, 9H) ppm; ^{13}C{^{1}H} NMR (100 MHz, $CDCl_3$): δ 154.6, 144.8, 141.8, 110.3, 110.0, 106.9, 63.4, 49.6, 26.4, -1.3 ppm; ESI-HRMS *m/z* calcd for $C_{12}H_{20}OSi$ $[M+H]^+$: 225.1305, found 225.1302.

2-(Furan-2-yl)-3-methylbut-3-en-1-ol (17b): pale-yellow oil; R_f (30% EtOAc / hexane) 0.53; IR (neat) 3433, 2953, 1651, 1377, 1165, 847 cm^{-1}; ^{1}H NMR (400 MHz, $CDCl_3$): δ 7.36 (d, *J* = 1.8 Hz, 1H), 6.33 (dd, *J* = 3.3, 1.9 Hz, 1H), 6.13 (d, *J* = 3.2 Hz, 1H), 5.00 (s, 1H), 4.91 (s, 1H), 3.99 – 3.85 (m, 2H), 3.64 (t, *J* = 7.0 Hz, 1H), 1.72 (s, 3H) ppm; ^{13}C{^{1}H} NMR (100 MHz, $CDCl_3$): δ 154.2, 143.0, 141.8, 114.0, 110.3, 106.4, 62.7, 49.5, 20.7 ppm; ESI-HRMS *m/z* calcd for $C_9H_{12}O_2$ $[M+H]^+$: 153.0910, found 153.0904.

4-(Furan-2-yl)-3-((trimethylsilyl)methyl)but-2-en-1-ol (17c): pale yellow oil; R_f (15% EtOAc / hexane) 0.65; IR (neat) 3416, 2953, 2920, 2854, 1247 cm^{-1}; ^{1}H NMR (400 MHz, $CDCl_3$): δ 7.32 (s, 1H), 6.30 (s, 1H), 6.05 (s, 1H), 5.33 (t, *J* = 6.9 Hz, 1H), 4.10 (d, *J* = 6.8 Hz, 2H), 3.29 (s, 2H), 1.59 (s, 2H), 0.05 (s, 9H) ppm; ^{13}C{^{1}H} NMR (100 MHz, $CDCl_3$): δ 153.6, 141.5, 122.9, 114.0, 110.4, 106.9, 62.7, 38.2, 21.8, -0.8 ppm; ESI-HRMS *m/z* calcd for $C_{12}H_{20}O_2Si$ $[M]^+$: 224.1227, found 224.1230.

4.2 Reaction of epoxy allylsilane **14** with 2,5-dimethylfuran

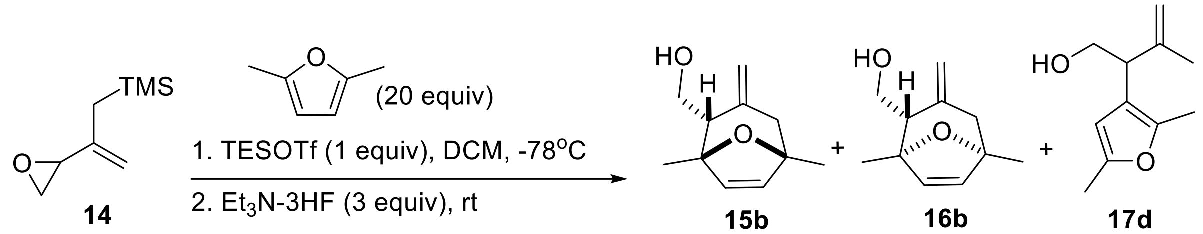

According to General Procedure A, treatment of **14** (50 mg, 0.32 mmol, 1.0 equiv) in DCM (1.6 mL, 0.2 M) with 2,5-dimethylfuran (0.69 mL, 6.4 mmol, 20 equiv) and 0.2M TESOTf in DCM (1.6 mL, 0.32 mmol, 1 equiv) afforded, after flash column chromatography using 30% EtOAc in hexane, a 6.7:1 mixture of **15b** + **16b** as determined by ^{1}H NMR spectroscopy, in the form of a pale yellow oil (22.1 mg, 0.123 mmol, 38% yield) and **17d** (2.3 mg, 0.013 mmol, 4% yield). Compounds **15b** and **16b** cannot be separated.

Mixture of 15b + 16b: R$_f$ (30% EtOAc in hexane) 0.38; IR (neat) 3414, 2971, 2929, 2888, 1642, 1446, 1375, 1341, 1172, 1135, 1084, 1014, 951, 932, 892, 862, 823, 743, 649 cm^{-1}; ^{1}H NMR (400 MHz, $CDCl_3$): Major cycloadduct **15b**: δ 5.93 (d, *J* = 5.8 Hz, 1H), 5.79 (d, *J* = 5.8 Hz, 1H), 4.91 (dt, *J* = 13.6, 2.1, 0.8 Hz, 2H), 4.10 (dt, *J* = 12.1, 2.4 Hz, 1H), 3.79 (ddd, *J* = 11.9, 9.2, 3.9 Hz, 1H), 2.44 – 2.35 (m, 2H), 2.14 (dd, *J* = 14.3, 2.9 Hz, 1H), 1.50 (s, 3H), 1.39 (s, 3H) ppm; ^{13}C{^{1}H} NMR (100 MHz, $CDCl_3$): Major cycloadduct **15b**: δ 144.2, 135.4, 134.3, 110.8, 86.5, 84.7, 59.5, 52.5, 42.8, 23.7, 22.0 ppm; EI-MS (20 eV) m/z 162 (M^+-H_2O, 100),147 (97), 119 (95), 107 (80), 95 (85), 91 (84); HRMS (EI, 40eV) m/z $[M]^+$ calcd for $C_{11}H_{16}O_2$ 180.1145, found 180.1141.

2-(2,5-Dimethylfuran-3-yl)-3-methylbut-3-en-1-ol (17d): Pale yellow oil; R$_f$ (15% EtOAc / hexane) 0.32; IR (neat) 3416, 2922, 2884, 1645, 1435, 1040, 891 cm^{-1}; ^{1}H NMR (500 MHz, $CDCl_3$): δ 5.78 (s, 1H), 4.89 (s, 1H), 4.83 (s, 1H), 3.89 – 3.82 (m, 1H), 3.74 – 3.67 (m, 1H), 3.28 (t, *J* = 7.4 Hz, 1H), 2.21 (d, *J* = 6.8 Hz, 6H), 1.68 (s, 3H) ppm; ^{13}C{^{1}H} NMR (125 MHz, $CDCl_3$): δ 149.9, 147.2, 145.2, 118.1, 111.3, 105.6, 64.0, 46.0, 21.6, 13.6, 11.7 ppm; ESI-HRMS *m/z* calcd for $C_{11}H_{16}O_2$ $[M]^+$: 180.1145, found 180.1147.

4.3 Reaction of epoxy allylsilane **14** with cyclopentadiene

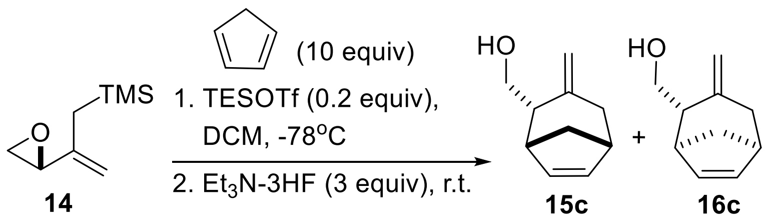

According to General Procedure A, treatment of **14** (0.10 g, 0.64 mmol, 1.0 equiv) in DCM (6.4 mL, 0.1 M) with freshly cracked cyclopentadiene (0.54 mL, 6.4 mmol, 10 equiv) and 0.2M TESOTf in DCM (0.64 mL, 0.13 mmol, 0.20 equiv) afforded, after flash column chromatography using 15% EtOAc in hexane, a mixture of **15c** + **16c** in a ratio of 1.2:1 as determined by ^{1}H NMR spectroscopy, in the form of a colorless oil (52.4 mg, 0.349 mmol, 55% yield). Analytically pure samples of **15c** and **16c** were obtained by further separation using preparative TLC (eluent: 60% DCM/ hexane) by developing the TLC plates 4 times.

15c: yellow oil; R$_f$ (60% DCM/ hexane) 0.63; IR (neat) 3326, 2930, 1636, 1353, 1033, 887, 836, 787, 726 cm^{-1}; ^{1}H NMR (600 MHz, $CDCl_3$): δ 5.90 (d, *J* = 1.9 Hz, 2H), 4.78 (d, *J* = 1.9

Hz, 1H), 4.73 (d, J = 1.7 Hz, 1H), 3.85 (dd, J = 10.8, 5.0 Hz, 1H), 3.66 (dd, J = 10.8, 6.5 Hz, 1H), 2.79 – 2.74 (m, 1H), 2.66 – 2.61 (m, 1H), 2.46 – 2.40 (m, 1H), 2.39 – 2.27 (m, 1H), 2.11 – 2.02 (m, 2H), 1.57 (d, J = 10.1 Hz, 1H) ppm; $^{13}C\{^1H\}$ NMR (150 MHz, $CDCl_3$): δ 146.4, 134.8, 132.0, 110.8, 64.2, 47.8, 44.7, 41.9, 39.6, 37.0 ppm; EI-MS (20 eV) m/z 150 (M^+, 9), 119 (67), 117 (96), 91 (100); HRMS (EI, 40eV) m/z $[M]^+$ calcd for $C_{10}H_{14}O$ 150.1039, found 150.1035.

16c: colorless oil; R_f (60% DCM/ hexane) 0.56; IR (neat) 3326, 2930, 1636, 1353, 1033, 887, 836, 787, 726 cm^{-1}; ^1H NMR (600 MHz, $CDCl_3$): δ 5.98 (dd, 1H), 5.93 (dd, J = 5.7, 2.8 Hz, 1H), 4.82 (t, J = 2.3 Hz, 1H), 4.79 (t, J = 2.4 Hz, 1H), 3.69 – 3.59 (m, 2H), 2.65 – 2.59 (m, 3H), 2.40 – 2.31 (m, 2H), 2.16 – 2.10 (m, 1H), 1.76 – 1.69 (m, 1H), 1.68 (d, 1H) ppm; $^{13}C\{^1H\}$ NMR (150 MHz, $CDCl_3$): δ 145.8, 134.9, 134.7, 115.5, 64.1, 48.1, 40.9, 39.2, 37.3, 35.1 ppm; EI-MS (20 eV) m/z 150 (M^+, 17), 119 (69), 117 (100), 91 (85); HRMS (EI, 40eV) m/z $[M]^+$ calcd for $C_{10}H_{14}O$ 150.1039, found 150.1034.

*Unsymmetrical dienes were not tried due to their tendency to yield regioisomers.

5. Computed TS Geometries and Energies/ Computational Methods

Computational Methods

Density functional theory computations were performed in Gaussian 16.[4] Transition state, reactant and product geometries were optimized with B3LYP-D3(BJ)/6-31G(d,p) using the CPCM implicit solvent model of dichloromethane.[5-14] Vibrational frequency calculations at this level gave thermochemical quantities. Entropies and vibrational energies were calculated using Shermo 2.6 with the quasi-RRHO model developed by Grimme and Minenkov.[15-17] Single-point energies were calculated with M06-2X/def2-TZVPP using the SMD implicit solvent model of dichloromethane.[18-20] Gibbs free energies were calculated by adding the quasi-harmonically corrected B3LYP thermochemical quantities to the M06-2X single-point energy, and are reported at a standard state of 298.15 K and 1 mol/L. IGMH weak interaction analysis and localized orbital locator analysis were conducted with Multiwfn 3.8 dev.[21-24]

The coordinates for each optimized TS are listed below, along with the following energies (all in Hartree):

E_{B3LYP} = B3LYP electronic potential energy plus solvation energy in CPCM implicit dichloromethane

G_{B3LYP} = B3LYP Gibbs free energy (quasi-harmonically corrected) direct from calculation in CPCM implicit dichloromethane at 298.15 K

$E_{M06\text{-}2X}$ = M06-2X single-point electronic potential energy in SMD implicit dichloromethane

G_{tot} = total M06-2X//B3LYP Gibbs free energy in dichloromethane at 298.15 K and 1 mol/L

6. Computed reaction energy profiles for intermolecular (4+3) cycloadditions

6.1 Intermolecular (4+3) Cycloaddition of Activated Epoxy Enolsilane **B** with Furan

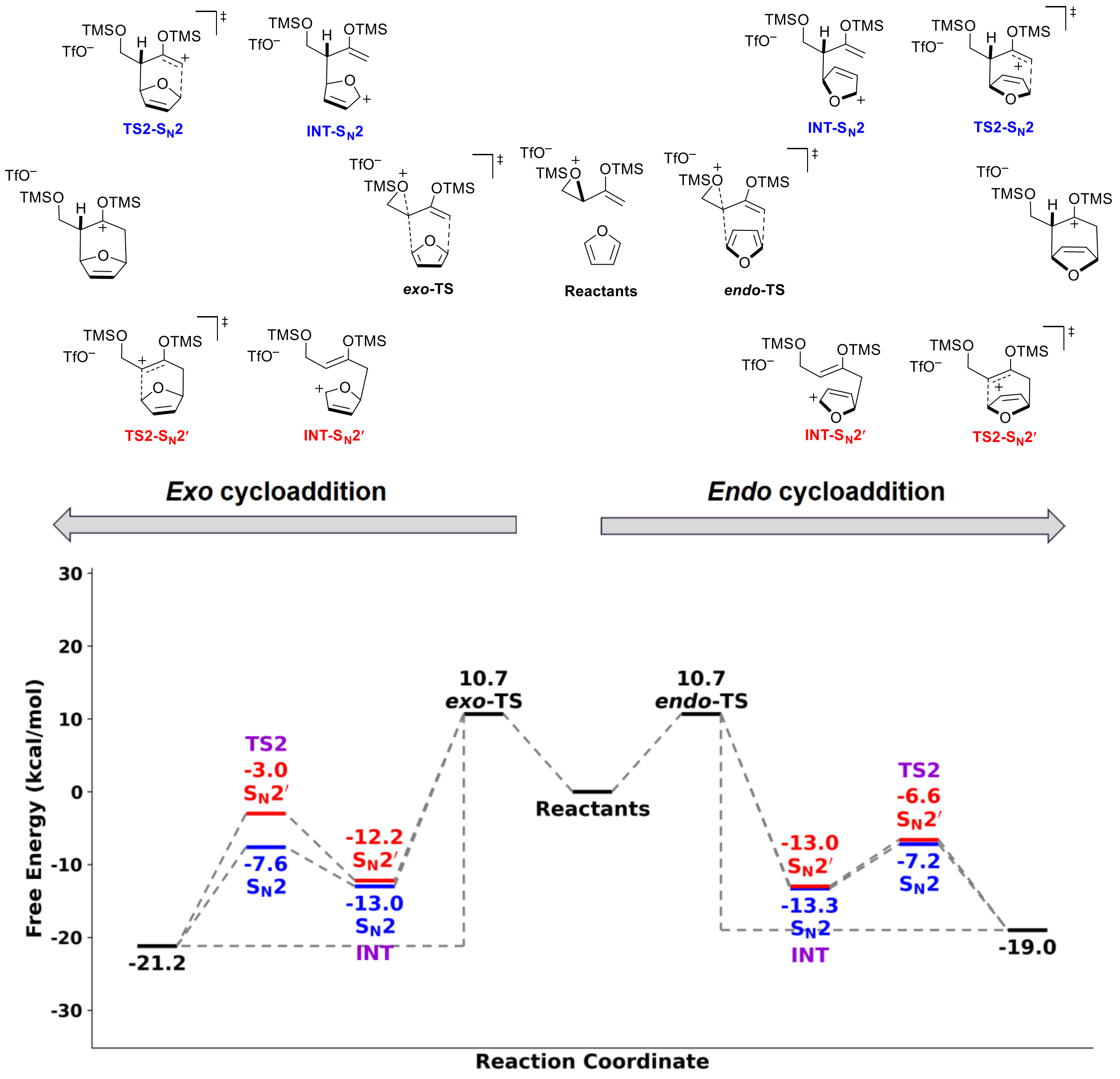

Scheme S2. Free Energy Profile for the intermolecular (4+3) cycloaddition of furan with activated enolsilane **B**. There are three possible pathways the reaction could follow for the attack by the diene: concerted formation of both new C–C bonds leading directly to the cycloadduct, or stepwise formation of one C–C bond followed by the other via an intermediate. There are two stepwise pathways depending on which C–C bond forms first.

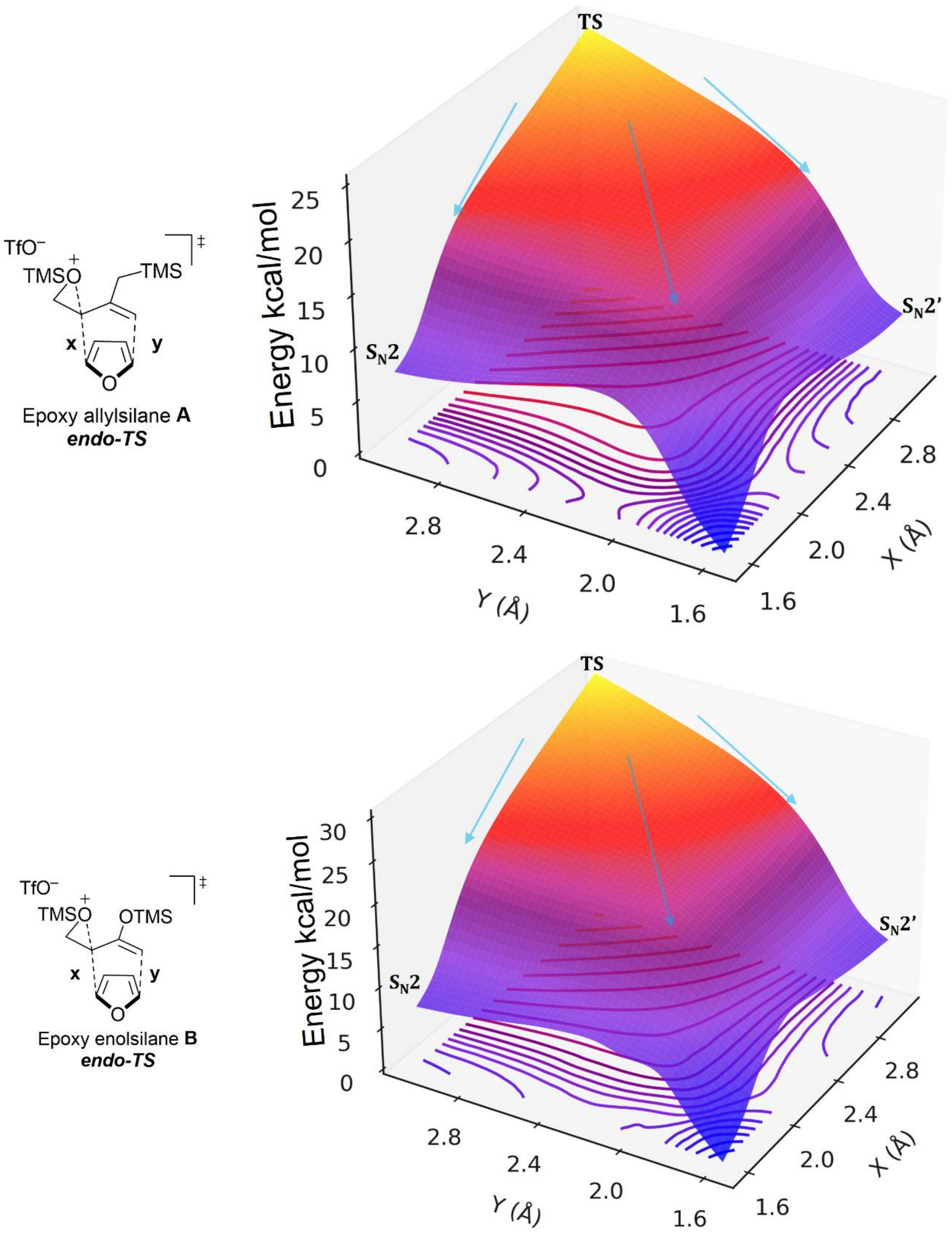

Scheme S3. Potential energy surfaces obtained by relaxed scans of bonds **x** and **y** of the *endo* reaction between furan and (a) epoxy allylsilane **A** or (b) epoxy enolsilane **B**. The calculations were conducted at the B3LYP-D3(BJ)/6-31G(d,p) level of theory with the CPCM implicit solvent model of dichloromethane.

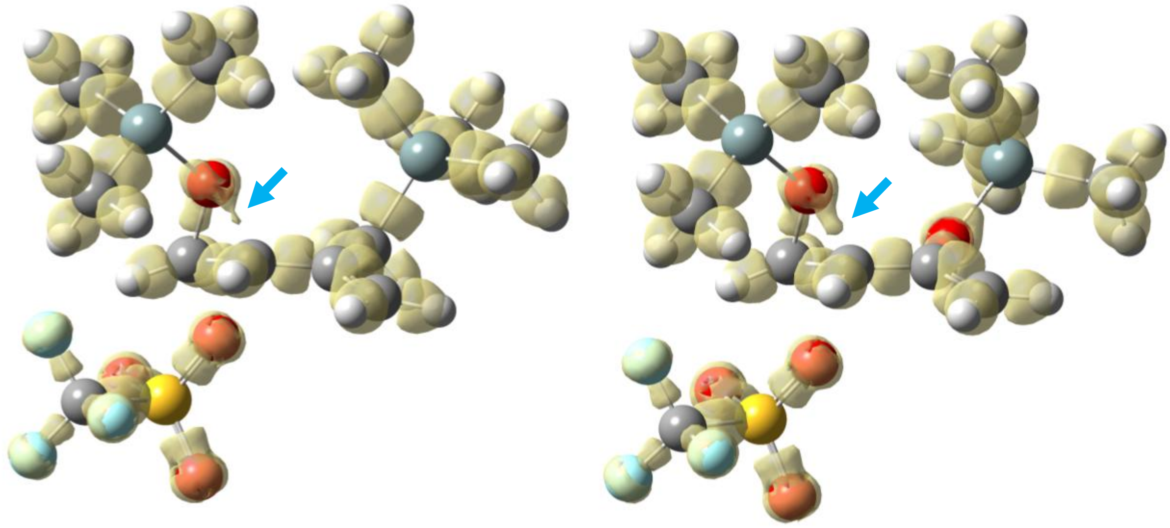

Figure S1. Localized orbital locator (LOL) analysis of epoxy allylsilane **A** and epoxy enolsilane **B**. The existence of enol oxygen in **B** induces a more localized epoxy C-O bond.

6.2 Intermolecular (4+3) Cycloaddition of Epoxy Allylsilane **A** with 2,5-Dimethylfuran

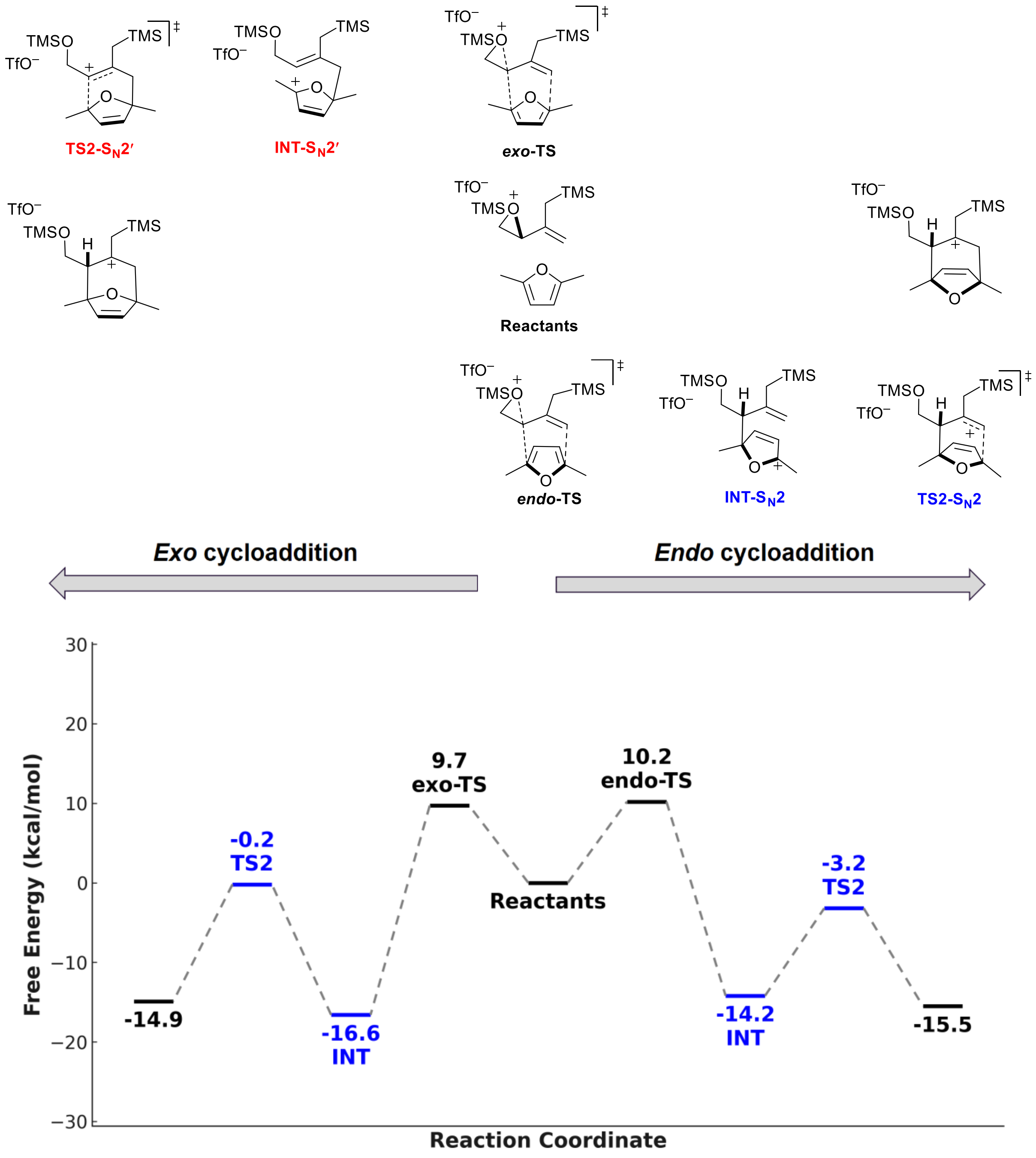

Scheme S4. Free energy profile for the intermolecular (4+3) cycloaddition of 2,5-dimethylfuran with epoxy allylsilane **A**.

The reaction between epoxy allylsilane **A** and 2,5-dimethylfuran displays several differences compared to the reaction with furan **(Scheme S4)**. Firstly, for the *endo* cycloaddition, only the intermediate corresponding to formation of the S_N2 C–C bond could be located. Secondly, for the *exo* cycloaddition, only the intermediate corresponding to the formation of the S_N2' C–C bond could be located. The first step in the *exo* cycloaddition (which may lead either to a stepwise intermediate or to the cycloadduct) is favoured by 0.5 kcal/mol

relative to that for the *endo* cycloaddition. This selectivity is similar to that observed for furan (0.6 kcal/mol). However, unlike the reaction of furan, the barriers for the ring closure steps for 2,5-dimethylfuran in either the *endo* or *exo* mode are relatively high. Between these two ring closures, the barrier for the *endo* process is 5.8 kcal/mol lower than for *exo*. Furthermore, in the *exo* cycloaddition, the intermediate is 1.7 kcal/mol thermodynamically more stable than the cycloadduct. This suggests that the relatively high *dr* obtained experimentally (*endo*/*exo* = 6.7:1) might result, at least in part, from the *exo* intermediate undergoing alternative reactions rather than ring closure. An investigation of the non-covalent interactions with the IGMH analytical method **(Figure S2)** reveals a pronounced steric clash between the methyl substituent of the furan and the ring-opened epoxy fragment in the *exo* cycloadduct, suggesting a possible explanation for the relatively low stability of this compound.

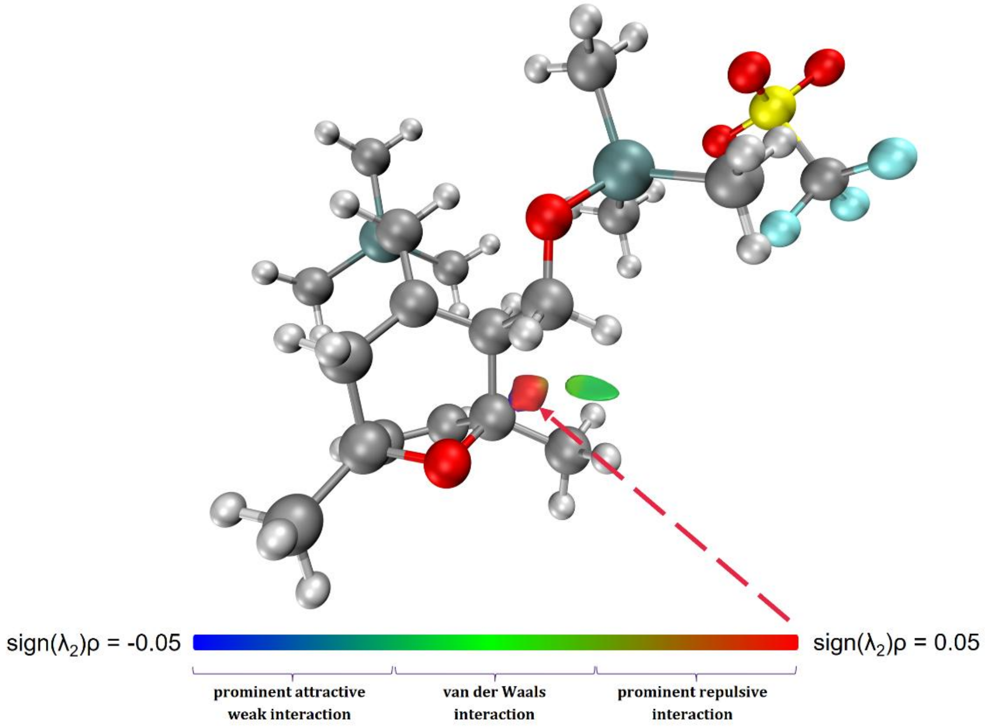

Figure S2. IGMH weak interaction analysis showing the interactions between the 2-methyl group of the 2,5-dimethylfuran and the ring-opened epoxy methylene moiety of allylsilane **A**. The green surfaces represent regions possessing stabilizing van der Waals interactions whereas the red surfaces represent repulsion. A pronounced steric clash can be observed between these two moieties.

7. Experimental Procedures: Preparation of Epoxy Allylsilanes (±)-25a-k

General synthetic route to the synthesis of epoxy allylsilanes **(±)-25a-k**

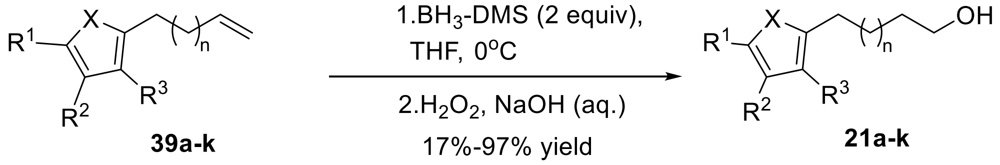

Compound	X	R^1	R^2	R^3	n
**39a**	O	H	H	H	2
**39b**	O	Me	H	H	2
**39c**	O	$CH_2OTBDPS$	H	H	2
**39d**	O	H	H	Me	2
**39e**	O	H	H	H	1
**39f**	O	Me	H	H	1
**39g**	*N*Boc	H	H	H	2
**39h**	*N*Ts	H	H	H	2
**39i**	S	H	H	H	2
**39j**	S	H	H	Me	2
**39k**	S	H	Me	Me	2

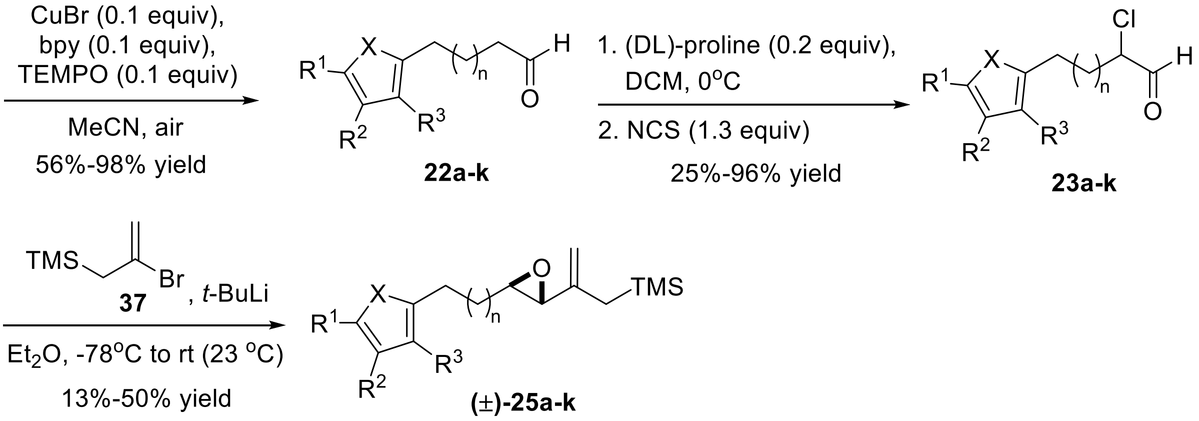

The preparations of furans **39a, 39b,**[25] **39c,**[26] **39e,**[27] **39f,**[28] have been reported.

The preparations of pyrroles **39g**, **h** from 2-(pent-4-en-1-yl)-1*H*-pyrrole have been reported.[29]

2-(Pent-4-en-1-yl)thiophene (**39i**),[30] 3-methyl-2-(pent-4-en-1-yl)thiophene (**39j**) and 3,4-dimethyl-2-(pent-4-en-1-yl)thiophene (**39k**) were prepared according to the literature.[31]

7.1 Preparation of alcohols **21a-k**

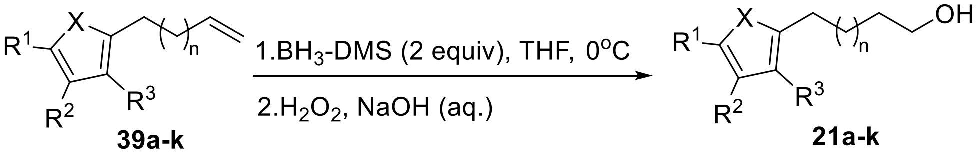

The preparation of furan **21b** has been reported in the literature.[28]

5-(Furan-2-yl)pentan-1-ol (21a)

According to the literature procedure for preparing **21b**,[28] **39a** (1.22 g, 8.97 mmol, 1.00 equiv) in anhydrous THF (30 mL, 0.3M) at 0 °C was treated with BH_3-DMS (1.8 mL, 18 mmol, 2.0 equiv). The reaction was worked up with 1.0 M aqueous NaOH and 30% w/w aqueous H_2O_2 (1.8 mL). The crude material obtained was purified by flash column chromatography (eluent: 15% EtOAc in hexane) to afford **21a** as colorless oil (1.00 g, 6.49 mmol, 72% yield).

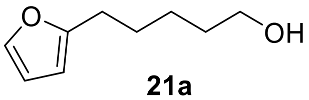

21a

21a: R_f (15% EtOAc / hexane) 0.27; ^{1}H NMR (500 MHz, $CDCl_3$): δ 7.29 (d, *J* = 1.8 Hz, 1H), 6.27 (t, *J* = 2.6 Hz, 1H), 5.97 (d, *J* = 3.1 Hz, 1H), 3.65 (q, *J* = 6.3 Hz, 2H), 2.64 (t, *J* = 7.5 Hz, 2H), 1.72 – 1.60 (m, 2H), 1.59 (d, *J* = 7.2 Hz, 2H), 1.42 (d, *J* = 7.5 Hz, 2H) ppm; $^{13}C\{^1H\}$ NMR (100 MHz, $CDCl_3$): δ 156.4, 140.9, 110.2, 104.8, 63.0, 32.6, 28.0, 28.0, 25.4 ppm. The spectral characteristics are consistent with those of **21a** in the literature.[32]

5-(5-((*tert*-Butyldiphenylsiloxy)methyl)furan-2-yl)pentan-1-ol (21c)

According to the literature procedure,[28] **39c** (0.43 g, 1.06 mmol, 1 equiv) in anhydrous THF (3.6 mL, 0.3M) was treated with BH_3-DMS (0.2 mL, 2.1 mmol, 2 equiv). The reaction was worked up with reaction was worked up with 1.0 M aqueous NaOH and 30% w/w aqueous H_2O_2 (0.2 mL). The crude material obtained was purified by flash column chromatography (eluent: 15% EtOAc in hexane) to give **21c** as a yellow oil (0.293 g, 0.693 mmol, 65% yield).

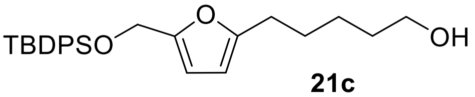

21c

21c: R_f (15% EtOAc / hexane) 0.20; IR (neat) 3350, 2931, 2858, 1428, 1060, 823 cm^{-1}; ^{1}H NMR (500 MHz, $CDCl_3$): δ 7.74 – 7.69 (m, 4H), 7.47 – 7.35 (m, 6H), 6.01 (d, *J* = 2.9 Hz, 1H), 5.88 (d, *J* = 3.0 Hz, 1H), 4.61 (s, 2H), 3.65 (t, *J* = 6.7 Hz, 2H), 2.60 (t, *J* = 7.5 Hz, 2H), 1.70 – 1.63 (m, 2H), 1.65 – 1.58 (m, 2H), 1.47 – 1.37 (m, 2H), 1.06 (s, 9H) ppm; $^{13}C\{^1H\}$ NMR (100 MHz, $CDCl_3$): δ 155.9, 152.2, 135.8, 133.7, 129.7, 127.7, 108.2, 105.4, 63.0, 59.1, 32.6, 28.1, 27.9, 26.9, 25.4, 19.4 ppm; HRMS (EI, 40eV) *m/z* [M-^{t}Bu]$^+$ calcd for $C_{22}H_{25}O_3Si$ 365.1567, found 365.1569.

5-(3-Methylfuran-2-yl)pentan-1-ol (21d)

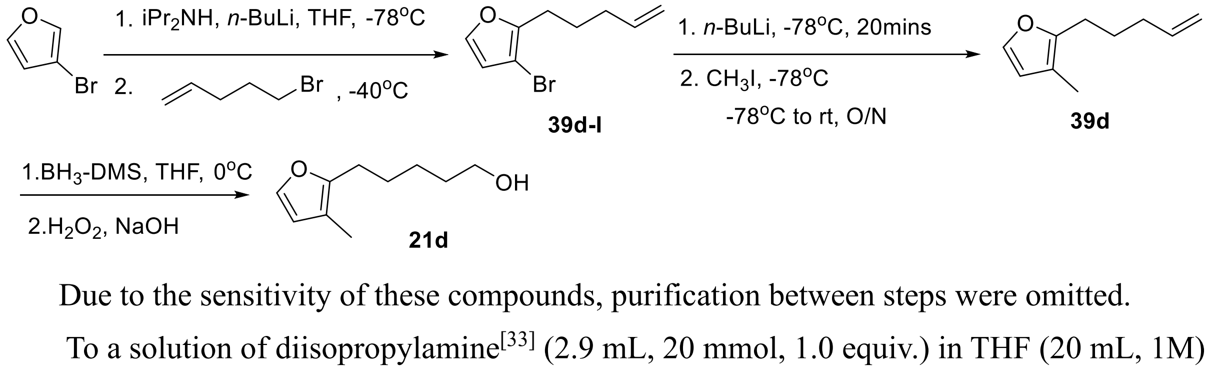

Due to the sensitivity of these compounds, purification between steps were omitted.

To a solution of diisopropylamine[33] (2.9 mL, 20 mmol, 1.0 equiv.) in THF (20 mL, 1M) at −78 °C was added *n*-BuLi (1.89 M in hexanes, 11 mL, 20 mmol, 1 equiv). After stirring at −78 °C for 25 min, neat 3-bromofuran (1.8 mL, 20.4 mmol, 1 equiv) was added dropwise. The reaction mixture was allowed to slowly warm to –40 °C, then 5-bromo-1-pentene (2.5 mL, 20.8 mmol, 1.02 equiv.) was added dropwise to the reaction mixture at -40 °C. The resultant solution

was allowed to warm to room temperature overnight with stirring. The reaction was quenched with saturated aqueous NH_4Cl, and the organic layer was separated. The aqueous layer was back-extracted with Et_2O twice. The combined organics were washed with brine and dried over anhydrous $MgSO_4$. The volatiles were removed *in vacuo* to give a 1:1.6 mixture of unreacted 5-bromo-1-pentene and 3-bromo-2-(pent-4-en-1-yl)furan (**39d-I**). The residual unreacted 5-bromo-1-pentene was removed from the desired product **39d-I** by placing the mixture under high vacuum (23 °C, <1 mmHg for 10 minutes) to give 3-bromo-2-(pent-4-en-1-yl)furan (**39d-I**) as a pale yellow oil, which was used in the next reaction without further purification.

The crude 3-bromo-2-(pent-4-en-1-yl)furan (**39d-I**) (1 equiv) was dissolved in THF (40 mL) and cooled to -78 °C. *n*-BuLi (1.89 M in hexanes, 4.3 mL, 8.1 mmol, 1.0 equiv) was added dropwise and with stirring and the reaction was maintained at -78 °C for 25 mins. MeI (2.0 mL, 32 mmol, 4.0 equiv) was added dropwise, and the mixture was allowed to slowly warm to room temperature. The reaction mixture was quenched with saturated aqueous NH_4Cl, and the organic layer was separated. The aqueous layer was back-extracted with Et_2O twice. The combined organics were washed with brine and dried over anhydrous $MgSO_4$. The volatiles were removed *in vacuo* to give a 1:1.1 mixture of 3-methyl-2-(pent-4-en-1-yl)furan **(39d)** and 2-(pent-4-en-1-yl)furan (**39a**) as a pale yellow oil, which was used in the next step without further purification.

According to the literature procedure,[28] the crude mixture of **39d** and **39a** in anhydrous THF (23 mL, 0.35M) was treated with BH_3-DMS (1.6 mL, 15.8 mmol, 2.0 equiv). The reaction was worked up with 1.0 M aqueous NaOH and 30% w/w aqueous H_2O_2 (1.6 mL). The crude material obtained was separated and purified by flash column chromatography (eluent: 10% EtOAc in hexane) to give **21d** as a pale-yellow oil (0.224 g, 1.33 mmol, 17% yield over three steps).

21d: R_f (15% EtOAc / hexane) 0.24; IR (neat) 3316, 2930, 2860, 1511, 1445, 1048, 890 cm^{-1}; ^1H NMR (400 MHz, $CDCl_3$): δ 7.21 (s, 1H), 6.15 (s, 1H), 3.69 – 3.59 (m, 2H), 2.57 (t, J = 7.4 Hz, 2H), 1.95 (s, 3H), 1.68 – 1.52 (m, 3H), 1.45 – 1.28 (m, 1H), 1.26 (s, 1H), 1.23 – 1.16 (m, 1H) ppm; $^{13}C\{^1H\}$ NMR (125 MHz, $CDCl_3$): δ 151.3, 139.8, 112.8, 109.4, 63.1, 32.7, 28.4, 25.9, 25.4, 9.2 ppm; HRMS (EI, 40eV) *m/z* $[M]^+$ calcd for $C_{10}H_{16}O_2$ 168.1145, found 168.1149.

4-(Furan-2-yl)butan-1-ol (21e)

According to the literature procedure,[28] furan **39e** (0.97 g, 7.9 mmol, 1 equiv) in anhydrous THF (27 mL, 0.3M) was treated with BH_3-DMS (1.6 mL, 15.9 mmol, 2 equiv). The reaction was worked up with 1.0 M aqueous NaOH and 30% w/w aqueous H_2O_2 (1.6 mL). The crude material obtained was purified by flash column chromatography (eluent: 15% EtOAc in hexane) to give **21e** as a yellow oil (0.675 g, 4.82 mmol, 61% yield).

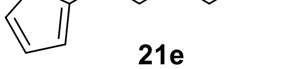

21e

21e: R_f (20% EtOAc / hexane) 0.23; ^1H NMR (400 MHz, $CDCl_3$): δ 7.30 (d, J = 1.9 Hz, 1H), 6.28 (d, J = 3.3, 1.8 Hz, 1H), 5.99 (d, J = 3.2 Hz, 1H), 3.71 – 3.62 (m, 2H), 2.66 (t, J = 7.4 Hz, 2H), 1.84 – 1.66 (m, 2H), 1.68 – 1.56 (m, 2H), 1.27 (t, J = 5.1 Hz, 1H) ppm; $^{13}C\{^1H\}$ NMR (100 MHz, $CDCl_3$): δ 156.1, 140.9, 110.2, 105.0, 62.8, 32.3, 27.8, 24.4 ppm. The spectral characteristics are consistent with those of **21e** in the literature.[34]

4-(5-Methylfuran-2-yl)butan-1-ol (21f)

According to the literature procedure,[28] furan **39f** (1.86 g, 13.7 mmol, 1.0 equiv) in anhydrous THF (46 mL, 0.3 M) was treated with BH_3-DMS (2.8 mL, 27.4 mmol, 2.0 equiv). The reaction was worked up with 1.0 M aqueous NaOH and 30% w/w aqueous H_2O_2 (2.8 mL). The crude material obtained was purified by flash column chromatography (eluent: 15% EtOAc in hexane) to give **21f** as a yellow oil (1.78 g, 11.5 mmol, 84% yield).

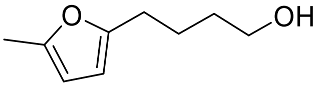

21f

21f: R_f (15% EtOAc / hexane) 0.27; ^{1}H NMR (400 MHz, $CDCl_3$): δ 5.88 – 5.82 (m, 2H), 3.71 – 3.62 (m, 2H), 2.60 (t, J = 7.2 Hz, 2H), 2.25 (s, 3H), 1.77 – 1.64 (m, 2H), 1.68 – 1.56 (m, 3H), 1.25 (t, 1H) ppm; ^{13}C{^{1}H} NMR (100 MHz, $CDCl_3$): δ 154.3, 150.4, 105.9, 105.6, 62.9, 32.4, 27.9, 24.5, 13.6 ppm. The spectral characteristics are consistent with those of **21f** in the literature.[35]

***tert*-Butyl 2-(5-hydroxypentyl)-1*H*-pyrrole-1-carboxylate (21g)**

According to the literature procedure,[28] pyrrole **39g** (0.78 g, 3.4 mmol, 1.0 equiv) in anhydrous THF (11 mL, 0.3 M) was treated with BH_3-DMS (0.7 mL, 6.7 mmol, 2.0 equiv). The reaction was worked up with 1.0 M aqueous NaOH and 30% w/w aqueous H_2O_2 (0.7 mL). The crude material obtained was purified by flash column chromatography (eluent: 15% EtOAc in hexane) to give **21g** as a colorless oil (0.50 g, 2.0 mmol 59% yield).

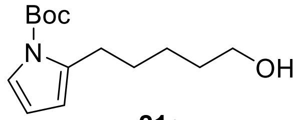

21g

21g: R_f (15% EtOAc / hexane) 0.36; IR (neat) 3354, 2933, 1737, 1323, 1123, 1058, 716 cm^{-1}; ^{1}H NMR (500 MHz, $CDCl_3$): δ 7.18 (dd, J = 3.4, 1.8 Hz, 1H), 6.07 (t, J = 3.3 Hz, 1H), 5.97 – 5.92 (m, 1H), 3.65 (t, J = 6.6 Hz, 2H), 2.88 – 2.82 (m, 2H), 1.70 – 1.59 (m, 4H), 1.59 (s, 9H), 1.55 (s, 2H) ppm; ^{13}C{^{1}H} NMR (100 MHz, $CDCl_3$): δ 150.0, 136.3, 121.0, 111.0, 110.0, 83.4, 63.1, 32.8, 28.9, 28.8, 28.2, 25.7 ppm; HRMS (EI, 40eV) m/z [M]$^+$ calcd for $C_{14}H_{23}NO_3$ 253.1672, found 253.1677.

5-(1-Tosyl-1*H*-pyrrol-2-yl)pentan-1-ol (21h)

According to the literature procedure,[28] pyrrole **39h** (0.158 g, 0.54 mmol, 1 equiv) in anhydrous THF (1.8 mL, 0.3M) was treated with BH_3-DMS (0.1 mL, 1.1 mmol, 2 equiv). The reaction was worked up with 1.0 M aqueous NaOH and 30% w/w aqueous H_2O_2 (0.1 mL). The crude material obtained was purified by flash column chromatography (eluent: 15% EtOAc in hexane) to give **21h** as a colorless oil (86.7 mg, 0.282 mmol, 52% yield).

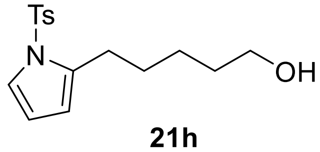

21h

21h: R_f (15% EtOAc / hexane) 0.16; IR (neat) 3270, 2933, 2858, 1597, 1361, 811 cm^{-1}; ^{1}H NMR (500 MHz, $CDCl_3$): δ 7.63 (d, 2H), 7.34 – 7.24 (m, 3H), 6.19 (t, J = 3.3 Hz, 1H), 6.01 – 5.96 (m, 1H), 3.62 (t, J = 6.5 Hz, 2H), 2.66 (t, J = 7.7 Hz, 2H), 2.40 (s, 3H), 1.60 – 1.51 (m, 4H), 1.45 – 1.31 (m, 2H) ppm; ^{13}C{^{1}H} NMR (100 MHz, $CDCl_3$): δ 144.9, 136.7, 135.8, 130.1, 126.8, 122.4, 112.0, 111.4, 63.0, 32.6, 31.1, 28.7, 27.2, 25.5, 21.7 ppm; HRMS (EI, 40eV) m/z [M]$^+$ calcd for $C_{16}H_{21}NO_3S$ 307.1237, found 307.1247.

5-(Thiophen-2-yl)pentan-1-ol (21i)

According to the literature procedure,[28] thiophene **39i** (0.77 g, 5.1 mmol, 1.0 equiv) in anhydrous THF (28 mL, 0.3M) was treated with BH_3-DMS (1.0 mL, 10 mmol, 2.0 equiv). The

reaction was worked up with 1.0 M aqueous NaOH and 30% w/w aqueous H_2O_2 (1 mL). The crude material obtained was purified by flash column chromatography (eluent: 15% EtOAc in hexane) to give **21i** as a yellow oil (0.436 g, 2.56 mmol, 51% yield).

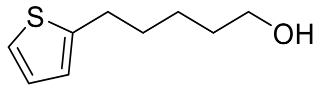

21i

21i: R_f (15% EtOAc / hexane) 0.23; ^{1}H NMR (500 MHz, $CDCl_3$): δ 7.11 (dd, J = 5.1, 1.2 Hz, 1H), 6.91 (dd, J = 5.1, 3.4 Hz, 1H), 6.80 – 6.76 (m, 1H), 3.65 (t, J = 6.6 Hz, 2H), 2.84 (t, J = 8.1 Hz, 2H), 1.77 – 1.67 (m, 2H), 1.66 – 1.56 (m, 2H), 1.49 – 1.37 (m, 2H) ppm; ^{13}C{^{1}H} NMR (100 MHz, $CDCl_3$): δ 145.6, 126.8, 124.2, 123.0, 63.0, 32.6, 31.7, 30.0, 25.9 ppm. The spectral characteristics are consistent with those of **21i** in the literature.[32]

5-(3-Methylthiophen-2-yl)pentan-1-ol (21j)

According to the literature procedure,[28] thiophene **39j** (2.49 g, 15 mmol, 1.0 equiv) in anhydrous THF (50 mL, 0.3 M) was treated with BH_3-DMS (3.0 mL, 30 mmol, 2.0 equiv). The reaction was worked up with 1.0 M aqueous NaOH and 30% w/w aqueous H_2O_2 (3 mL). The crude material obtained was purified by flash column chromatography (eluent: 15% EtOAc in hexane) to give **21j** as a yellow oil (1.46 g, 7.92 mmol, 53% yield).

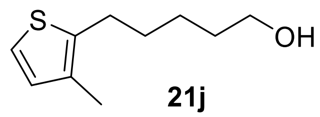

21j

21j: R_f (15% EtOAc / hexane) 0.24; IR (neat) 3318, 2929, 2857, 1449, 1048, 1061, 834 cm^{-1}; ^{1}H NMR (400 MHz, $CDCl_3$): δ 7.01 (d, J = 5.0 Hz, 1H), 6.78 (d, J = 5.1 Hz, 1H), 3.66 (t, 2H), 2.73 (t, J = 7.6 Hz, 2H), 2.15 (s, 3H), 1.72 – 1.55 (m, 4H), 1.50 – 1.38 (m, 2H), 1.27 (t, J = 3.9 Hz, 1H) ppm; ^{13}C{^{1}H} NMR (100 MHz, $CDCl_3$): δ 138.6, 132.6, 130.0, 121.0, 63.0, 32.7, 31.4, 27.9, 25.5, 13.7 ppm; ESI-HRMS *m/z* calcd for $C_{10}H_{17}OS$ $[M+H]^+$: 185.0995, found 185.0993.

5-(3,4-Dimethylthiophen-2-yl)pentan-1-ol (21k)

According to the literature procedure,[28] thiophene **39k** (1.89 g, 10.5 mmol, 1 equiv) in anhydrous THF (35 mL, 0.3M) was treated with BH_3-DMS (2.1 mL, 21 mmol, 2.0 equiv). The reaction was worked up with 1.0 M aqueous NaOH and 30% w/w aqueous H_2O_2 (2.1 mL). The crude material obtained was purified by flash column chromatography (eluent: 15% EtOAc in hexane) to give **21k** as a pale-yellow oil (1.32 g, 6.66 mmol, 63% yield).

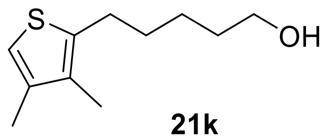

21k

21k: R_f (15% EtOAc / hexane) 0.28; IR (neat) 3330, 2931, 2857, 1441, 1048, 908, 858 cm^{-1}; ^{1}H NMR (400 MHz, $CDCl_3$): δ 6.69 (s, 1H), 3.65 (t, J = 6.5 Hz, 2H), 2.72 (t, 2H), 2.13 (s, 3H), 2.03 (s, 3H), 1.69 – 1.55 (m, 4H), 1.50 – 1.38 (m, 2H), 1.27 (s, 1H) ppm; ^{13}C{^{1}HF NMR (100 MHz, $CDCl_3$): δ 138.6, 137.9, 132.4, 116.6, 63.0, 32.7, 31.3, 28.5, 25.5, 15.4, 12.3 ppm; ESI-HRMS *m/z* calcd for $C_{11}H_{19}OS$ $[M+H]^+$: 199.1151, found 199.1140.

7.2 Preparation of aldehydes 22a-k

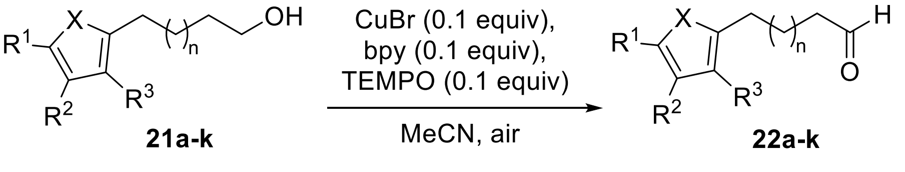

5-(Furan-2-yl)pentanal (22a)

To a solution of **21a** (1.03 g, 6.70 mmol, 1.0 equiv) in MeCN (34 mL, 0.2 M) was added CuBr (96 mg, 0.67 mmol, 0.1 equiv), bpy (0.105 g, 0.670 mmol, 0.10 equiv), and TEMPO (0.105 g, 0.670 mmol, 0.10 equiv). The dark red/brown reaction mixture was stirred rapidly open to air. The reaction was monitored by TLC until no starting material remained which was often accompanied by a change in colour to green/blue. When the reaction was complete, water and pentane were added to the reaction mixture, and the organic phase was separated. The aqueous phase was back-extracted thrice with EtOAc. The combined organics were washed with brine and dried over anhydrous $MgSO_4$. The volatiles were removed *in vacuo*. The residue was purified by flash column chromatography (eluent: 10% EtOAc in hexane) to afford **22a** as a blood orange oil (0.79g, 5.19 mmol, 77% yield).

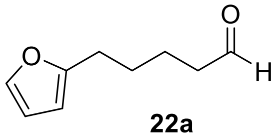

22a

22a: R_f(15% EtOAc / hexane) 0.56; ^{1}H NMR (500 MHz, $CDCl_3$): δ 9.77 (d, *J* = 1.8 Hz, 1H), 7.30 (s, 1H), 6.28 (d, *J* = 2.9 Hz, 1H), 5.99 (d, *J* = 2.9 Hz, 1H), 2.68 – 2.63 (m, 2H), 2.48 – 2.44 (m, 2H), 1.71 – 1.67 (m, 4H) ppm; ^{13}C{^{1}H} NMR (100 MHz, $CDCl_3$): δ 202.5, 155.6, 140.9, 110.1, 104.6, 43.6, 27.7, 27.6, 21.6 ppm. The spectral characteristics are consistent with those of **22a** in the literature.[36]

5-(5-Methylfuran-2-yl)pentanal (22b)

According to the procedure for the synthesis of **22a**, compound **22b** was obtained from the treatment of alcohol **21b** (1.04 g, 6.18 mmol, 1.0 equiv) in MeCN (31 mL, 0.2 M) with CuBr (89 mg, 0.62 mmol, 0.1 equiv), bpy (97 mg, 0.62 mmol, 0.1 equiv), and TEMPO (97 mg, 0.62 mmol, 0.1 equiv). The crude material obtained was purified by flash column chromatography (eluent: 10% EtOAc in hexane) to give aldehyde **22b** as a blood orange oil (0.91 g, 5.5 mmol, 89% yield).

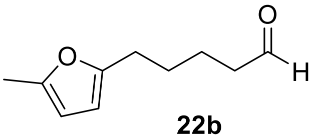

22b

22b: R_f(15% EtOAc / hexane) 0.56; ^{1}H NMR (400 MHz, $CDCl_3$): δ 9.76 (t, *J* = 1.7 Hz, 1H), 5.88 – 5.81 (m, 2H), 2.59 (t, *J* = 6.8 Hz, 2H), 2.51 – 2.40 (m, 2H), 2.24 (s, 3H), 1.77 – 1.58 (m, 4H) ppm; ^{13}C{^{1}H} NMR (100 MHz, $CDCl_3$): δ 202.7, 153.9, 149.6, 105.9, 105.7, 43.7, 27.9, 27.8, 21.7, 13.6 ppm. The spectral characteristics are consistent with those of **22b** in the literature.[37]

5-(5-*Tert*-butyldiphenylsilyloxymethylfuran-2-yl)pentanal (22c)

According to the procedure for the synthesis of **22a**, compound **22c** was obtained from the treatment of alcohol **21c** (0.293 g, 0.693 mmol, 1.00 equiv) in MeCN (3.5 mL, 0.2 M) with CuBr (9.9 mg, 0.069 mmol, 0.1 equiv), bpy (11 mg, 0.069 mmol, 0.1 equiv), and TEMPO (11 mg, 0.069 mmol, 0.1 equiv). The crude material obtained was purified by flash column chromatography (eluent: 10% EtOAc in hexane) to give aldehyde **22c** as a blood orange oil (0.282 g, 0.670 mmol, 97% yield).

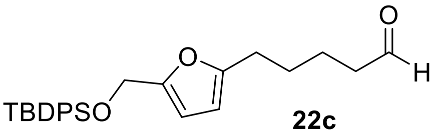

22c

22c: R_f(15% EtOAc / hexane) 0.37; IR (neat) 2930, 2857, 1723, 1427, 1110, 1061, 701 cm^{-1}; ^{1}H NMR (500 MHz, $CDCl_3$): δ 9.76 (s, 1H), 7.73 – 7.69 (m, 4H), 7.45 – 7.36 (m, 6H), 6.02 (s, 1H), 5.89 (s, 1H), 4.61 (s, 2H), 2.62 (d, *J* = 7.0 Hz, 2H), 2.46 (d, *J* = 5.8 Hz, 2H), 1.68 (s, 4H), 1.06 (d, *J* = 2.5 Hz, 9H) ppm; ^{13}C{^{1}H} NMR (100 MHz, $CDCl_3$): δ 202.5, 155.2, 152.8, 135.7, 133.6, 129.7, 127.7, 108.1, 105.6, 59.0, 43.6, 27.8, 27.5, 26.8, 21.6, 19.3 ppm; HRMS (EI,

40eV) *m/z* [M-^{t}Bu]$^+$ calcd for $C_{22}H_{23}O_3Si$ 363.1411, found 363.1403.

5-(3-Methylfuran-2-yl)pentanal (22d)

According to the procedure for the synthesis of **22a**, compound **22d** was obtained from the treatment of alcohol **21d** (0.230 g, 1.39 mmol, 1.00 equiv) in MeCN (7 mL, 0.2 M) with CuBr (20 mg, 0.14 mmol, 0.1 equiv), bpy (22 mg, 0.14 mmol, 0.1 equiv), and TEMPO (22 mg, 0.14 mmol, 0.1 equiv). The crude material obtained was purified by flash column chromatography (eluent: 10% EtOAc in hexane) to give aldehyde **22d** as a blood orange oil (0.175 g, 1.05 mmol, 76% yield).

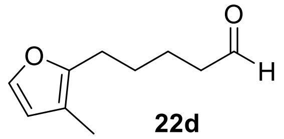

22d

22d: R_f (10% EtOAc / hexane) 0.60; IR (neat) 2934, 1723, 1511, 1149, 890, 731 cm^{-1}; ^{1}H NMR (500 MHz, $CDCl_3$): δ 9.76 (t, *J* = 1.8 Hz, 1H), 7.21 (d, *J* = 1.8 Hz, 1H), 6.16 (d, *J* = 1.8 Hz, 1H), 2.62 – 2.56 (m, 2H), 2.44 (ddt, *J* = 7.1, 5.0, 1.8 Hz, 2H), 1.96 (s, 3H), 1.70 – 1.56 (m, 4H) ppm; $^{13}C\{^1H\}$ NMR (100 MHz, $CDCl_3$): δ 202.6, 150.5, 139.9, 114.0, 112.8, 43.7, 28.0, 25.6, 21.6, 9.9 ppm; HRMS (EI, 40eV) *m/z* [M]$^+$ calcd for $C_{10}H_{14}O_2$ 166.0988, found 166.0989.

4-(Furan-2-yl)butanal (22e)

According to the procedure for the synthesis of **22a**, compound **22e** was obtained from the treatment of alcohol **21e** (0.42 g, 3.0 mmol, 1.0 equiv) in MeCN (15 mL, 0.2 M) with CuBr (43 mg, 0.30 mmol, 0.1 equiv), bpy (47 mg, 0.30 mmol, 0.1 equiv), and TEMPO (47 mg, 0.30 mmol, 0.1 equiv). The crude material obtained was purified by flash column chromatography (eluent: 10% EtOAc in hexane) to give aldehyde **22e** as a blood orange oil (0.298 g, 2.16 mmol, 72% yield).

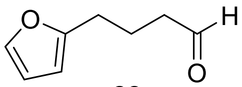

22e

22e: R_f (20% EtOAc / hexane) 0.60; ^{1}H NMR (400 MHz, $CDCl_3$): δ 9.76 (t, *J* = 1.5 Hz, 1H), 7.32 (d, *J* = 1.8 Hz, 1H), 6.29 (dd, *J* = 3.2, 1.8 Hz, 1H), 6.02 (d, 1H), 2.70 (t, *J* = 7.3 Hz, 2H), 2.50 (td, *J* = 7.3, 1.5 Hz, 2H), 2.00 (q, *J* = 7.3 Hz, 2H) ppm; $^{13}C\{^1H\}$ NMR (100 MHz, $CDCl_3$): δ 202.0, 154.8, 141.1, 110.1, 105.5, 43.0, 27.1, 20.6 ppm. The spectral characteristics are consistent with those of **22e** in the literature.[26]

4-(5-Methylfuran-2-yl)butanal (22f)

According to the procedure for the synthesis of **22a**, compound **22f** was obtained from the treatment of alcohol **21f** (1.78 g, 11.5 mmol, 1.0 equiv) in MeCN (58 mL, 0.2 M) with CuBr (0.166 g, 1.15 mmol, 0.1 equiv), bpy (0.180 g, 1.15 mmol, 0.1 equiv), and TEMPO (0.180 g, 1.15 mmol, 0.1 equiv). The crude material obtained was purified by flash column chromatography (eluent: 10% EtOAc in hexane) to give aldehyde **22f** as a blood orange oil (0.98 g, 6.4 mmol, 56% yield).

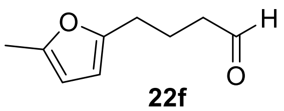

22f

22f: R_f (15% EtOAc / hexane) 0.58; ^{1}H NMR (500 MHz, $CDCl_3$): δ 9.76 (t, *J* = 1.5 Hz, 1H), 5.88 (d, *J* = 3.0 Hz, 1H), 5.86 – 5.83 (m, 1H), 2.64 (t, *J* = 7.3 Hz, 2H), 2.49 (td, *J* = 7.3, 1.6 Hz, 2H), 2.26 (s, 3H), 1.97 (m, *J* = 7.3 Hz, 2H) ppm; $^{13}C\{^1H\}$ NMR (100 MHz, $CDCl_3$): δ 202.1, 152.9, 150.5, 106.0, 105.7, 43.0, 27.1, 20.6, 13.4 ppm. The spectral characteristics are consistent with those of **22f** in the literature.[38]

tert-Butyl 2-(5-oxopentyl)-1*H*-pyrrole-1-carboxylate (22g)

According to the procedure for the synthesis of **22a**, compound **22g** was obtained from the treatment of alcohol **21g** (0.32 g, 1.3 mmol, 1.0 equiv) in MeCN (6.3 mL, 0.2 M) with CuBr (18 mg, 0.13 mmol, 0.1 equiv), bpy (20 mg, 0.13 mmol, 0.1 equiv), and TEMPO (20 mg, 0.13 mmol, 0.1 equiv). The crude material obtained was purified by flash column chromatography (eluent: 10% EtOAc in hexane) to give aldehyde **22g** as a blood orange oil (0.295 g, 1.17 mmol, 93% yield).

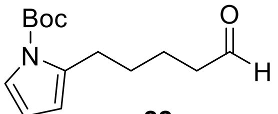

22g

22g: R_f(10% EtOAc / hexane) 0.41; IR (neat) 2936, 1730, 1322, 1123, 720 cm^{-1}; ^1H NMR (500 MHz, $CDCl_3$): δ 9.77 (t, *J* = 1.8 Hz, 1H), 7.18 (dd, *J* = 3.4, 1.8 Hz, 1H), 6.07 (t, *J* = 3.3 Hz, 1H), 5.97 – 5.93 (m, 1H), 2.87 (t, 2H), 2.47 (td, *J* = 7.1, 1.8 Hz, 2H), 1.78 – 1.61 (m, 4H), 1.59 (s, 9H) ppm; $^{13}C\{^1H\}$ NMR (100 MHz, $CDCl_3$): δ 202.7, 149.8, 135.7, 121.0, 111.1, 110.0, 83.4, 43.8, 28.6, 28.5, 28.1, 21.9 ppm; HRMS (EI, 40eV) *m/z* $[M]^+$ calcd for $C_{14}H_{21}NO_3$ 251.1516, found 251.1523.

5-(1-Tosyl-1*H*-pyrrol-2-yl)pentanal (22h)

According to the procedure for the synthesis of **22a**, compound **22h** was obtained from the treatment of alcohol **21h** (0.219 g, 0.710 mmol, 1.0 equiv) in MeCN (3.6 mL, 0.2 M) with CuBr (10 mg, 0.071 mmol, 0.1 equiv), bpy (11 mg, 0.071 mmol, 0.1 equiv), and TEMPO (11 mg, 0.071 mmol, 0.1 equiv). The crude material obtained was purified by flash column chromatography (eluent: 10% EtOAc in hexane) to give aldehyde **22h** as a blood orange oil (0.166 g, 0.544 mmol, 77% yield).

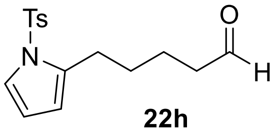

22h

22h: R_f(30% EtOAc / hexane) 0.51; IR (neat) 2943, 1719, 1354, 1154, cm^{-1}; ^1H NMR (400 MHz, $CDCl_3$): δ 9.76 (t, *J* = 1.7 Hz, 1H), 7.68 – 7.61 (m, 2H), 7.36 – 7.27 (m, 3H), 6.22 (t, *J* = 3.3 Hz, 1H), 6.01 (dd, *J* = 3.2, 1.6 Hz, 1H), 2.70 (t, *J* = 7.1 Hz, 2H), 2.48 – 2.41 (m, 5H), 1.77 – 1.53 (m, 4H) ppm; $^{13}C\{^1H\}$ NMR (100 MHz, $CDCl_3$): δ 202.5, 144.9, 136.6, 135.2, 130.1, 126.8, 122.5, 112.2, 111.5, 43.7, 28.4, 27.1, 21.7, 21.7 ppm; HRMS (EI, 40eV) *m/z* $[M]^+$ calcd for $C_{16}H_{19}NO_3S$ 305.1086, found 305.1082.

5-(Thiophen-2-yl)pentanal (22i)

According to the procedure for the synthesis of **22a**, compound **22i** was obtained from the treatment of alcohol **21i** (0.432 g, 2.54 mmol, 1.0 equiv) in MeCN (13 mL, 0.2 M) with CuBr (36 mg, 0.25 mmol, 0.1 equiv), bpy (40 mg, 0.25 mmol, 0.1 equiv), and TEMPO (40 mg, 0.25 mmol, 0.1 equiv). The crude material obtained was purified by flash column chromatography (eluent: 10% EtOAc in hexane) to give aldehyde **22i** as a blood orange oil (0.420 g, 2.50 mmol, 98% yield).

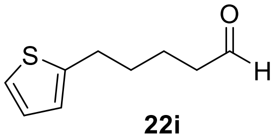

22i

22i: R_f(15% EtOAc / hexane) 0.58; ^1H NMR (500 MHz, $CDCl_3$): δ 9.77 (t, *J* = 1.7 Hz, 1H), 7.12 (dd, *J* = 5.1, 1.2 Hz, 1H), 6.92 (dd, *J* = 5.1, 3.4 Hz, 1H), 6.79 (dd, *J* = 3.4, 1.2 Hz, 1H), 2.86 (dd, *J* = 7.9, 5.7 Hz, 2H), 2.47 (td, *J* = 6.9, 2.1 Hz, 2H), 1.78 – 1.66 (m, 4H) ppm; $^{13}C\{^1H\}$ NMR (100 MHz, $CDCl_3$): δ 202.1, 144.5, 126.5, 124.0, 122.8, 43.3, 30.9, 29.4, 21.2 ppm. The spectral characteristics are consistent with those of **22i** in the literature.[38]

5-(3-Methylthiophen-2-yl)pentanal (22j)

According to the procedure for the synthesis of **22a**, compound **22j** was obtained from the treatment of alcohol **21j** (1.01 g, 5.48 mmol, 1.0 equiv) in MeCN (27 mL, 0.2 M) with CuBr (79 mg, 0.55 mmol, 0.1 equiv), bpy (86 mg, 0.55 mmol, 0.1 equiv), and TEMPO (86 mg, 0.55 mmol, 0.1 equiv). The crude material obtained was purified by flash column chromatography (eluent: 10% EtOAc in hexane) to give aldehyde **22j** as a blood orange oil (0.82 g, 4.5 mmol, 82% yield).

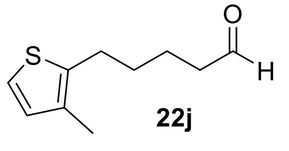

22j

22j: R_f (10% EtOAc / hexane) 0.61; IR (neat) 2930, 1721, 1455, 1070, 1061, 702 cm^{-1}; ^{1}H NMR (500 MHz, $CDCl_3$): δ 9.77 (t, J = 1.7 Hz, 1H), 7.02 (d, J = 5.0 Hz, 1H), 6.78 (d, J = 5.1 Hz, 1H), 2.75 (t, J = 7.1 Hz, 2H), 2.47 (td, J = 7.0, 1.7 Hz, 2H), 2.16 (s, 3H), 1.78 – 1.58 (m, 4H) ppm; $^{13}C\{^{1}H\}$ NMR (100 MHz, $CDCl_3$): δ 202.4, 137.8, 132.7, 130.0, 121.1, 43.7, 30.9, 27.6, 21.6, 13.6 ppm; ESI-HRMS *m/z* calcd for $C_{10}H_{15}OS$ $[M+H]^+$: 183.0838, found 185.0837.

5-(3,4-Dimethylthiophen-2-yl)pentanal (22k)

According to the procedure for the synthesis of **22a**, compound **22k** was obtained from the treatment of alcohol **21k** (0.75 g, 3.8 mmol, 1.0 equiv) in MeCN (19 mL, 0.2 M) with CuBr (54 mg, 0.38 mmol, 0.1 equiv), bpy (59 mg, 0.38 mmol, 0.1 equiv), and TEMPO (59 mg, 0.38 mmol, 0.1 equiv). The crude material obtained was purified by flash column chromatography (eluent: 10% EtOAc in hexane) to give aldehyde **22k** as a blood orange oil (0.639 g, 3.26 mmol, 86% yield).

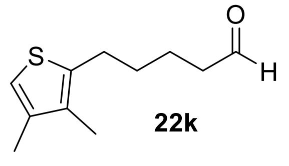

22k

22k: R_f (15% EtOAc / hexane) 0.63; IR (neat) 2927, 1722, 1444, 909, 730 cm^{-1}; ^{1}H NMR (500 MHz, $CDCl_3$): δ 9.78 (t, J = 1.7 Hz, 1H), 6.70 (s, 1H), 2.75 (t, J = 7.2 Hz, 2H), 2.47 (td, J = 7.1, 1.7 Hz, 2H), 2.14 (s, 3H), 2.04 (s, 3H), 1.77 – 1.62 (m, 4H) ppm; $^{13}C\{^{1}H\}$ NMR (100 MHz, $CDCl_3$): δ 202.4, 137.8, 137.8, 132.5, 116.6, 43.6, 30.8, 28.1, 21.6, 15.3, 12.1 ppm; ESI-HRMS *m/z* calcd for $C_{11}H_{16}OS$ $[M+H]^+$: 197.0995, found 197.0987.

7.3 Preparation of chloroaldehydes **23a-k**

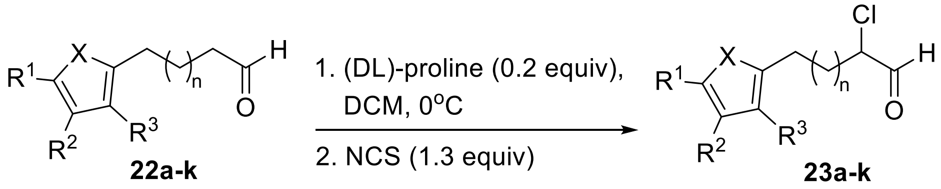

2-Chloro-5-(furan-2-yl)pentanal (23a)

To a solution of aldehyde **22a** (0.12 g, 0.77 mmol, 1 equiv) in anhydrous DCM (1.5 mL, 0.5 M) was added (DL)-proline (18 mg, 0.15 mmol, 0.2 equiv) and NCS (0.13 g, 0.99 mmol, 1.3 equiv) at 0 °C. The reaction was stirred at room temperature and monitored by TLC until no starting material remained. The resulting mixture was cooled to -78 °C and pentane was added. The mixture was filtered through a fritted funnel, and the volatiles were removed *in vacuo*. The residue was purified by flash column chromatography (eluent: 10% EtOAc in hexane) to give **23a** as a pale-yellow oil (96 mg, 0.51 mmol, 66% yield).

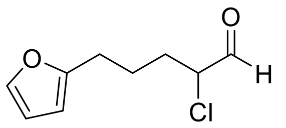

23a

23a: R_f (5% EtOAc in hexane) 0.18; IR (neat) 2926, 1723, 1569, 1453, 1386, 1217, 1019, 957, 778 cm^{-1}; ^1H NMR (400 MHz, $CDCl_3$): δ 9.49 (d, J = 2.2 Hz, 1H), 7.31 (dd, J = 1.9, 0.9 Hz, 1H), 6.28 (dd, J = 3.2, 1.9 Hz, 1H), 6.06 – 5.98 (m, 1H), 4.18 (ddd, J = 7.6, 5.1, 2.2 Hz, 1H), 2.69 (t, J = 6.9 Hz, 2H), 2.11 – 1.98 (m, 1H), 1.99 – 1.71 (m, 3H) ppm; $^{13}C\{^1H\}$ NMR (100 MHz, $CDCl_3$): δ 195.2, 154.9, 141.3, 110.3, 105.5, 63.8, 31.4, 27.3, 24.3 ppm; ESI-HRMS *m/z* calcd for $C_9H_{12}ClO_2$ $[M+H]^+$: 187.0520, found 187.0519.

2-Chloro-5-(5-methylfuran-2-yl)pentanal (23b)

According to the procedure for the synthesis of **23a**, compound **23b** was obtained from the treatment of aldehyde **22b** (0.1 g, 0.6 mmol, 1 equiv) in anhydrous DCM (1.2 mL, 0.5 M) with (DL)-proline (14 mg, 0.12 mmol, 0.2 equiv) and NCS (0.10 g, 0.78 mmol, 1.3 equiv) at 0 °C. The crude material obtained was purified by flash column chromatography (eluent: 10% EtOAc in hexane) to afford **23b** as an orange oil (65.8 mg, 0.330 mmol, 55% yield).

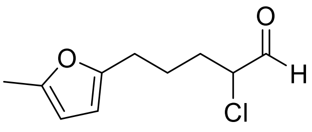

23b

23b: R_f (10% EtOAc in hexane) 0.28; IR (neat) 2921, 2850, 1743, 1617, 1569, 1455, 1434, 1379, 1218, 1135, 1020, 994, 961, 851, 780, 667, 609 cm^{-1}; ^1H NMR (500 MHz, $CDCl_3$): δ 9.49 (d, J = 2.2 Hz, 1H), 5.87 (d, J = 3.1 Hz, 1H), 5.84 (d, J = 3.0 Hz, 1H), 4.17 (ddd, J = 7.8, 5.3, 2.4 Hz, 1H), 2.63 (t, J = 7.2 Hz, 2H), 2.25 (s, 3H), 2.09 – 1.98 (m, 1H), 1.93 – 1.83 (m, 2H), 1.83 – 1.71 (m, 1H) ppm; $^{13}C\{^1H\}$ NMR (100 MHz, $CDCl_3$): δ 195.2, 153.0, 150.7, 106.1, 106.0, 63.8, 31.4, 27.4, 24.3, 13.6 ppm; ESI-HRMS *m/z* calcd for $C_{10}H_{14}ClO_2$ $[M+H]^+$: 201.0676, found 201.0678.

5-(5-*tert*-Butyldiphenylsiloxymethylfuran-2-yl)-2-chloropentanal (23c)

According to the procedure for the synthesis of **23a**, compound **23c** was obtained from the treatment of aldehyde **22c** (0.158 g, 0.376 mmol, 1 equiv) in anhydrous DCM (0.8 mL, 0.5 M) with (DL)-proline (9 mg, 0.08 mmol, 0.2 equiv) and NCS (65 mg, 0.49 mmol, 1.3 equiv) at 0 °C. The crude material obtained was purified by flash column chromatography (eluent: 10% EtOAc in hexane) to afford **23c** as a colorless oil (97 mg, 0.213 mmol, 57% yield).

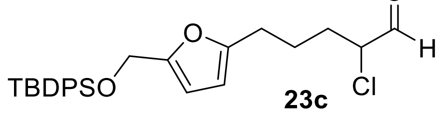

23c: R_f (15% EtOAc in hexane) 0.25; IR (neat) 2935, 2862, 1732, 1428, 703 cm^{-1}; ^1H NMR (500 MHz, $CDCl_3$): δ 9.48 (d, J = 2.1 Hz, 1H), 7.69 (d, J = 7.2 Hz, 4H), 7.46 – 7.35 (m, 6H), 6.01 (d, J = 3.0 Hz, 1H), 5.90 (d, J = 3.0 Hz, 1H), 4.59 (s, 2H), 4.16 (dt, J = 5.7, 3.0 Hz, 1H), 2.63 (t, J = 7.3 Hz, 2H), 2.05 – 1.98 (m, 1H), 1.92 – 1.70 (m, 4H), 1.05 (s, 9H) ppm; $^{13}C\{^1H\}$ NMR (100 MHz, $CDCl_3$): δ 195.2, 154.5, 152.6, 135.8, 133.6, 129.8, 127.8, 108.2, 106.1, 63.8, 59.0, 31.4, 27.4, 26.9, 24.2, 19.4 ppm; HRMS (EI, 40eV) *m/z* $[M-^tBu]^+$ calcd for $C_{22}H_{22}ClO_3Si$ 397.1021, found 397.1019.

2-Chloro-5-(3-methylfuran-2-yl)pentanal (23d)

According to the procedure for the synthesis of **23a**, compound **23d** was obtained from the treatment of aldehyde **22d** (0.1 g, 0.6 mmol, 1 equiv) in anhydrous DCM (1.2 mL, 0.5 M) with (DL)-proline (14 mg, 0.12 mmol, 0.2 equiv) and NCS (0.10 g, 0.78 mmol, 1.3 equiv) at 0 °C. The crude material obtained was purified by flash column chromatography

(eluent: 10% EtOAc in hexane) to afford **23d** as a yellow oil (30.8 mg, 0.153 mmol, 25% yield).

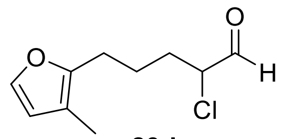

23d

23d: R_f (15% EtOAc in hexane) 0.30; IR (neat) 2944, 1741, 1536, 908, 727 cm^{-1}; ^1H NMR (500 MHz, $CDCl_3$): δ 9.48 (d, *J* = 2.3 Hz, 1H), 7.22 (d, *J* = 1.8 Hz, 1H), 6.16 (d, *J* = 1.8 Hz, 1H), 4.15 (ddd, *J* = 7.9, 5.1, 2.3 Hz, 1H), 2.62 (t, *J* = 6.9 Hz, 2H), 2.06 – 1.95 (m, 1H), 1.95 (s, 3H), 1.95 – 1.69 (m, 3H) ppm; $^{13}C\{^1H\}$ NMR (100 MHz, $CDCl_3$): δ 195.2, 149.5, 140.2, 114.5, 112.9, 63.8, 31.4, 25.1, 24.6, 9.9 ppm; HRMS (EI, 40eV) *m/z* $[M]^+$ calcd for $C_{10}H_{13}ClO_2$ 200.0599, found 200.0596.

2-Chloro-4-(furan-2-yl)butanal (23e)

According to the procedure for the synthesis of **23a**, compound **23e** was obtained from the treatment of aldehyde **22e** (0.100 g, 0.724 mmol, 1 equiv) in anhydrous DCM (1.5 mL, 0.5 M) with (DL)-proline (16 mg, 0.072 mmol, 0.2 equiv) and NCS (0.126 g, 0.941 mmol, 1.3 equiv) at 0 °C. The crude material obtained was purified by flash column chromatography (eluent: 10% EtOAc in hexane) to afford **23e** as a blood orange oil (0.116 g, 0.670 mmol, 93% yield).

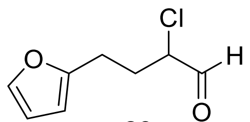

23e

23e: R_f (15% EtOAc in hexane) 0.34; IR (neat) 2926, 2853, 1731, 1597, 1507, 1146, 924 cm^{-1}; ^1H NMR (500 MHz, $CDCl_3$) δ 9.49 (d, *J* = 1.8 Hz, 1H), 7.33 (d, *J* = 1.8 Hz, 1H), 6.29 (dd, *J* = 3.2, 1.9 Hz, 1H), 6.08 (d, *J* = 3.1 Hz, 1H), 4.19 (ddd, *J* = 8.9, 4.8, 1.8 Hz, 1H), 3.06 – 2.79 (m, 2H), 2.44 – 2.33 (m, 1H), 2.21 – 2.05 (m, 1H) ppm; $^{13}C\{^1H\}$ NMR (100 MHz, $CDCl_3$): δ 194.9, 153.3, 141.7, 110.3, 106.4, 63.1, 30.7, 24.0 ppm; HRMS (EI, 40eV) *m/z* $[M]^+$ calcd for $C_8H_9ClO_2$ 172.0286, found 172.0288.

2-Chloro-4-(5-methylfuran-2-yl)butanal (23f)

According to the procedure for the synthesis of **23a**, compound **23f** was obtained from the treatment of aldehyde **22f** (0.10 g, 0.66 mmol, 1 equiv) in anhydrous DCM (1.3 mL, 0.5 M) with (DL)-proline (15 mg, 0.13 mmol, 0.2 equiv) and NCS (0.12 g, 0.85 mmol, 1.3 equiv) at 0 °C. The crude material obtained was purified by flash column chromatography (eluent: 10% EtOAc in hexane) to afford **23f** as a blood orange oil (72 mg, 0.39 mmol, 59% yield).

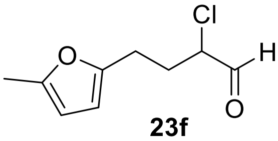

23f

23f: R_f (15% EtOAc in hexane) 0.58; IR (neat) 2923, 2854, 1731, 1570, 1341, 1217, 999 cm^{-1}; ^1H NMR (400 MHz, $CDCl_3$): δ 9.49 (d, *J* = 1.9 Hz, 1H), 5.93 (d, *J* = 3.0 Hz, 1H), 5.85 (dd, *J* = 3.0, 1.2 Hz, 1H), 4.20 (ddd, *J* = 8.9, 4.8, 1.9 Hz, 1H), 2.91 – 2.71 (m, 2H), 2.43 – 2.29 (m, 1H), 2.25 (s, 3H), 2.20 – 2.04 (m, 1H) ppm; $^{13}C\{^1H\}$ NMR (100 MHz, $CDCl_3$): δ 195.1, 151.4, 151.2, 107.1, 106.1, 63.2, 30.9, 24.1, 13.6 ppm; ESI-HRMS *m/z* calcd for $C_9H_{12}ClO_2$ $[M+H]^+$: 187.0520, found 187.0520.

***tert*-Butyl 2-(4-chloro-5-oxopentyl)-1*H*-pyrrole-1-carboxylate (23g)**

According to the procedure for the synthesis of **23a**, compound **23g** was obtained from the treatment of aldehyde **22g** (0.15 g, 0.59 mmol, 1 equiv) in anhydrous DCM (1.2 mL, 0.5 M) with (DL)-proline (14 mg, 0.12 mmol, 0.2 equiv) and NCS (0.10 g, 0.78 mmol, 1.3 equiv) at 0 °C. The crude material obtained was purified by flash column chromatography

(eluent: 10% EtOAc in hexane) to afford **23g** as a yellow oil (0.132 g, 0.462 mmol, 78% yield).

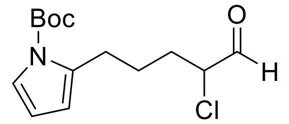
23g

23g: R_f (15% EtOAc in hexane) 0.36; IR (neat) 2963, 1735, 1370, 1322, 1122, 725cm^{-1}; ^{1}H NMR (400 MHz, $CDCl_3$): δ 9.49 (d, *J* = 2.3 Hz, 1H), 7.18 (dd, *J* = 3.4, 1.8 Hz, 1H), 6.07 (t, *J* = 3.3 Hz, 1H), 5.97 (dd, *J* = 3.1, 1.8 Hz, 1H), 4.19 (ddd, *J* = 8.0, 5.3, 2.3 Hz, 1H), 2.90 (t, *J* = 6.8, 6.3 Hz, 2H), 2.13 – 1.97 (m, 1H), 2.00 – 1.66 (m, 3H), 1.59 (s, 9H) ppm; $^{13}C\{^1H\}$ NMR (100 MHz, $CDCl_3$): δ 195.3, 149.5, 134.9, 121.2, 111.5, 110.1, 83.6, 63.9, 31.7, 28.2, 28.1, 25.1 ppm; HRMS (EI, 40eV) *m/z* $[M]^+$ calcd for $C_{14}H_{20}ClNO_3$ 285.1126, found 285.1127.

2-Chloro-5-(1-tosyl-1*H*-pyrrol-2-yl)pentanal (23h)

According to the procedure for the synthesis of **23a**, compound **23h** was obtained from the treatment of aldehyde **22h** (0.172 g, 0.560 mmol, 1.0 equiv) in anhydrous DCM (1.1 mL, 0.5 M) with (DL)-proline (12 mg, 0.11 mmol, 0.2 equiv) and NCS (97 mg, 0.73 mmol, 1.3 equiv) at 0 °C. The crude material obtained was purified by flash column chromatography (eluent: 10% EtOAc in hexane) to afford **23h** as a yellow oil (0.18 g, 0.54 mmol, 96% yield).

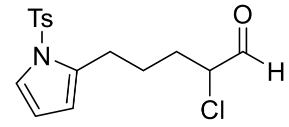
23h

23h: R_f (30% EtOAc in hexane) 0.33; IR (neat) 2943, 1719, 1354, 1154, 665, 591 cm^{-1}; ^{1}H NMR (500 MHz, $CDCl_3$): δ 9.44 (d, *J* = 2.2 Hz, 1H), 7.60 (d, *J* = 8.2 Hz, 3H), 7.29 – 7.22 (m, 3H), 6.18 (d, *J* = 3.4 Hz, 1H), 5.98 (s, 1H), 4.09 (td, *J* = 6.4, 5.2, 2.2 Hz, 1H), 2.69 (t, *J* = 7.6 Hz, 2H), 2.38 (s, 3H), 2.04 – 1.91 (m, 1H), 1.82 – 1.68 (m, 2H), 1.71 – 1.56 (m, 1H) ppm; $^{13}C\{^1H\}$ NMR (100 MHz, $CDCl_3$): δ 195.1, 145.1, 136.5, 134.4, 130.2, 126.8, 122.7, 112.5, 111.5, 63.7, 31.5, 26.5, 25.0, 21.7 ppm; HRMS (EI, 40eV) *m/z* $[M]^+$ calcd for $C_{16}H_{18}ClNO_3S$ 339.0696, found 339.0694.

2-Chloro-5-(thiophen-2-yl)pentanal (23i)

According to the procedure for the synthesis of **23a**, compound **23i** was obtained from the treatment of aldehyde **22i** (0.100 g, 0.594 mmol, 1.0 equiv) in anhydrous DCM (1.2 mL, 0.5 M) with (DL)-proline (14 mg, 0.12 mmol, 0.2 equiv) and NCS (0.10 g, 0.77 mmol, 1.3 equiv) at 0 °C. The crude material obtained was purified by flash column chromatography (eluent: 10% EtOAc in hexane) to afford **23i** as a yellow oil (82.5 mg, 0.407 mmol, 69% yield).

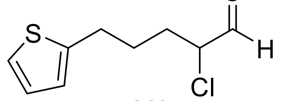
23i

23i: R_f (15% EtOAc in hexane) 0.34; IR (neat) 2929, 1731, 1438, 1034, 848, 695 cm^{-1}; ^{1}H NMR (400 MHz, $CDCl_3$): δ 9.51 (d, *J* = 2.2 Hz, 1H), 7.16 (d, *J* = 5.1 Hz, 1H), 6.95 (dd, *J* = 5.2, 3.4 Hz, 1H), 6.82 (d, *J* = 3.4 Hz, 1H), 4.21 (ddd, *J* = 7.6, 5.0, 2.2 Hz, 1H), 2.92 (t, *J* = 6.9 Hz, 2H), 2.15 – 2.00 (m, 1H), 2.03 – 1.77 (m, 3H) ppm; $^{13}C\{^1H\}$ NMR (100 MHz, $CDCl_3$): δ 195.2, 143.9, 127.0, 124.6, 123.5, 63.7, 31.3, 29.2, 27.7 ppm; ESI-HRMS *m/z* calcd for $C_9H_{12}ClOS$ $[M+H]^+$: 203.0292, found 203.0300.

2-Chloro-5-(3-methylthiophen-2-yl)pentanal (23j)

According to the procedure for the synthesis of **23a**, compound **23j** was obtained from the treatment of aldehyde **22j** (0.15 g, 0.82 mmol, 1 equiv) in anhydrous DCM (1.6 mL, 0.5 M) with (DL)-proline (19 mg, 0.16 mmol, 0.2 equiv) and NCS (0.14 g, 1.1 mmol, 1.3 equiv) at 0

°C. The crude material obtained was purified by flash column chromatography (eluent: 10% EtOAc in hexane) to afford **23j** as a yellow oil (0.14 g, 0.65 mmol, 79% yield).

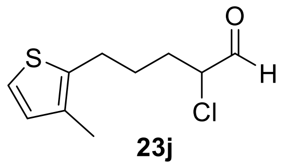

23j

23j: R_f (15% EtOAc in hexane) 0.34; IR (neat) 2925, 1731, 1454, 1231, 1033, 703 cm^{-1}; ^{1}H NMR (400 MHz, $CDCl_3$): δ 9.49 (d, *J* = 2.3 Hz, 1H), 7.03 (d, *J* = 5.1 Hz, 1H), 6.78 (d, *J* = 5.0 Hz, 1H), 4.18 (td, *J* = 5.4, 2.7 Hz, 1H), 2.78 (t, *J* = 7.3 Hz, 3H), 2.16 (s, 3H), 2.14 – 1.97 (m, 1H), 1.97 – 1.70 (m, 3H) ppm; ^{13}C{^{1}H} NMR (100 MHz, $CDCl_3$): δ 195.2, 137.0, 133.2, 130.2, 121.5, 63.8, 31.5, 27.5, 27.2, 13.3 ppm; ESI-HRMS *m/z* calcd for $C_{10}H_{14}ClOS$ [M+H]$^+$: 217.0448, found 217.0444.

2-Chloro-5-(3,4-dimethylthiophen-2-yl)pentanal (23k)

According to the procedure for the synthesis of **23a**, compound **23k** was obtained from the treatment of aldehyde **22k** (0.15 g, 0.76 mmol, 1 equiv) in anhydrous DCM (1.5 mL, 0.5 M) with (DL)-proline (18 mg, 0.15 mmol, 0.2 equiv) and NCS (0.13 g, 0.99 mmol, 1.3 equiv) at 0 °C. The crude material obtained was purified by flash column chromatography (eluent: 10% EtOAc in hexane) to afford **23k** as a yellow oil (0.112 g, 0.485 mmol, 64% yield).

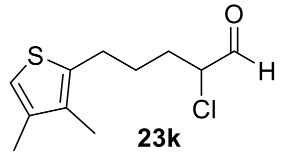

23k

23k: R_f (15% EtOAc in hexane) 0.36; IR (neat) 2923, 1731, 1442, 1026, 727 cm^{-1}; ^{1}H NMR (500 MHz, $CDCl_3$): δ 9.49 (d, *J* = 2.3 Hz, 1H), 6.71 (s, 1H), 4.17 (ddd, *J* = 8.0, 5.3, 2.3 Hz, 1H), 2.77 (t, *J* = 7.0 Hz, 2H), 2.13 (s, 3H), 2.09 – 2.00 (m, 4H), 1.94 – 1.81 (m, 2H), 1.83 – 1.70 (m, 1H) ppm; ^{13}C{^{1}H} NMR (125 MHz, $CDCl_3$): δ 195.2, 138.0, 137.0, 133.0, 116.5, 63.8, 31.5, 27.8, 27.4, 15.4, 12.3 ppm; ESI-HRMS *m/z* calcd for $C_{11}H_{16}ClOS$ [M+H]$^+$: 231.0605, found 231.0599.

7.4 Preparation of epoxy allylic silanes **25a-k**

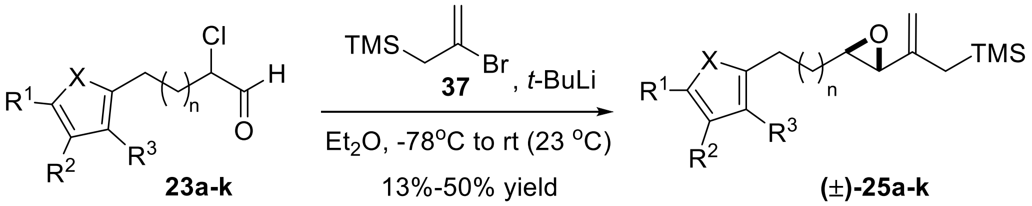

2-(*2S*,3S)-3-3-(Furan-2-yl-propyl)oxiran-2-yl-allyltrimethylsilane (25a)**

To a solution of **37** (0.128 g, 0.664 mmol, 1.3 equiv) in anhydrous Et_2O (0.8 mL, 0.8 M) at –78 °C was added *tert*-butyllithium (1.0 M in pentanes, 1.4 mL, 1.4 mmol, 2.7 equiv) dropwise. The reaction mixture was stirred at –78 °C for 25 mins, then at room temperature for 25 mins. The mixture was then cooled back to –78 °C, and a solution of α-chloroaldehyde **23a** (95.4 mg, 0.511 mmol, 1 equiv) in anhydrous THF (2.6 mL, 0.2 M) at 0 °C was added by cannula to the reaction mixture. The resulting mixture was allowed to stir and warm to room temperature, and the reaction progress was monitored by TLC until no starting material remained. The reaction mixture was quenched with saturated aqueous NH_4Cl, and the organic layer was separated. The aqueous layer was extracted with Et_2O three times. The combined organic layers were washed with brine, dried over anhydrous $MgSO_4$, and concentrated *in vacuo*. The residue was purified by flash column chromatography (eluent: 0.3% Et_2O / hexane) to afford **25a** as a pale-yellow oil (68 mg, 0.26 mmol, 51% yield).

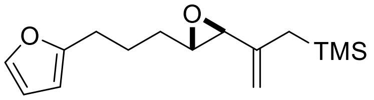

25a

25a: R_f (5% EtOAc in hexane) 0.56; IR (neat) 2925, 1602, 1507, 1248, 1147, 1006, 884, 848, 727, 694, 626, 599 cm^{-1}; ^1H NMR (500 MHz, $CDCl_3$): δ 7.30 (s, 1H), 6.28 (dd, J = 3.1, 1.9 Hz, 1H), 5.99 (d, J = 3.1 Hz, 1H), 4.95 (s, 1H), 4.71 (s, 1H), 3.01 (d, J = 2.2 Hz, 1H), 2.77 (ddd, J = 6.7, 4.7, 2.1 Hz, 1H), 2.69 (t, J = 7.5 Hz, 2H), 1.88 – 1.74 (m, 2H), 1.71 – 1.63 (m, 1H), 1.62 – 1.56 (m, 1H), 1.49 – 1.40 (m, 2H), 0.03 (s, 9H) ppm; $^{13}C\{^1H\}$ NMR (125 MHz, $CDCl_3$): δ 155.8, 143.5, 141.0, 110.2, 109.4, 105.2, 60.6, 59.5, 31.6, 27.8, 24.7, 21.8, -1.3 ppm; ESI-HRMS *m/z* calcd for $C_{15}H_{25}O_2Si$ $[M+H]^+$: 265.1618, found 265.1620.

Trimethyl-2-(*2S*,3S)-3-3-(5-methylfuran-2-yl)propyl-oxiran-2-yl-allylsilane (25b)**

According to the procedure for the synthesis of **25a**, epoxy allylsilane **25b** was obtained from the treatment of α-chloroaldehyde **23b** (65.9 mg, 0.328 mmol, 1 equiv) in anhydrous THF (0.7 mL, 0.5 M) with **37** (0.080 g, 0.43 mmol, 1.3 equiv) in Et_2O (1 mL, 0.4 M) and *tert*-butyllithium (1.2 M in pentanes, 0.75 mL, 0.89 mmol, 2.7 equiv). The crude material obtained was purified by flash column chromatography (eluent: 0.3% Et_2O / hexane) to give **25b** as a pale-yellow oil (26 mg, 0.093 mmol, 28% yield).

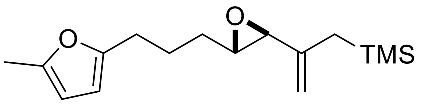

25b

25b: R_f (5% EtOAc in hexane) 0.59; IR (neat) 2952, 1626, 1570, 1455, 1418, 1247, 1219, 1157, 841 cm^{-1}; ^1H NMR (500 MHz, $CDCl_3$): δ 5.86 (d, J = 3.0 Hz, 1H), 5.83 (d, J = 3.1 Hz, 1H), 4.95 (s, 1H), 4.71 (s, 1H), 3.01 (d, J = 2.1 Hz, 1H), 2.77 (ddd, J = 6.7, 4.8, 2.1 Hz, 1H), 2.63 (t, J = 7.5 Hz, 2H), 2.25 (s, 3H), 1.87 – 1.53 (m, 4H), 1.49 – 1.39 (m, 2H), 0.03 (s, 9H) ppm; $^{13}C\{^1H\}$ NMR (100 MHz, $CDCl_3$): δ 153.9, 150.5, 143.5, 109.4, 105.9, 105.7, 60.6, 59.5, 31.6, 27.9, 24.8, 21.8, 13.7, -1.3 ppm; ESI-HRMS *m/z* calcd for $C_{16}H_{26}O_2Si$ $[M]^+$: 279.1775, found 279.1776.

***tert*-Butyldiphenyl-5-(3-(*2S*,3S**)-3-3-trimethylsilyl-prop-1-en-2-yl-oxiran-2-yl-propyl-furan-2-yl-methoxysilane (25c)**

According to the procedure for the synthesis of **25a**, epoxy allylsilane **25c** was obtained from the treatment of α-chloroaldehyde **23c** (62.2 mg, 0.137 mmol, 1 equiv) in anhydrous THF (0.7 mL, 0.2 M) with **37** (0.034 g, 0.18 mmol, 1.3 equiv) in Et_2O (0.5 mL, 0.4 M) and *tert*-butyllithium (1.0 M in pentanes, 0.37 mL, 0.37 mmol, 2.7 equiv). The crude material obtained was purified by flash column chromatography (eluent: 0.3% Et_2O / hexane) to give **25c** as a pale-yellow oil (23 mg, 0.043 mmol, 31% yield).

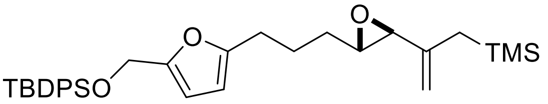

25c

25c: R_f (15% EtOAc in hexane) 0.76; IR(neat) 2955, 2930, 1732, 1678, 1428, 1248, 909, 845 cm^{-1}; ^1H NMR (600 MHz, $CDCl_3$): δ 7.73 – 7.65 (m, 4H), 7.46 – 7.35 (m, 6H), 6.00 (d, J = 3.0 Hz, 1H), 5.89 (d, J = 3.1 Hz, 1H), 4.96 (s, 1H), 4.72 (s, 1H), 4.59 (s, 2H), 3.01 (d, J = 2.1 Hz, 1H), 2.77 (ddd, J = 6.7, 4.7, 2.1 Hz, 1H), 2.64 (t, J = 7.5 Hz, 2H), 1.87 – 1.70 (m, 2H), 1.70 – 1.55 (m, 2H), 1.49 – 1.40 (m, 2H), 1.05 (s, 9H), 0.03 (s, 9H) ppm; $^{13}C\{^1H\}$ NMR (150 MHz, $CDCl_3$): δ 155.3, 152.5, 143.5, 135.8, 133.7, 129.8, 127.8, 109.4, 108.2, 105.7, 60.6, 59.5, 59.1, 31.6, 27.9, 26.9, 24.7, 21.7, 19.4, -1.2 ppm; ESI-HRMS *m/z* calcd for $C_{32}H_{44}NaO_3Si_2$ $[M+Na]^+$: 555.2721, found 555.2722.

Trimethyl-2-(*2S*,3S)-3-3-(3-methylfuran-2-yl-propyl-oxiran-2-yl)allylsilane (25d)**

According to the procedure for the synthesis of **25a**, epoxy allylsilane **25d** was obtained from the treatment of α-chloroaldehyde **23d** (27.4 mg, 0.137 mmol, 1 equiv) in anhydrous THF (0.7 mL, 0.2 M) with **37** (0.034 g, 0.18 mmol, 1.3 equiv) in Et_2O (0.5 mL, 0.4M) and *tert*-butyllithium (1.0 M in pentanes, 0.37 mL, 0.37 mmol, 2.7 equiv). The crude material obtained was purified by flash column chromatography (eluent: 0.3% Et_2O / hexane) to give **25d** as a colorless oil (18 mg, 0.065 mmol, 47% yield).

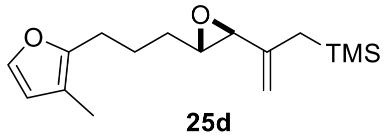

25d

25d: R_f(5% EtOAc in hexane) 0.20; IR (neat) 2926, 1629, 1248, 843, 727 cm^{-1}; ^{1}H NMR (500 MHz, $CDCl_3$): δ 7.21 (d, *J* = 2.9 Hz, 1H), 6.15 (d, *J* = 2.6 Hz, 1H), 4.95 (s, 1H), 4.71 (s, 1H), 3.00 (d, *J* = 2.2 Hz, 1H), 2.79 – 2.72 (m, 1H), 2.62 (t, *J* = 7.2 Hz, 2H), 1.95 (s, 3H), 1.83 – 1.56 (m, 3H), 1.52 – 1.39 (m, 3H), 0.03 (s, 9H) ppm; ^{13}C{^{1}H} NMR (100 MHz, $CDCl_3$): δ 150.7, 143.5, 140.0, 121.1, 112.8, 109.4, 60.6, 59.5, 32.1, 29.8, 29.5, 22.8, 14.3, -1.3 ppm; HRMS (EI, 40eV) *m/z* [M]$^+$ calcd for $C_{16}H_{26}O_2Si$ 278.1697, found 264.1691.

2-(2*R,3*R**)-3-(2-Furan-2-ylethyloxiran-2-yl-allyl)trimethylsilane (25e)**

According to the procedure for the synthesis of **25a**, epoxy allylsilane **25e** was obtained from the treatment of α-chloroaldehyde **23d** (0.116 g, 0.672 mmol, 1 equiv) in anhydrous THF (3.4 mL, 0.2 M) with **37** (0.17 g, 0.87 mmol, 1.3 equiv) in Et_2O (1.5 mL, 0.6 M) and *tert*-butyllithium (1.2 M in pentanes, 1.6 mL, 1.8 mmol, 2.7 equiv). The crude material obtained was purified by flash column chromatography (eluent: 0.3% Et_2O / hexane) to give **25e** as a pale-yellow oil (43 mg, 0.17 mmol, 25% yield).

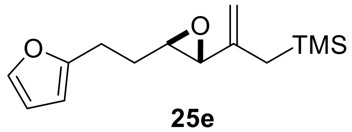

25e

25e: R_f(15% EtOAc in hexane) 0.74; IR (neat) 2940, 1615, 1256, 1010, 846 cm^{-1}; ^{1}H NMR (500 MHz, $CDCl_3$): δ 7.31 (d, *J* = 1.8 Hz, 1H), 6.28 (dd, *J* = 3.1, 1.9 Hz, 1H), 6.03 (d, *J* = 3.2 Hz, 1H), 4.93 (s, 1H), 4.70 (s, 1H), 3.01 (d, *J* = 2.1 Hz, 1H), 2.87 – 2.72 (m, 3H), 2.03 – 1.93 (m, 1H), 1.92 – 1.82 (m, 1H), 1.42 (s, 2H), 0.02 (s, 9H) ppm; ^{13}C{^{1}H} NMR (125 MHz, $CDCl_3$): δ 155.1, 143.6, 141.2, 110.3, 109.4, 105.4, 60.7, 59.0, 30.8, 24.6, 21.8, 0.1 ppm; HRMS (EI, 40eV) *m/z* [M]$^+$ calcd for $C_{14}H_{22}O_2Si$ 250.1384, found 250.1387.

Trimethyl-2-(*2R*,3R)-3-(2-(5-methylfuran-2-yl)ethyl)oxiran-2-yl-allylsilane (25f)**

According to the procedure for the synthesis of **25a**, epoxy allylsilane **25f** was obtained from the treatment of α-chloroaldehyde **23f** (72 mg, 0.39 mmol, 1 equiv) in anhydrous THF (1.9 mL, 0.2 M) with **37** (97 mg, 0.50 mmol, 1.3 equiv) in Et_2O (0.5 mL, 1 M) and *tert*-butyllithium (1.0 M in pentanes, 1.1 mL, 1.1 mmol, 2.7 equiv). The crude material obtained was purified by flash column chromatography (eluent: 0.3% Et_2O / hexane) to give **25f** as a colorless oil (27 mg, 0.10 mmol, 26% yield).

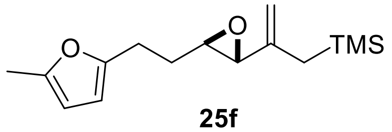

25f

25f: R_f(15% EtOAc in hexane) 0.74; IR (neat) 2922, 1568, 1248, 848, 783 cm^{-1}; ^{1}H NMR (400 MHz, $CDCl_3$): δ 5.89 (d, *J* = 3.0 Hz, 1H), 5.84 (d, *J* = 3.0 Hz, 1H), 4.93 (s, 1H), 4.70 (s, 1H), 3.01 (d, *J* = 2.1 Hz, 1H), 2.81 (ddt, *J* = 9.0, 6.2, 3.1 Hz, 1H), 2.78 – 2.64 (m, 2H), 2.25 (s, 3H), 2.00 – 1.80 (m, 2H), 1.42 (s, 2H), 0.02 (s, 9H) ppm; ^{13}C{^{1}H} NMR (150 MHz, $CDCl_3$): δ 153.2, 150.7, 143.4, 109.4, 106.0, 106.0, 60.8, 59.1, 30.9, 24.6, 21.8, 13.6, -1.3 ppm; HRMS (EI, 40eV) *m/z* [M]$^+$ calcd

for $C_{15}H_{24}O_2Si$ 264.1540, found 264.1543.

tert-Butyl-2-(3-((*2S*,3S**)-3-(3-(trimethylsilyl)prop-1-en-2-yl)oxiran-2-yl)propyl)-1*H*-pyrrole-1-carboxylate (25g)

According to the procedure for the synthesis of **25a**, epoxy allylsilane **25g** was obtained from the treatment of α-chloroaldehyde **23g** (0.122 g, 0.427 mmol, 1 equiv) in anhydrous THF (2.1 mL, 0.2 M) with **37** (0.11 g, 0.56 mmol, 1.3 equiv) in Et_2O (1.4 mL, 0.4 M) and *tert*-butyllithium (1.0 M in pentanes, 1.2 mL, 1.2 mmol, 2.7 equiv). The crude material obtained was purified by flash column chromatography (eluent: 0.3% Et_2O / hexane) to give **25g** as a pale-yellow oil (29 mg, 0.080 mmol, 19% yield).

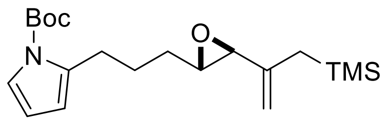

25g

25g: R_f (5% EtOAc in hexane) 0.49; IR (neat) 2958, 1740, 1635, 1322, 1248, 1162, 1127, 845, 719 cm^{-1}; ^1H NMR (500 MHz, $CDCl_3$): δ 7.21 – 7.16 (m, 1H), 6.07 (d, *J* = 3.3 Hz, 1H), 5.96 (s, 1H), 4.95 (s, 1H), 4.71 (s, 1H), 3.01 (s, 1H), 2.90 (t, *J* = 7.7 Hz, 2H), 2.79 (d, *J* = 6.2 Hz, 1H), 1.95 – 1.59 (m, 4H), 1.59 (s, 9H), 1.50 – 1.40 (m, 2H), 0.03 (s, 9H) ppm; $^{13}C\{^1H\}$ NMR (100 MHz, $CDCl_3$): δ 149.6, 143.6, 135.7, 121.1, 111.2, 110.0, 109.3, 83.5, 60.7, 59.7, 31.9, 28.8, 28.2, 25.4, 21.8, -1.3 ppm; HRMS (EI, 40eV) *m/z* $[M-^tBu]^+$ calcd for $C_{16}H_{25}NO_3Si$ 307.1604, found 307.1610.

1-Tosyl-2-(3-((*2S*,3S**)-3-(3-(trimethylsilyl)prop-1-en-2-yl)oxiran-2-yl)propyl)-1*H*-pyrrole (25h)

According to the procedure for the synthesis of **25a**, epoxy allylsilane **25h** was obtained from the treatment of α-chloroaldehyde **23h** (0.19 g, 0.56 mmol. 1 equiv) in anhydrous THF (2.8 mL, 0.2 M) with **37** (0.14 g, 0.73 mmol, 1.3 equiv) in Et_2O (1.8 mL, 0.4 M) and *tert*-butyllithium (1.0 M in pentanes, 1.50 mL, 1.53 mmol, 2.73 equiv). The crude material obtained was purified by flash column chromatography (eluent: 0.3% Et_2O / hexane) to give **25h** as a pale-yellow oil in (29 mg, 0.070 mmol, 13% yield).

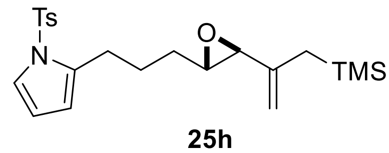

25h

25h: R_f (15% EtOAc in hexane) 0.73; IR (neat) 2950, 1637, 1368, 1174, 848 cm^{-1}; ^1H NMR (500 MHz, $CDCl_3$): δ 7.63 (d, *J* = 8.4 Hz, 2H), 7.30 – 7.25 (m, 3H), 6.20 (t, *J* = 3.3 Hz, 1H), 6.00 (dd, *J* = 3.2, 1.7 Hz, 1H), 4.94 (s, 1H), 4.71 (s, 1H), 2.98 (d, *J* = 2.1 Hz, 1H), 2.72 (t, *J* = 8.1 Hz, 2H), 2.40 (s, 4H), 1.81 – 1.59 (m, 4H), 1.55 – 1.44 (m, 2H), 0.03 (s, 9H) ppm; $^{13}C\{^1H\}$ NMR (100 MHz, $CDCl_3$): δ 144.6, 143.5, 137.0, 135.2, 130.1, 126.9, 122.5, 112.2, 111.5, 109.4, 60.6, 59.5, 31.8, 27.1, 25.4, 21.8, 21.8, -1.3 ppm; HRMS (EI, 40eV) *m/z* $[M-^tBu]^+$ calcd for $C_{22}H_{31}NO_3SSi$ 417.1788, found 417.1777.

Trimethyl-(2-(*2S*,3S**)-3-(3-(thiophen-2-yl)propyl)oxiran-2-yl)allylsilane (25i)

According to the procedure for the synthesis of **25a**, epoxy allylsilane **25i** was obtained from the treatment of α-chloroaldehyde **23i** (72.8 mg, 0.359 mmol, 1 equiv) in anhydrous THF (1.8 mL, 0.2 M) with **37** (90 mg, 0.47 mmol, 1.3 equiv) in Et_2O (0.7 mL, 0.7 M) and *tert*-butyllithium (1.0 M in pentanes, 0.90 mL, 0.98 mmol, 2.7 equiv). The crude material obtained was purified by flash column chromatography (eluent: 0.3% Et_2O / hexane) to give **25i** as a pale-yellow oil in (34 mg, 0.12 mmol, 33% yield).

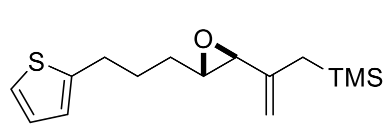

25i

35i: R_f (15% EtOAc in hexane) 0.78; IR (neat) 2949, 1629, 1440, 1247, 1158, 846, 733, 693 cm^{-1}; ^1H NMR (500 MHz, $CDCl_3$): δ 7.12 (d, *J* = 5.2 Hz, 1H), 6.92 (dd, *J* = 5.1, 3.4 Hz, 1H), 6.79 (d, *J* = 3.2 Hz, 1H), 4.96 (s, 1H), 4.72 (s, 1H), 3.01 (d, *J* = 2.1 Hz, 1H), 2.89 (t, *J* = 7.6 Hz, 2H), 2.78 (ddd, *J* = 6.6, 4.6, 2.1 Hz, 1H), 1.93 – 1.77 (m, 2H), 1.75 – 1.66 (m, 1H), 1.65 – 1.56 (m, 1H), 1.50 – 1.40 (m, 2H), 0.03 (s, 9H) ppm; $^{13}C\{^1H\}$ NMR (125 MHz, $CDCl_3$): δ 144.9, 143.5, 126.9, 124.4, 123.2, 109.4, 60.6, 59.5, 31.6, 29.7, 28.3, 21.8, -1.2 ppm; ESI-HRMS *m/z* calcd for $C_{15}H_{25}OSSi$ $[M+H]^+$: 281.1390, found 281.1405.

Trimethyl-2-(2*R,3*R**)-3-3-3-methylthiophen-2-yl-propyl-oxiran-2-yl-allylsilane (25j)**

According to the procedure for the synthesis of **25a**, epoxy allylsilane **25j** was obtained from the treatment of α-chloroaldehyde **23j** (0.133 g, 0.614 mmol, 1 equiv) in anhydrous THF (3.1 mL, 0.2 M) with **37** (0.16 g, 0.80 mmol, 1.3 equiv) in Et_2O (1.3 mL, 0.6 M) and *tert*-butyllithium (1.0 M in pentanes, 1.7 mL, 1.7 mmol, 2.7 equiv). The crude material obtained was purified by flash column chromatography (eluent: 0.3% Et_2O / hexane) to give **25j** as a pale-yellow oil (70 mg, 0.24 mmol, 39% yield).

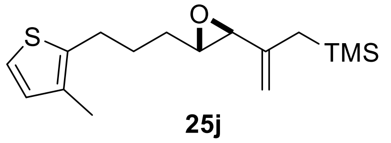

25j

25j: R_f (5% EtOAc in hexane) 0.70; IR (neat) 2944, 1636, 1456, 1247, 1158, 846, 697, 628 cm^{-1}; ^1H NMR (500 MHz, $CDCl_3$): δ 7.02 (d, *J* = 5.1 Hz, 1H), 6.78 (d, *J* = 5.1 Hz, 1H), 4.95 (s, 1H), 4.72 (s, 1H), 3.01 (d, *J* = 2.1 Hz, 1H), 2.82 – 2.75 (m, 3H), 2.15 (s, 3H), 1.90 – 1.65 (m, 3H), 1.64 – 1.56 (m, 1H), 1.49 – 1.39 (m, 2H), 0.03 (s, 9H) ppm; $^{13}C\{^1H\}$ NMR (150 MHz, $CDCl_3$): δ 143.5, 137.9, 132.9, 130.1, 121.2, 109.4, 60.6, 59.5, 31.7, 27.9, 27.7, 21.8, 13.8, -1.2 ppm; ESI-HRMS *m/z* calcd for $C_{16}H_{27}OSSi$ $[M+H]^+$: 295.1546, found 295.1523.

2-(2*S,3*S**)-3-(3-(3,4-Dimethylthiophen-2-yl)propyl)oxiran-2-yl-allyltrimethylsilane (25k)**

According to the procedure for the synthesis of **25a**, epoxy allylsilane **25k** was obtained from the treatment of α-chloroaldehyde **23k** (0.135 g, 0.584 mmol, 1 equiv) in anhydrous THF (1.2 mL, 0.5 M) with **37** (0.15 g, 0.76 mmol, 1.3 equiv) in Et_2O (1.9 mL, 0.4 M) and *tert*-butyllithium (1.0 M in pentanes, 1.6 mL, 1.6 mmol, 2.7 equiv). The crude material obtained was purified by flash column chromatography (eluent: 0.3% Et_2O / hexane) to give **25k** as an orange oil (72 mg, 0.23 mmol, 39% yield).

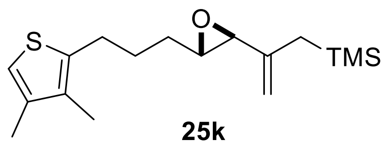

25k

25k: R_f (15% EtOAc in hexane) 0.82; IR (neat) 2939, 1678, 1443, 1247, 1159, 846, 722 cm^{-1}; ^1H NMR (400 MHz, $CDCl_3$): δ 6.69 (s, 1H), 4.95 (s, 1H), 4.71 (s, 1H), 3.01 (s, 1H), 2.81 – 2.69 (m, 3H), 2.13 (s, 3H), 2.06 – 1.99 (m, 4H), 1.87 – 1.56 (m, 3H), 1.48 – 1.40 (m, 2H), 0.03 (s, 9H) ppm; $^{13}C\{^1H\}$ NMR (150 MHz, $CDCl_3$): δ 143.5, 138.0, 138.0, 132.7, 116.8, 109.4, 60.6, 59.6, 31.7, 28.3, 27.8, 21.8, 15.4, 12.3, -1.2 ppm; ESI-HRMS *m/z* calcd for $C_{17}H_{29}OSSi$ $[M+H]^+$: 309.1703, found 309.1716.

8. Experimental Procedures: Preparation of Epoxy Allylsilanes (±)-25m-r

General synthetic route to the synthesis of epoxy allylsilanes **(±)-25m-r**

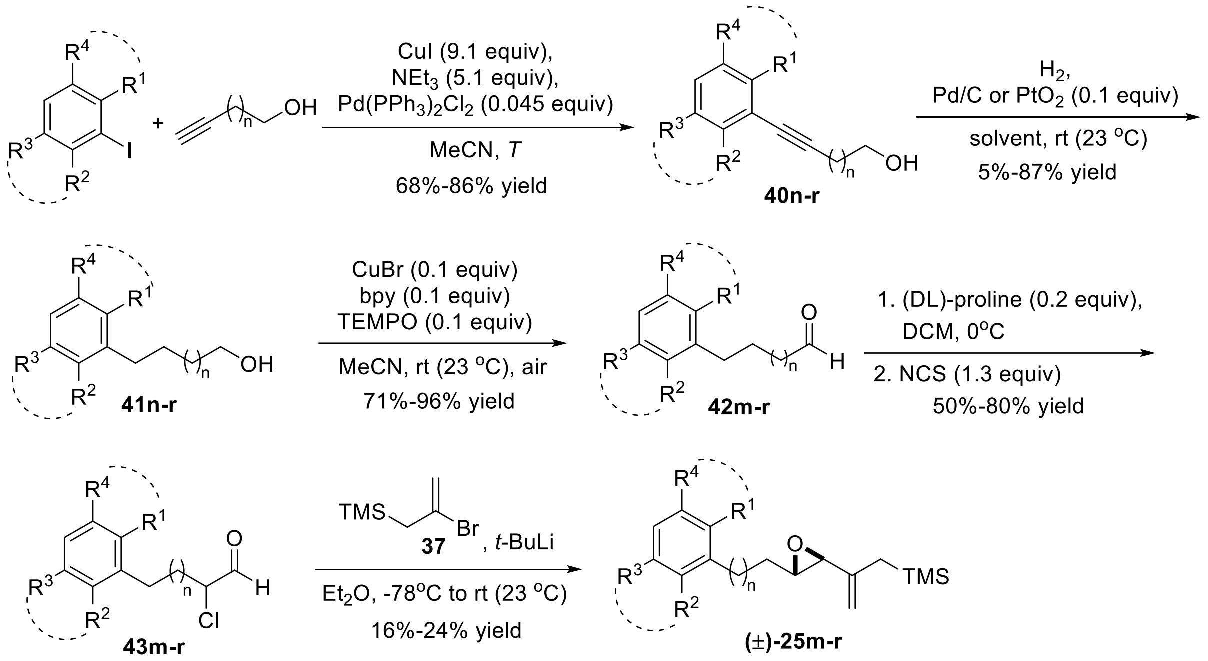

8.1 Preparation of alkynols **40n-r**

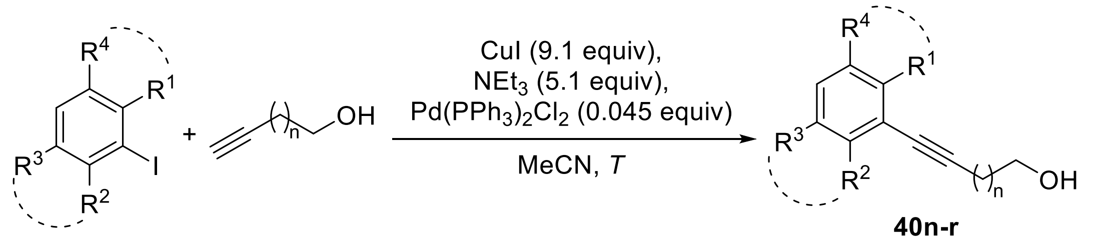

5-(*o*-Tolyl)pent-4-yn-1-ol (40n)

To a solution of 1-iodo-2-methylbenzene (2.19 g, 10.1 mmol, 1.00 equiv) in MeCN (59 mL, 0.17 M) under argon was added NEt_3 (7.1 mL, 51 mmol, 5.1 equiv), $(Ph_3P)_2PdCl_2$ (0.32 g, 0.45 mmol, 0.045 equiv), CuI (0.14 g, 0.91 mmol, 0.091 equiv), followed by pent-4-yn-1-ol (1.1 mL, 12 mmol, 1.2 equiv). The reaction mixture was allowed to stir at room temperature overnight until the reaction was complete as judged by TLC analysis. Then the reaction mixture was removed of volatiles by rotary evaporation, and the residue was subjected to flash column chromatography (eluent: 10% EtOAc / hexane) to afford **40n** as a brown oil (1.51 g, 8.64 mmol, 86% yield).

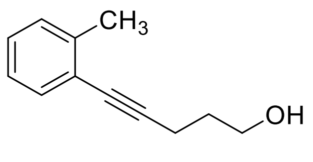

40n

40n: R_f (15% EtOAc / hexane) 0.23; ^{1}H NMR (500 MHz, $CDCl_3$): δ 7.36 (d, J = 7.6 Hz, 1H), 7.20 – 7.14 (m, 2H), 7.13 – 7.07 (m, 1H), 3.85 (q, J = 5.9 Hz, 2H), 2.59 (t, J = 6.9 Hz, 2H), 2.41 (s, 3H), 1.89 (p, J = 6.5 Hz, 2H), 1.49 (t, J = 5.4 Hz, 1H) ppm; $^{13}C\{^1H\}$ NMR (125 MHz, $CDCl_3$): δ 140.0, 132.0, 129.5, 127.8, 125.6, 123.6, 93.4, 80.2, 62.0, 31.7, 20.9, 16.3 ppm. The spectral characteristics are

consistent with those of **40n** in the literature.[39]

4-(2,6-Dimethylphenyl)but-3-yn-1-ol (40o)

To a solution of 2-iodo-1,3-dimethylbenzene (1.09 g, 4.68 mmol, 1.00 equiv) in MeCN (28 mL, 0.17 M) under argon was added NEt_3 (3.3 mL, 24 mmol, 5.1 equiv), $(Ph_3P)_2PdCl_2$ (0.15 g, 0.21 mmol, 0.045 equiv), CuI (82 mg, 0.43 mmol, 0.091 equiv), followed by but-3-yn-1-ol (0.71 mL, 9.4 mmol, 2.0 equiv). The reaction mixture was heated to reflux overnight until the reaction was complete as judged by TLC analysis. Then the reaction mixture was removed of volatiles by rotary evaporation, and the residue was subjected to flash column chromatography (eluent: 15% EtOAc / hexane) to afford **40o** as an orange oil (0.552 g, 3.17 mmol, 68% yield).

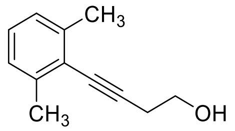

40o

40o: R_f (15% EtOAc / hexane) 0.29; ^1H NMR (500 MHz, $CDCl_3$): 7.09 (dd, J = 8.4, 6.5 Hz, 1H), 7.03 (d, J = 7.5 Hz, 2H), 3.85 (q, J = 6.2 Hz, 2H), 2.79 (t, J = 6.3 Hz, 2H), 2.42 (s, 6H), 1.88 (t, J = 6.3 Hz, 1H) ppm; $^{13}C\{^1H\}$ NMR (125 MHz, $CDCl_3$): δ 140.2, 127.5, 126.7, 123.2, 94.8, 80.3, 61.6, 24.3, 21.3 ppm. The spectral characteristics are consistent with those of **40o** in the literature.[40]

5-(Naphthalen-1-yl)pent-4-yn-1-ol (40p)

To a solution of 1-bromonaphthalene (0.507 g, 2.45 mmol, 1.00 equiv) in *n*-Pr_2NH (19 mL, 0.13 M) under argon was added $(Ph_3P)_2PdCl_2$ (85.8 mg, 0.122 mmol, 0.05 equiv), CuI (45.9 mg, 0.241 mmol, 0.1 equiv), followed by pent-4-yn-1-ol (0.41 mL, 4.4 mmol, 1.8 equiv). The reaction mixture was heated to reflux overnight until the reaction was complete as judged by TLC analysis. Then the reaction mixture was removed of volatiles by rotary evaporation, and the residue was subjected to flash column chromatography (eluent: 20% EtOAc / hexane) to afford **40p** as a brown liquid (0.364 g, 1.73 mmol, 72% yield).

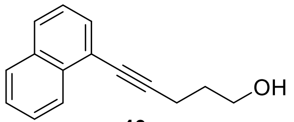

40p

40p: R_f (30% EtOAc / hexane) 0.41; ^1H NMR (500 MHz, $CDCl_3$): δ 8.33 (d, J = 8.3 Hz, 1H), 7.83 (d, J = 8.1 Hz, 1H), 7.79 (d, J = 8.2 Hz, 1H), 7.63 (d, J = 7.2, 1H), 7.59 – 7.47 (m, 2H), 7.40 (dd, J = 8.3, 7.2 Hz, 1H), 3.90 (t, J = 6.2 Hz, 2H), 2.71 (t, J = 6.9 Hz, 2H), 1.97 (p, J = 6.6 Hz, 2H) ppm; $^{13}C\{^1H\}$ NMR (125 MHz, $CDCl_3$): δ 133.6, 133.3, 130.2, 128.4, 128.2, 126.7, 126.4, 126.3, 125.3, 121.5, 94.5, 79.3, 62.0, 31.7, 16.4 ppm. The spectral characteristics are consistent with those of **40p** in the literature.[41]

4-(Naphthalen-1-yl)but-3-yn-1-ol (40q)

To a solution of 1-bromonaphthalene (1.14 g, 5.50 mmol, 1.00 equiv) in *n*-Pr_2NH (42 mL, 0.13 M) under argon was added $(Ph_3P)_2PdCl_2$ (0.193 g, 0.275 mmol, 0.05 equiv), CuI (0.11 g, 0.55 mmol, 0.1 equiv), followed by but-3-yn-1-ol (0.75 mL, 9.9 mmol, 1.8 equiv). The reaction mixture was heated to reflux overnight until the reaction was complete as judged by TLC analysis. Then the reaction mixture was removed of volatiles by rotary evaporation, and the residue was subjected to flash column chromatography (eluent: 20% EtOAc / hexane) to afford **40q** as a brown liquid (0.799 g, 4.08 mmol, 74% yield).

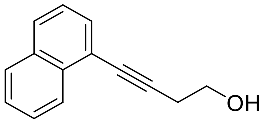

40q

40q: R_f (30% EtOAc / hexane) 0.43; ^{1}H NMR (400 MHz, $CDCl_3$): δ 8.32 (d, *J* = 8.1 Hz, 1H), 7.84 (d, *J* = 8.1 Hz, 1H), 7.81 (d, *J* = 8.1 Hz, 1H), 7.65 (d, *J* = 7.1 Hz, 1H), 7.60 – 7.48 (m, 2H), 7.41 (dd, *J* = 8.3, 7.2 Hz, 1H), 3.92 (t, *J* = 6.3 Hz, 2H), 2.86 (t, *J* = 6.3 Hz, 2H) ppm; ^{13}C{^{1}H} NMR (100 MHz, $CDCl_3$): δ 133.5, 133.3, 130.5, 128.5, 128.4, 126.8, 126.5, 126.2, 125.3, 121.1, 91.5, 80.7, 61.5, 24.3 ppm. The spectral characteristics are consistent with those of **40q** in the literature.[40]

4-(Anthracen-9-yl)but-3-yn-1-ol (40r)

To a solution of 9-bromoanthracene (1.009 g, 3.92 mmol, 1.00 equiv) in *n*-Pr_2NH (30 mL, 0.13 M) under argon was added $(Ph_3P)_2PdCl_2$ (52 mg, 0.074 mmol, 0.019 equiv), CuI (11 mg, 0.055 mmol, 0.014 equiv), followed by but-3-yn-1-ol (0.60 mL, 8.0 mmol, 2.1 equiv). The reaction mixture was heated to reflux overnight until the reaction was complete as judged by TLC analysis. Then the reaction mixture was removed of volatiles by rotary evaporation, and the residue was subjected to flash column chromatography (eluent: 30% EtOAc / hexane) to afford **40r** as an orange solid (0.772 g, 3.13 mmol, 81% yield).

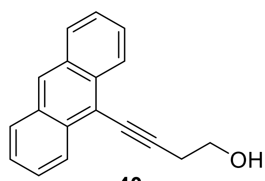

40r

40r: R_f (30% EtOAc / hexane) 0.44; ^{1}H NMR (400 MHz, $CDCl_3$): δ 8.53 (d, *J* = 8.7 Hz, 2H), 8.41 (s, 1H), 8.00 (d, *J* = 8.4 Hz, 2H), 7.62 – 7.52 (m, 2H), 7.52 – 7.42 (m, 2H), 4.04 (q, *J* = 5.7 Hz, 2H), 3.05 (t, *J* = 6.3 Hz, 2H), 1.96 (s, 1H) ppm; ^{13}C{^{1}H} NMR (100 MHz, $CDCl_3$): δ 132.8, 131.3, 128.8, 127.4, 126.8, 126.6, 125.7, 117.6, 98.0, 79.2, 61.7, 24.7 ppm. The spectral characteristics are consistent with those of **40r** in the literature.[42]

8.2 Preparation of alcohols **41n-r**

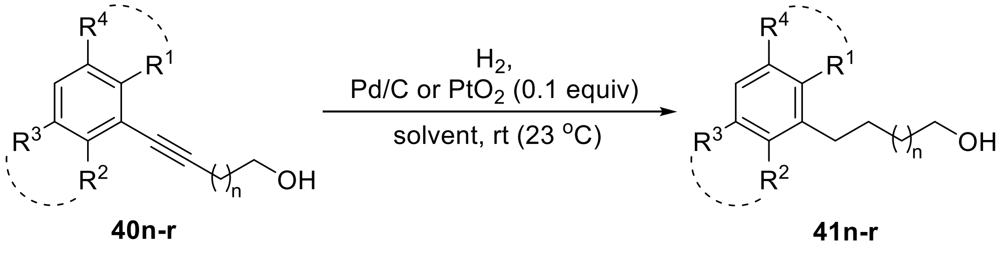

5-(*o*-Tolyl)pentan-1-ol (41n)

To a solution of alkynol **40n** (0.238 g, 1.37 mmol, 1.00 equiv) in 95% EtOH (8.0 mL, 0.17 M) was added Pd/C (5 wt%) (0.291 g, 0.137 mmol, 0.1 equiv). The reaction mixture was stirred under hydrogen using a hydrogen-filled balloon at room temperature until the reaction is complete as judged by TLC analysis. The reaction mixture was filtered through a short pad of Celite and the celite was washed with EtOAc three times. The filtrate was removed of volatiles *in vacuo*. The residue was subjected to flash column chromatography (eluent: 15% EtOAc / hexane) to afford **41n** as an orange oil (0.213 g, 1.20 mmol, 87% yield).

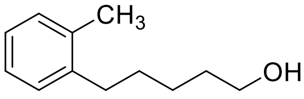

41n

41n: R_f (15% EtOAc in hexane) 0.38; IR (neat) 3340, 2932, 2861, 1460, 1045, 740 cm^{-1}; ^{1}H NMR (400 MHz, $CDCl_3$): δ 7.19 – 7.05 (m, 4H), 3.66 (t, *J* = 6.5, 3.1 Hz, 2H), 2.68 – 2.54 (m, 2H), 2.31 (s, 3H), 1.69 – 1.55 (m, 4H), 1.51 – 1.40 (m, 2H), 1.29 (s, 1H) ppm;

$^{13}C\{^{1}H\}$ NMR (100 MHz, $CDCl_3$): δ 140.9, 135.9, 130.3, 128.9, 126.0, 125.9, 63.1, 33.4, 32.8, 30.2, 25.9, 19.4 ppm; HRMS (EI, 40eV) *m/z* $[M]^+$ calcd for $C_{12}H_{18}O$ 178.1352, found 178.1354.

4-(2,6-Dimethylphenyl)butan-1-ol (41o)

To a solution of alkynol **40o** (0.552 g, 3.17 mmol, 1.00 equiv) in EtOAc (16 mL, 0.2 M) was added PtO_2 (0.144 g, 0.634 mmol, 0.2 equiv). The reaction mixture was stirred under hydrogen using a hydrogen-filled balloon at room temperature until the reaction is complete as judged by TLC analysis. The reaction mixture was filtered through a short pad of Celite and washed with EtOAc three times. The filtrate was removed of volatiles *in vacuo*. The residue was subjected to flash column chromatography (eluent: 20% EtOAc / hexane) to afford **41o** as a pale-yellow oil (0.494 g, 2.77 mmol, 87% yield).

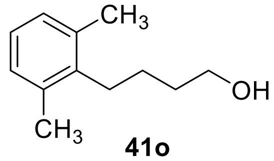

41o

41o: R_f (30% EtOAc in hexane) 0.5; IR (neat) 3352, 2936, 1651, 1468, 1064 cm^{-1}; ^{1}H NMR (600 MHz, $CDCl_3$): δ 7.00 (s, 3H), 3.70 (t, *J* = 6.6 Hz, 2H), 2.69 – 2.61 (m, 2H), 2.32 (s, 6H), 1.74 – 1.67 (m, 2H), 1.59 – 1.51 (m, 2H) ppm; $^{13}C\{^{1}H\}$ NMR (150 MHz, $CDCl_3$): δ 139.4, 136.1, 128.2, 125.7, 63.0, 33.3, 29.6, 25.4, 20.0 ppm; HRMS (EI, 40eV) *m/z* $[M]^+$ calcd for $C_{12}H_{18}O$ 178.1352, found 178.1351.

5-(Naphthalen-1-yl)pentan-1-ol (41p)

To a solution of alkynol **40p** (0.137 g, 0.651 mmol, 1.00 equiv) in MeOH (6.5 mL, 0.1 M) was added Pd/C (5 wt%) (0.14 g, 0.065 mmol, 0.1 equiv). The reaction mixture was stirred under hydrogen using a hydrogen-filled balloon at room temperature until the reaction is complete as judged by TLC analysis. The reaction mixture was filtered through a short pad of Celite and washed with EtOAc three times. The filtrate was removed of volatiles *in vacuo* to afford **41p** as a pale-yellow oil (0.119 g, 0.553 mmol, 85% yield), which was used directly in the next step.

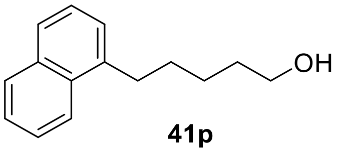

41p

41p: R_f (30% EtOAc / hexane) 0.47; ^{1}H NMR (500 MHz, $CDCl_3$): δ 8.04 (d, *J* = 8.4 Hz, 1H), 7.91 – 7.83 (d, *J* = 8.1 Hz, 1H), 7.71 (d, *J* = 8.2 Hz, 1H), 7.56 – 7.43 (m, 2H), 7.42 – 7.37 (m, 1H), 7.32 (d, *J* = 7.0 Hz, 1H), 3.66 (t, *J* = 6.6 Hz, 2H), 3.09 (t, *J* = 7.9, 1.8 Hz, 2H), 1.86 – 1.73 (m, 2H), 1.69 – 1.60 (m, 2H), 1.59 (s, 1H), 1.57 – 1.42 (m, 2H) ppm; $^{13}C\{^{1}H\}$ NMR (100 MHz, $CDCl_3$): δ 138.8, 134.0, 132.0, 128.9, 126.6, 126.0, 125.8, 125.7, 125.5, 124.0, 63.1, 33.2, 32.8, 30.8, 26.1 ppm. The spectral characteristics are consistent with those of **41p** in the literature.[41]

4-(Naphthalen-1-yl)butan-1-ol (41q)

To a solution of alkynol **40q** (0.787 g, 4.01 mmol, 1.00 equiv) in MeOH (40 mL, 0.1 M) was added Pd/C (5 wt%) (0.853 g, 0.401 mmol, 0.1 equiv). The reaction mixture was stirred under hydrogen using a hydrogen-filled balloon at room temperature until the reaction is complete as judged by TLC analysis. The reaction mixture was filtered through a short pad of Celite and washed with EtOAc three times. The filtrate was removed of volatiles *in vacuo* to afford **41q** as a brown liquid (0.621 g, 3.10 mmol, 77% yield), which was used directly in the next step.

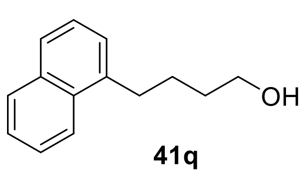

41q

41q: R_f(30% EtOAc / hexane) 0.42; ^{1}H NMR (400 MHz, $CDCl_3$): δ 8.05 (d, J = 8.1 Hz, 1H), 7.86 (d, J = 7.8 Hz, 1H), 7.72 (d, J = 8.2 Hz, 1H), 7.55 – 7.44 (m, 2H), 7.40 (t, J = 7.6 Hz, 1H), 7.33 (d, J = 6.9 Hz, 1H), 3.69 (q, J = 6.0 Hz, 2H), 3.12 (t, J = 7.7 Hz, 2H), 1.89 – 1.78 (m, 2H), 1.77 – 1.64 (m, 2H) ppm; $^{13}C\{^1H\}$ NMR (100 MHz, $CDCl_3$): δ 138.5, 134.0, 132.0, 128.9, 126.7, 126.1, 125.9, 125.6, 125.5, 123.9, 63.0, 32.9, 32.9, 27.0 ppm. The spectral characteristics are consistent with those of **41q** in the literature.[43]

4-(Anthracen-9-yl)butan-1-ol (41r)

To a solution of alkynol **40r** (0.786 g, 3.19 mmol, 1.00 equiv) in 95% EtOH (16 mL, 0.2 M) was added Pd/C (5wt%) (0.679 g, 0.319 mmol, 0.1 equiv). The reaction mixture was stirred under hydrogen using a hydrogen-filled balloon at room temperature until the reaction is complete as judged by TLC analysis. The reaction mixture was filtered through a short pad of Celite and washed with EtOAc three times. The filtrate was removed of volatiles *in vacuo*. The residue was subjected to flash column chromatography (eluent: 10% EtOAc / hexane) to afford **41r** as an orange solid (38.3 mg, 0.153 mmol, 5% yield).

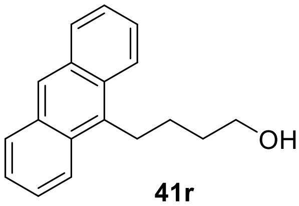

41r

41r: R_f(30% EtOAc / hexane) 0.40; ^{1}H NMR (500 MHz, $CDCl_3$): δ 8.34 (s, 1H), 8.27 (d, J = 8.8 Hz, 2H), 8.01 (d, J = 8.0 Hz, 2H), 7.55 – 7.49 (m, 2H), 7.49 – 7.44 (m, 2H), 3.71 (t, J = 6.2, 1.4 Hz, 2H), 3.68 – 3.61 (m, 2H), 1.95 – 1.86 (m, 2H), 1.85 – 1.74 (m, 2H) ppm; $^{13}C\{^1H\}$ NMR (125 MHz, $CDCl_3$): δ 134.9, 131.7, 129.7, 129.3, 125.8, 125.6, 124.9, 124.5, 62.9, 33.2, 27.7, 27.6 ppm. The spectral characteristics are consistent with those of **41r** in the literature.[44]

8.3 Preparation of aldehydes **42m-r**

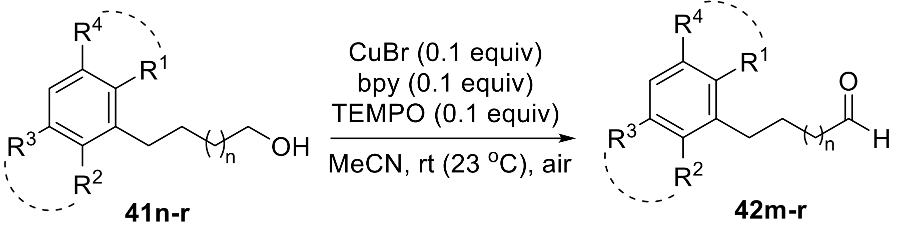

5-Phenylpentanal (42m)

According to the procedure for the synthesis of **22a**, compound **42m** was obtained from the treatment of 5-phenylpentan-1-ol (1.13 g, 6.89 mmol, 1.00 equiv) in MeCN (34 mL, 0.2 M) with CuBr (99 mg, 0.69 mmol, 0.1 equiv), bpy (0.108 g, 0.689 mmol, 0.1 equiv), and TEMPO (0.108 g, 0.689 mmol, 0.1 equiv). The crude material obtained was purified by flash column chromatography (eluent: 10% EtOAc in hexane) to give aldehyde **42m** as a blood orange oil (1.08 g, 6.66 mmol, 97% yield).

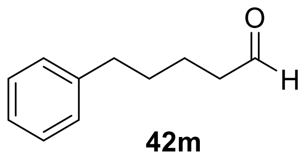

42m

42m: R_f(15% EtOAc / hexane) 0.61; ^{1}H NMR (400 MHz, $CDCl_3$): δ 9.77 (t, J = 1.8 Hz, 1H), 7.34 – 7.24 (m, 2H), 7.23 – 7.11 (m, 3H), 2.66 (t, 2H), 2.47 (t, 2H), 1.79 – 1.57 (m, 4H) ppm; $^{13}C\{^1H\}$ NMR (100 MHz, $CDCl_3$): δ 202.4, 141.8, 128.2, 128.2, 125.7, 43.6, 35.5, 30.7, 21.5 ppm. The spectral characteristics are consistent with those of **42m** in the literature.[45]

5-(*o*-Tolyl)pentanal (42n)

According to the procedure for the synthesis of **22a**, compound **42n** was obtained from the treatment of alcohol **41n** (0.195 g, 1.09 mmol, 1.0 equiv) in MeCN (5.5 mL, 0.2 M) with CuBr (15.7 mg, 0.110 mmol, 0.1 equiv), bpy (17.1 mg, 0.110 mmol, 0.1 equiv), and TEMPO (17.1 mg, 0.110 mmol, 0.1 equiv). The crude material obtained was purified by flash column chromatography (eluent: 10% EtOAc in hexane) to give aldehyde **42n** as a blood orange oil (0.184 g, 1.04 mmol, 96% yield).

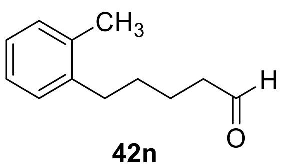

42n

42n: R_f (15% EtOAc in hexane) 0.60; IR (neat) 2936, 2869, 1722, 1461, 742 cm^{-1}; ^1H NMR (400 MHz, $CDCl_3$): δ 9.79 (t, *J* = 1.7 Hz, 1H), 7.20 – 7.07 (m, 4H), 2.71 – 2.58 (m, 2H), 2.49 (td, *J* = 7.2, 1.8 Hz, 2H), 2.32 (s, 3H), 1.80 – 1.68 (m, 2H), 1.67 – 1.54 (m, 2H) ppm; $^{13}C\{^1H\}$ NMR (100 MHz, $CDCl_3$): δ 202.4, 140.0, 135.7, 130.1, 128.7, 125.9, 125.8, 43.7, 32.9, 29.6, 22.0, 19.2 ppm; HRMS (EI, 40eV) *m/z* $[M]^+$ calcd for $C_{12}H_{16}O$ 176.1196, found 176.1196.

4-(2,6-Dimethylphenyl)butanal (42o)

According to the procedure for the synthesis of **22a**, compound **42o** was obtained from the treatment of alcohol **41o** (0.565 g, 3.17 mmol, 1.0 equiv) in MeCN (16 mL, 0.2 M) with CuBr (45.5 mg, 0.317 mmol, 0.1 equiv), bpy (49.5 mg, 0.317 mmol, 0.1 equiv), and TEMPO (49.5 mg, 0.317 mmol, 0.1 equiv). The crude material obtained was purified by flash column chromatography (eluent: 10% EtOAc in hexane) to give aldehyde **42o** as a blood orange oil (0.405 g, 2.30 mmol, 73% yield).

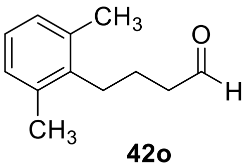

42o

42o: R_f (30% EtOAc in hexane) 0.75; IR (neat) 2951, 2719, 1724, 1469, 770 cm^{-1}; ^1H NMR (500 MHz, $CDCl_3$): δ 9.84 (s, 1H), 7.03 (s, 3H), 2.70 – 2.63 (m, 2H), 2.58 (t, *J* = 8.0, 4.1 Hz, 2H), 2.35 (d, *J* = 2.1 Hz, 6H), 1.88 – 1.77 (m, 2H) ppm; $^{13}C\{^1H\}$ NMR (125 MHz, $CDCl_3$): δ 202.1, 138.2, 135.9, 128.1, 125.8, 43.8, 28.9, 21.4, 19.7 ppm; HRMS (EI, 40eV) *m/z* $[M]^+$ calcd for $C_{12}H_{16}O$ 176.1201, found 176.1197.

5-(Naphthalen-1-yl)pentanal (42p)

According to the procedure for the synthesis of **22a**, compound **42p** was obtained from the treatment of alcohol **41p** (0.306 g, 1.43 mmol, 1.00 equiv) in MeCN (7.1 mL, 0.2 M) with CuBr (20.5 mg, 0.143 mmol, 0.1 equiv), bpy (22.3 mg, 0.143 mmol, 0.1 equiv), and TEMPO (22.3 mg, 0.143 mmol, 0.1 equiv). The crude material obtained was purified by flash column chromatography (eluent: 10% EtOAc in hexane) to give aldehyde **42p** as a blood orange oil (0.215 g, 1.01 mmol, 71% yield).

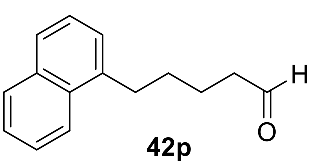

42p

42p: R_f(30% EtOAc / hexane) 0.76; ^1H NMR (400 MHz, $CDCl_3$): δ 9.77 (t, *J* = 1.8 Hz, 1H), 8.02 (d, *J* = 8.4 Hz, 1H), 7.86 (d, *J* = 8.0 Hz, 1H), 7.72 (d, *J* = 8.1 Hz, 1H), 7.54 – 7.45 (m, 2H), 7.40 (dd, *J* = 8.2, 7.0 Hz, 1H), 7.32 (d, *J* = 7.0, 1.2 Hz, 1H), 3.11 (t, *J* = 7.4 Hz, 2H), 2.49 (td, *J* = 7.0, 1.7 Hz, 2H), 1.86 – 1.71 (m, 4H) ppm; $^{13}C\{^1H\}$ NMR (100 MHz, $CDCl_3$): δ 202.5, 138.0, 134.0, 131.8, 128.9, 126.7, 126.0, 125.8, 125.6, 125.5, 123.7, 43.8, 32.9, 30.2, 22.2 ppm. The spectral characteristics are consistent with those of **42p** in the literature.[41]

4-(Naphthalen-1-yl)butanal (42q)

According to the procedure for the synthesis of **22a**, compound **42q** was obtained from the treatment of alcohol **41q** (0.604 g, 3.02 mmol, 1.00 equiv) in MeCN (15 mL, 0.2 M) with CuBr (43.3 mg, 0.302 mmol, 0.1 equiv), bpy (47.2 mg, 0.302 mmol, 0.1 equiv), and TEMPO (47.2 mg, 0.302 mmol, 0.1 equiv). The crude material obtained was purified by flash column chromatography (eluent: 10% EtOAc in hexane) to give aldehyde **42q** as a blood orange oil (0.425 g, 2.14 mmol, 71% yield).

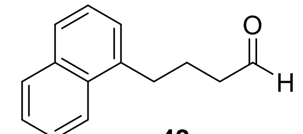

42q

42q: R_f (30% EtOAc / hexane) 0.76; ^{1}H NMR (400 MHz, $CDCl_3$): δ 9.80 (t, *J* = 1.5 Hz, 1H), 8.07 (d, *J* = 8.2 Hz, 1H), 7.93 – 7.84 (m, 1H), 7.75 (d, *J* = 8.2 Hz, 1H), 7.58 – 7.45 (m, 2H), 7.41 (t, *J* = 8.2 Hz, 1H), 7.32 (d, *J* = 7.0 Hz, 1H), 3.14 (t, *J* = 7.7 Hz, 2H), 2.55 (td, *J* = 7.2, 1.5 Hz, 2H), 2.12 (p, *J* = 7.3 Hz, 2H) ppm; ^{13}C{^{1}H} NMR (100 MHz, $CDCl_3$): δ 202.3, 137.4, 133.9, 131.8, 128.8, 127.0, 126.2, 126.0, 125.6, 125.5, 123.7, 43.5, 32.2, 23.0 ppm. The spectral characteristics are consistent with those of **42q** in the literature.[46]

4-(Anthracen-9-yl)butanal (42r)

According to the procedure for the synthesis of **22a**, compound **42r** was obtained from the treatment of alcohol **41r** (0.171 g, 0.682 mmol, 1.0 equiv) in MeCN (3.4 mL, 0.2 M) with CuBr (9.8 mg, 0.068 mmol, 0.1 equiv), bpy (10.7 mg, 0.0682 mmol, 0.1 equiv), and TEMPO (10.7 mg, 0.0682 mmol, 0.1 equiv). The crude material obtained was purified by flash column chromatography (eluent: 10% EtOAc in hexane) to give aldehyde **42r** as an orange solid (0.150 g, 0.604 mmol, 88% yield).

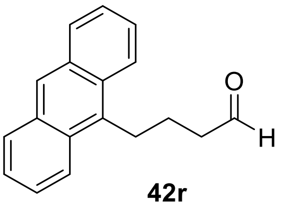

42r

42r: R_f (30% EtOAc in hexane) 0.66; IR (neat) 2824, 1705, 1383, 733 cm^{-1}; ^{1}H NMR (400 MHz, $CDCl_3$): δ 9.85 (t, *J* = 1.4 Hz, 1H), 8.36 (s, 1H), 8.28 (d, *J* = 8.8 Hz, 2H), 8.01 (d, *J* = 8.4 Hz, 2H), 7.57 – 7.49 (m, 2H), 7.50 – 7.40 (m, 2H), 3.74 – 3.58 (m, 2H), 2.66 (td, *J* = 7.0, 1.4 Hz, 2H), 2.24 – 2.10 (m, 2H) ppm; ^{13}C{^{1}H} NMR (150 MHz, $CDCl_3$): δ 202.3, 133.9, 131.7, 129.8, 129.4, 126.2, 125.9, 125.0, 124.4, 43.9, 27.1, 23.5 ppm; HRMS (EI, 40eV) *m/z* [M]$^+$ calcd for $C_{18}H_{16}O$ 248.1196, found 248.1195; m.p. 100.0-101.9 °C.

8.4 Preparation of chloroaldehydes **43m-r**

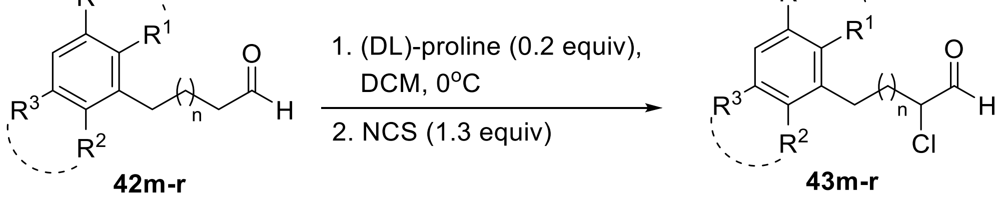

42m-r **43m-r**

2-Chloro-5-phenylpentanal (43m)

According to the procedure for the synthesis of **23a**, compound **43m** was obtained from the treatment of aldehyde **42m** (0.157 g, 0.969 mmol, 1.00 equiv) in anhydrous DCM (1.9 mL, 0.5 M) with (DL)-proline (22 mg, 0.19 mmol, 0.2 equiv) and NCS (0.168 g, 1.26 mmol, 1.3 equiv) at 0 °C. The crude material obtained was purified by flash column chromatography

(eluent: 10% EtOAc in hexane) to afford **43m** as a blood orange oil (0.153 g, 0.777 mmol, 80% yield).

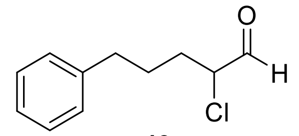
43m

43m: R_f (15% EtOAc / hexane) 0.36; ^{1}H NMR (600 MHz, $CDCl_3$): δ 9.49 (d, J = 2.3 Hz, 1H), 7.30 (m, 2H), 7.24 – 7.16 (m, 3H), 4.18 (ddd, J = 7.7, 5.2, 2.3 Hz, 1H), 2.80 – 2.59 (m, 2H), 2.08 – 1.98 (m, 1H), 1.95 – 1.81 (m, 2H), 1.83 – 1.73 (m, 1H) ppm; $^{13}C\{^1H\}$ NMR (150 MHz, $CDCl_3$): δ 195.2, 141.2, 128.5, 128.4, 126.1, 63.8, 35.1, 31.5, 27.2 ppm. The spectral characteristics are consistent with those of **43m** in the literature.[47]

2-Chloro-5-(*o*-tolyl)pentanal (43n)

According to the procedure for the synthesis of **23a**, compound **43n** was obtained from the treatment of aldehyde **42n** (0.169 g, 0.959 mmol, 1.00 equiv) in anhydrous DCM (1.9 mL, 0.5 M) with (DL)-proline (22 mg, 0.19 mmol, 0.2 equiv) and NCS (0.167 g, 1.25 mmol, 1.3 equiv) at 0 °C. The crude material obtained was purified by flash column chromatography (eluent: 10% EtOAc in hexane) to afford **43n** as a blood orange oil (0.132 g, 0.628 mmol, 65% yield).

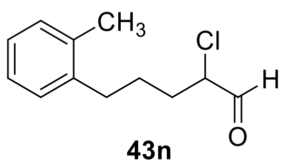
43n

43n: R_f (15% EtOAc in hexane) 0.33; IR (neat) 2944, 1732, 1460, 1029, 743, 608 cm^{-1}; ^{1}H NMR (400 MHz, $CDCl_3$): δ 9.50 (d, J = 2.3 Hz, 1H), 7.19 – 7.08 (m, 4H), 4.19 (td, J = 5.4, 2.6 Hz, 1H), 2.71 – 2.61 (m, 2H), 2.31 (s, 3H), 2.16 – 1.98 (m, 2H), 1.98 – 1.64 (m, 2H) ppm; $^{13}C\{^1H\}$ NMR (100 MHz, $CDCl_3$): δ 195.3, 139.5, 135.9, 130.4, 128.8, 126.3, 126.1, 63.9, 32.6, 31.9, 26.1, 19.4 ppm; HRMS (EI, 40eV) *m/z* $[M]^+$ calcd for $C_{12}H_{15}OCl$ 210.0806, found 210.0800.

2-Chloro-4-(2,6-dimethylphenyl)butanal (43o)

According to the procedure for the synthesis of **23a**, compound **43o** was obtained from the treatment of aldehyde **42o** (0.125 g, 0.710 mmol, 1.00 equiv) in anhydrous DCM (1.4 mL, 0.5 M) with (DL)-proline (16.4 mg, 0.142 mmol, 0.2 equiv) and NCS (0.123 g, 0.923 mmol, 1.3 equiv) at 0 °C. The crude material obtained was purified by flash column chromatography (eluent: 10% EtOAc in hexane) to afford **43o** as a blood orange oil (88.5 mg, 0.420 mmol, 59% yield).

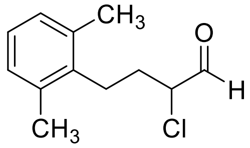
43o

43o: R_f (15% EtOAc in hexane) 0.34; IR (neat) 2963, 2922, 1736, 1468, 770 cm^{-1}; ^{1}H NMR (500 MHz, $CDCl_3$): δ 9.58 (s, 1H), 7.09 – 6.97 (m, 3H), 4.29 (ddd, J = 7.6, 4.9, 1.8 Hz, 1H), 2.91 – 2.72 (m, 2H), 2.34 (s, 6H), 2.21 – 2.07 (m, 1H), 2.05 – 1.90 (m, 1H) ppm; $^{13}C\{^1H\}$ NMR (125 MHz, $CDCl_3$): δ 195.2, 137.0, 136.2, 128.4, 126.3, 64.1, 31.2, 25.6, 19.8 ppm; HRMS (EI, 40eV) *m/z* $[M]^+$ calcd for $C_{12}H_{15}OCl$ 210.0806, found 210.0811.

2-Chloro-5-(naphthalen-1-yl)pentanal (43p)

According to the procedure for the synthesis of **23a**, compound **43p** was obtained from the treatment of aldehyde **42p** (0.191 g, 0.901 mmol, 1.00 equiv) in anhydrous DCM (1.8 mL, 0.5 M) with (DL)-proline (20.8 mg, 0.180 mmol, 0.2 equiv) and NCS (0.156 g, 1.17 mmol, 1.3 equiv) at 0 °C. The crude material obtained was purified by flash column chromatography (eluent: 10% EtOAc in hexane) to afford **43p** as a blood orange oil (0.178 g, 0.721 mmol, 80%

yield).

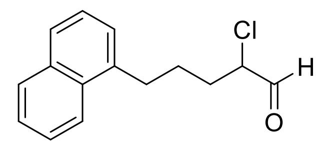

43p

43p: R_f (30% EtOAc in hexane) 0.57; IR (neat) 2934, 2357, 1732, 1595, 968 cm^{-1}; ^1H NMR (400 MHz, $CDCl_3$): δ 9.49 (d, *J* = 2.2 Hz, 1H), 8.01 (d, *J* = 8.2 Hz, 1H), 7.87 (d, *J* = 7.8, 1.7 Hz, 1H), 7.74 (d, *J* = 8.2 Hz, 1H), 7.57 – 7.46 (m, 2H), 7.41 (t, *J* = 7.6 Hz, 1H), 7.32 (d, *J* = 7.0 Hz, 1H), 4.21 (ddd, *J* = 7.8, 5.1, 2.3 Hz, 1H), 3.20 – 3.07 (m, 2H), 2.19 – 1.79 (m, 4H) ppm; $^{13}C\{^1H\}$ NMR (100 MHz, $CDCl_3$): δ 195.3, 137.4, 134.1, 131.8, 129.0, 127.1, 126.2, 126.1, 125.7, 125.6, 123.6, 63.9, 32.4, 32.0, 26.7 ppm; HRMS (EI, 40eV) *m/z* $[M]^+$ calcd for $C_{15}H_{15}OCl$ 246.0806, found 246.0810.

2-Chloro-4-(naphthalen-1-yl)butanal (43q)

According to the procedure for the synthesis of **23a**, compound **43q** was obtained from the treatment of aldehyde **42q** (0.207 g, 1.04 mmol, 1.00 equiv) in anhydrous DCM (2 mL, 0.5 M) with (DL)-proline (24 mg, 0.21 mmol, 0.2 equiv) and NCS (0.181 g, 1.45 mmol, 1.3 equiv) at 0 °C. The crude material obtained was purified by flash column chromatography (eluent: 10% EtOAc in hexane) to afford **43q** as a yellow oil (0.187 g, 0.804 mmol, 77% yield).

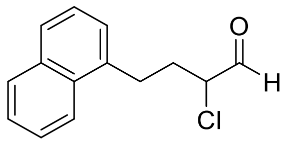

43q

43q: R_f (30% EtOAc in hexane) 0.5; IR (neat) 2953, 2872, 1732, 798 cm^{-1}; ^1H NMR (400 MHz, $CDCl_3$): δ 9.54 (d, *J* = 1.8 Hz, 1H), 8.04 (d, *J* = 8.3 Hz, 1H), 7.87 (d, *J* = 8.1 Hz, 1H), 7.76 (d, *J* = 7.9 Hz, 1H), 7.60 – 7.44 (m, 2H), 7.46 – 7.33 (m, 2H), 4.22 (ddd, *J* = 8.9, 4.6, 1.8 Hz, 1H), 3.49 – 3.34 (m, 1H), 3.27 – 3.18 (m, 1H), 2.51 – 2.38 (m, 1H), 2.31 – 2.19 (m, 1H) ppm; $^{13}C\{^1H\}$ NMR (100 MHz, $CDCl_3$): δ 195.2, 136.0, 134.1, 131.7, 129.1, 127.6, 126.7, 126.4, 125.9, 125.7, 123.5, 63.7, 33.0, 28.8 ppm; HRMS (EI, 40eV) *m/z* $[M]^+$ calcd for $C_{14}H_{13}OCl$ 232.0649, found 232.0651.

4-(Anthracen-9-yl)-2-chlorobutanal (43r)

According to the procedure for the synthesis of **23a**, compound **43r** was obtained from the treatment of aldehyde **42r** (0.102 g, 0.412 mmol, 1 equiv) in anhydrous DCM (0.8 mL, 0.5 M) with (DL)-proline (9.4 mg, 0.082 mmol, 0.2 equiv) and NCS (71.5 mg, 0.536 mmol, 1.3 equiv) at 0 °C. The crude material obtained was purified by flash column chromatography (eluent: 10% EtOAc in hexane) to afford **43r** as a yellow syrup (58.8 mg, 0.208 mmol, 50% yield).

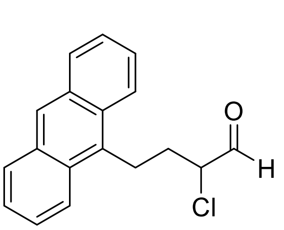

43r

43r: R_f (15% EtOAc in hexane) 0.26; IR (neat) 2922, 2848, 1732, 731 cm^{-1}; ^1H NMR (400 MHz, $CDCl_3$): δ 9.62 (d, *J* = 1.6 Hz, 1H), 8.39 (s, 1H), 8.27 (d, *J* = 8.9 Hz, 2H), 8.03 (d, *J* = 8.5 Hz, 2H), 7.60 – 7.51 (m, 2H), 7.52 – 7.44 (m, 2H), 4.41 (ddd, *J* = 8.1, 4.6, 1.6 Hz, 1H), 3.90 – 3.74 (m, 2H), 2.57 – 2.27 (m, 2H) ppm; $^{13}C\{^1H\}$ NMR (150 MHz, $CDCl_3$): δ 195.2, 132.2, 131.7, 129.8, 129.5, 126.7, 126.2, 125.1, 124.0, 64.1, 33.1, 23.8 ppm; HRMS (EI, 40eV) *m/z* $[M]^+$ calcd for $C_{18}H_{15}OCl$ 282.0806, found 282.0802.

8.5 Preparation of epoxy allylic silanes **25m-r**

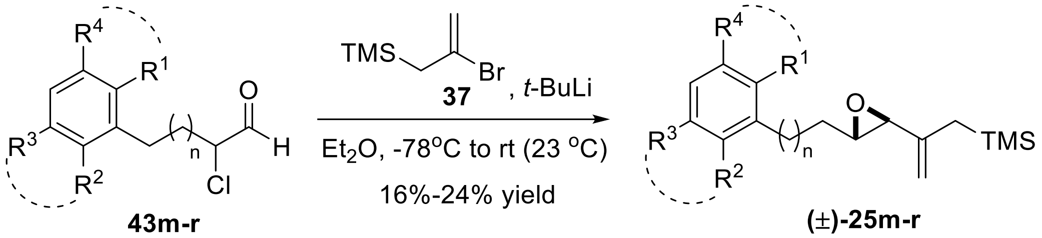

Trimethyl(2-((*2S*,3S)-3-(3-phenylpropyl)oxiran-2-yl)allyl)silane (35m)**

According to the procedure for the synthesis of **25a**, epoxy allylsilane **25m** was obtained from the treatment of α-chloroaldehyde **43m** (0.153 g, 0.780 mmol, 1.00 equiv) in anhydrous Et_2O (2.6 mL, 0.3 M) with **37** (0.271 g, 1.40 mmol, 1.80 equiv) in Et_2O (1.3 mL, 1.1 M) and *tert*-butyllithium (0.81 M in pentanes, 3.6 mL, 2.9 mmol, 3.8 equiv). The crude material obtained was purified by flash column chromatography (eluent: 0.3% Et_2O / hexane) to give **25m** as a pale-yellow oil (37.8 mg, 0.138 mmol, 18% yield).

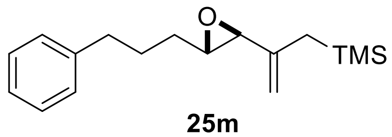

25m

25m: R_f (5% EtOAc / hexane) 0.54; IR (neat) 2952, 1454, 1247, 1157, 841, 735, 697 cm^{-1}; ^1H NMR (400 MHz, $CDCl_3$): δ 7.33 – 7.26 (m, 2H), 7.23 – 7.15 (m, 3H), 4.95 (s, 1H), 4.71 (s, 1H), 3.00 (d, J = 2.1 Hz, 1H), 2.78 (ddd, J = 6.6, 4.7, 2.2 Hz, 1H), 2.67 (t, J = 7.6 Hz, 2H), 1.89 – 1.50 (m, 4H), 1.50 – 1.39 (m, 2H), 0.03 (s, 9H) ppm; $^{13}C\{^1H\}$ NMR (100 MHz, $CDCl_3$): δ 143.6, 142.1, 128.6, 128.5, 126.0, 109.4, 60.6, 59.7, 35.8, 31.8, 27.9, 21.8, -1.2 ppm; HRMS (EI, 40eV) *m/z* $[M]^+$ calcd for $C_{17}H_{26}OSi$ 274.1747, found 274.1746.

Trimethyl(2-((*2R*,3R)-3-(3-(*o*-tolyl)propyl)oxiran-2-yl)allyl)silane (25n)**

According to the procedure for the synthesis of **25a**, epoxy allylsilane **25n** was obtained from the treatment of α-chloroaldehyde **43n** (0.124 g, 0.589 mmol, 1.00 equiv) in anhydrous Et_2O (2 mL, 0.3 M) with **37** (0.171 g, 0.884 mmol, 1.50 equiv) in Et_2O (1 mL, 0.8 M) and *tert*-butyllithium (0.81 M in pentanes, 2.3 mL, 1.9 mmol, 3.2 equiv). The crude material obtained was purified by flash column chromatography (eluent: 0.3% Et_2O / hexane) to give **25n** as a colorless oil (40 mg, 0.13 mmol, 23% yield).

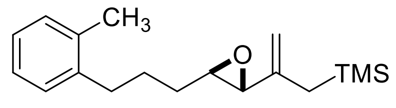

25n

25n: R_f (5% EtOAc in hexane) 0.64; IR (neat) 2951, 1460, 1248, 1157, 841 cm^{-1}; ^1H NMR (500 MHz, $CDCl_3$): δ 7.17 – 7.06 (m, 4H), 4.95 (s, 1H), 4.72 (s, 1H), 3.02 (s, 1H), 2.80 (ddd, J = 6.4, 4.4, 2.1 Hz, 1H), 2.66 (t, J = 7.6 Hz, 2H), 2.31 (s, 3H), 1.83 – 1.54 (m, 4H), 1.50 – 1.39 (m, 2H), 0.03 (s, 9H) ppm; $^{13}C\{^1H\}$ NMR (150 MHz, $CDCl_3$): δ 143.6, 140.3, 136.0, 130.3, 128.9, 126.1, 126.1, 109.4, 60.6, 59.7, 33.1, 32.1, 26.6, 21.8, 19.4, -1.2 ppm; HRMS (EI, 40eV) *m/z* $[M]^+$ calcd for $C_{18}H_{28}OSi$ 288.1904, found 288.1912.

(2-((*2S*,3S)-3-(2,6-Dimethylphenethyl)oxiran-2-yl)allyl)trimethylsilane (25o)**

According to the procedure for the synthesis of **25a**, epoxy allylsilane **25o** was obtained from the treatment of α-chloroaldehyde **43o** (82.8 mg, 0.393 mmol, 1.00 equiv) in anhydrous Et_2O (1.3 mL, 0.3 M) with **37** (0.11 g, 0.59 mmol, 1.5 equiv) in Et_2O (0.6 mL, 0.9 M) and *tert*-

butyllithium (0.81 M in pentanes, 1.5 mL, 1.2 mmol, 3.2 equiv). The crude material obtained was purified by flash column chromatography (eluent: 0.3% Et_2O / hexane) to give **25o** as a pale-yellow oil (17.6 mg, 0.0610 mmol, 16% yield).

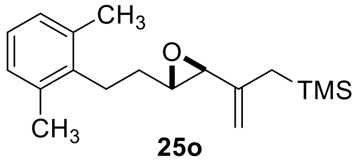

25o

25o: R_f (5% EtOAc in hexane) 0.70; IR (neat) 3021, 2955, 2918, 1631, 1468, 1248,1159 cm^{-1}; ^1H NMR (400 MHz, $CDCl_3$): δ 7.01 (s, 3H), 4.95 (s, 1H), 4.72 (s, 1H), 3.03 (s, 1H), 2.90 – 2.71 (m, 3H), 2.33 (s, 6H), 1.89 – 1.76 (m, 1H), 1.73 – 1.61 (m, 1H), 1.45 (s, 2H), 0.03 (s, 9H) ppm; $^{13}C\{^1H\}$ NMR (100 MHz, $CDCl_3$): δ 143.4, 138.1, 136.2, 128.4, 126.0, 109.3, 60.7, 59.7, 31.6, 26.0, 22.0, 19.9, -1.3 ppm; HRMS (EI, 40eV) *m/z* $[M]^+$ calcd for $C_{18}H_{28}OSi$ 288.1904, found 288.1906.

Trimethyl(2-((*2R*,3R)-3-(3-(naphthalen-1-yl)propyl)oxiran-2-yl)allyl)silane (25p)**

According to the procedure for the synthesis of **25a**, epoxy allylsilane **25p** was obtained from the treatment of α-chloroaldehyde **43p** (0.162 g, 0.658 mmol, 1.00 equiv) in anhydrous Et_2O (2.2 mL, 0.3 M) with **37** (0.19 g, 0.99 mmol, 1.5 equiv) in Et_2O (1.1 mL, 0.9 M) and *tert*-butyllithium (0.81 M in pentanes, 2.6 mL, 2.1 mmol, 3.2 equiv). The crude material obtained was purified by flash column chromatography (eluent: 0.3% Et_2O / hexane) to give **25p** as a colorless oil (48.3 mg, 0.149 mmol, 23% yield).

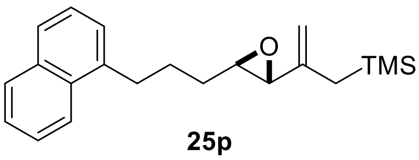

25p

25p: R_f (5% EtOAc in hexane) 0.74; IR (neat) 2951, 2922, 2854, 1250, 850 cm^{-1}; ^1H NMR (400 MHz, $CDCl_3$): δ 8.04 (d, *J* = 8.2 Hz, 1H), 7.86 (d, *J* = 7.5 Hz, 1H), 7.72 (d, *J* = 8.1 Hz, 1H), 7.55 – 7.44 (m, 2H), 7.40 (t, *J* = 7.8 Hz, 1H), 7.33 (d, *J* = 7.1 Hz, 1H), 4.95 (s, 1H), 4.71 (s, 1H), 3.14 (t, *J* = 7.7 Hz, 2H), 3.03 (d, *J* = 2.1 Hz, 1H), 2.82 (ddd, *J* = 6.6, 4.7, 2.1 Hz, 1H), 2.05 – 1.84 (m, 2H), 1.82 – 1.59 (m, 2H), 1.51 – 1.39 (m, 2H), 0.03 (s, 9H) ppm; $^{13}C\{^1H\}$ NMR (150 MHz, $CDCl_3$): δ 143.5, 138.2, 134.1, 132.0, 128.9, 126.8, 126.1, 125.9, 125.7, 125.6, 123.9, 109.4, 60.6, 59.7, 32.9, 32.2, 27.2, 21.8, -1.2 ppm; HRMS (EI, 40eV) *m/z* $[M]^+$ calcd for $C_{21}H_{28}OSi$ 324.1904, found 324.1901.

Trimethyl(2-((*2S*,3S)-3-(2-(naphthalen-1-yl)ethyl)oxiran-2-yl)allyl)silane (25q)**

According to the procedure for the synthesis of **25a**, epoxy allylsilane **25q** was obtained from the treatment of α-chloroaldehyde **43q** (0.174 mg, 0.748 mmol, 1.00 equiv) in anhydrous Et_2O (2.5 mL, 0.3 M) with **37** (0.217 g, 1.12 mmol, 1.50 equiv) in Et_2O (1.2 mL, 0.9 M) and *tert*-butyllithium (0.81 M in pentanes, 2.9 mL, 2.4 mmol, 3.2 equiv). The crude material obtained was purified by flash column chromatography (eluent: 0.3% Et_2O / hexane) to give **25q** as a colorless oil (48.4 mg, 0.156 mmol, 21% yield).

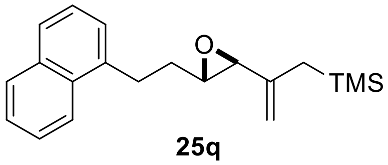

25q

25q: R_f (5% EtOAc in hexane) 0.73; IR (neat) 3046, 2953, 2918, 1633, 1248, 851, 777 cm^{-1}; ^1H NMR (400 MHz, $CDCl_3$): δ 8.04 (d, *J* = 8.2 Hz, 1H), 7.86 (d, *J* = 7.7 Hz, 1H), 7.73 (d, *J* = 8.0 Hz, 1H), 7.55 – 7.45 (m, 2H), 7.43 – 7.33 (m, 2H), 4.92 (s, 1H), 4.70 (s, 1H), 3.38 – 3.27 (m, 1H), 3.24 – 3.13 (m, 1H), 3.01 (d, *J* = 2.1 Hz, 1H), 2.89 (ddd, *J* = 6.7, 4.6, 2.1 Hz, 1H), 2.18 – 2.03 (m, 1H), 2.03 – 1.91 (m, 1H), 1.46 – 1.34 (m, 2H), 0.01 (s, 9H) ppm; $^{13}C\{^1H\}$ NMR (150 MHz, $CDCl_3$): δ 143.4, 137.4, 134.1, 131.9, 129.0, 127.0, 126.2, 126.1, 125.7, 125.7, 123.8, 109.3, 60.8, 59.5, 33.3, 29.4, 21.9, -1.3 ppm; HRMS (EI, 40eV) *m/z* $[M]^+$

calcd for $C_{20}H_{26}OSi$ 310.1747, found 310.1745.

(2-((*2S*,3S)-3-(2-(anthracen-9-yl)ethyl)oxiran-2-yl)allyl)trimethylsilane (25r)**

According to the procedure for the synthesis of **25a**, epoxy allylsilane **25r** was obtained from the treatment of α-chloroaldehyde **43r** (56.7 mg, 0.201 mmol, 1.00 equiv) in anhydrous Et_2O (0.7 mL, 0.3 M) with **37** (0.058 g, 0.30 mmol, 1.5 equiv) in Et_2O (0.3 mL, 1 M) and *tert*-butyllithium (0.81 M in pentanes, 0.78 mL, 0.63 mmol, 3.2 equiv). The crude material obtained was purified by flash column chromatography (eluent: 0.3% Et_2O / hexane) to give **25r** as a pale-yellow oil (14.6 mg, 0.0400 mmol, 20% yield).

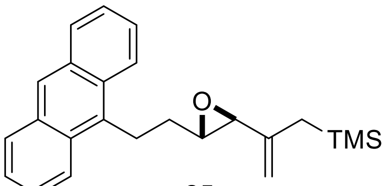

25r

25r: R_f (5% EtOAc in hexane) 0.61; IR (neat) 3053, 2953, 2897, 1247, 881 cm^{-1}; ^1H NMR (400 MHz, $CDCl_3$): δ 8.36 (s, 1H), 8.28 (d, J = 8.7 Hz, 2H), 8.01 (d, J = 8.7 Hz, 2H), 7.56 – 7.42 (m, 4H), 4.93 (s, 1H), 4.70 (s, 1H), 3.86 – 3.73 (m, 2H), 3.07 (d, J = 2.1 Hz, 1H), 2.97 (ddd, J = 7.1, 4.1, 2.1 Hz, 1H), 2.27 – 2.14 (m, 1H), 2.06 – 1.92 (m, 1H), 1.41 – 1.31 (m, 2H), 0.01 (s, 9H). ppm; $^{13}C\{^1H\}$ NMR (150 MHz, $CDCl_3$): δ 143.3, 133.5, 131.8, 129.7, 129.4, 126.2, 125.9, 125.0, 124.3, 109.4, 60.9, 59.6, 33.6, 24.2, 21.8, -1.3 ppm; HRMS (EI, 40eV) m/z $[M]^+$ calcd for $C_{24}H_{28}OSi$ 360.1904, found 360.1914.

9. General Procedure B for the intramolecular (4+3) cycloadditions of (±)-25a-r

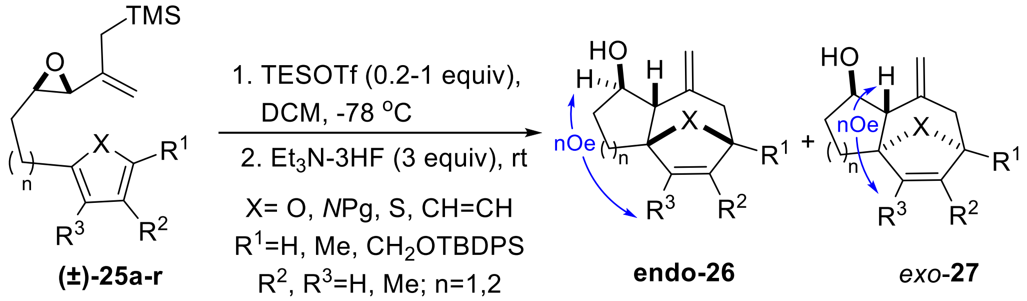

To a solution of epoxy allylsilane **25** (1 equiv) in dry DCM (0.05 M) was added TESOTf (0.2 M solution in DCM, 0.2-1 equiv) at –78 °C. The reaction progress was monitored by TLC. When the reaction is complete, Et_3N-3HF (3 equiv) was added. The mixture was allowed to warm to room temperature with stirring over 1 h. An aqueous solution of saturated $NaHCO_3$ was added with stirring until the effervescence ceased. The resulting mixture was extracted with EtOAc three times. The combined organics were washed with brine and dried over anhydrous $MgSO_4$. The crude product was concentrated *in vacuo* and the residue was purified by flash column chromatography (EtOAc/hexane) to afford the products.

9.1 Reaction of epoxy allylsilane **25a**

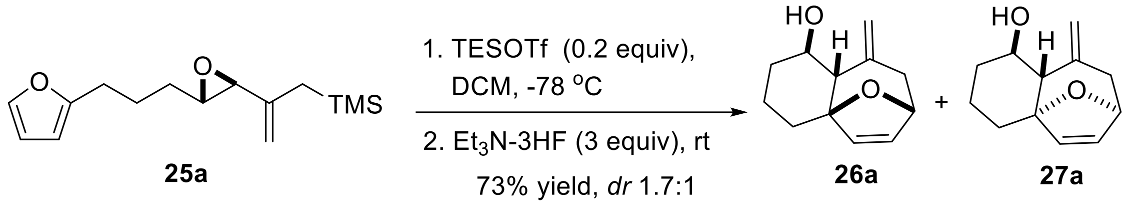

According to General Procedure B, treatment of **25a** (62.4 mg, 0.236 mmol, 1.00 equiv) in DCM (4.8 mL, 0.05 M) with 0.2 M TESOTf in DCM (0.24 mL, 0.05 mmol, 0.2 equiv) afforded a mixture of **26a** + **27a** in a ratio of 1.7:1 as determined by ^{1}H NMR spectroscopy, in the form of a pale yellow oil. The crude material obtained was purified by flash column chromatography (eluent: 20% EtOAc in hexane), which provided **26a** (23.8 mg, 52% yield) and **27a** (9.5 mg, 21% yield).

26a: white solid; R_f(30% EtOAc in hexane) 0.33; IR (neat) 3393, 2922, 2851, 1643, 1450, 1351, 1225, 1160, 1064, 1030, 1000, 969, 952, 904, 883, 866, 828, 803, 739, 673, 643, 532, 452, 407 cm^{-1}; ^{1}H NMR (600 MHz, $CDCl_3$): δ 6.11 (d, *J* = 6.0, 1.8, 0.9 Hz, 1H), 5.99 (d, *J* = 6.0 Hz, 1H), 5.16 (d, *J* = 1.7 Hz, 1H), 4.85 (d, *J* = 1.7 Hz, 1H), 4.78 (dt, *J* = 3.6, 1.7 Hz, 1H), 3.63 – 3.56 (m, 1H), 2.60 (d, *J* = 13.7, 3.4, 1.6 Hz, 1H), 2.24 (d, *J* = 10.2, 1.7 Hz, 1H), 2.14 – 2.05 (m, 1H), 2.04 (dd, *J* = 13.7, 1.6 Hz, 1H), 1.88 – 1.80 (m, 2H), 1.69 – 1.63 (m, 1H), 1.48 – 1.37 (m, 3H) ppm; $^{13}C\{^1H\}$ NMR (150 MHz, $CDCl_3$): δ 143.4, 132.4, 131.3, 112.1, 87.3, 79.3, 69.3, 57.1, 39.0, 35.3, 33.2, 20.5 ppm; EI-MS (20 eV) *m/z* 192 (M^+, 13), 145 (100), 91 (44); HRMS (EI, 40eV) *m/z* $[M]^+$ calcd for $C_{12}H_{16}O_2$ 192.1145, found 192.1141; m.p. 66.5-69.1 °C.

27a: colorless oil; R_f(30% EtOAc in hexane) 0.38; IR (neat) 3401, 2926, 2853, 1644, 1450, 1161, 1072, 1023, 986, 944, 906, 861, 817, 722, 649 cm^{-1}; ^{1}H NMR (600 MHz, $CDCl_3$): δ 6.09 (dd, *J* = 5.9, 1.8 Hz, 1H), 5.93 (d, *J* = 5.9 Hz, 1H), 4.91 (t, *J* = 2.2 Hz, 1H), 4.89 (t, *J* = 2.3 Hz, 1H), 4.86 (dt, *J* = 3.8, 1.6 Hz, 1H), 3.84 – 3.77 (m, 1H), 2.70 – 2.64 (m, 1H), 2.15 – 2.06 (m, 1H), 2.04 (dd, *J* = 14.6, 1.4 Hz, 1H), 1.89 (d, *J* = 1.8 Hz, 1H), 1.87 – 1.81 (m, 2H), 1.75 – 1.68 (m, 3H), 1.65 – 1.60 (m, 1H) ppm; $^{13}C\{^1H\}$ NMR (150 MHz, $CDCl_3$): δ 142.7, 135.9, 132.3, 116.0, 86.0, 79.8, 67.7, 55.4, 34.5, 32.6, 32.5, 19.7 ppm; EI-MS (20 eV) *m/z* 192 (M^+, 9), 162 (45), 133 (48), 119 (100), 91 (87); HRMS (EI, 40eV) *m/z* $[M]^+$ calcd for $C_{12}H_{16}O_2$ 192.1145, found 192.1147.

9.2 Reaction of epoxy allylsilane **25b**

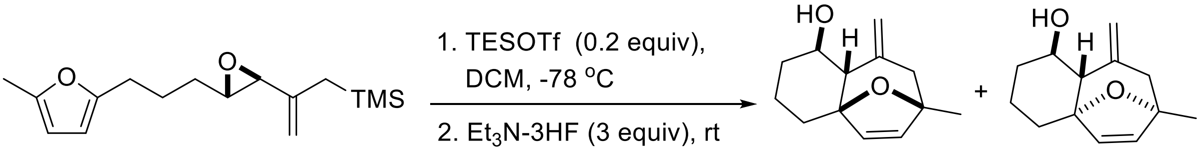

According to General Procedure B, treatment of **25b** (33.2 mg, 0.12 mmol, 1 equiv) in DCM (2.4 mL, 0.05M) with 0.2 M TESOTf in DCM (0.12 mL, 0.02 mmol, 0.2 equiv) afforded a mixture of **26b** + **27b** in a ratio of 1.1:1 as determined by ^{1}H NMR spectroscopy, in the form of pale yellow oil. The crude material obtained was purified by flash column chromatography (eluent: 15% EtOAc in hexane), which provided **26b** (10.4 mg, 42% yield) and **27b** (6.5 mg, 26% yield).

26b: white solid; R_f(15% EtOAc in hexane) 0.18; IR (neat) 3401, 2929, 1643, 1450, 1377,

1217, 1164, 1078, 1049, 1024, 978, 884, 866, 820, 742, 628 cm^{-1}; ^{1}H NMR (600 MHz, $CDCl_3$): δ 5.93 – 5.87 (m, 2H), 5.13 (q, *J* = 1.7 Hz, 1H), 4.83 (q, *J* = 1.8 Hz, 1H), 3.60 – 3.55 (m, 1H), 2.38 (dq, *J* = 13.6, 1.7 Hz, 1H), 2.19 – 2.14 (m, 1H), 2.12 – 2.05 (m, 2H), 1.88 – 1.80 (m, 2H), 1.68 – 1.54 (m, 1H), 1.45 – 1.38 (m, 2H), 1.26 (s, 3H) ppm; ^{13}C{^{1}H} NMR (150 MHz, $CDCl_3$): δ 144.7, 135.8, 131.2, 111.3, 87.8, 84.6, 69.5, 56.1, 45.2, 35.3, 33.5, 29.7, 20.4 ppm; EI-MS (20 eV) *m/z* 206 (M^+, 8), 188 (M^+-H_2O, 68), 145 (100); HRMS (EI, 40eV) *m/z* $[M]^+$ calcd for $C_{13}H_{18}O_2$ 206.1301, found 206.1302; m.p. 61.7-64.9 °C.

27b: colorless oil; R_f(15% EtOAc in hexane) 0.27; IR (neat) 3420, 2928, 1641, 137j7, 1338, 1244, 1195, 1176, 1143, 1078, 1023, 976, 896, 866, 741, 653, 564 cm^{-1}; ^{1}H NMR (600 MHz, $CDCl_3$): δ 5.87 – 5.83 (m, 2H), 4.88 (dt, *J* = 10.4, 2.2 Hz, 2H), 3.76 (ddd, *J* = 11.1, 9.7, 4.3 Hz, 1H), 2.41 (dt, *J* = 14.5, 2.4 Hz, 1H), 2.13 – 2.07 (m, 2H), 1.89 (s, 1H), 1.88 – 1.83 (m, 1H), 1.81 (d, *J* = 9.7 Hz, 1H), 1.77 – 1.64 (m, 2H), 1.63 – 1.55 (m, 2H), 1.41 (s, 3H) ppm; ^{13}C{^{1}H} NMR (150 MHz, $CDCl_3$): δ 143.9, 135.8, 135.6, 115.2, 86.7, 85.1, 67.6, 54.4, 40.7, 32.7, 32.6, 23.5, 19.7 ppm; EI-MS (20 eV) *m/z* 206 (M^+, 25), 145 (100), 135 (45), 121 (12); HRMS (EI, 40eV) *m/z* $[M]^+$ calcd for $C_{13}H_{18}O_2$ 206.1301, found 206.1303.

9.3 Reaction of epoxy allylsilane **25c**

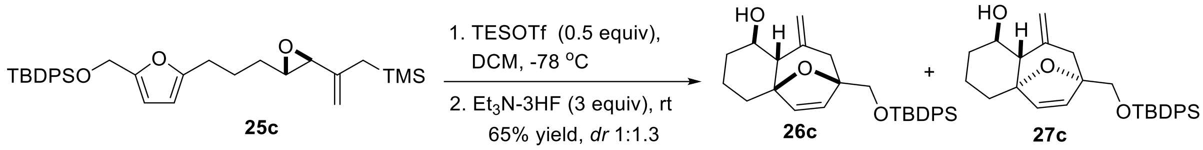

According to General Procedure B, treatment of **25c** (6.3 mg, 0.012 mmol, 1.00 equiv) in DCM (0.24 mL, 0.05 M) with 0.2 M TESOTf in DCM (0.03 mL, 0.006 mmol, 0.5 equiv) afforded a mixture of **26c** + **27c** in a ratio of 1:1.3 as determined by ^{1}H NMR spectroscopy, in the form of pale yellow oil. The crude material obtained was purified by flash column chromatography (eluent: 15% EtOAc in hexane), which provided **26c** (2 mg, 36 % yield) and **27c** (1.6 mg, 29% yield).

26c: colorless oil; R_f(10% EtOAc in hexane) 0.18; IR (neat) 3489, 2930, 2857, 1428, 1109, 701 cm^{-1}; ^{1}H NMR (600 MHz, $CDCl_3$): δ 7.67 (dt, *J* = 6.8, 1.5 Hz, 4H), 7.45 – 7.34 (m, 6H), 6.06 (d, *J* = 6.0, 0.8 Hz, 1H), 5.93 (d, *J* = 6.0 Hz, 1H), 5.16 (d, *J* = 1.8 Hz, 1H), 4.88 (d, *J* = 1.8 Hz, 1H), 3.78 (q, 2H), 3.61 – 3.58 (m, 1H), 2.38 (dq, *J* = 13.5, 1.6 Hz, 1H), 2.25 (d, *J* = 13.5 Hz, 1H), 2.22 – 2.17 (m, 1H), 2.11 – 2.05 (m, 1H), 1.86 – 1.81 (m, 2H), 1.69 – 1.58 (m, 1H), 1.47 – 1.36 (m, 2H), 1.07 (s, 9H) ppm; ^{13}C{^{1}H} NMR (150 MHz, $CDCl_3$): δ 144.4, 135.7, 133.5, 131.3, 129.7, 127.6, 111.7, 87.9, 87.9, 69.5, 67.0, 56.5, 40.8, 35.3, 33.3, 29.7, 26.9, 20.4, 19.4 ppm; HRMS (EI, 40eV) *m/z* $[M-^tBu]^+$ calcd for $C_{25}H_{27}O_3Si$ 403.1724, found 403.1716.

27c: colorless oil; R_f(10% EtOAc in hexane) 0.31; IR (neat) 3479, 2930, 2859, 1639, 1461, 1108, 741 cm^{-1}; ^{1}H NMR (600 MHz, $CDCl_3$): δ 7.69 (ddd, *J* = 7.7, 6.0, 1.5 Hz, 4H), 7.45 – 7.39 (m, 2H), 7.40 – 7.34 (m, 4H), 6.01 (d, *J* = 5.9 Hz, 1H), 5.88 (d, *J* = 5.8 Hz, 1H), 4.94 (t, *J* = 2.1 Hz, 1H), 4.91 (t, *J* = 2.3 Hz, 1H), 3.80 (s, 2H), 3.75 (td, *J* = 11.1, 9.6, 4.3 Hz, 1H), 2.51 (dt, *J* = 14.5, 2.4 Hz, 1H), 2.23 (d, *J* = 14.4 Hz, 1H), 2.12 – 2.07 (m, 1H), 1.90 (s, 1H), 1.86 – 1.80 (m, 2H), 1.74 – 1.64 (m, 2H), 1.60 (dd, *J* = 13.4, 5.3 Hz, 1H), 1.30 – 1.26 (m, 1H), 1.07 (s, 9H) ppm; ^{13}C{^{1}H} NMR (150 MHz, $CDCl_3$): δ 143.8, 136.1, 135.7, 133.5, 133.1, 129.6, 127.6, 115.5, 88.5, 86.7, 67.7, 67.3, 54.9, 36.1, 32.6, 32.5, 26.8, 19.7, 19.4 ppm; HRMS (EI, 40eV) *m/z* $[M-^tBu]^+$ calcd for $C_{25}H_{27}O_3Si$ 403.1724, found 403.1723.

9.4 Reaction of epoxy allylsilane **25d**

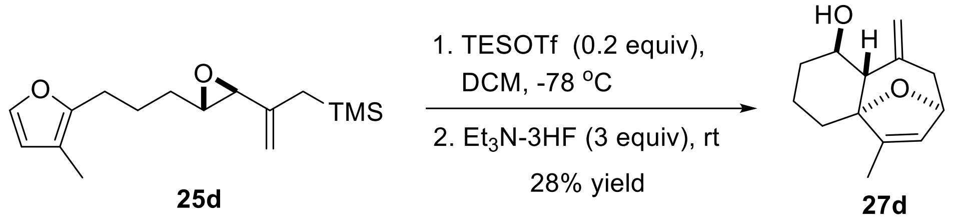

According to General Procedure B, **25d** (5.0 mg, 0.018 mmol, 1.0 equiv) in DCM (0.6 mL, 0.05 M) was treated with 0.2 M TESOTf in DCM (0.03 mL, 0.004 mmol, 0.2 equiv). The crude material obtained was purified by flash column chromatography (eluent: 15% EtOAc in hexane) to afford **27d** (1 mg, 28% yield).

27d: colorless oil; R_f(5% EtOAc in hexane) 0.31; IR (neat) 3414, 2923, 2853, 1649, 1457, 1075, 989 cm^{-1}; ^{1}H NMR (600 MHz, $CDCl_3$): δ 5.68 (s, 1H), 4.88 (d, *J* = 2.2 Hz, 1H), 4.86 (d, *J* = 2.3 Hz, 1H), 4.74 – 4.71 (m, 1H), 3.81 (td, *J* = 10.4, 4.3 Hz, 1H), 2.58 (d, *J* = 14.6, 4.3, 2.4 Hz, 1H), 2.12 (dt, *J* = 12.4, 3.8 Hz, 1H), 1.96 (d, *J* = 14.5 Hz, 1H), 1.90 (s, 1H), 1.85 (d, *J* = 9.6 Hz, 1H), 1.76 – 1.66 (m, 3H), 1.60 – 1.54 (m, 5H) ppm; $^{13}C\{^1H\}$ NMR (150 MHz, $CDCl_3$): δ 143.8, 143.1, 126.1, 115.7, 86.7, 78.0, 67.7, 54.0, 33.6, 32.6, 30.8, 19.7, 11.8 ppm; HRMS (EI, 40eV) *m/z* $[M]^+$ calcd for $C_{13}H_{18}O_2$ 206.1301, found 206.1298.

9.5 Reaction of epoxy allylsilane **25e**

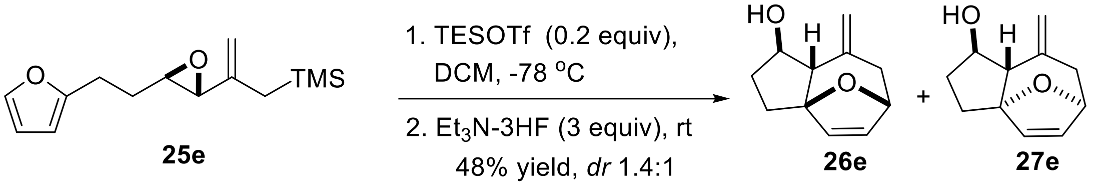

According to General Procedure B, treatment of **25e** (32.6 mg, 0.130 mmol, 1.00 equiv) in DCM (2.6 mL, 0.05 M) with 0.2 M TESOTf in DCM (0.13 mL, 0.030 mmol, 0.2 equiv) afforded a mixture of **26e** + **27e** in a ratio of 1.4:1 as determined by ^{1}H NMR spectroscopy, in the form of pale yellow oil. The crude material obtained was purified by flash column chromatography (eluent: 15% EtOAc in hexane), which provided **26e** (6.3 mg, 27 % yield) and **27e** (5.0 mg, 21% yield).

26e: colorless oil; R_f(30% EtOAc in hexane) 0.20; IR (neat) 3226, 2951, 1650, 1347, 1159, 1051, 883, 738, cm^{-1}; ^{1}H NMR (600 MHz, $CDCl_3$): δ 6.13 (dd, *J* = 5.9, 1.9 Hz, 1H), 5.86 (d, *J* = 5.9 Hz, 1H), 4.94 (d, *J* = 2.0 Hz, 1H), 4.84 (dt, *J* = 3.6, 1.7 Hz, 1H), 4.81 (d, *J* = 2.0 Hz, 1H), 4.01 (dt, *J* = 9.5, 4.7 Hz, 1H), 2.49 – 2.43 (m, 2H), 2.30 – 2.21 (m, 1H), 2.21 – 2.06 (m, 1H), 1.95 – 1.84 (m, 2H), 1.79 (dddd, *J* = 14.4, 11.9, 5.6, 2.8 Hz, 1H), 1.69 (s, 1H) ppm; $^{13}C\{^1H\}$ NMR (150 MHz, $CDCl_3$): δ 143.5, 133.4, 132.4, 110.2, 89.8, 80.1, 70.8, 58.6, 34.3, 31.4, 28.8 ppm; ESI-HRMS *m/z* calcd for $C_{11}H_{15}O_2$ $[M+H]^+$: 179.1066, found 179.1066.

27e: colorless oil; R_f(30% EtOAc in hexane) 0.23; IR (neat) 3499, 2931, 2899, 1649, 1352, 1058, 906, 721 cm^{-1}; ^{1}H NMR (600 MHz, $CDCl_3$): δ 6.14 (dd, *J* = 5.9, 1.8 Hz, 1H), 6.08 (d, *J* = 5.9 Hz, 1H), 4.93 (t, *J* = 2.3 Hz, 2H), 4.76 (d, *J* = 4.1 Hz, 1H), 4.33 (q, *J* = 8.3 Hz, 1H), 2.68 – 2.61 (m, 1H), 2.34 (dddd, *J* = 13.6, 9.5, 8.3, 5.6 Hz, 1H), 2.20 (ddd, *J* = 14.5, 12.2, 5.7 Hz, 1H), 2.11 (dd, 2H), 1.87 (ddd, *J* = 14.7, 9.5, 5.4 Hz, 1H), 1.83 – 1.79 (m, 1H), 1.73 – 1.62 (m, 1H) ppm; $^{13}C\{^1H\}$ NMR (150 MHz, $CDCl_3$): δ 141.8, 134.7, 133.2, 115.7, 91.9, 78.7, 74.3, 58.4, 33.2, 31.2, 29.5 ppm; ESI-HRMS *m/z* calcd for $C_{11}H_{15}O_2$ $[M+H]^+$:179.1066, found 179.1065.

9.6 Reaction of epoxy allylsilane **25f**

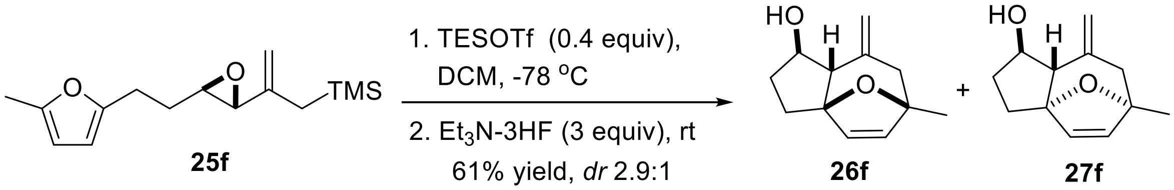

According to General Procedure B, treatment of **25f** (7.5 mg, 0.028 mmol, 1.00 equiv) in DCM (0.6 mL, 0.05 M) with 0.2 M TESOTf in DCM (0.06 mL, 0.02 mmol, 0.4 equiv) afforded a mixture of **26f** + **27f** in a ratio of 2.9:1 as determined by ^{1}H NMR spectroscopy, in the form of pale yellow oil. The crude material obtained was purified by flash column chromatography (eluent: 15% EtOAc in hexane), which provided **26f** (1.6 mg, 30% yield) and **27f** (1.7 mg, 31% yield).

26f: white solid; R_f (30% EtOAc in hexane) 0.24; IR (neat) 3220, 2928, 1650, 1342, 1059, 893 cm^{-1}; ^{1}H NMR (600 MHz, $CDCl_3$): δ 5.91 (d, *J* = 5.8, 0.8 Hz, 1H), 5.79 (d, *J* = 5.8 Hz, 1H), 4.91 (d, 1H), 4.79 (d, *J* = 2.0 Hz, 1H), 4.00 (td, *J* = 9.3, 5.6 Hz, 1H), 2.42 (d, *J* = 9.9, 1.9 Hz, 1H), 2.32 – 2.22 (m, 3H), 2.15 – 2.07 (m, 1H), 1.98 (d, *J* = 14.2 Hz, 1H), 1.90 (ddd, *J* = 12.2, 9.0, 2.9 Hz, 1H), 1.80 (dddd, *J* = 14.1, 12.1, 5.6, 2.8 Hz, 1H), 1.44 (s, 3H) ppm; ^{13}C{^{1}H} NMR (150 MHz, $CDCl_3$): δ 144.3, 136.7, 132.4, 109.4, 90.6, 85.9, 70.8, 57.2, 40.8, 31.6, 29.0, 22.8 ppm; ESI-HRMS *m/z* calcd for $C_{12}H_{17}O_2$ [M+H]$^+$: 193.1223, found 193.1213; m.p. 44.6-47.9 °C.

27f: colorless oil; R_f (30% EtOAc in hexane) 0.30; IR (neat) 3392, 2923, 1642, 1455, 1376, 1342, 1071, 879, cm^{-1}; ^{1}H NMR (600 MHz, $CDCl_3$): δ 6.00 (d, *J* = 5.8 Hz, 1H), 5.92 (d, *J* = 5.7 Hz, 1H), 4.93 – 4.89 (m, 2H), 4.31 (q, *J* = 8.3 Hz, 1H), 2.40 (dtd, *J* = 15.1, 2.6, 1.0 Hz, 1H), 2.34 (dddd, *J* = 13.6, 9.5, 8.3, 5.6 Hz, 1H), 2.23 – 2.14 (m, 2H), 2.09 (d, *J* = 9.1 Hz, 1H), 1.88 (ddd, *J* = 14.7, 9.5, 5.4 Hz, 1H), 1.78 (s, 1H), 1.71 – 1.62 (m, 1H), 1.38 (s, 3H) ppm; ^{13}C{^{1}H} NMR (150 MHz, $CDCl_3$): δ 142.8, 136.5, 134.5, 114.9, 92.8, 84.4, 74.2, 57.4, 39.6, 31.2, 29.7, 24.0 ppm; ESI-HRMS *m/z* calcd for $C_{12}H_{17}O_2$ [M+H]$^+$: 193.1223, found 193.1221.

9.7 Reaction of epoxy allylsilane **25g**

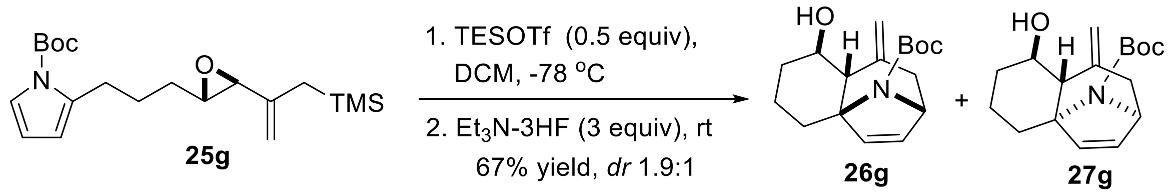

According to General Procedure B, treatment of **25g** (10.1 mg, 0.0275 mmol, 1.00 equiv) in DCM (0.55 mL, 0.05 M) with 0.2 M TESOTf in DCM (0.07 mL, 0.01 mmol, 0.5 equiv) afforded a mixture of **26g** + **27g** in a ratio of 1.9:1 as determined by ^{1}H NMR spectroscopy, in the form of pale yellow oil. The crude material obtained was purified by flash column chromatography (eluent: 15% EtOAc in hexane), which provided **26g** (3.9 mg, 48% yield) and **27g** (1.5 mg, 19% yield).

26g: pale yellow oil; R_f (30% EtOAc in hexane) 0.45; IR (neat) 3453, 2930, 1639, 1688, 1366, 1302, 1253, 1159, 774 cm^{-1}; ^{1}H NMR (600 MHz, $CDCl_3$): δ 5.97 (dd, *J* = 6.4, 2.6 Hz, 1H), 5.92 (d, *J* = 6.3 Hz, 1H), 5.27 (s, 1H), 4.92 (d, *J* = 1.8 Hz, 1H), 4.54 (s, 1H), 3.64 (ddd, *J* = 10.5, 8.0, 4.5 Hz, 1H), 2.62 – 2.48 (m, 2H), 2.06 (ddd, *J* = 14.2, 8.5, 2.6 Hz, 2H), 1.84 – 1.76 (m, 1H), 1.67 (ddt, *J* = 13.2, 4.4, 2.1 Hz, 1H), 1.53 – 1.35 (m, 11H), 1.32 – 1.17 (m, 2H) ppm;

$^{13}C\{^1H\}$ NMR (150 MHz, $CDCl_3$): δ 153.2, 144.1, 133.2, 130.0, 113.8, 69.9, 69.2, 61.0, 53.1, 35.2, 31.9, 28.5, 22.7, 20.8, 14.1 ppm; HRMS (EI, 40eV) *m/z* $[M]^+$ calcd for $C_{17}H_{25}NO_3$ 291.1834, found 291.1836.

27g: pale yellow oil; R_f (30% EtOAc in hexane) 0.50; IR (neat) 3473, 2928, 1703, 1642, 1365, 1162, 977, 848 cm^{-1}; ^1H NMR (600 MHz, $CDCl_3$): δ 6.00 (dd, J = 5.9, 2.5 Hz, 1H), 5.87 (d, J = 5.9 Hz, 1H), 4.98 (t, J = 2.1 Hz, 1H), 4.93 – 4.88 (m, 2H), 3.56 (td, J = 10.5, 3.9 Hz, 1H), 2.63 – 2.48 (m, 3H), 2.42 – 2.30 (m, 1H), 2.19 (dd, J = 14.9, 2.5 Hz, 1H), 2.16 – 2.13 (m, 1H), 1.94 – 1.85 (m, 2H), 1.79 – 1.71 (m, 1H), 1.63 (ddd, J = 14.2, 13.1, 5.1 Hz, 1H), 1.45 (s, 9H) ppm; $^{13}C\{^1H\}$ NMR (150 MHz, $CDCl_3$): δ 152.9, 142.6, 139.5, 132.0, 117.7, 79.7, 69.4, 67.1, 61.0, 58.3, 35.0, 33.3, 30.6, 28.5, 21.4 ppm; HRMS (EI, 40eV) *m/z* $[M]^+$ calcd for $C_{17}H_{25}NO_3$ 291.1934, found 291.1835.

9.8 Reaction of epoxy allylsilane **25h**

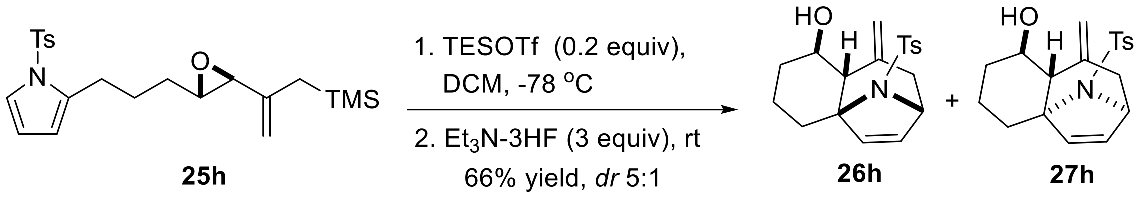

According to General Procedure B, treatment of **25h** (25.6 mg, 0.0610 mmol, 1.00 equiv) in DCM (1.2 mL, 0.05 M) with 0.2 M TESOTf in DCM (0.06 mL, 0.01 mmol, 0.2 equiv) afforded a mixture of **26h** + **27h** in a ratio of 5:1 as determined by ^1H NMR spectroscopy, in the form of pale yellow oil. The crude material obtained was purified by flash column chromatography (eluent: 30% EtOAc in hexane), which provided **26h** (11.3 mg, 54 % yield) and **27h** (2.5 mgl, 12% yield).

26h: pale yellow oil; R_f (30% EtOAc in hexane) 0.26; IR (neat) 3523, 2933, 1637, 1345, 1103, 877, 696 cm^{-1}; ^1H NMR (600 MHz, $CDCl_3$): δ 7.66 (d, J = 8.2 Hz, 2H), 7.23 (d, J = 8.0 Hz, 2H), 6.02 (dd, J = 6.2, 2.6 Hz, 1H), 5.82 (d, J = 6.1 Hz, 1H), 5.27 – 5.24 (m, 1H), 4.93 (s, 1H), 4.86 – 4.82 (m, 1H), 3.59 – 3.51 (m, 1H), 2.85 (d, J = 14.1, 3.3, 1.6 Hz, 1H), 2.68 (d, J = 9.9 Hz, 1H), 2.40 (s, 3H), 2.27 – 2.17 (m, 2H), 2.07 – 2.01 (m, 1H), 1.80 – 1.72 (m, 1H), 1.59 – 1.38 (m, 2H), 1.44 – 1.22 (m, 2H) ppm; $^{13}C\{^1H\}$ NMR (150 MHz, $CDCl_3$): δ 143.4, 142.9, 140.2, 132.6, 131.4, 129.4, 127.0, 114.3, 72.1, 69.4, 63.5, 56.5, 39.9, 34.7, 29.3, 21.5, 20.6 ppm; HRMS (EI, 40eV) *m/z* $[M]^+$ calcd for $C_{19}H_{23}NO_3S$ 345.1393, found 345.1390.

27h: pale yellow oil; R_f (30% EtOAc in hexane) 0.42; IR (neat) 3492, 2925, 1646, 1400, 1357, 1172, 812, 672, cm^{-1}; ^1H NMR (600 MHz, $CDCl_3$): δ 7.62 (d, 2H), 7.29 (d, J = 8.1 Hz, 2H), 7.23 (d, J = 3.4 Hz, 1H), 6.05 (d, J = 3.3 Hz, 1H), 4.97 (s, 1H), 4.88 (s, 1H), 3.98 (td, J = 7.9, 5.5, 2.2 Hz, 1H), 3.45 (s, 1H), 3.05 – 2.96 (m, 1H), 2.79 – 2.72 (m, 1H), 2.42 (s, 4H), 1.93 (ddt, J = 11.1, 7.8, 5.7 Hz, 1H), 1.80 – 1.76 (m, 3H), 1.50 – 1.37 (m, 3H) ppm; $^{13}C\{^1H\}$ NMR (150 MHz, $CDCl_3$): δ 145.2, 144.9, 136.6, 132.6, 130.0, 126.6, 124.4, 121.0, 113.9, 113.8, 70.8, 50.9, 36.9, 25.2, 23.4, 22.2, 21.7 ppm; HRMS (EI, 40eV) *m/z* $[M]^+$ calcd for $C_{19}H_{23}NO_3S$ 345.1393, found 345.1388.

9.9 Reaction of epoxy allylsilane **25i**

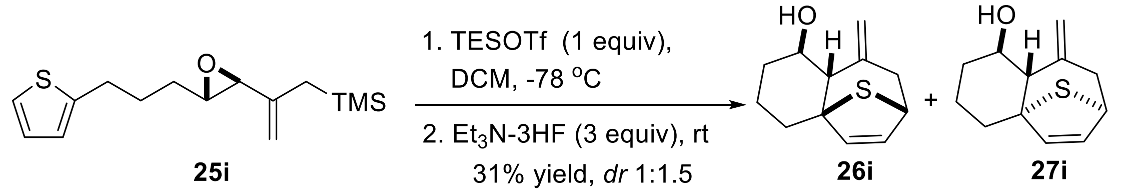

According to General Procedure B, treatment of **25i** (15.7 mg, 0.055 mmol, 1.0 equiv) in DCM (1.1 mL, 0.05 M) with 0.2 M TESOTf in DCM (0.27 mL, 0.053 mmol, 1 equiv) afforded a mixture of **26i** + **27i** in a ratio of 1:1.5 as determined by ^{1}H NMR spectroscopy, in the form of pale yellow oil. The crude material obtained was purified by flash column chromatography (eluent: 15% EtOAc in hexane), which provided **26i** (1 mg, 9 % yield) and **27i** (2.5 mg, 22% yield).

26i: pale yellow oil; R_f(10% EtOAc in hexane) 0.38; IR (neat) 3499, 2924, 1634, 1454, 1334, 1051, 855 cm^{-1}; ^{1}H NMR (600 MHz, $CDCl_3$): δ 6.12 (dd, *J* = 6.2, 3.6 Hz, 1H), 5.94 (d, *J* = 6.2 Hz, 1H), 5.23 (s, 1H), 4.93 (s, 1H), 3.73 – 3.69 (m, 1H), 3.62 – 3.54 (m, 1H), 2.96 (d, *J* = 14.1, 1.8 Hz, 1H), 2.81 (d, *J* = 9.9, 1.7 Hz, 1H), 2.53 (dd, 1H), 2.12 – 2.07 (m, 1H), 2.04 – 1.98 (m, 1H), 1.82 – 1.77 (m, 1H), 1.71 – 1.62 (m, 2H), 1.55 – 1.37 (m, 2H) ppm; ^{13}C{^{1}H} NMR (150 MHz, $CDCl_3$): δ 146.2, 135.1, 134.3, 114.2, 69.5, 60.6, 59.4, 46.8, 42.6, 35.0, 34.4, 21.5 ppm; HRMS (EI, 40eV) *m/z* [M]$^+$ calcd for $C_{12}H_{16}OS$ 208.0916, found 208.0922.

27i: pale yellow oil; R_f(30% EtOAc in hexane) 0.45; IR (neat) 3370, 2930, 1627, 1445, 1246, 1031 cm^{-1}; ^{1}H NMR (600 MHz, $CDCl_3$): δ 6.16 (dd, *J* = 6.1, 3.6 Hz, 1H), 5.90 (d, *J* = 6.1 Hz, 1H), 4.97 (d, *J* = 2.3 Hz, 1H), 4.94 (d, *J* = 2.4 Hz, 1H), 4.05 (td, *J* = 10.0, 4.4 Hz, 1H), 3.76 (d, *J* = 3.3 Hz, 1H), 2.86 (d, *J* = 15.3, 2.9 Hz, 1H), 2.50 (dd, *J* = 15.1, 3.6 Hz, 1H), 2.19 – 2.14 (m, 1H), 2.10 (d, *J* = 9.3 Hz, 1H), 2.01 (s, 1H), 1.99 – 1.93 (m, 1H), 1.88 – 1.78 (m, 2H), 1.53 – 1.46 (m, 1H), 1.32 – 1.22 (m, 1H) ppm; ^{13}C{^{1}H} NMR (150 MHz, $CDCl_3$): δ 144.2, 140.2, 135.3, 118.0, 70.2, 62.6, 57.5, 46.9, 38.0, 34.5, 33.4, 22.2 ppm; HRMS (EI, 40eV) *m/z* [M]$^+$ calcd for $C_{12}H_{16}OS$ 208.0916, found 208.0919.

9.10 Reaction of epoxy allylsilane **25j**

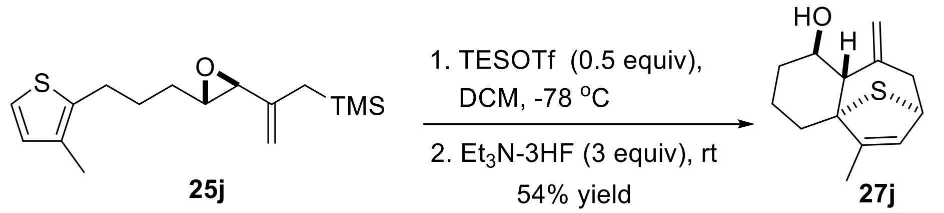

According to General Procedure B, **25j** (6 mg, 0.02 mmol, 1 equiv) in DCM (0.4 mL, 0.05 M) was treated with 0.2 M TESOTf in DCM (0.05 mL, 0.01 mmol, 0.5 equiv). The crude material obtained was purified by flash column chromatography (eluent: 10% EtOAc in hexane) to afford **27j** (2.4 mg, 54% yield).

27j: pale yellow oil; R_f(40% EtOAc in hexane) 0.34; IR (neat) 3385, 2926, 2851, 1641, 1440, 1062, 867 cm^{-1}; ^{1}H NMR (600 MHz, $CDCl_3$): δ 5.77 (s, 1H), 4.92 (dt, *J* = 2.2 Hz, 1H), 4.90 (dt, *J* = 2.4 Hz, 1H), 4.10 (ddd, *J* = 10.7, 9.2, 4.5 Hz, 1H), 3.63 – 3.60 (m, 1H), 2.78 (dd, *J* = 15.0, 2.5 Hz, 1H), 2.40 (dd, *J* = 15.0, 3.7 Hz, 1H), 2.20 – 2.11 (m, 1H), 2.09 (d, *J* = 9.3 Hz, 1H), 1.87 – 1.72 (m, 3H), 1.70 (d, *J* = 1.6 Hz, 3H), 1.57 – 1.44 (m, 1H), 1.26 (tdd, *J* = 12.9, 10.7, 4.1 Hz, 2H) ppm; ^{13}C{^{1}H} NMR (150 MHz, $CDCl_3$): δ 147.3, 144.5, 129.8, 117.6, 69.8, 63.4, 56.0, 44.3, 36.8, 33.4, 32.8, 22.4, 13.2 ppm; HRMS (EI, 40eV) *m/z* [M]$^+$ calcd for

$C_{13}H_{18}OS$ 222.1073, found 222.1072.

9.11 Reaction of epoxy allylsilane **25k**

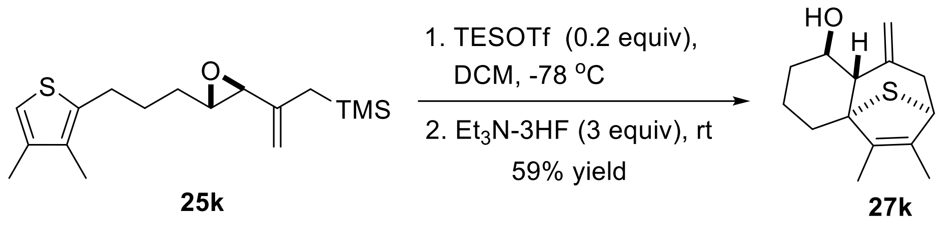

According to General Procedure B, **25k** (21.5 mg, 0.0690 mmol, 1.00 equiv) in DCM (2.6 mL, 0.05 M) was treated with 0.2 M TESOTf in DCM (0.07 mL, 0.01 mmol, 0.2 equiv). The crude material obtained was purified by flash column chromatography (eluent: 5% EtOAc in hexane) to afford **27k** (9.6 mg, 59% yield).

27k: colorless oil; R_f (5% EtOAc in hexane) 0.31; IR (neat) 3464, 2929, 2857, 1626, 1435, 1267, 965, 848 cm^{-1}; ^1H NMR (500 MHz, $CDCl_3$): δ 4.87 (t, *J* = 2.2 Hz, 1H), 4.84 (t, *J* = 2.3 Hz, 1H), 4.08 (ddd, *J* = 10.7, 9.3, 4.5 Hz, 1H), 3.41 (t, *J* = 3.0 Hz, 1H), 2.73 (d, *J* = 15.0, 2.5 Hz, 1H), 2.46 (dd, *J* = 15.0, 3.8 Hz, 1H), 2.21 – 2.11 (m, 1H), 2.02 (d, *J* = 9.3 Hz, 1H), 1.96 (d, *J* = 3.6 Hz, 1H), 1.85 – 1.74 (m, 3H), 1.66 (d, *J* = 1.4 Hz, 3H), 1.61 – 1.47 (m, 4H) ppm; $^{13}C\{^1H\}$ NMR (125 MHz, $CDCl_3$): δ 144.3, 137.8, 136.7, 117.1, 69.9, 55.2, 49.4, 35.2, 33.5, 33.4, 29.7, 22.1, 13.5, 10.6 ppm; HRMS (EI, 40eV) *m/z* $[M]^+$ calcd for $C_{14}H_{20}OS$ 236.1229, found 236.1230.

9.12 Reaction of epoxy allylsilane **25m**

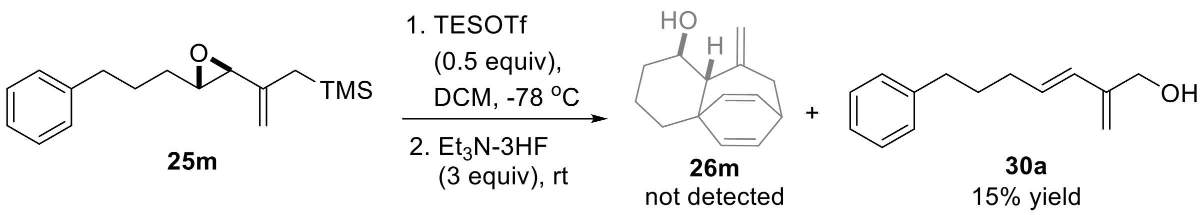

According to General Procedure B, **25m** (10 mg, 0.036 mmol, 1.0 equiv) in DCM (0.7 mL, 0.05 M) was treated with 0.2 M TESOTf in DCM (0.09 mL, 0.02 mmol, 0.5 equiv). The crude material obtained was purified by flash column chromatography (eluent: 3% EtOAc in hexane) to afford **30a** (1.1 mg, 15% yield).

(*E*)-2-Methylene-7-phenylhept-3-en-1-ol (30a): colorless oil; R_f (15% EtOAc in hexane) 0.36; IR (neat) 3422, 2957, 2926, 1644, 1454, 793, 737 cm^{-1}; ^1H NMR (400 MHz, $CDCl_3$): δ 7.29 (d, *J* = 8.2 Hz, 2H), 7.21 – 7.13 (m, 3H), 6.09 (d, *J* = 16.1 Hz, 1H), 5.84 – 5.72 (m, 1H), 5.15 (s, 1H), 5.03 (s, 1H), 4.31 (d, *J* = 5.5 Hz, 2H), 2.63 (t, 2H), 2.19 – 2.11 (m, 2H), 1.75 (p, *J* = 7.6 Hz, 2H) ppm; $^{13}C\{^1H\}$ NMR (150 MHz, $CDCl_3$): δ 145.3, 142.4, 130.8, 129.9, 128.6, 128.4, 125.9, 113.5, 63.5, 35.6, 32.7, 31.0 ppm; HRMS (EI, 40eV) *m/z* $[M]^+$ calcd for $C_{14}H_{18}O$ 202.1352, found 202.1347.

9.13 Reaction of epoxy allylsilane **25n**

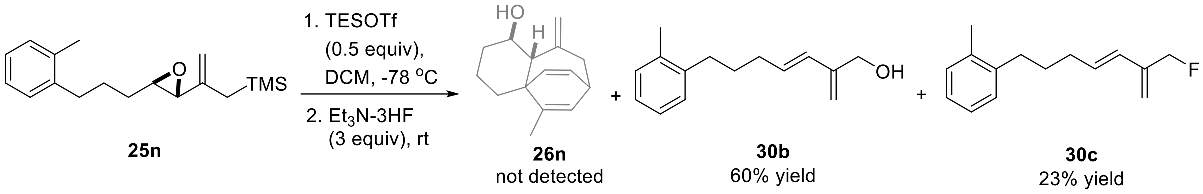

According to General Procedure B, **35n** (10 mg, 0.035 mmol, 1.0 equiv) in DCM (0.7 mL, 0.05 M) was treated with 0.2 M TESOTf in DCM (0.09 mL, 0.02 mmol, 0.5 equiv). The crude material obtained was purified by flash column chromatography (eluent: 5% EtOAc in hexane) to afford **30b** (4.5 mg, 60% yield) and **30c** (1.7 mg, 23% yield).

(*E*)-2-Methylene-7-(*o*-tolyl)hept-3-en-1-ol (30b): colorless oil; R_f (15% EtOAc in hexane) 0.33; IR (neat) 3401, 2925, 1583, 1461, 744 cm^{-1}; ^{1}H NMR (600 MHz, $CDCl_3$): δ 7.18 – 7.10 (m, 4H), 6.13 (d, *J* = 16.0 Hz, 1H), 5.86 – 5.78 (m, 1H), 5.17 (s, 1H), 5.06 (s, 1H), 4.34 (d, *J* = 4.4 Hz, 2H), 2.66 – 2.61 (m, 2H), 2.32 (s, 3H), 2.25 – 2.18 (m, 2H), 1.76 – 1.68 (m, 2H) ppm; ^{13}C{^{1}H} NMR (150 MHz, $CDCl_3$): δ 145.3, 140.6, 136.0, 130.9, 130.3, 129.9, 129.0, 126.0, 126.0, 113.6, 63.5, 33.0, 32.9, 29.8, 19.4 ppm; HRMS (EI, 40eV) *m/z* $[M]^+$ calcd for $C_{15}H_{20}O$ 216.1509, found 216.1512.

(*E*)-1-(6-(Fluoromethyl)hepta-4,6-dien-1-yl)-2-methylbenzene (30c): colorless oil; R_f (15% EtOAc in hexane) 0.81; IR (neat) 2926, 2855, 1460, 997, 741 cm^{-1}; ^{1}H NMR (600 MHz, $CDCl_3$): δ 7.15 – 7.08 (m, 4H), 6.10 (d, *J* = 16.1 Hz, 1H), 5.82 – 5.72 (m, 1H), 5.18 (s, 1H), 5.13 (s, 1H), 5.05 (s, 1H), 4.98 (s, 1H), 2.67 – 2.54 (m, 2H), 2.30 (s, 3H), 2.27 – 2.12 (m, 2H), 1.73 – 1.67 (m, 2H) ppm; ^{13}C{^{1}H} NMR (150 MHz, $CDCl_3$): δ 141.1 (d, $J_{C\text{-}C\text{-}F}$= 14.5 Hz), 140.4, 135.8, 131.6, 130.1, 128.9, 128.8, 125.9, 125.9, 115.8 (d, $J_{C\text{-}C\text{-}C\text{-}F}$= 11.2 Hz), 83.1 (d, $J_{C\text{-}F}$= 167.2 Hz), 32.9, 32.7, 29.6, 19.3 ppm; ^{19}F NMR (564 MHz, $CDCl_3$): δ -215.0 ppm (t, *J* = 47.4 Hz); HRMS (EI, 40eV) *m/z* $[M]^+$ calcd for $C_{15}H_{19}F$ 218.1465, found 218.1462.

9.14 Reaction of epoxy allylsilane **25o**

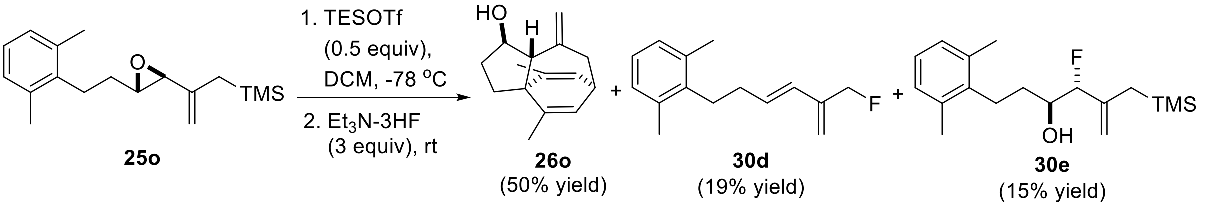

According to General Procedure B, **25o** (7.5 mg, 0.026 mmol, 1.0 equiv) in DCM (0.5 mL, 0.05 M) was treated with 0.2 M TESOTf in DCM (0.06 mL, 0.01 mmol, 0.5 equiv). The crude material obtained was purified by flash column chromatography (eluent: 5% EtOAc in hexane) to afford **26o** (2.8 mg, 50% yield), **30d** (1.1 mg, 19% yield), and **30e** (1.2 mg, 15% yield).

26o: colorless oil; R_f (15% EtOAc in hexane) 0.37; IR (neat) 3352, 2957, 2922, 2851, 1632, 833 cm^{-1}; ^{1}H NMR (600 MHz, $CDCl_3$): δ 6.01 – 5.81 (m, 2H), 4.80 (d, *J* = 1.7 Hz, 1H), 4.74 (d, *J* = 1.8 Hz, 1H), 4.12 – 4.00 (m, 1H), 2.85 (q, *J* = 6.6, 1.1 Hz, 1H), 2.27 – 2.09 (m, 4H), 2.05 (d, *J* = 13.7, 1.6 Hz, 1H), 2.02 – 1.91 (m, 1H), 1.86 (d, *J* = 1.5 Hz, 3H), 1.78 (d, *J* = 1.5 Hz, 3H), 1.76 – 1.64 (m, 1H) ppm; ^{13}C{^{1}H} NMR (150 MHz, $CDCl_3$): δ 148.7, 147.2, 141.9, 130.0, 127.6, 110.8, 73.3, 61.2, 53.7, 36.0, 34.5, 34.2, 24.5, 21.2, 18.4 ppm; HRMS (EI, 40eV) *m/z* $[M]^+$ calcd for $C_{15}H_{20}O$ 216.1509, found 216.1510.

(*E*)-2-(5-(Fluoromethyl)hexa-3,5-dien-1-yl)-1,3-dimethylbenzene (30d): colorless oil; R_f

(15% EtOAc in hexane) 0.80; IR (neat) 2953, 2924, 1651, 1468, 768 cm^{-1}; ^{1}H NMR (400 MHz, $CDCl_3$): δ 7.01 (s, 3H), 6.15 (d, J = 16.1 Hz, 1H), 5.92 – 5.78 (m, 1H), 5.21 (s, 1H), 5.16 (s, 1H), 5.09 (s, 1H), 4.98 (s, 1H), 2.80 – 2.69 (m, 2H), 2.33 (s, 6H), 2.31 – 2.22 (m, 2H) ppm; $^{13}C\{^{1}H\}$ NMR (150 MHz, $CDCl_3$): δ 141.2 (d, $J_{C\text{-}C\text{-}F}$= 14.8 Hz), 138.5, 136.2, 131.5, 128.9, 128.3, 126.0, 116.4 (d, $J_{C\text{-}C\text{-}C\text{-}F}$= 10.8 Hz), 83.3 (d, $J_{C\text{-}F}$= 164.4 Hz), 32.6, 29.5, 20.0 ppm; ^{19}F NMR (376 MHz, $CDCl_3$): δ -217.0 ppm (t, J = 46.7 Hz); HRMS (EI, 40eV) *m/z* $[M]^+$ calcd for $C_{15}H_{19}F$ 218.1465, found 218.1463.

(*3S*,4R)-1-(2,6-Dimethylphenyl)-4-fluoro-5-((trimethylsilyl)methyl)hex-5-en-3-ol (30e):** colorless oil; R_f (15% EtOAc in hexane) 0.52; IR (neat) 3456, 2953, 2922, 1634, 1248, 851 cm^{-1}; ^{1}H NMR (600 MHz, $CDCl_3$): δ 7.03 (s, 3H), 5.03 (s, 1H), 4.91 (s, 1H), 4.62 (dd, J = 48.3, 5.6 Hz, 1H), 3.89 – 3.80 (m, 1H), 2.99 – 2.90 (m, 1H), 2.74 – 2.67 (m, 1H), 2.36 (s, 6H), 2.18 (s, 1H), 1.70 – 1.59 (m, 2H), 1.47 – 1.39 (m, 1H), 0.07 (s, 9H) ppm; $^{13}C\{^{1}H\}$ NMR (150 MHz, $CDCl_3$): δ 142.6 (d, $J_{C\text{-}C\text{-}F}$= 17.3 Hz), 138.8, 136.3, 128.3, 125.9, 112.4 (d, $J_{C\text{-}C\text{-}C\text{-}F}$= 10.4 Hz), 98.3 (d, $J_{C\text{-}F}$= 173.8 Hz), 72.1 (d, $J_{C\text{-}C\text{-}F}$= 21.4 Hz), 31.5 (d, $J_{C\text{-}C\text{-}C\text{-}F}$= 4.5 Hz), 25.9, 22.6 (d, $J_{C\text{-}C\text{-}C\text{-}F}$= 2.5 Hz), 19.9, -1.2 ppm; ^{19}F NMR (470 MHz, $CDCl_3$): δ -187.7 ppm (dd, J = 48.4, 17.8 Hz); HRMS (EI, 40eV) *m/z* $[M]^+$ calcd for $C_{18}H_{29}OFSi$ 308.1966, found 308.1961.

9.15 Reaction of epoxy allylsilane **25p**

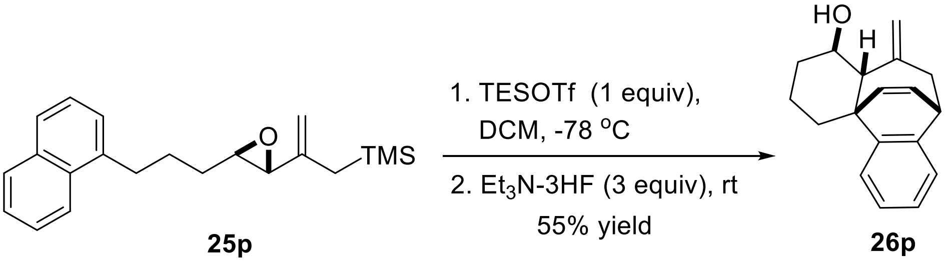

According to General Procedure B, **25p** (15 mg, 0.047 mmol, 1.0 equiv) in DCM (0.93 mL, 0.05 M) was treated with 0.2 M TESOTf in DCM (0.23 mL, 0.047 mmol, 1 equiv). The crude material obtained was purified by flash column chromatography (eluent: 15% EtOAc in hexane) to afford the **26p** (6.6 mg, 55% yield).

26p: white solid; R_f (15% EtOAc in hexane) 0.30; IR (neat) 3462, 2934, 2876, 1476, 741 cm^{-1}; ^{1}H NMR (600 MHz, $CDCl_3$): δ 7.47 (dd, J = 7.0, 1.7 Hz, 1H), 7.18 – 7.08 (m, 3H), 6.39 (dd, J = 8.5, 6.6 Hz, 1H), 6.08 (d, J = 8.5, 1.1 Hz, 1H), 4.77 (s, 1H), 4.73 (s, 1H), 3.58 – 3.50 (m, 1H), 3.38 (td, J = 10.3, 4.1, 1.4 Hz, 1H), 2.59 (dd, J = 13.9, 4.4, 1.1 Hz, 1H), 2.48 – 2.40 (m, 2H), 2.30 (d, J = 10.1 Hz, 1H), 2.23 – 2.13 (m, 2H), 2.03 – 1.95 (m, 1H), 1.89 – 1.79 (m, 2H), 1.51 – 1.42 (m, 1H) ppm; $^{13}C\{^{1}H\}$ NMR (150 MHz, $CDCl_3$): δ 146.7, 145.6, 143.3, 142.8, 133.7, 125.8, 125.3, 125.3, 125.0, 116.9, 67.4, 57.9, 45.1, 40.7, 39.6, 36.2, 34.5, 22.2 ppm; HRMS (EI, 40eV) *m/z* $[M]^+$ calcd for $C_{18}H_{20}O$ 252.1509, found 252.1508; m.p. 68.0-71.2 °C.

9.16 Reaction of epoxy allylsilane **25q**

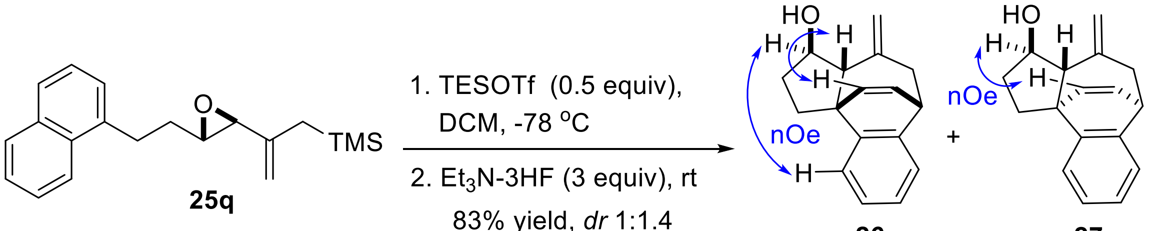

According to General Procedure B, treatment of **25q** (10.9 mg, 0.0350 mmol, 1.00 equiv) in DCM (0.7 mL, 0.05 M) with 0.2 M TESOTf in DCM (0.09 mL, 0.02 mmol, 0.5 equiv) afforded a mixture of **26q + 27q** in a ratio of 1:1.4 as determined by ^{1}H NMR spectroscopy, in the form of pale yellow oil. The crude material obtained was purified by flash column chromatography (eluent: 5% EtOAc in hexane), which provided **26q** (3.3 mg, 40 % yield) and **27q** (3.6 mg, 43% yield).

26q: white solid; R_f (15% EtOAc in hexane) 0.20; IR (neat) 3364, 2928, 1634, 970, 893 cm^{-1}; ^{1}H NMR (600 MHz, $CDCl_3$): δ 7.20 – 7.08 (m, 4H), 6.51 (dd, *J* = 8.4, 6.7 Hz, 1H), 6.29 (dd, *J* = 8.4, 1.2 Hz, 1H), 4.62 (s, 1H), 4.49 (s, 1H), 4.05 (q, *J* = 8.2 Hz, 1H), 3.57 (t, *J* = 6.6 Hz, 1H), 2.58 – 2.51 (m, 1H), 2.50 – 2.42 (m, 2H), 2.40 (d, *J* = 5.8 Hz, 2H), 2.19 (ddd, *J* = 13.9, 11.9, 6.1 Hz, 1H), 1.96 – 1.87 (m, 1H) ppm; ^{13}C{^{1}H} NMR (150 MHz, $CDCl_3$): δ 146.9, 143.0, 142.2, 141.8, 135.9, 125.7, 125.5, 125.5, 122.6, 112.9, 74.7, 59.6, 50.8, 40.6, 38.5, 33.4, 31.2 ppm; HRMS (EI, 40eV) *m/z* [M]$^+$ calcd for $C_{17}H_{18}O$ 238.1352, found 238.1357; m.p. 48.2-50.9 °C.

27q: pale yellow oil; R_f (15% EtOAc in hexane) 0.20; IR (neat) 3383, 2955, 2357, 745 cm^{-1}; ^{1}H NMR (600 MHz, $CDCl_3$): δ 7.33 (d, *J* = 7.8 Hz, 1H), 7.22 – 7.15 (m, 2H), 7.14 – 7.10 (m, 1H), 6.32 (dd, *J* = 8.5, 6.6 Hz, 1H), 5.97 (d, *J* = 8.3 Hz, 1H), 4.88 (s, 1H), 4.81 (s, 1H), 4.31 – 4.23 (m, 1H), 3.60 (t, *J* = 6.6 Hz, 1H), 2.86 – 2.76 (m, 1H), 2.54 (dd, *J* = 14.2, 6.8 Hz, 1H), 2.31 – 2.23 (m, 1H), 2.23 – 2.14 (m, 2H), 1.91 – 1.82 (m, 2H) ppm; ^{13}C{^{1}H} NMR (150 MHz, $CDCl_3$): δ 147.4, 147.0, 144.6, 139.3, 131.9, 125.9, 125.7, 125.5, 120.1, 111.6, 74.9, 63.0, 50.5, 41.4, 38.5, 34.2, 30.7 ppm; HRMS (EI, 40eV) *m/z* [M]$^+$ calcd for $C_{17}H_{18}O$ 238.1352, found 238.1351.

9.17 Reaction of epoxy allylsilane **25r**

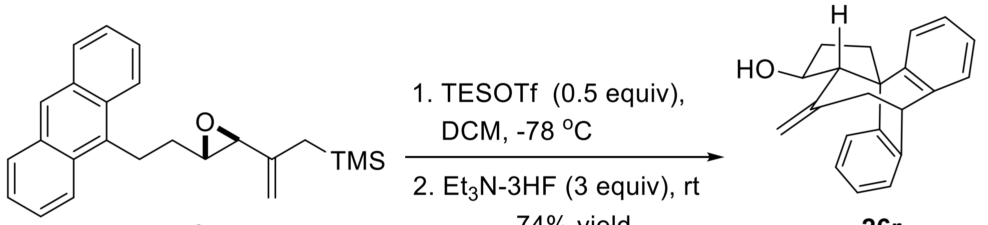

25r **26r**

According to General Procedure B, **25r** (8.7 mg, 0.024 mmol, 1.0 equiv) in DCM (0.5 mL, 0.05 M) was treated with 0.2 M TESOTf in DCM (0.06 mL, 0.01 mmol, 0.5 equiv). The crude material obtained was purified by flash column chromatography (eluent: 15% EtOAc in hexane) to afford **26r** (5.1 mg, 74% yield).

26r: white solid; R_f (30% EtOAc in hexane) 0.59; IR (neat) 3350, 2953, 2930, 2887, 770 cm^{-1}; ^{1}H NMR (600 MHz, $CDCl_3$): δ 7.46 (d, *J* = 7.6 Hz, 1H), 7.31 (dd, *J* = 7.2, 1.7 Hz, 1H), 7.28 (dd, *J* = 7.1, 1.6 Hz, 1H), 7.24 (dd, *J* = 6.7, 2.0 Hz, 1H), 7.20 – 7.12 (m, 4H), 4.63 (s, 1H), 4.58 (s, 1H), 4.24 (q, *J* = 6.2 Hz, 1H), 4.10 (d, *J* = 7.3 Hz, 1H), 3.00 – 2.94 (m, 1H), 2.73 (dd, *J* = 13.8, 7.4 Hz, 1H), 2.59 – 2.46 (m, 4H), 2.05 – 1.97 (m, 1H) ppm; ^{13}C{^{1}H} NMR (150 MHz, $CDCl_3$): δ 146.3, 145.6, 144.6, 142.1, 141.7, 126.2, 126.2, 126.2, 126.2, 126.1, 125.9, 123.3, 121.3, 112.1, 74.7, 63.8, 53.4, 46.8, 41.7, 33.5, 25.8 ppm; HRMS (EI, 40eV) *m/z* [M]$^+$ calcd for $C_{21}H_{20}O$ 288.1509, found 288.1507; m.p. 48.1-51.8 °C.

10. Proposed mechanism accounting for the formation of 30d and 30e

Using epoxy allylsilane **25o** as an example, **Scheme S5** shows the proposed mechanism to account for the formation of cycloadduct **26o** and side products **30d-e**.

The overall mechanistic picture is one of the competitive nucleophilic attack of the activated epoxide **25o-I**. Pathway a is the route culminating in (4+3) cycloaddition. Fluorohydrin **30e** is generated through the ring-opening of activated epoxide **25o-I** by the fluoride nucleophile (pathway b). Lastly, the S_N2' attack of the fluoride to the terminal alkene of **25o-I** via pathway c forms intermediate **25o-IV**, followed by another attack of the fluoride anion to the trimethylsilyl group to afford diene **30d**.

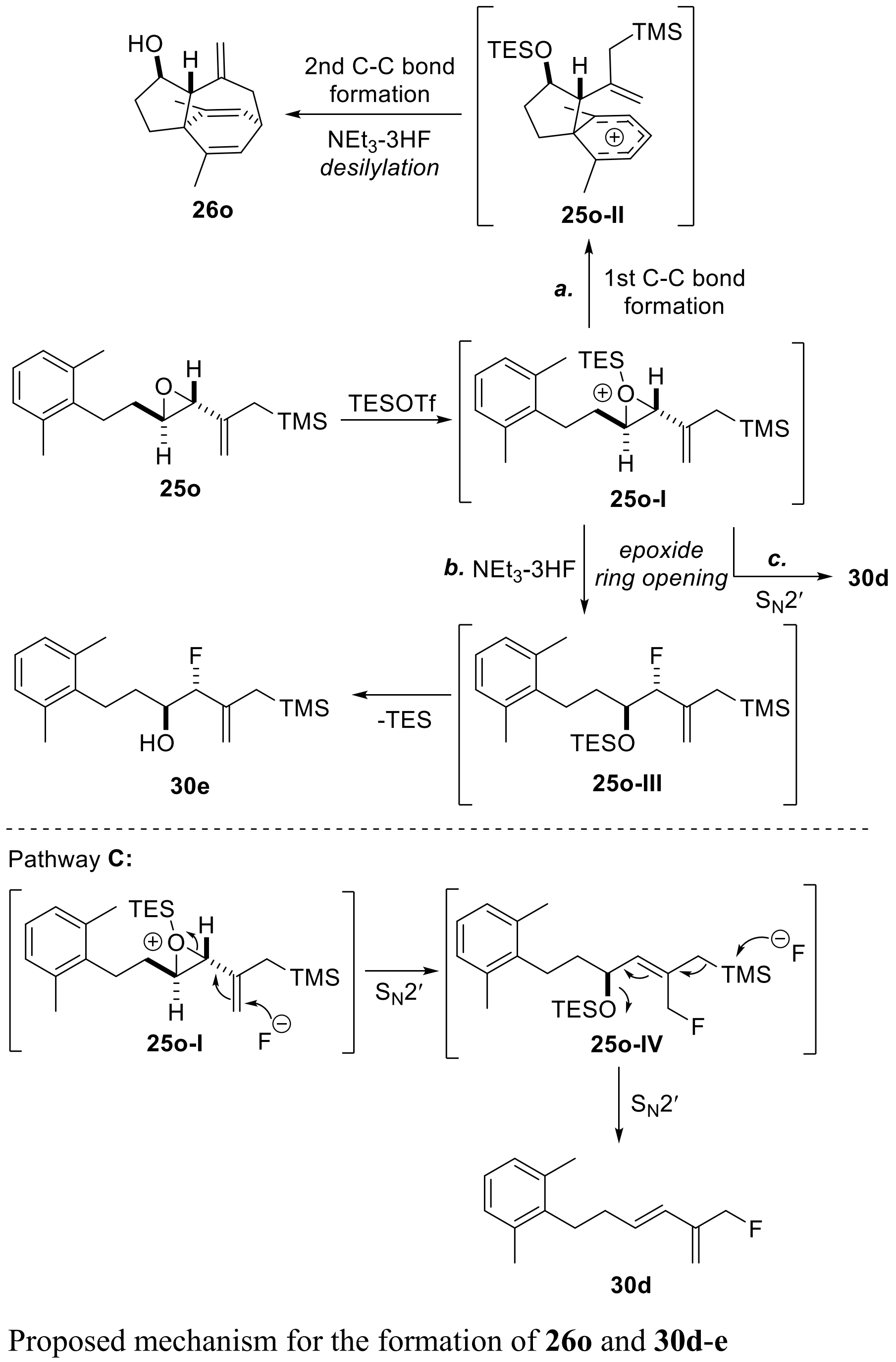

Scheme S5. Proposed mechanism for the formation of **26o** and **30d-e**

11. Computed reaction energy profiles for intramolecular (4+3) cycloadditions

11.1 Intramolecular (4+3) Cycloaddition of Epoxy Allylsilane **C**

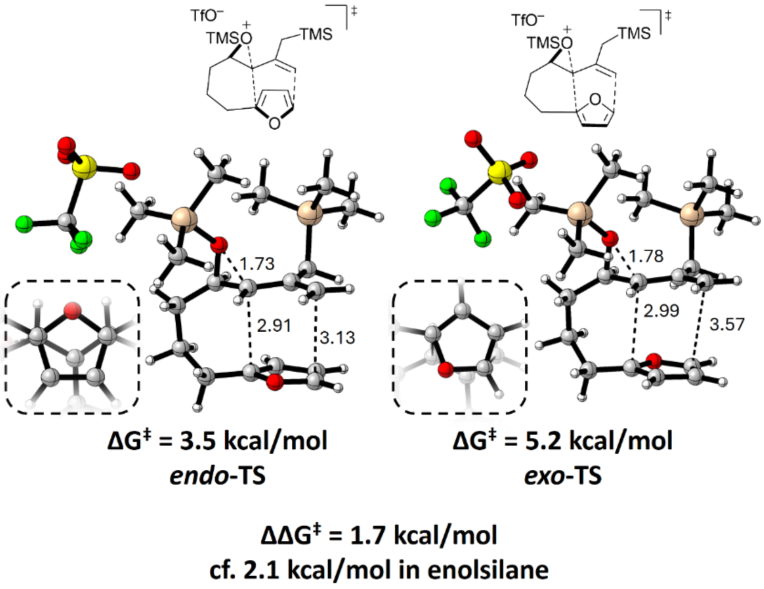

Figure S3. Geometries of *endo* and *exo* transition states for the intramolecular (4+3) cycloaddition of allylsilane **C**. The insets at the bottom left of each structure show a bottom-up view of the two reacting moieties and show that the two reactive moieties in the *exo*-TS do not align as well as those in the *endo*-TS, resulting in a higher energy for the *exo* TS.

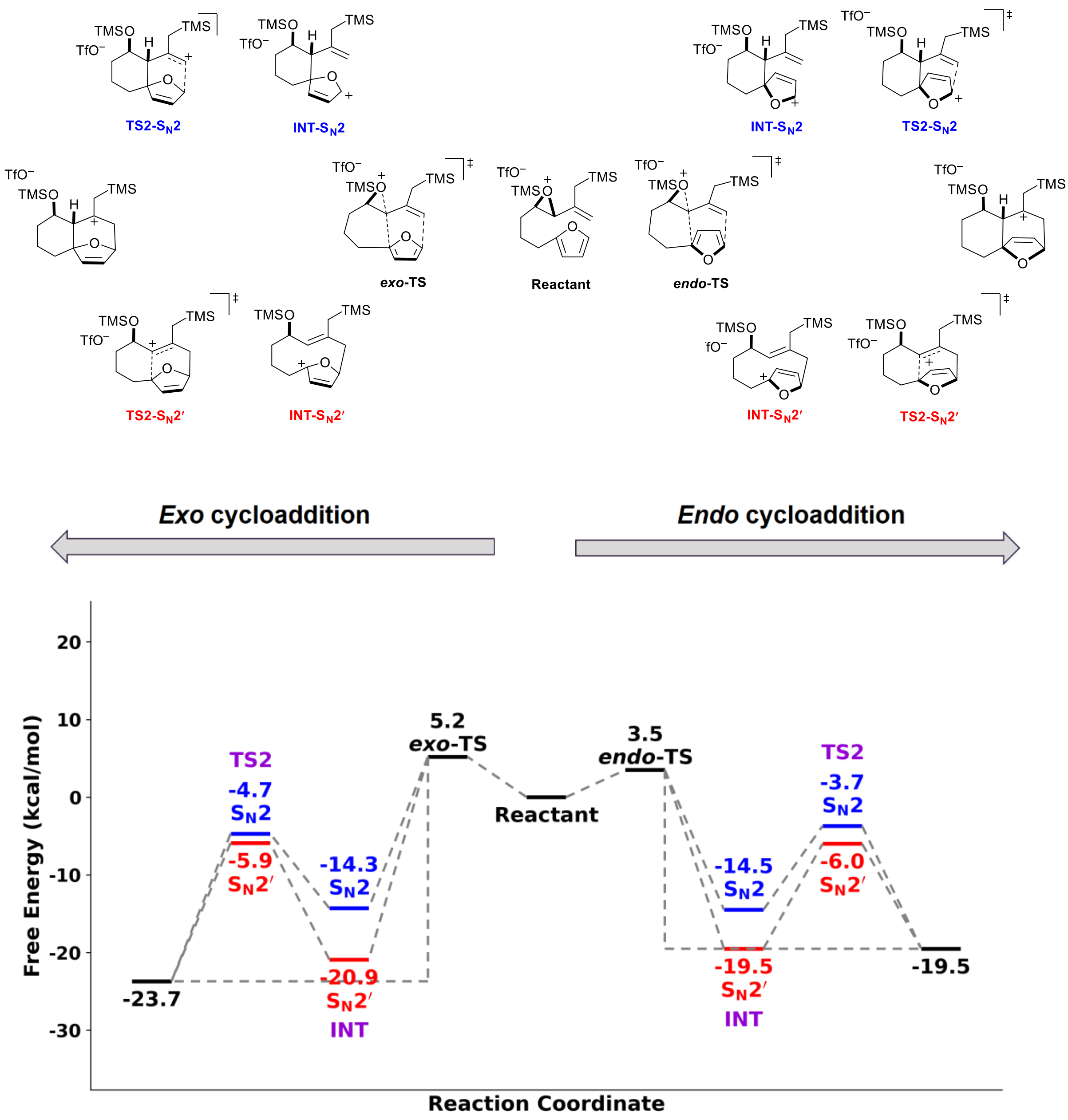

Scheme S6. Free Energy Profile for the intramolecular (4+3) cycloaddition of furan-tethered allylsilane **C** (Analogue of **25a**)

11.2 Intramolecular (4+3) Cycloaddition of Epoxy Enolsilane **D**

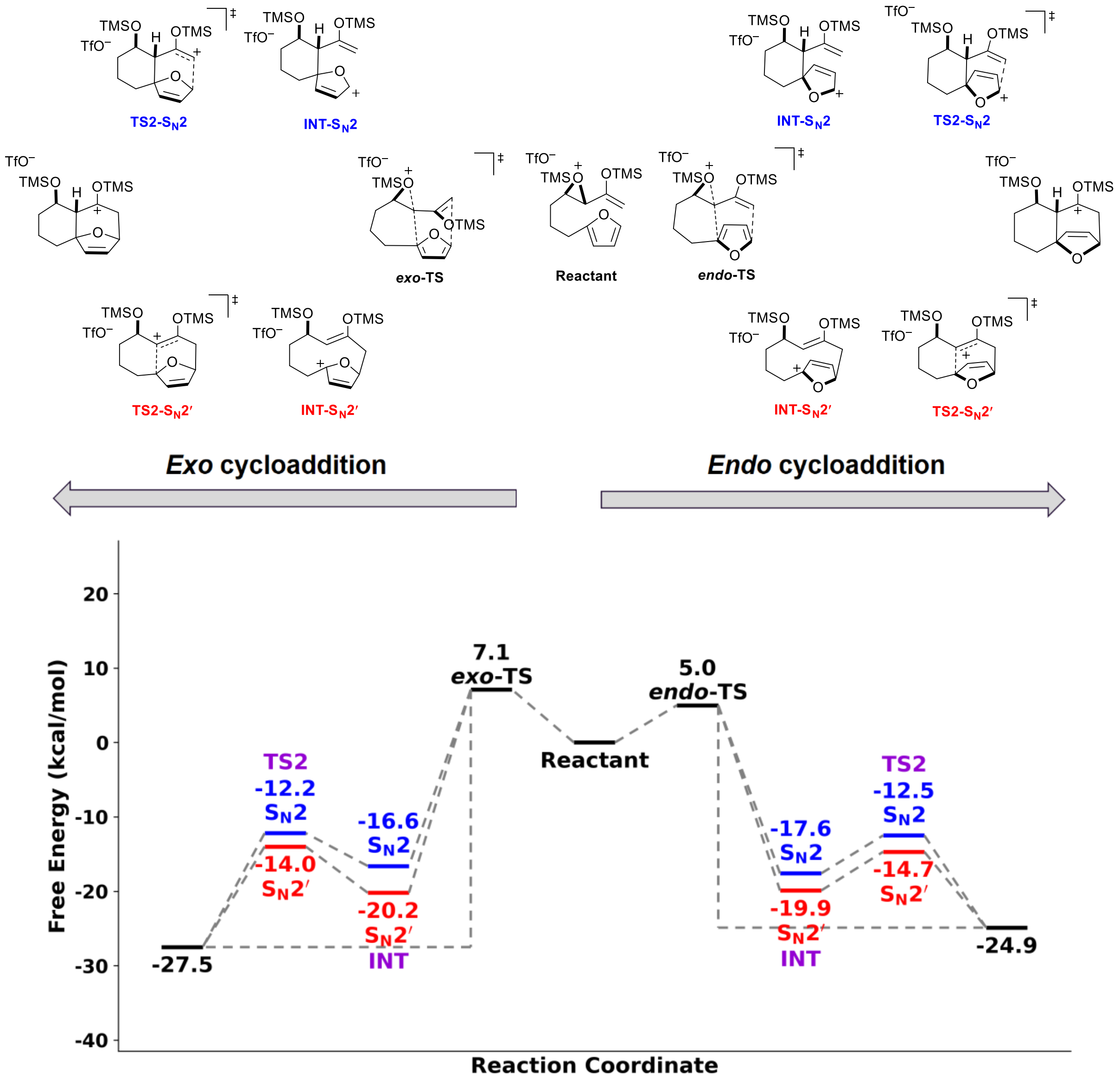

Scheme S7. Free Energy Profile for the intramolecular (4+3) cycloaddition of furan- tethered enolsilane **D** (Analogue of Enolsilane **12)**

For epoxy enolsilanes, computational studies were previously performed to account for the observed diastereoselectivity of intramolecular (4+3) cycloaddition.[48] Taking the furan-tethered epoxy enolsilane shown in **Scheme S7** as an example, the TS leading to the *endo* cycloadduct was previously calculated to be 2.6 kcal/mol energetically less demanding compared to the TS leading to the *exo* cycloadduct. Here, we recalculated the two TSs with the same level of theory that we used elsewhere in our current study.

The value of $\Delta\Delta G^{\ddagger}$ was 2.1 kcal/mol. In the *exo* transition state, the reactive sites for cycloaddition are not as well aligned as in the *endo* TS. Although the enolsilane fragment can rotate to improve alignment with the furan, such a conformational adjustment would result in a steric clash between the methylene group and the epoxy ring, as well as a twist of the highlighted dihedral bond **(Figure S4)**, making it difficult to achieve.

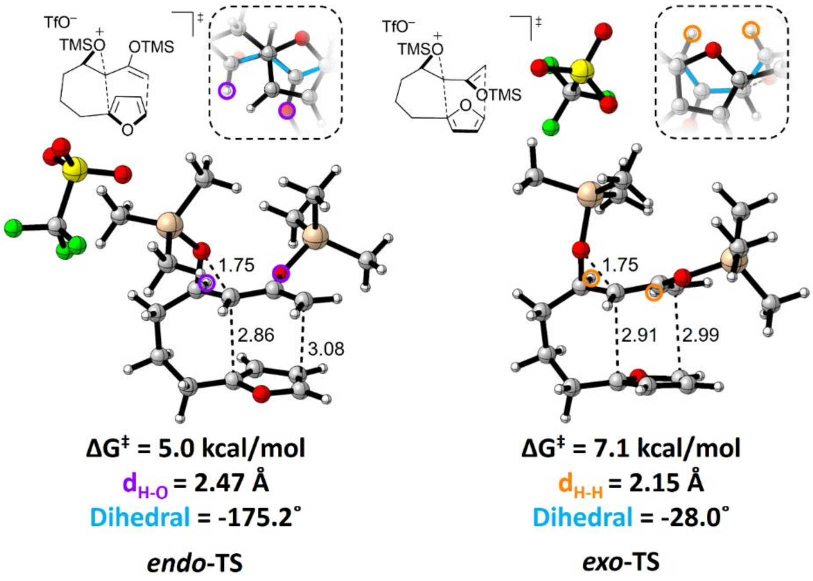

Figure S4. Geometries of *endo* and *exo* transition states for the intramolecular (4+3) cycloaddition of enolsilane **D**. The insets at the top right of each structure show a bottom-up view of the two reacting moieties. In the *exo*-TS, alignment of the reactive moieties is achieved by flipping of the enosilane, but this leads to a steric clash between the two highlighted hydrogens and an unfavorable torsion of the highlighted dihedral angle.

For the corresponding furan-tethered epoxy allylsilane, our computational results **(Scheme S6)** suggest a more complex explanation for the selectivity. The initial attack by the diene in the *endo* mode is favoured by 1.7 kcal/mol relative to the *exo* mode. On this basis, the reaction would be expected to display strong *endo* selectivity, a result that is contrary to the small experimentally observed *dr* (1.7:1). However, although the energies of the TSs for the initial attack by the diene make an important contribution the overall stereoselectivity, post-transition state dynamical effects are also expected to play a role. Some of the reaction flux may occur in a concerted manner similar to that shown in **Figure S3** for the reaction of **A** with furan. For the stepwise pathways, the ring closure steps have relatively high barriers and a thermodynamically competitive S_N2' intermediate, which means that any intermediates that do form may have longer lifetimes, increasing their potential for side reactions. Overall, the relative contributions of the various possible reaction pathways cannot be predicted from the energies of the TSs and intermediates.

12. Asymmetric (4+3) cycloaddition of epoxy allylsilane (+)-25a

12.1 Preparation of **(*R*)-2-Chloro-5-(furan-2-yl)pentanal (+)-23a**

To a solution of aldehyde **22a** (0.15 g, 1.0 mmol, 1.0 equiv) in anhydrous DCM (2 mL, 0.5M) was added (*L*)-prolinamide (45 mg, 0.40 mmol, 0.4 equiv) and NCS (0.17 g, 1.3 mmol, 1.3 equiv) at 0 °C. The reaction mixture was stirred at room temperature and monitored by TLC until no starting material remained. The reaction mixture was cooled to –78 °C and pentane was added. The mixture was filtered through a fritted funnel and the volatiles were removed *in vacuo*. The residue was purified by chromatography through a short column (eluent: 15% EtOAc in hexane) to afford **(+)-23a** (97 mg, 0.52 mmol, 52% yield, 78% *ee*). The absolute stereochemistry of **(+)-23a** as the major enantiomer is assigned based on the analogous application of (*L*)-prolinamide in this reaction in the literature.[49]

The enantiomeric excess (ee) of **(+)-23a** was analyzed by HPLC (Daicel Chiralcel OJ-3 column), hexane/2-propanol = 90/10, flow = 1.0 mL/ min, UV= 210nm, t_{major} = 8.25 min, t_{minor} = 8.62 min.

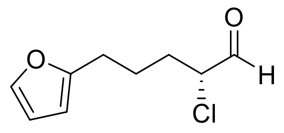

(+)-23a, 78 *%ee*

(+)-23a: pale-yellow oil; R_f(5% EtOAc in hexane) 0.18; $[\alpha]_D^{23}$ = +46.9 (*c* 0.0018, $CHCl_3$); IR (neat) 2926, 1723, 1569, 1453, 1386, 1217, 1019, 957, 778 cm^{-1}; ^{1}H NMR (400 MHz, $CDCl_3$): δ 9.49 (d, *J* = 2.2 Hz, 1H), 7.31 (dd, *J* = 1.9, 0.9 Hz, 1H), 6.28 (dd, *J* = 3.2, 1.9 Hz, 1H), 6.06 – 5.98 (m, 1H), 4.18 (ddd, *J* = 7.6, 5.1, 2.2 Hz, 1H), 2.69 (t, *J* = 6.9 Hz, 2H), 2.11 – 1.98 (m, 1H), 1.99 – 1.71 (m, 3H) ppm; ^{13}C{^{1}H} NMR (100 MHz, $CDCl_3$): δ 195.2, 154.9, 141.3, 110.3, 105.5, 63.8, 31.4, 27.3, 24.3 ppm; ESI-HRMS *m/z* calcd for $C_9H_{12}ClO_2$ [M+H]$^+$: 187.0520, found 187.0519.

12.2 Preparation of **2-(*2S,3S*)-3-3-(Furan-2-yl-propyl-oxiran-2-yl-allyl)trimethylsilane (+)-25a**

According to the procedure for the synthesis of **25a**, epoxy allylsilane **(+)-25a** was obtained from the treatment of α-chloroaldehyde **(+)-23a** (70 mg, 0.38 mmol. 1.0 equiv) in anhydrous THF (1.9 mL, 0.2 M) with **37** (95 mg, 0.49 mmol, 1.3 equiv) in Et_2O (0.8 mL, 0.6 M) and *tert*-butyllithium (1.18M in pentanes, 0.86 mL, 1.0 mmol, 2.7 equiv). The crude material obtained was purified by flash column chromatography (eluent: 0.3% Et_2O / hexane) to give **(+)-25a** (8 mg, 0.03 mmol, 8% yield).

The enantiomeric excess of **(+)-25a** could not be determined as this epoxy allylsilane failed to achieve separation in all the chiral columns tried. As the epoxide formation step from chloroaldehyde **(+)-33a** is stereospecific, the corresponding chiral information and *ee* are assumed to be retained in epoxy allylsilane **(+)-25a**. This was verified to be the case in the analysis of the final cycloaddition products (+)-**26a** and (–)-**27a**.

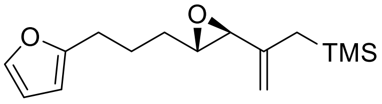

(+)-25a

(+)-25a: R_f(5% EtOAc in hexane) 0.56; $[\alpha]_D^{24}$ = +3.56 (*c* 0.014, $CHCl_3$); IR (neat) 2925, 1602, 1507, 1248, 1147, 1006, 884, 848, 727, 694, 626, 599 cm^{-1}; ^{1}H NMR (500 MHz, $CDCl_3$): δ 7.30 (s, 1H), 6.28 (dd, *J* = 3.1, 1.9 Hz, 1H), 5.99 (d, *J* = 3.1 Hz, 1H), 4.95 (s, 1H), 4.71 (s, 1H), 3.01 (d, *J* = 2.2 Hz, 1H), 2.77 (ddd, *J* = 6.7, 4.7, 2.1 Hz, 1H), 2.69 (t, *J* = 7.5 Hz, 2H), 1.88 – 1.74 (m, 2H), 1.71 – 1.63 (m, 1H), 1.62 – 1.56 (m, 1H), 1.49 – 1.40 (m, 2H), 0.03 (s, 9H) ppm; ^{13}C{^{1}H} NMR (125 MHz, $CDCl_3$): δ 155.8, 143.5, 141.0, 110.2, 109.4, 105.2, 60.6, 59.5, 31.6, 27.8, 24.7,

21.8, -1.3 ppm; ESI-HRMS *m/z* calcd for $C_{15}H_{25}O_2Si$ $[M+H]^+$: 265.1618, found 265.1620.

12.3 Reaction of epoxy allylsilane **(+)-25a**

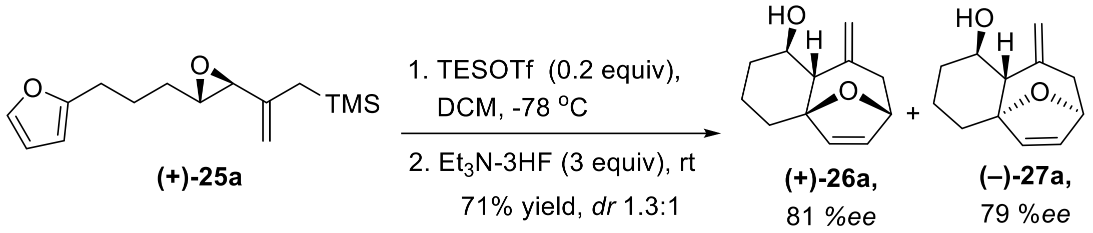

According to General Procedure B, treatment of **(+)-25a** (8 mg, 0.03 mmol, 1 equiv) in DCM (0.6 mL, 0.05 M) with 0.2 M TESOTf in DCM (0.03 mL, 0.006 mmol, 0.2 equiv) afforded a mixture of (+)-**26a** and (–)-**27a** in a ratio of 1.3:1 as determined by ^{1}H NMR spectroscopy, in the form of pale yellow oil. The crude material obtained was purified by flash column chromatography (eluent: 20% EtOAc in hexane), which provided (+)-**26a** (2.2 mg, 38% yield, 81 *%ee*) and (–)-**27a** (1.9 mg, 33% yield, 79 *%ee*).

The enantiomeric excess (ee) of (+)-**26a** was measured by HPLC (Daicel Chiralcel OJ-3 column), hexane/2-propanol = 95/5, flow = 1.0 mL/ min, UV=210nm, t_{major} = 9.03 min, t_{minor} = 13.5 min.

The enantiomeric excess (ee) of (–)-**27a** was measured by HPLC (Daicel Chiralcel OJ-3 column), hexane/2-propanol = 95/5, flow = 1.0 mL/ min, UV=210nm, t_{minor} = 6.52 min, t_{major} = 7.68 min.

(+)-26a: white solid; R_f(30% EtOAc in hexane) 0.33; $[\alpha]_D^{21}$ = +76.6 (*c* 0.0029, $CHCl_3$); IR (neat) 3393, 2922, 2851, 1643, 1450, 1351, 1225, 1160, 1064, 1030, 1000, 969, 952, 904, 883, 866, 828, 803, 739, 673, 643, 532, 452, 407 cm^{-1}; ^{1}H NMR (600 MHz, $CDCl_3$): δ 6.11 (d, *J* = 6.0, 1.8, 0.9 Hz, 1H), 5.99 (d, *J* = 6.0 Hz, 1H), 5.16 (d, *J* = 1.7 Hz, 1H), 4.85 (d, *J* = 1.7 Hz, 1H), 4.78 (dt, *J* = 3.6, 1.7 Hz, 1H), 3.63 – 3.56 (m, 1H), 2.60 (d, *J* = 13.7, 3.4, 1.6 Hz, 1H), 2.24 (d, *J* = 10.2, 1.7 Hz, 1H), 2.14 – 2.05 (m, 1H), 2.04 (dd, *J* = 13.7, 1.6 Hz, 1H), 1.88 – 1.80 (m, 2H), 1.69 – 1.63 (m, 1H), 1.48 – 1.37 (m, 3H) ppm; $^{13}C\{^1H\}$ NMR (150 MHz, $CDCl_3$): δ 143.4, 132.4, 131.3, 112.1, 87.3, 79.3, 69.3, 57.1, 39.0, 35.3, 33.2, 20.5 ppm; EI-MS (20 eV) *m/z* 192 (M^+, 13), 145 (100), 91 (44); HRMS (EI, 40eV) *m/z* $[M]^+$ calcd for $C_{12}H_{16}O_2$ 192.1145, found 192.1141; m.p. 60.2-63.7 °C.

(–)-27a: colorless oil; R_f(30% EtOAc in hexane) 0.38; $[\alpha]_D^{21}$ = –5.71 (*c* 0.0014, $CHCl_3$); IR (neat) 3401, 2926, 2853, 1644, 1450, 1161, 1072, 1023, 986, 944, 906, 861, 817, 722, 649 cm^{-1}; ^{1}H NMR (600 MHz, $CDCl_3$): δ 6.09 (dd, *J* = 5.9, 1.8 Hz, 1H), 5.93 (d, *J* = 5.9 Hz, 1H), 4.91 (t, *J* = 2.2 Hz, 1H), 4.89 (t, *J* = 2.3 Hz, 1H), 4.86 (dt, *J* = 3.8, 1.6 Hz, 1H), 3.84 – 3.77 (m, 1H), 2.70 – 2.64 (m, 1H), 2.15 – 2.06 (m, 1H), 2.04 (dd, *J* = 14.6, 1.4 Hz, 1H), 1.89 (d, *J* = 1.8 Hz, 1H), 1.87 – 1.81 (m, 2H), 1.75 – 1.68 (m, 3H), 1.65 – 1.60 (m, 1H) ppm; $^{13}C\{^1H\}$ NMR (150 MHz, $CDCl_3$): δ 142.7, 135.9, 132.3, 116.0, 86.0, 79.8, 67.7, 55.4, 34.5, 32.6, 32.5, 19.7 ppm; EI-MS (20 eV) *m/z* 192 (M^+, 9), 162 (45), 133 (48), 119 (100), 91 (87); HRMS (EI, 40eV) *m/z* $[M]^+$ calcd for $C_{12}H_{16}O_2$ 192.1145, found 192.1147.

Scalemic epoxy allylsilane **14** was also prepared for examining its (4+3) cycloaddition; however, the ee of **14** could not be accurately determined after trying a battery of chiral HPLC columns; thus the cycloaddition experimental outcomes were inconclusive.

13. Studies on the (3+2) cycloaddition of epoxy allylsilane 25l

13.1 Synthetic route for the synthesis of epoxy allylsilane **25l**

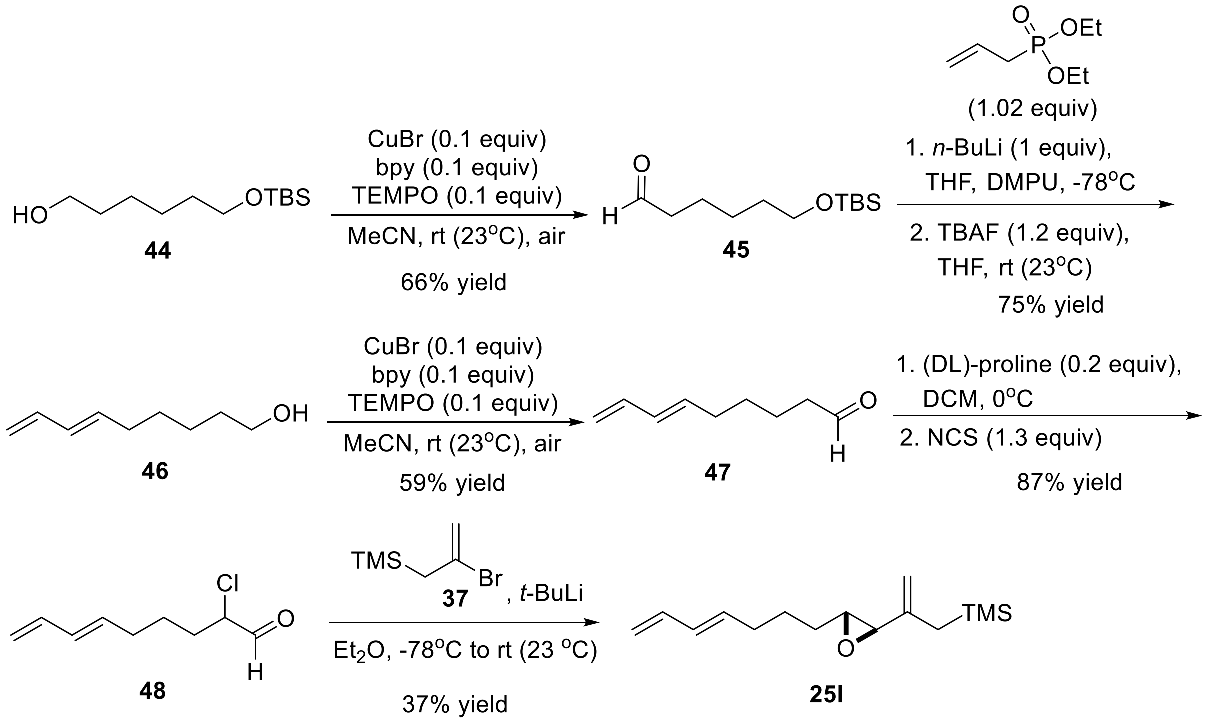

The preparations of compounds **45**[50], **46**[51], and **47**[52] have been reported and these compounds were prepared according to literature.

13.2 Preparation of **(*E*)-2-Chloronona-6,8-dienal (48)**

According to the procedure for the synthesis of **23a**, compound **48** was obtained from the treatment of aldehyde **47** (0.193 g, 1.40 mmol, 1.00 equiv) in anhydrous DCM (2.8 mL, 0.5 M) with (DL)-proline (32 mg, 0.28 mmol, 0.2 equiv) and NCS (0.24 g, 1.8 mmol, 1.3 equiv) at 0 °C. The crude material obtained was purified by flash column chromatography (eluent: 10% EtOAc in hexane) to afford **48** (0.21 g, 1.2 mmol, 87% yield).

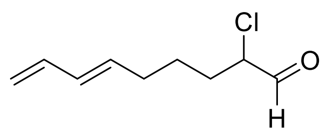

48

48: pale yellow oil; R_f (10% EtOAc in hexane) 0.30; IR (neat) 2931, 1743, 1651, 1603, 1456, 1002, 951 cm^{-1}; ^{1}H NMR (500 MHz, $CDCl_3$): δ 9.49 (d, *J* = 2.3 Hz, 1H), 6.30 (dt, *J* = 16.9, 10.3 Hz, 1H), 6.07 (dd, *J* = 15.2, 10.4 Hz, 1H), 5.66 (dt, *J* = 14.7, 7.0 Hz, 1H), 5.15 – 5.08 (m, 1H), 4.99 (d, *J* = 10.1 Hz, 1H), 4.17 (ddd, *J* = 8.1, 5.2, 2.3 Hz, 1H), 2.22 – 2.09 (m, 2H), 2.05 – 1.95 (m, 1H), 1.89 – 1.78 (m, 1H), 1.71 – 1.54 (m, 2H) ppm; ^{13}C{^{1}H} NMR (100 MHz, $CDCl_3$): δ 196.0, 137.1, 134.7, 132.4, 115.3, 64.0, 31.9, 31.6, 25.3 ppm; APCI-HRMS *m/z* calcd for $C_9H_{14}ClO$ [M+H]$^+$: 173.0728, found 173.0721.

13.3 Preparation of **(2-((2*S**,3*S**)-3-((*E*)-Hepta-4,6-dien-1-yl)oxiran-2-yl)allyl)trimethylsilane (25l)**

According to the procedure for the synthesis of **25a**, epoxy allylsilane **25l** was obtained from the treatment of α-chloroaldehyde **48** (0.13 g, 0.72 mmol, 1.0 equiv) in anhydrous THF (3.6 mL, 0.2 M) with **37** (0.18 g, 0.94 mmol, 1.3 equiv) in Et_2O (1.9 mL, 0.5 M) and *tert*-butyllithium (1.0 M in pentanes, 2.0 mL, 1.9 mmol, 2.7 equiv). The crude material obtained was purified by flash column chromatography (eluent: 0.3% Et_2O / hexane) to give **25l** (66 mg, 0.26 mmol, 37% yield).

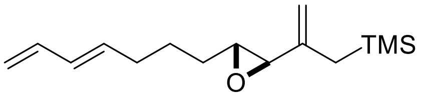

25l

25l: pale yellow oil; R_f(10% EtOAc in hexane) 0.79; IR (neat) 2947, 1635, 1416, 1248, 1158, 1002, 845 cm^{-1}; ^1H NMR (400 MHz, $CDCl_3$): δ 6.37 – 6.24 (m, 1H), 6.12 – 6.01 (m, 1H), 5.74 – 5.63 (m, 1H), 5.14 – 5.03 (m, 1H), 5.02 – 4.88 (m, 2H), 4.71 (s, 1H), 3.00 (d, *J* = 2.1 Hz, 1H), 2.79 – 2.72 (m, 1H), 2.20 – 2.04 (m, 3H), 1.69 – 1.56 (m, 3H), 1.44 (d, *J* = 3.2 Hz, 2H), 0.03 (s, 9H) ppm; $^{13}C\{^1H\}$ NMR (150 MHz, $CDCl_3$): δ 143.6, 137.3, 134.6, 131.6, 115.2, 109.4, 60.7, 59.6, 32.3, 31.7, 25.6, 21.8, -1.3 ppm; ESI-HRMS *m/z* calcd for $C_{15}H_{27}OSi$ $[M+H]^+$: 251.1826, found 251.1824.

13.4 Reaction of epoxy allylsilane **25l**

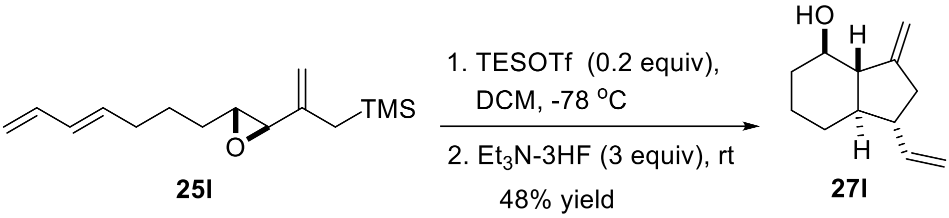

According to General Procedure B, **25l** (43.4 mg, 0.173 mmol, 1.00 equiv) in DCM (3.5 mL, 0.05 M) was treated with 0.2 M TESOTf in DCM (0.18 mL, 0.035 mmol, 0.2 equiv). The crude material obtained was purified by flash column chromatography (eluent: 10% EtOAc in hexane) to afford the **27l** (14.8 mg, 48% yield).

27l: white solid; R_f(10% EtOAc in hexane) 0.28; IR (neat) 3338, 2933, 2854, 1656, 1360, 1016, 911, 805 cm^{-1}; ^1H NMR (600 MHz, $CDCl_3$): δ 5.65 (ddd, *J* = 17.0, 10.2, 8.0 Hz, 1H), 5.33 – 5.29 (m, 1H), 5.06 – 4.96 (m, 2H), 4.86 – 4.83 (m, 1H), 3.70 – 3.63 (m, 1H), 2.64 (dddd, *J* = 16.7, 8.3, 3.8, 1.9 Hz, 1H), 2.23 – 2.15 (m, 1H), 2.15 – 2.07 (m, 1H), 2.04 – 1.97 (m, 1H), 1.84 – 1.76 (m, 3H), 1.32 – 1.19 (m, 3H), 1.08 – 0.99 (m, 1H), 0.99 – 0.85 (m, 1H) ppm; $^{13}C\{^1H\}$ NMR (150 MHz, $CDCl_3$): δ 151.8, 141.2, 114.5, 105.4, 73.8, 56.3, 50.0, 47.7, 38.3, 36.6, 29.2, 24.8 ppm; HRMS (EI, 40eV) *m/z* $[M]^+$ calcd for $C_{12}H_{18}O_3$ 178.1352, found 178.1351; m.p. 48.9-51.4 °C.

14. References

[1] B. M. Trost, T. A. Grese, D. M. T. Chan, "Intramolecular Palladium-Catalyzed Trimethylenemethane Cycloadditions: Initial Studies" *J. Am. Chem. Soc.* **1991**, *113*, 7350-7362.

[2] B. M. Trost, S. M. Silverman, J. P. Stambuli, "Development of an Asymmetric Trimethylenemethane Cycloaddition Reaction: Application in the Enantioselective Synthesis of Highly Substituted Carbocycles" *J. Am. Chem. Soc.* **2011**, *133*, 19483-19497.

[3] M. H. Nguyen, M. K. Imanishi, T., A. B. Smith, "Total Synthesis of (–)-Mandelalide A Exploiting Anion Relay Chemistry (ARC): Identification of a Type II ARC/CuCN Cross-Coupling Protocol" *J. Am. Chem. Soc.* **2016**, *138*, 3675-3678.

[4] M. J. Frisch, G. W. Trucks, H. B. Schlegel, G. E. Scuseria, M. A. Robb, J. R. Cheeseman, G. Scalmani, V. Barone, G. A. Petersson, H. Nakatsuji, X. Li, M. Caricato, A. V. Marenich, J. Bloino, B. G. Janesko, R. Gomperts, B. Mennucci, H. P. Hratchian, J. V. Ortiz, A. F. Izmaylov, J. L. Sonnenberg, Williams, F. Ding, F. Lipparini, F. Egidi, J. Goings, B. Peng, A. Petrone, T. Henderson, D. Ranasinghe, V. G. Zakrzewski, J. Gao, N. Rega, G. Zheng, W. Liang, M. Hada, M. Ehara, K. Toyota, R. Fukuda, J. Hasegawa, M. Ishida, T. Nakajima, Y. Honda, O. Kitao, H. Nakai, T. Vreven, K. Throssell, J. A. Montgomery Jr., J. E. Peralta, F. Ogliaro, M. J. Bearpark, J. J. Heyd, E. N. Brothers, K. N. Kudin, V. N. Staroverov, T. A. Keith, R. Kobayashi, J. Normand, K. Raghavachari, A. P. Rendell, J. C. Burant, S. S. Iyengar, J. Tomasi, M. Cossi, J. M. Millam, M. Klene, C. Adamo, R. Cammi, J. W. Ochterski, R. L. Martin, K. Morokuma, O. Farkas, J. B. Foresman, D. J. Fox, "Gaussian 16 Rev. C.01. Gaussian, Inc., Wallingford, CT" **2016**.

[5] V. Barone, M. Cossi, "Quantum Calculation of Molecular Energies and Energy Gradients in Solution by a Conductor Solvent Model" *J. Phys. Chem. A* **1998**, *102*, 1995-2001.

[6] M. Cossi, N. Rega, G. Scalmani, V. Barone, "Energies, Structures, and Electronic Properties of Molecules in Solution with the C-PCM Solvation Model" *J. Comput. Chem.* **2003**, *24*, 669-681.

[7] P. C. Hariharan, J. A. Pople, "The Influence of Polarization Functions on Molecular Orbital Hydrogenation Energies" *Theor. Chim. Acta.* **1973**, *28*, 213-222.

[8] S. Grimme, J. Antony, S. Ehrlich, H. Krieg, "A Consistent and Accurate Ab Initio Parametrization of Density Functional Dispersion Correction (DFT-D) for the 94 Elements H-Pu." *J. Chem. Phys.* **2010**, *132*, 154104.

[9] W. J. Hehre, R. Ditchfield, J. A. Pople, "Self—Consistent Molecular Orbital Methods. XII. Further Extensions of Gaussian—Type Basis Sets for Use in Molecular Orbital Studies of Organic Molecules" *J. Chem. Phys.* **1972**, *56*, 2257-2261.

[10] R. Ditchfield, W. J. Hehre, J. Pople, "Self-Consistent Molecular-Orbital Methods. IX. An Extended Gaussian-Type Basis for Molecular-Orbital Studies of Organic Molecules." *J. Chem. Phys.* **1971**, *54*, 724–728.

[11] S. Grimme, S. Ehrlich, L. Goerigk, "Effect of the Damping Function in Dispersion Corrected Density Functional Theory" *J. Comput. Chem.* **2011**, *32*, 1456-1465.

[12] M. S. Gordon, J. S. Binkley, J. A. Pople, W. J. Pietro, W. J. Hehre, "Self-consistent Molecular-Orbital Methods. 22. Small Split-valence Basis Sets for Second-Row Elements" *J. Am. Chem. Soc.* **1982**, *104*, 2797-2803.

[13] M. M. Francl, W. J. Pietro, W. J. Hehre, J. S. Binkley, M. S. Gordon, D. J. DeFrees, J. Pople, "Self-Consistent Molecular Orbital Methods. XXIII. A Polarization-Type Basis Set for Second-Row Elements." *J. Chem. Phys.* **1982**, *77*, 3654–3665.

[14] P. J. Stephens, F. J. Devlin, C. F. Chabalowski, M. J. Frisch, "Ab Initio Calculation of Vibrational Absorption and Circular Dichroism Spectra Using Density Functional Force Fields" *J. Phys. Chem.* **1994**, *98*, 11623-11627.

[15] L. Tian, C. Qinxue, "Shermo: A General Code for Calculating Molecular Thermochemistry Properties" *Comput. Theor. Chem.* **2021**, *1200*, 113249.

[16] A. A. Otlyotov, Y. Minenkov, "Gas-phase Thermochemistry of Noncovalent Ligand–alkali Metal Ion Clusters: An Impact of Low Frequencies" *J. Comput. Chem.* **2023**, *44*, 1807-1816.

[17] S. Grimme, "Supramolecular Binding Thermodynamics by Dispersion-Corrected Density Functional Theory" *Chem. Eur. J.* **2012**, *18*, 9955-9964.

[18] A. V. Marenich, C. J. Cramer, D. G. Truhlar, "Universal Solvation Model Based on Solute Electron Density and on a Continuum Model of the Solvent Defined by the Bulk Dielectric Constant and Atomic Surface Tensions" *J. Phys. Chem. B* **2009**, *113*, 6378-6396.

[19] Y. Zhao, D. G. Truhlar, "The M06 Suite of Density Functionals for Main Group Thermochemistry, Thermochemical Kinetics, Noncovalent Interactions, Excited States, and Transition Elements: Two New Functionals and Systematic Testing of Four M06-class Functionals and 12 Other Functionals" *Theor. Chem. Acc.* **2008**, *120*, 215-241.

[20] F. Weigend, R. Ahlrichs, "Balanced Basis Sets of Split Valence, Triple Zeta Valence and Quadruple Zeta Valence Quality for H to Rn: Design and Assessment of Accuracy" *Phys. Chem. Chem. Phys.* **2005**, *7*, 3297-3305.

[21] T. Lu, F. Chen, "Multiwfn: A Multifunctional Wavefunction Analyzer" *J. Comput. Chem.* **2012**, *33*, 580-592.

[22] T. Lu, Q. Chen, "Independent Gradient Model Based on Hirshfeld Partition: A New Method for Visual Study of Interactions in Chemical Systems" *J. Comput. Chem.* **2022**, *43*, 539-555.

[23] T. Lu, "A Comprehensive Electron Wavefunction Analysis Toolbox for Chemists,

Multiwfn" *J. Chem. Phys.* **2024**, *161*.

[24] H. L. Schmider, A. D. Becke, "Chemical Content of the Kinetic Energy Density" *J. Mol. Struct.:THEOCHEM* **2000**, *527*, 51-61.

[25] D. Kalaitzakis, E. Antonatou, G. Vassilikogiannakis, "One-pot synthesis of 1-azaspiro frameworks initiated by photooxidation of simple furans" *Chem. Commun.* **2014**, *50*, 400-402.

[26] W. K. Chung, S. K. Lam, B. Lo, L. L. Liu, W.-T. Wong, P. Chiu, "Inter- and Intramolecular [4 + 3] Cycloadditions Using Epoxy Enol Silanes As Functionalized Oxyallyl Cation Precursors" *J. Am. Chem. Soc.* **2009**, *131*, 4556-4557.

[27] S. A. Green, J. L. M. Matos, A. Yagi, R. A. Shenvi, "Branch-Selective Hydroarylation: Iodoarene–Olefin Cross-Coupling" *J. Am. Chem. Soc.* **2016**, *138*, 12779-12782.

[28] D. Kalaitzakis, M. Triantafyllakis, I. Alexopoulou, M. Sofiadis, G. Vassilikogiannakis, "One-Pot Transformation of Simple Furans into 4-Hydroxy-2-cyclopentenones in Water" *Angew. Chem., Int. Ed. Engl.* **2014**, *53*, 13201.

[29] J. He, Z. Chen, W. Li, K. H. Low, P. Chiu, "Intramolecular (4+3) Cycloadditions of Pyrroles and Application to the Synthesis of the Core of Class II Galbulimima Alkaloids" *Angew. Chem., Int. Ed.* **2018**, *57*, 5253-5256.

[30] L. Yan, Y. Meng, F. Haeffner, R. M. Leon, M. P. Crockett, J. P. Morken, "Carbohydrate/DBU Cocatalyzed Alkene Diboration: Mechanistic Insight Provides Enhanced Catalytic Efficiency and Substrate Scope" *J. Am. Chem. Soc.* **2018**, *140*, 3663-3673.

[31] Y. Zheng, PhD Thesis. University of Hong Kong, Pokfulam, Hong Kong SAR **2022**.

[32] S. Schoenauer, P. Schieberle, "Structure–Odor Correlations in Homologous Series of Mercapto Furans and Mercapto Thiophenes Synthesized by Changing the Structural Motifs of the Key Coffee Odorant Furan-2-ylmethanethiol" *J. Agric. Food Chem.* **2018**, *66*, 4189-4199.

[33] P. Weyerstahl, A. Schenk, H. Marschall, "Structure-odor Correlation, XXI. Olfactory Properties and Convenient Synthesis of Furans and Thiophenes Related to Rose Furan and Perillene and Their Isomers." *Liebigs Ann. Chem.* **1995**, *10*, 1849-1853.

[34] M. J. Sun, Y. J. Deng, E. Batyreva, W. Sha, R. G. Salomon, "Novel bioactive phospholipids: Practical total syntheses of products from the oxidation of arachidonic and linoleic esters of 2-lysophosphatidylcholine" *J. Org. Chem.* **2002**, *67*, 3575-3584.

[35] J. Li, H. Peng, F. Wang, X. Wang, H. Jiang, B. Yin, "2,5-Oxyarylation of Furans: Synthesis of Spiroacetals via Palladium-Catalyzed Aerobic Oxidative Coupling of Boronic Acids with α-Hydroxyalkylfurans" *Org. Lett.* **2016**, *18*, 3226-3229.

[36] R. R. Cesati, G. Dwyer, R. C. Jones, M. P. Hayes, P. Yalamanchili, D. S. Casebier, "Amino Acid Derived Enamides: Synthesis and Aminopeptidase Activity" *Org. Lett.* **2007**, *9*, 5617-5620.

[37] M. B. Groen, B. Hindriksen, F. J. Zeelen, "Biomimetic Total Synthesis of Steroids VI. Stereospecific Synthesis of 19-nor-8α-steroids and 11α-alkyl Derivatives." *Recl. Trav. Chim. Pays-Bas* **1982**, *101*, 148-155.

[38] J. Hu, X. Han, Y. Yuan, Z. Shi, "Stereoselective Synthesis of Z Fluoroalkenes through Copper-Catalyzed Hydrodefluorination of gem-Difluoroalkenes with Water" *Angew. Chem., Int. Ed.* **2017**, *56*, 13342-13346.

[39] J. P. Costello, E. M. Ferreira, "Regioselectivity Influences in Platinum-Catalyzed Intramolecular Alkyne O–H and N–H Additions" *Org. Lett.* **2019**, *21*, 9934-9939.

[40] M. Feuerstein, F. Berthiol, H. Doucet, M. Santelli, "Palladium-Tetraphosphine Complex: An Efficient Catalyst for the Alkynyl-ation of ortho-Substituted Aryl Bromides" *Synthesis* **2004**, *2004*, 1281-1289.

[41] K. C. Nicolaou, R. d. Reingruber, D. Sarlah, S. Bräse, "Enantioselective Intramolecular Friedel−Crafts-Type α-Arylation of Aldehydes" *J. Am. Chem. Soc.* **2009**, *131*, 2086-2087.

[42] M. Inouye, T. Konishi, K. Isagawa, "Artificial allosteric receptors for nucleotide bases and alkali-metal cations" *J. Am. Chem. Soc.* **1993**, *115*, 8091-8095.

[43] A. Fanourakis, B. D. Williams, K. J. Paterson, R. J. Phipps, "Enantioselective Intermolecular C–H Amination Directed by a Chiral Cation" *J. Am. Chem. Soc.* **2021**, *143*, 10070-10076.

[44] F. Effenberger, G. Götz, P. Bäuerle, "Synthese Und Eigenschaften von Σ-verbrückten Anthracen-Viologenen" *Chem. Ber.* **1992**, *125*, 941-950.

[45] A. Di Sabato, F. D'Acunzo, D. Filippini, F. Vetica, A. Brasiello, D. Corinti, E. Bodo, C. Michenzi, E. Panzetta, P. Gentili, "Unusually Chemoselective Photocyclization of 2-(Hydroxyimino)aldehydes to Cyclobutanol Oximes: Synthetic, Stereochemical, and Mechanistic Aspects" *J. Org. Chem.* **2022**, *87*, 13803-13818.

[46] T. Lee, J. B. Jones, "Probing the Abilities of Synthetically Useful Serine Proteases To Discriminate Remote Stereocenters. Chiral Naphthyl Aldehyde Inhibitors" *J. Am. Chem. Soc.* **1997**, *119*, 10260-10268.

[47] N. Ahlsten, A. Bermejo Gómez, B. Martín-Matute, "Iridium-Catalyzed 1,3-Hydrogen Shift/Chlorination of Allylic Alcohols" *Angew. Chem.* **2013**, *125*, 6393-6396.

[48] Y. Zheng, Y. Chen, Y. He, A. Rizzo, Y. Zhou, K. H. Low, E. H. Krenske, P. Chiu, "Dearomative Intramolecular (4+3) Cycloadditions of Thiophenes" *Angew. Chem., Int. Ed.* **2024**, *63*, e202407059-n/a.

[49] B. Kang, R. Britton, "A General Method for the Synthesis of Nonracemic trans-Epoxides: Concise Syntheses of trans-Epoxide-Containing Insect Sex Pheromones" *Org. Lett.* **2007**, *9*, 5083-5086.

[50] J. D. Trenkle, T. F. Jamison, "Macrocyclization by Nickel-Catalyzed, Ester-Promoted, Epoxide-Alkyne Reductive Coupling: Total Synthesis of (−)-Gloeosporone" *Angew.*

Chem., Int. Ed. **2009**, *48*, 5366-5368.

[51] Y. Chen, J. Ling, A. B. Keto, Y. He, K. H. Low, E. H. Krenske, P. Chiu, "Chemoselective and Diastereoselective Intramolecular (3+2) Cycloadditions of Epoxy and Aziridinyl Enolsilanes" *Angew. Chem., Int. Ed.* **2022**, *61*, e202116099-n/a.

[52] W. D. Wulff, T. S. Powers, "Stereochemical Control in Intramolecular Diels-Alder Reactions with Carbene Complexes as Ester Synthons" *J. Org. Chem.* **1993**, *58*, 2381-2393.

15. ^{1}H and ^{13}C NMR spectra of isolated compounds

^{1}H NMR (500 MHz, $CDCl_3$): **37**

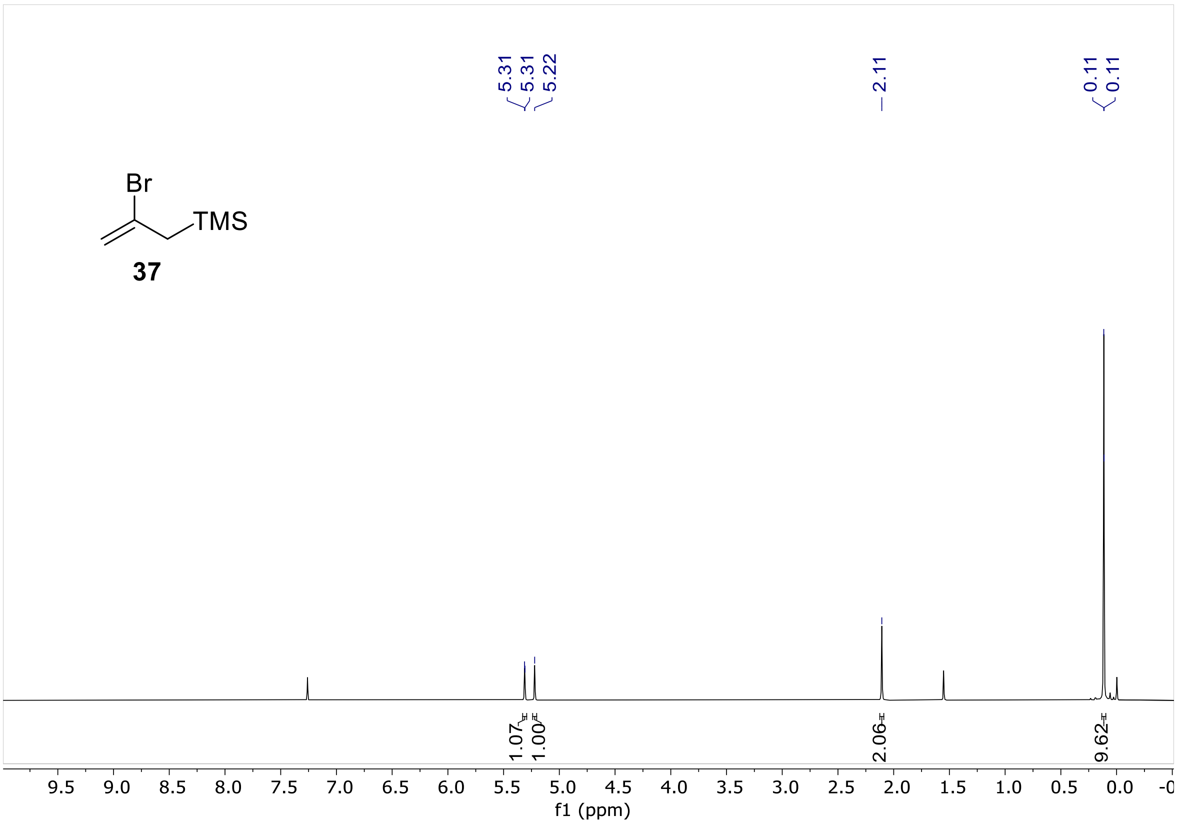

$^{13}C\{^1H\}$ NMR (125 MHz, $CDCl_3$): **37**

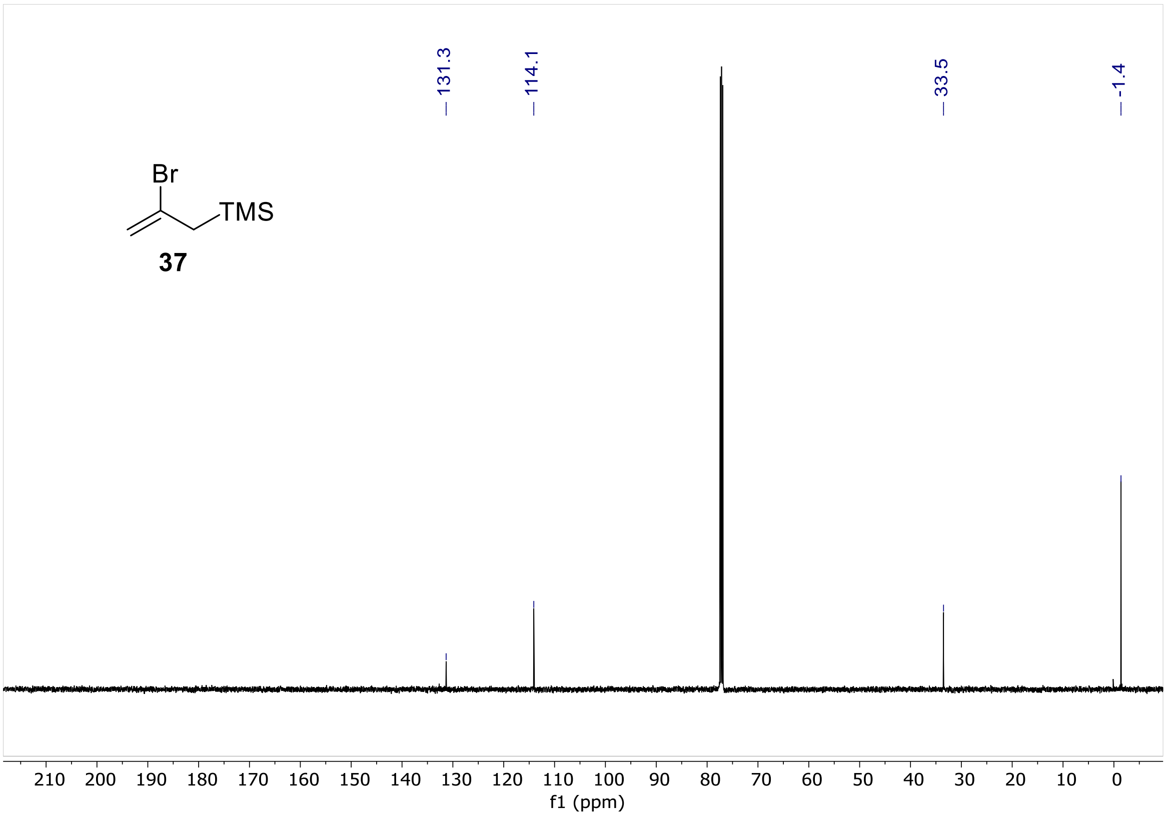

^{1}H NMR (500 MHz, $CDCl_3$): **38**

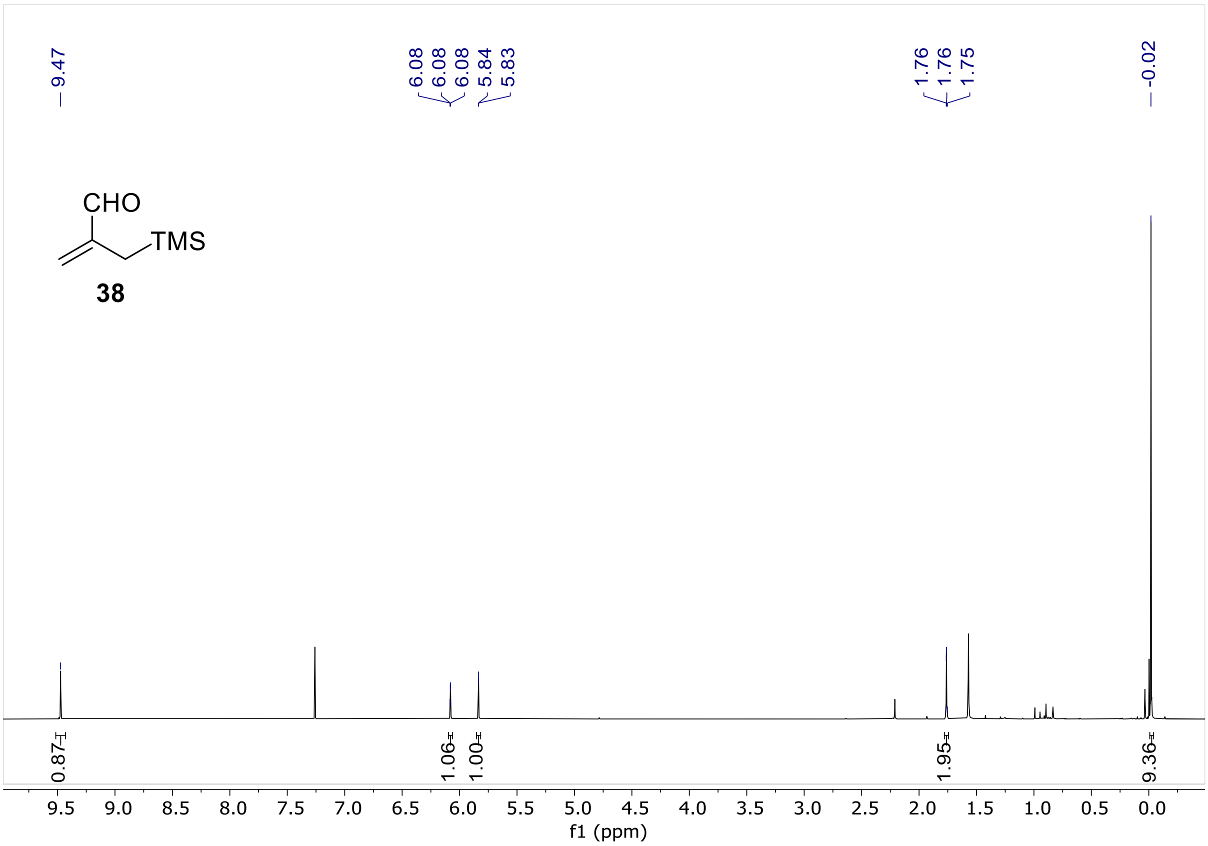

$^{13}C\{^{1}H\}$ NMR (100 MHz, $CDCl_3$): **38**

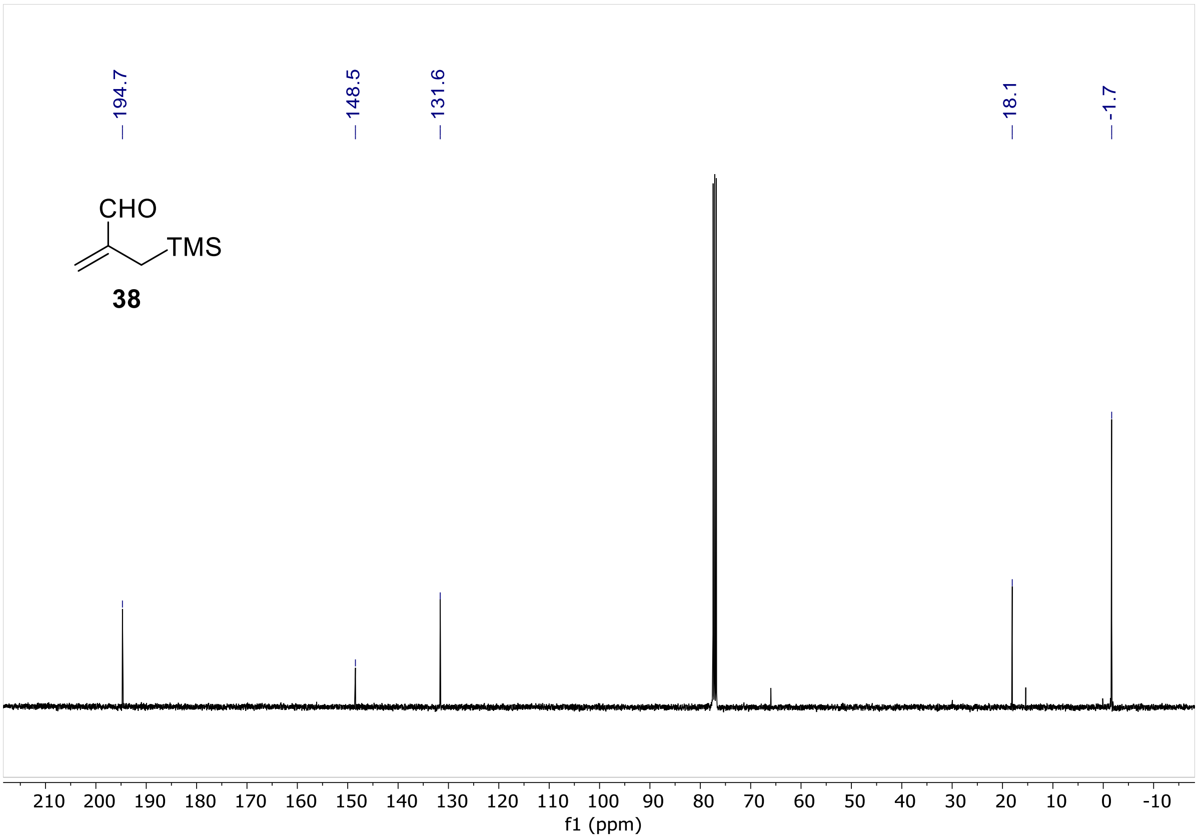

^{1}H NMR (500 MHz, $CDCl_3$): **14**

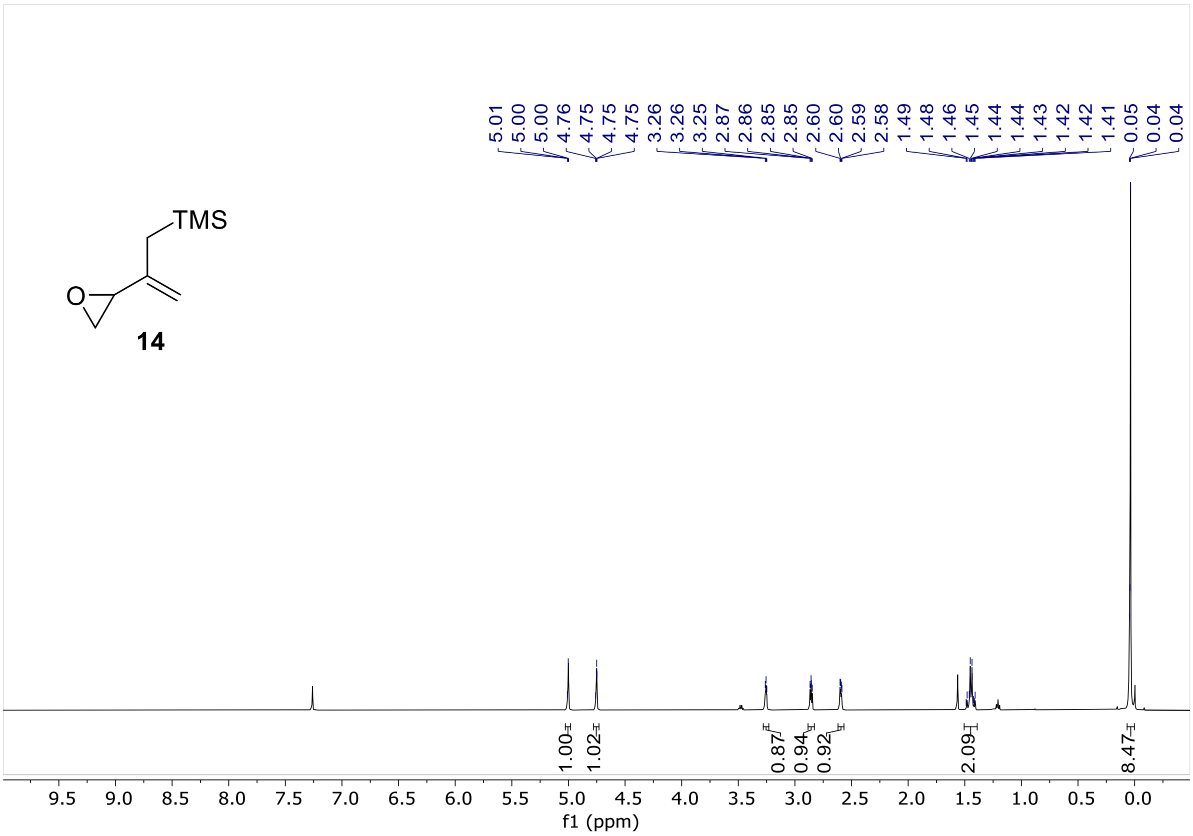

^{13}C{^{1}H} NMR (100 MHz, $CDCl_3$): **14**

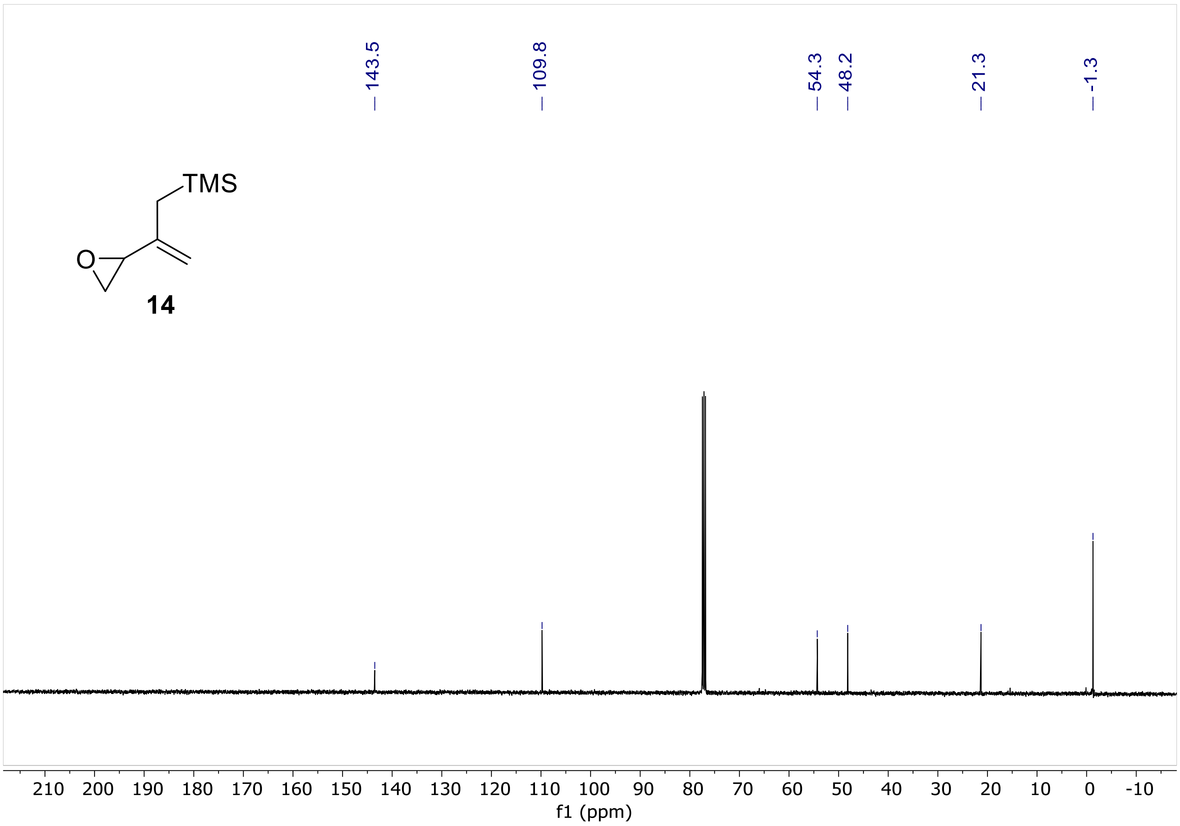

^1H NMR (600 MHz, $CDCl_3$): **15a**

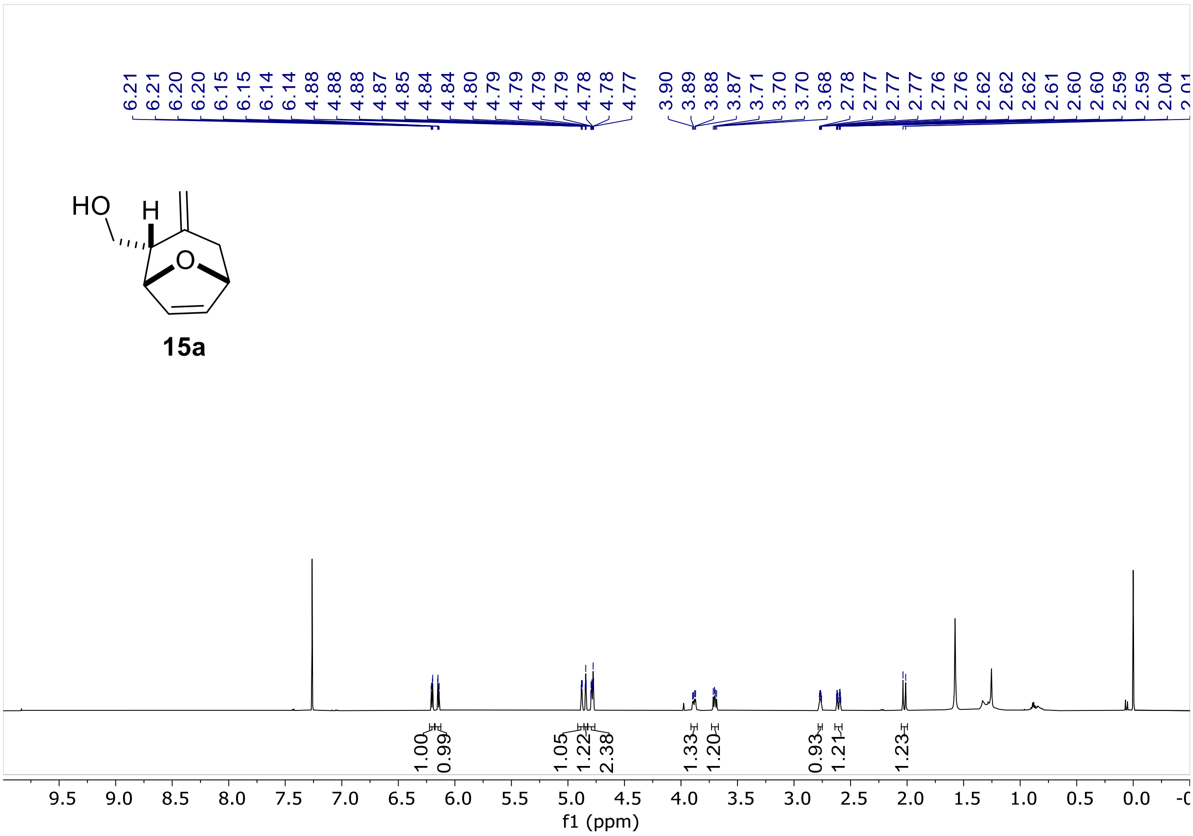

$^{13}C\{^1H\}$ NMR (150 MHz, $CDCl_3$): **15a**

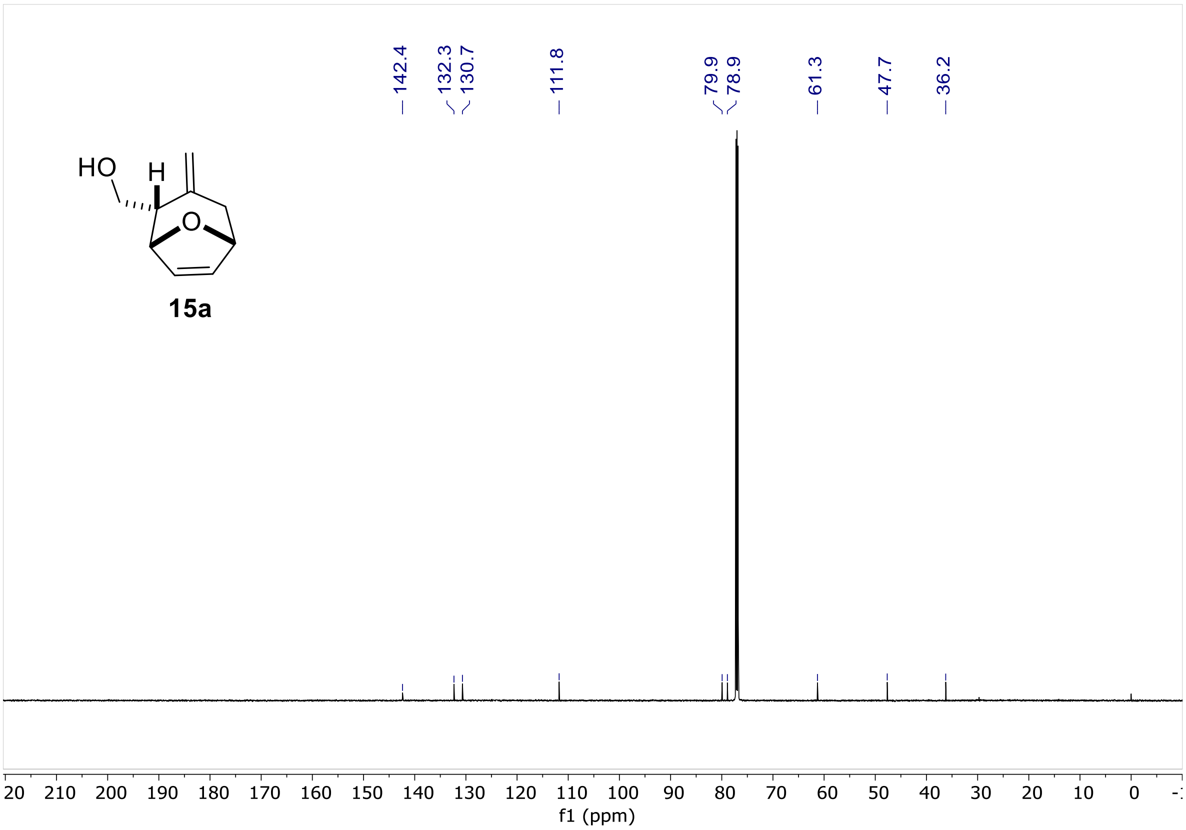

^{1}H NMR (600 MHz, $CDCl_3$): **16a**

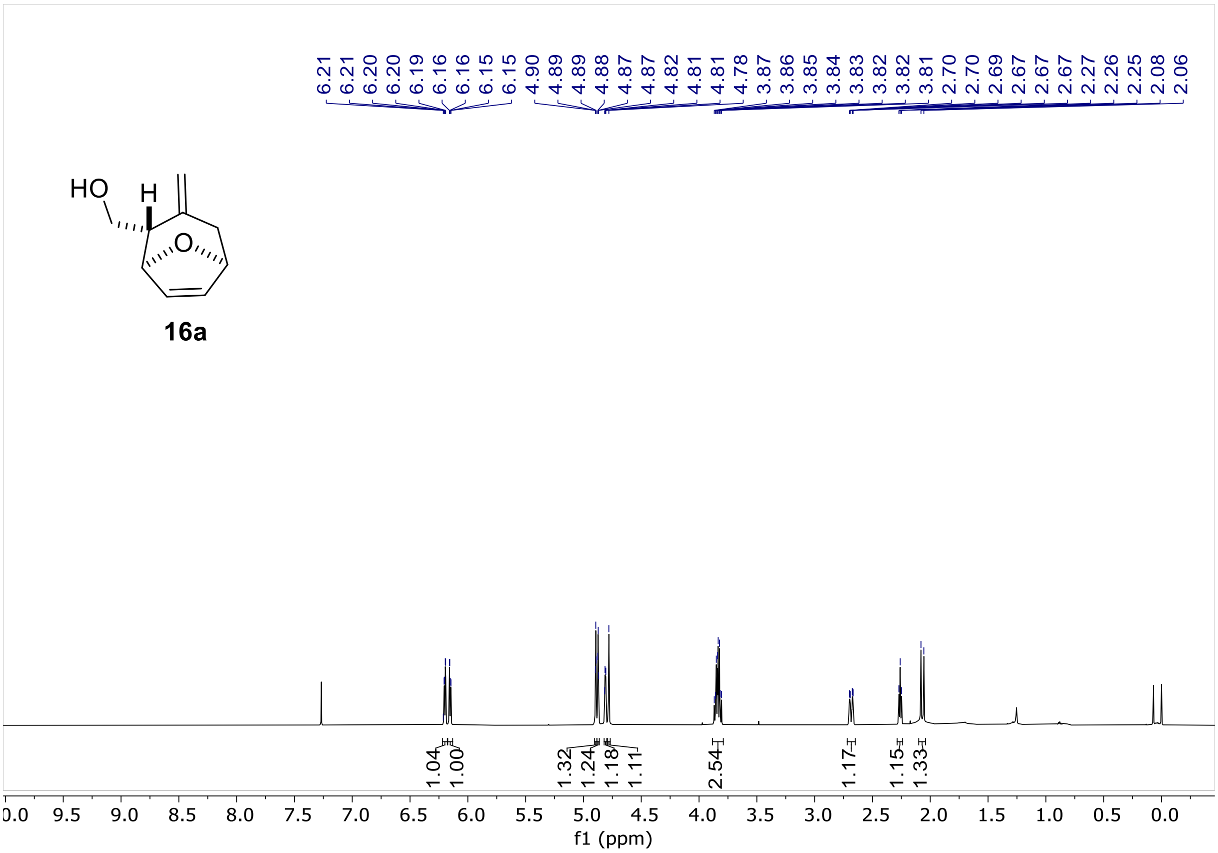

$^{13}C\{^{1}H\}$ NMR (150 MHz, $CDCl_3$): **16a**

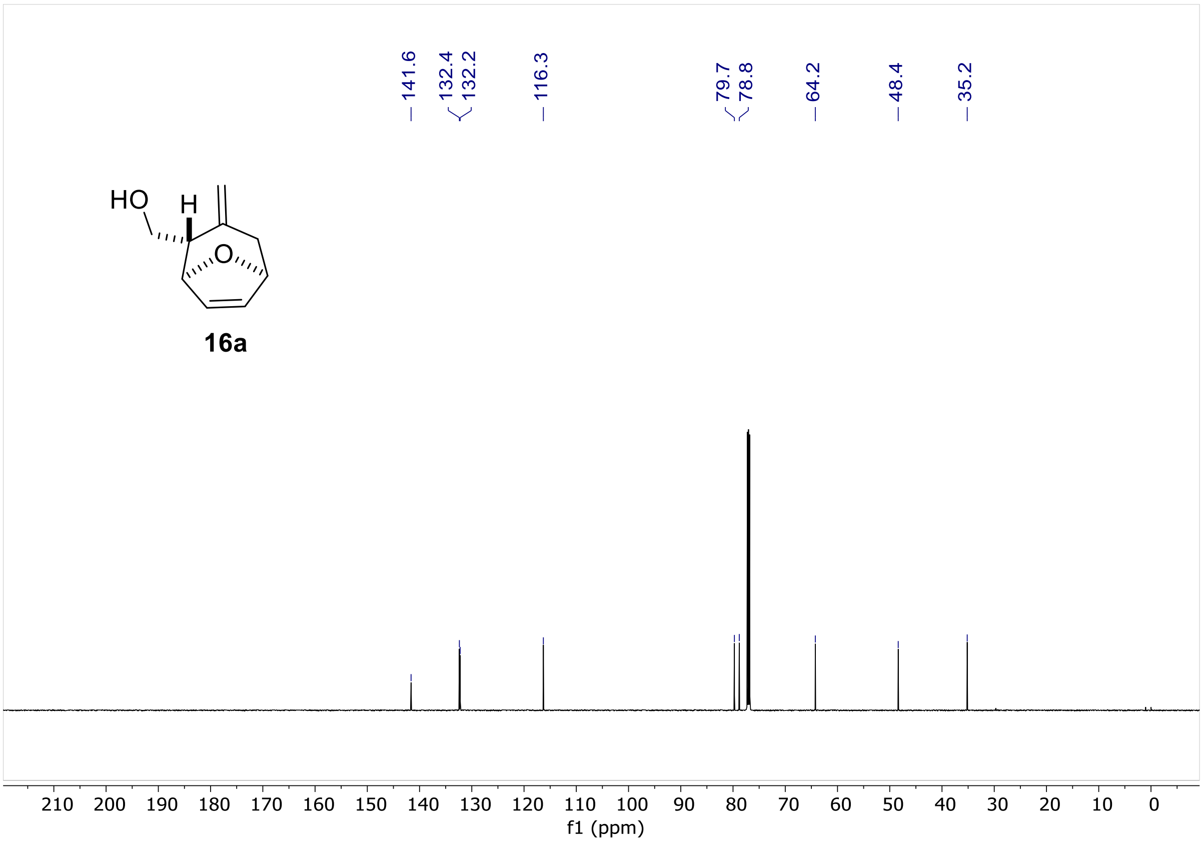

^{1}H NMR (500 MHz, $CDCl_3$): **17a**

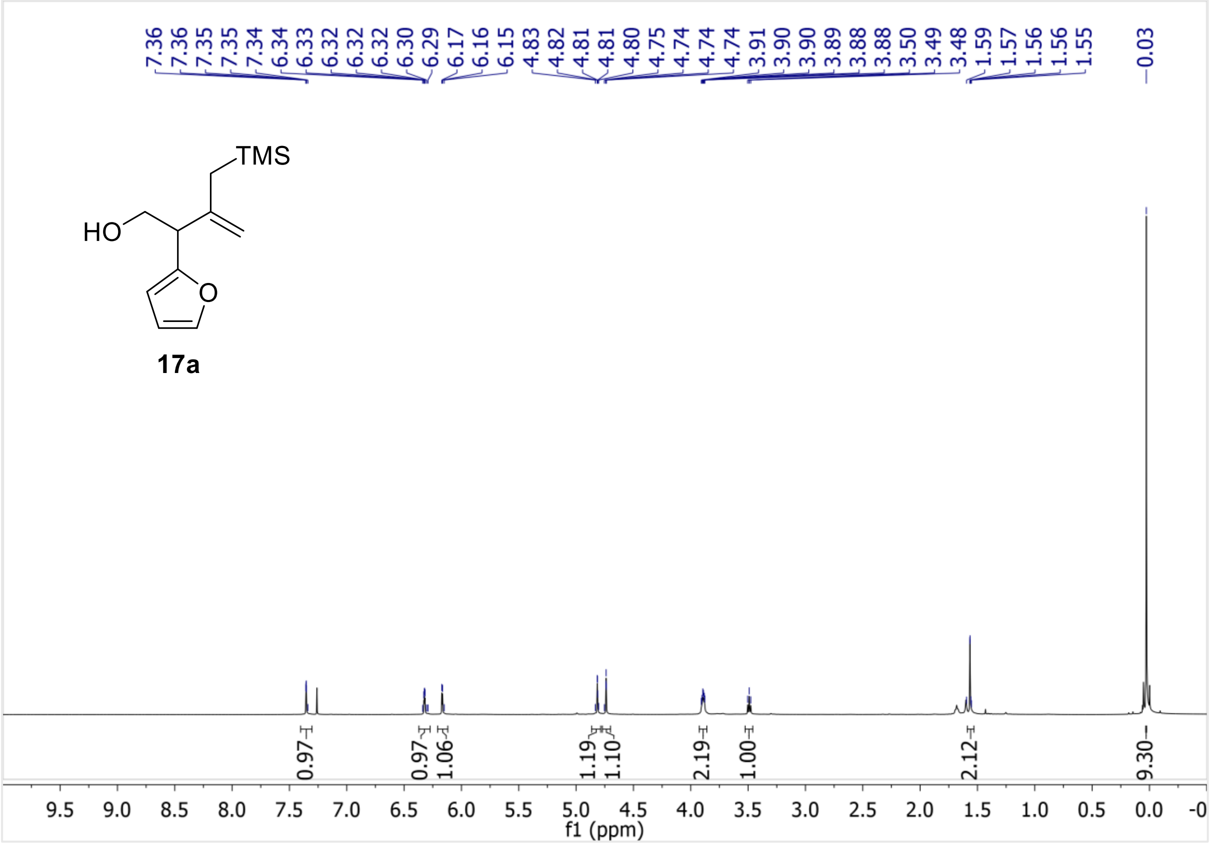

$^{13}C\{^1H\}$ NMR (100 MHz, $CDCl_3$): **17a**

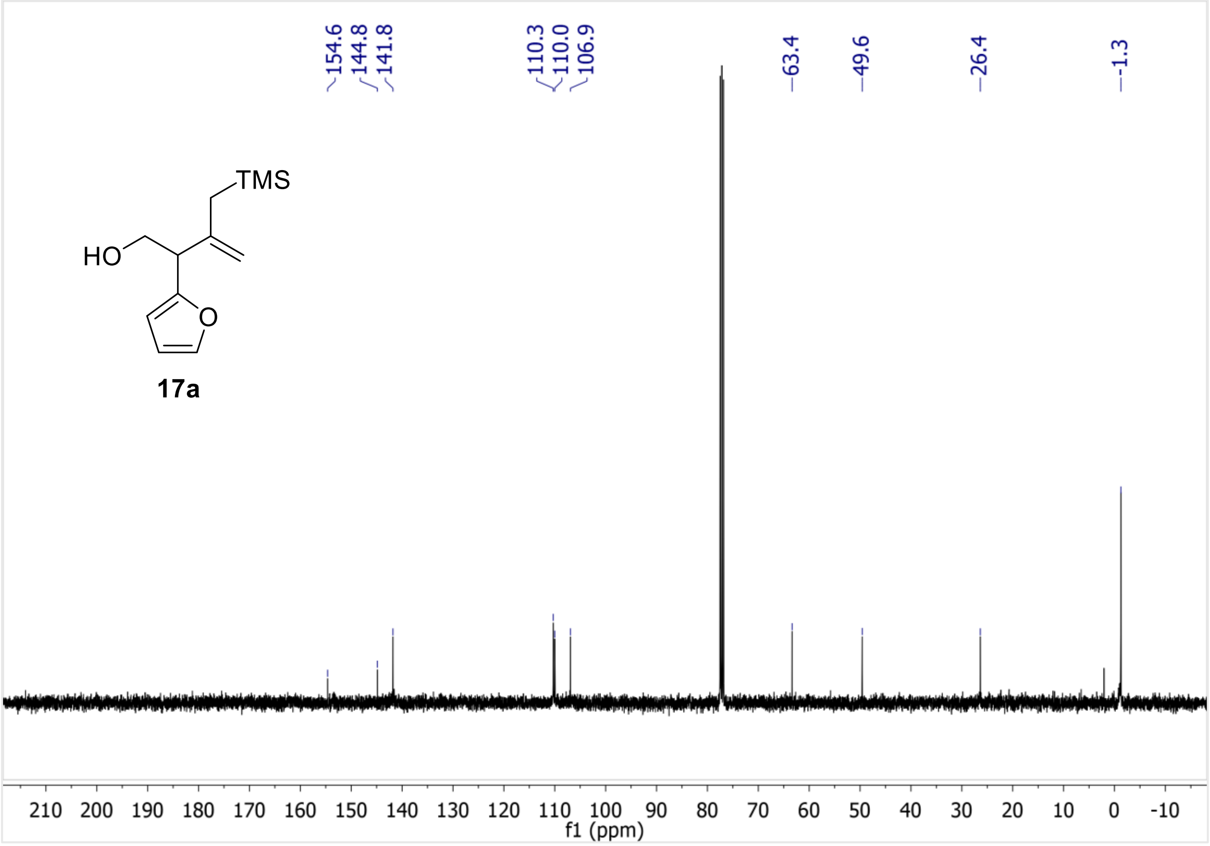

^{1}H NMR (400 MHz, $CDCl_3$): **17b**

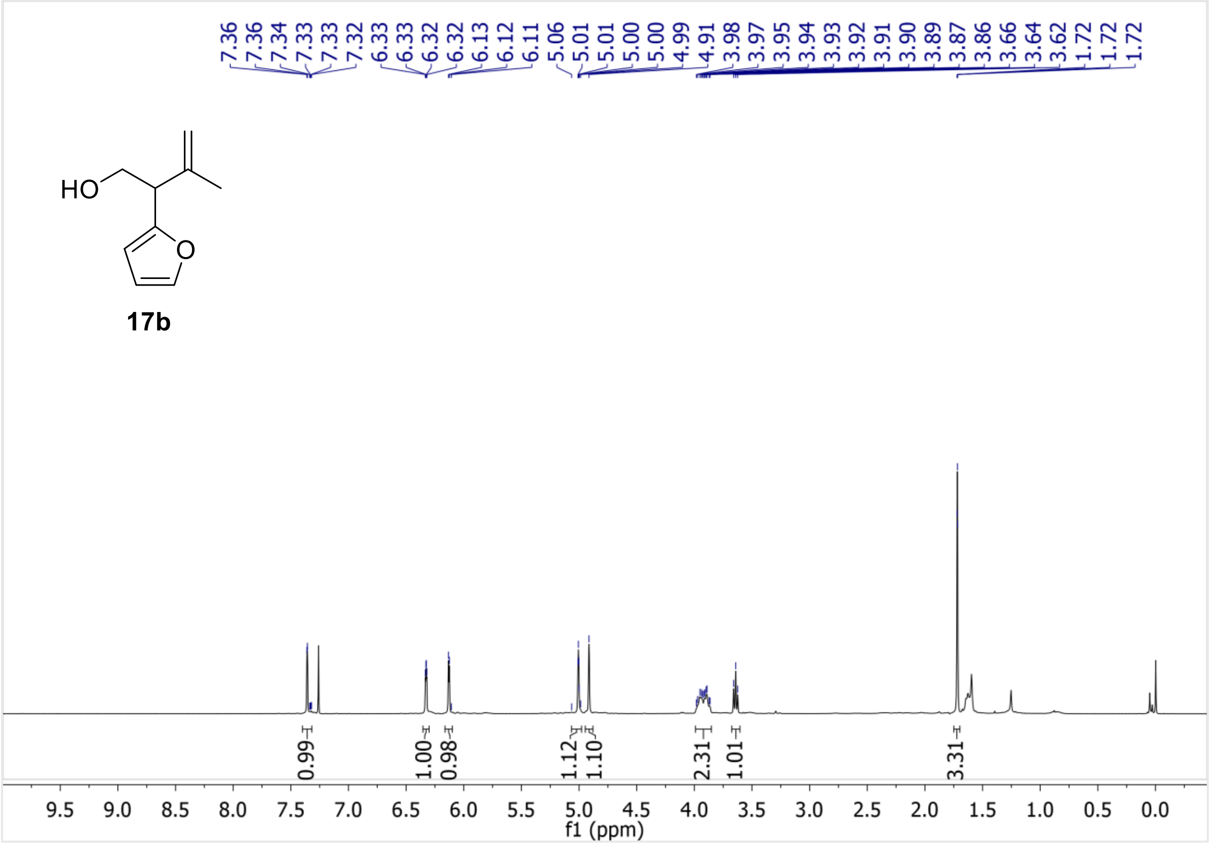

$^{13}C\{^{1}H\}$ NMR (100 MHz, $CDCl_3$): **17b**

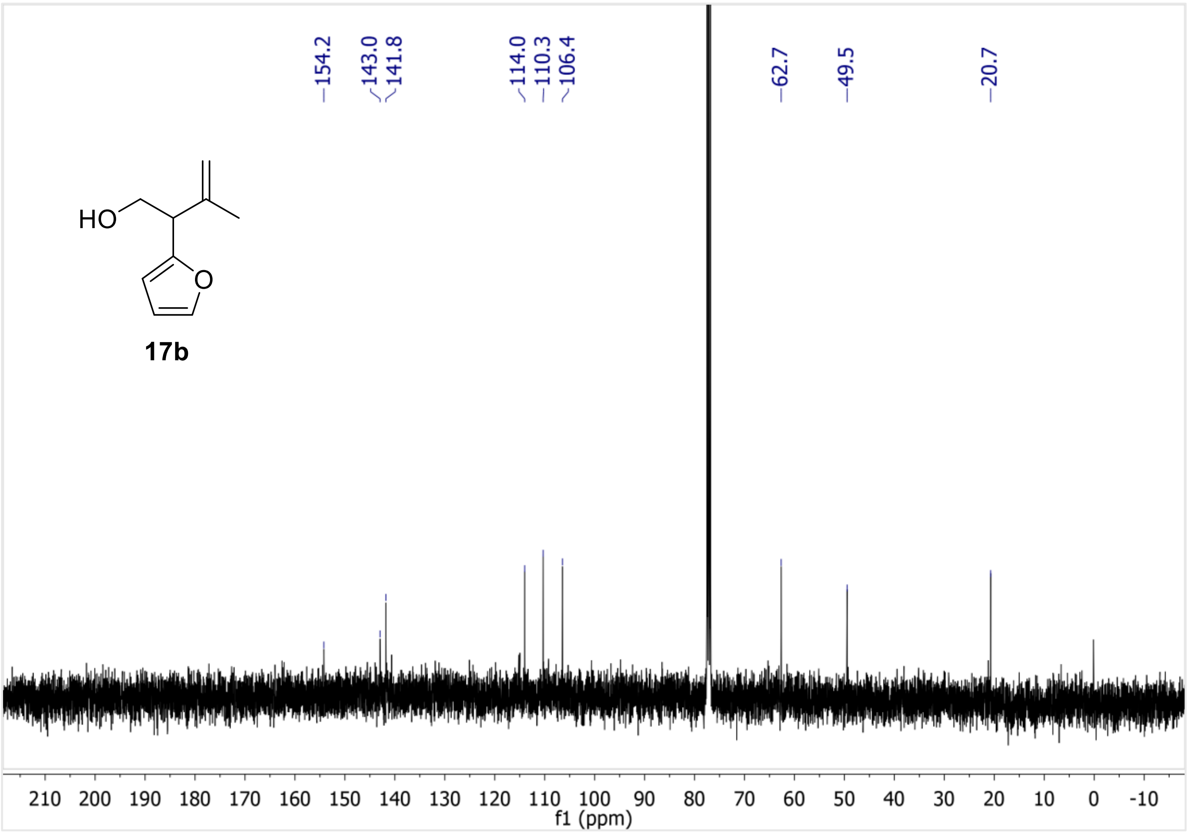

^{1}H NMR (400 MHz, $CDCl_3$): **17c**

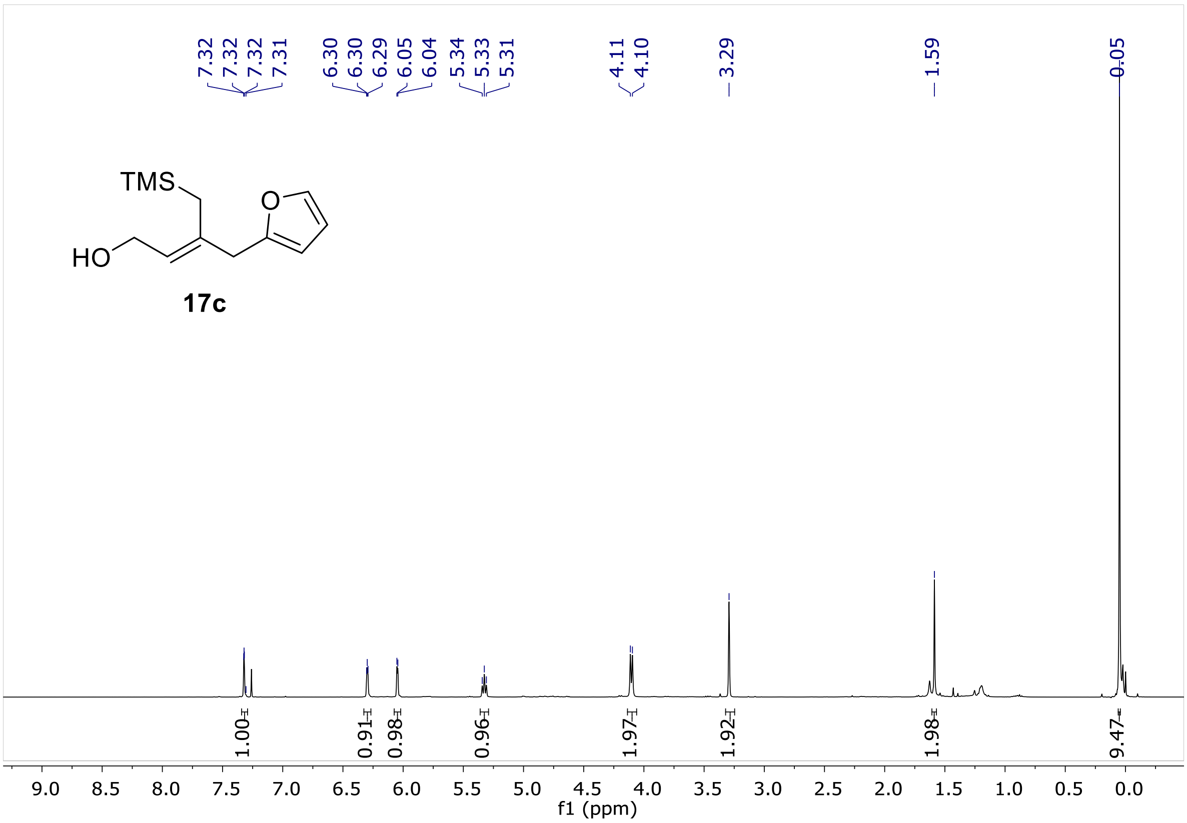

^{13}C{^{1}H} NMR (100 MHz, $CDCl_3$): **17c**

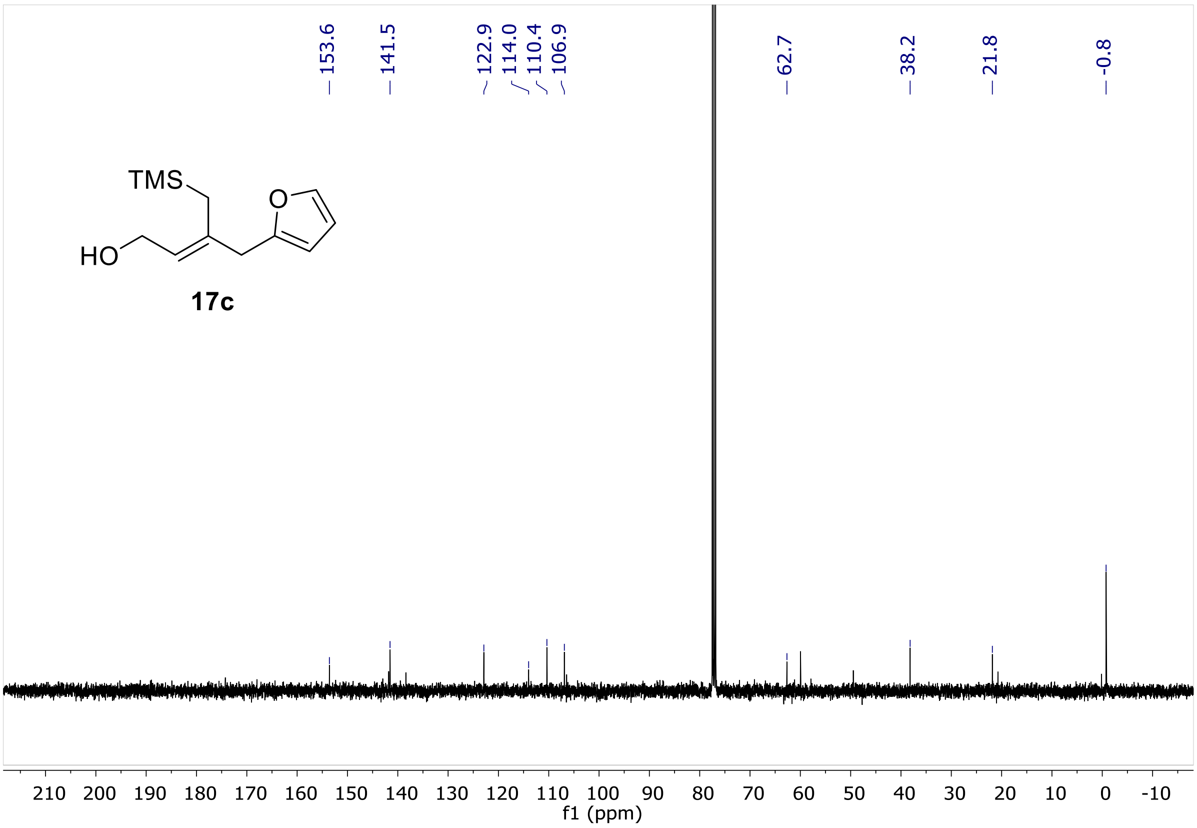

^{1}H NMR (400 MHz, $CDCl_3$): **15b + 16b (15b** and **16b** are inseparable)

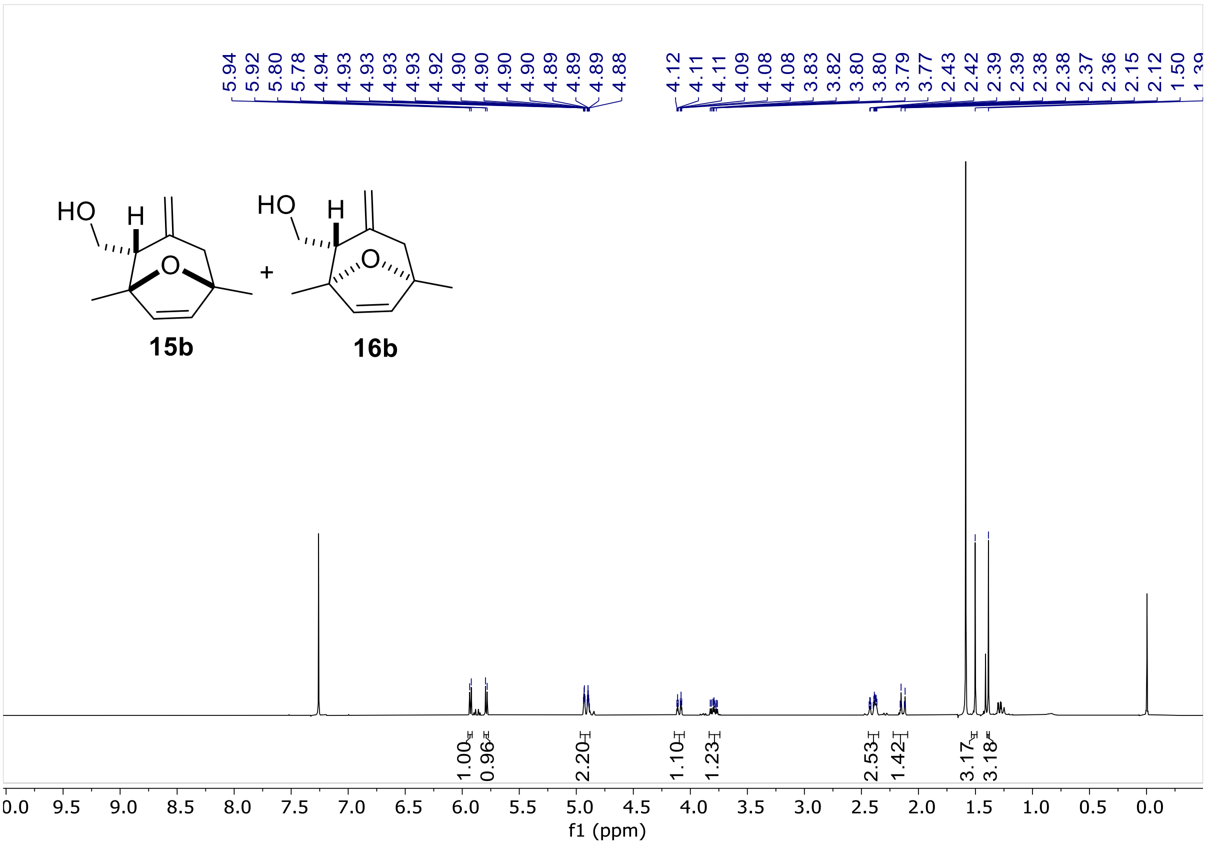

$^{13}C\{^{1}H\}$ NMR (100 MHz, $CDCl_3$): **15b + 16b (15b** and **16b** are inseparable)

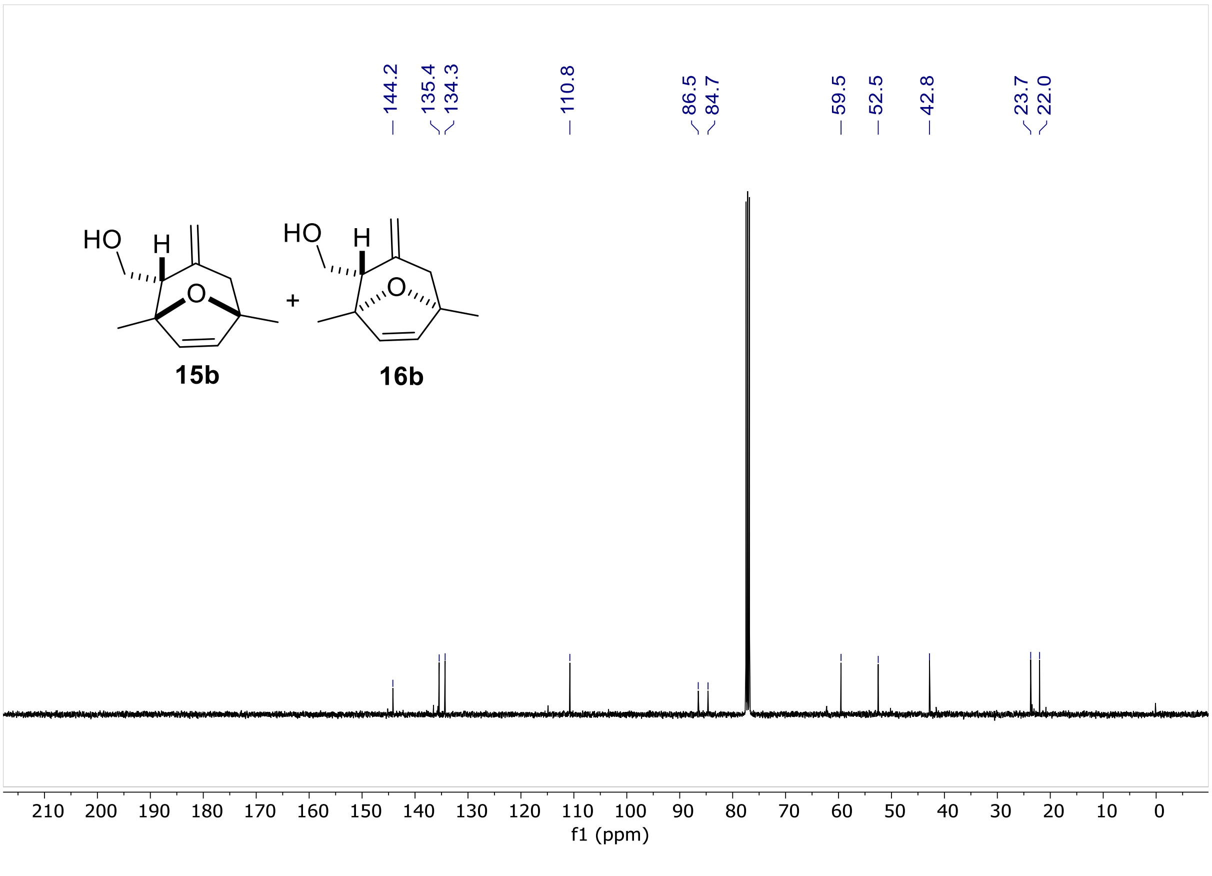

^{1}H NMR (500 MHz, $CDCl_3$): **17d**

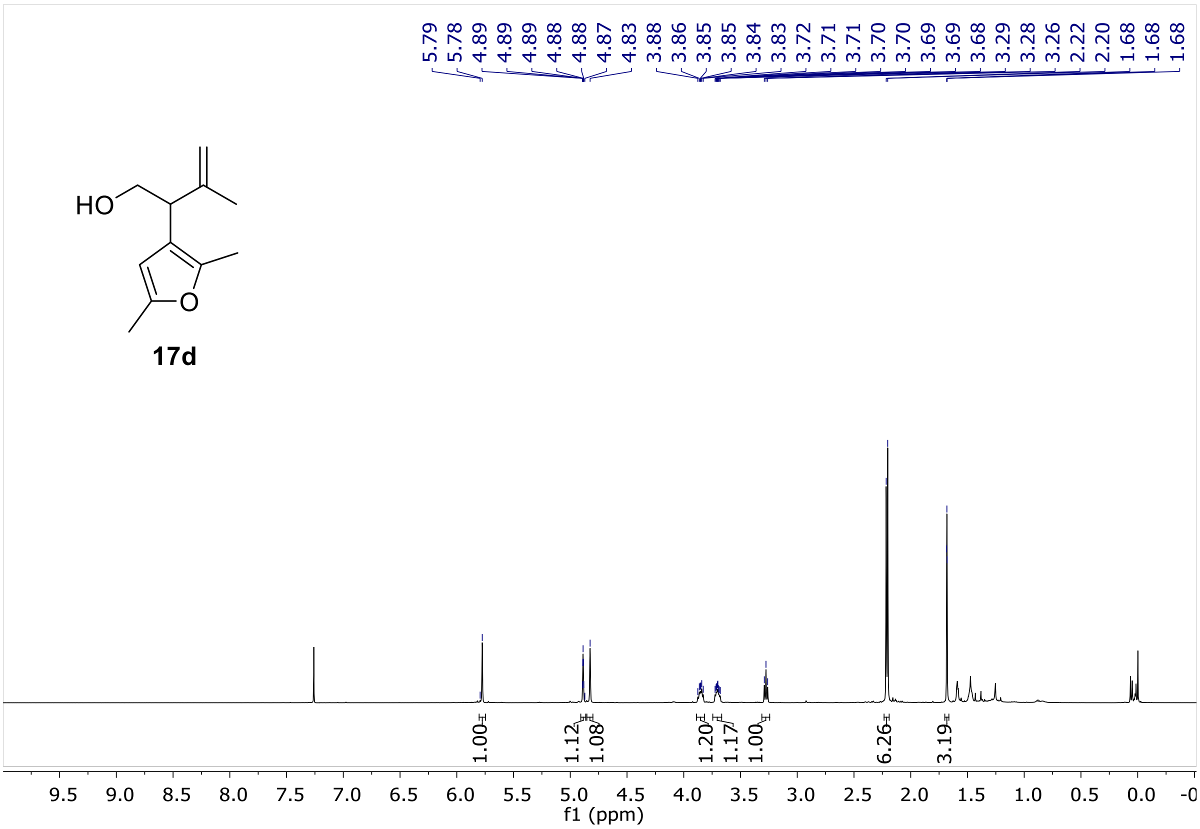

^{13}C{^{1}H} NMR (125 MHz, $CDCl_3$): **17d**

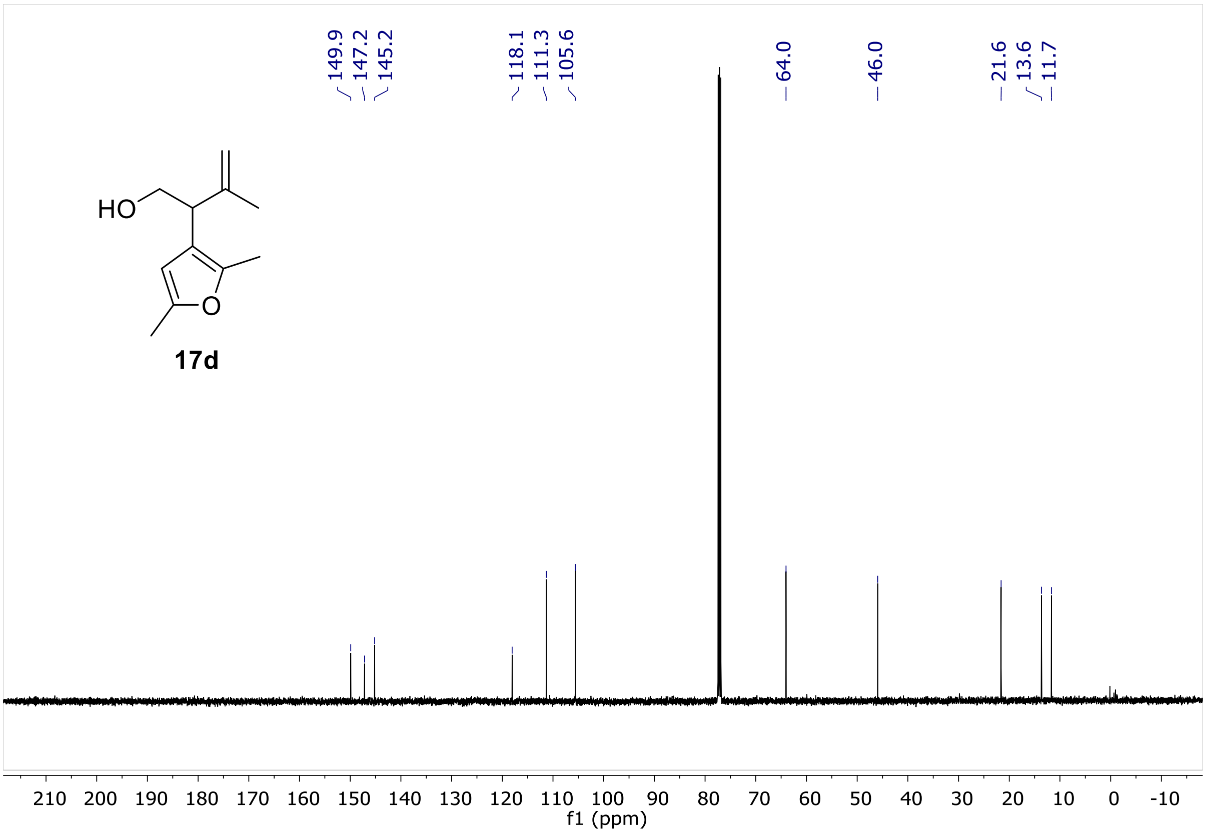

^{1}H NMR (600 MHz, $CDCl_3$): **15c**

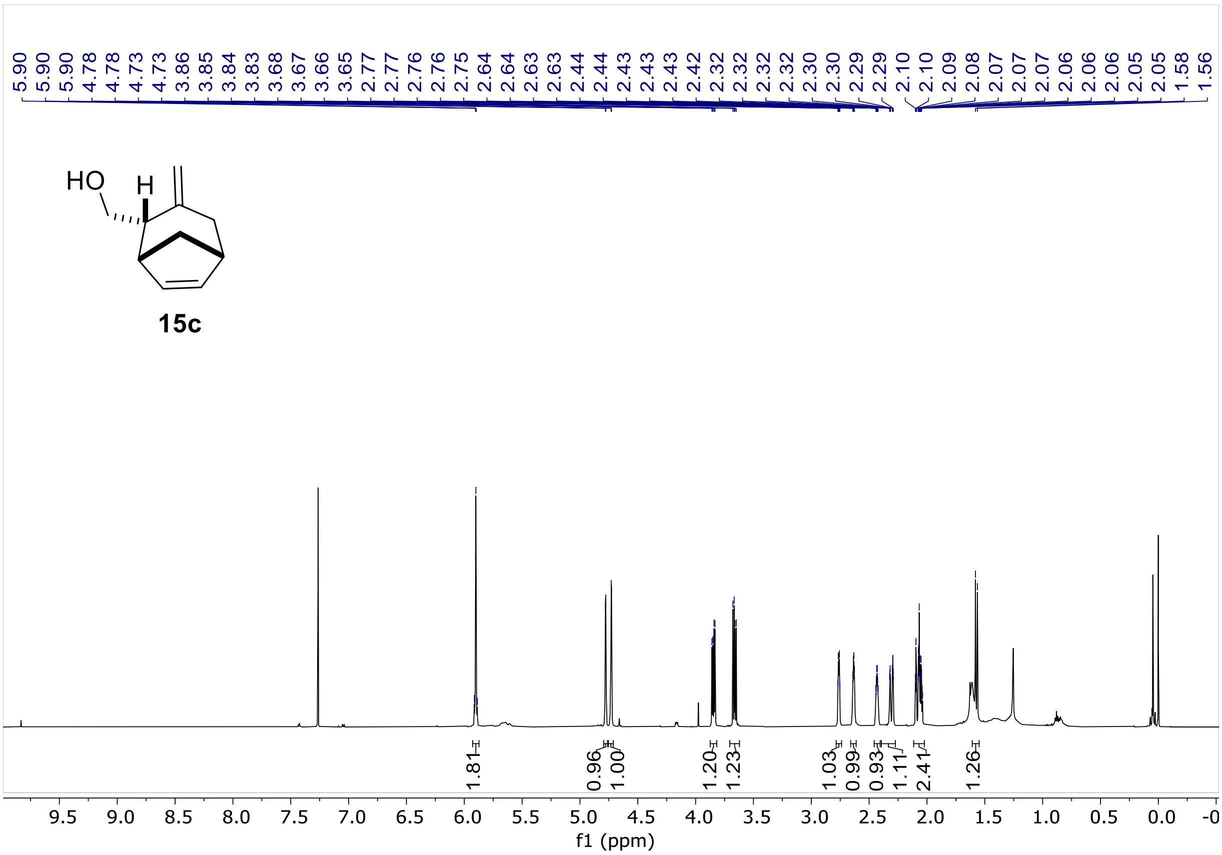

$^{13}C\{^{1}H\}$ NMR (150 MHz, $CDCl_3$): **15c**

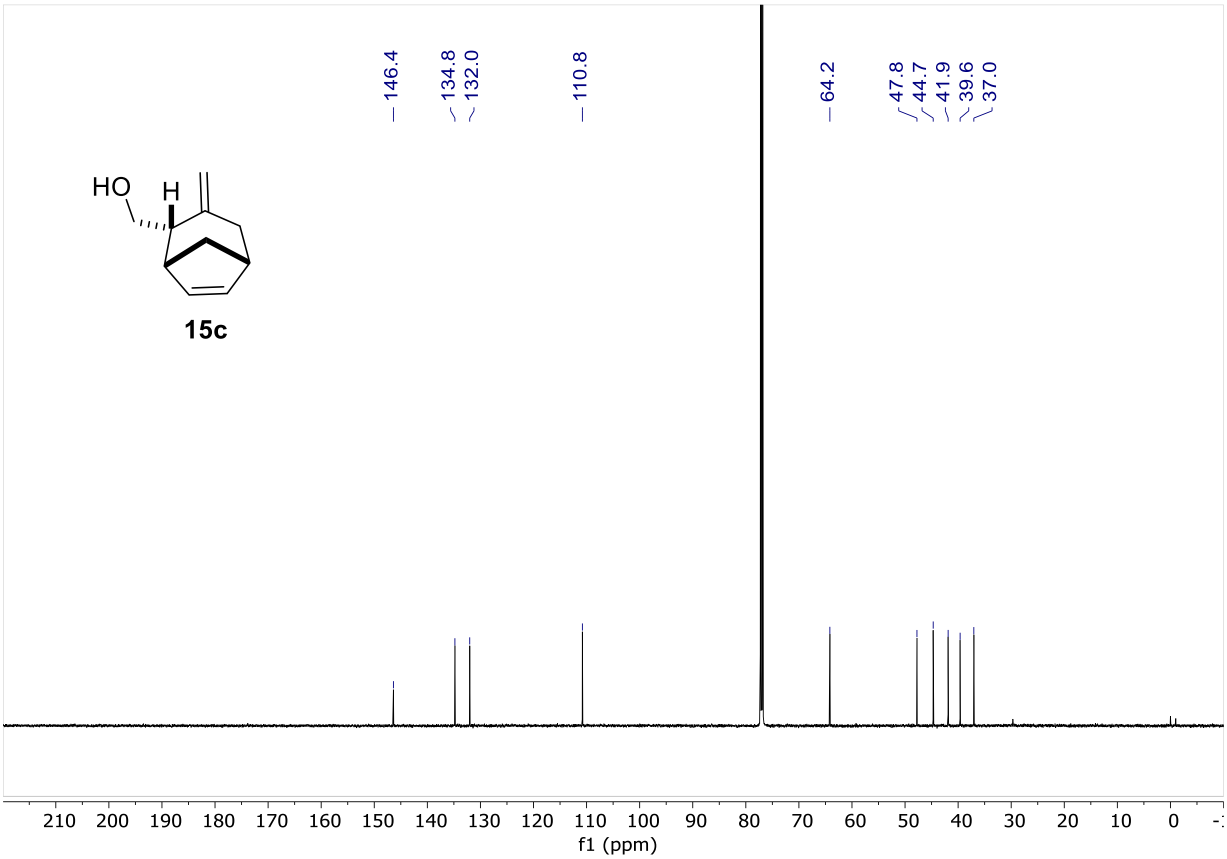

^{1}H NMR (600 MHz, $CDCl_3$): **16c**

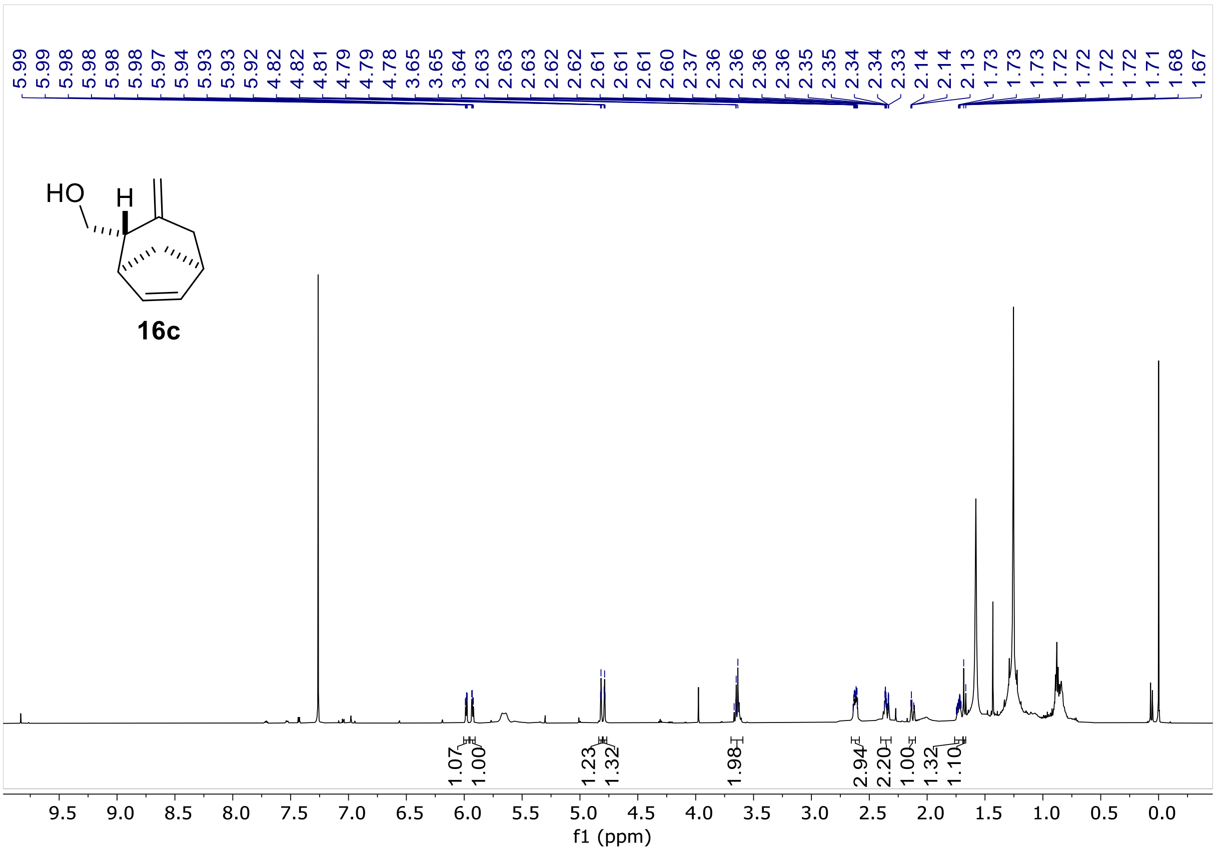

$^{13}C\{^{1}H\}$ NMR (150 MHz, $CDCl_3$): **16c**

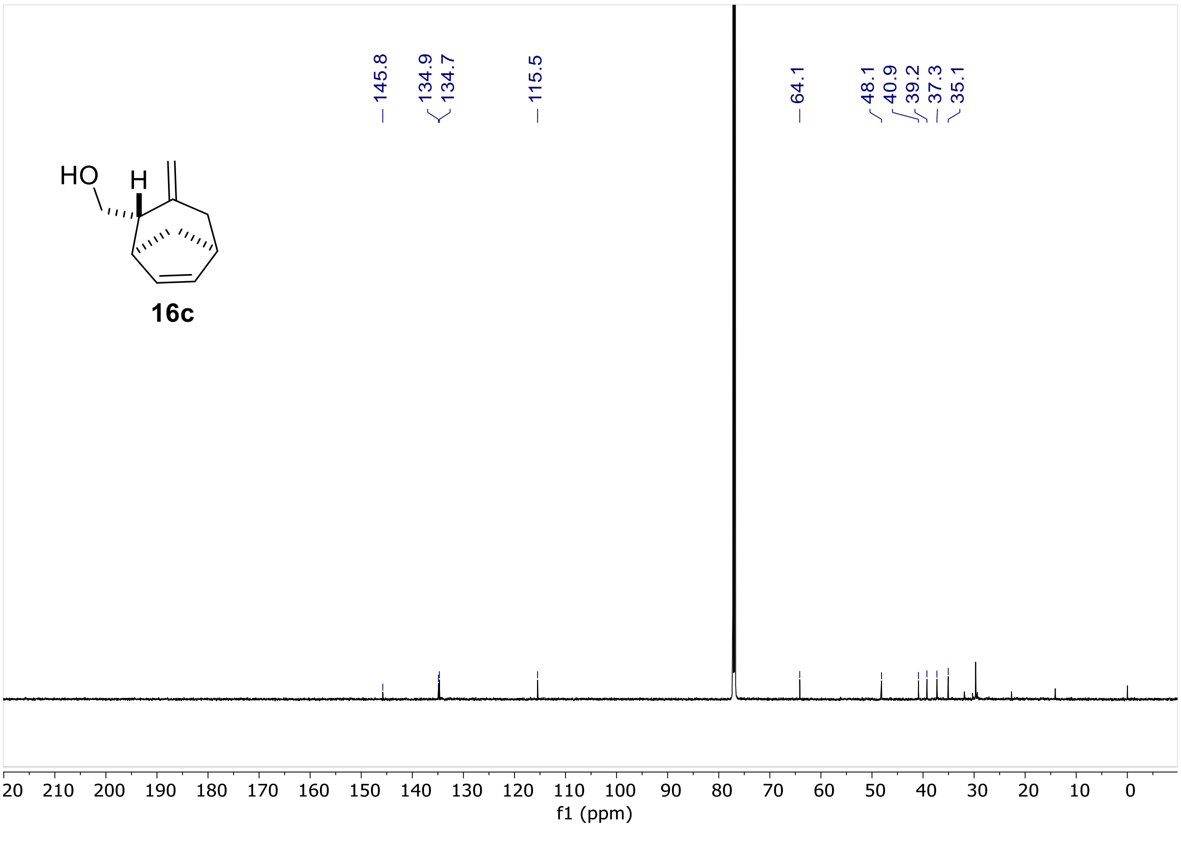

^{1}H NMR (500 MHz, $CDCl_3$): **21c**

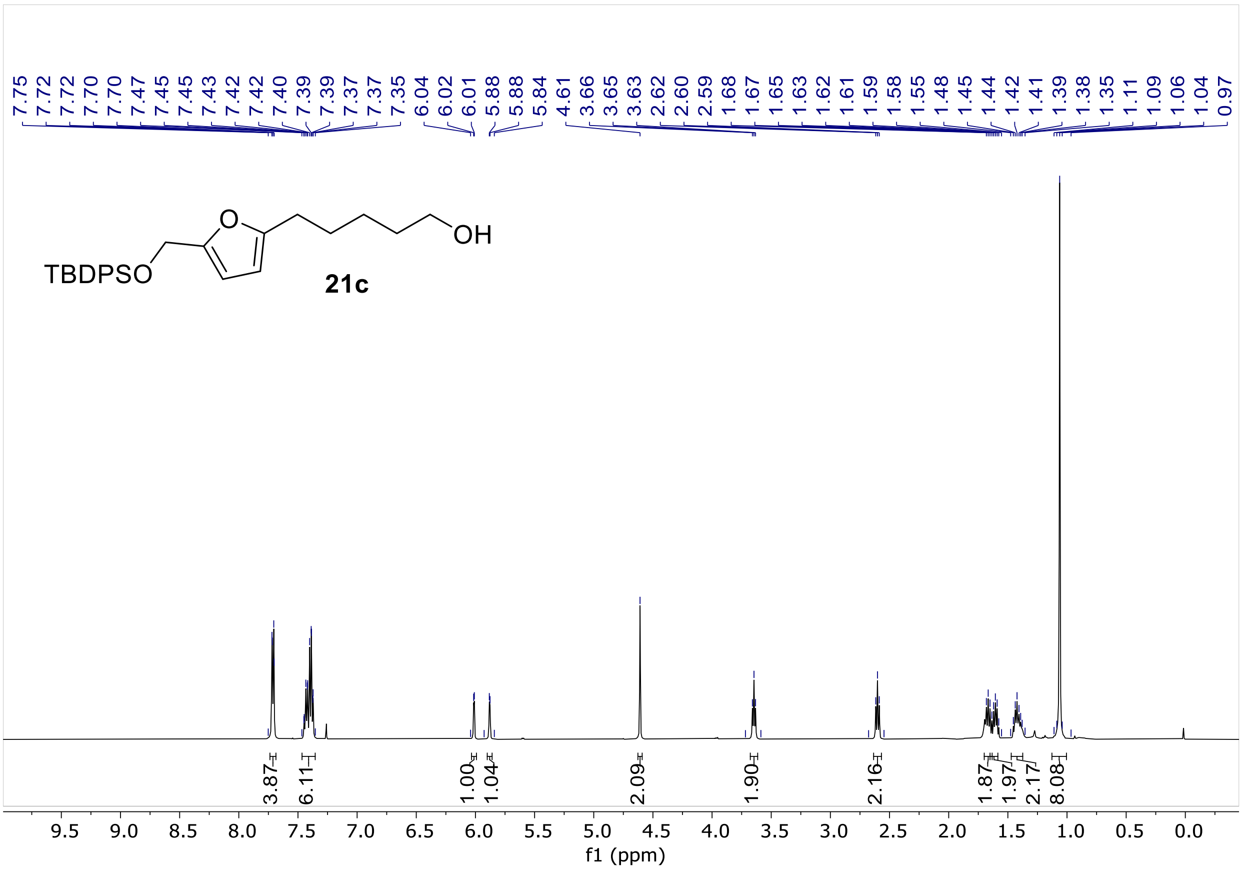

$^{13}C\{^{1}H\}$ NMR (100 MHz, $CDCl_3$): **21c**

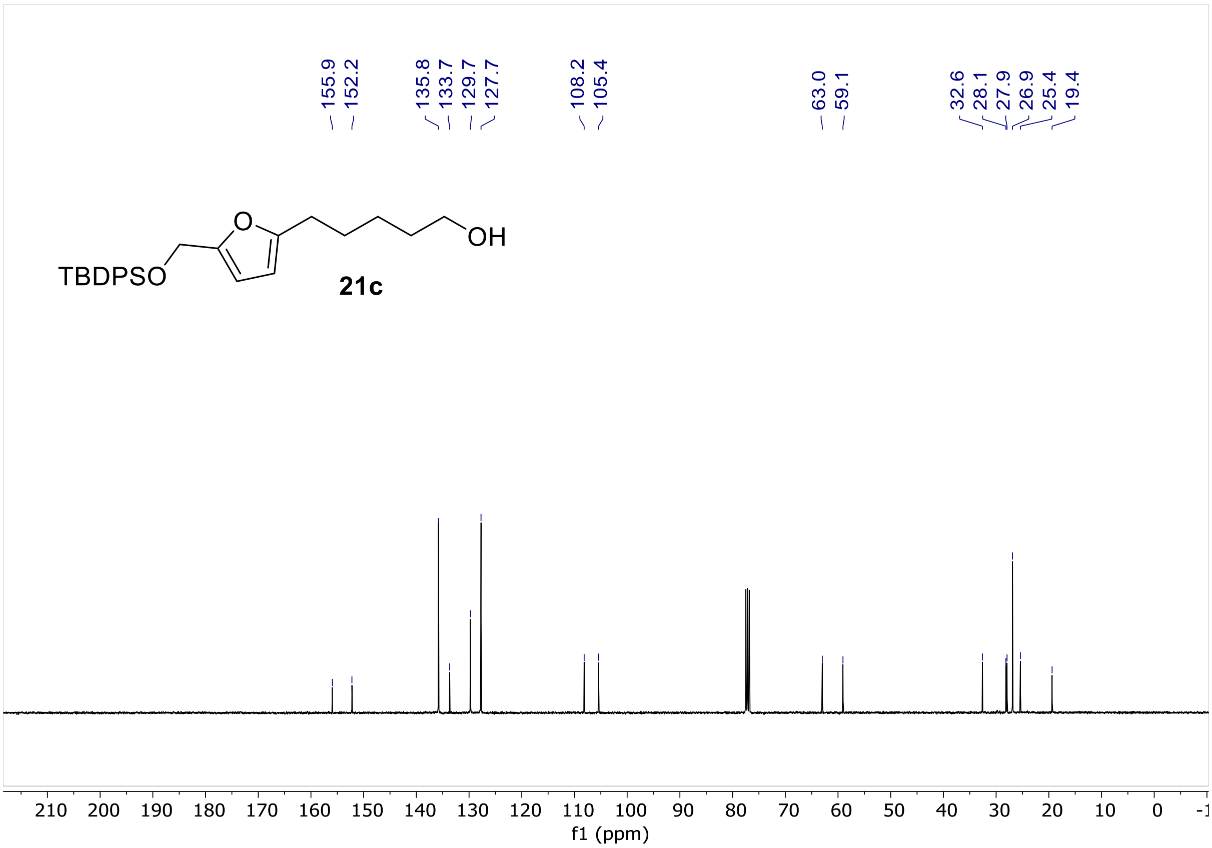

^{1}H NMR (400 MHz, $CDCl_3$): **21d**

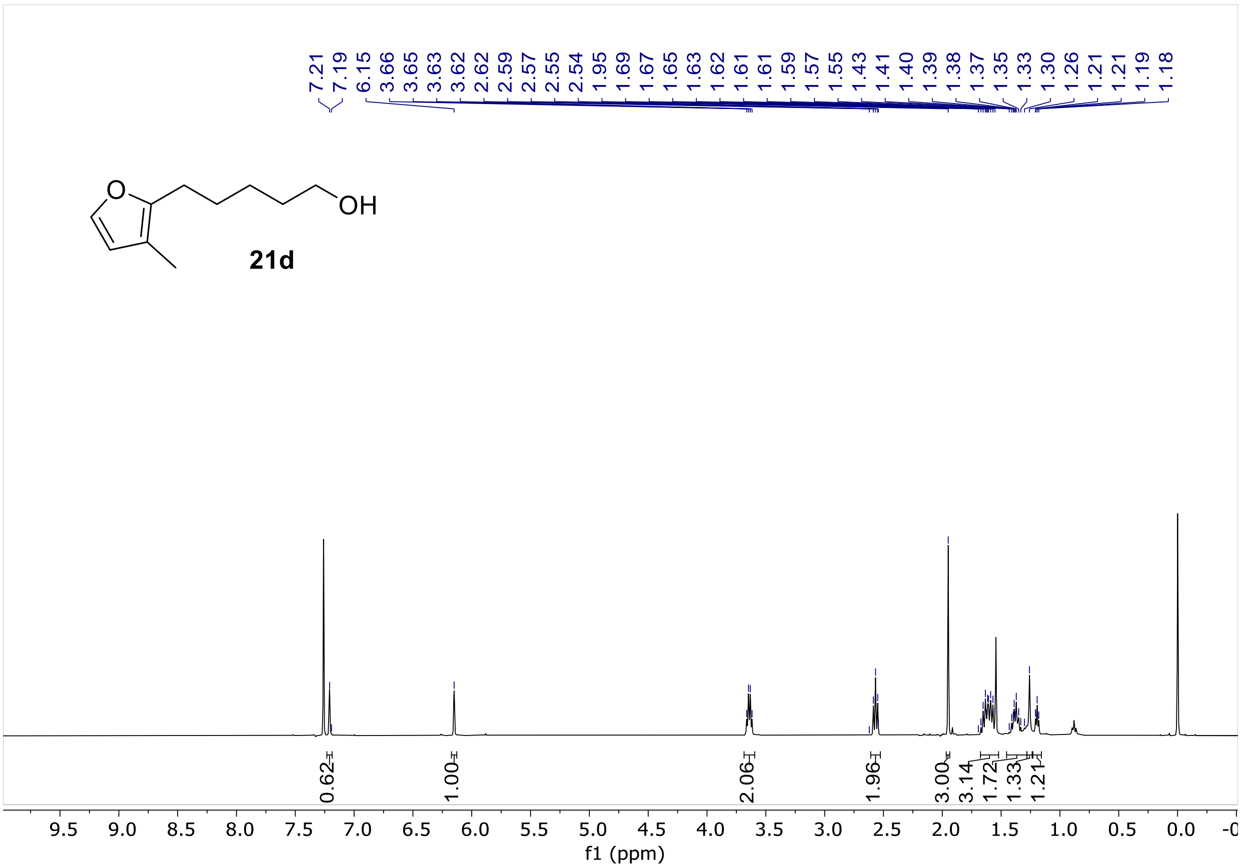

^{13}C{^{1}H} NMR (125 MHz, $CDCl_3$): **21d**

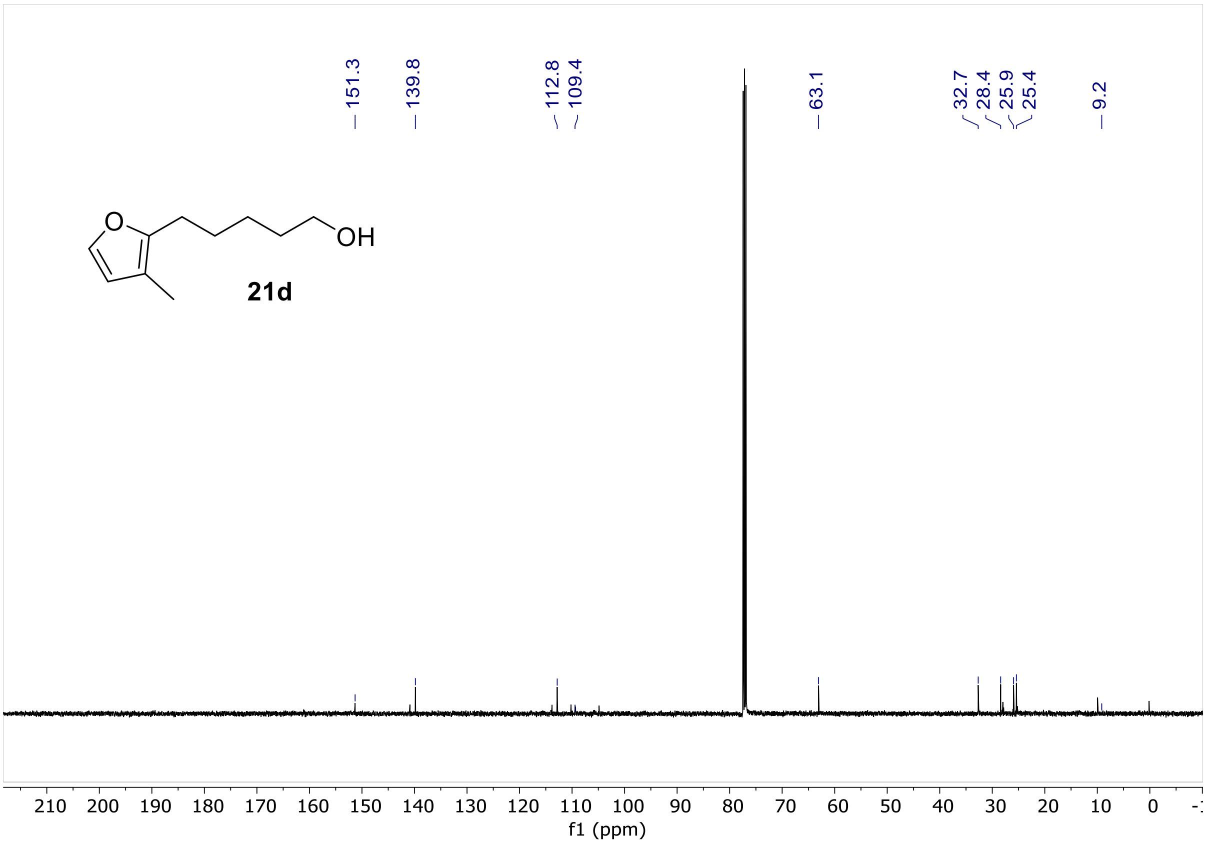

^{1}H NMR (500 MHz, $CDCl_3$): **21g**

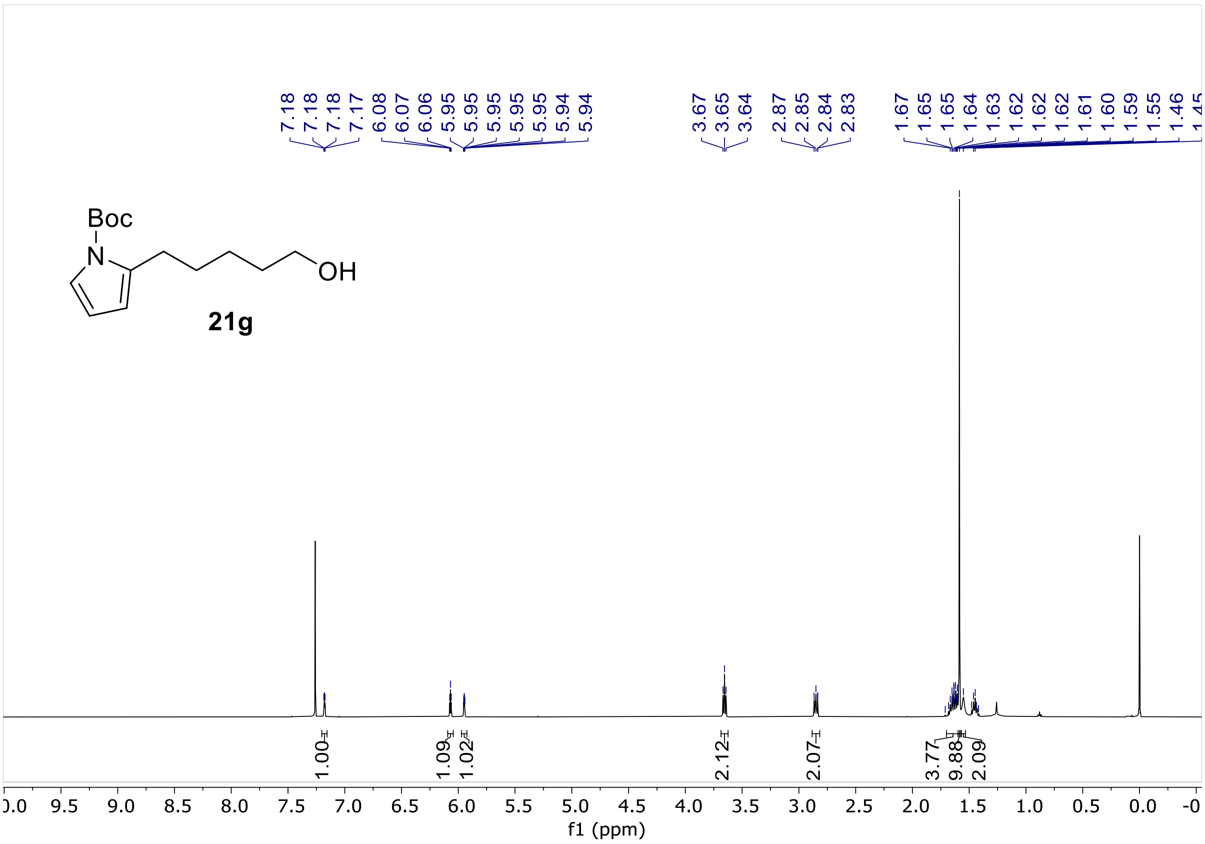

$^{13}C\{^1H\}$ NMR (100 MHz, $CDCl_3$): **21g**

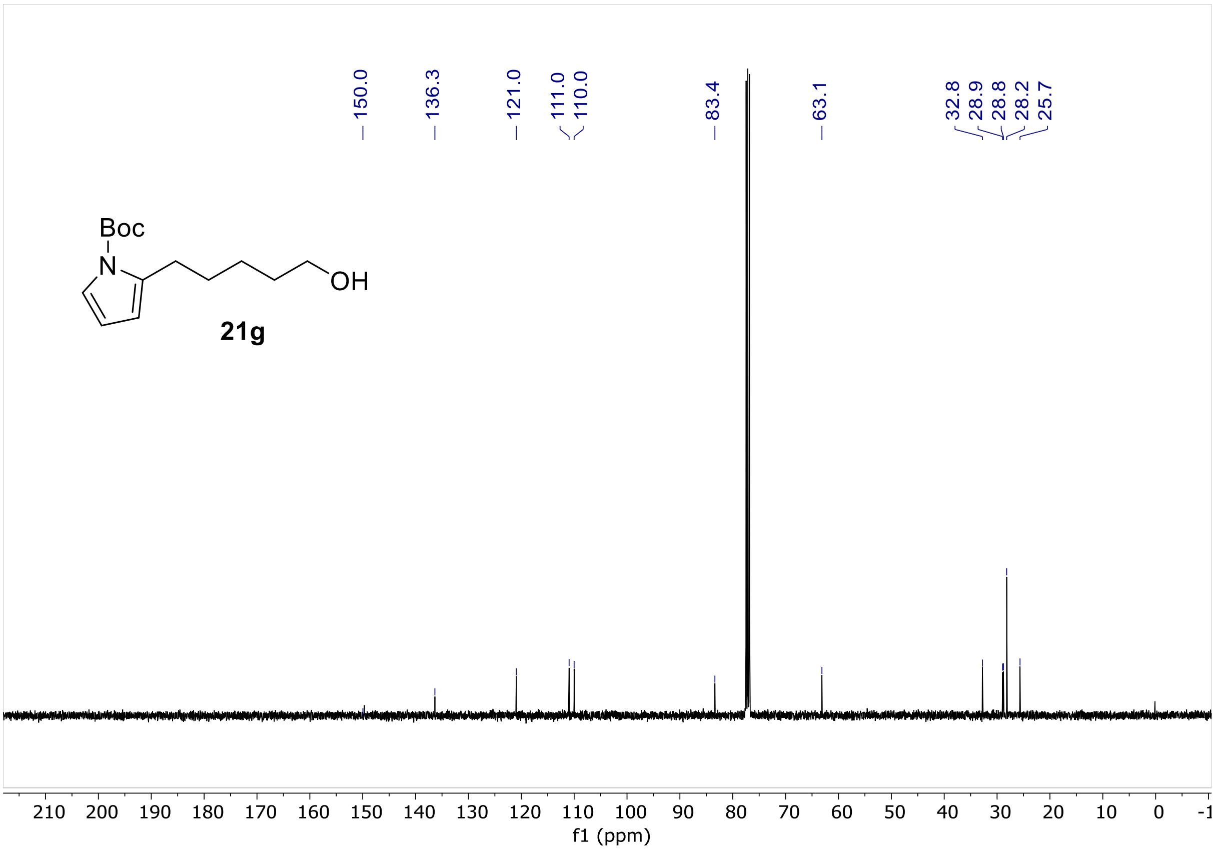

^{1}H NMR (500 MHz, $CDCl_3$): **21h**

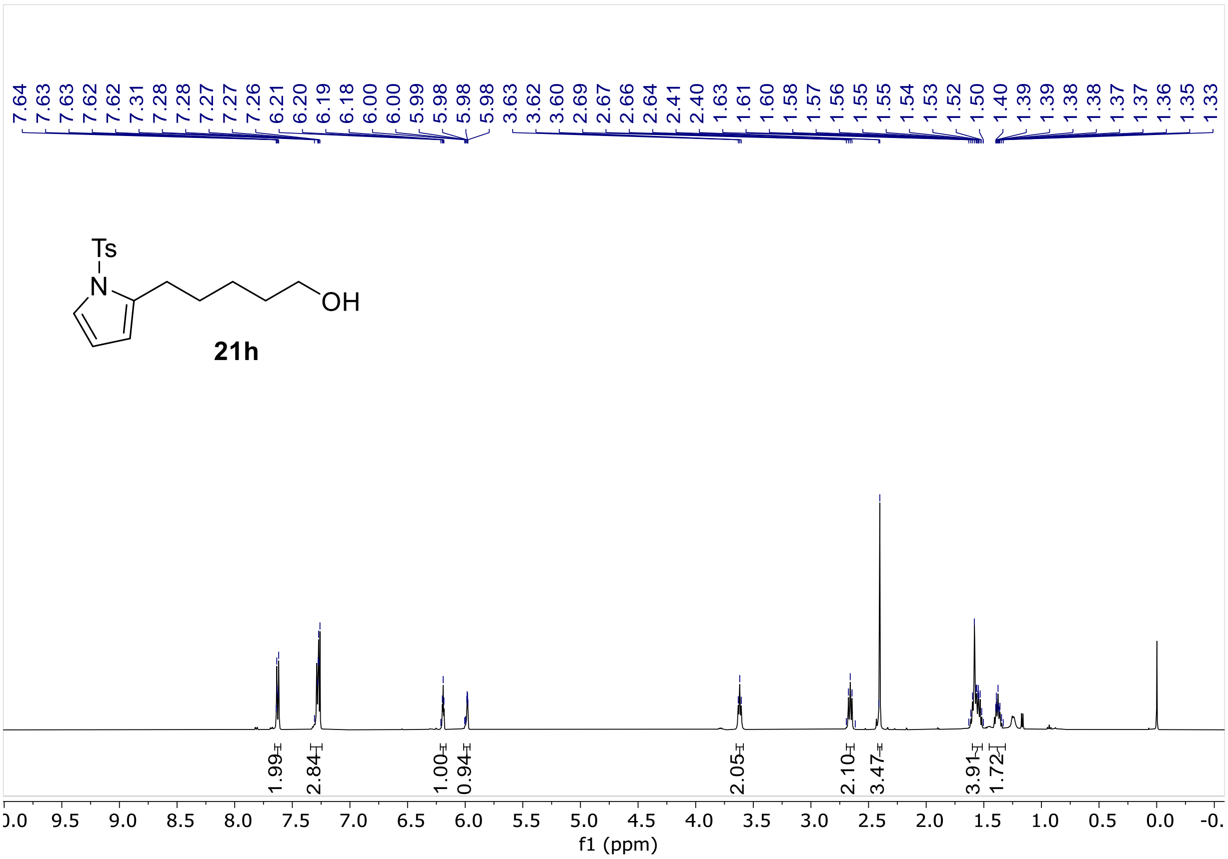

$^{13}C\{^{1}H\}$ NMR (100 MHz, $CDCl_3$): **21h**

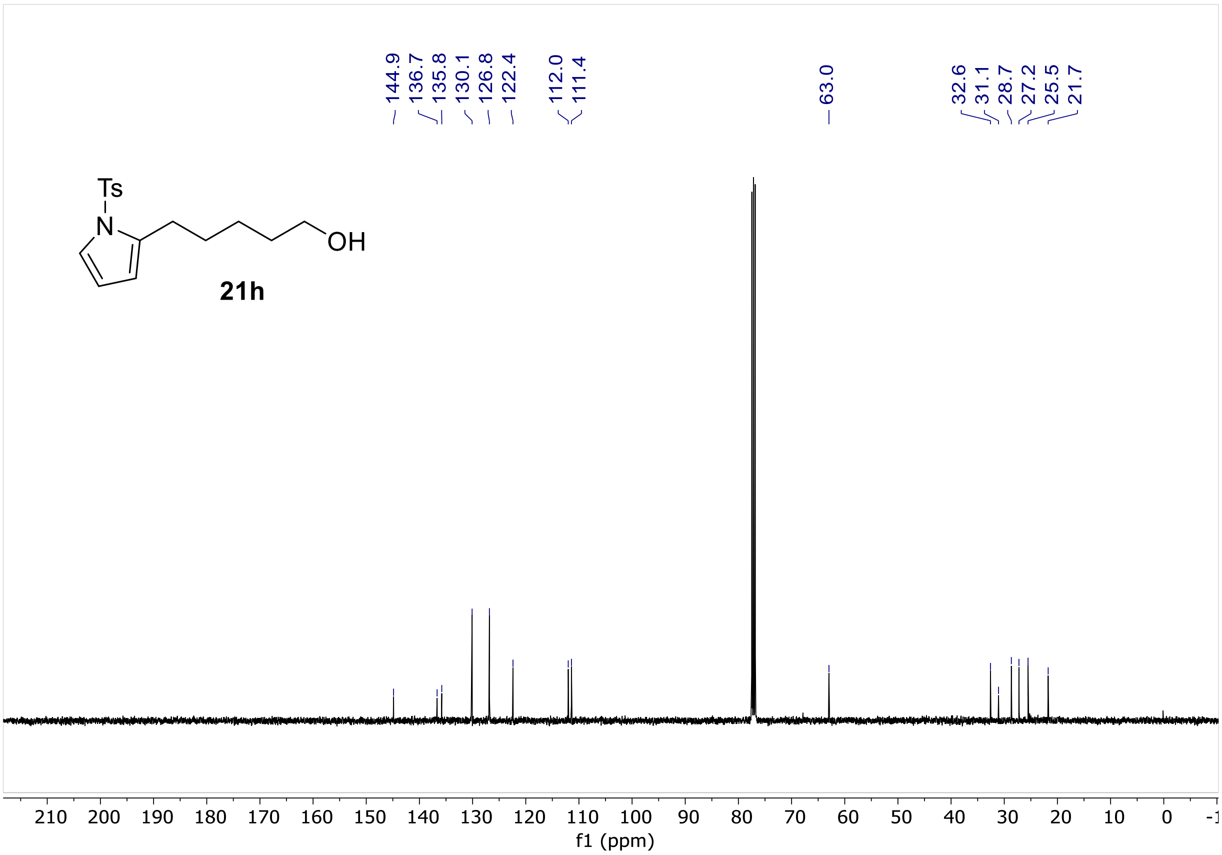

^{1}H NMR (400 MHz, $CDCl_3$): **21j**

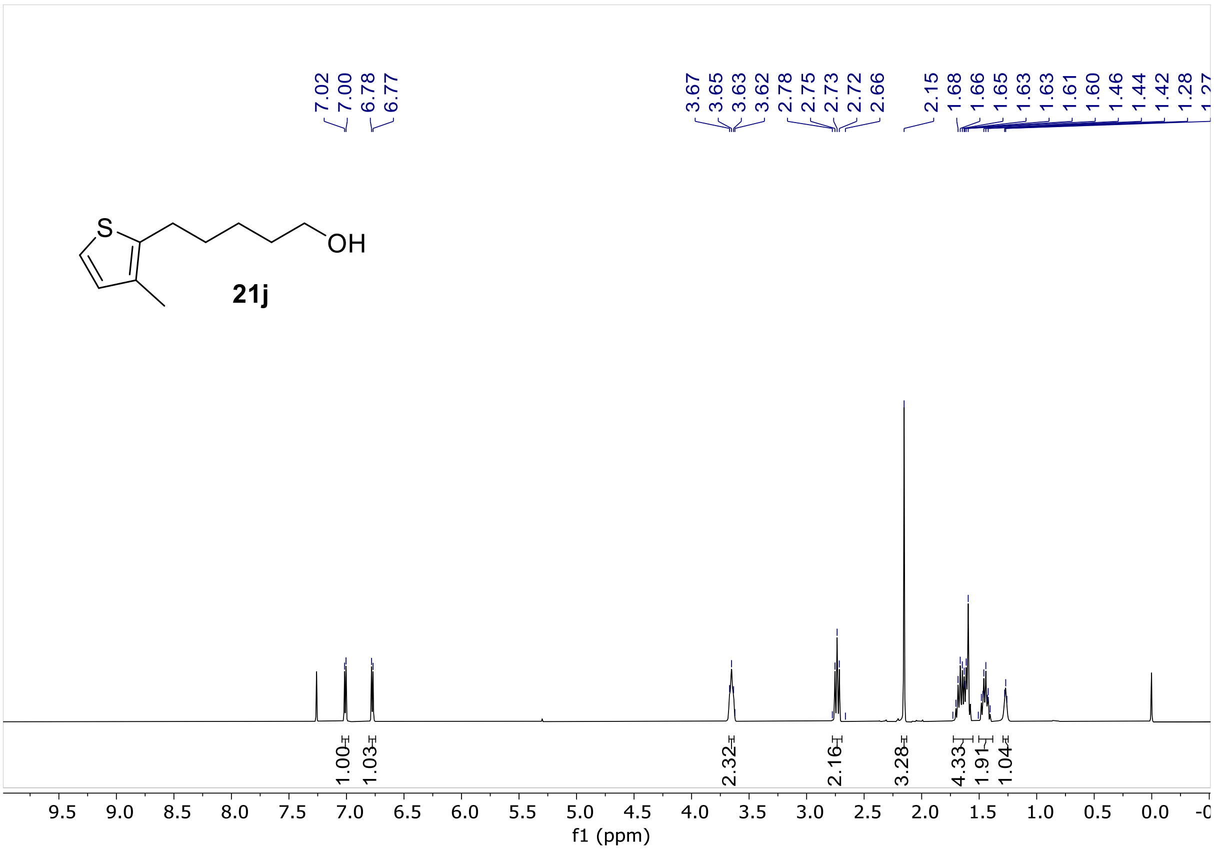

^{13}C{^{1}H} NMR (100 MHz, $CDCl_3$): **21j**

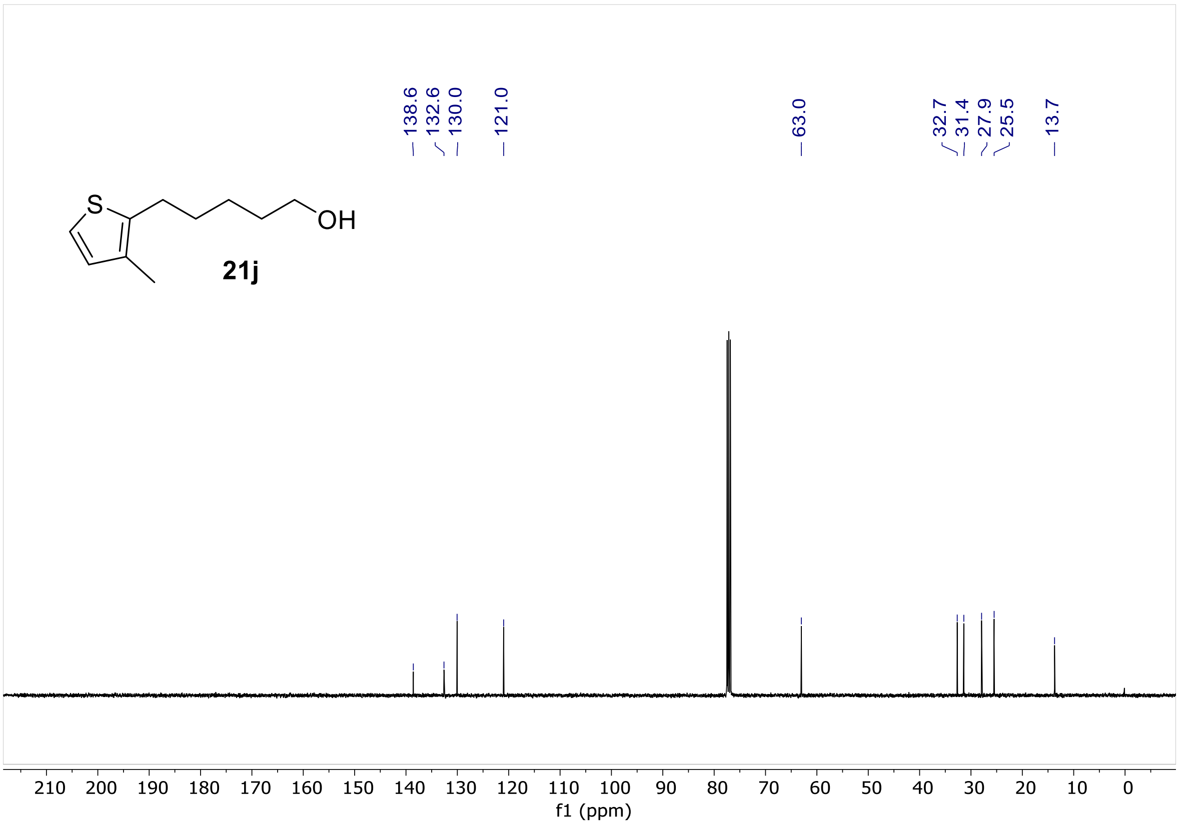

^{1}H NMR (400 MHz, $CDCl_3$): **21k**

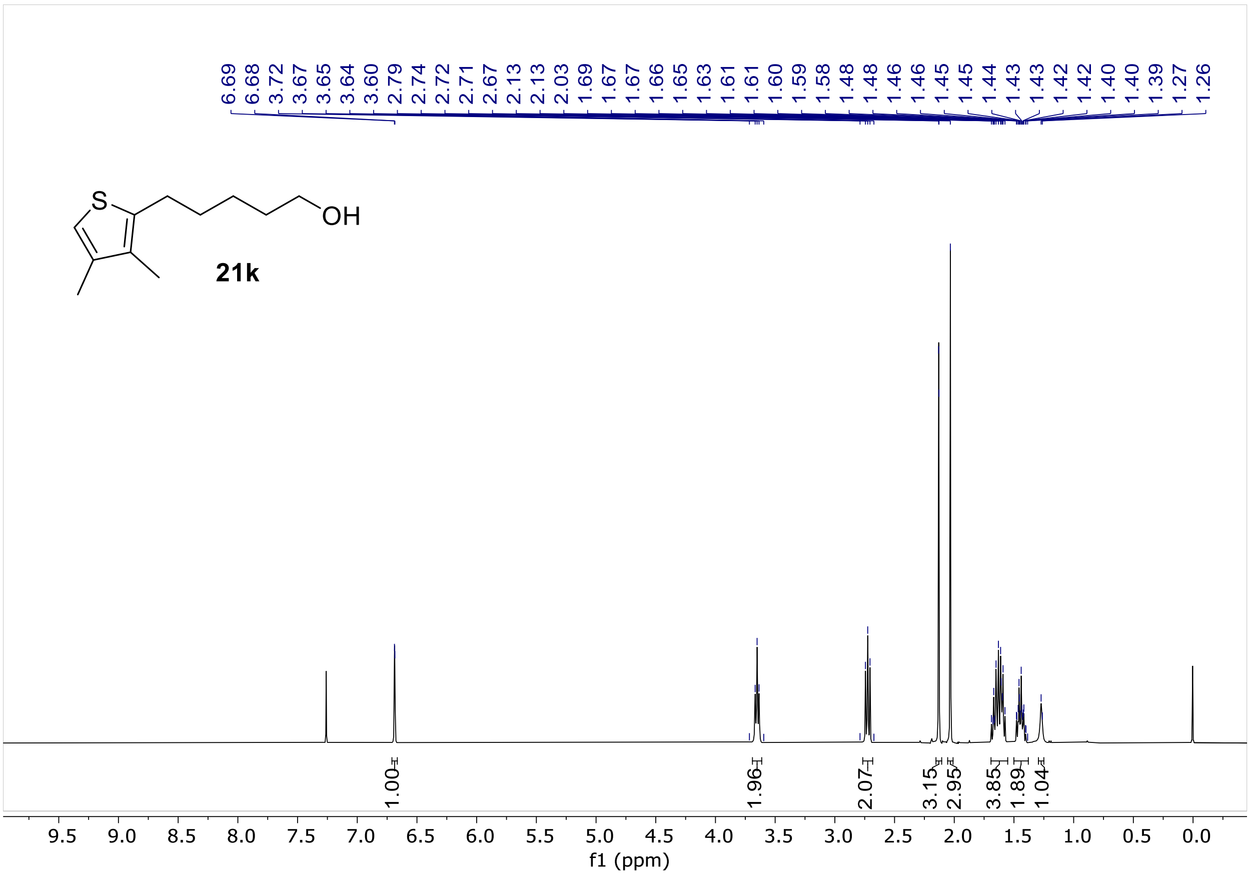

$^{13}C\{^{1}H\}$ NMR (100 MHz, $CDCl_3$): **21k**

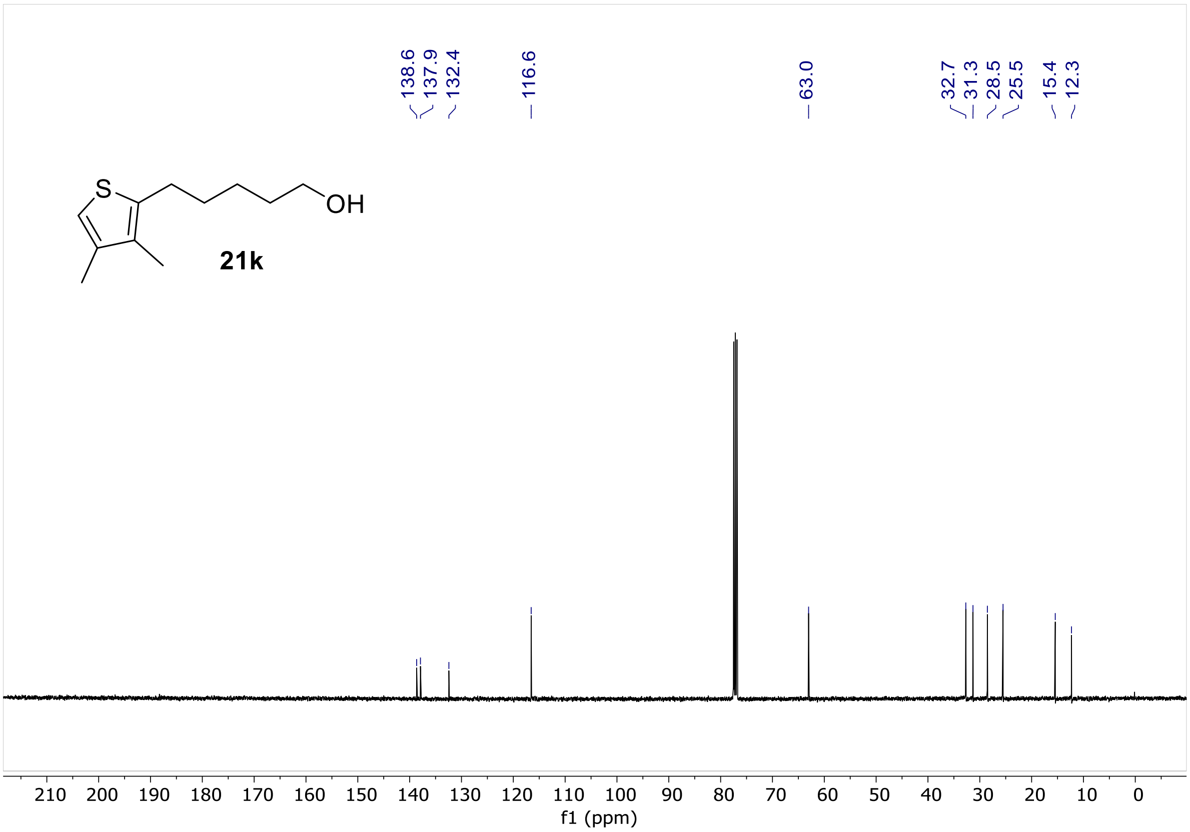

^{1}H NMR (500 MHz, $CDCl_3$): **22c**

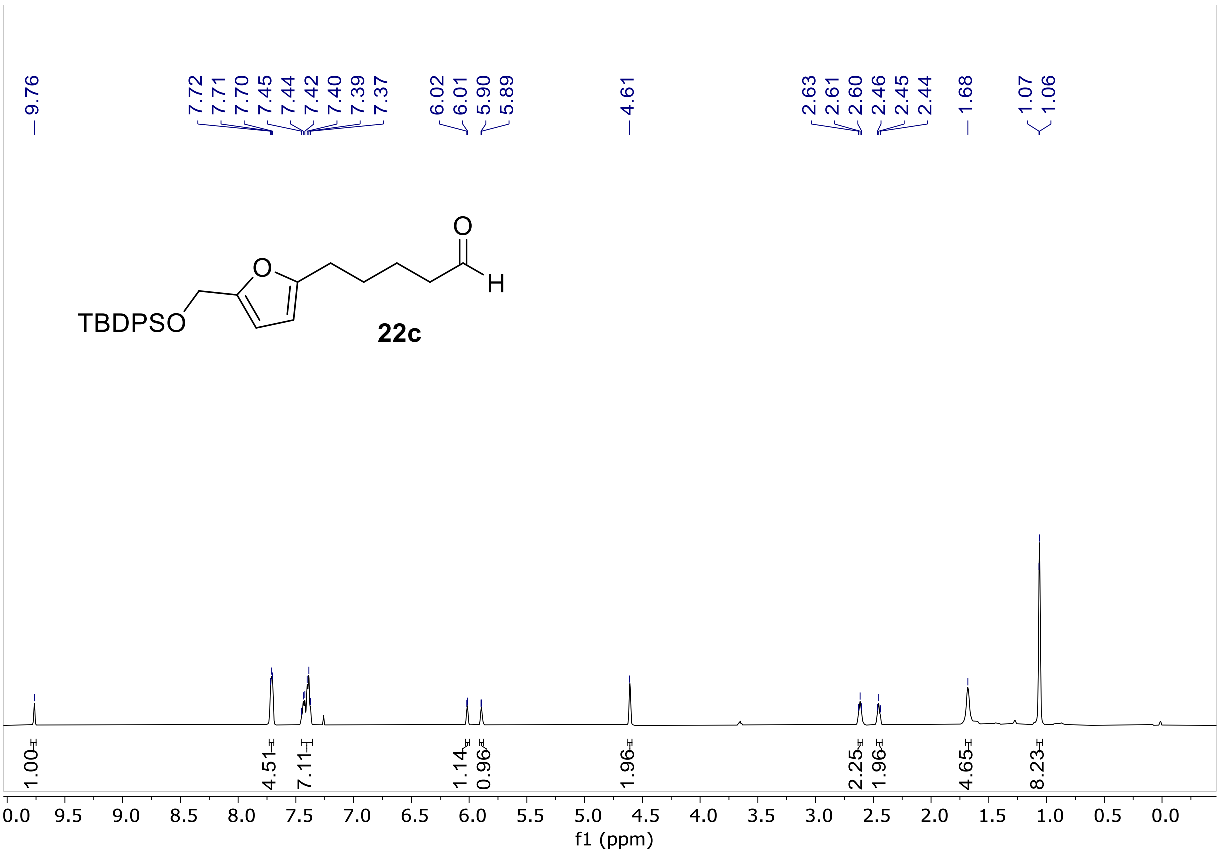

$^{13}C\{^1H\}$ NMR (100 MHz, $CDCl_3$): **22c**

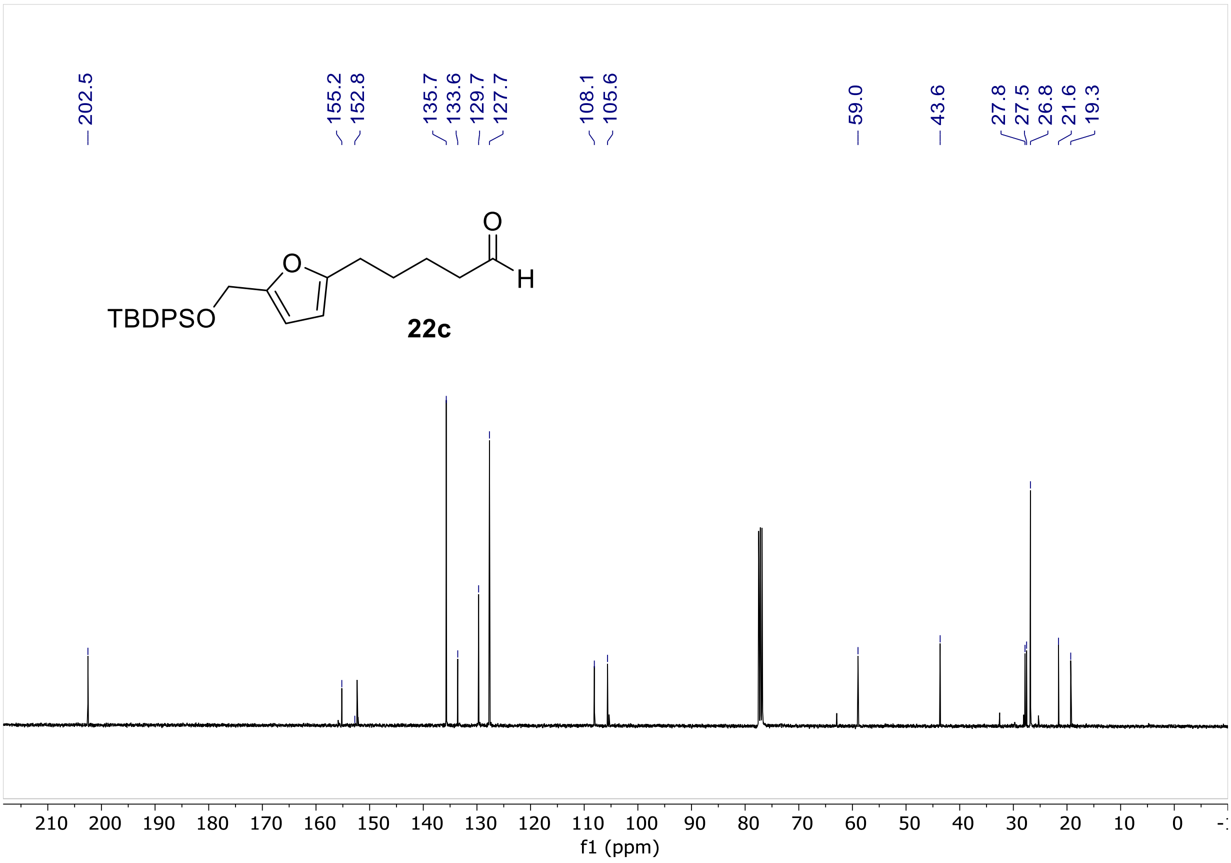

^{1}H NMR (500 MHz, $CDCl_3$): **22d**

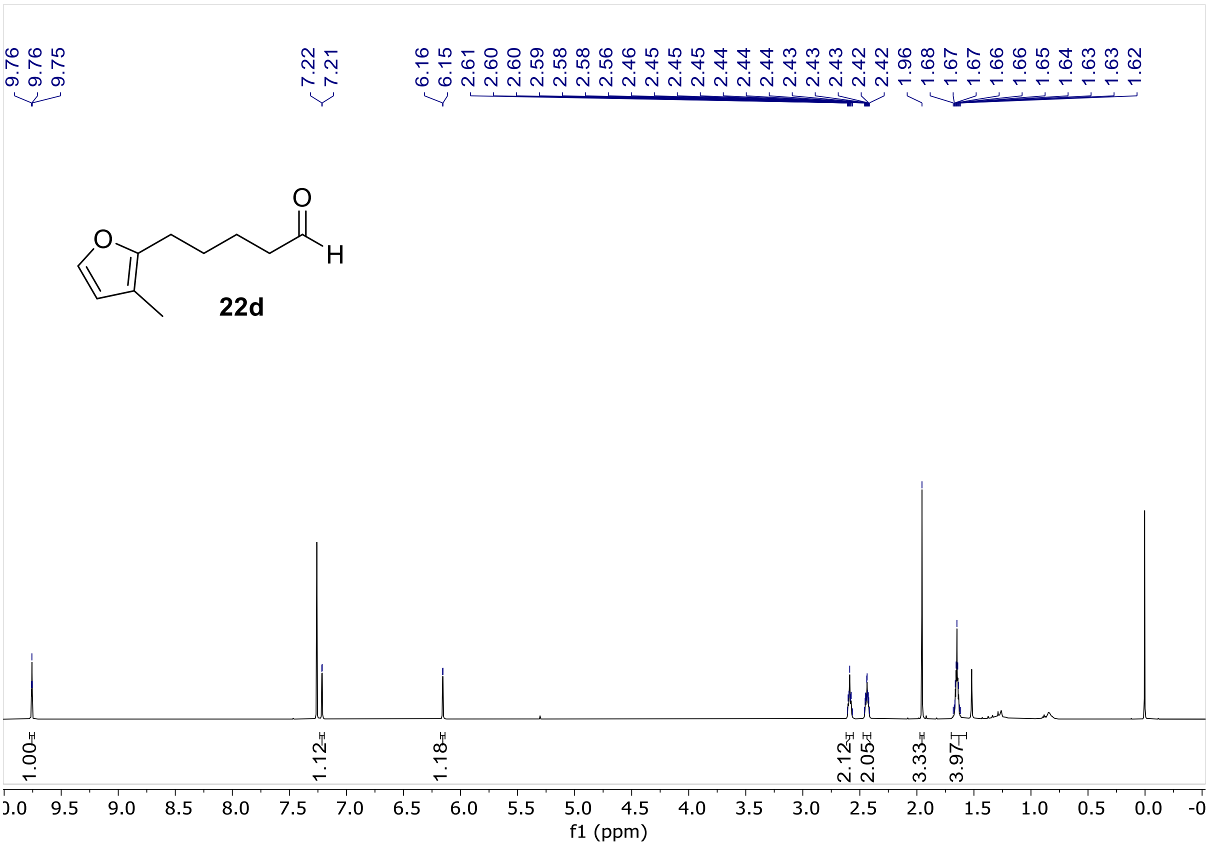

$^{13}C\{^{1}H\}$ NMR (100 MHz, $CDCl_3$): **22d**

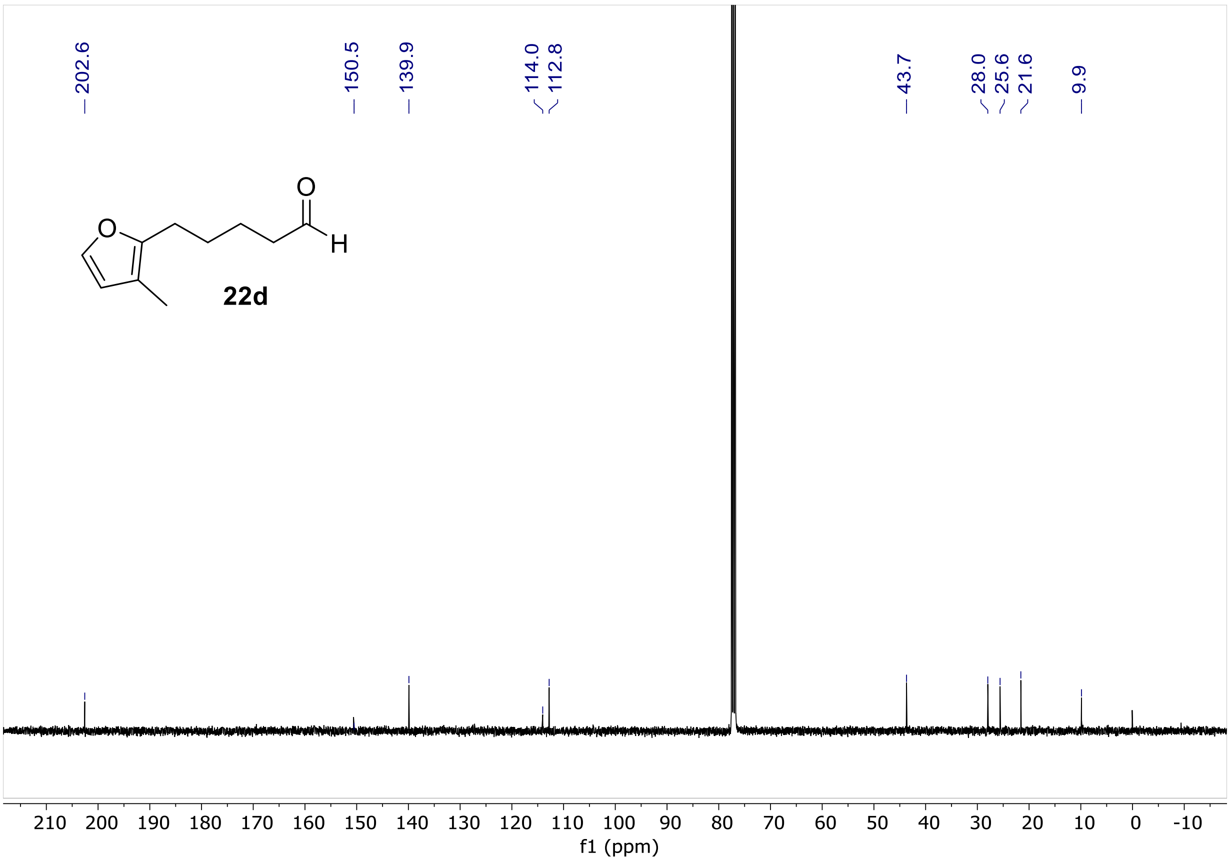

^{1}H NMR (500 MHz, $CDCl_3$): **22g**

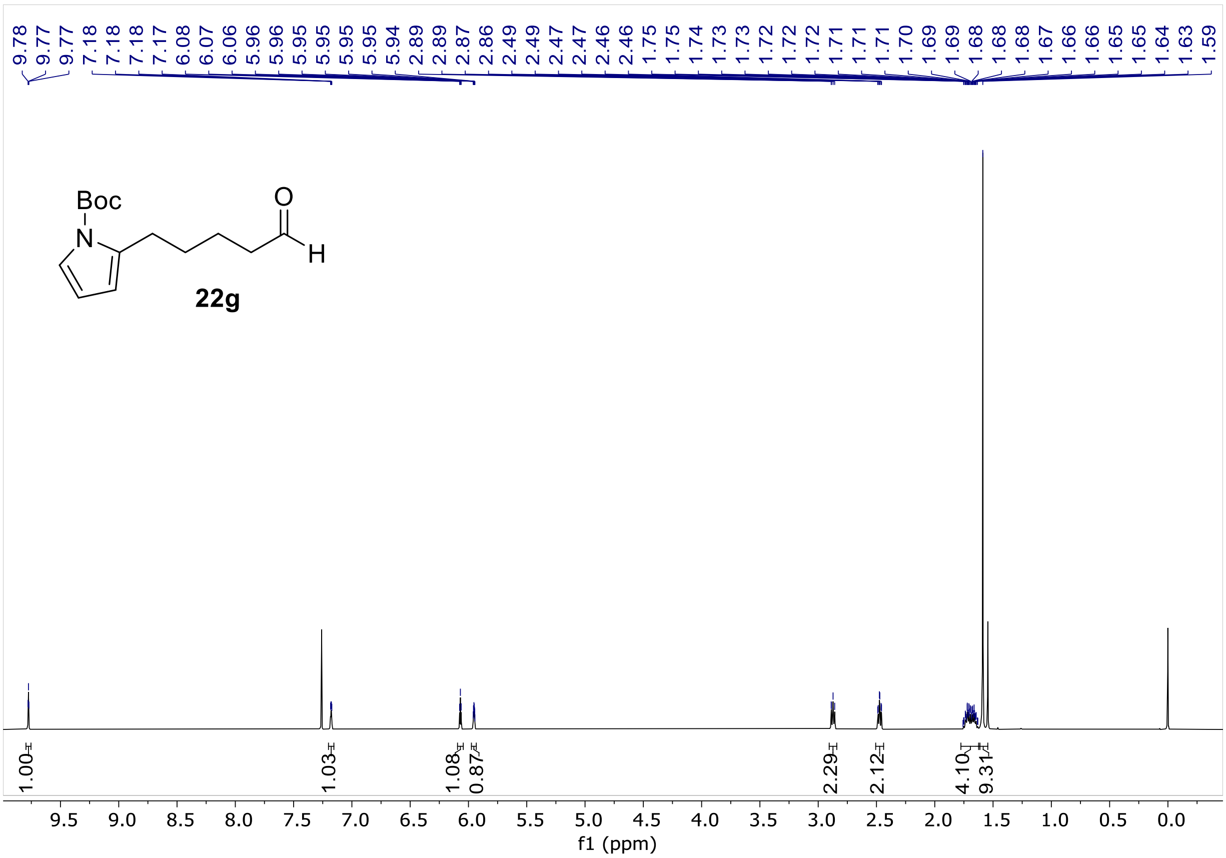

$^{13}C\{^{1}H\}$ NMR (100 MHz, $CDCl_3$): **22g**

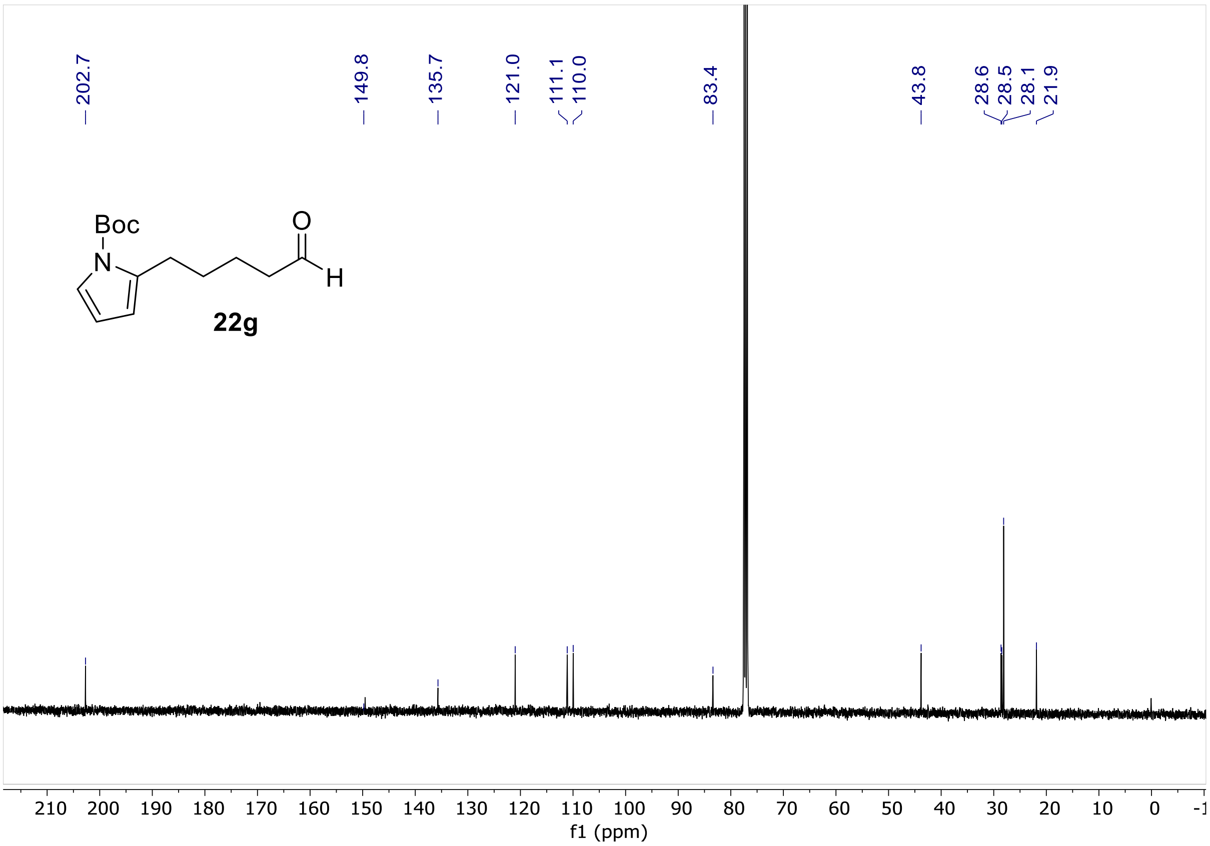

^{1}H NMR (400 MHz, $CDCl_3$): **22h**

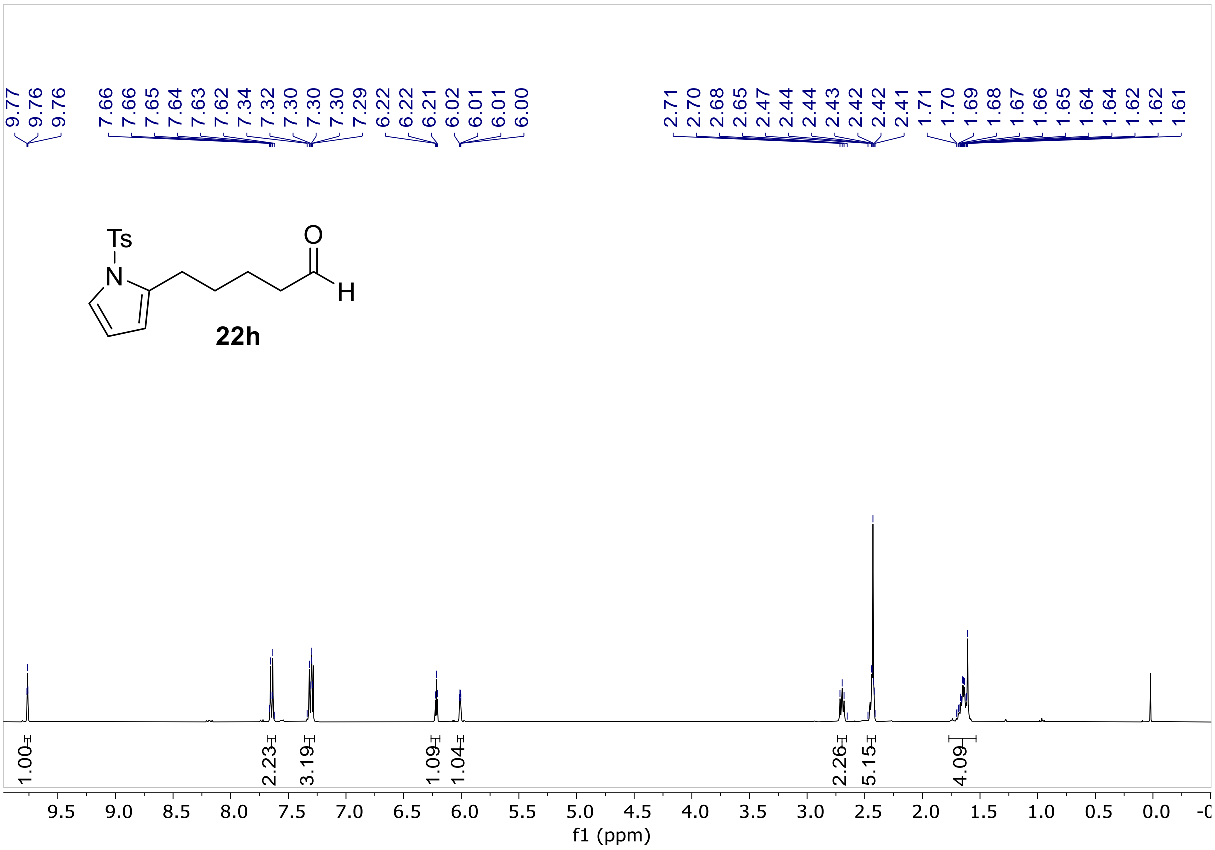

$^{13}C\{^1H\}$ NMR (100 MHz, $CDCl_3$): **22h**

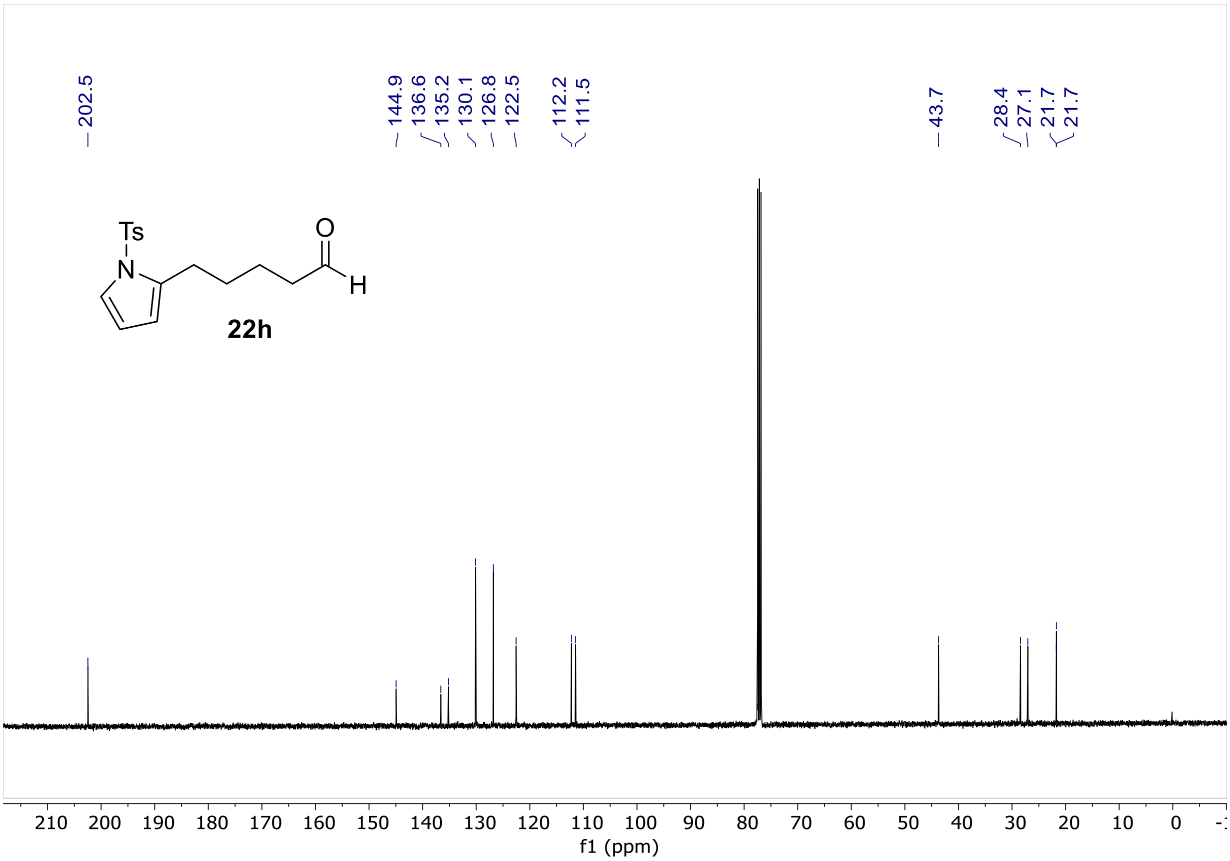

^{1}H NMR (500 MHz, $CDCl_3$): **22j**

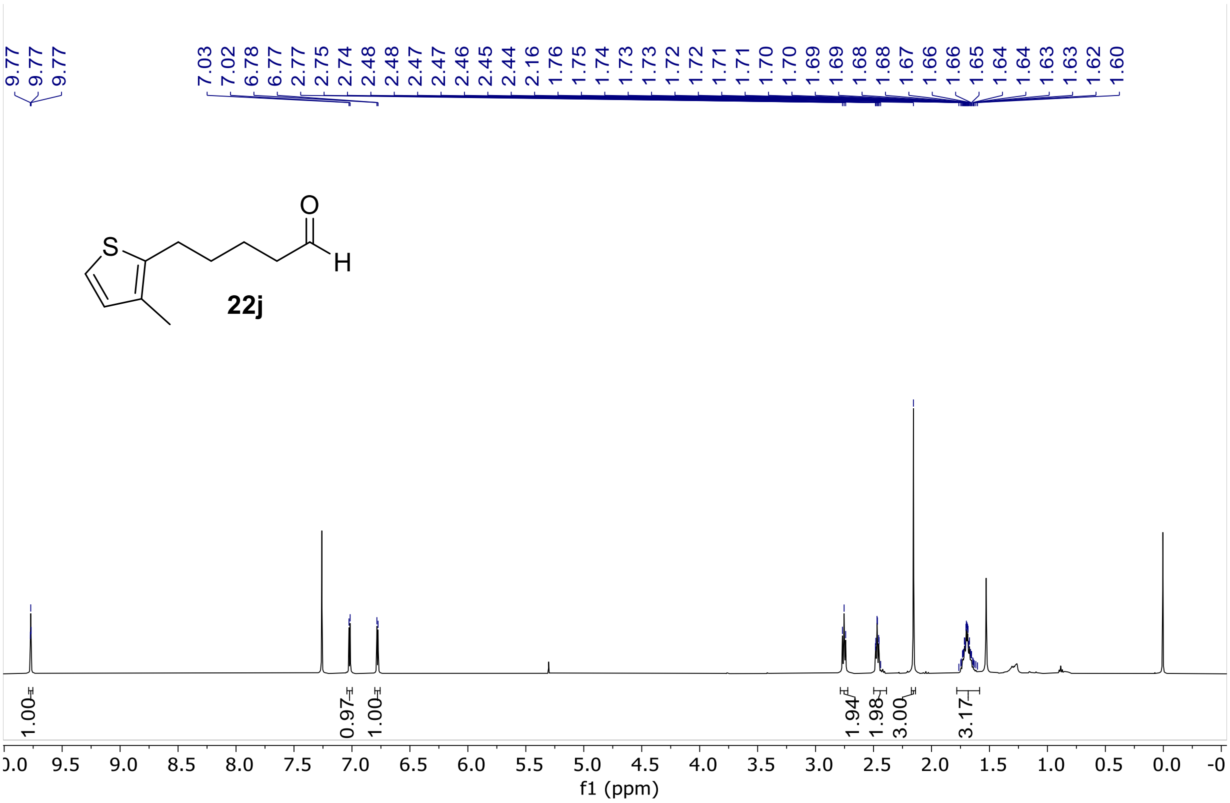

$^{13}C\{^{1}H\}$ NMR (100 MHz, $CDCl_3$): **22j**

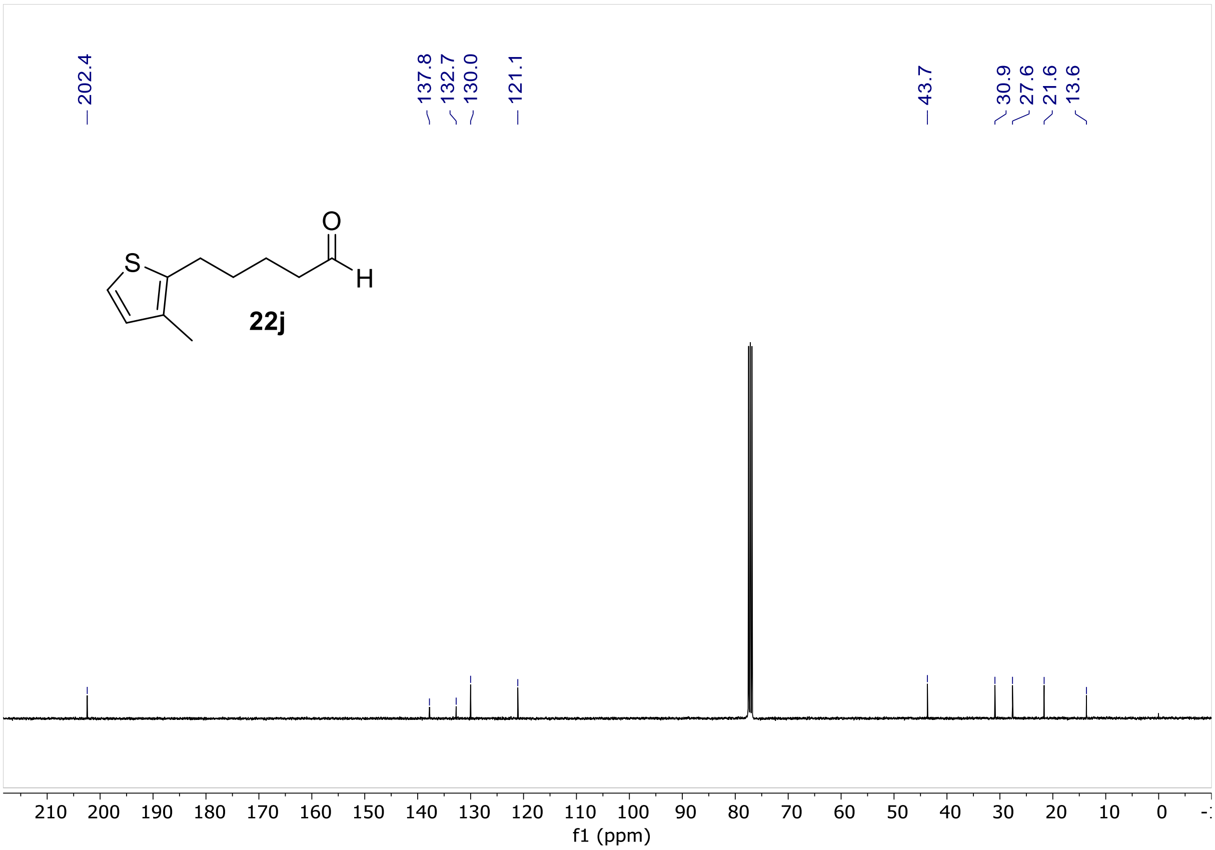

^{1}H NMR (500 MHz, $CDCl_3$): **22k**

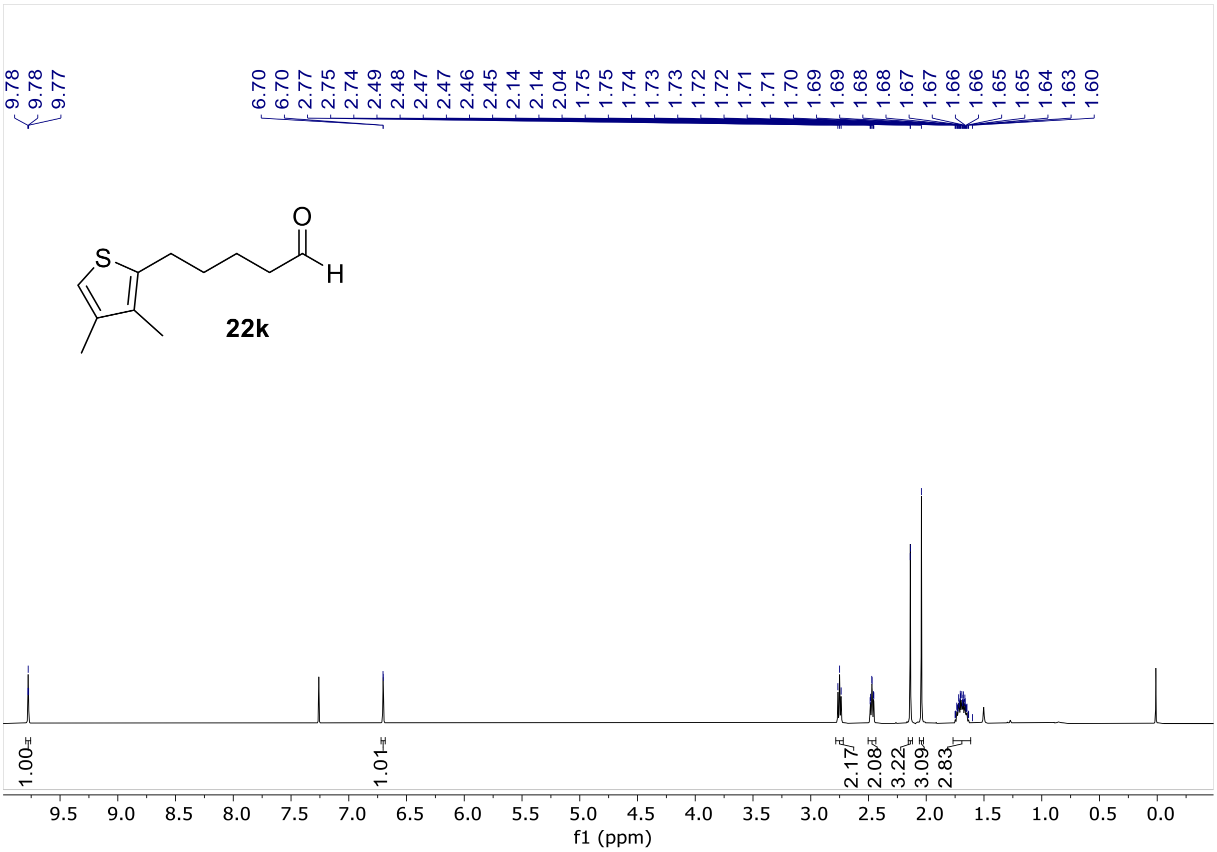

$^{13}C\{^1H\}$ NMR (100 MHz, $CDCl_3$): **22k**

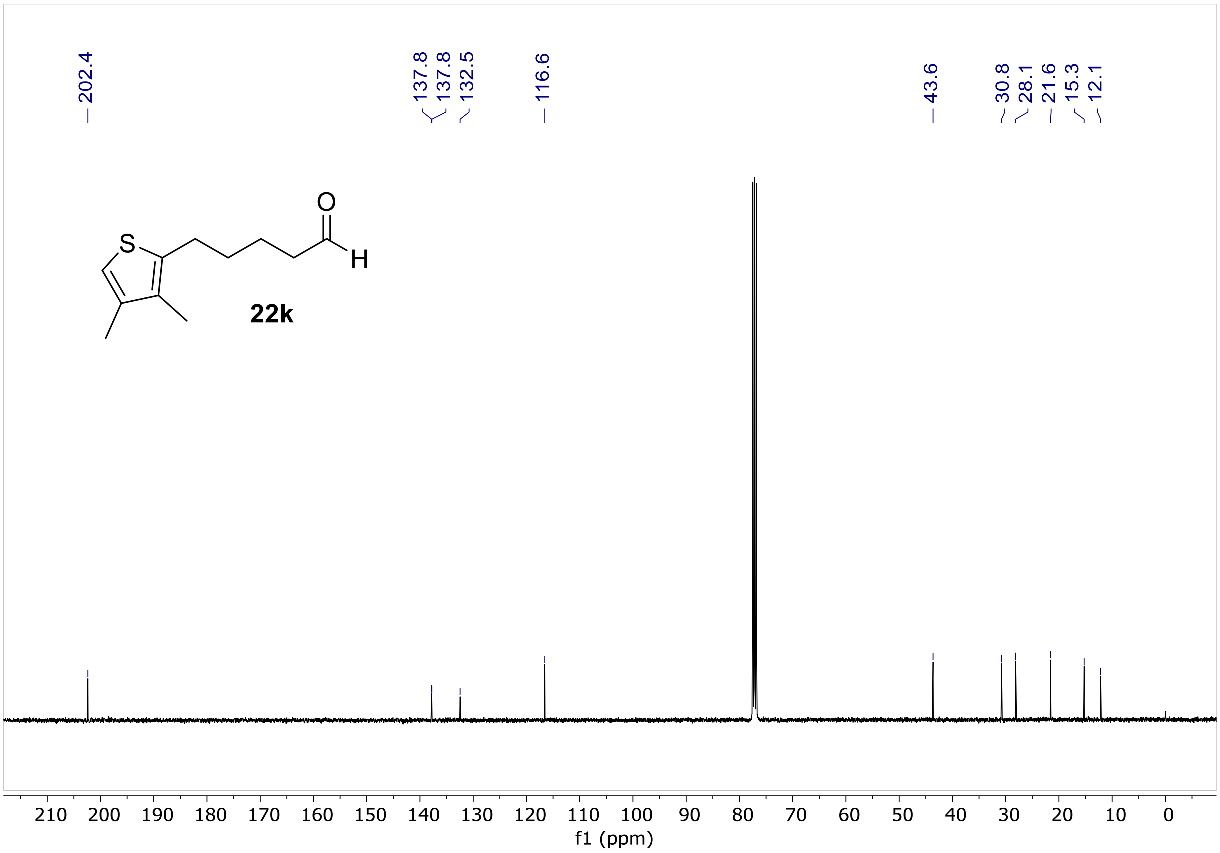

^{1}H NMR (400 MHz, $CDCl_3$): **23a**

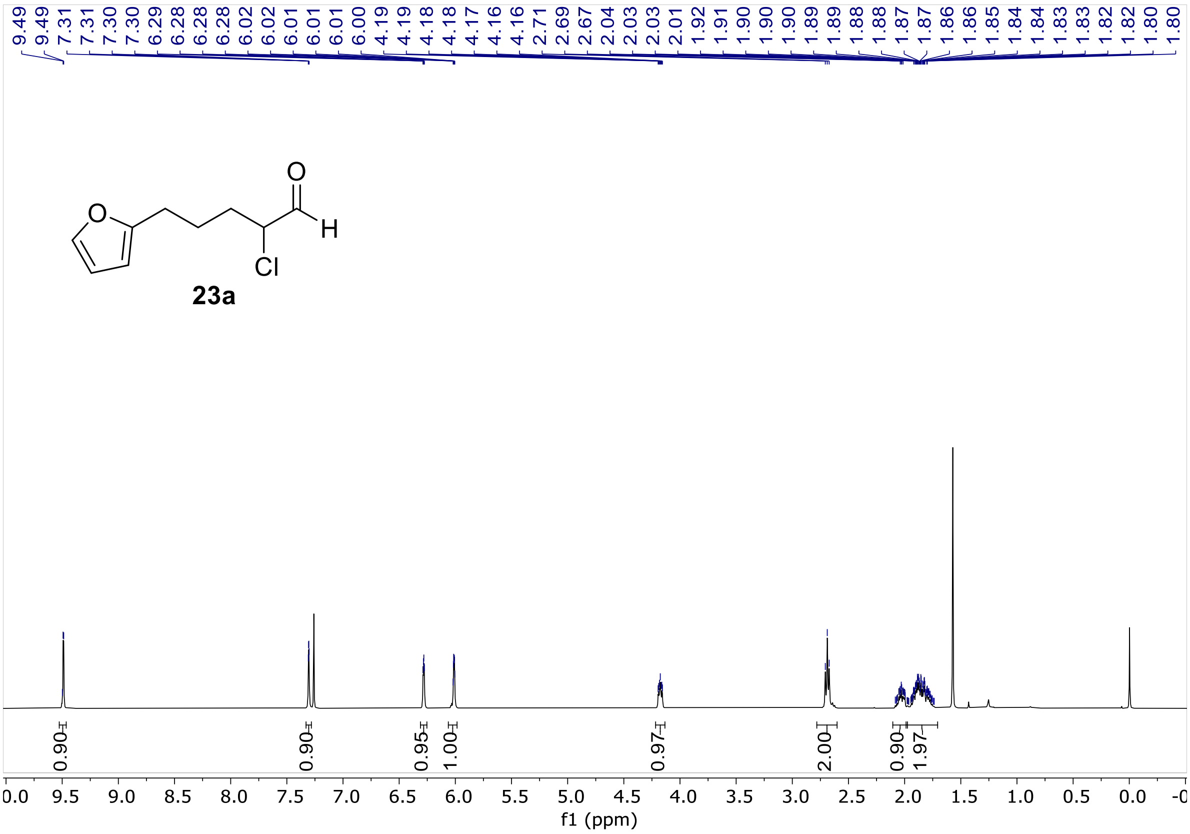

^{13}C{^{1}H} NMR (100 MHz, $CDCl_3$): **23a**

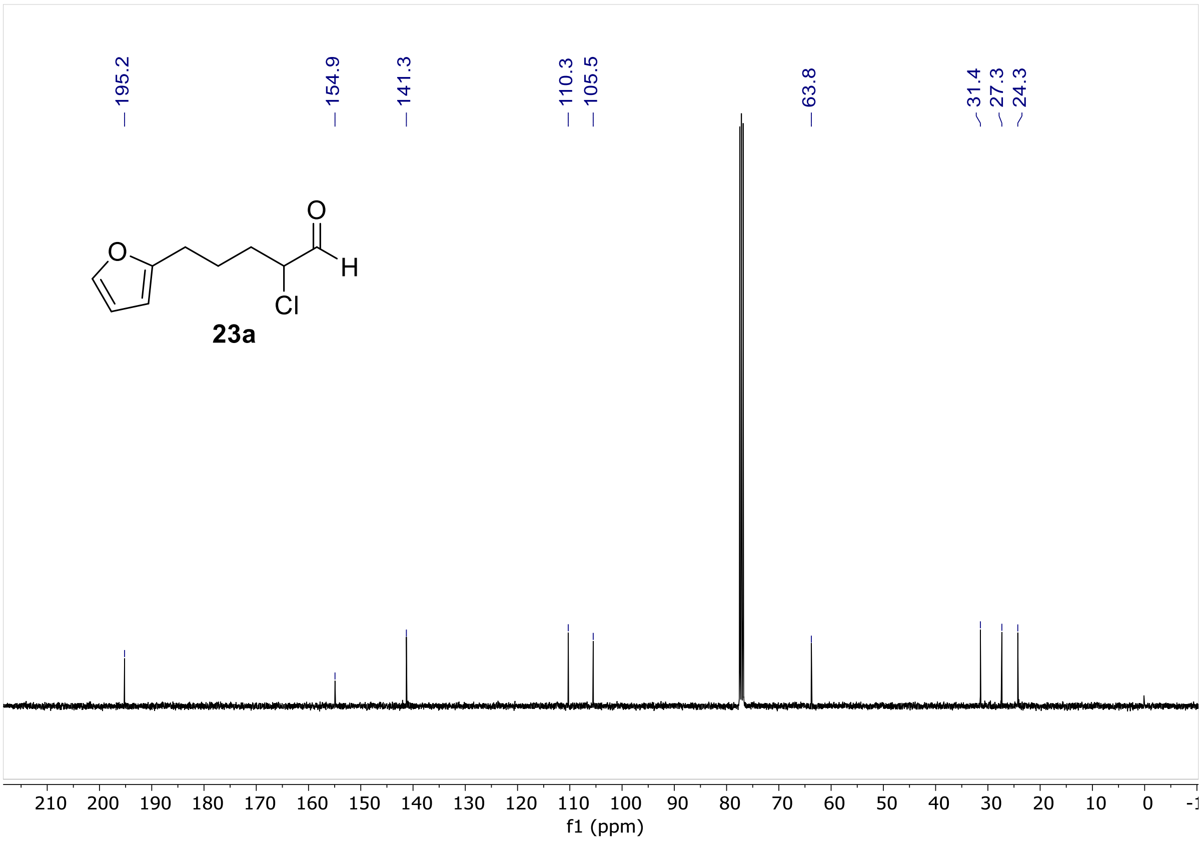

^{1}H NMR (500 MHz, $CDCl_3$): **23b**

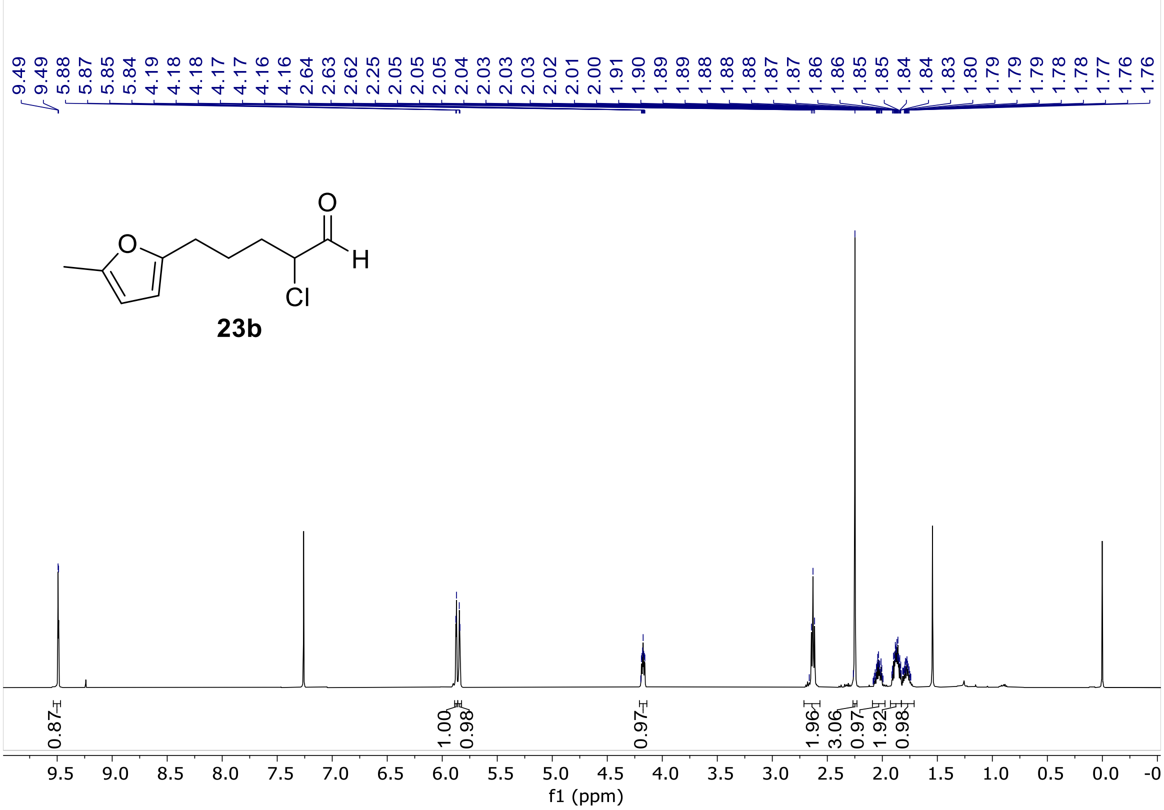

$^{13}C\{^{1}H\}$ NMR (100 MHz, $CDCl_3$): **23b**

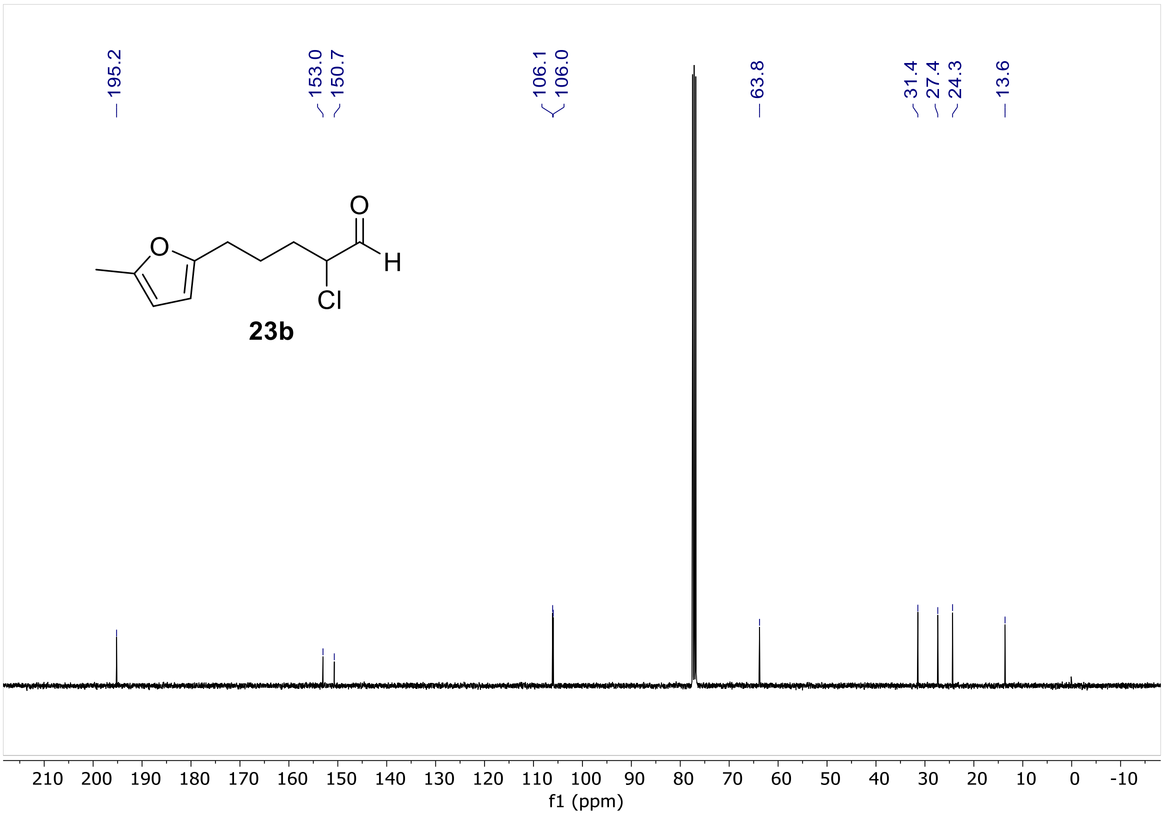

^{1}H NMR (500 MHz, $CDCl_3$): **23c**

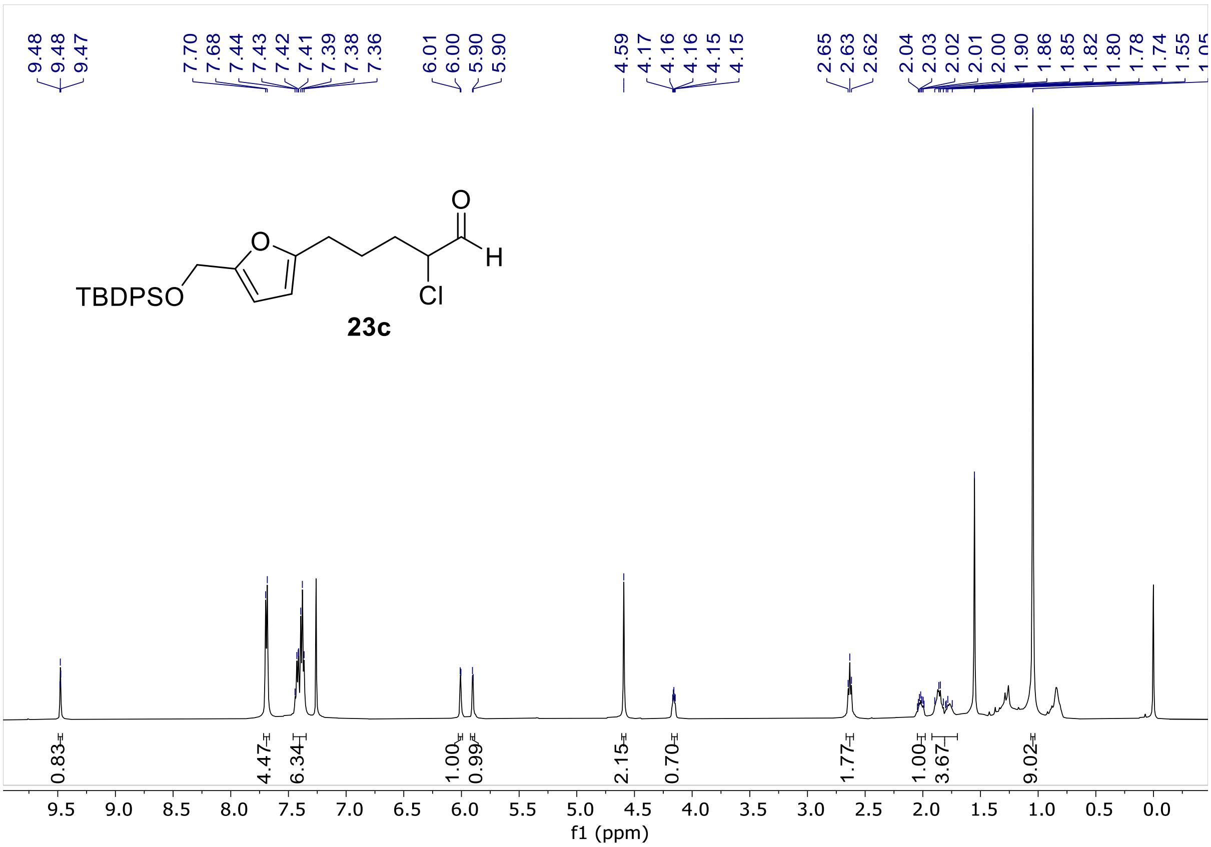

$^{13}C\{^{1}H\}$ NMR (100 MHz, $CDCl_3$): **23c**

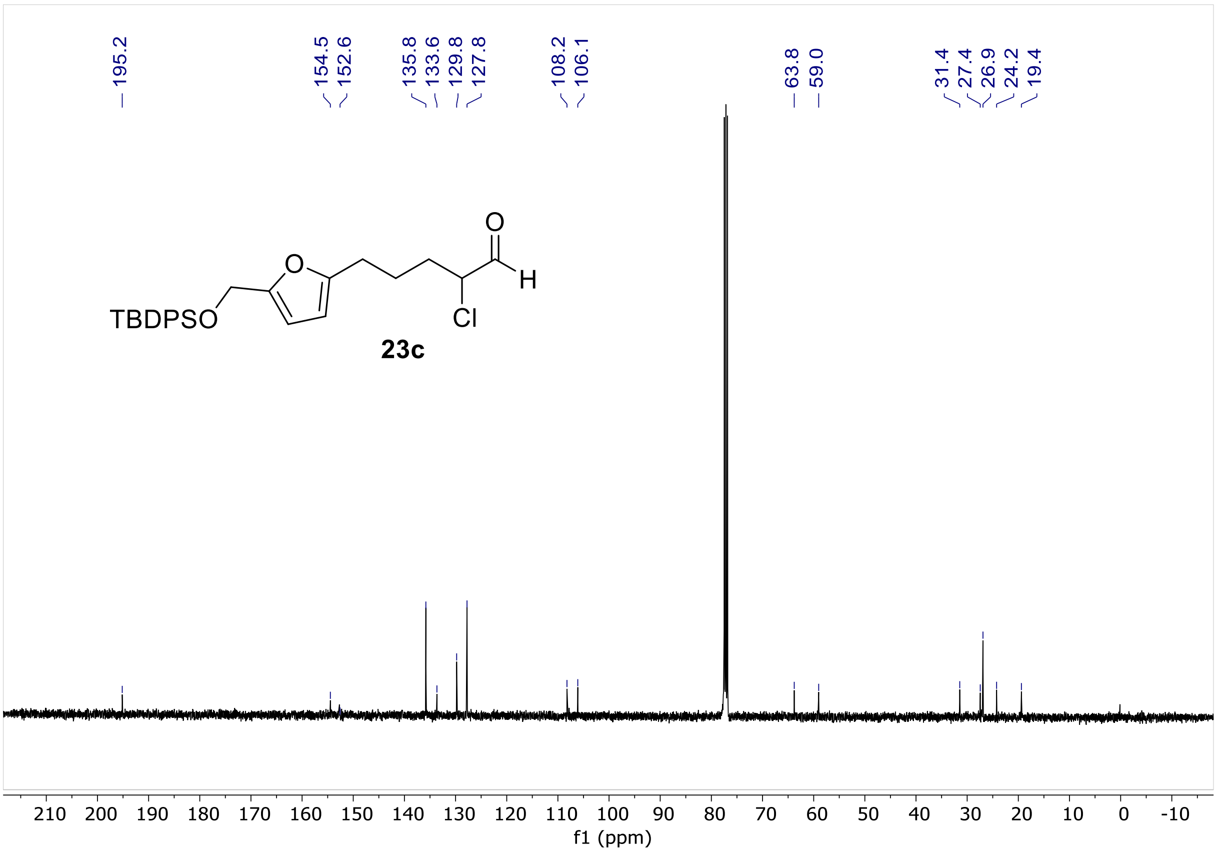

^{1}H NMR (500 MHz, $CDCl_3$): **23d**

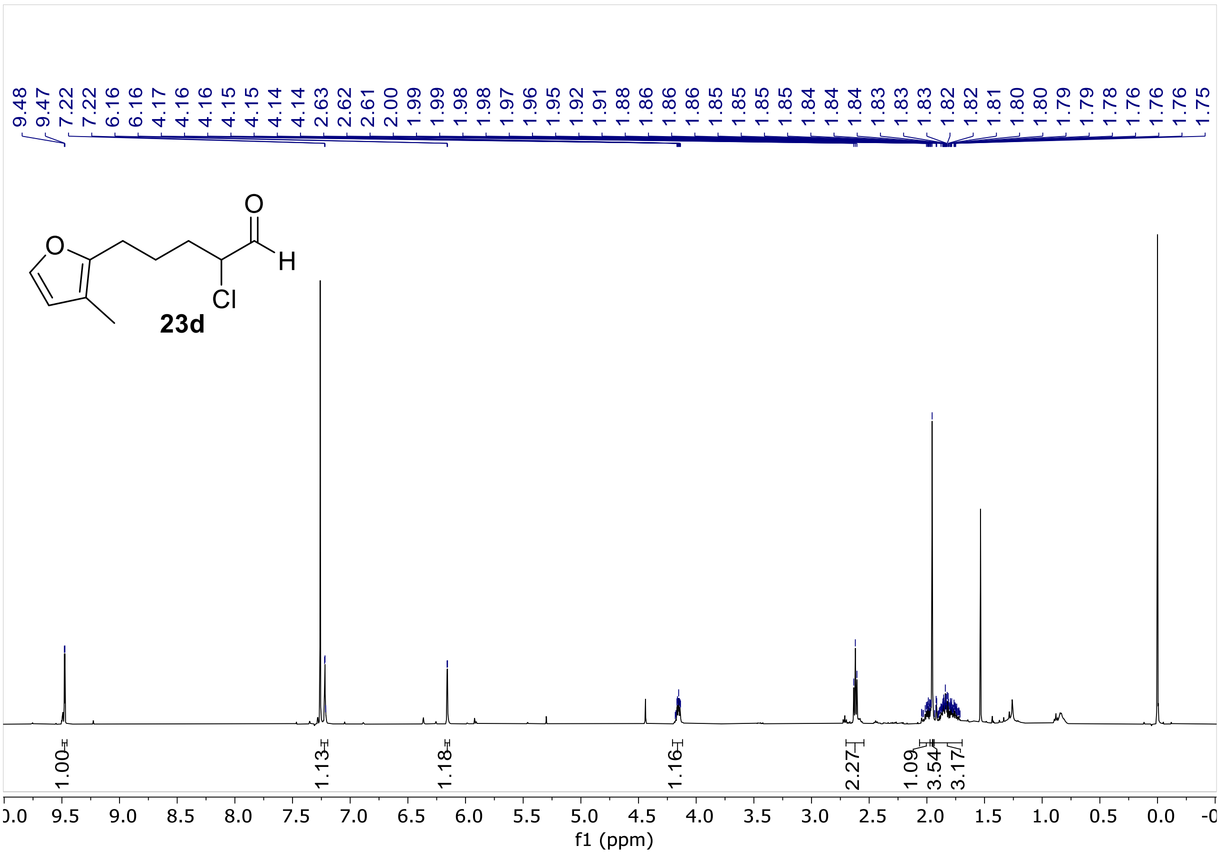

$^{13}C\{^{1}H\}$ NMR (100 MHz, $CDCl_3$): **23d**

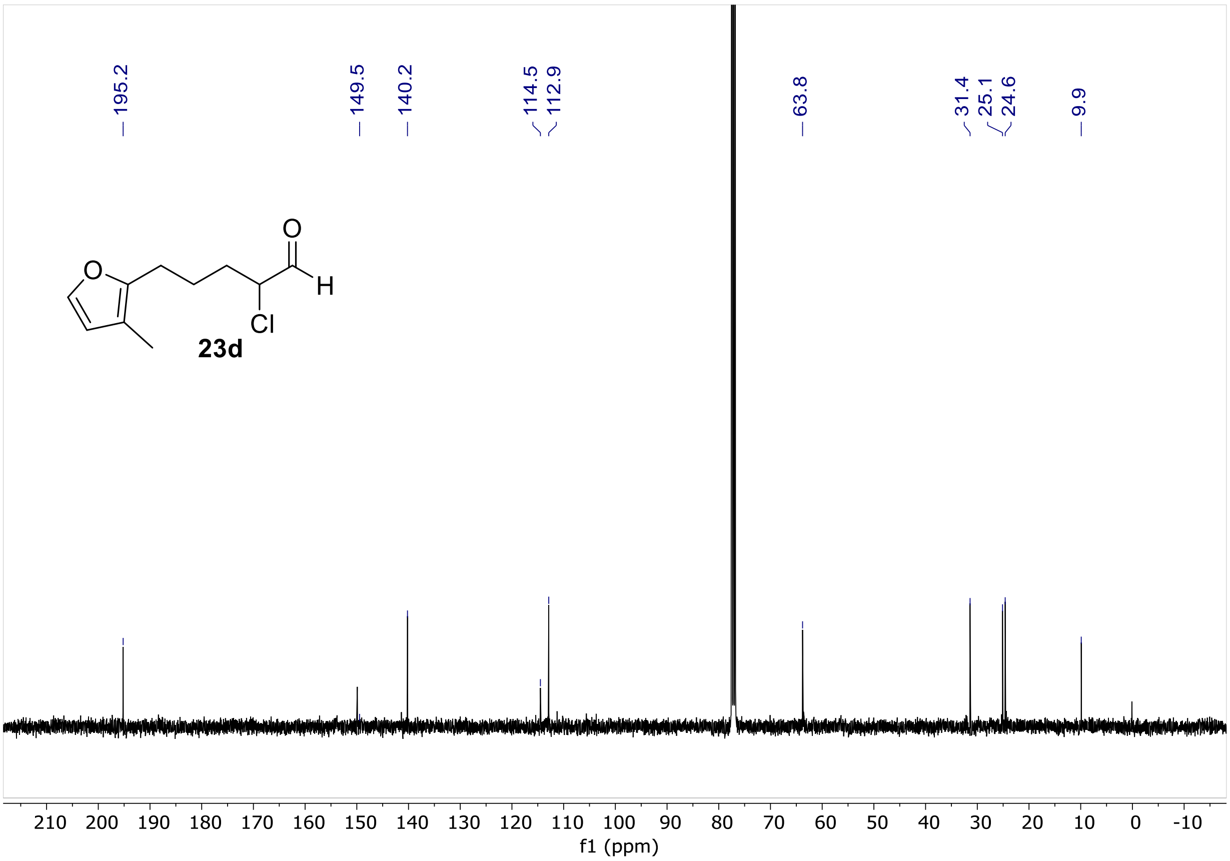

^1H NMR (500 MHz, $CDCl_3$): **23e**

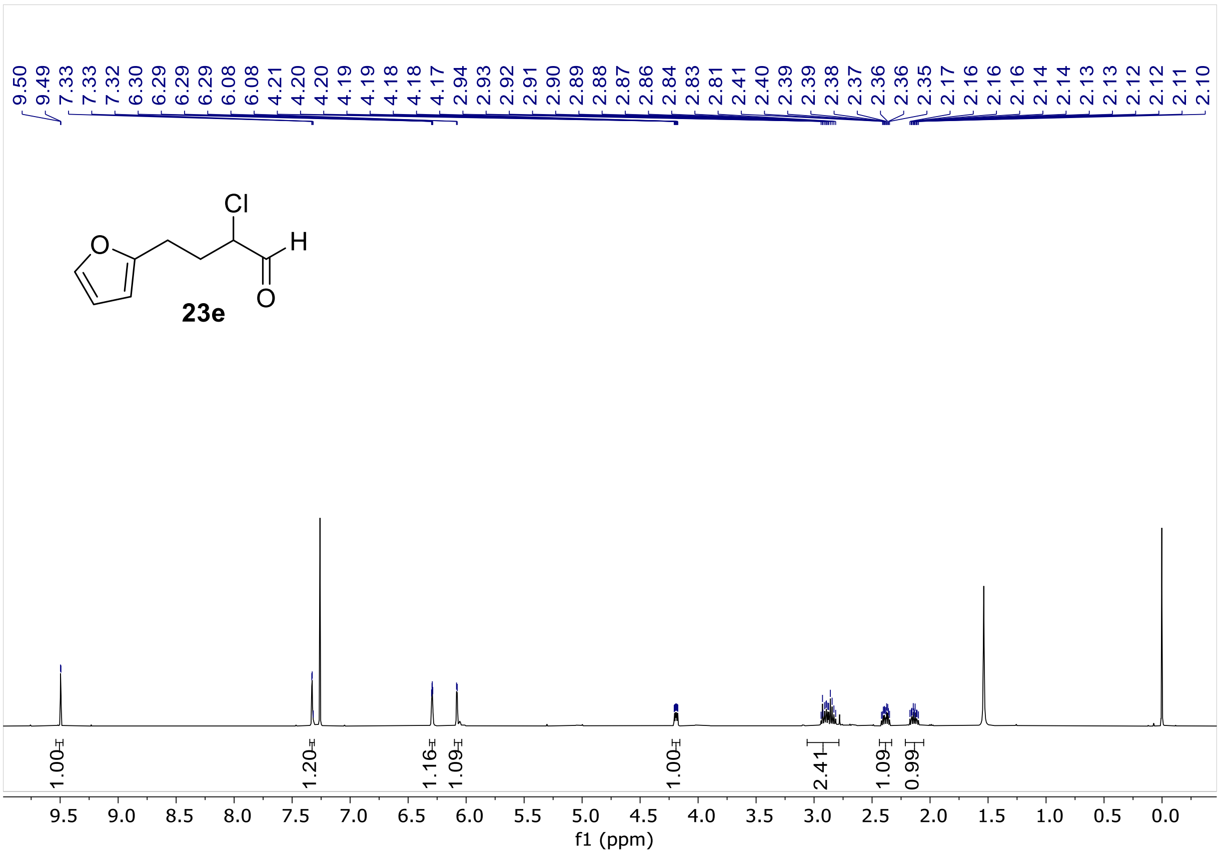

$^{13}C\{^1H\}$ NMR (100 MHz, $CDCl_3$): **23e**

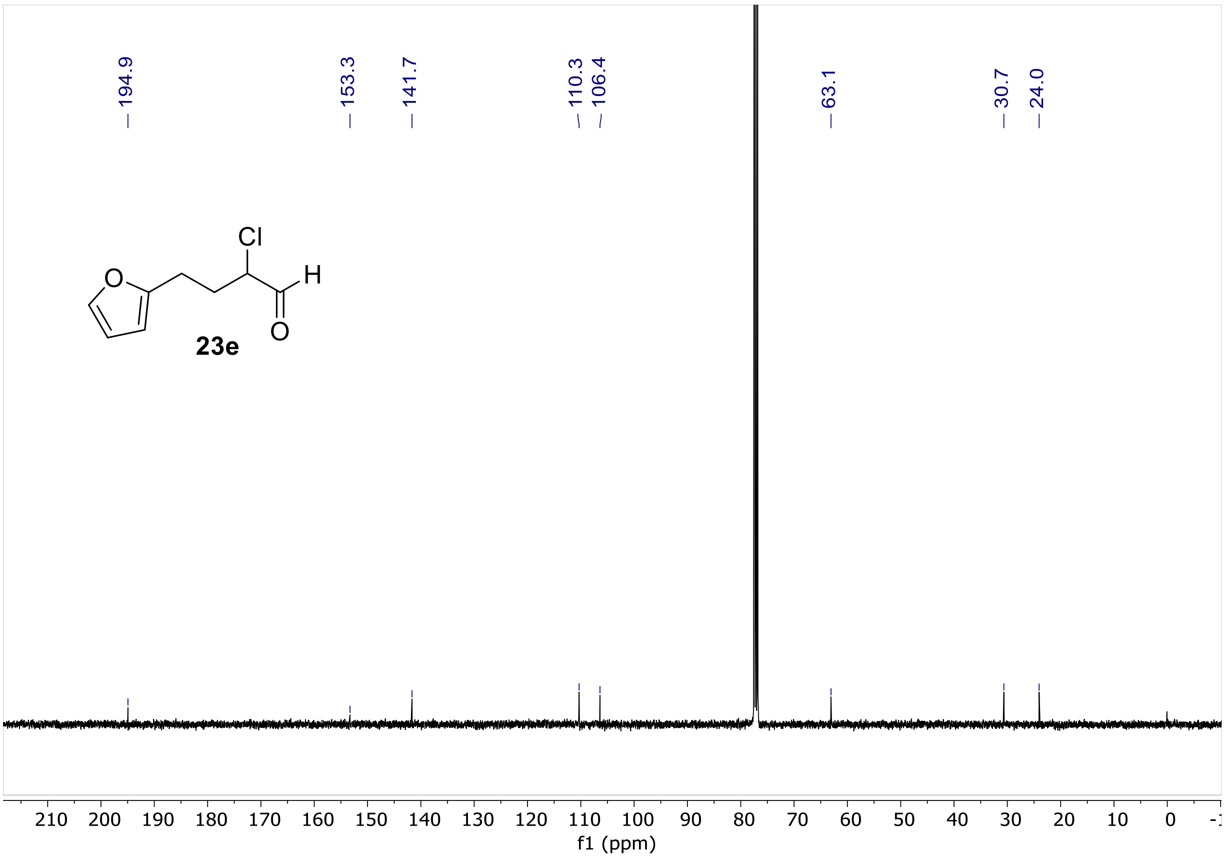

^{1}H NMR (400 MHz, $CDCl_3$): **23f**

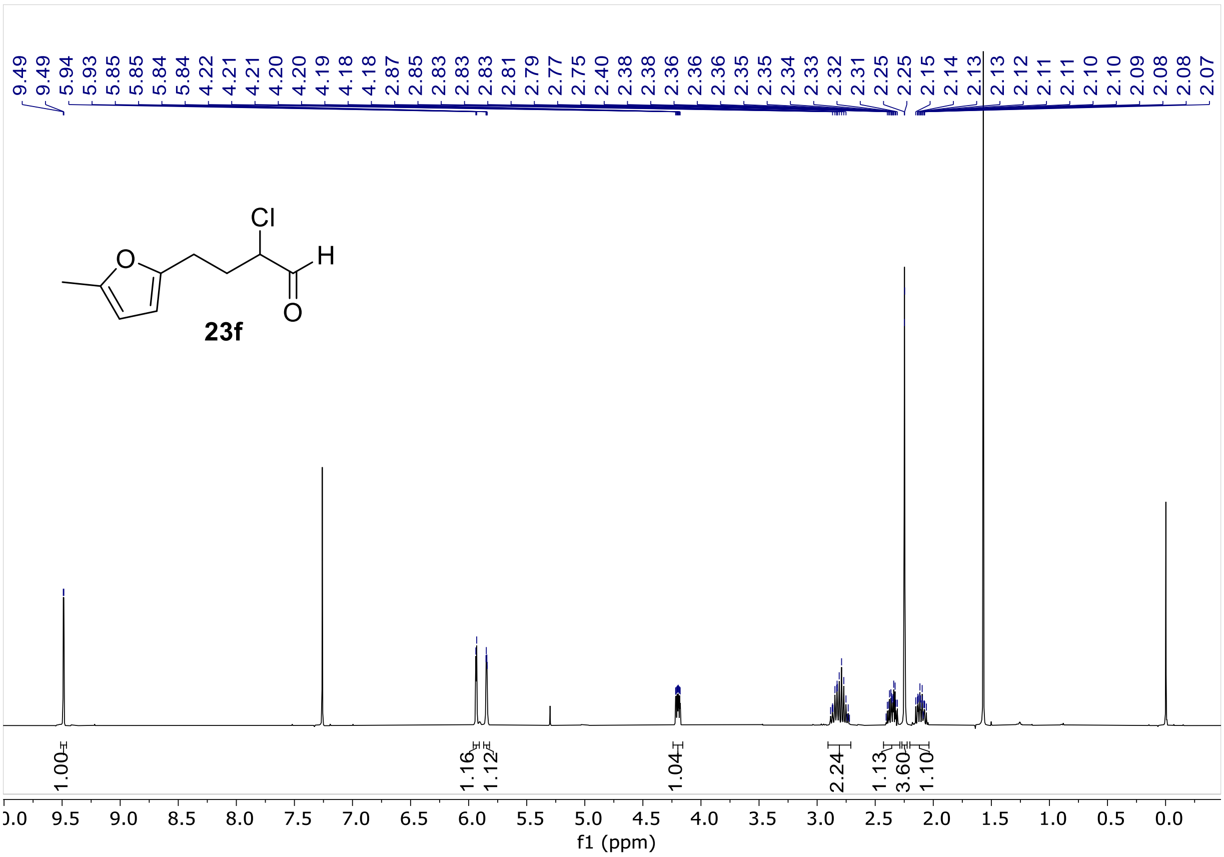

$^{13}C\{^{1}H\}$ NMR (100 MHz, $CDCl_3$): **23f**

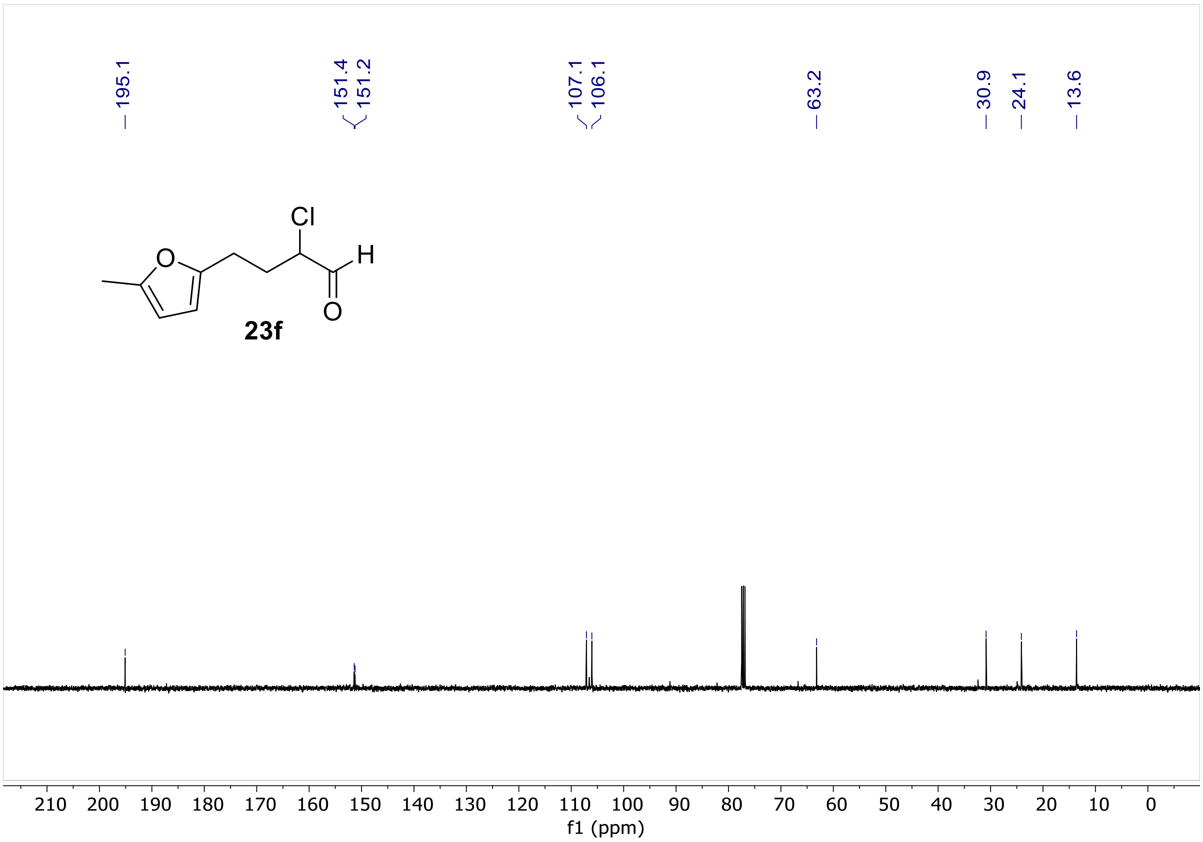

^{1}H NMR (400 MHz, $CDCl_3$): **23g**

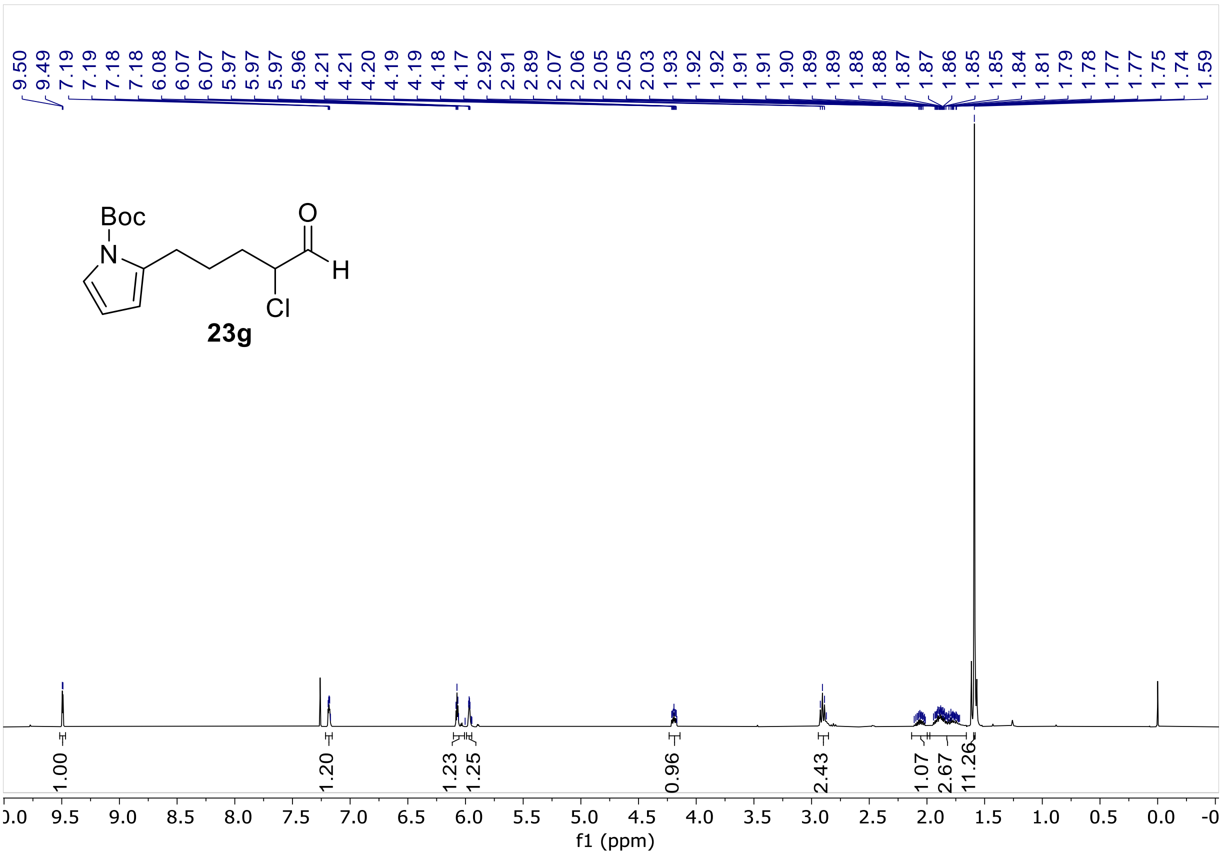

^{13}C{^{1}H} NMR (100 MHz, $CDCl_3$): **23g**

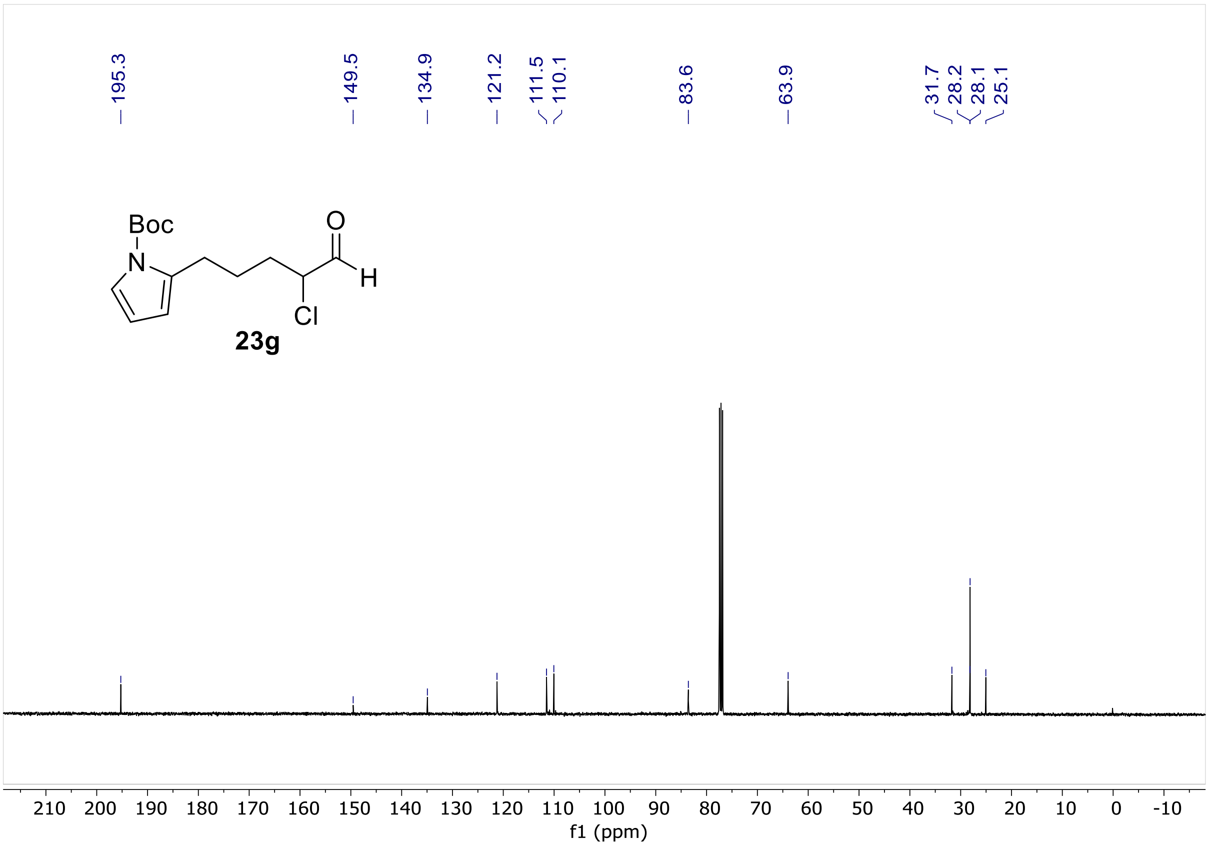

^{1}H NMR (500 MHz, $CDCl_3$): **23h**

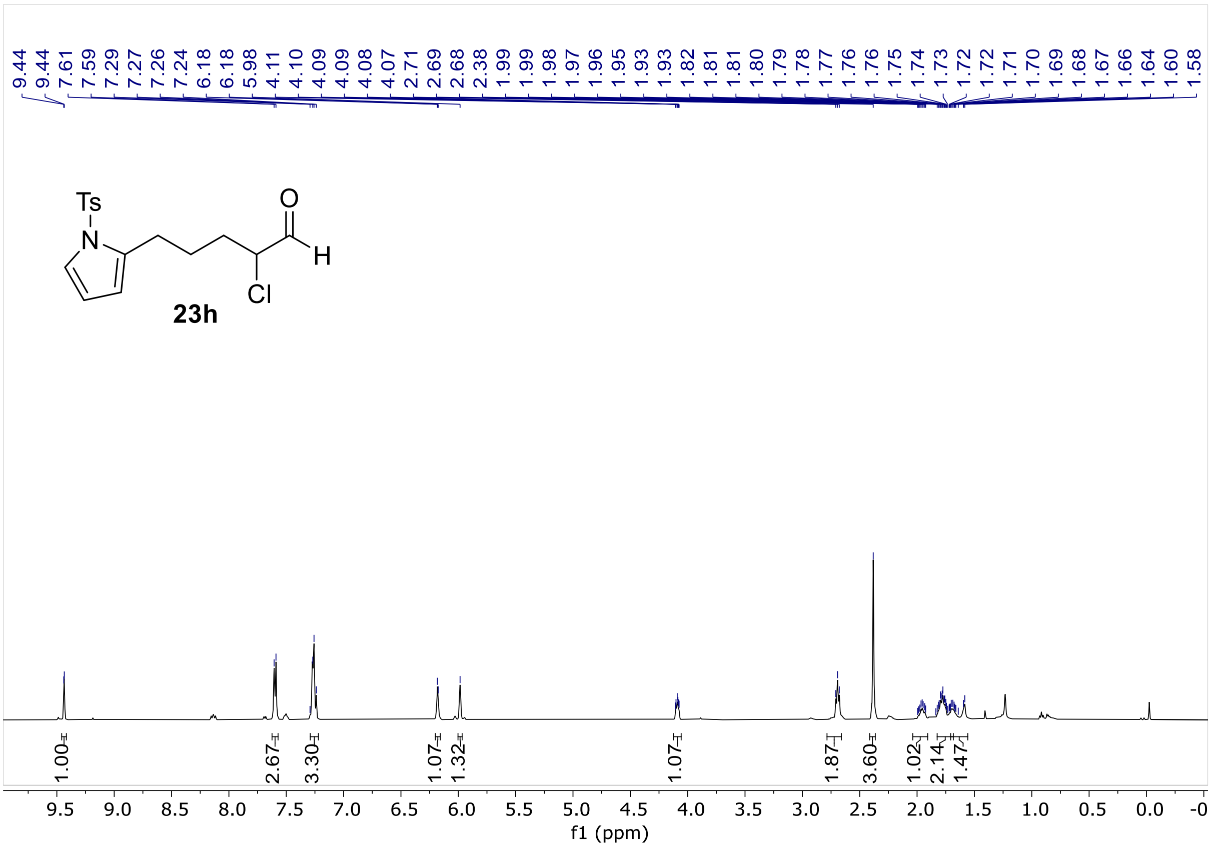

^{13}C{^{1}H} NMR (100 MHz, $CDCl_3$): **23h**

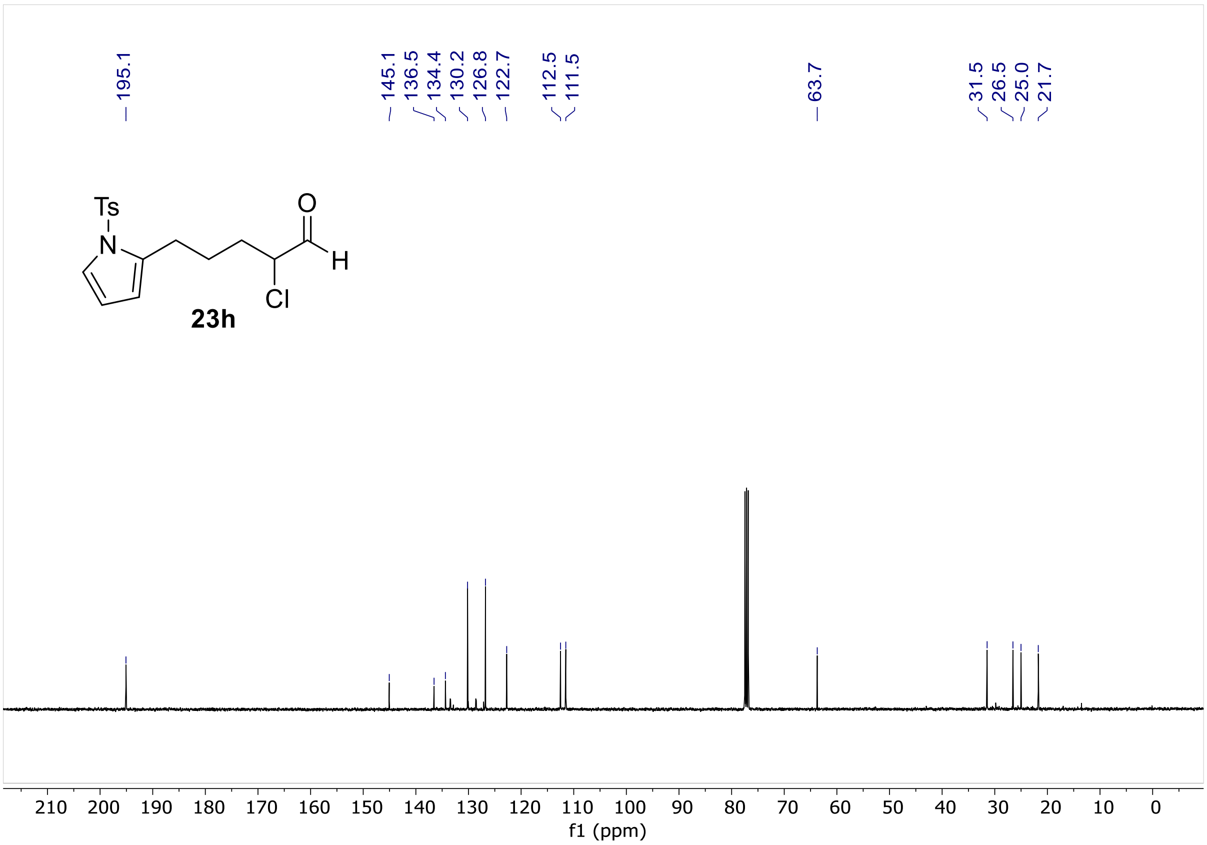

^1H NMR (400 MHz, $CDCl_3$): **23i**

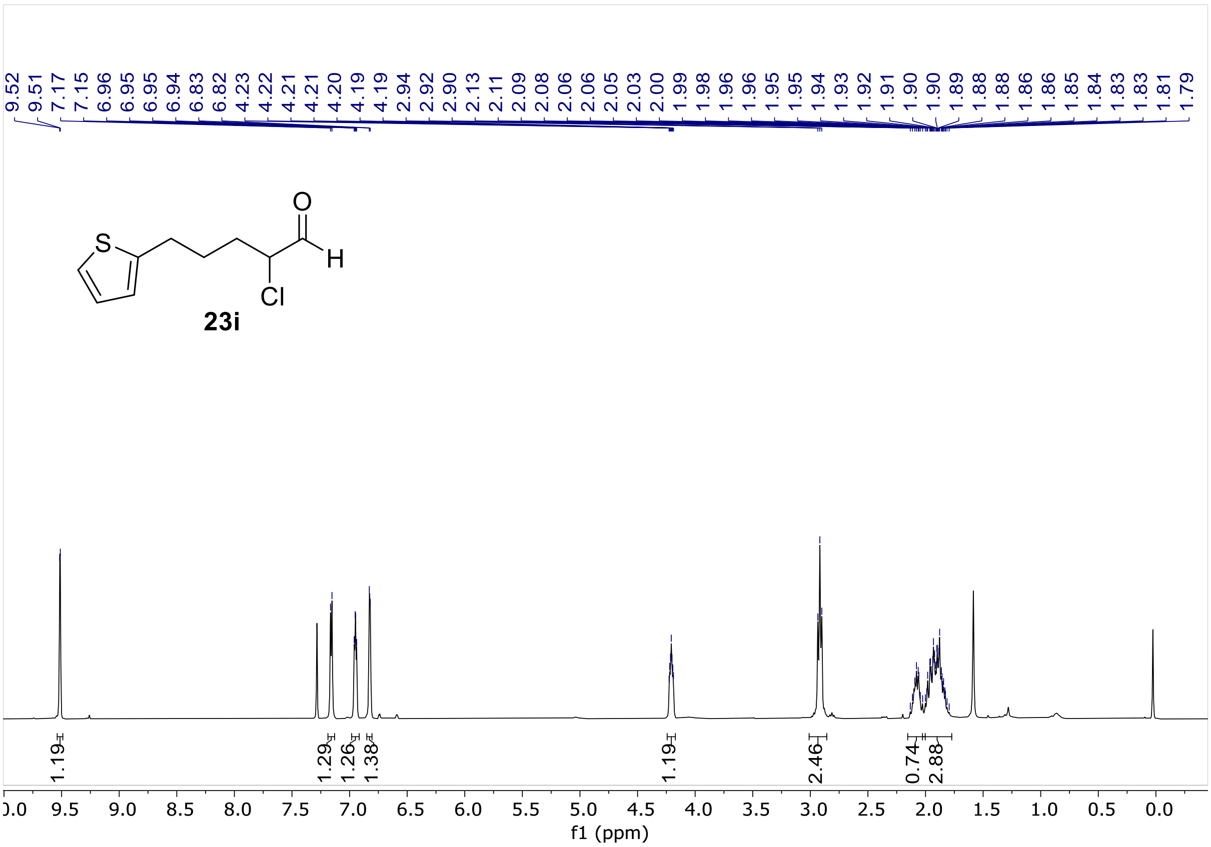

$^{13}C\{^1H\}$ NMR (100 MHz, $CDCl_3$): **23i**

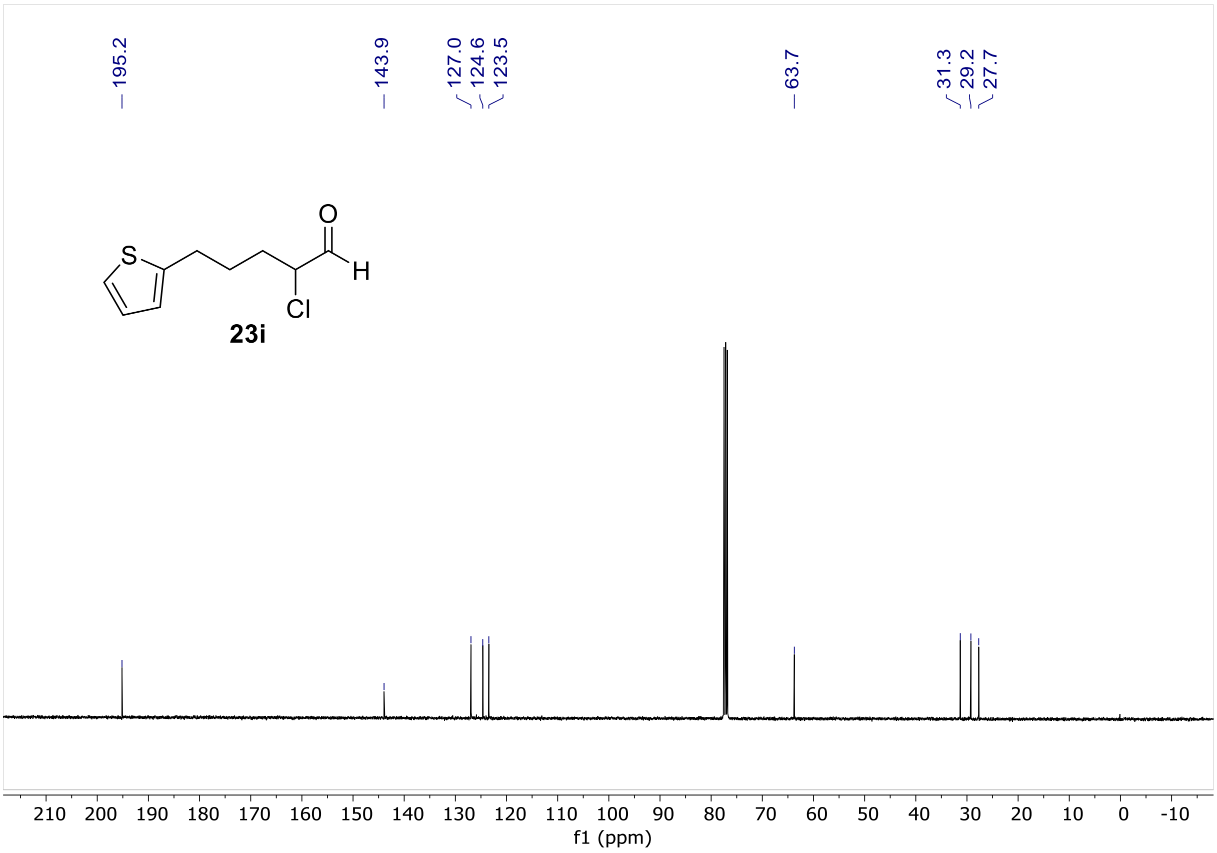

^{1}H NMR (400 MHz, $CDCl_3$): **23j**

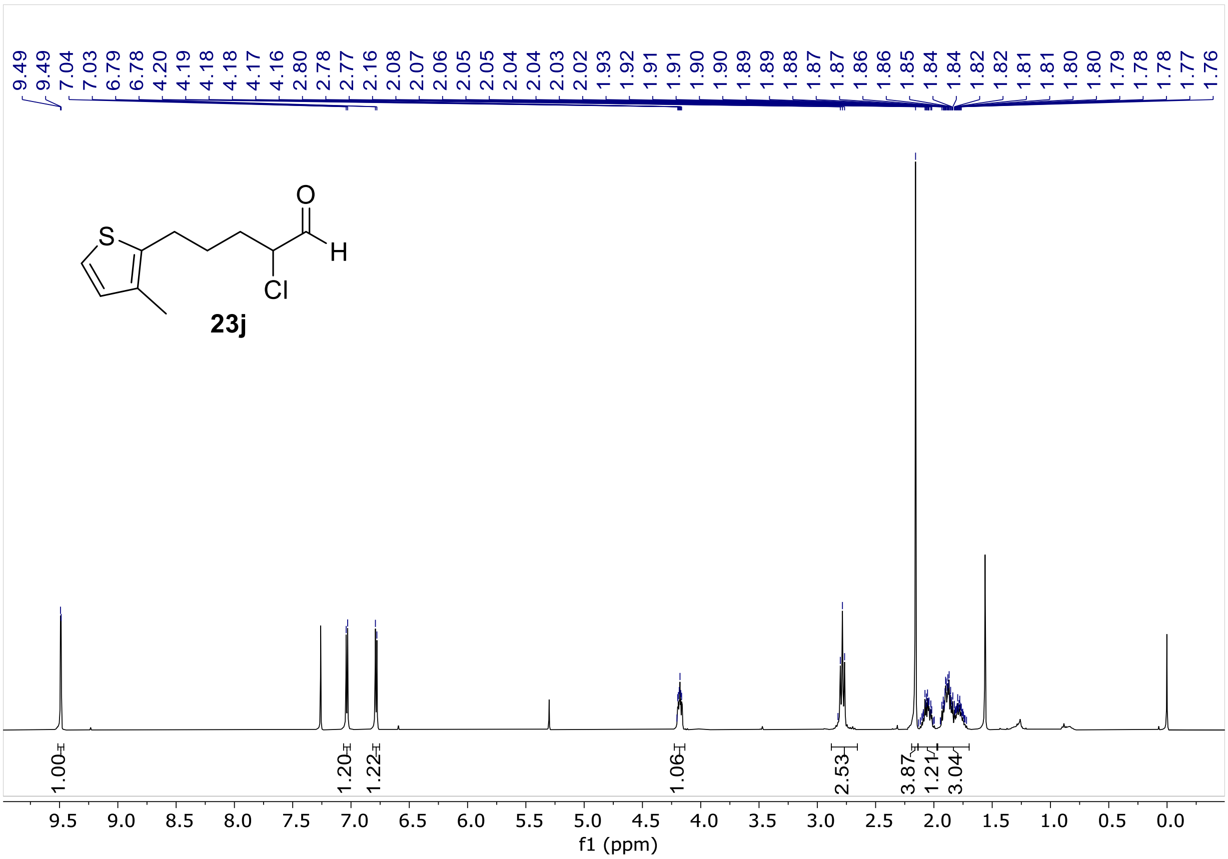

$^{13}C\{^1H\}$ NMR (100 MHz, $CDCl_3$): **23j**

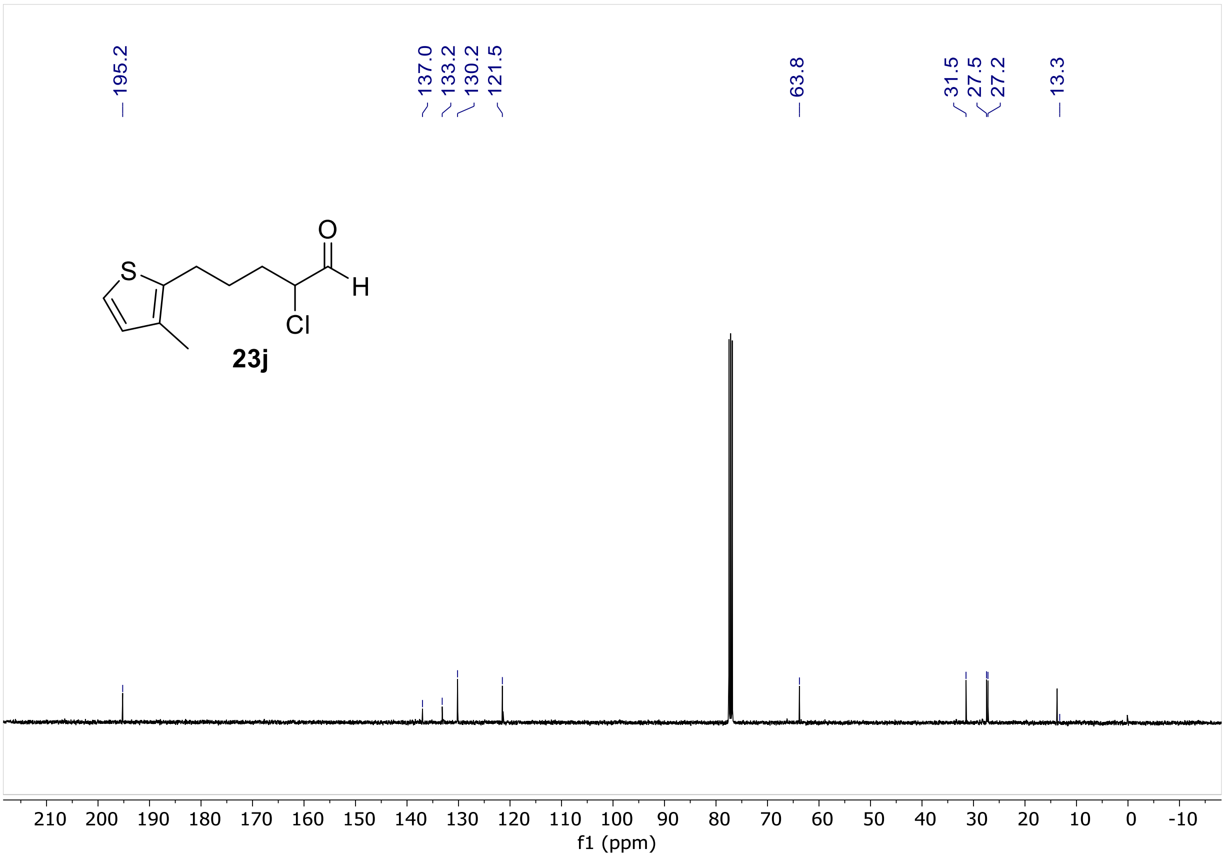

^{1}H NMR (500 MHz, $CDCl_3$): **23k**

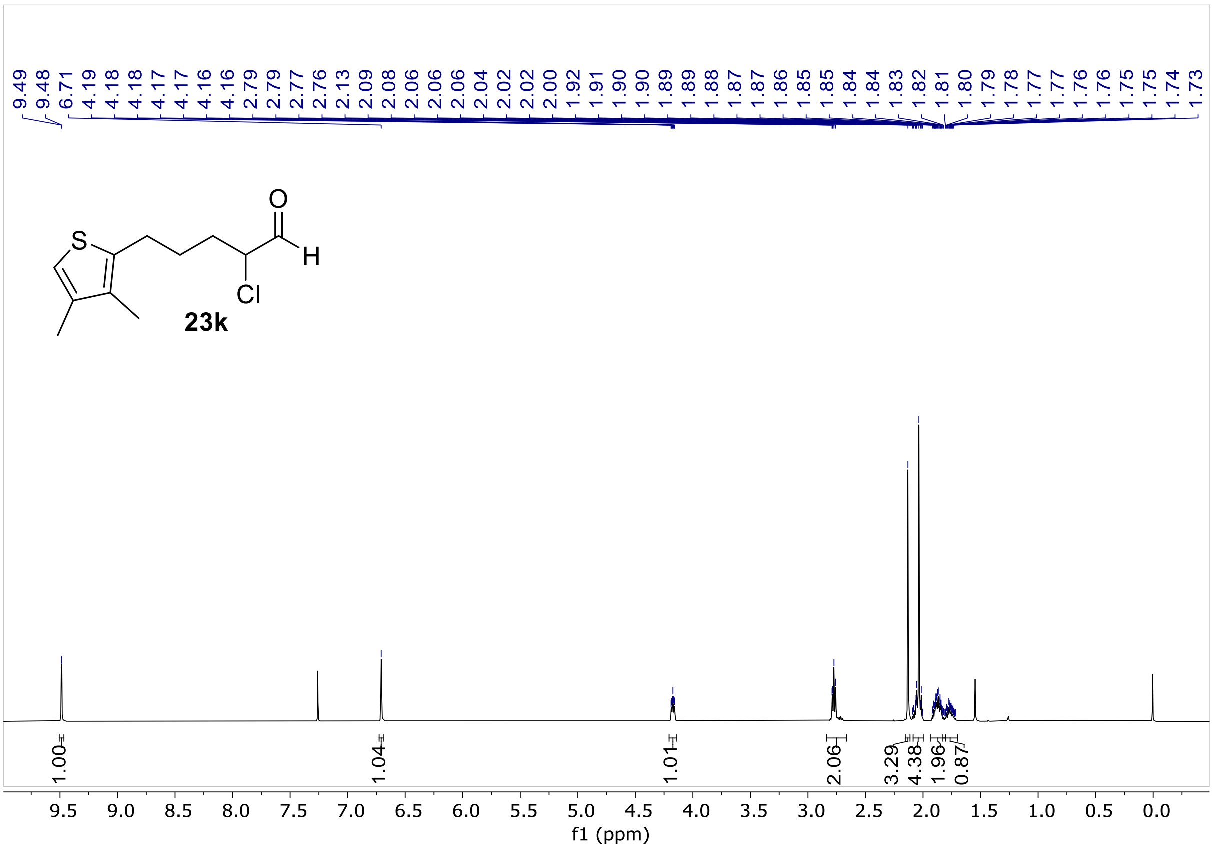

^{13}C{^{1}H} NMR (125 MHz, $CDCl_3$): **23k**

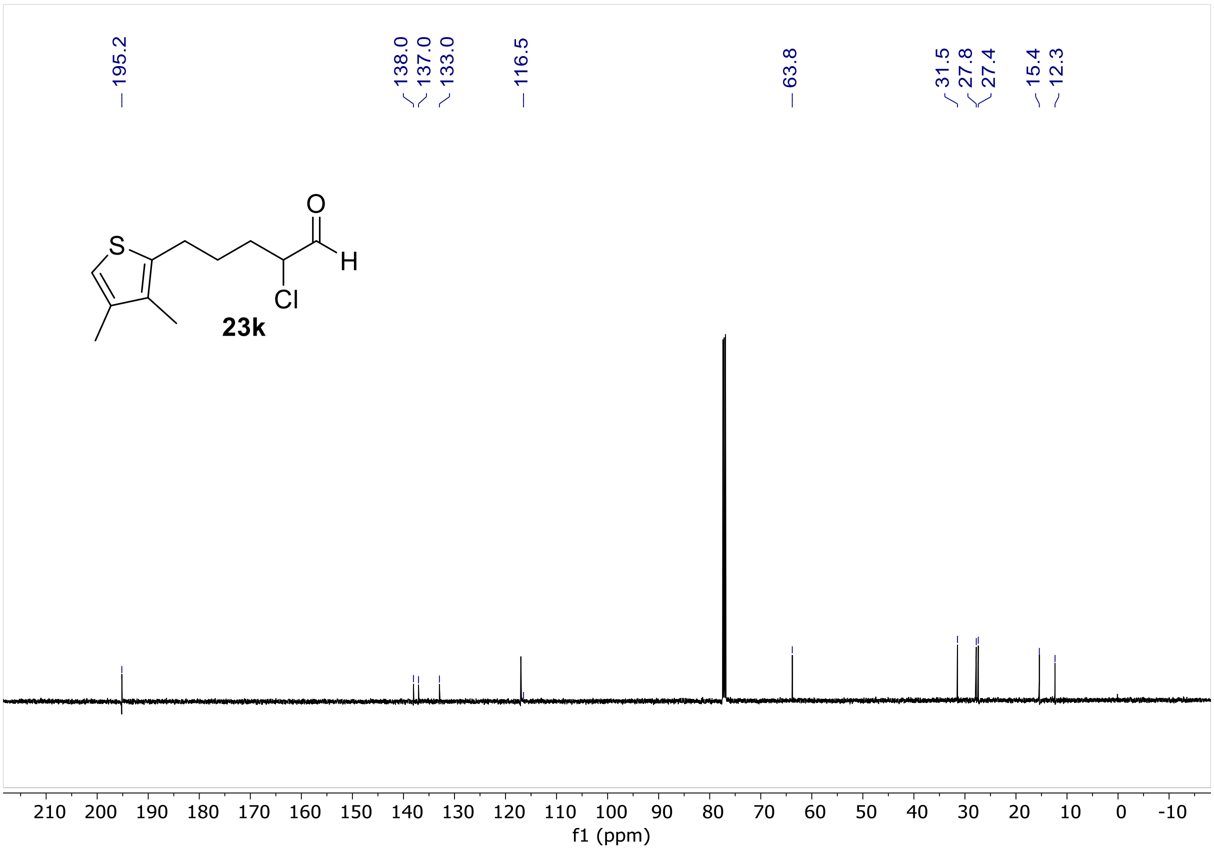

^{1}H NMR (500 MHz, $CDCl_3$): **25a**

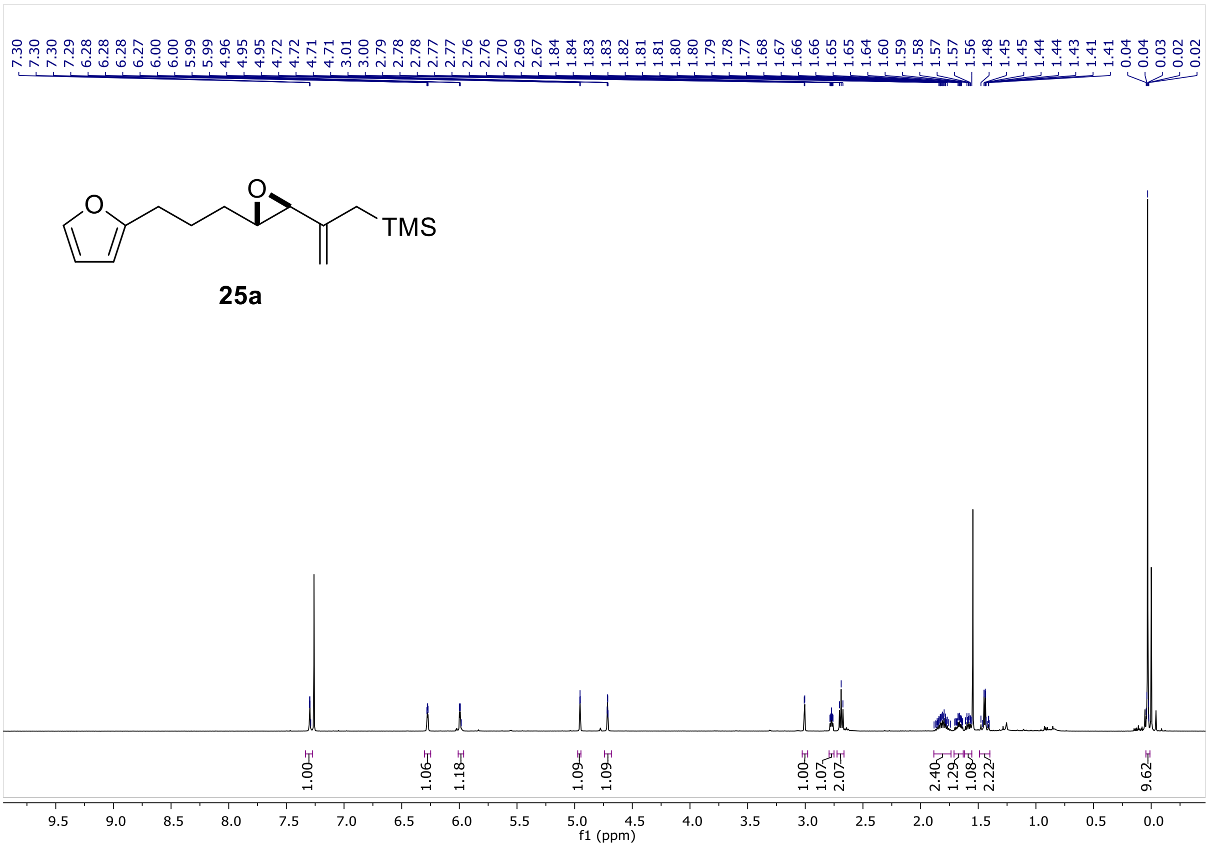

$^{13}C\{^{1}H\}$ NMR (125 MHz, $CDCl_3$): **25a**

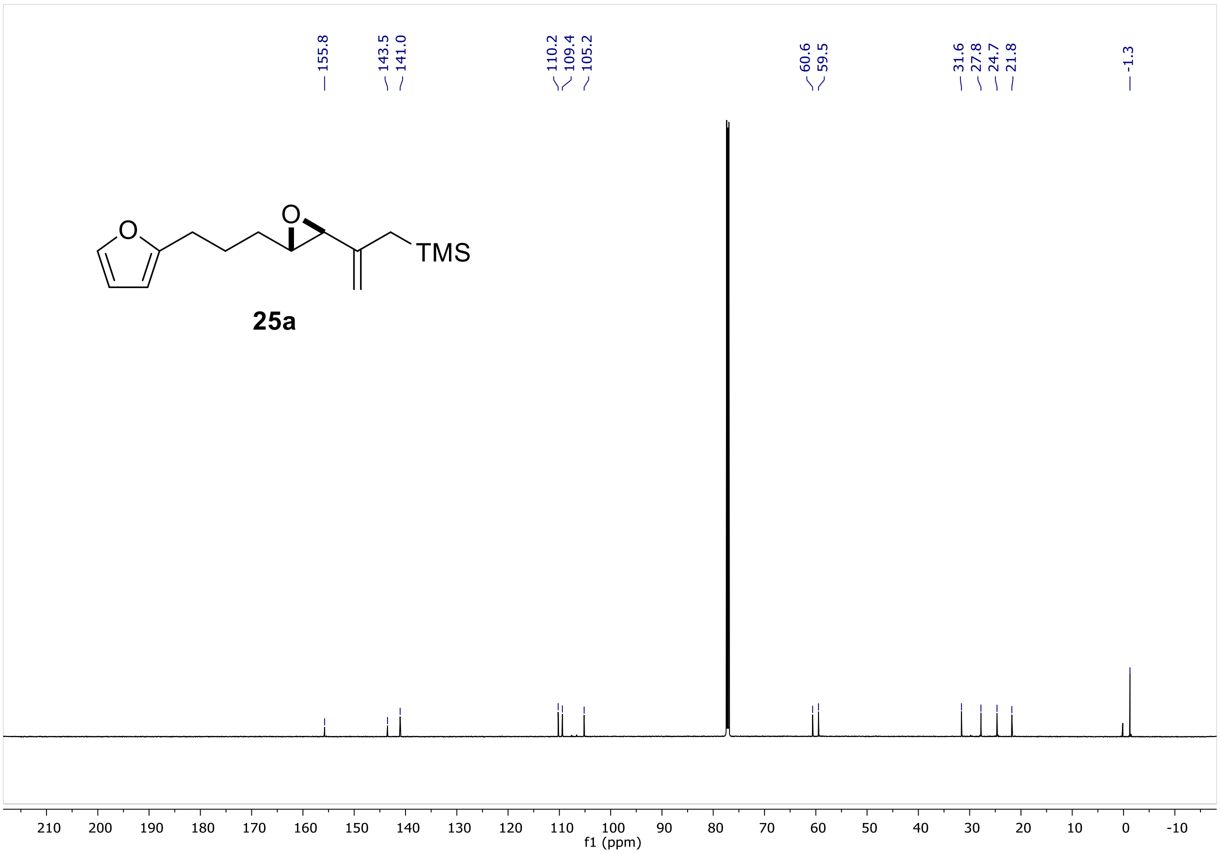

^{1}H NMR (500 MHz, $CDCl_3$): **25b**

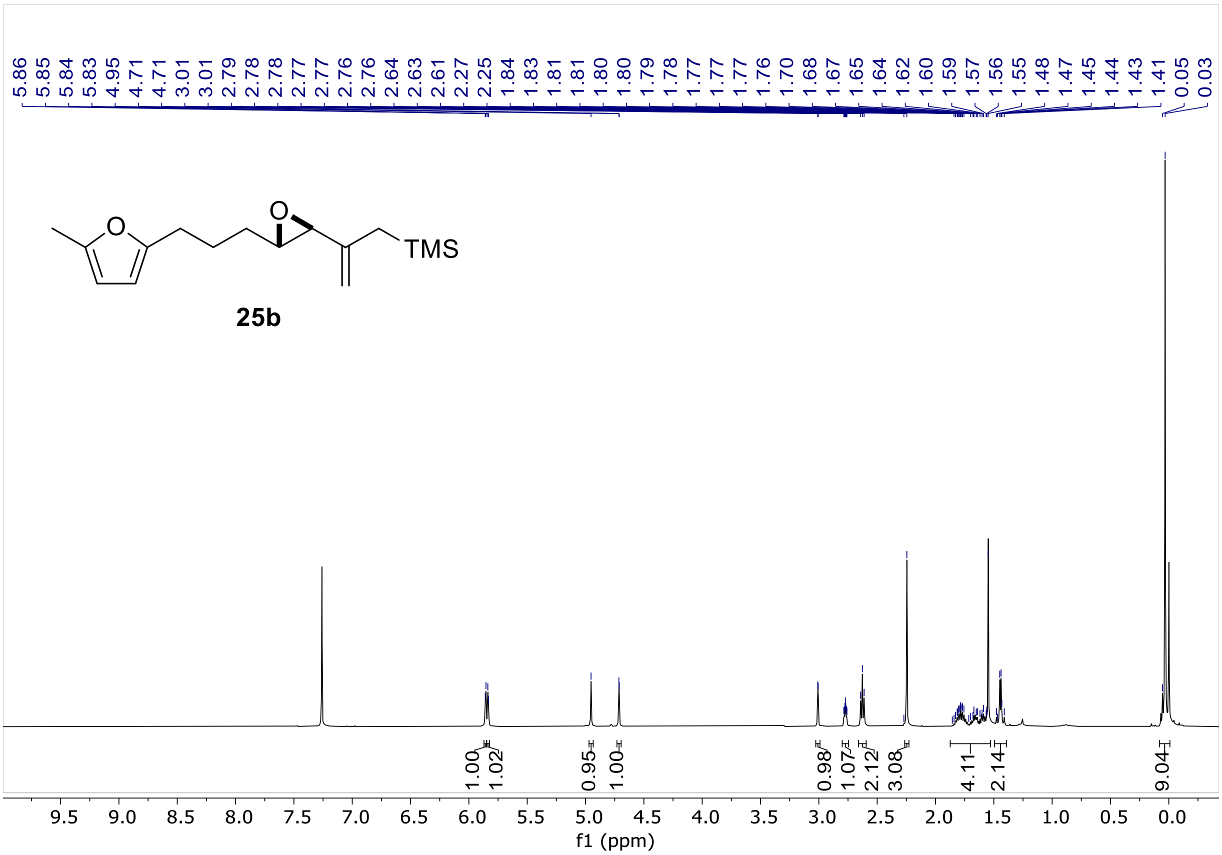

$^{13}C\{^{1}H\}$ NMR (100 MHz, $CDCl_3$): **25b**

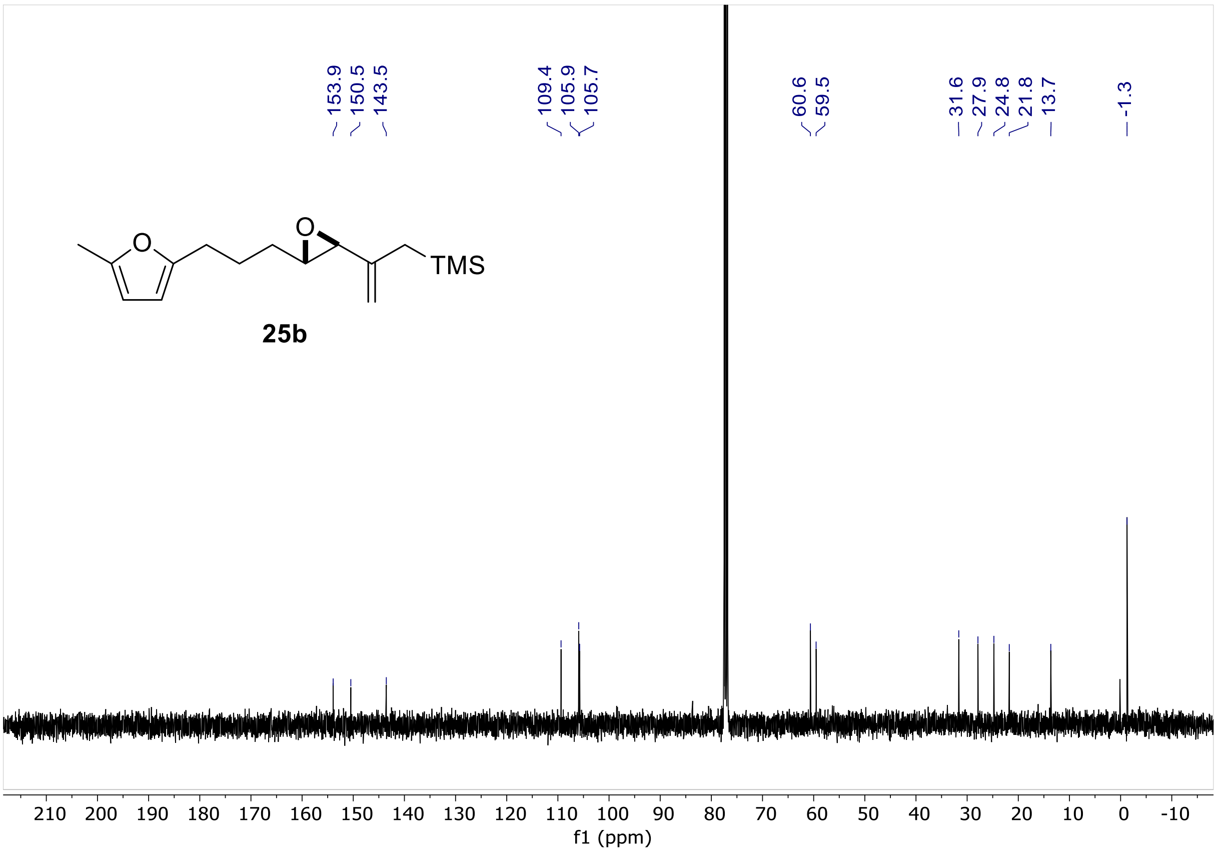

^{1}H NMR (600 MHz, $CDCl_3$): **25c**

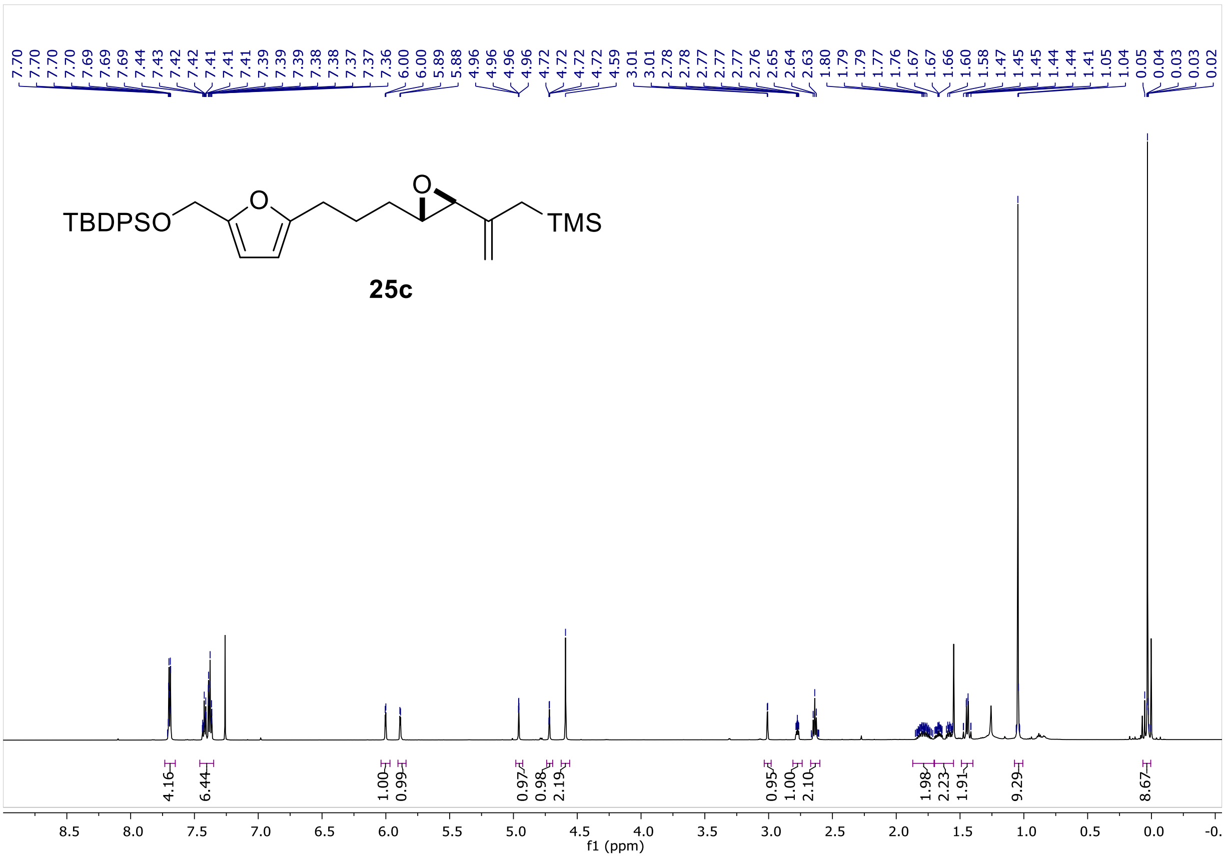

$^{13}C\{^{1}H\}$ NMR (150 MHz, $CDCl_3$): **25c**

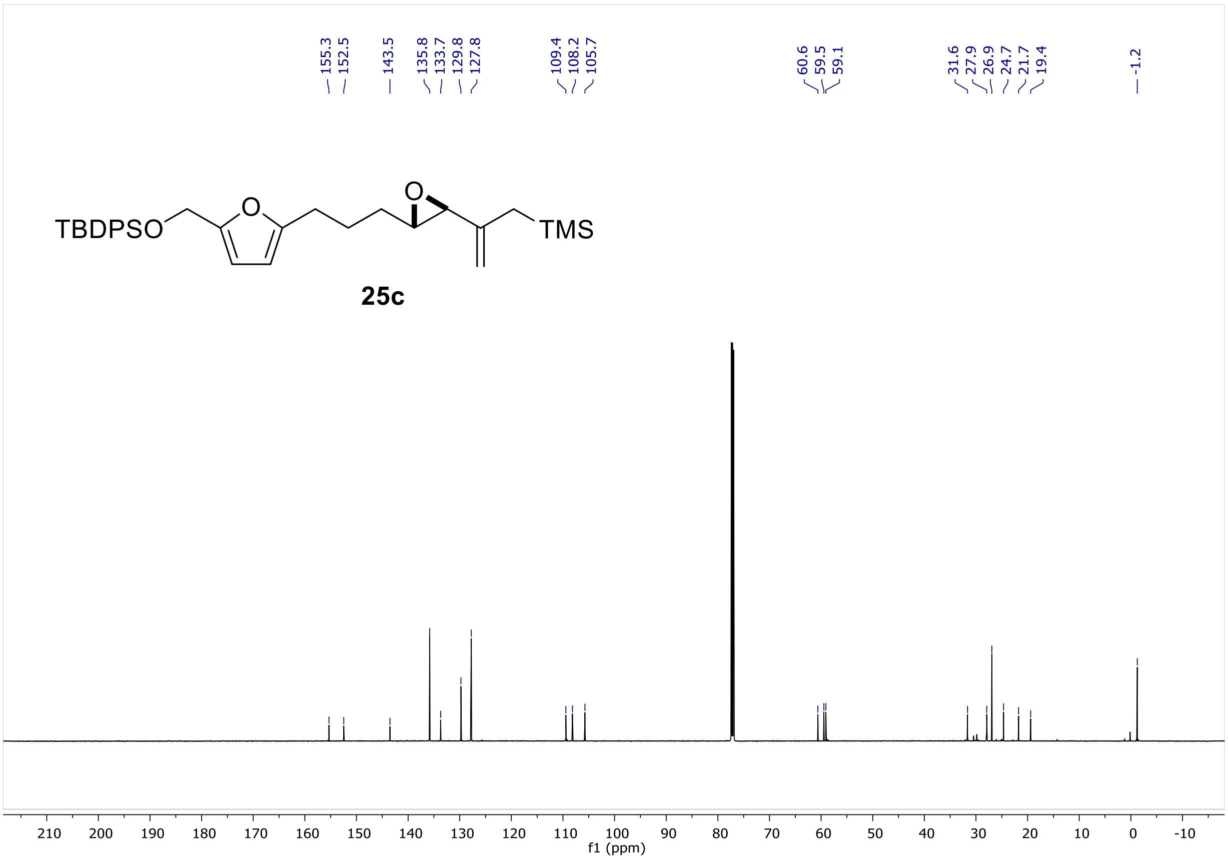

^{1}H NMR (500 MHz, $CDCl_3$): **25d**

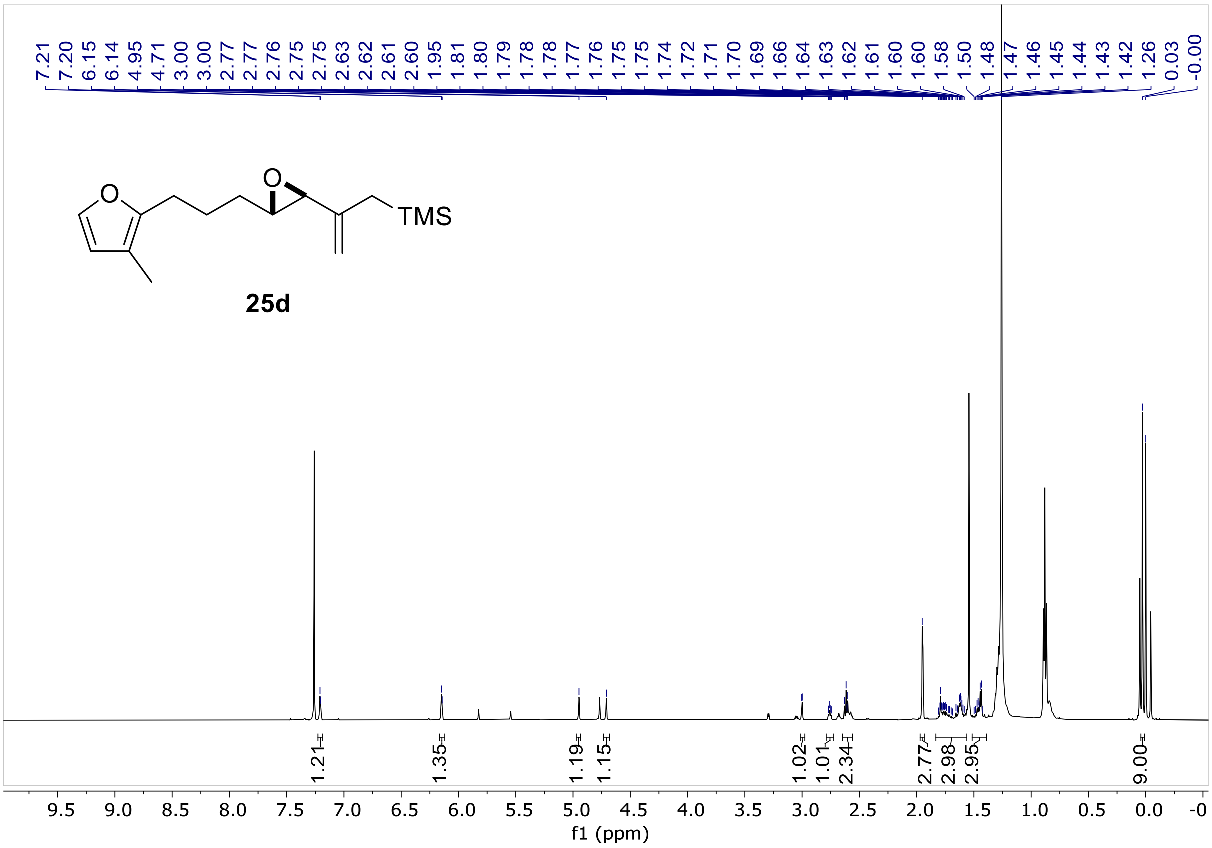

^{13}C{^{1}H} NMR (100 MHz, $CDCl_3$): **25d**

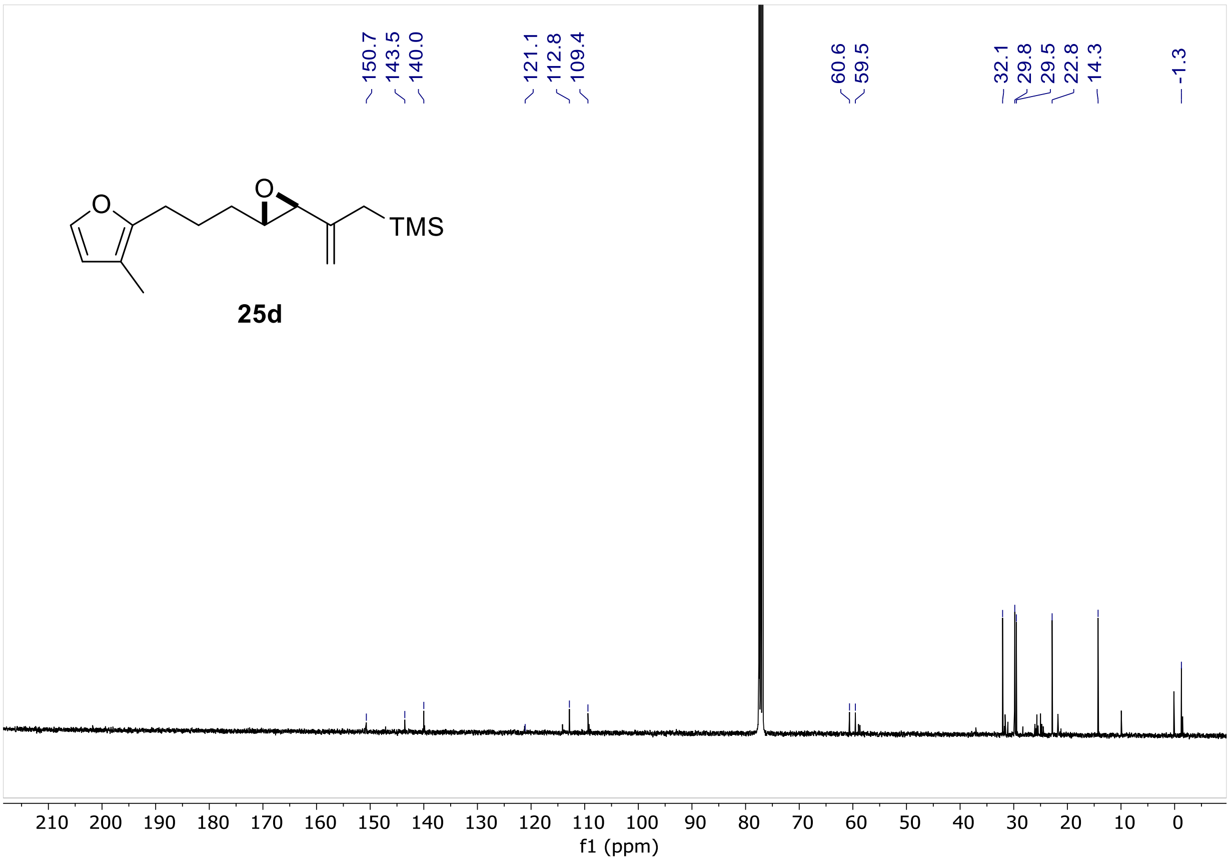

^{1}H NMR (500 MHz, $CDCl_3$): **25e**

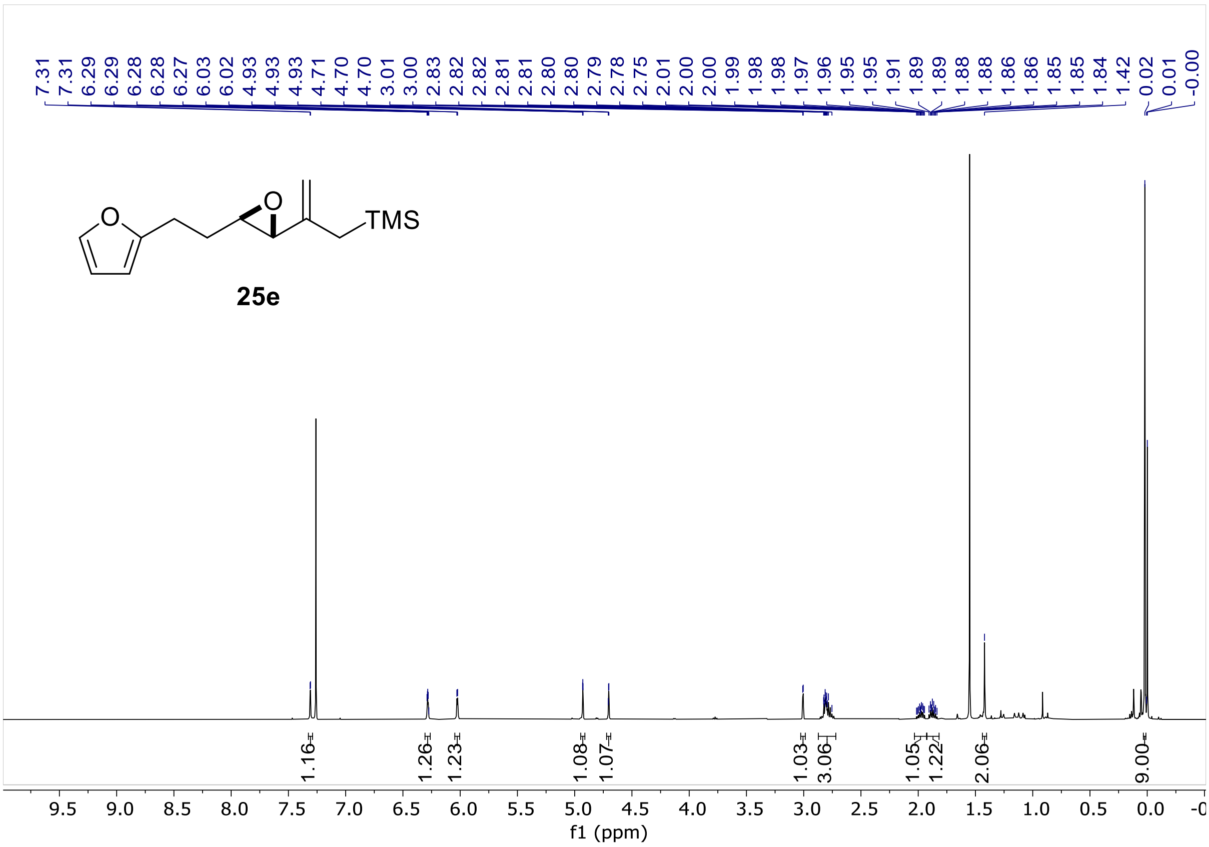

$^{13}C\{^{1}H\}$ NMR (125 MHz, $CDCl_3$): **25e**

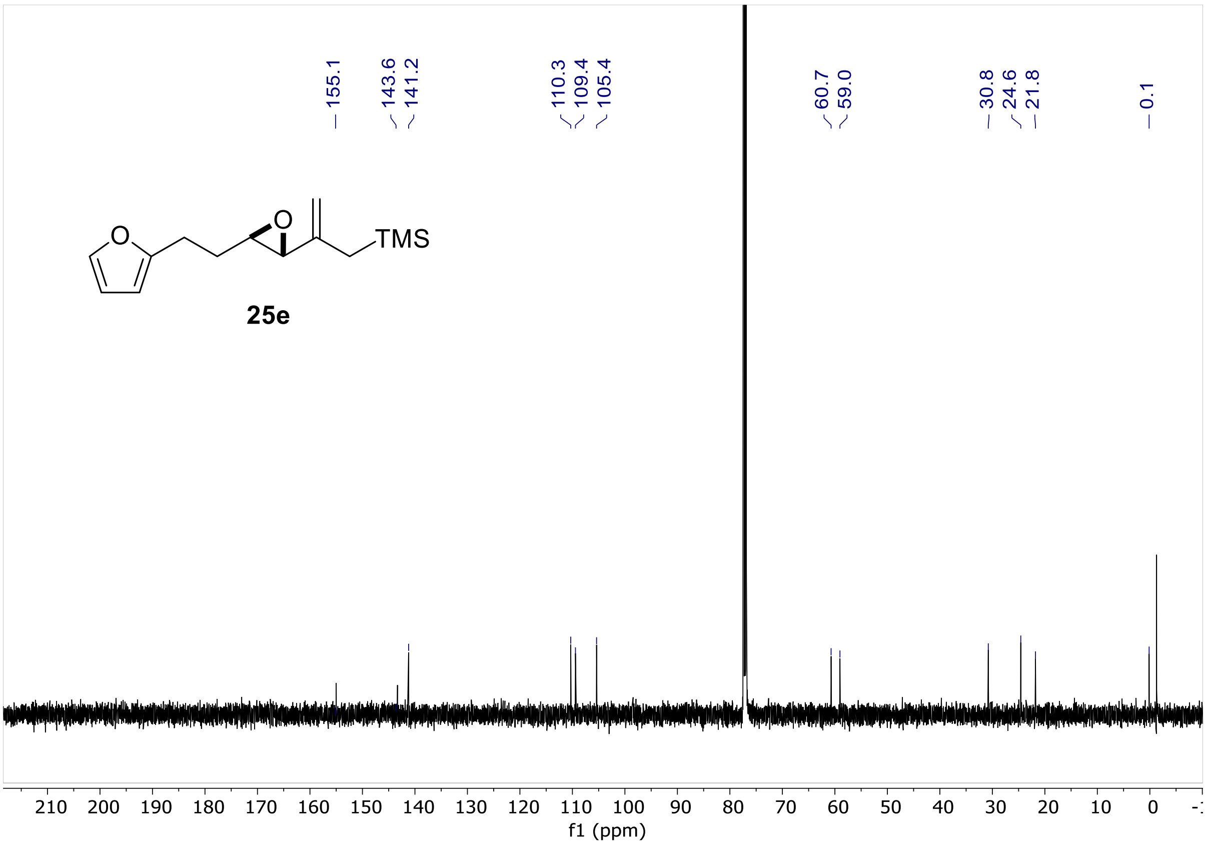

^{1}H NMR (400 MHz, $CDCl_3$): **25f**

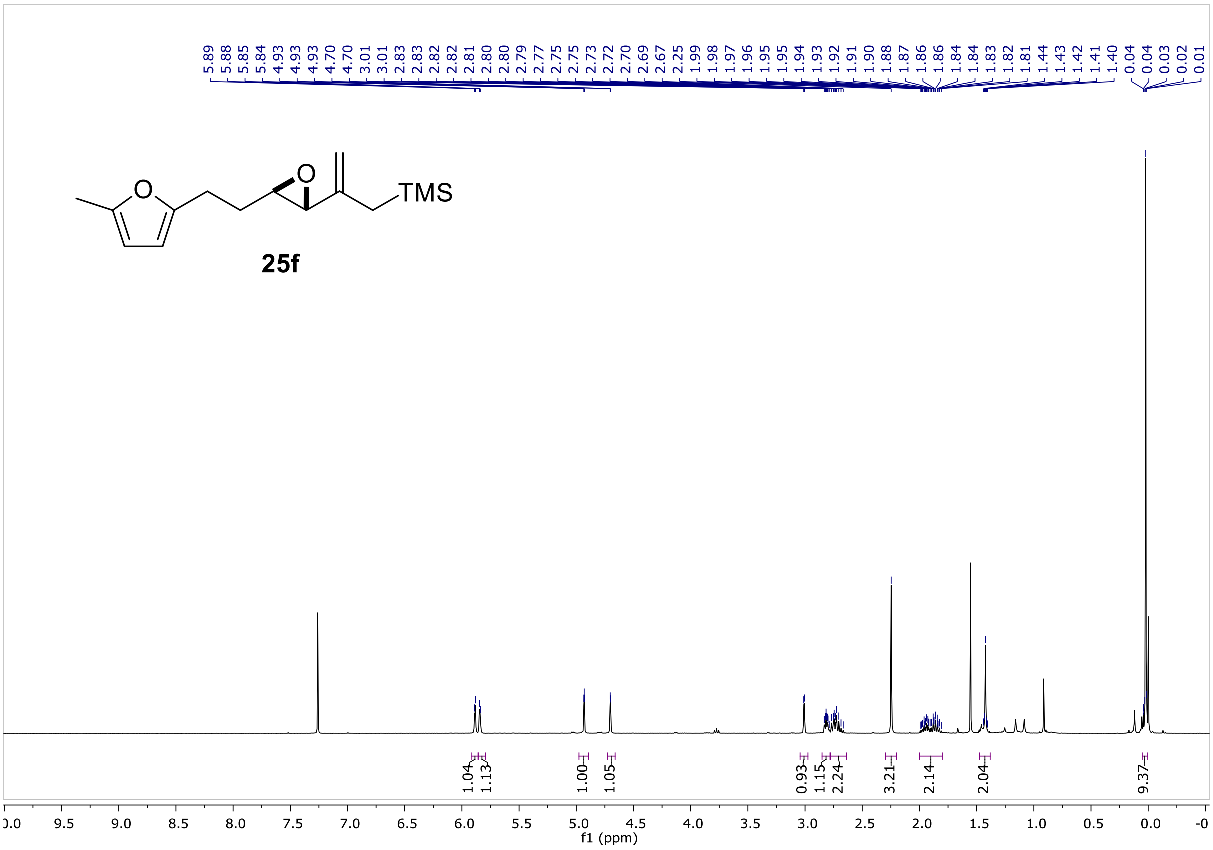

$^{13}C\{^{1}H\}$ NMR (150 MHz, $CDCl_3$): **25f**

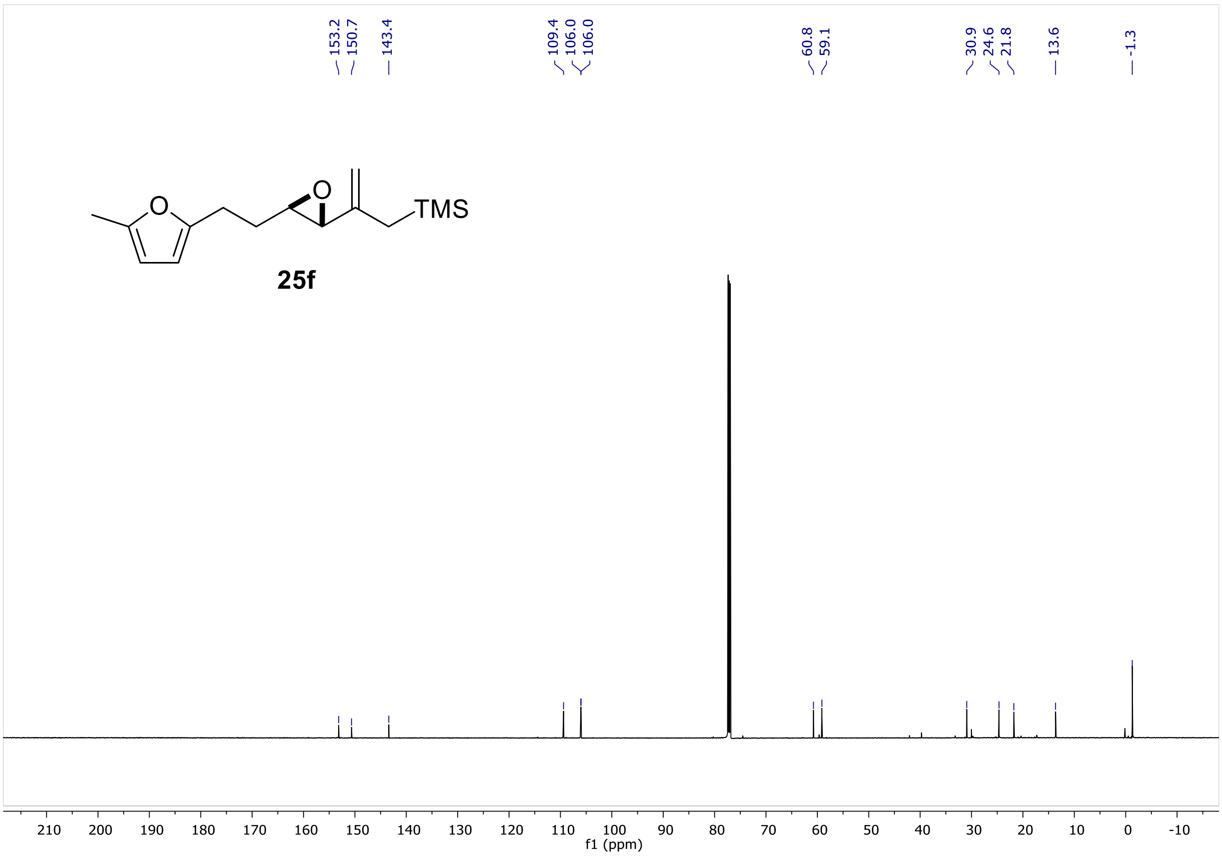

^{1}H NMR (500 MHz, $CDCl_3$): **25g**

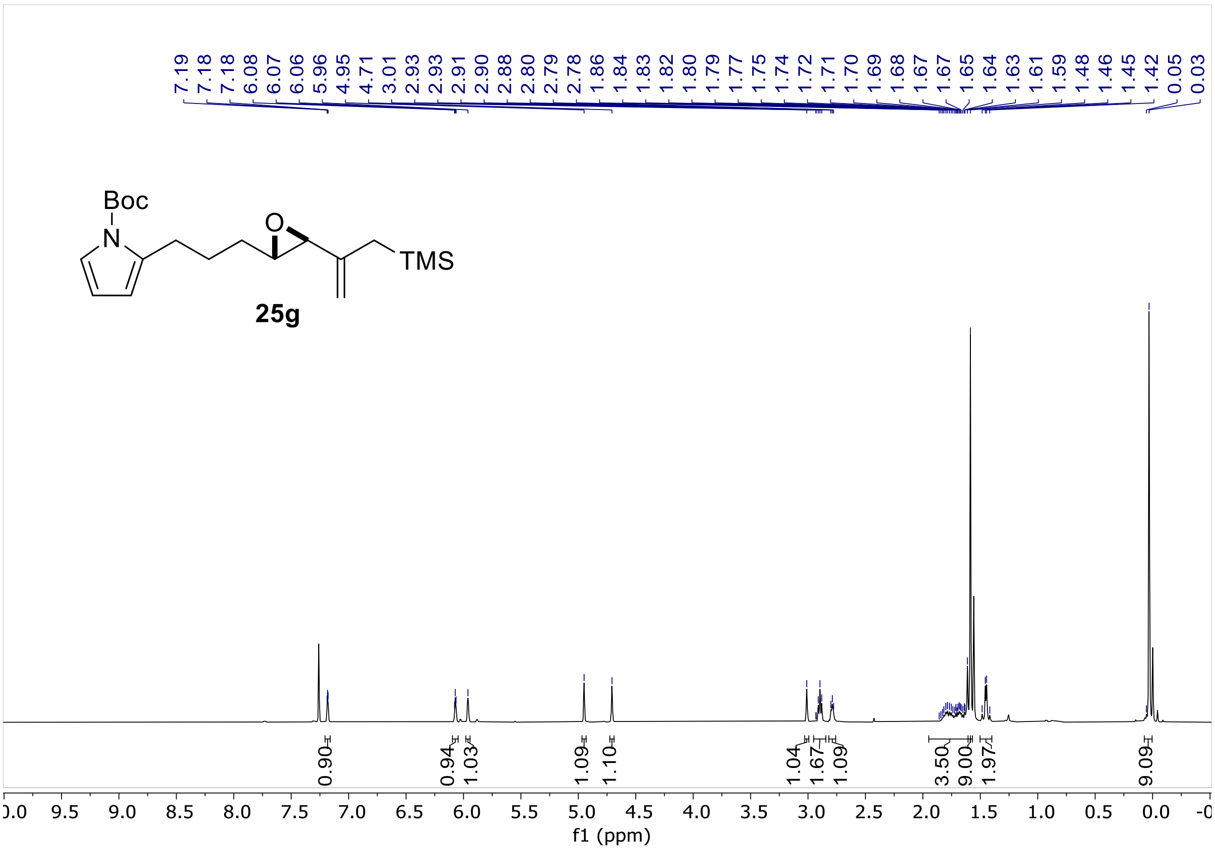

$^{13}C\{^{1}H\}$ NMR (100 MHz, $CDCl_3$): **25g**

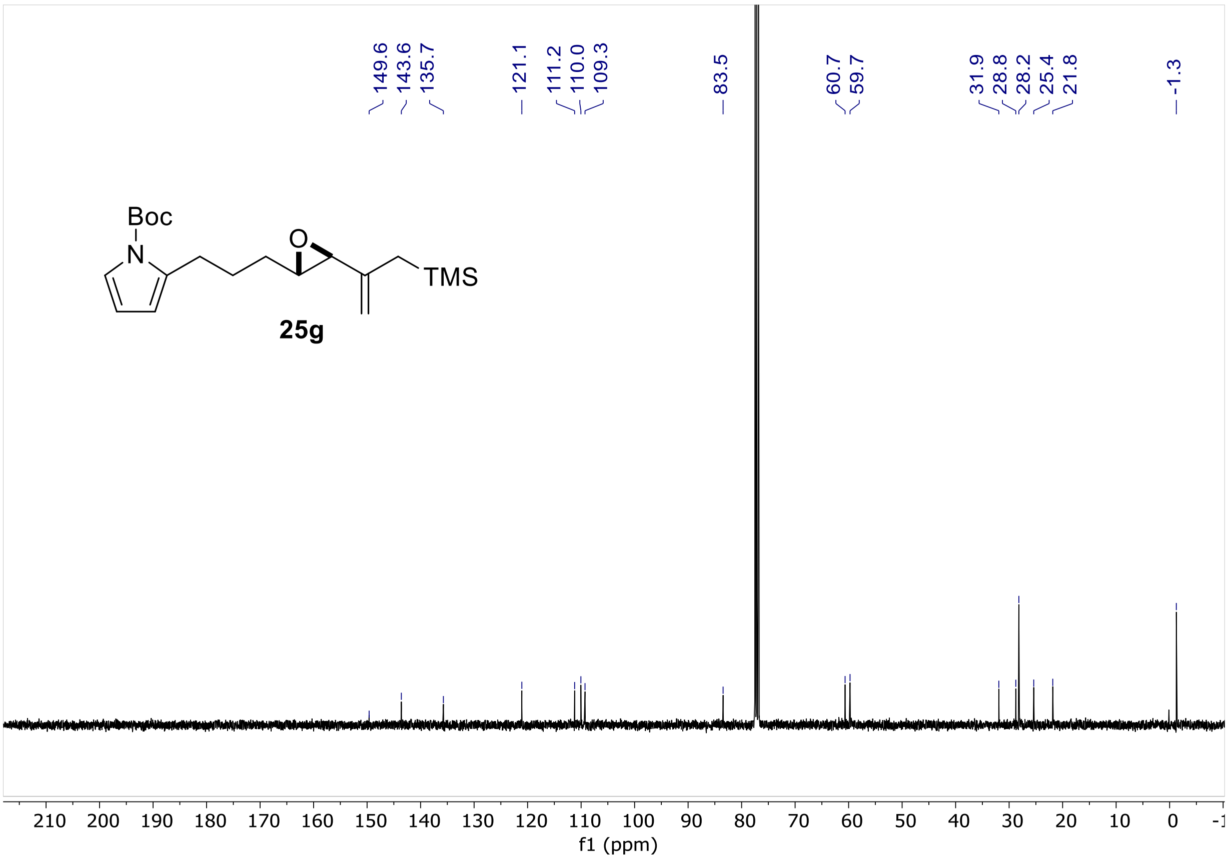

^{1}H NMR (500 MHz, $CDCl_3$): **25h**

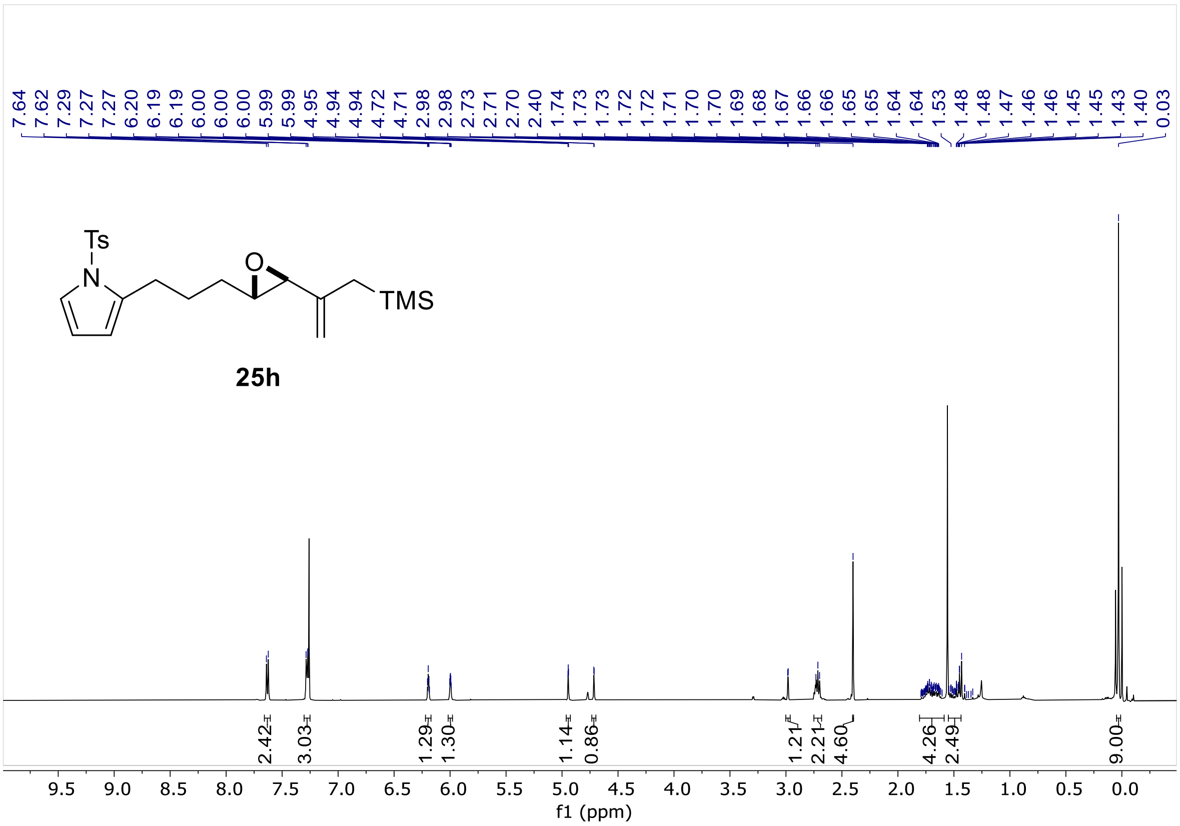

$^{13}C\{^{1}H\}$ NMR (100 MHz, $CDCl_3$): **25h**

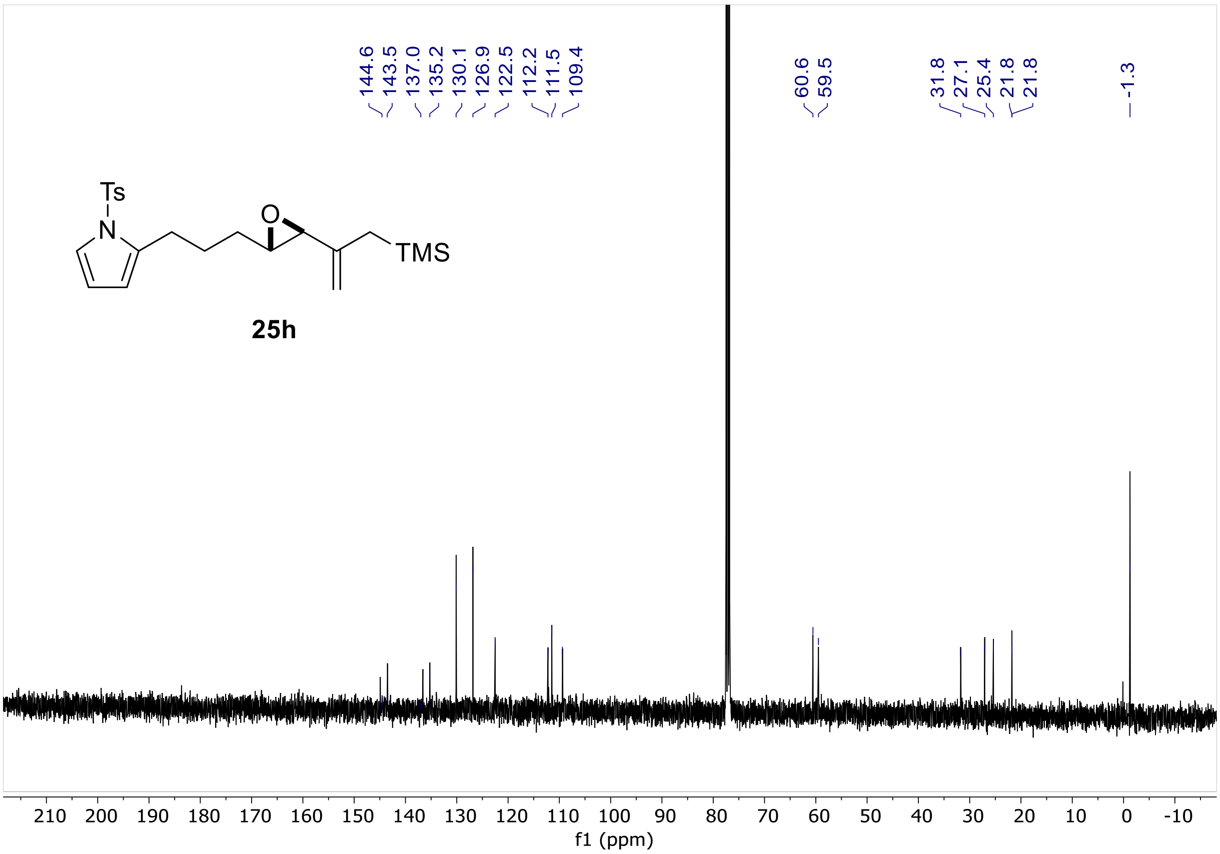

^{1}H NMR (500 MHz, $CDCl_3$): **25i**

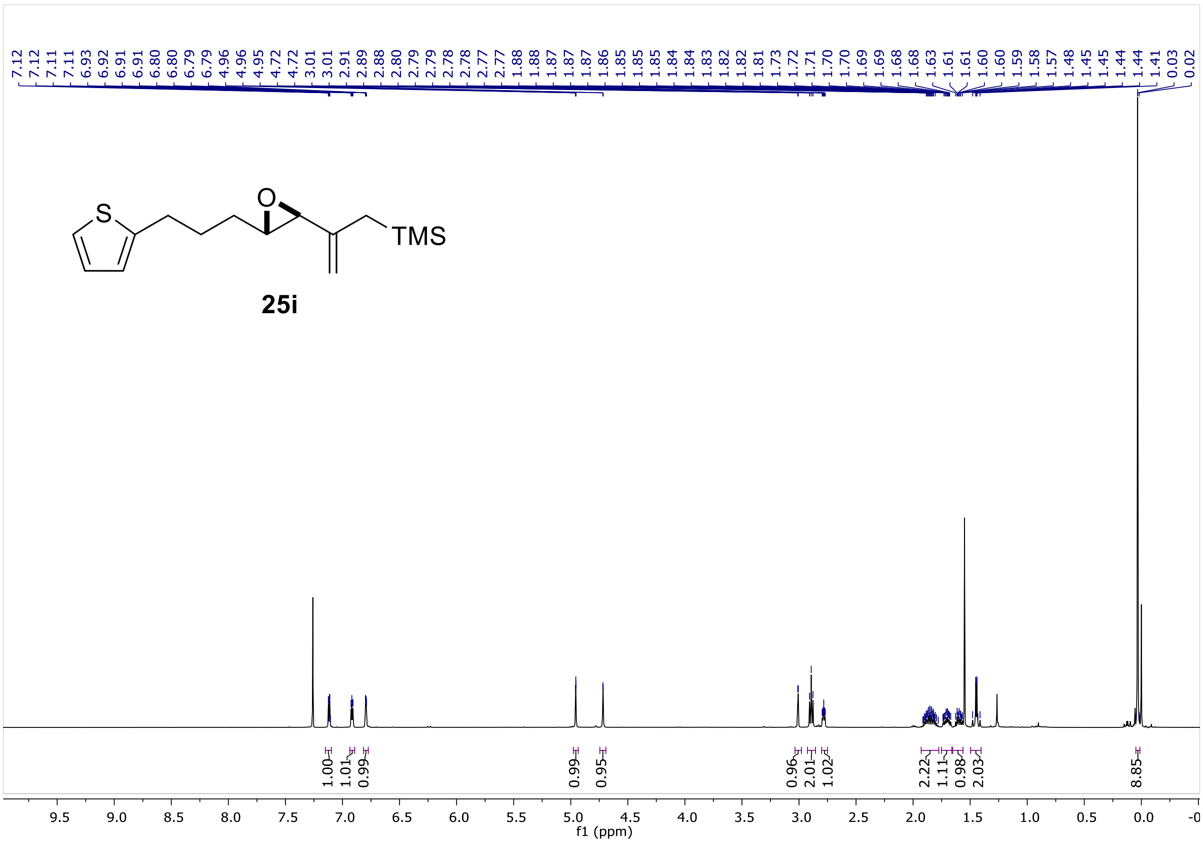

^{13}C{^{1}H} NMR (125 MHz, $CDCl_3$): **25i**

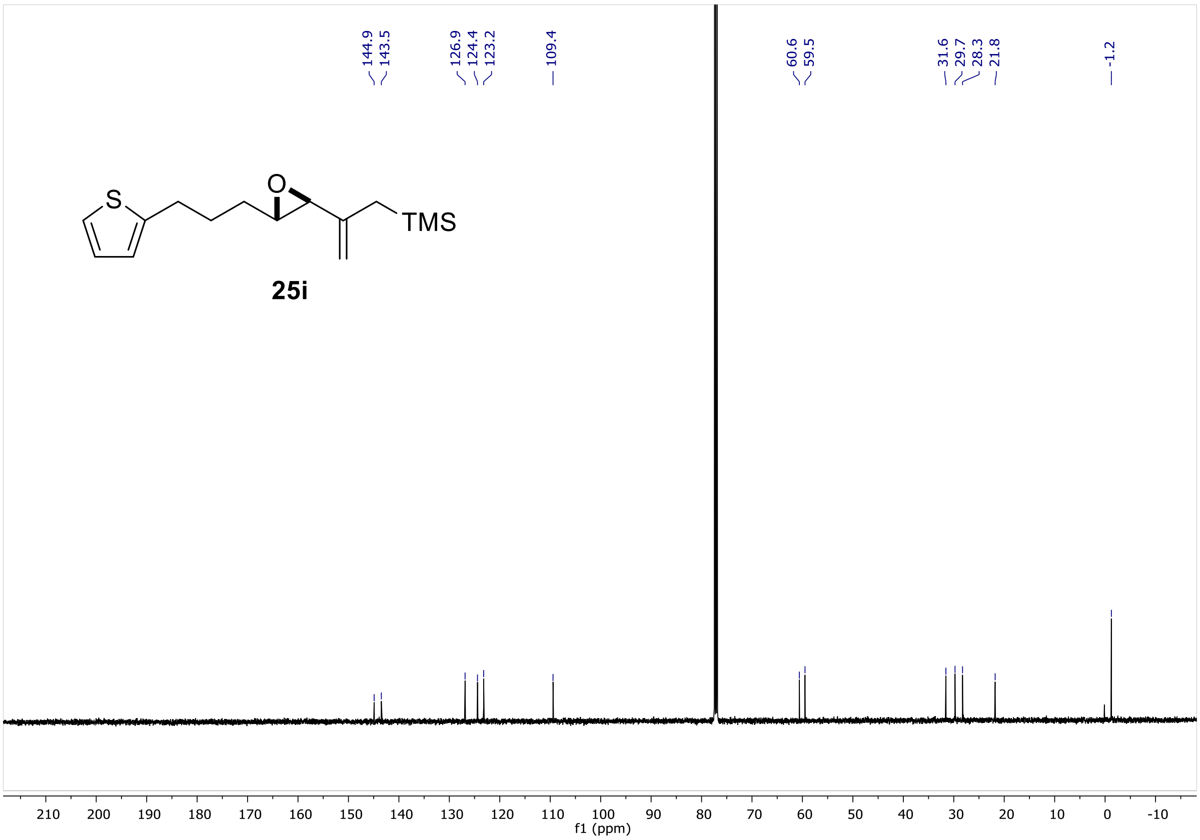

^{1}H NMR (500 MHz, $CDCl_3$): **25j**

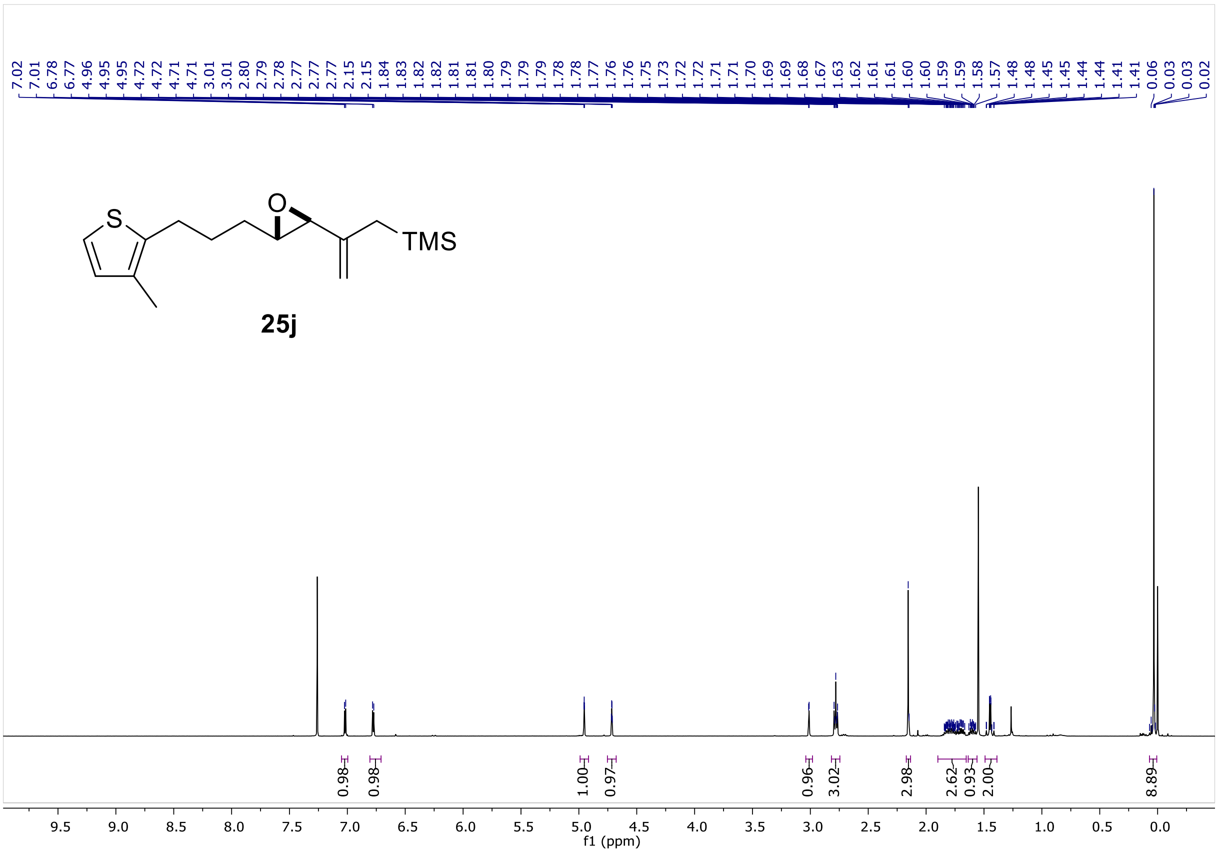

^{13}C{^{1}H} NMR (150 MHz, $CDCl_3$): **25j**

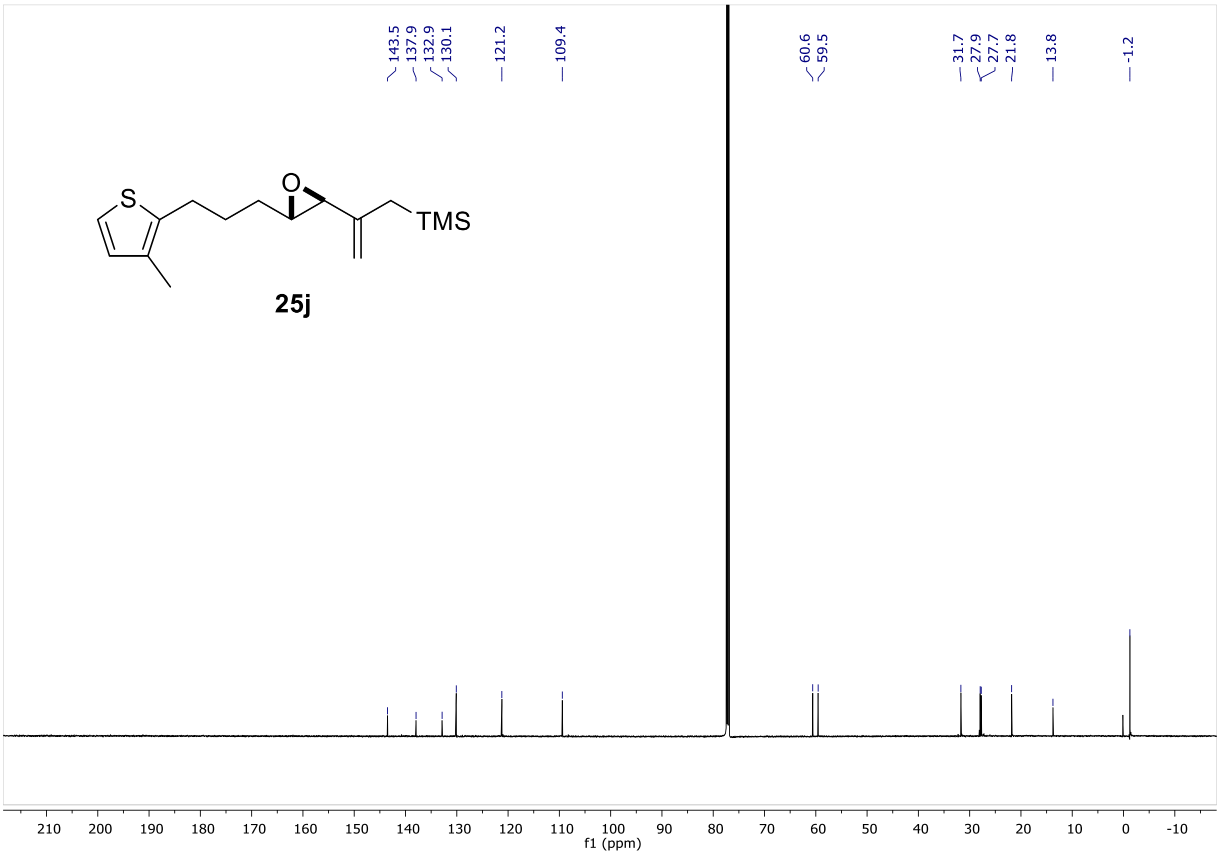

^{1}H NMR (400 MHz, $CDCl_3$): **25k**

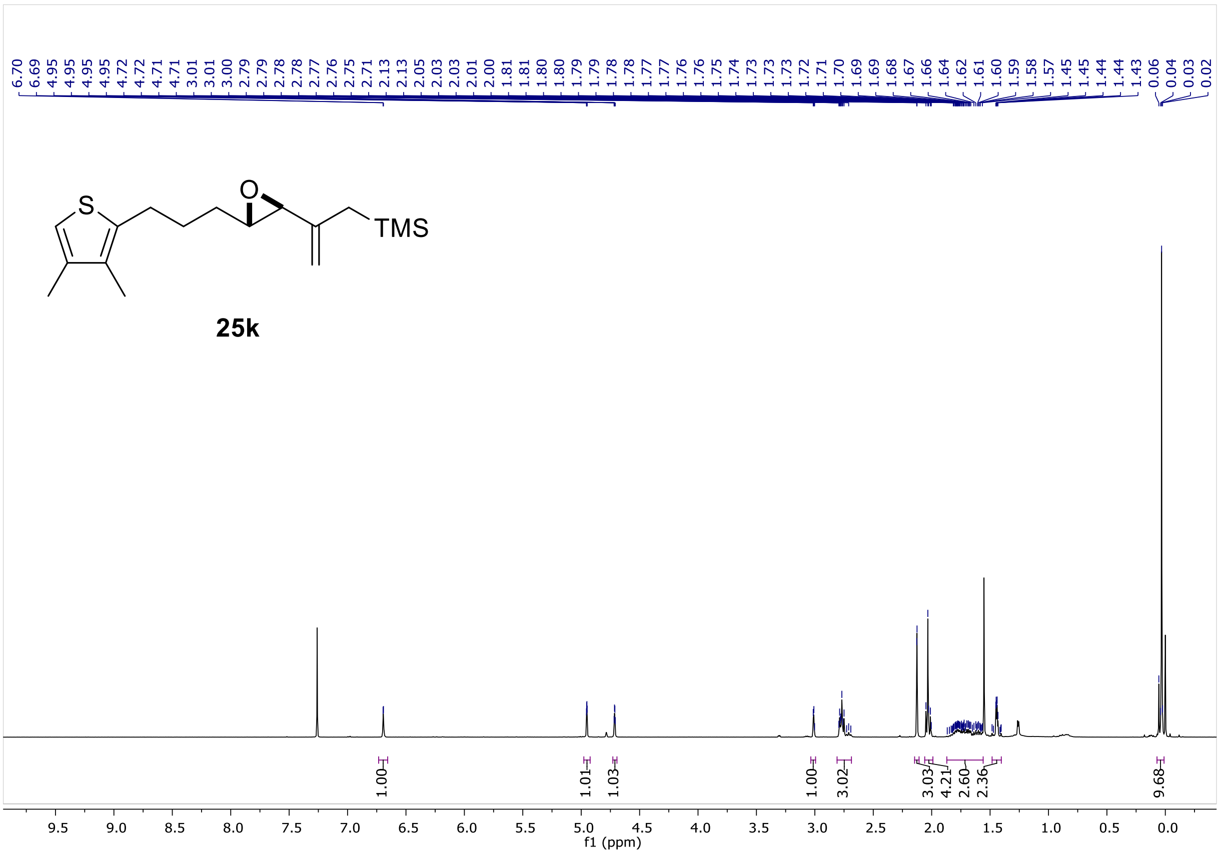

^{13}C{^{1}H} NMR (150 MHz, $CDCl_3$): **25k**

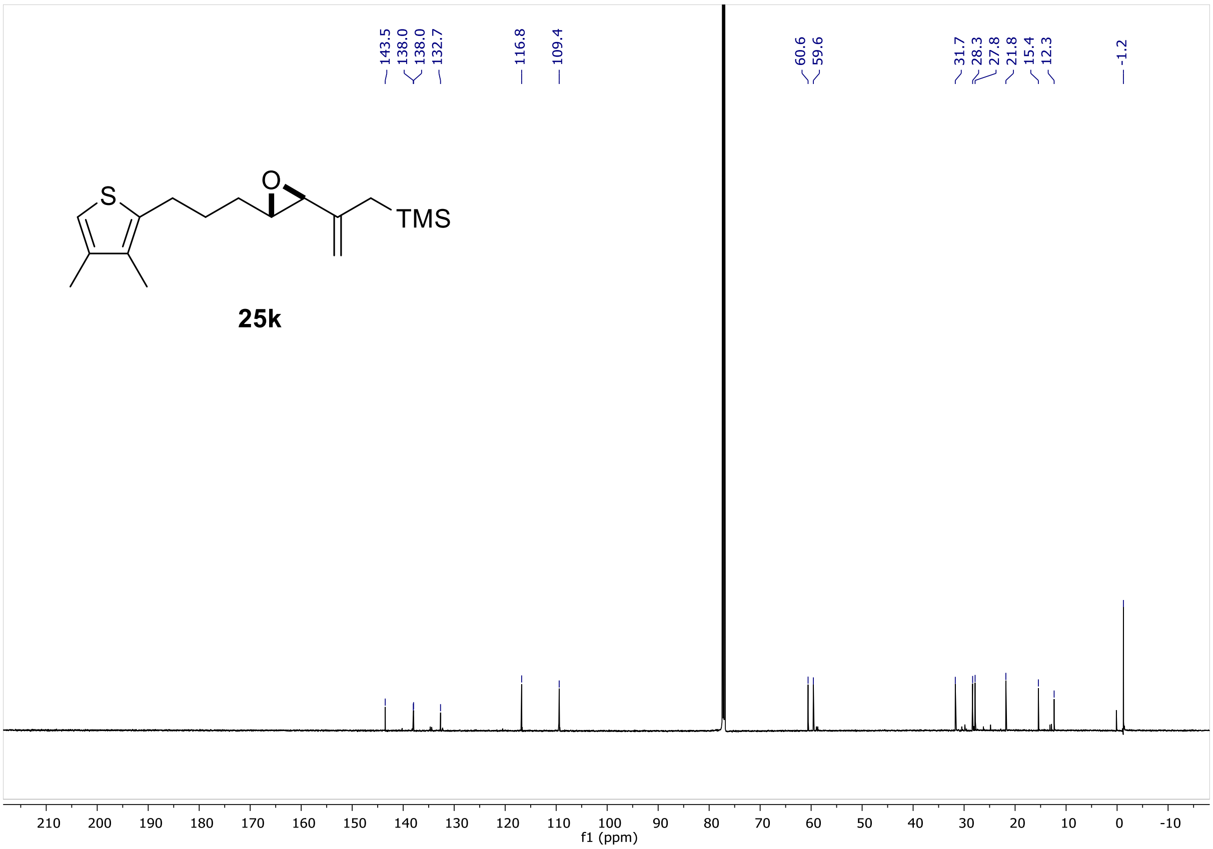

^{1}H NMR (400 MHz, $CDCl_3$): **41n**

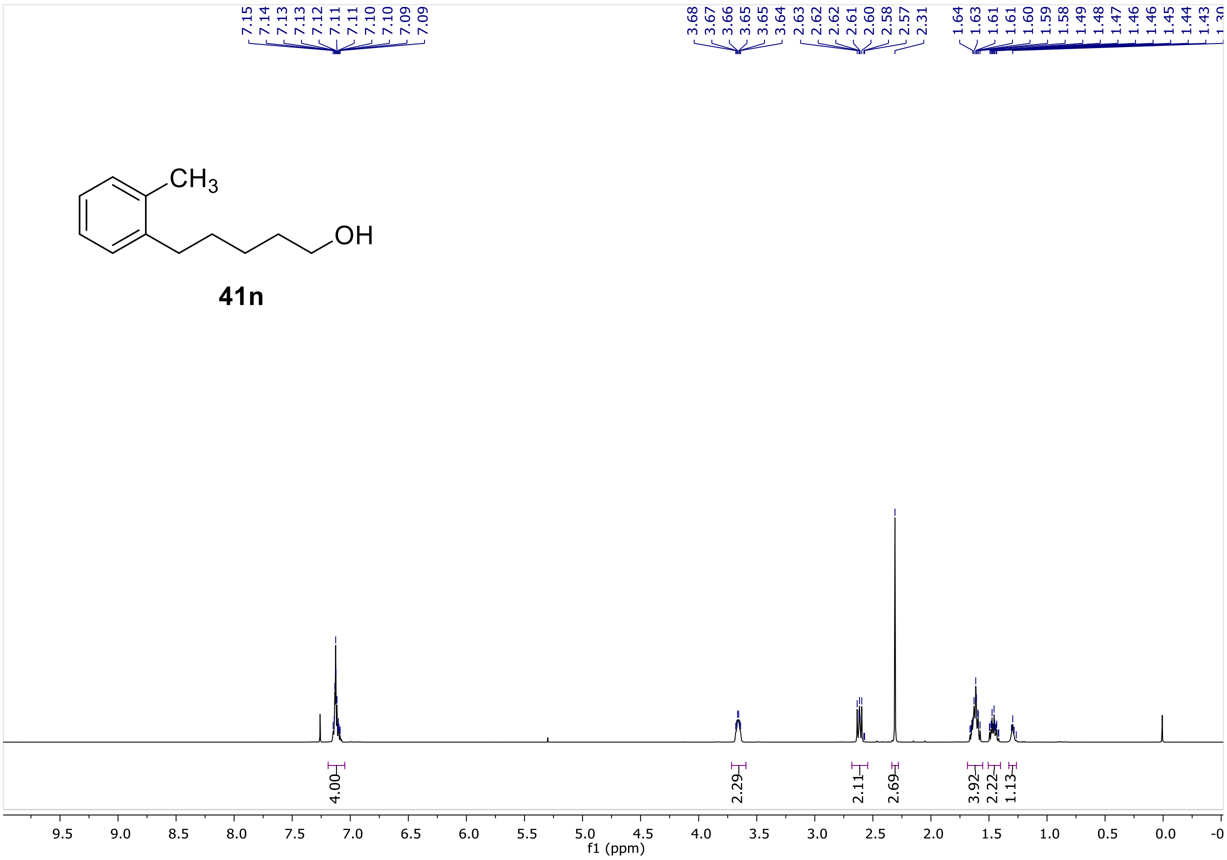

$^{13}C\{^{1}H\}$ NMR (100 MHz, $CDCl_3$): **41n**

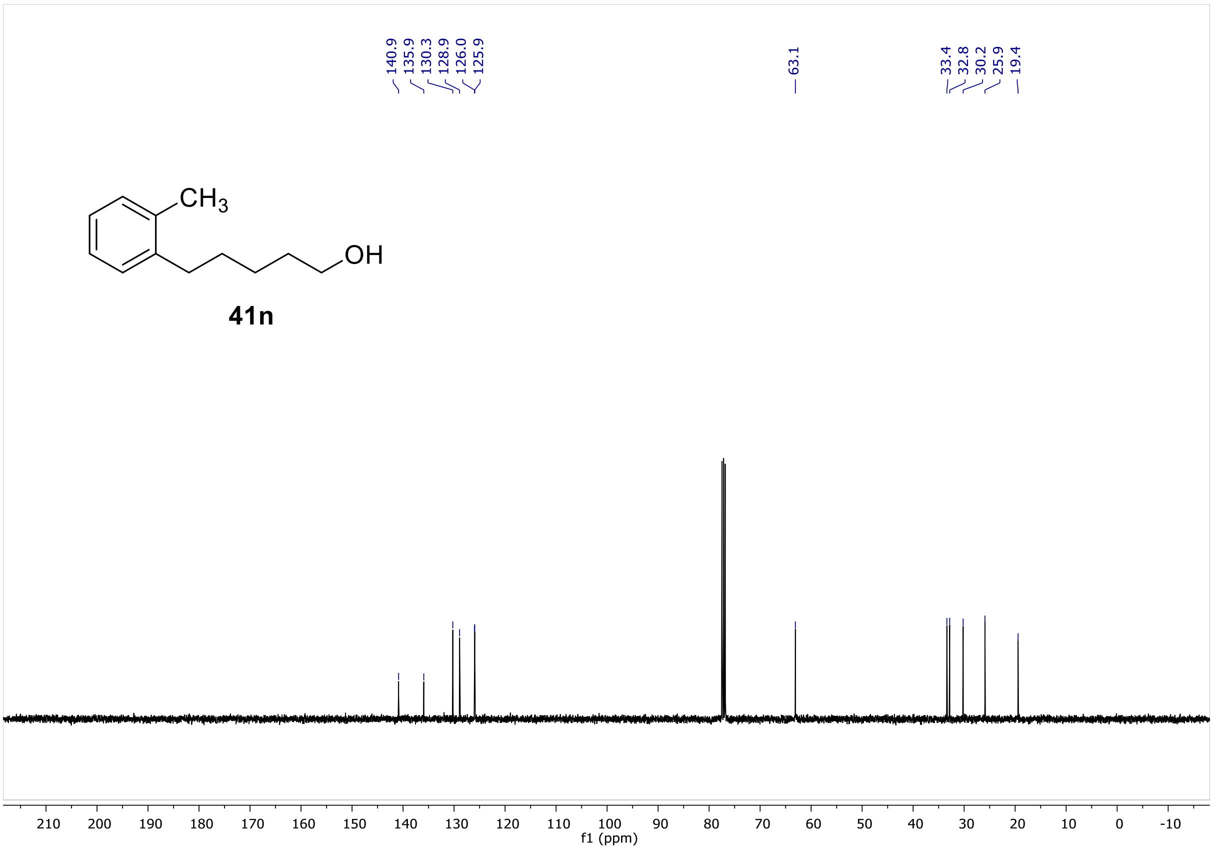

^{1}H NMR (600 MHz, $CDCl_3$): **41o**

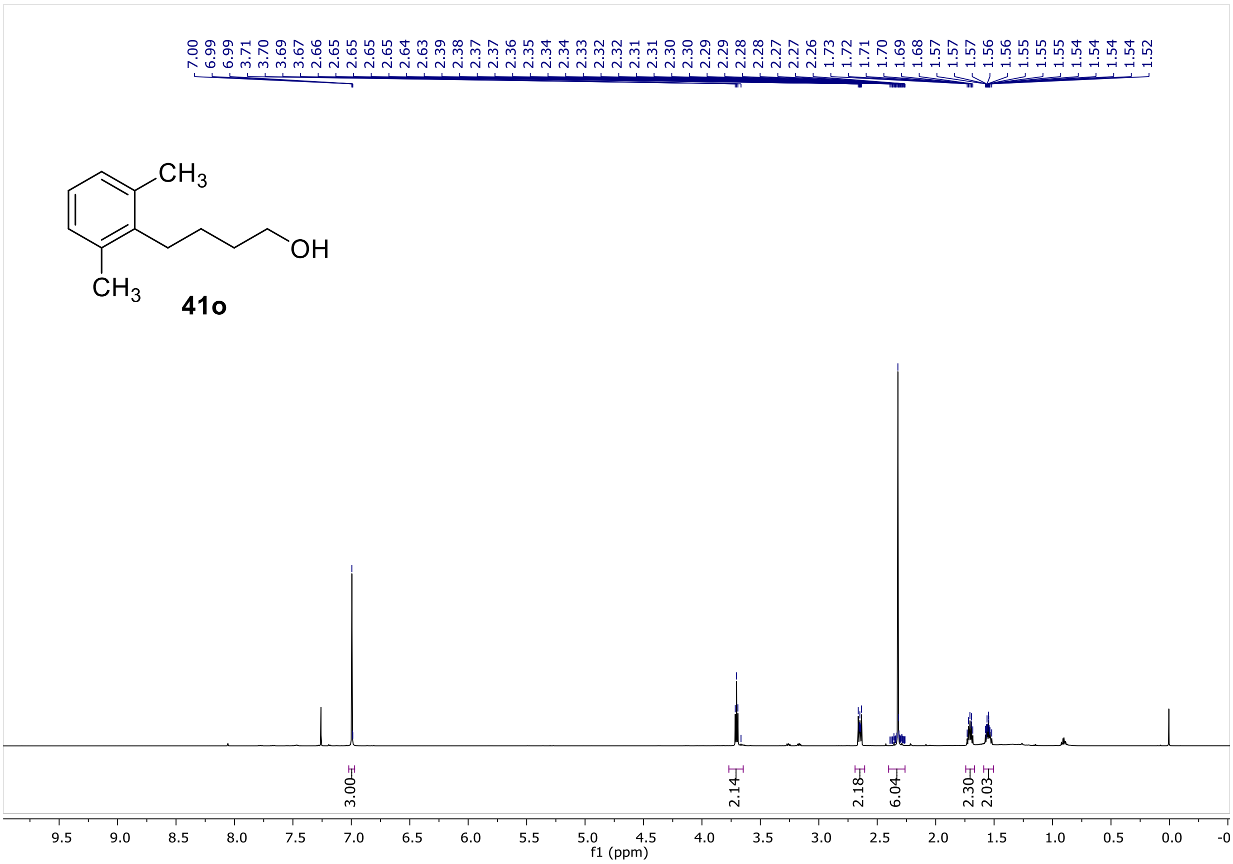

^{13}C{^{1}H} NMR (150 MHz, $CDCl_3$): **41o**

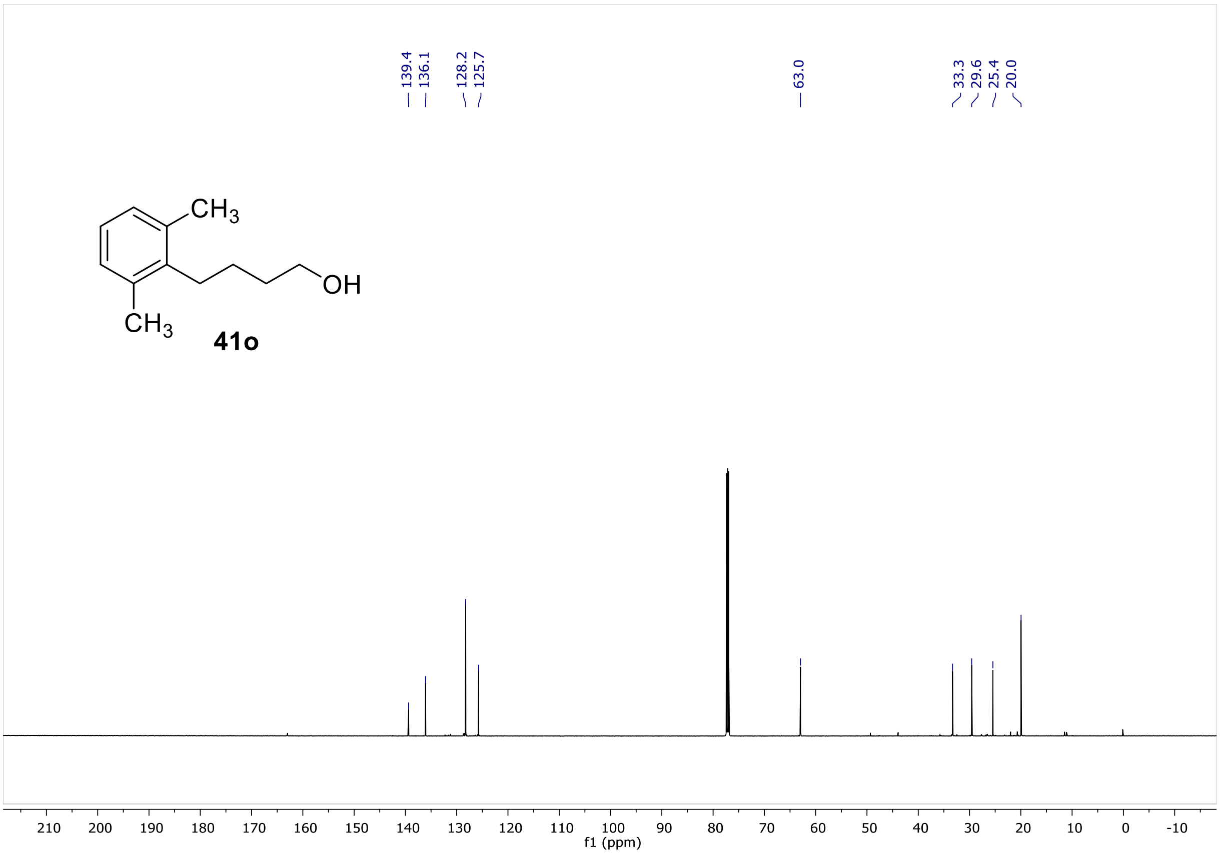

^{1}H NMR (400 MHz, $CDCl_3$): **26n**

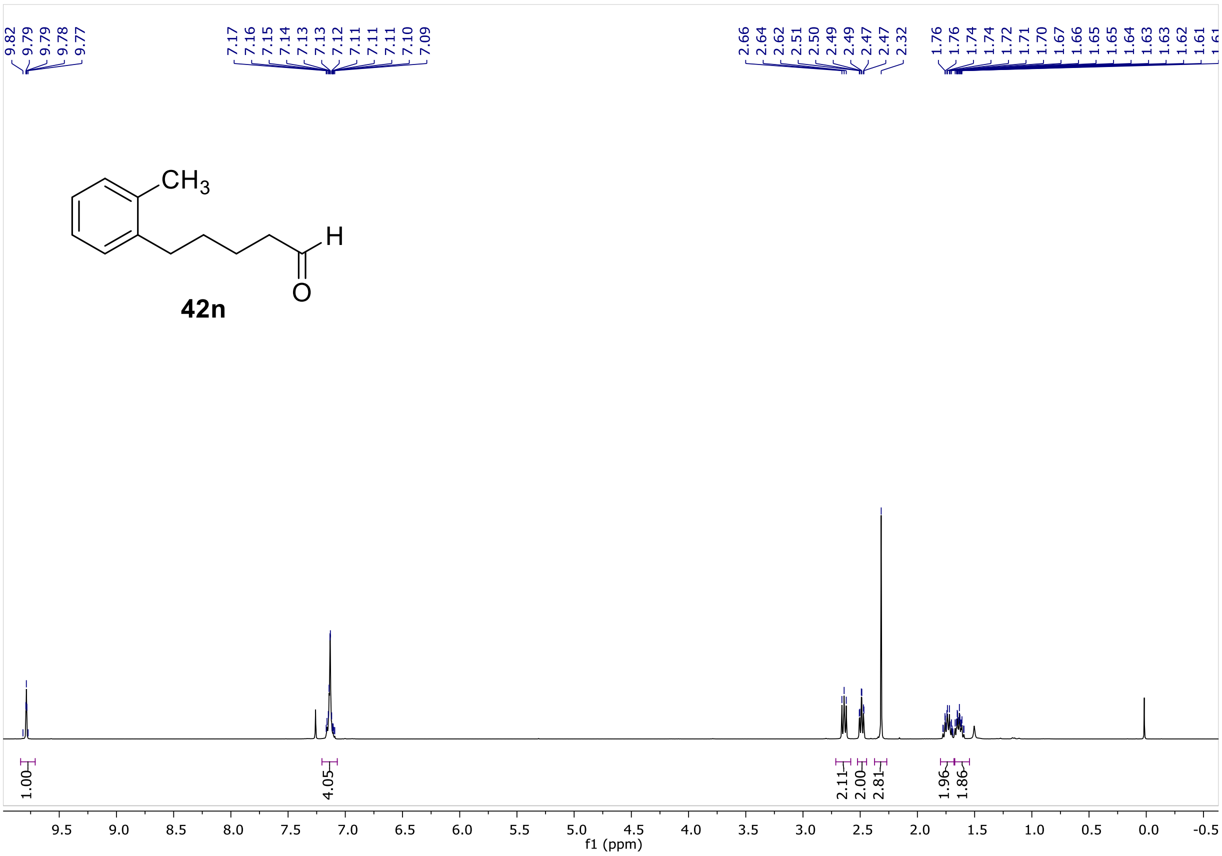

$^{13}C\{^{1}H\}$ NMR (100 MHz, $CDCl_3$): **42n**

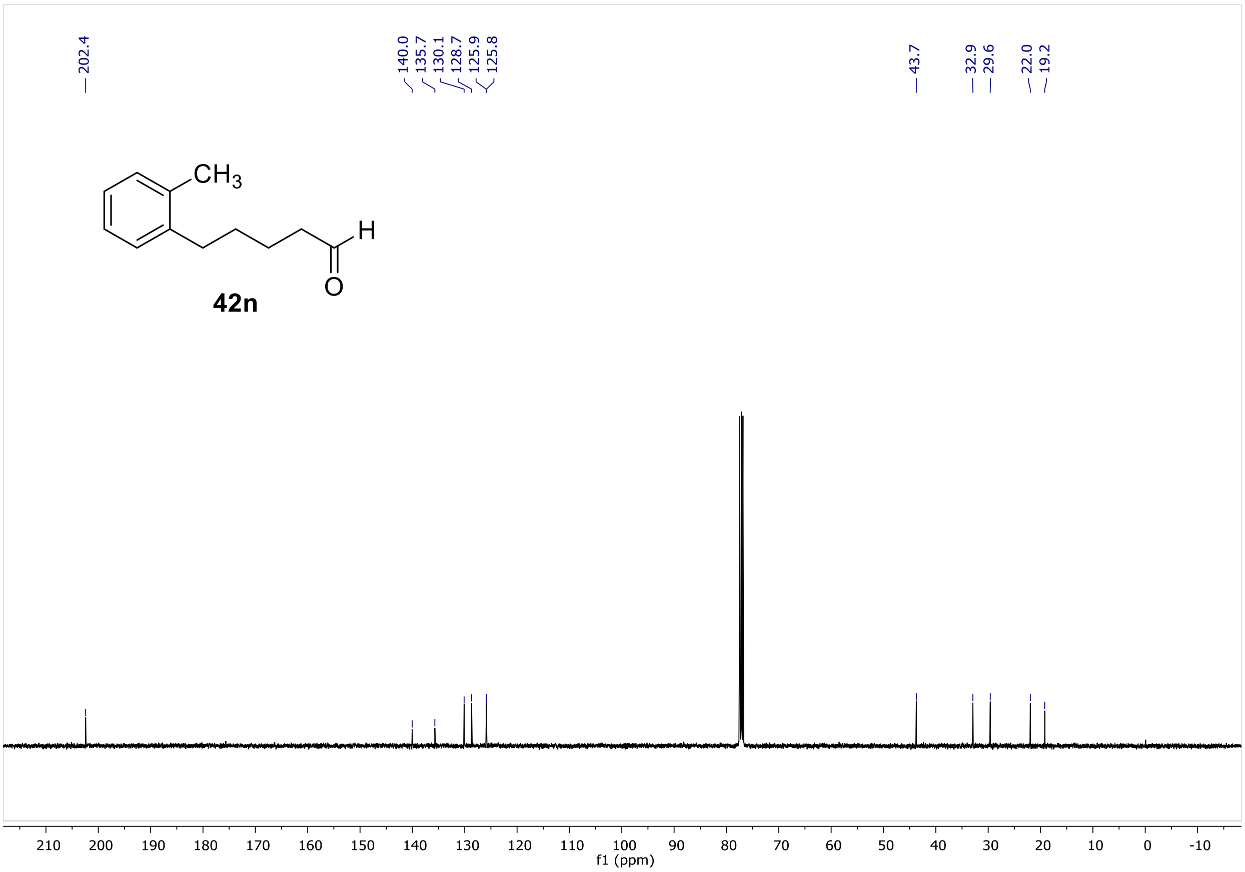

^{1}H NMR (500 MHz, $CDCl_3$): **42o**

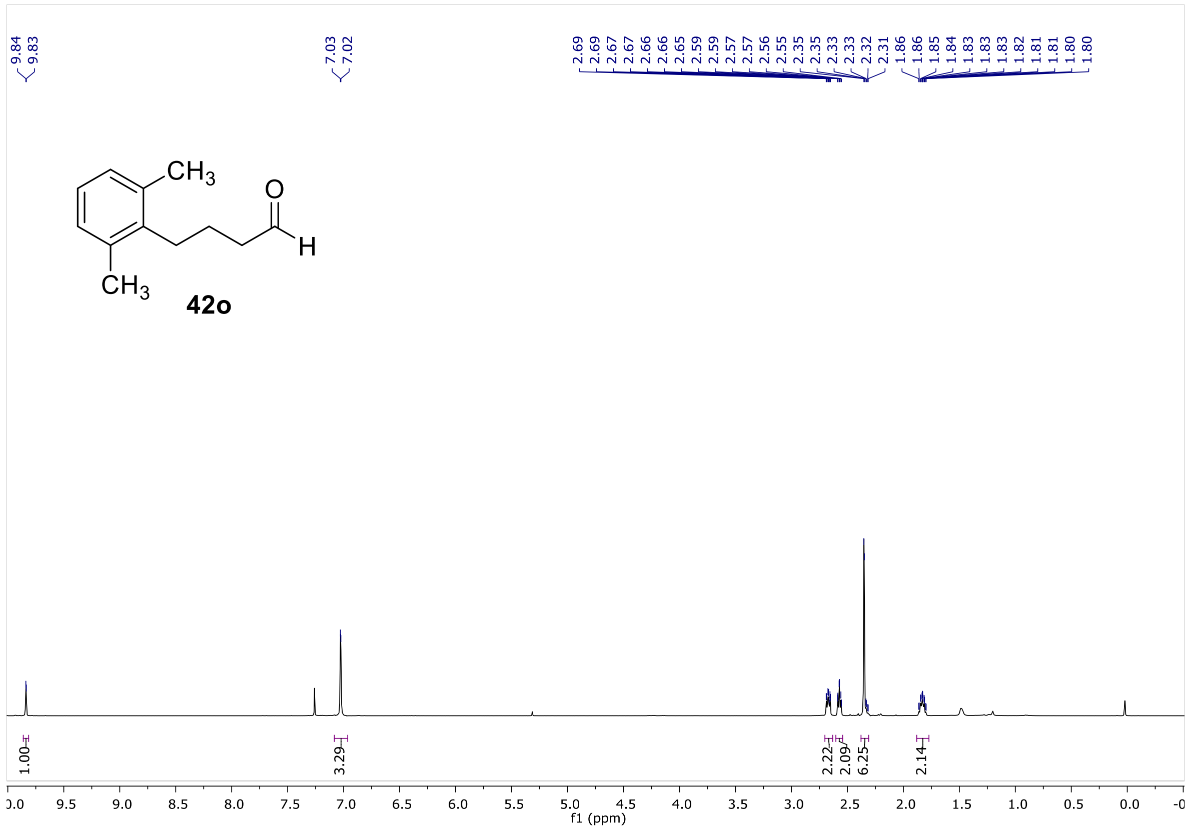

$^{13}C\{^{1}H\}$ NMR (125 MHz, $CDCl_3$): **42o**

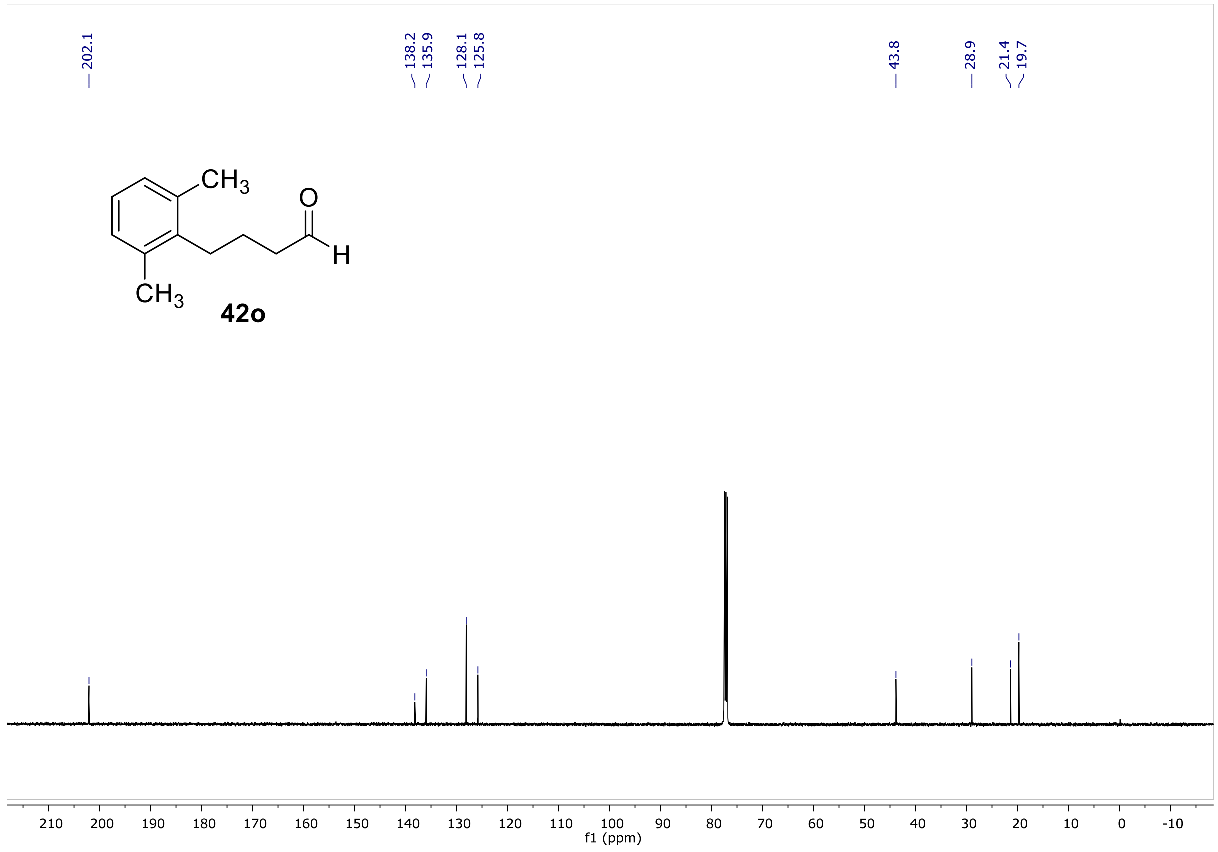

^{1}H NMR (400 MHz, $CDCl_3$): **42r**

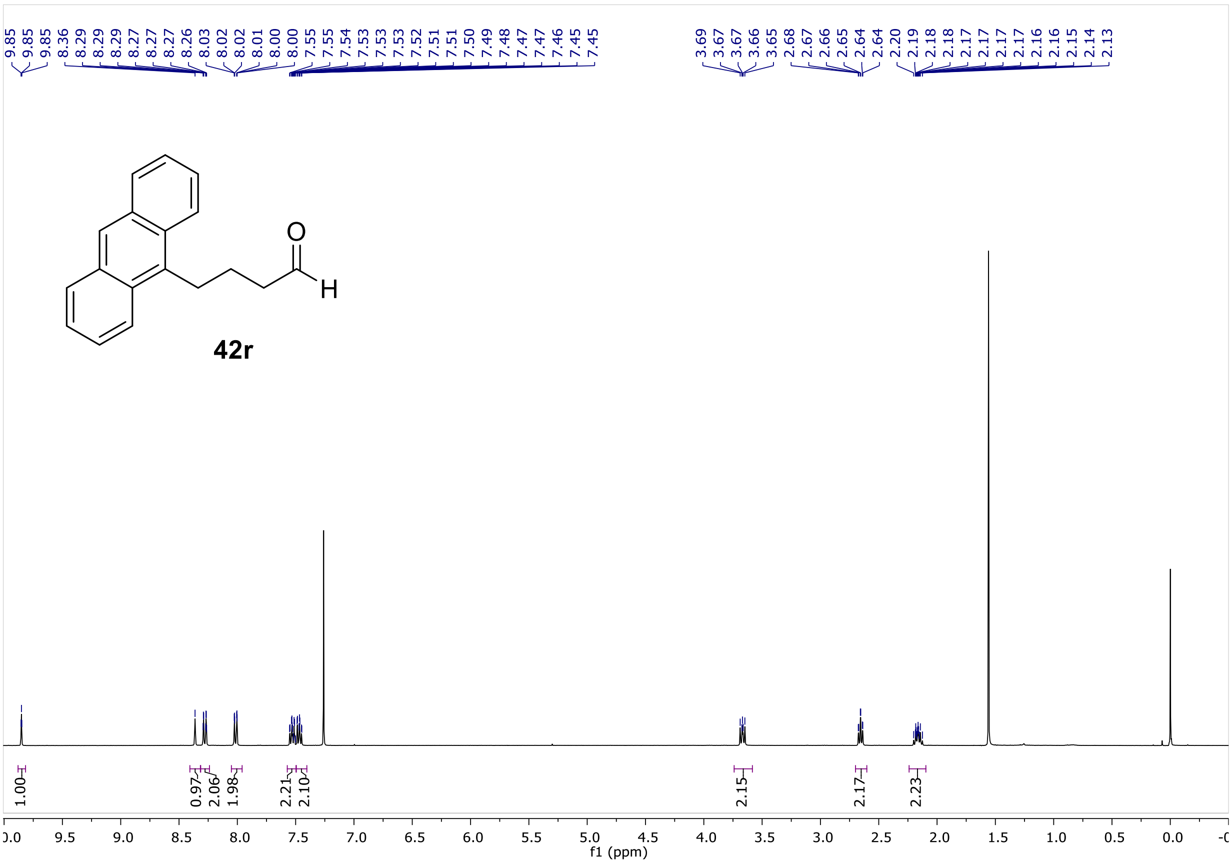

$^{13}C\{^{1}H\}$ NMR (100 MHz, $CDCl_3$): **42r**

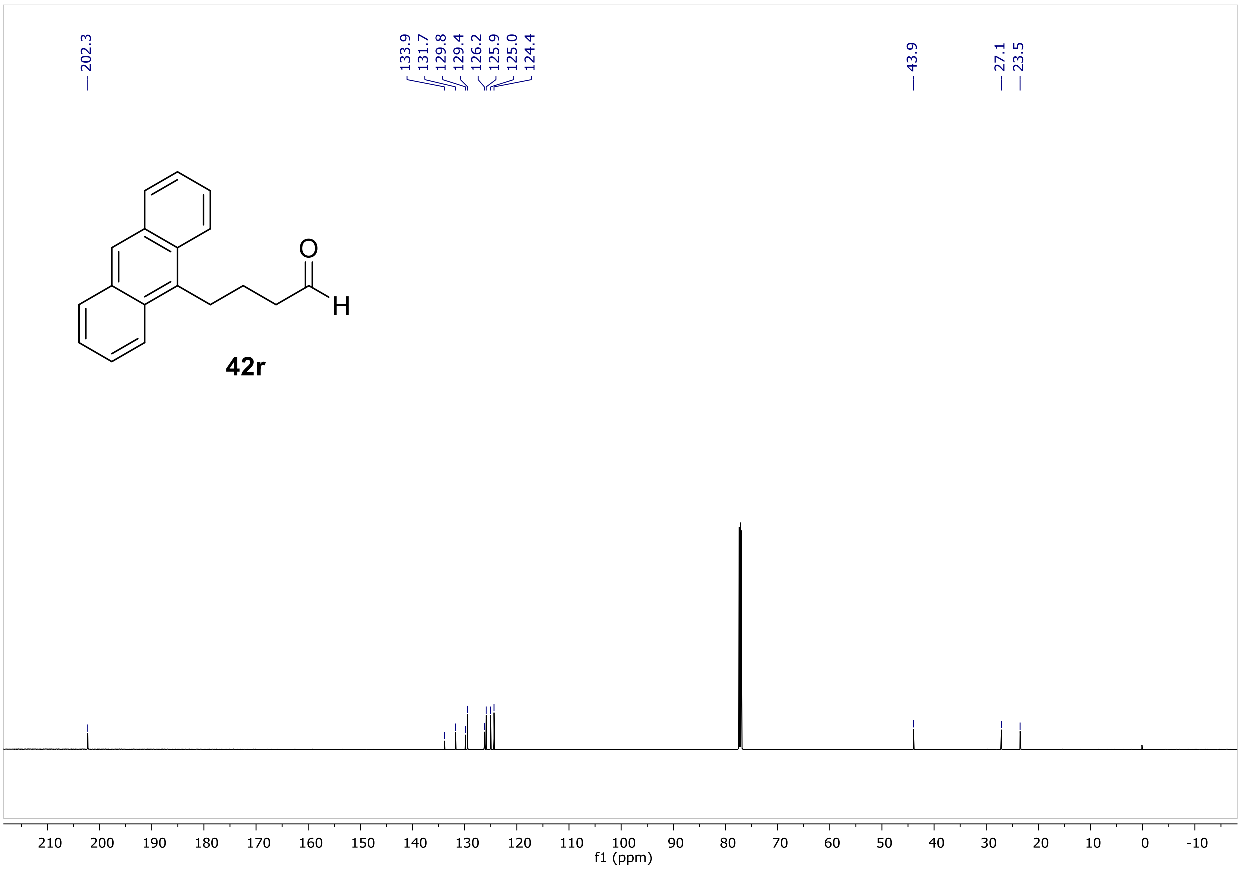

^{1}H NMR (400 MHz, $CDCl_3$): **43n**

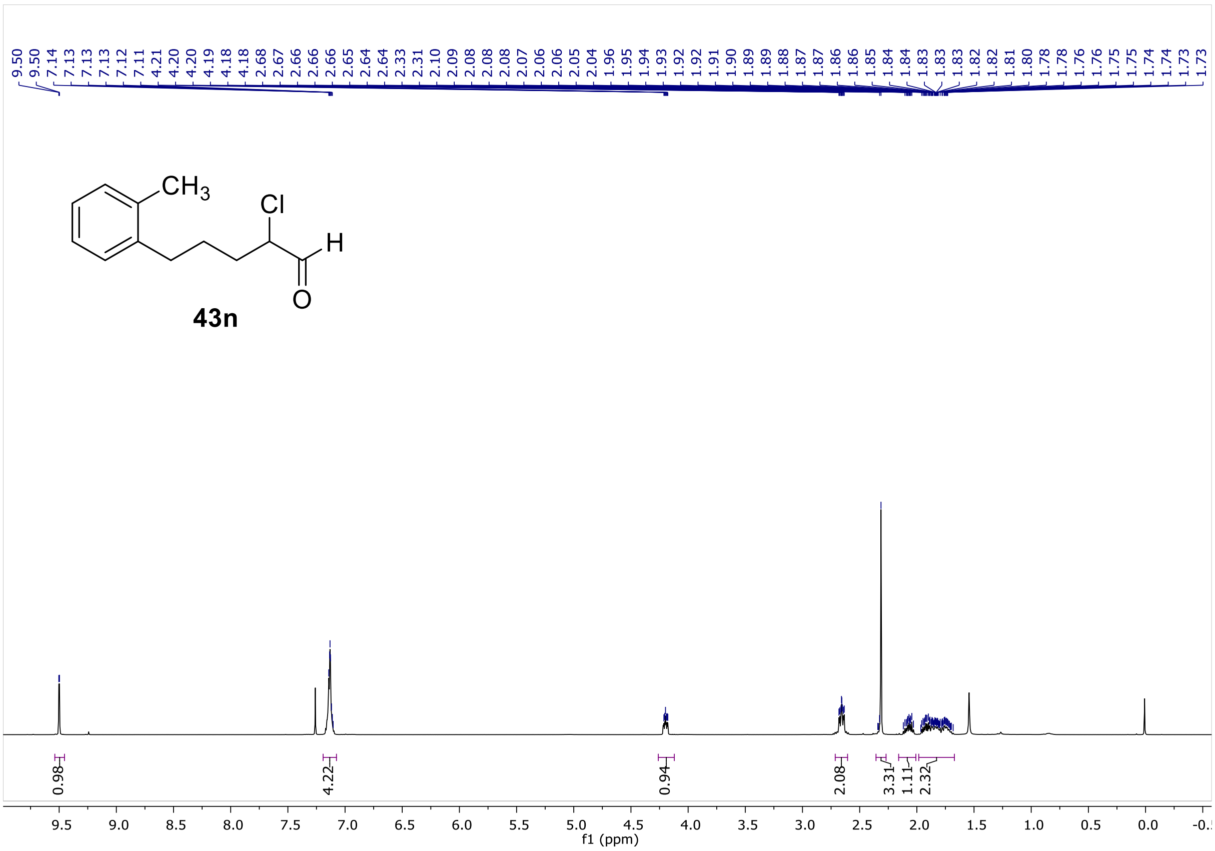

^{13}C{^{1}H} NMR (100 MHz, $CDCl_3$): **43n**

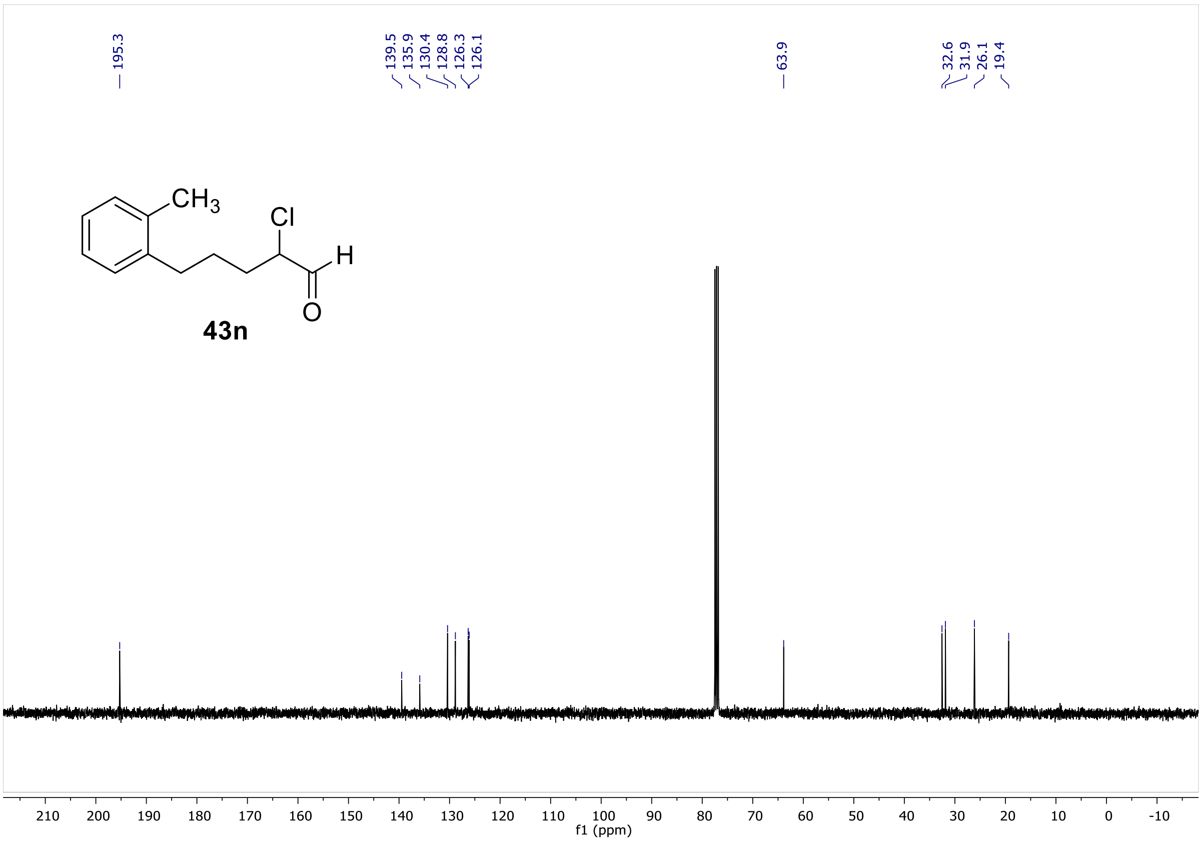

^{1}H NMR (500 MHz, $CDCl_3$): **43o**

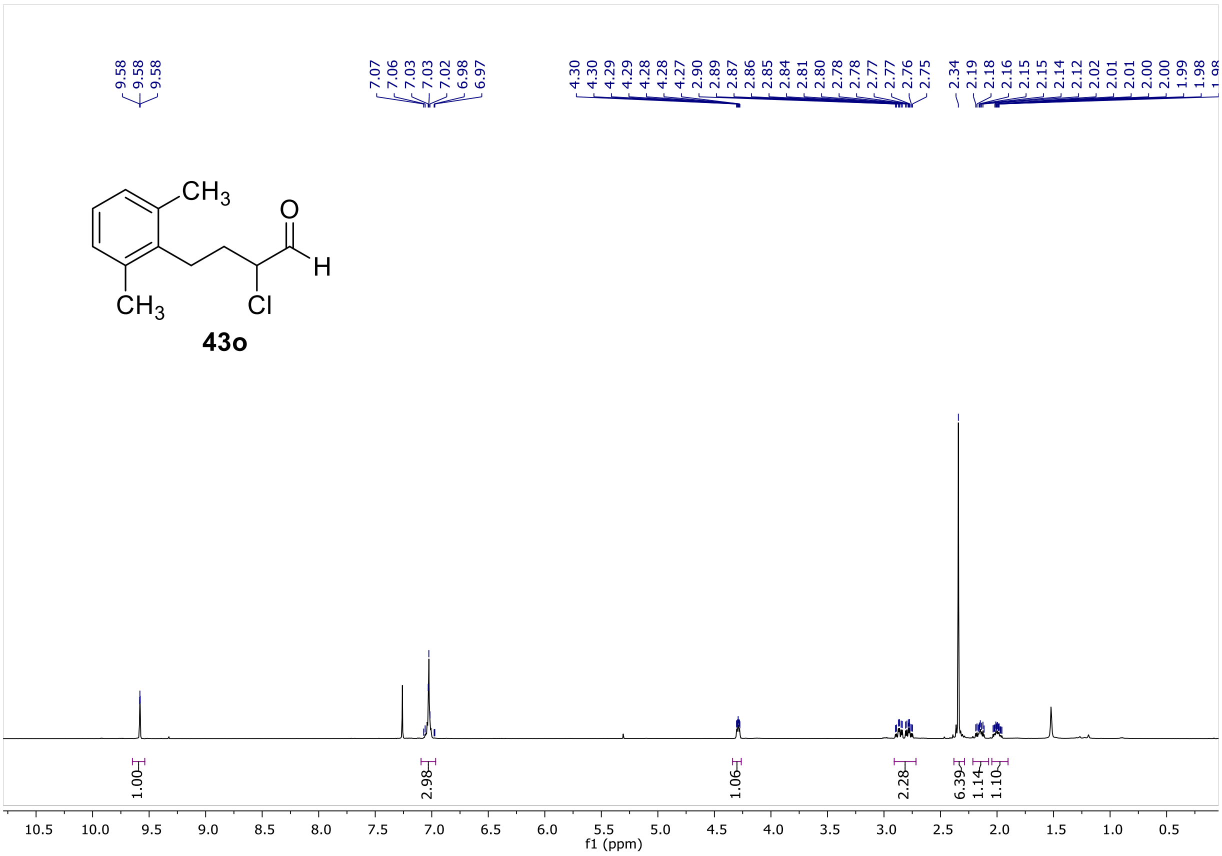

^{13}C{^{1}H} NMR (125 MHz, $CDCl_3$): **43o**

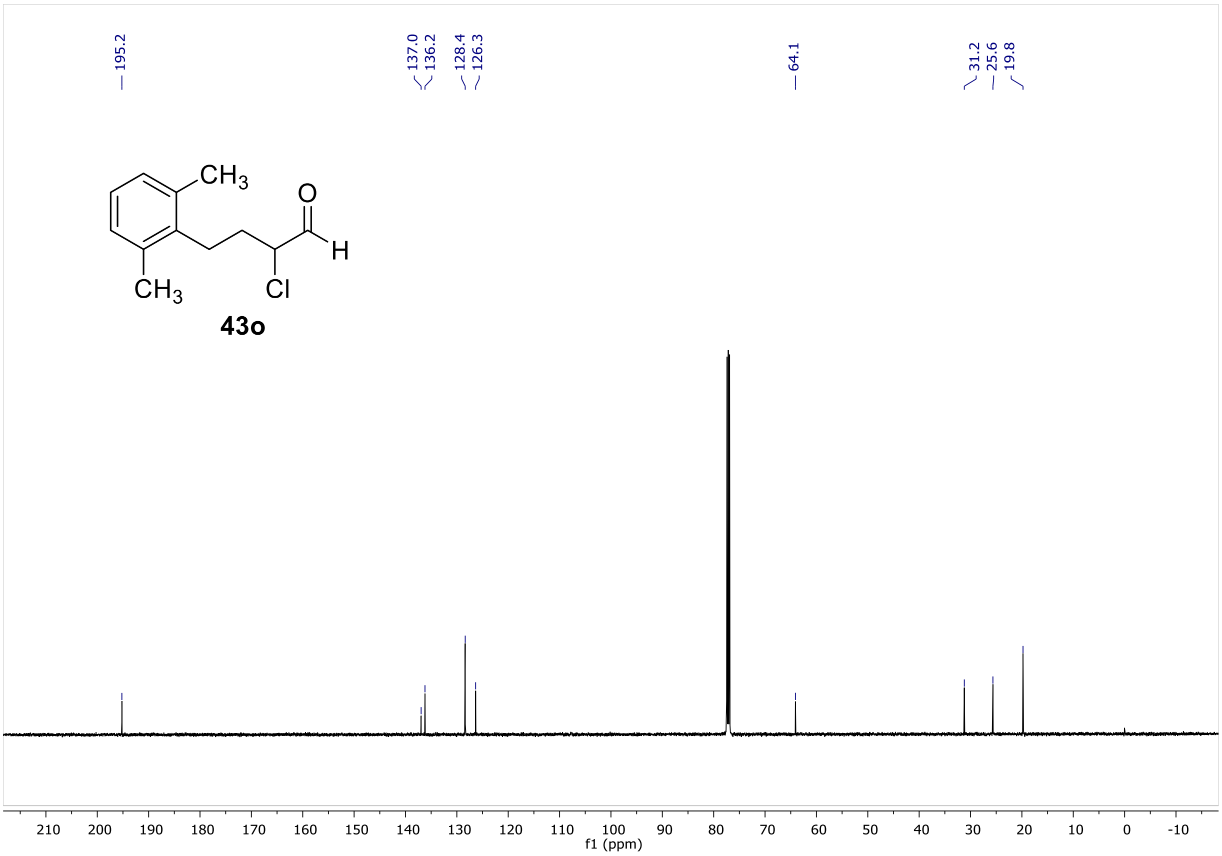

^{1}H NMR (400 MHz, $CDCl_3$): **43p**

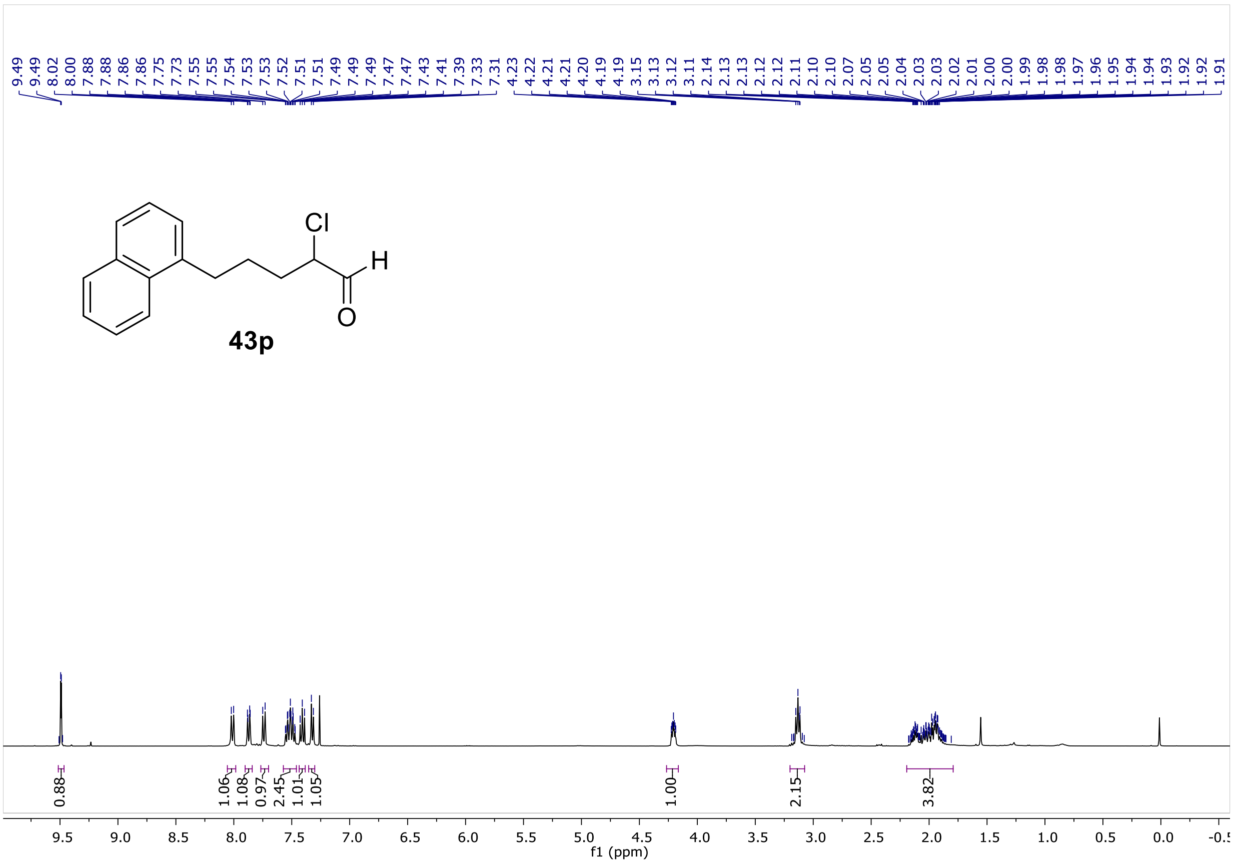

^{13}C{^{1}H} NMR (100 MHz, $CDCl_3$): **43p**

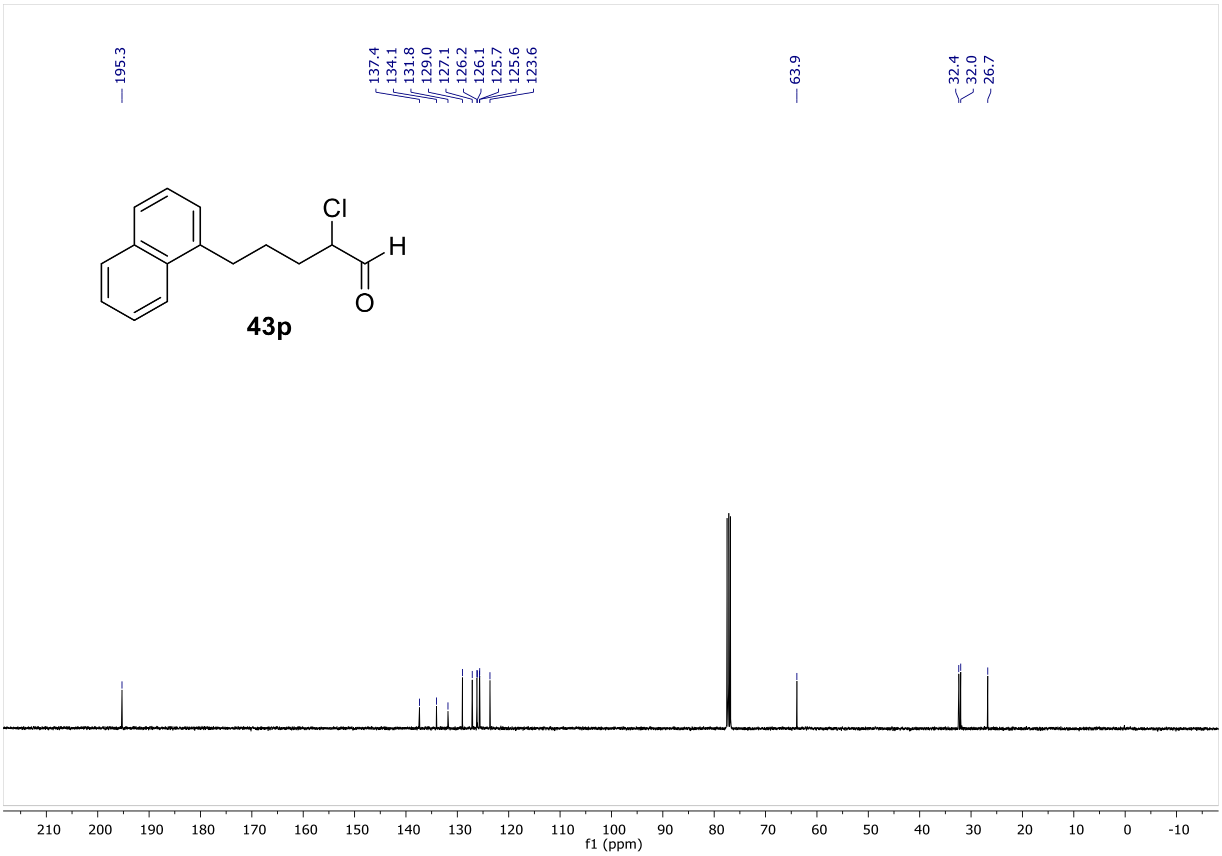

^{1}H NMR (400 MHz, $CDCl_3$): **43q**

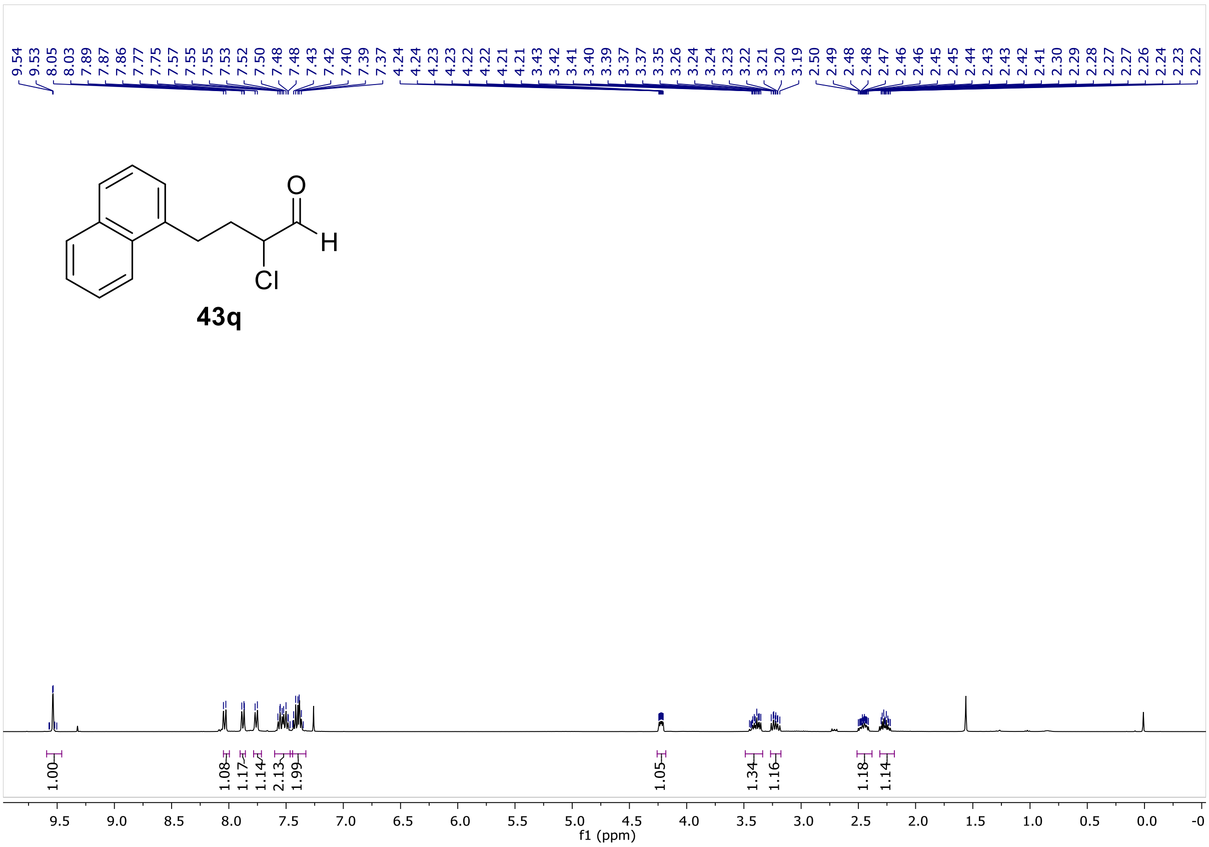

^{13}C{^{1}H} NMR (100 MHz, $CDCl_3$): **43q**

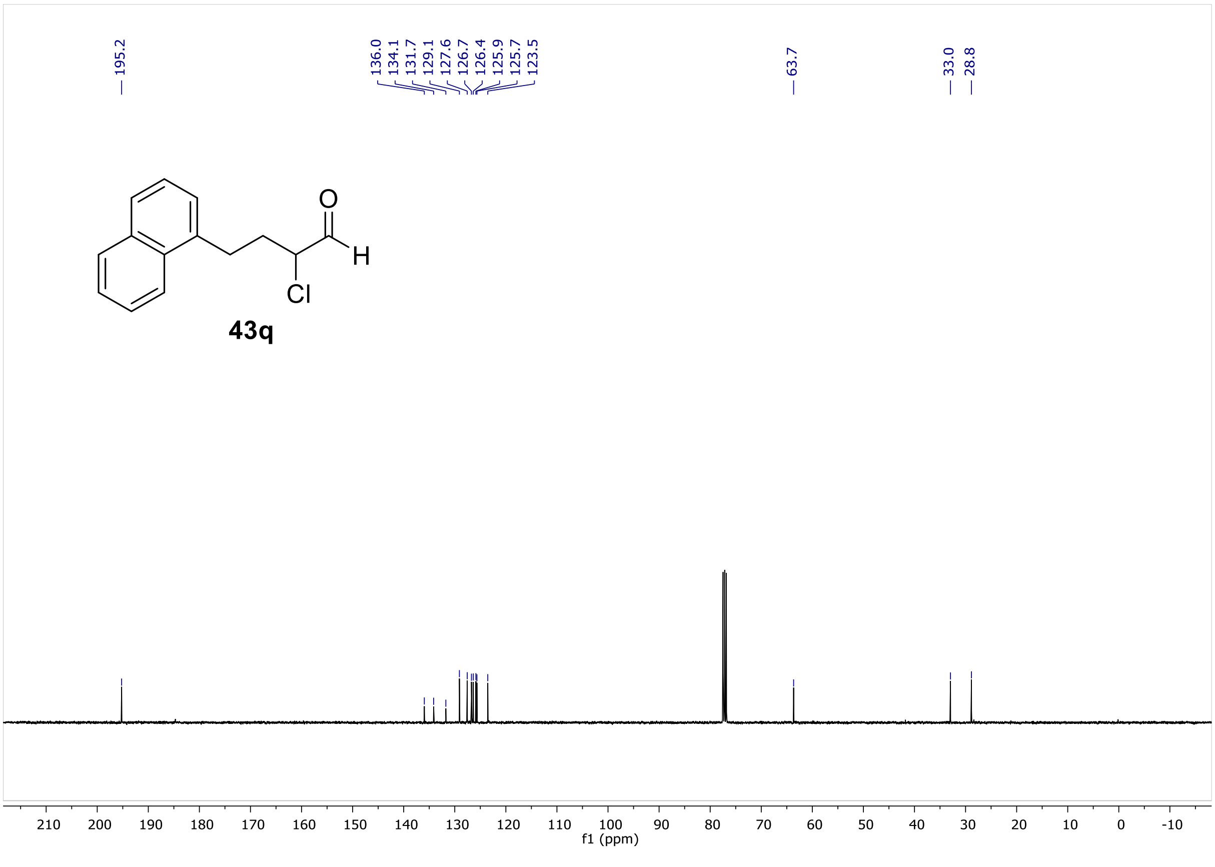

^{1}H NMR (400 MHz, $CDCl_3$): **43r**

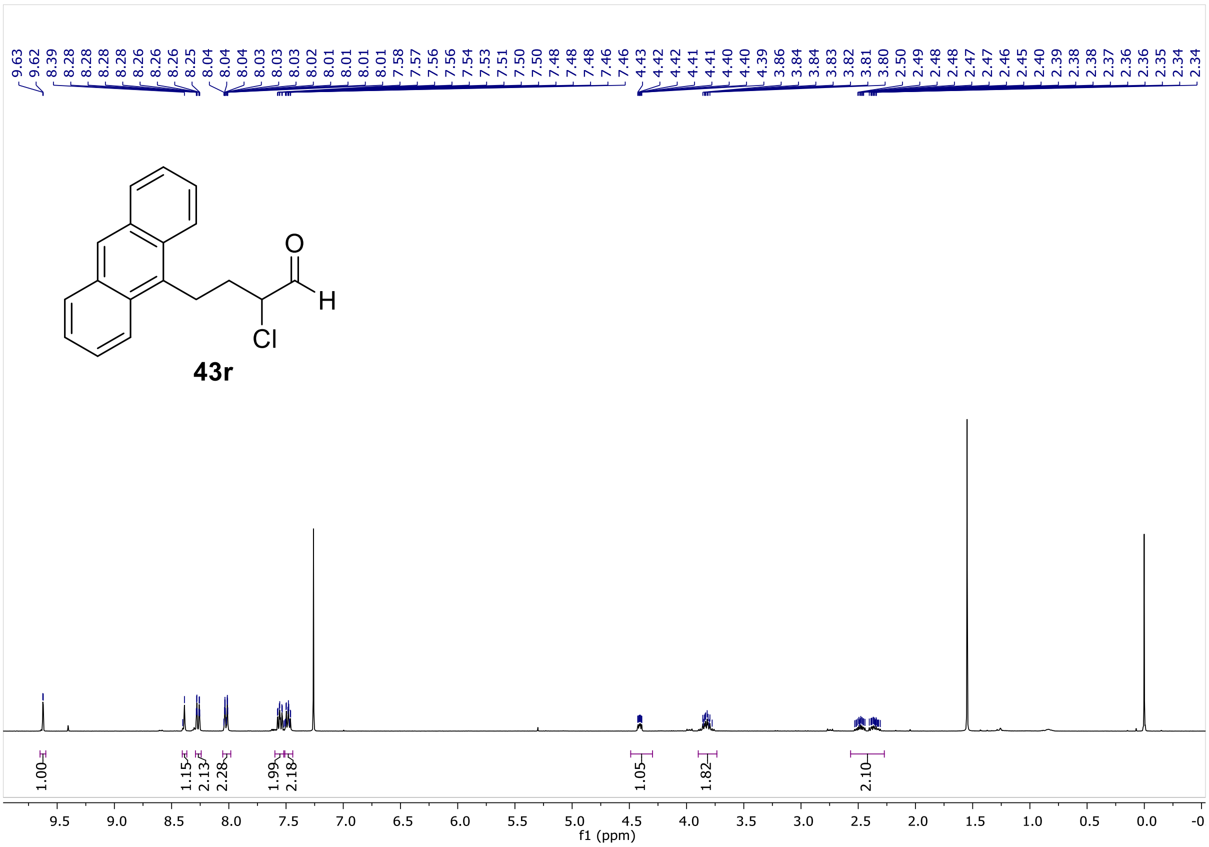

$^{13}C\{^{1}H\}$ NMR (150 MHz, $CDCl_3$): **43r**

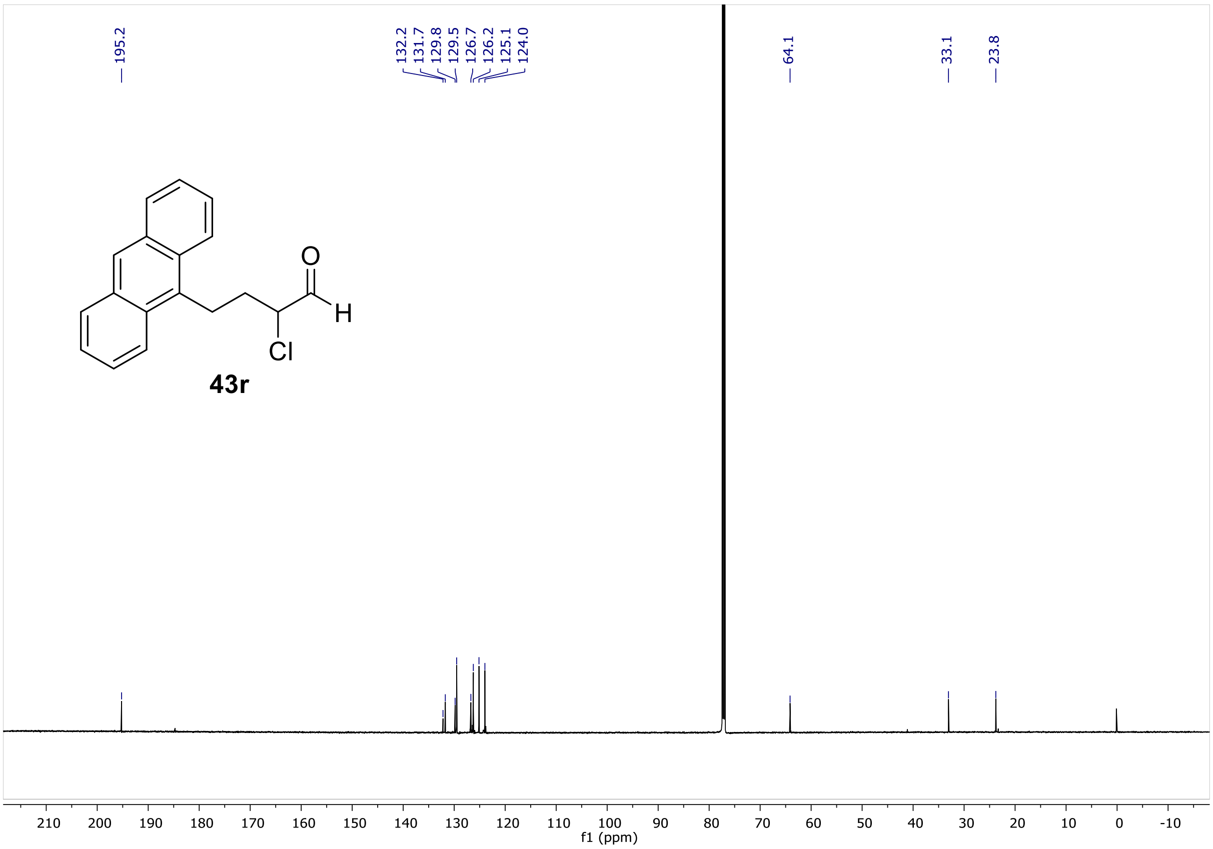

^{1}H NMR (400 MHz, $CDCl_3$): **25m**

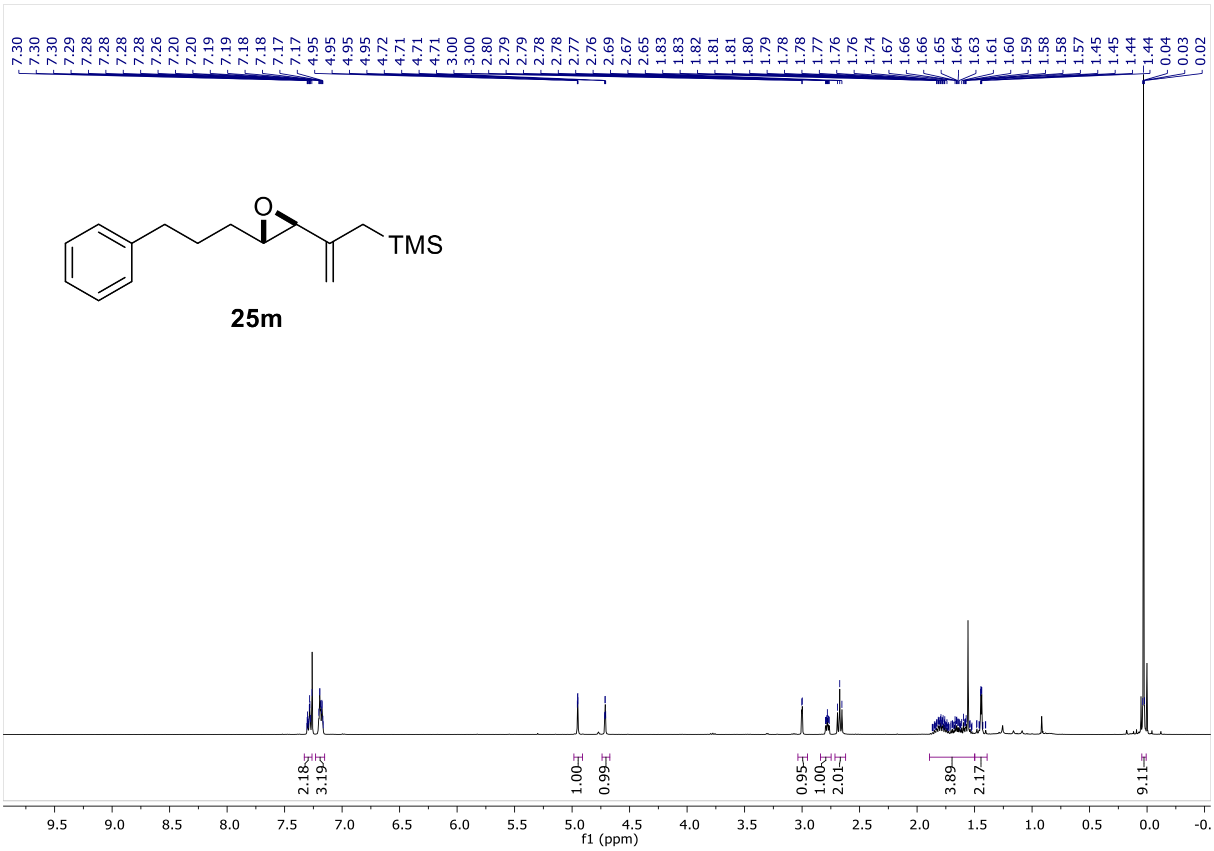

$^{13}C\{^{1}H\}$ NMR (100 MHz, $CDCl_3$): **25m**

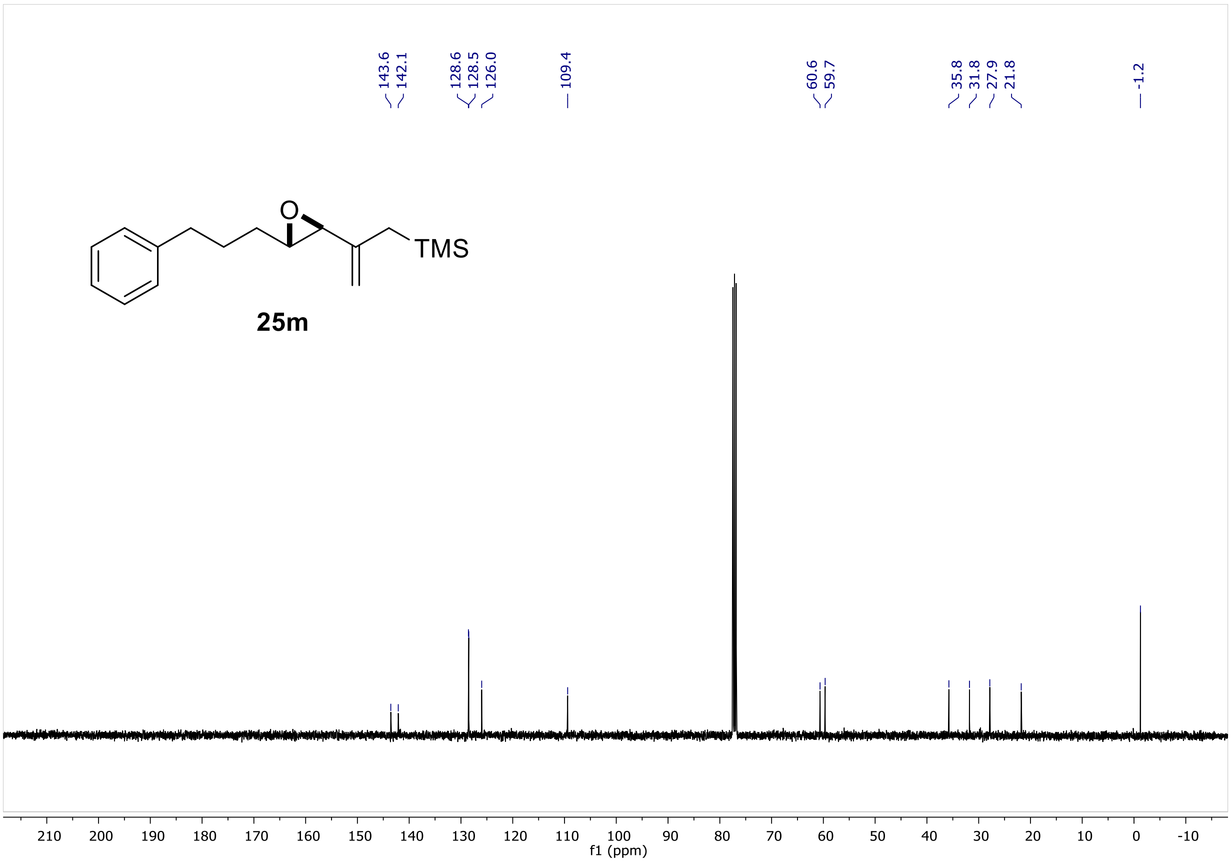

^{1}H NMR (500 MHz, $CDCl_3$): **25n**

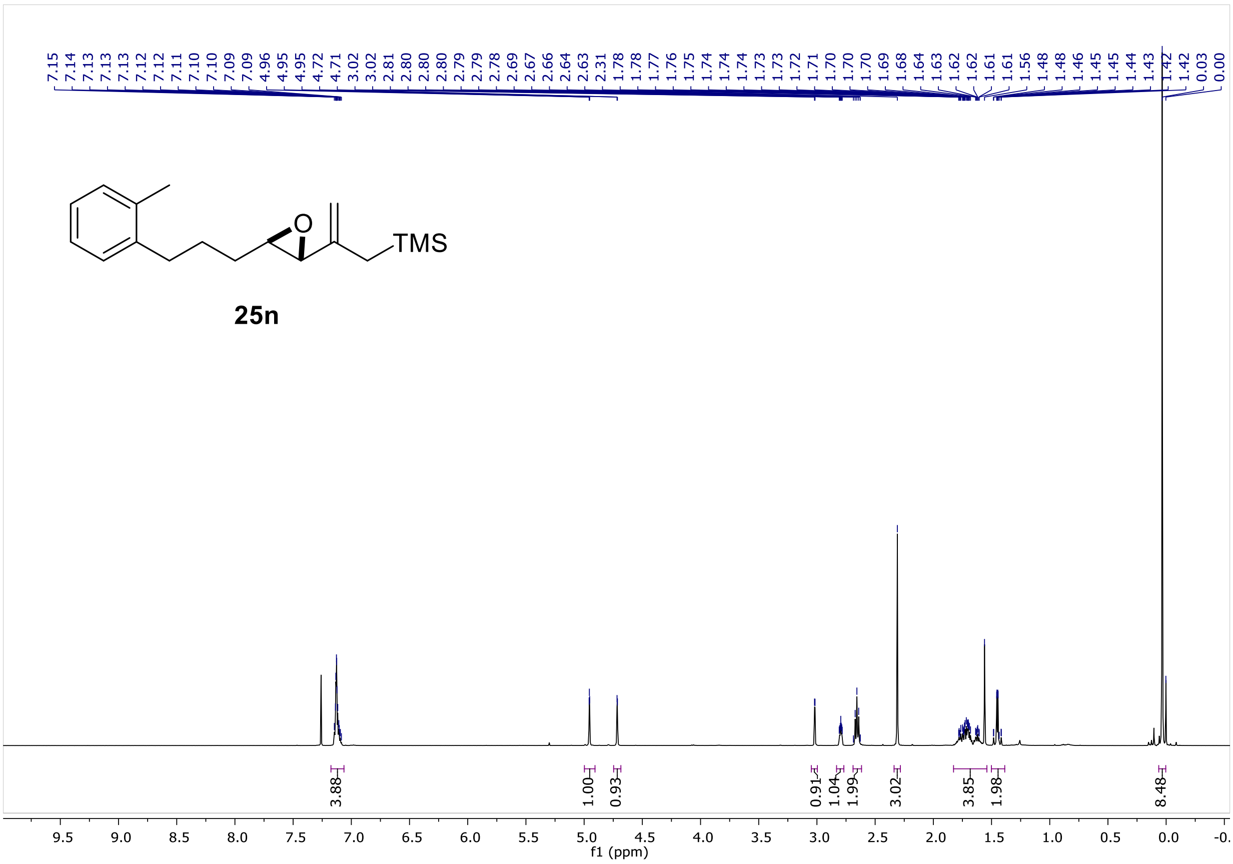

$^{13}C\{^{1}H\}$ NMR (150 MHz, $CDCl_3$): **25n**

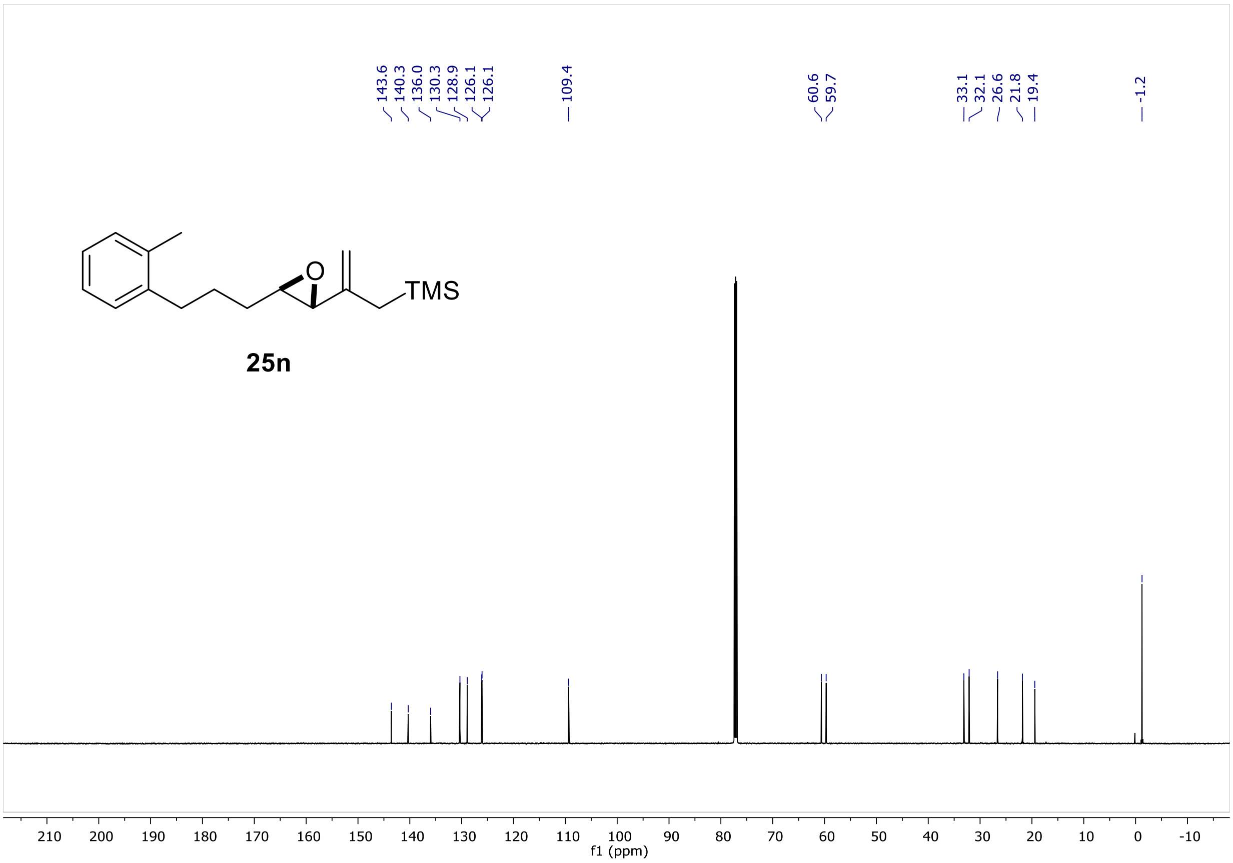

^{1}H NMR (400 MHz, $CDCl_3$): **25o**

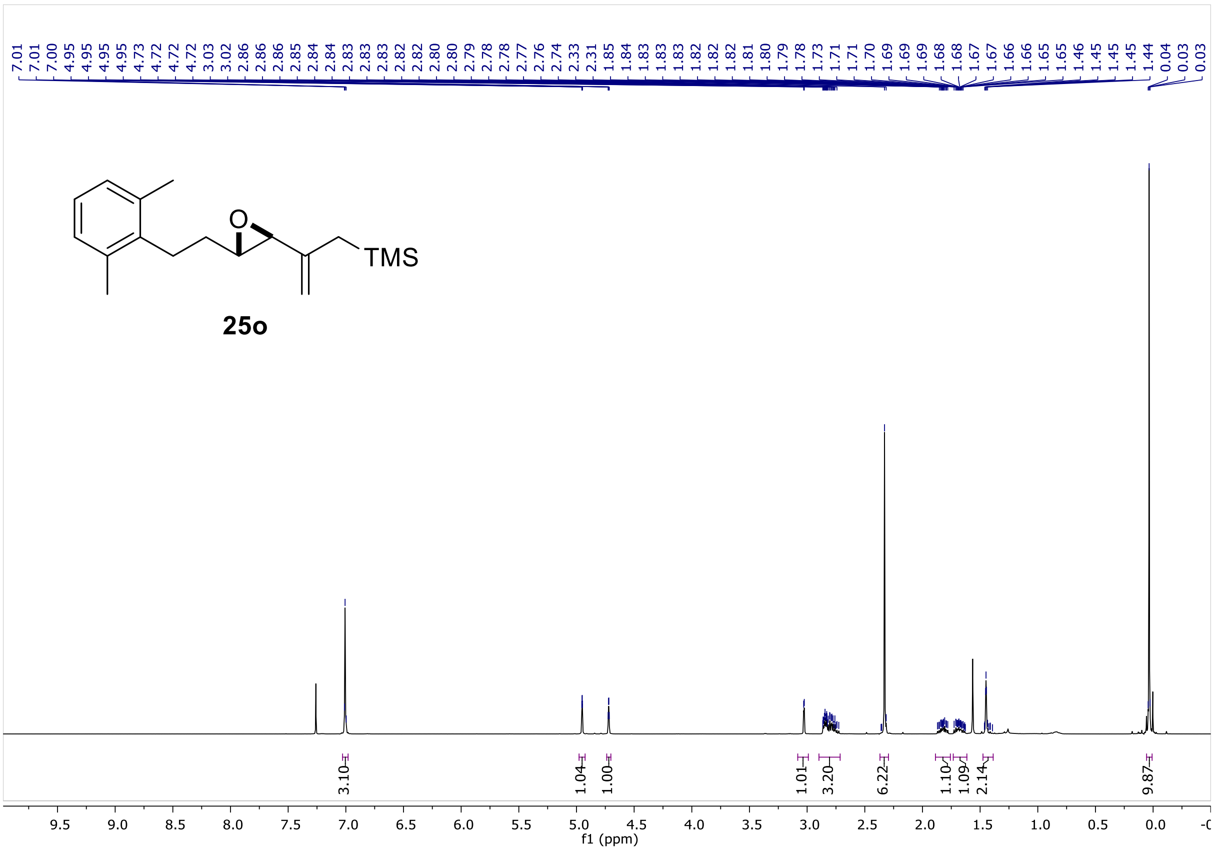

^{13}C{^{1}H} NMR (100 MHz, $CDCl_3$): **25o**

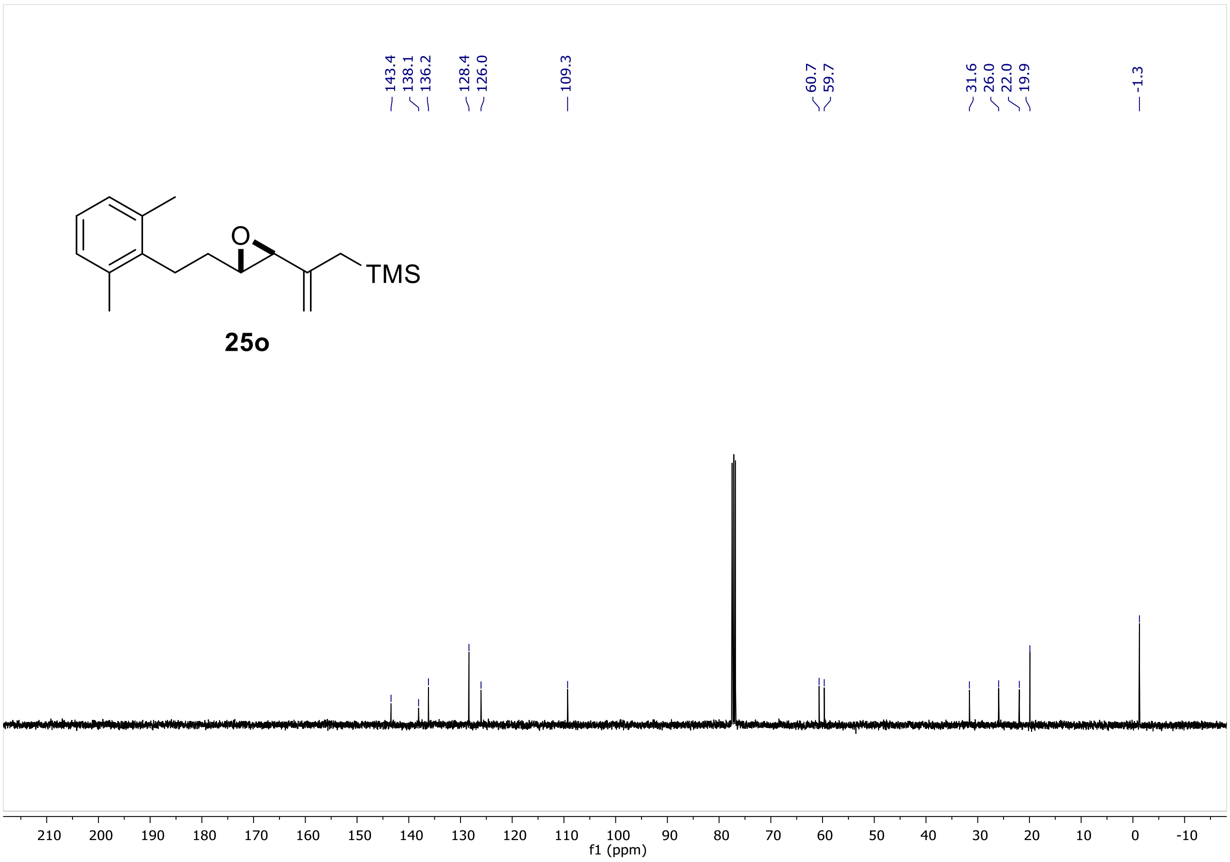

^{1}H NMR (400 MHz, $CDCl_3$): **25p**

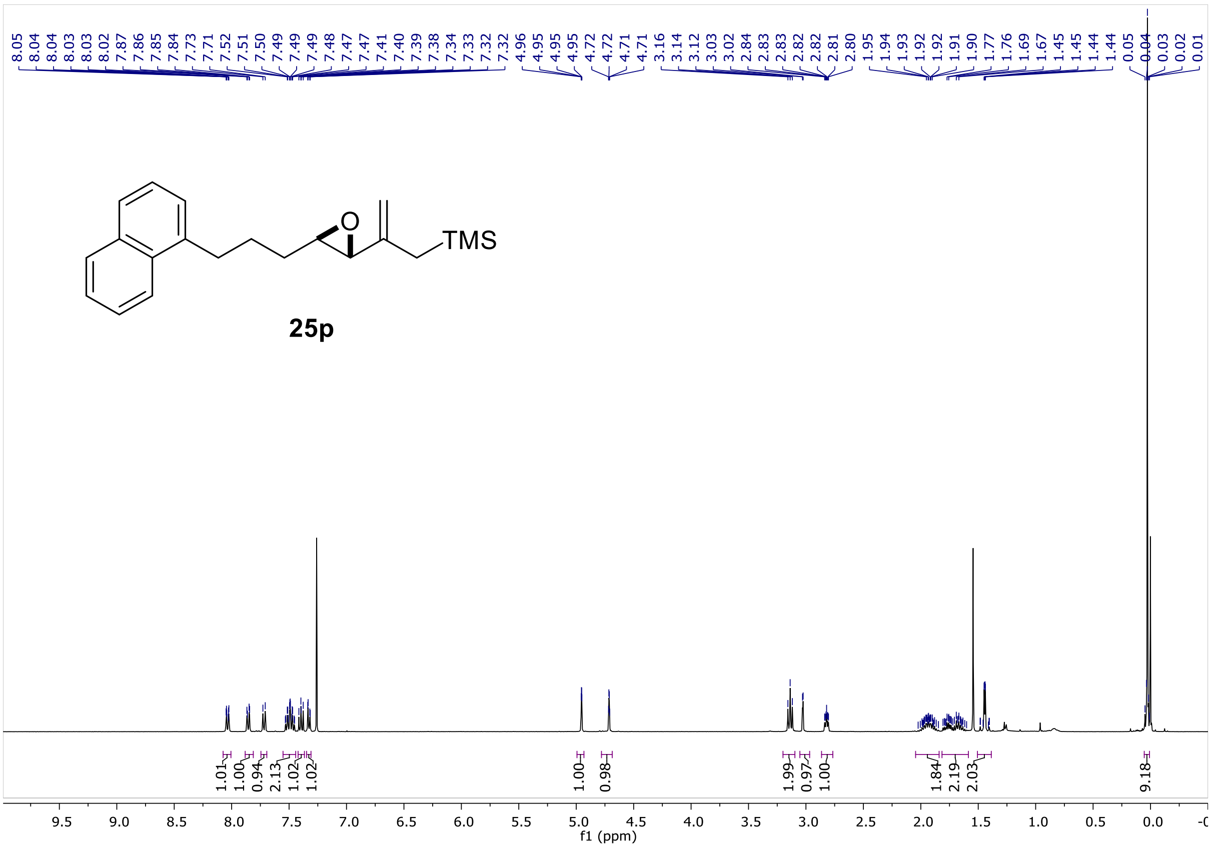

^{13}C{^{1}H} NMR (150 MHz, $CDCl_3$): **25p**

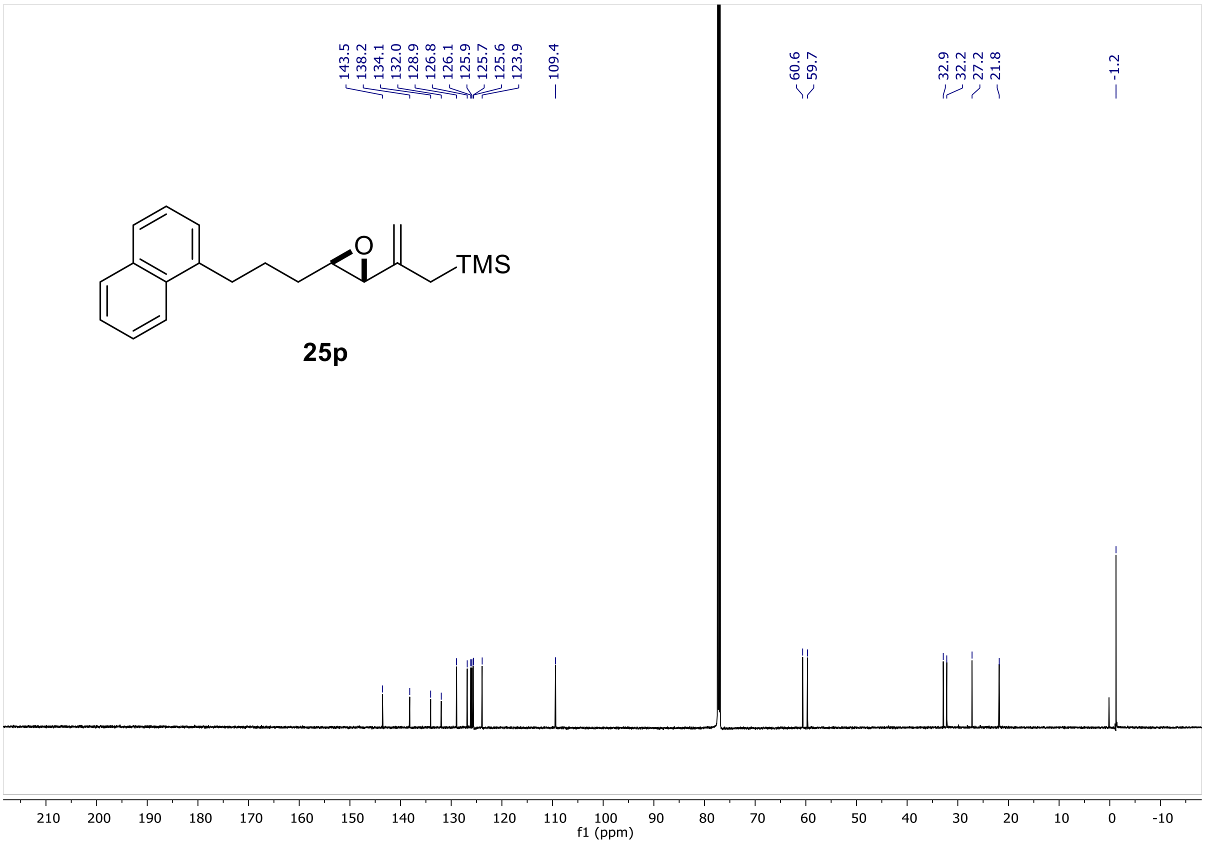

^{1}H NMR (400 MHz, $CDCl_3$): **25q**

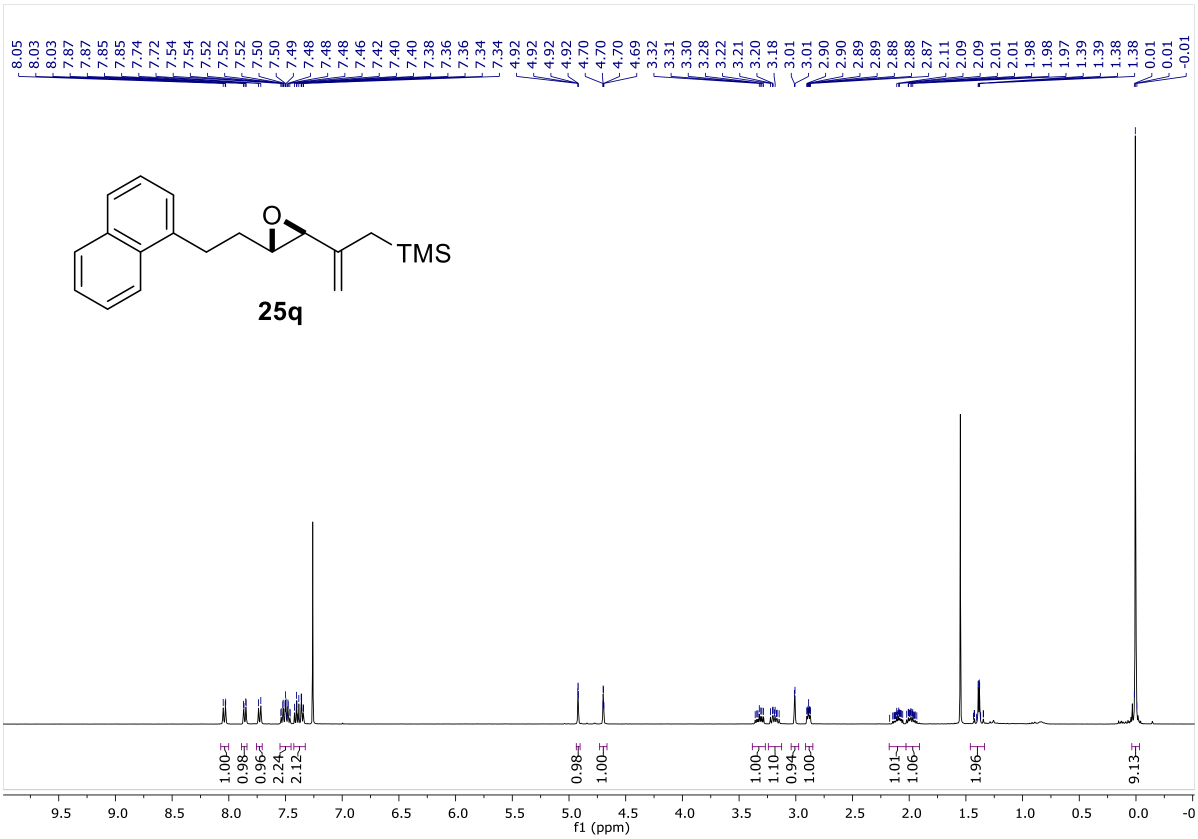

$^{13}C\{^{1}H\}$ NMR (150 MHz, $CDCl_3$): **25q**

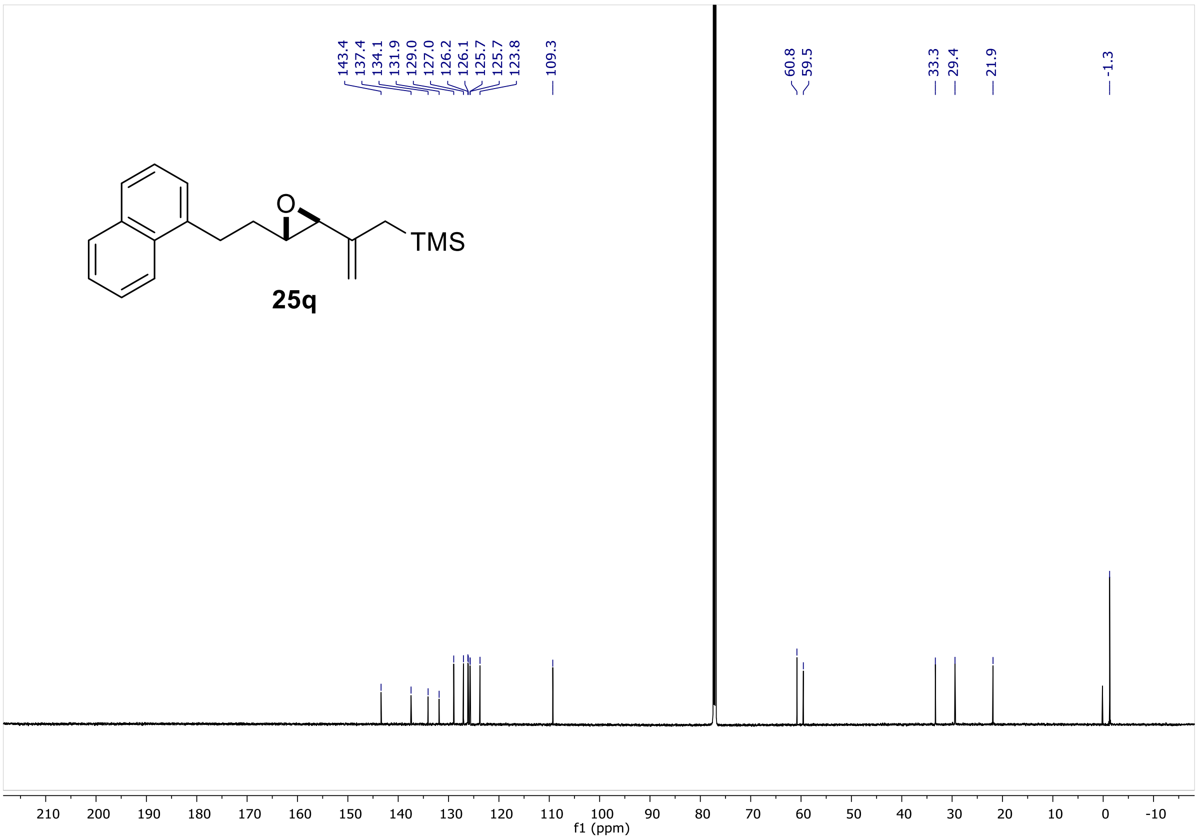

^{1}H NMR (400 MHz, $CDCl_3$): **25r**

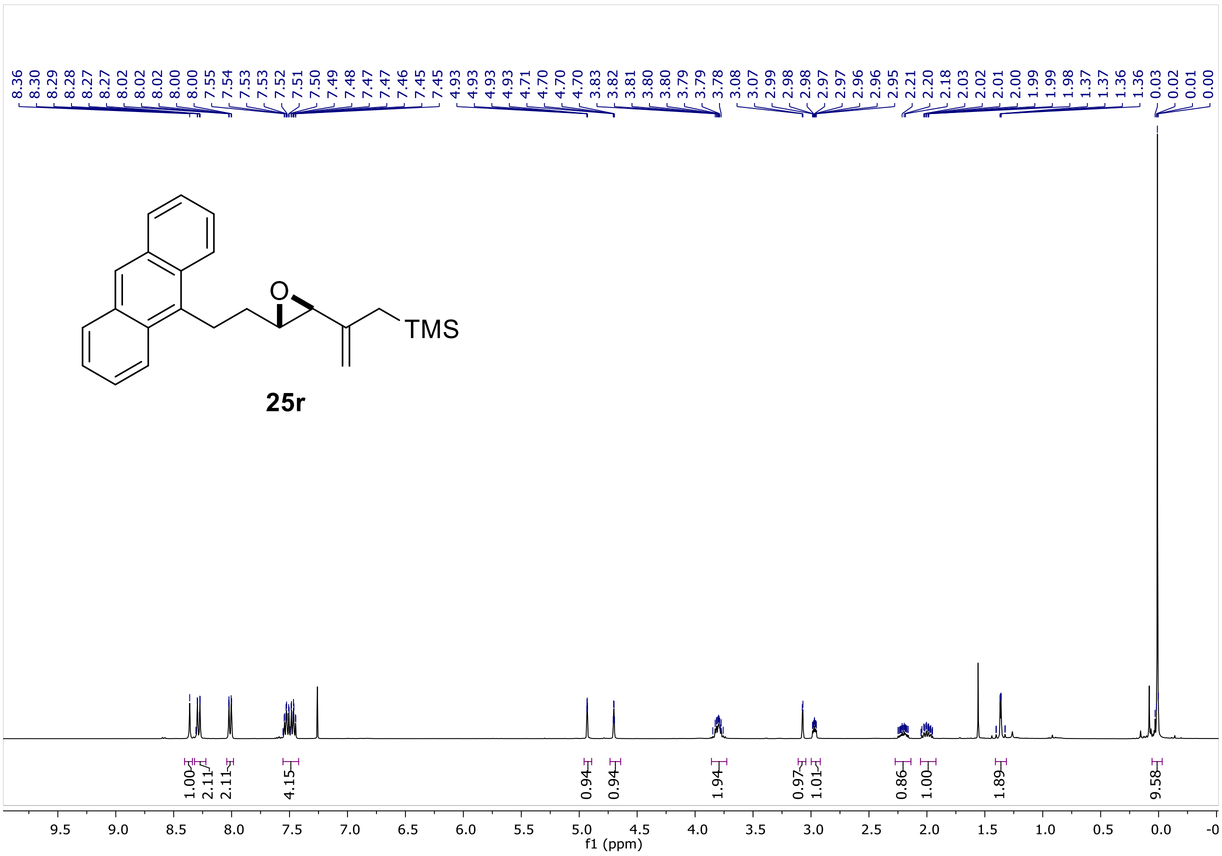

^{13}C{^{1}H} NMR (150 MHz, $CDCl_3$): **25r**

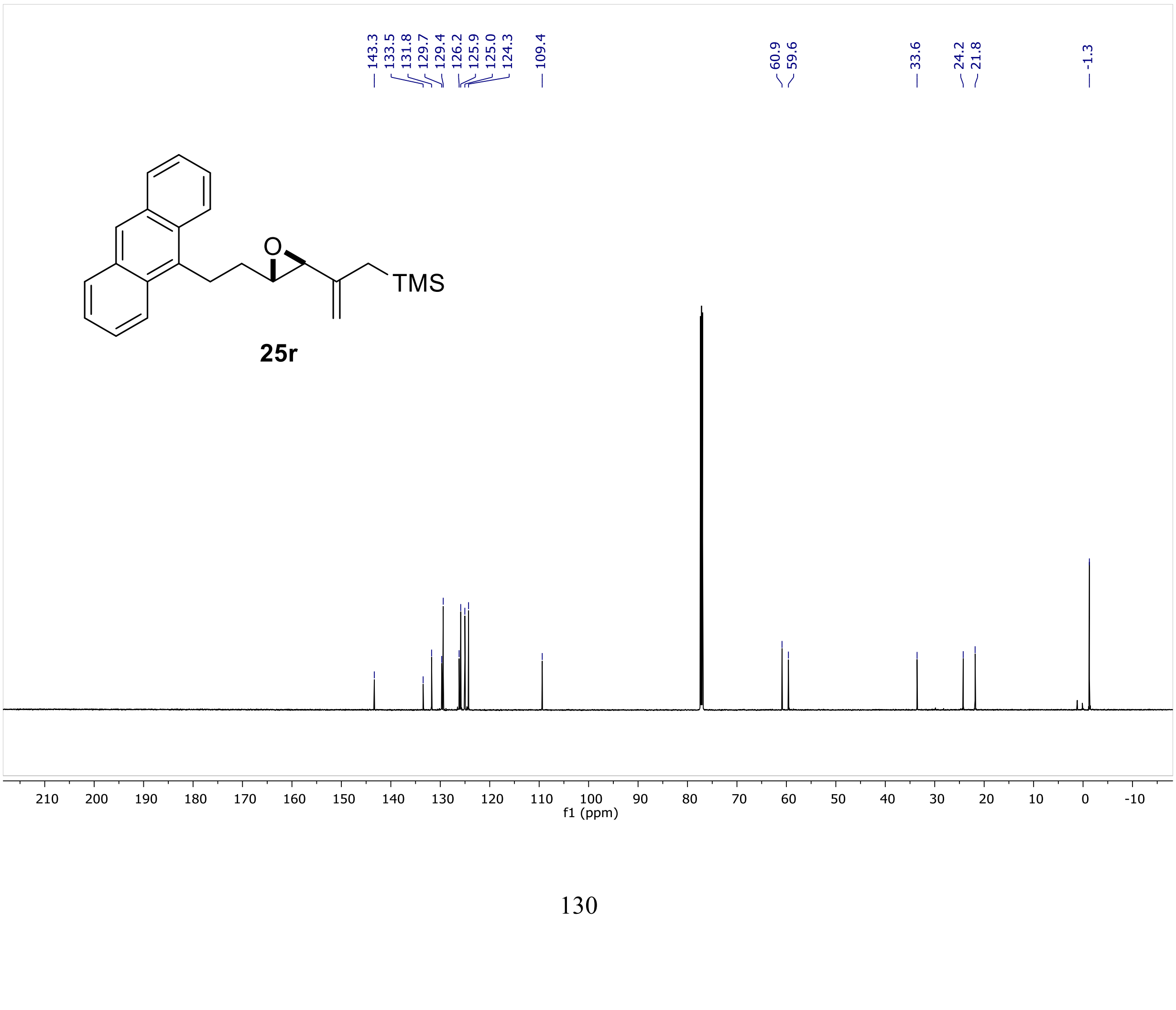

^{1}H NMR (600 MHz, $CDCl_3$): **26a**

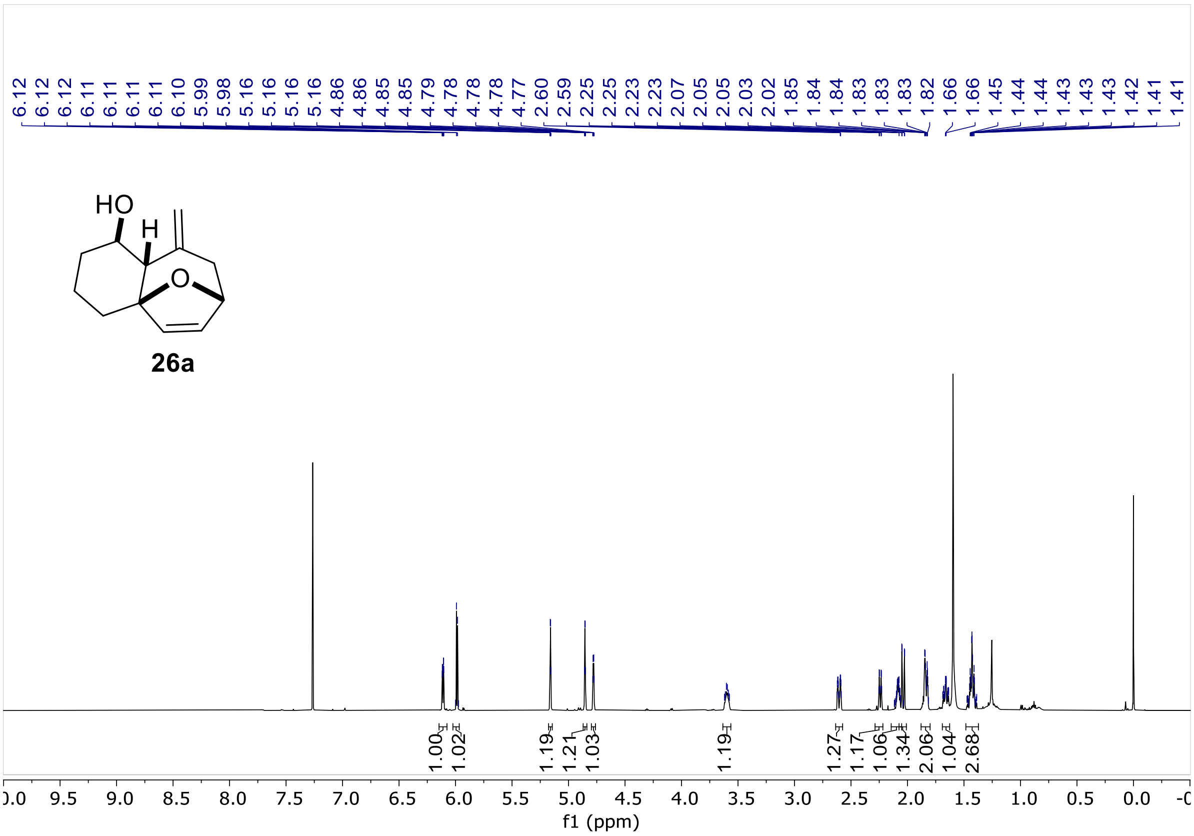

$^{13}C\{^{1}H\}$ NMR (150 MHz, $CDCl_3$): **26a**

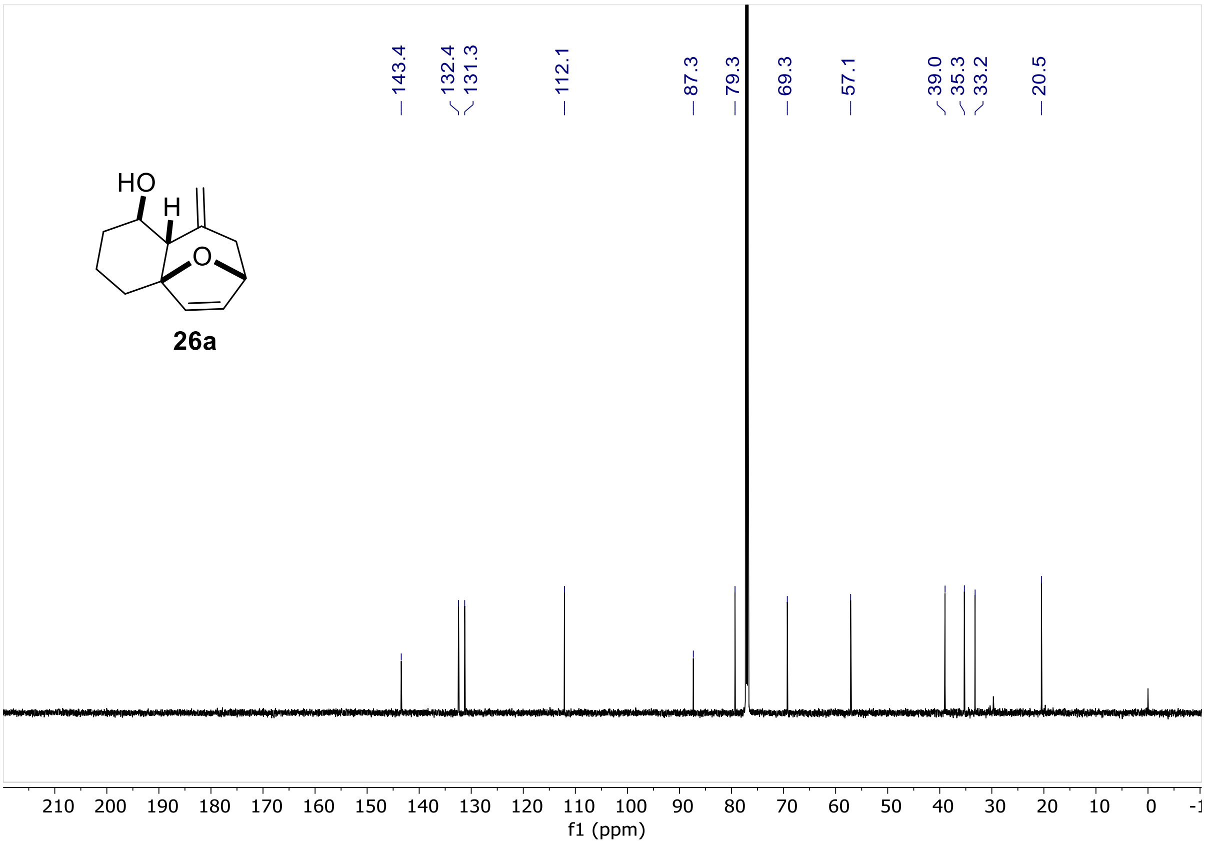

^{1}H NMR (600 MHz, $CDCl_3$): **27a**

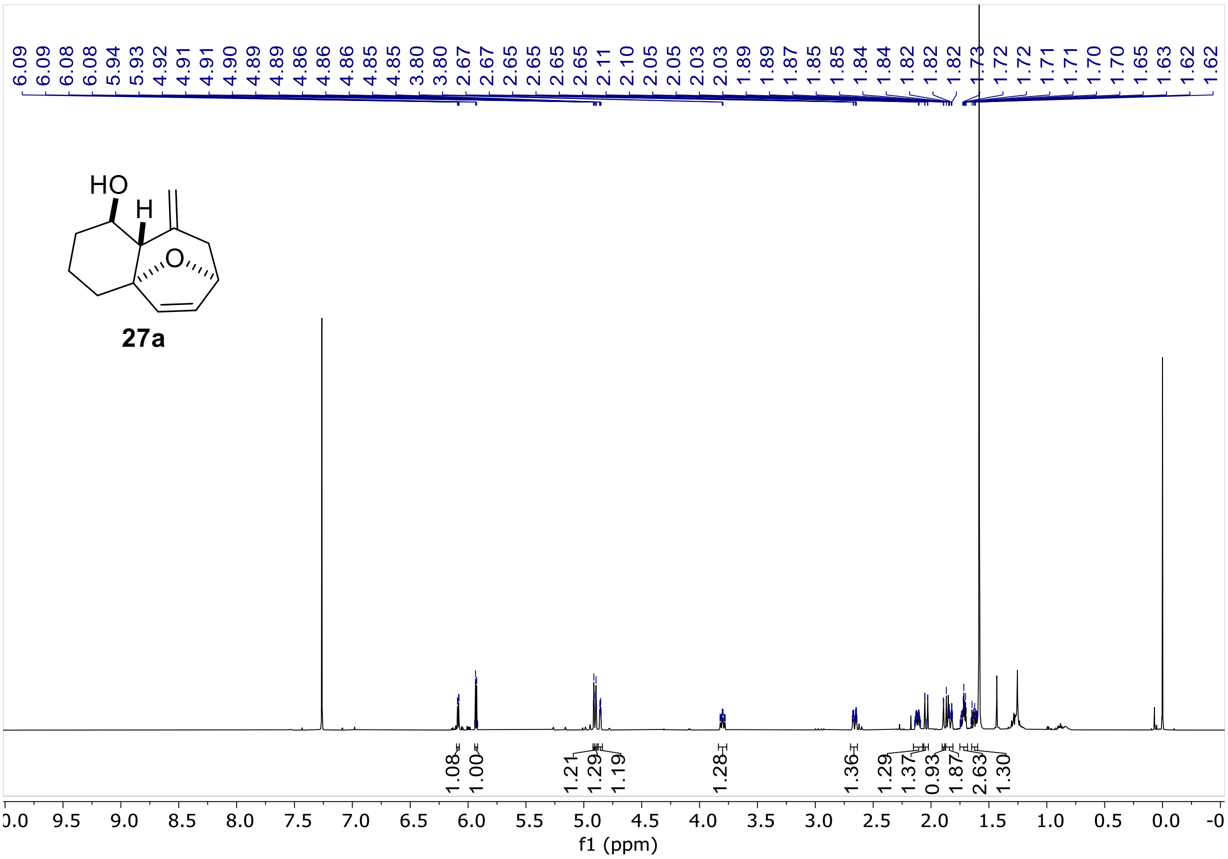

^{13}C{^{1}H} NMR (150 MHz, $CDCl_3$): **27a**

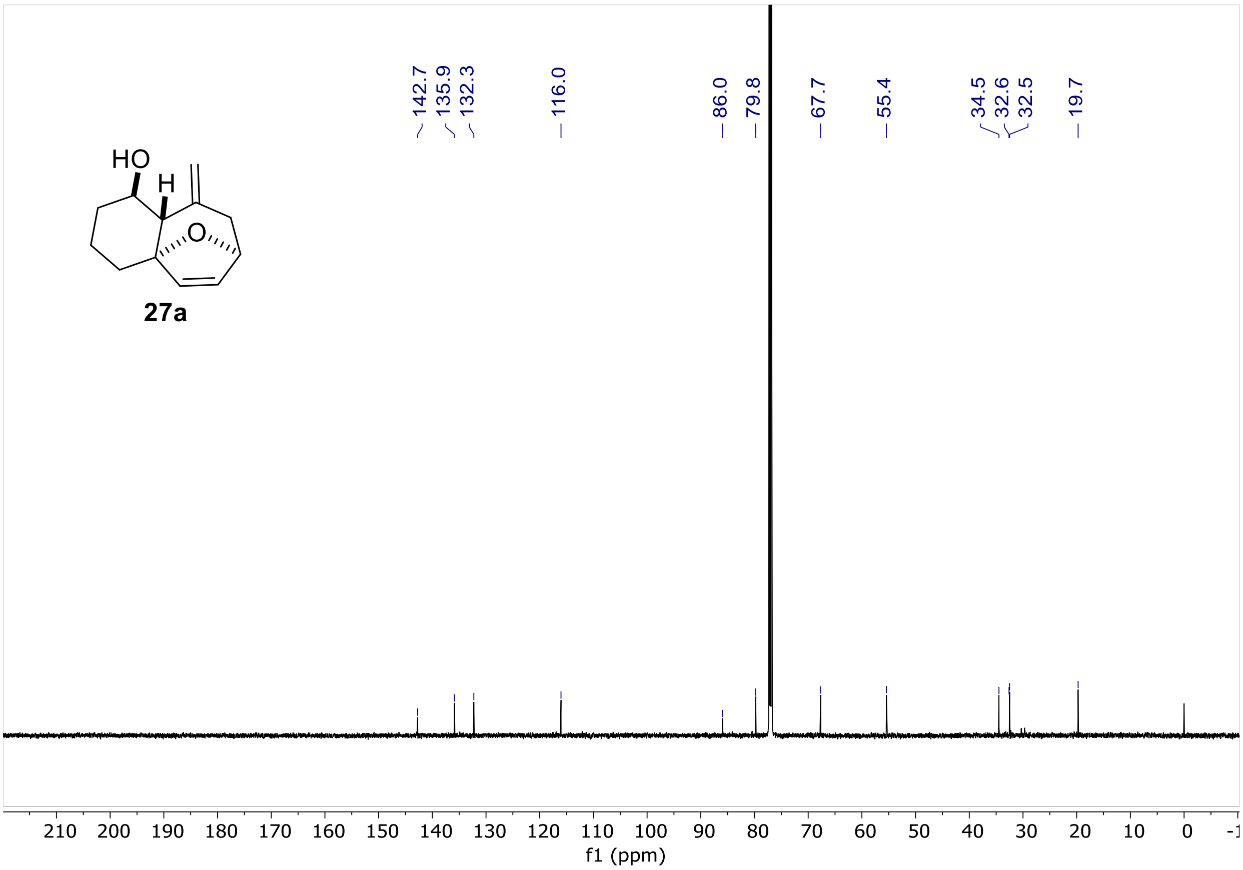

^{1}H NMR (600 MHz, $CDCl_3$): **26b**

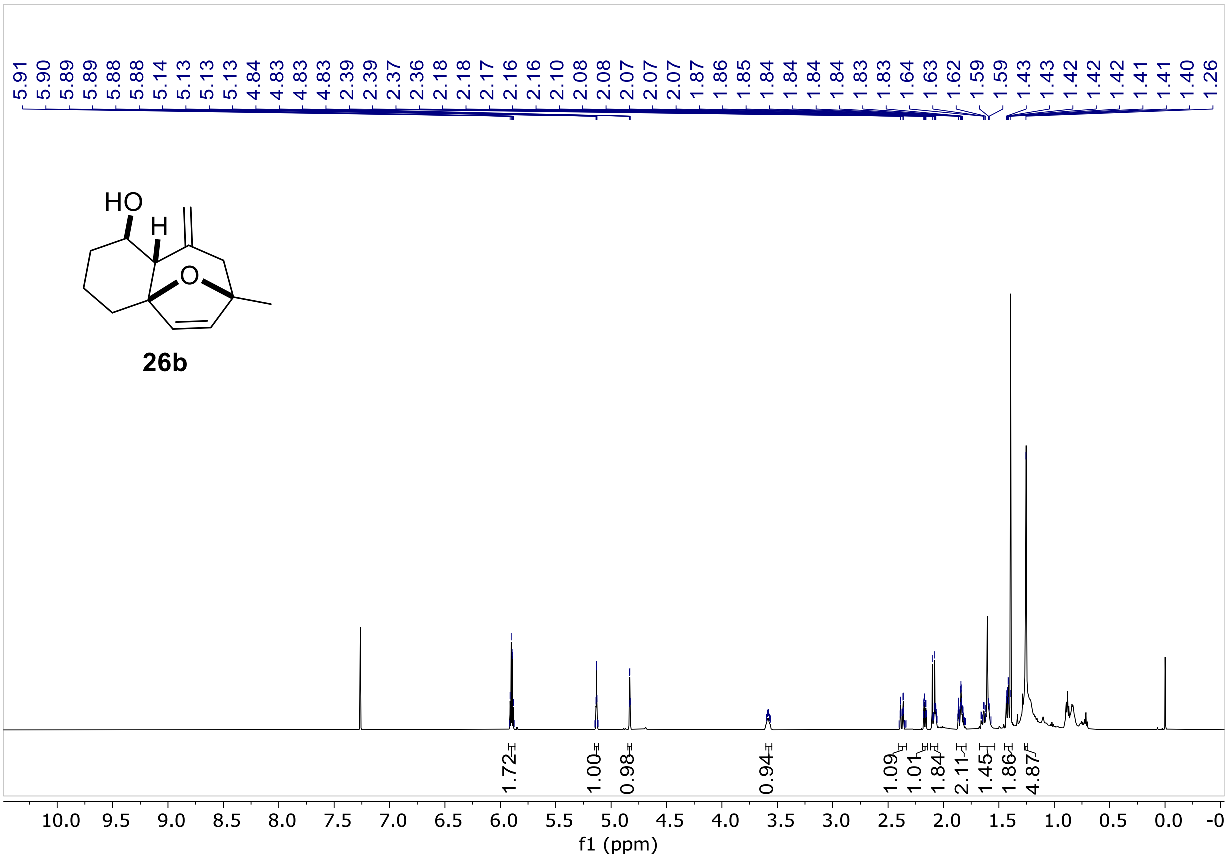

$^{13}C\{^{1}H\}$ NMR (150 MHz, $CDCl_3$): **26b**

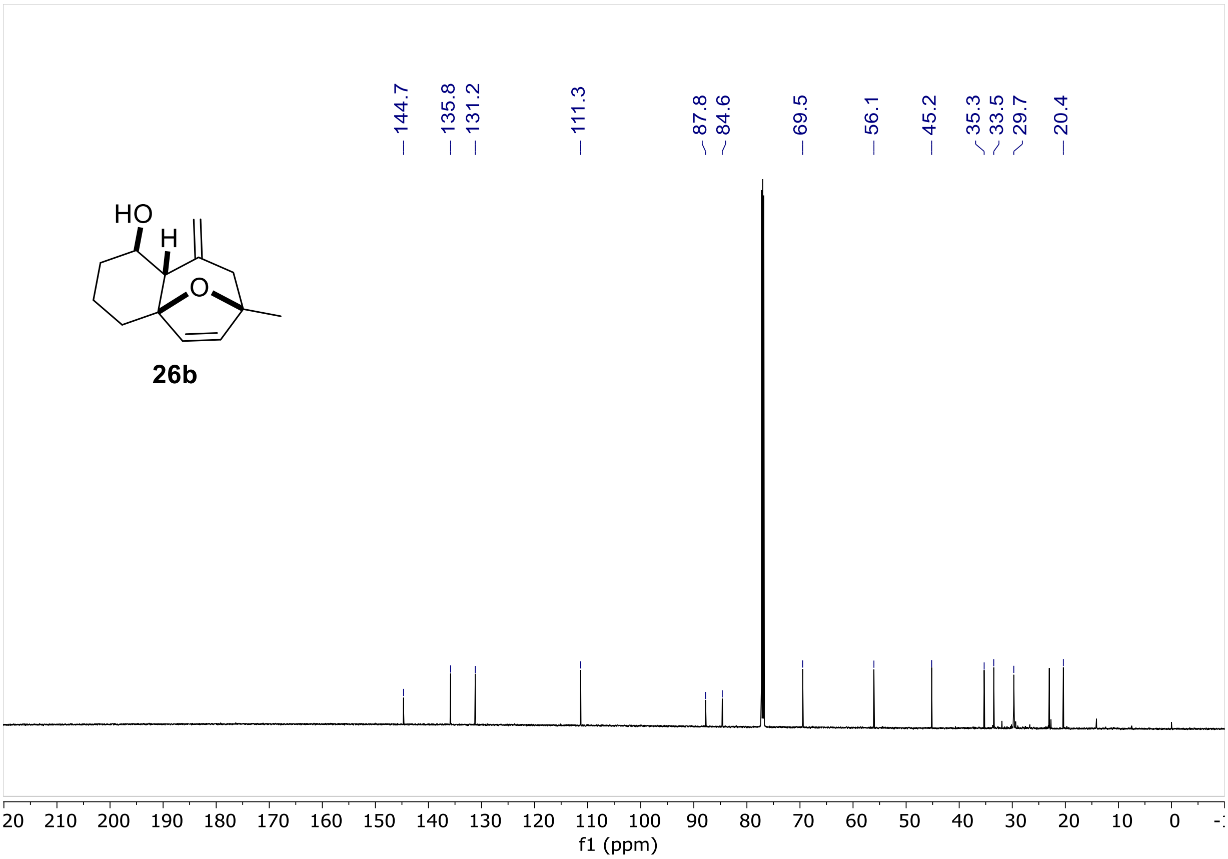

^{1}H NMR (600 MHz, $CDCl_3$): **27b**

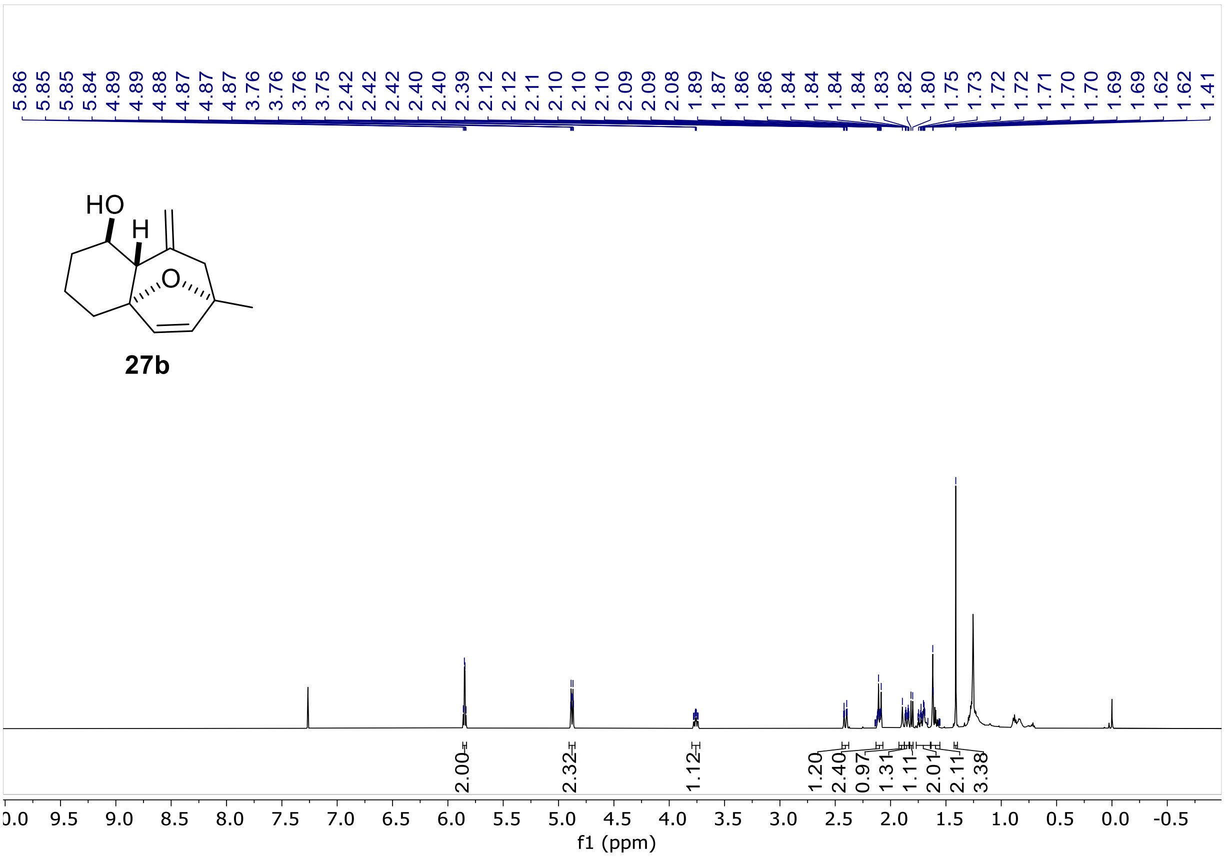

$^{13}C\{^{1}H\}$ NMR (150 MHz, $CDCl_3$): **27b**

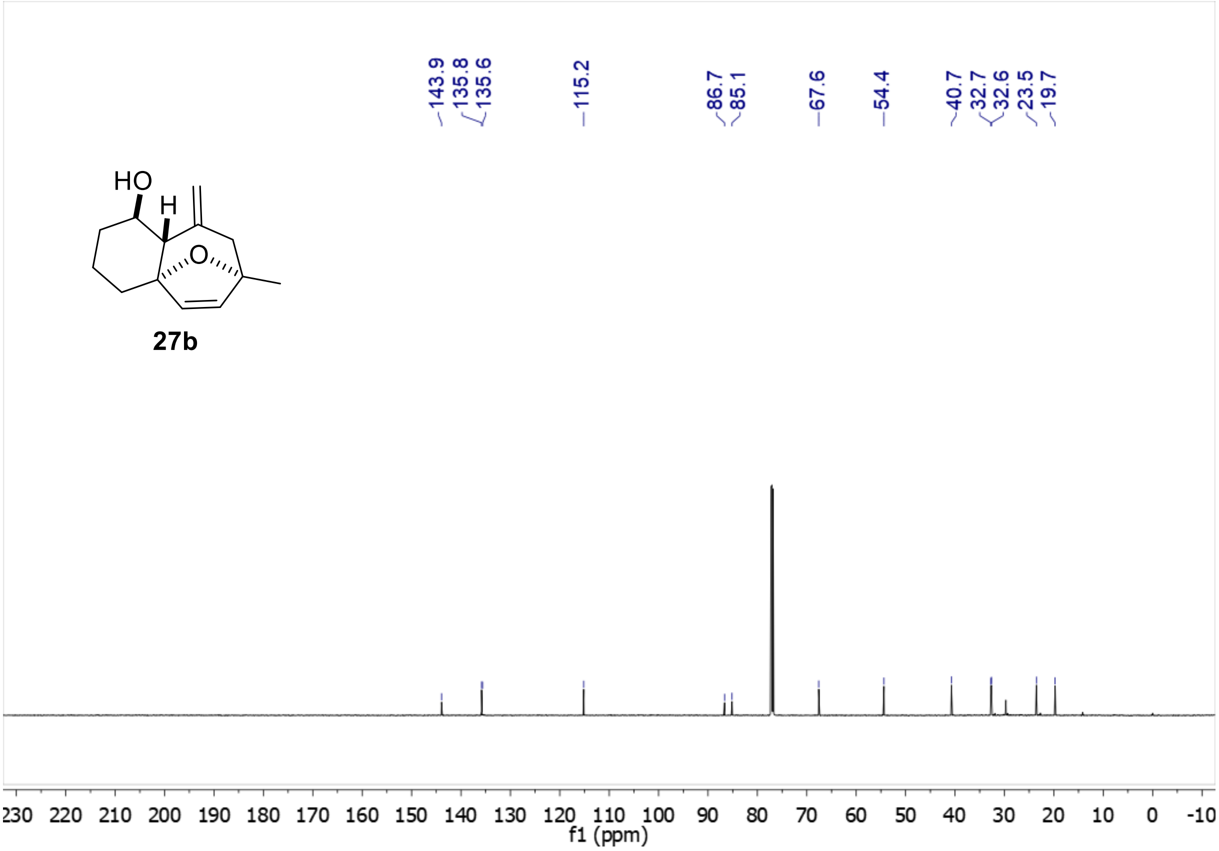

^{1}H NMR (600 MHz, $CDCl_3$): **26c**

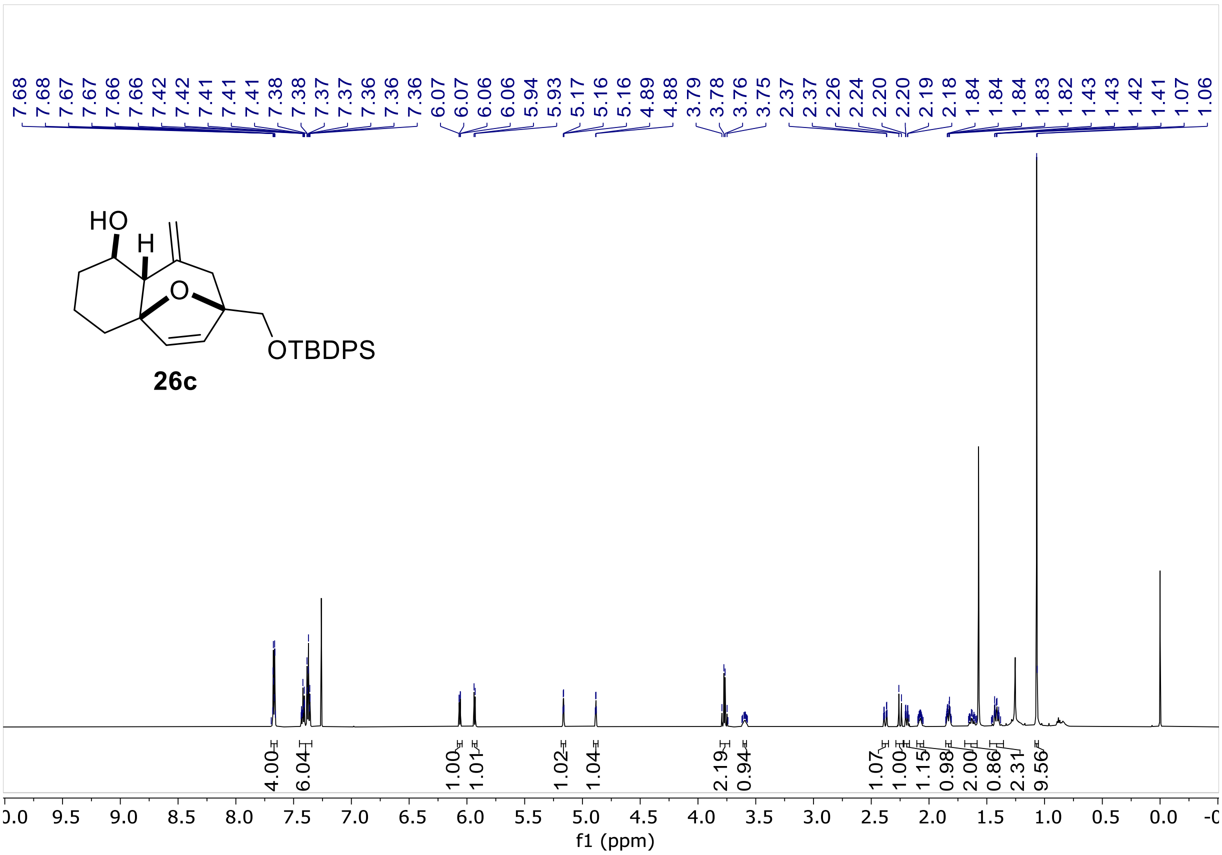

^{13}C{^{1}H} NMR (150 MHz, $CDCl_3$): **26c**

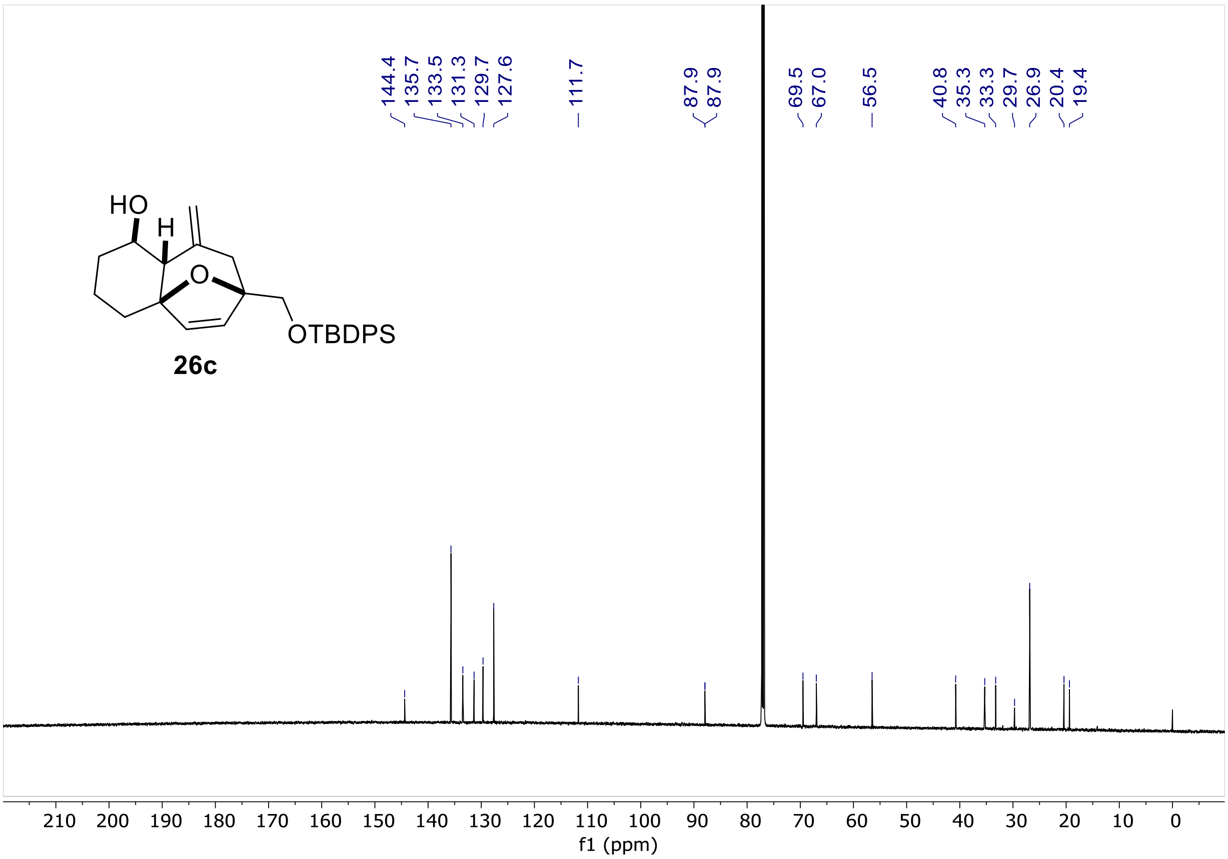

^{1}H NMR (600 MHz, $CDCl_3$): **27c**

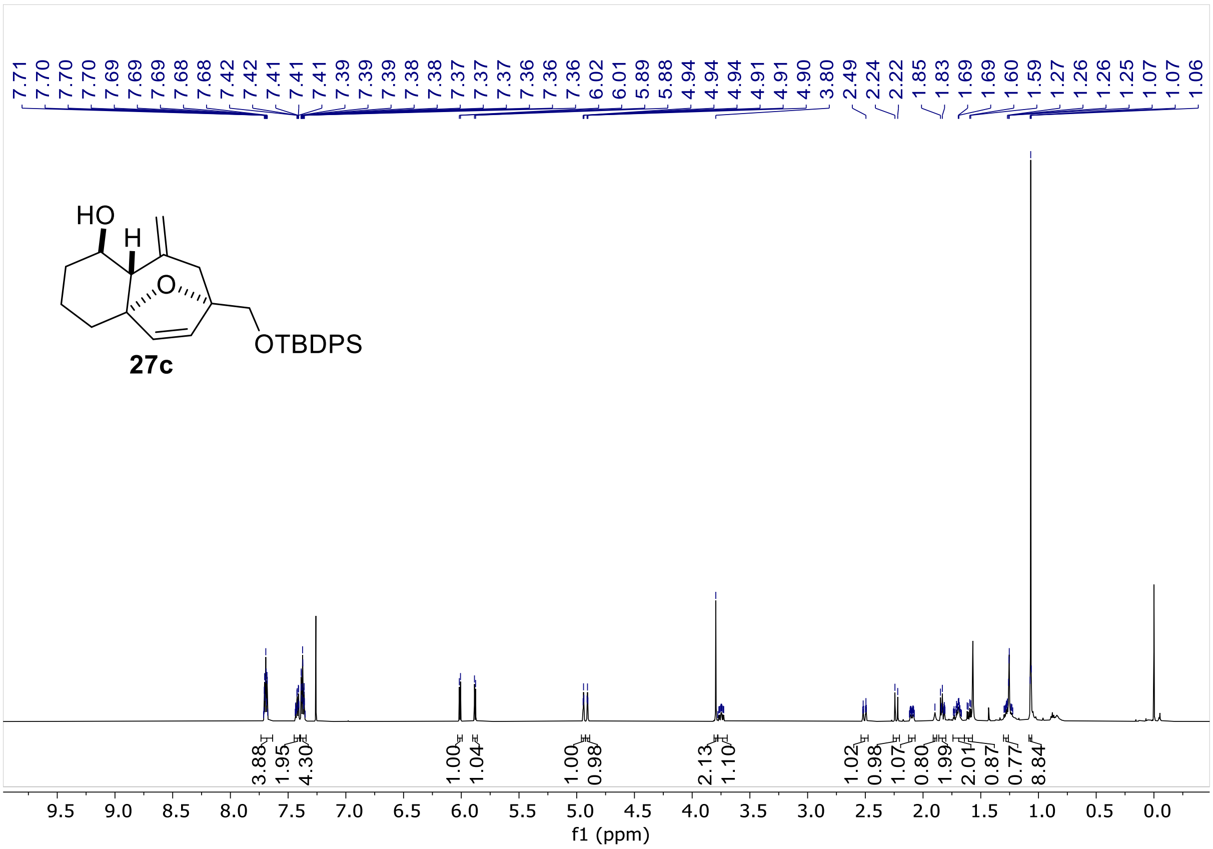

$^{13}C\{^{1}H\}$ NMR (150 MHz, $CDCl_3$): **27c**

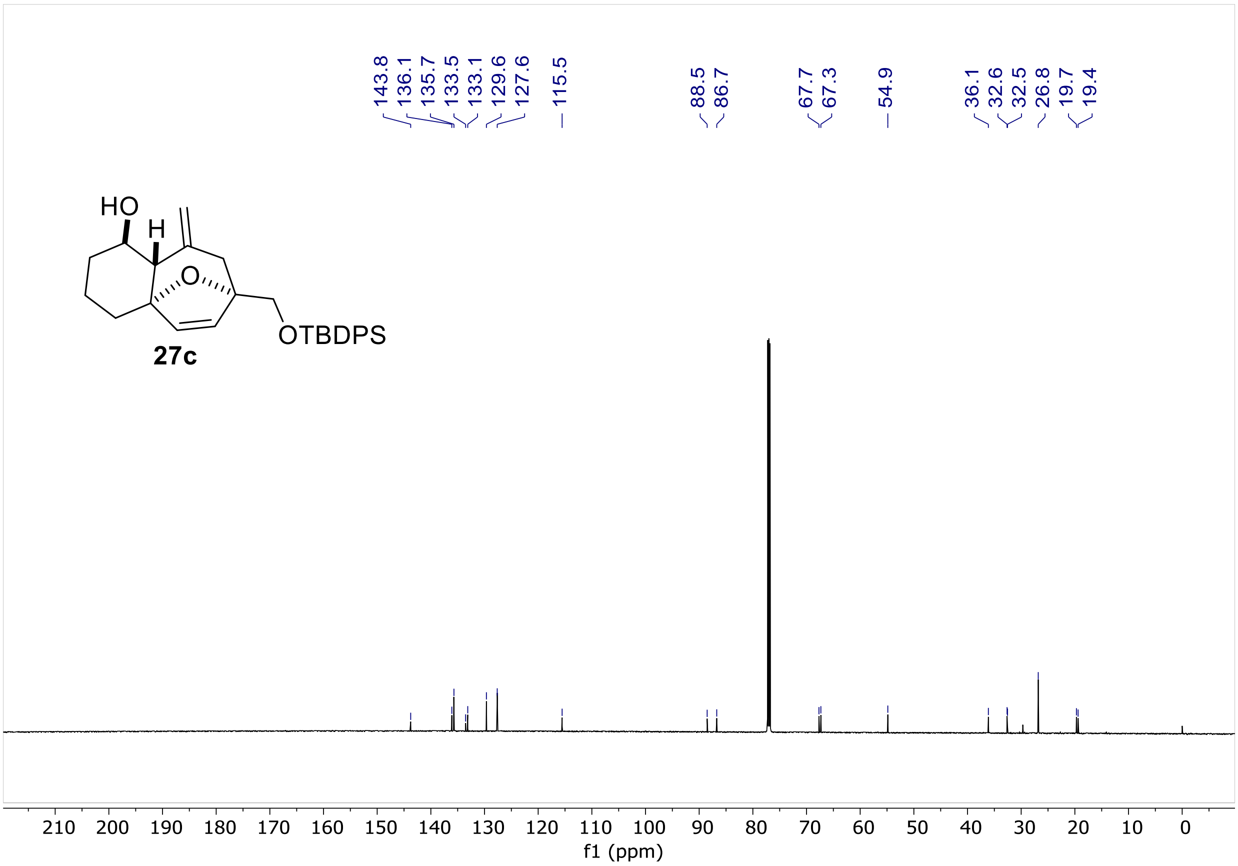

^{1}H NMR (600 MHz, $CDCl_3$): **27d**

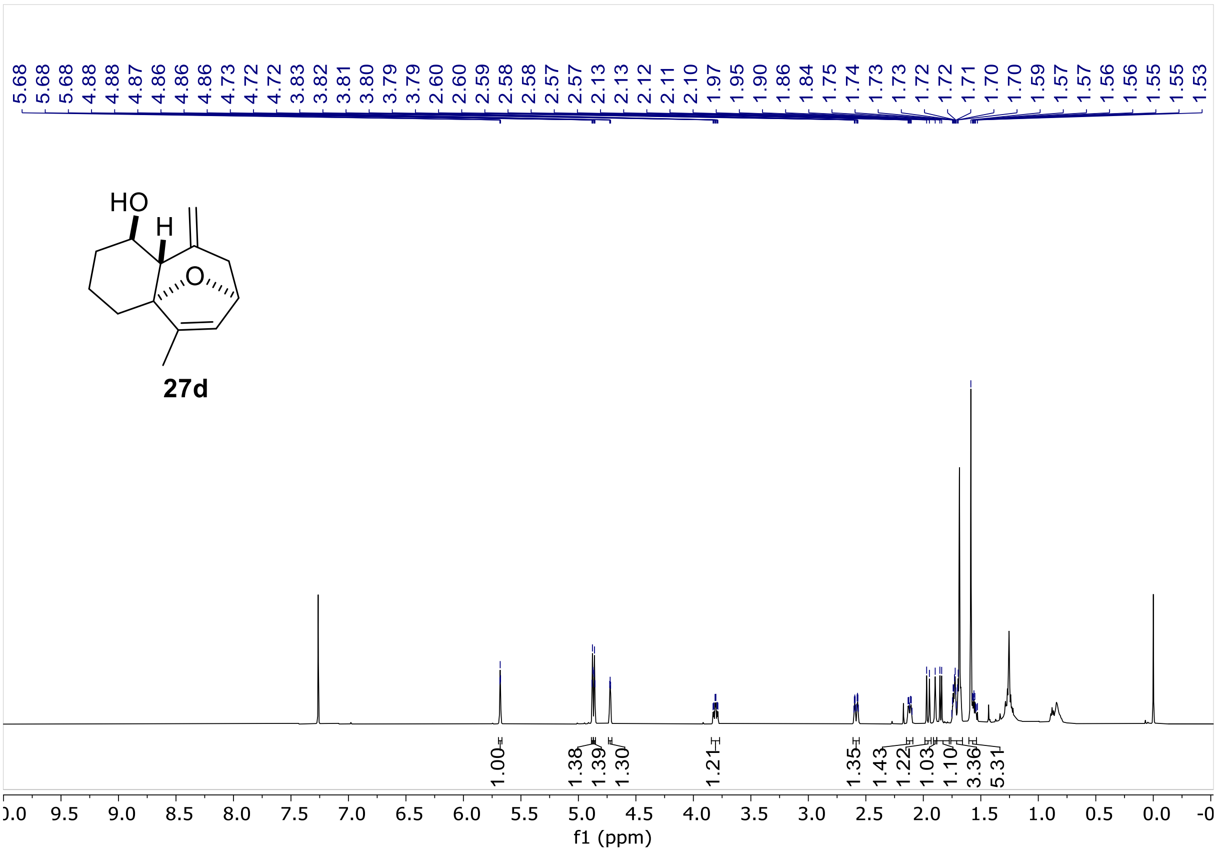

$^{13}C\{^{1}H\}$ NMR (150 MHz, $CDCl_3$): **27d**

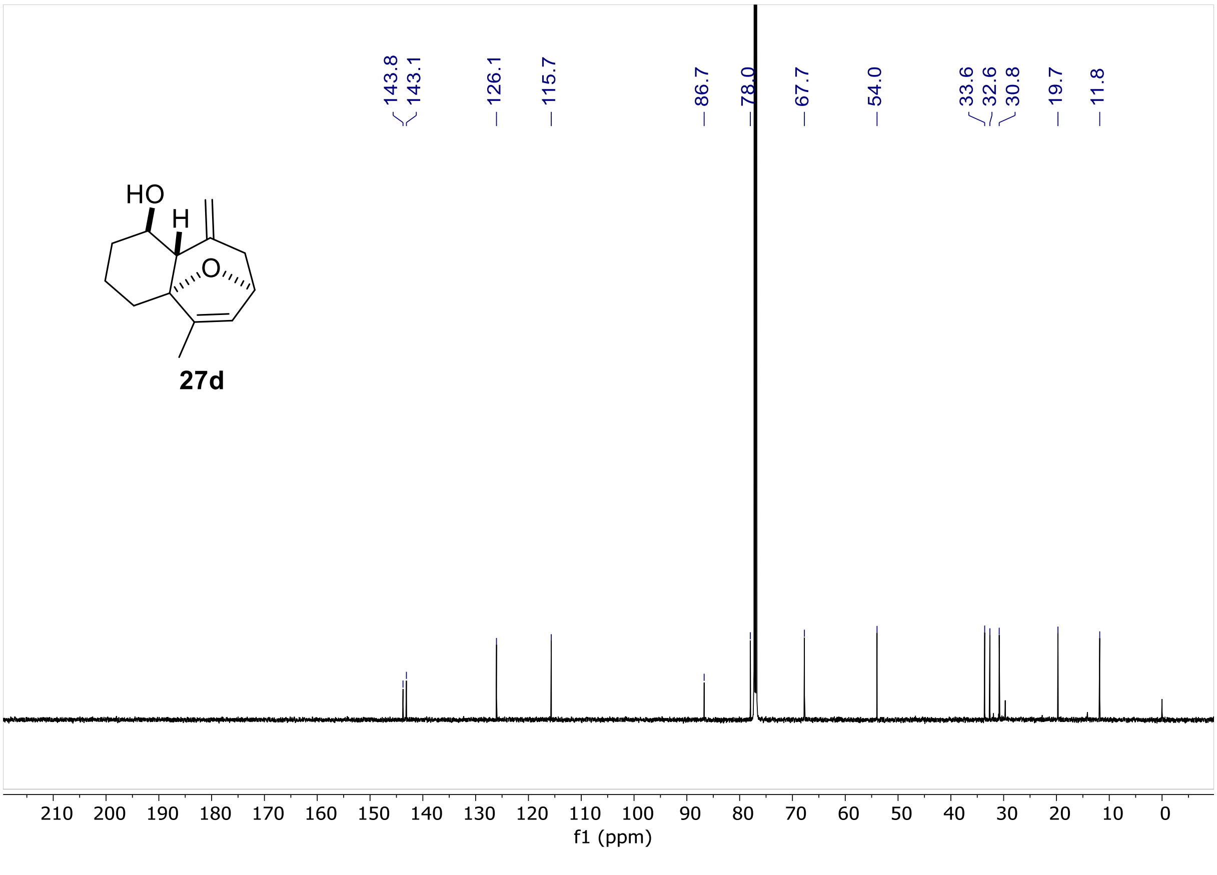

^{1}H NMR (600 MHz, $CDCl_3$): **26e**

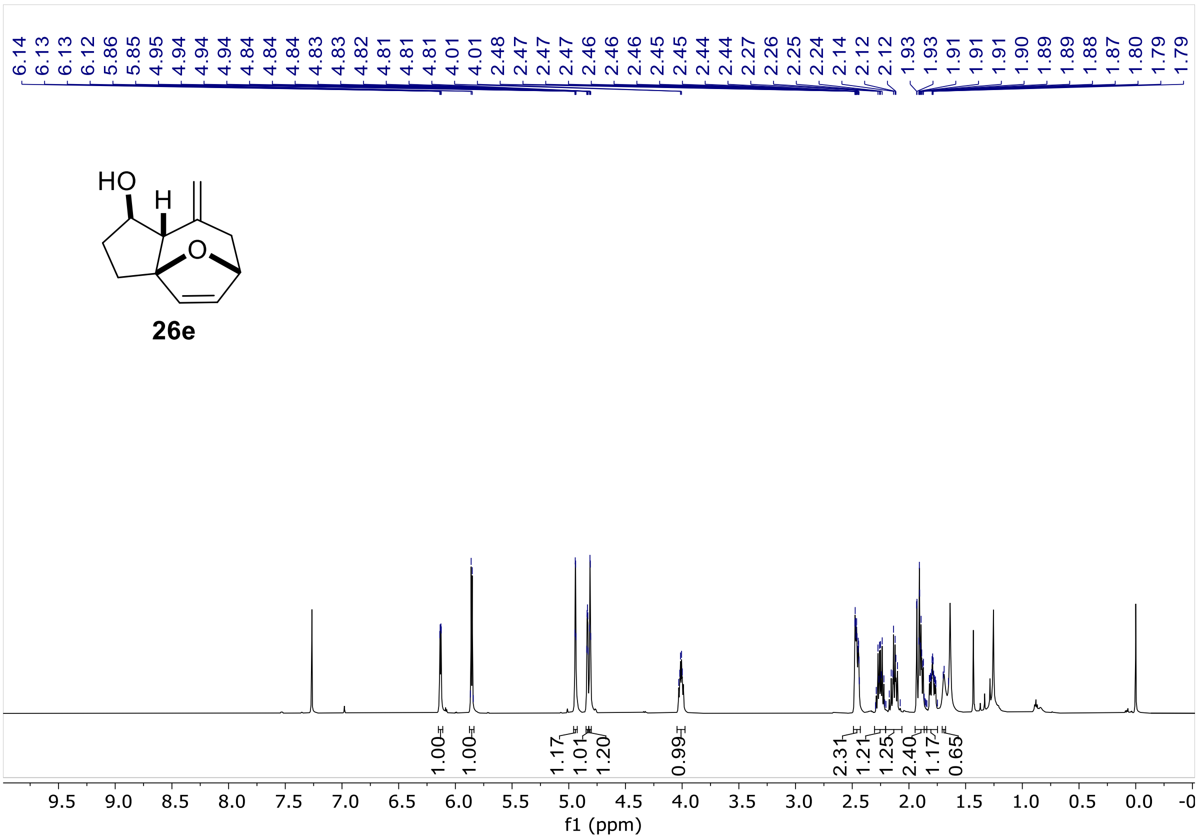

^{13}C{^{1}H} NMR (150 MHz, $CDCl_3$): **26e**

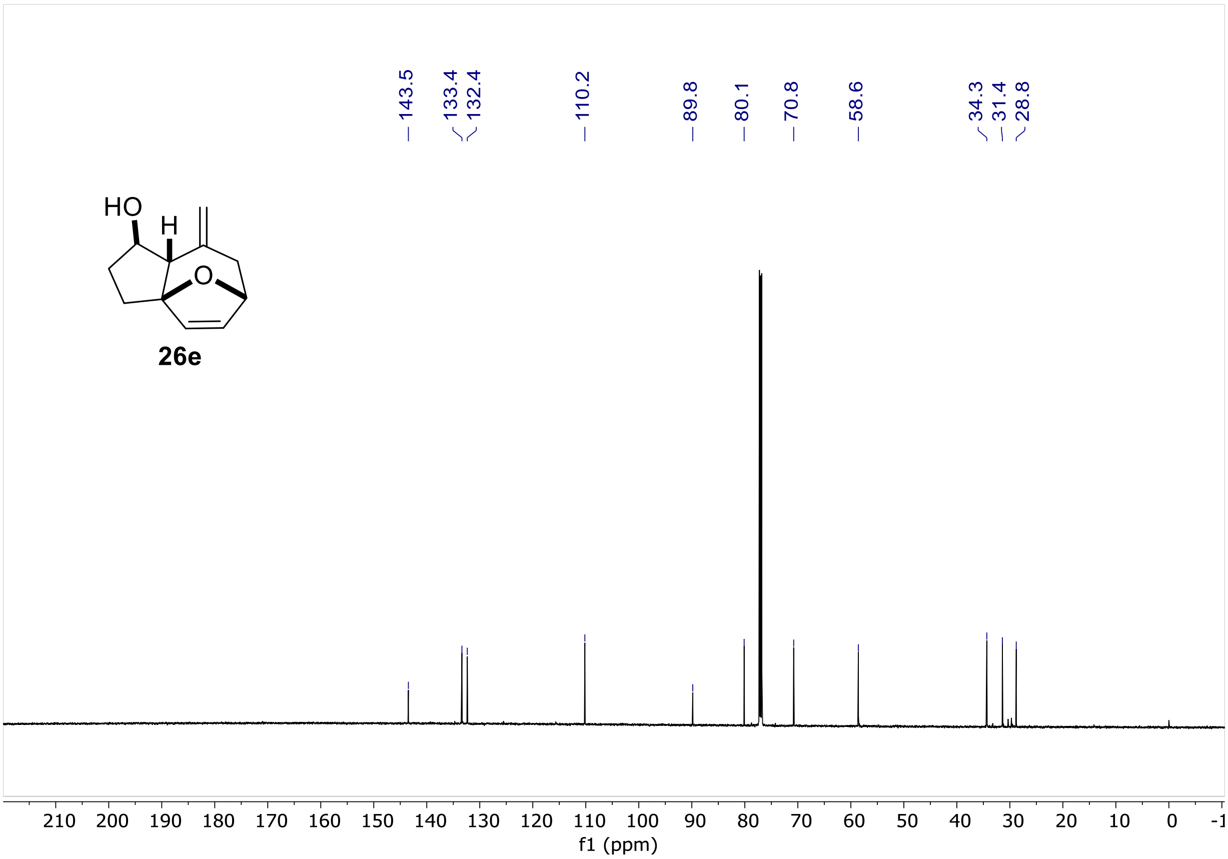

^{1}H NMR (600 MHz, $CDCl_3$): **27e**

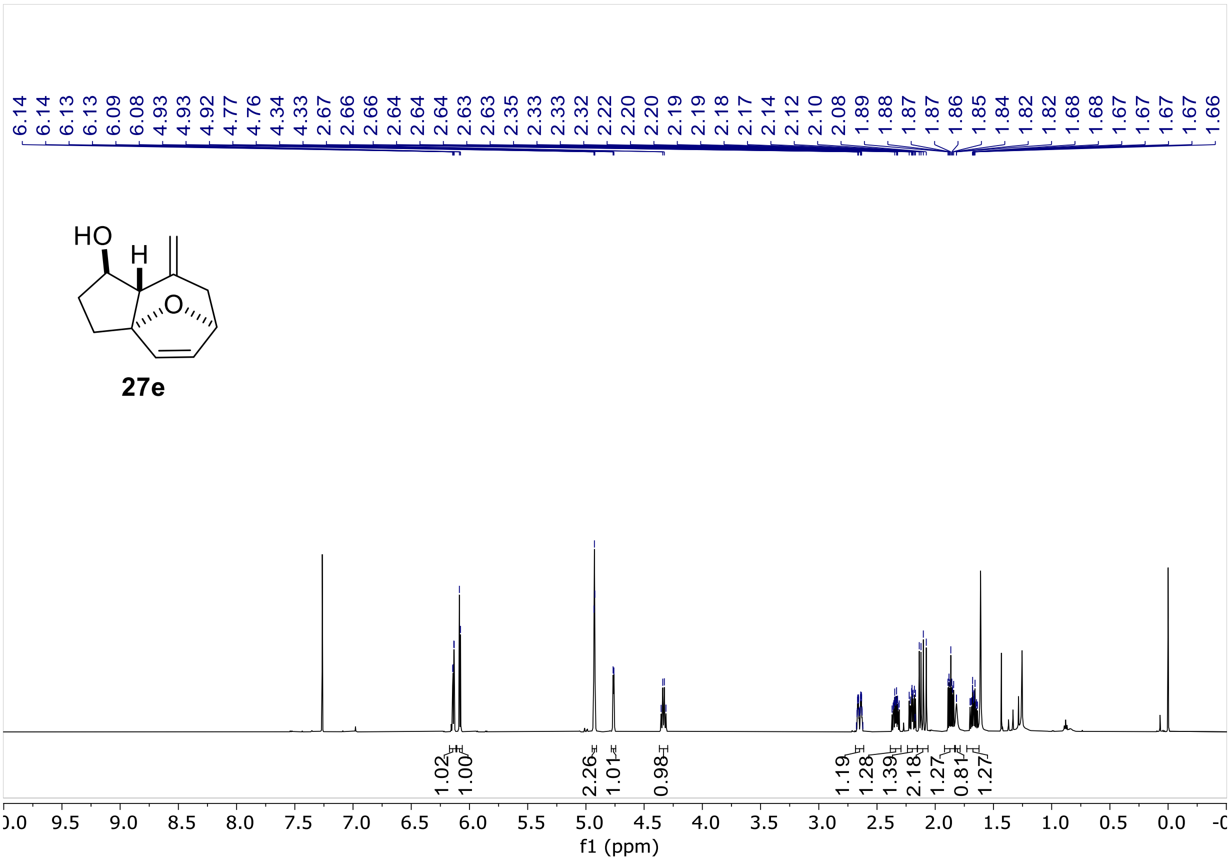

^{13}C{^{1}H} NMR (150 MHz, $CDCl_3$): **27e**

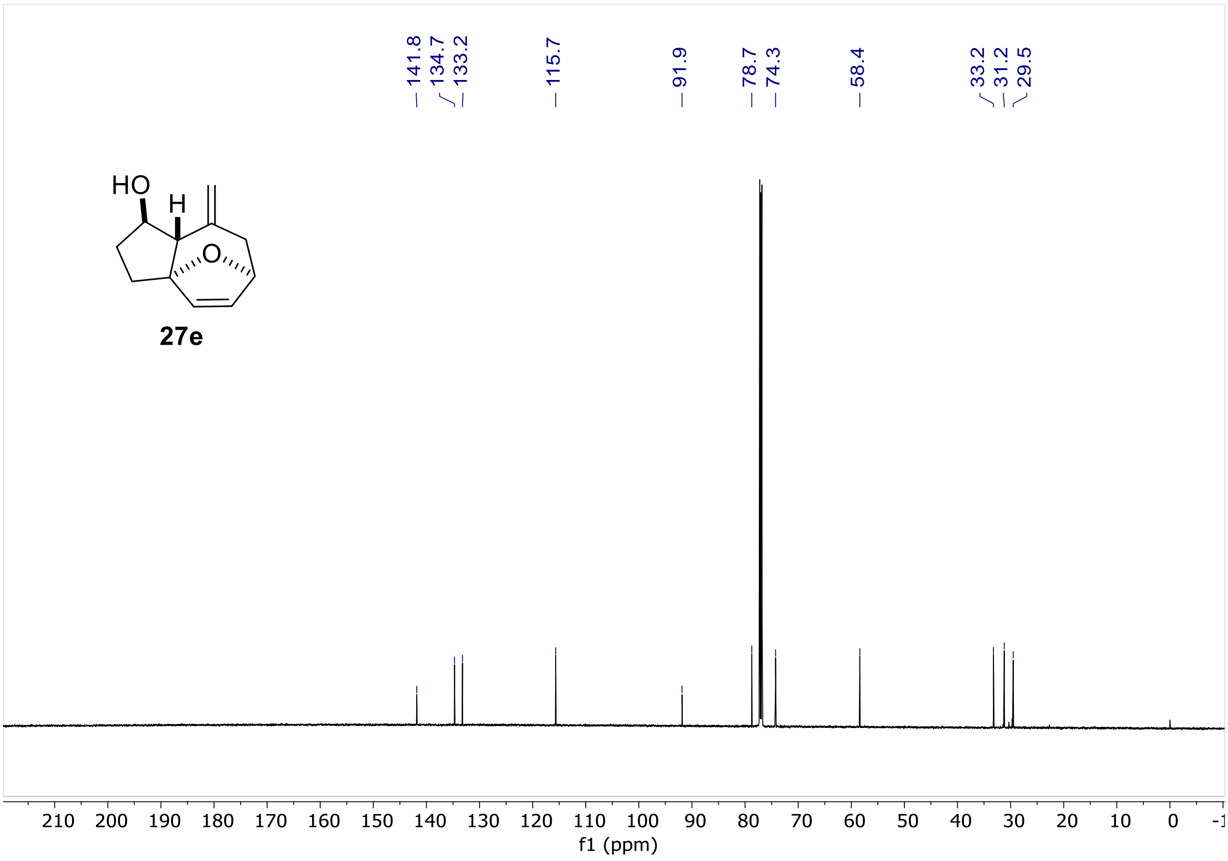

^{1}H NMR (600 MHz, $CDCl_3$): **26f**

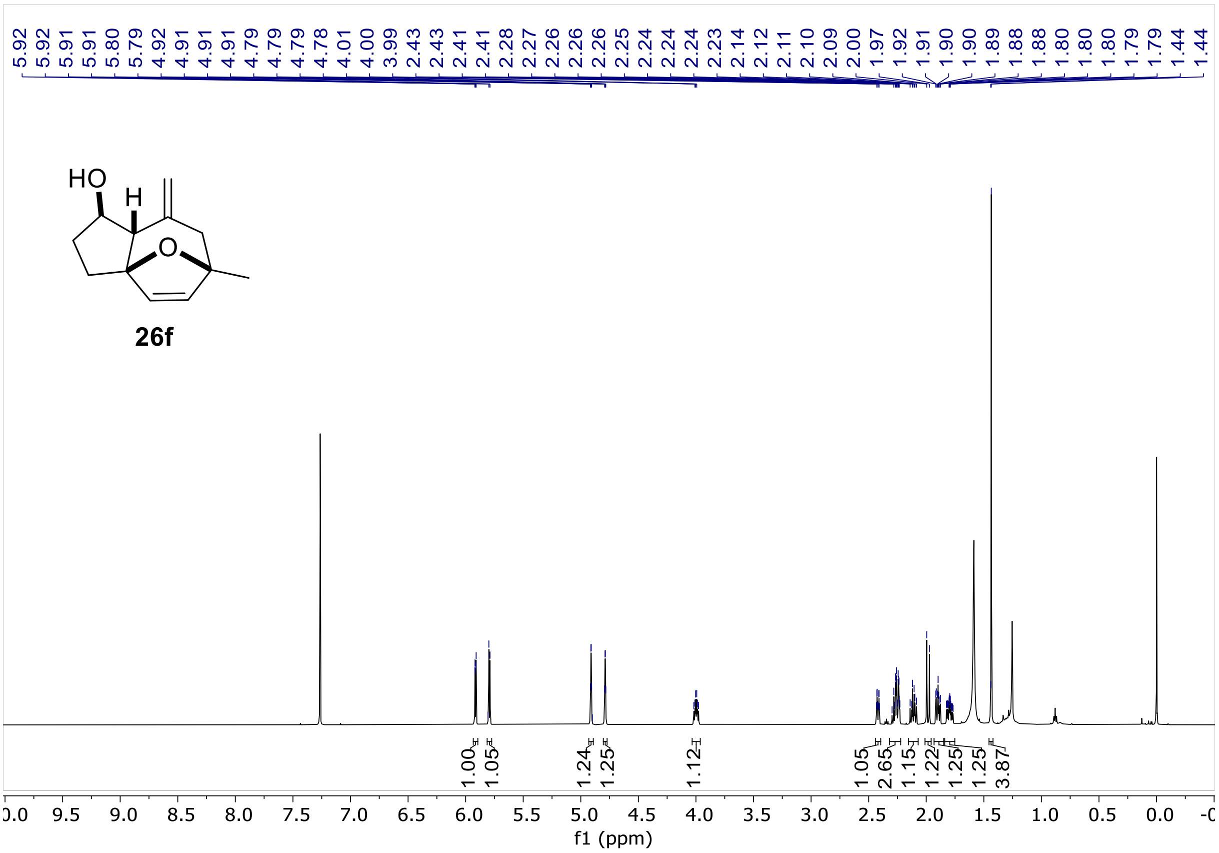

$^{13}C\{^{1}H\}$ NMR (150 MHz, $CDCl_3$): **26f**

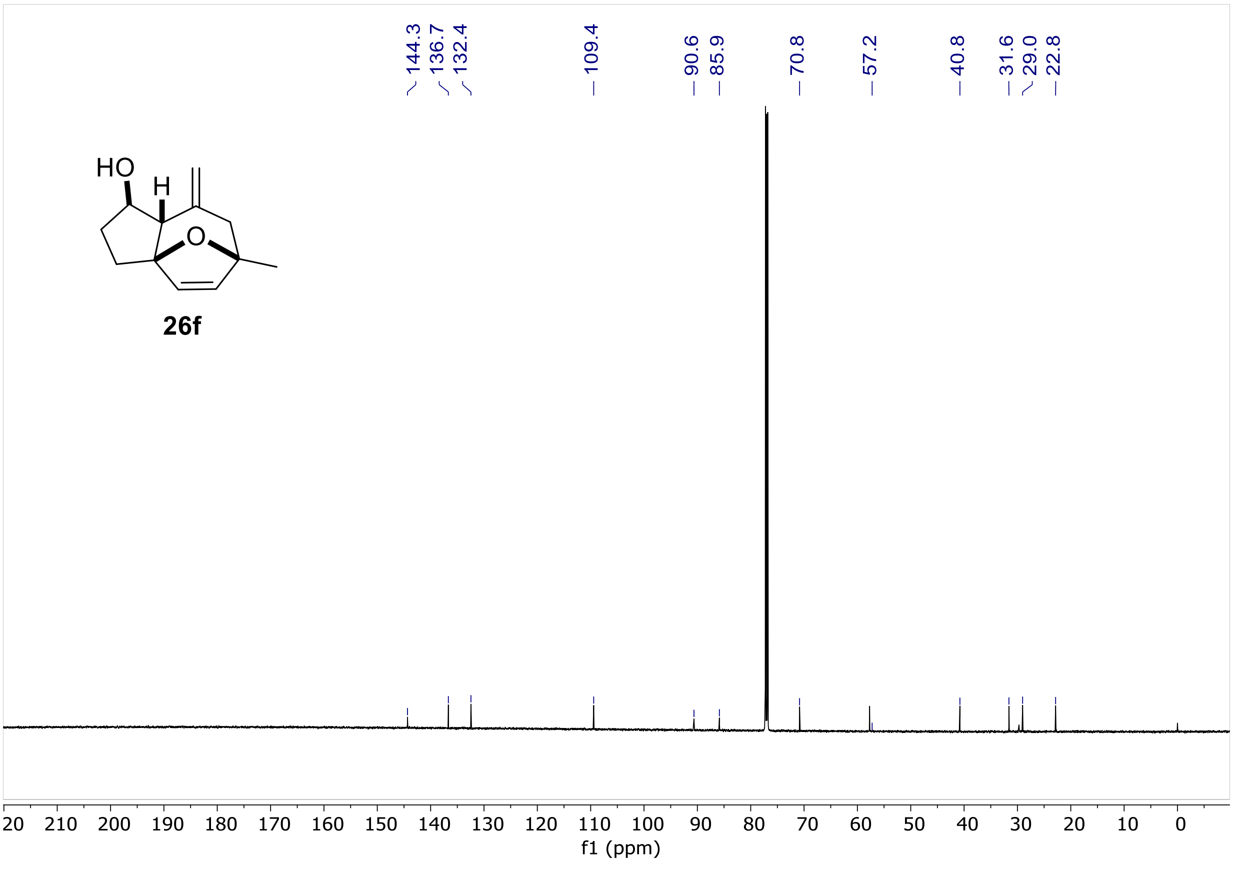

^{1}H NMR (600 MHz, $CDCl_3$): **27f**

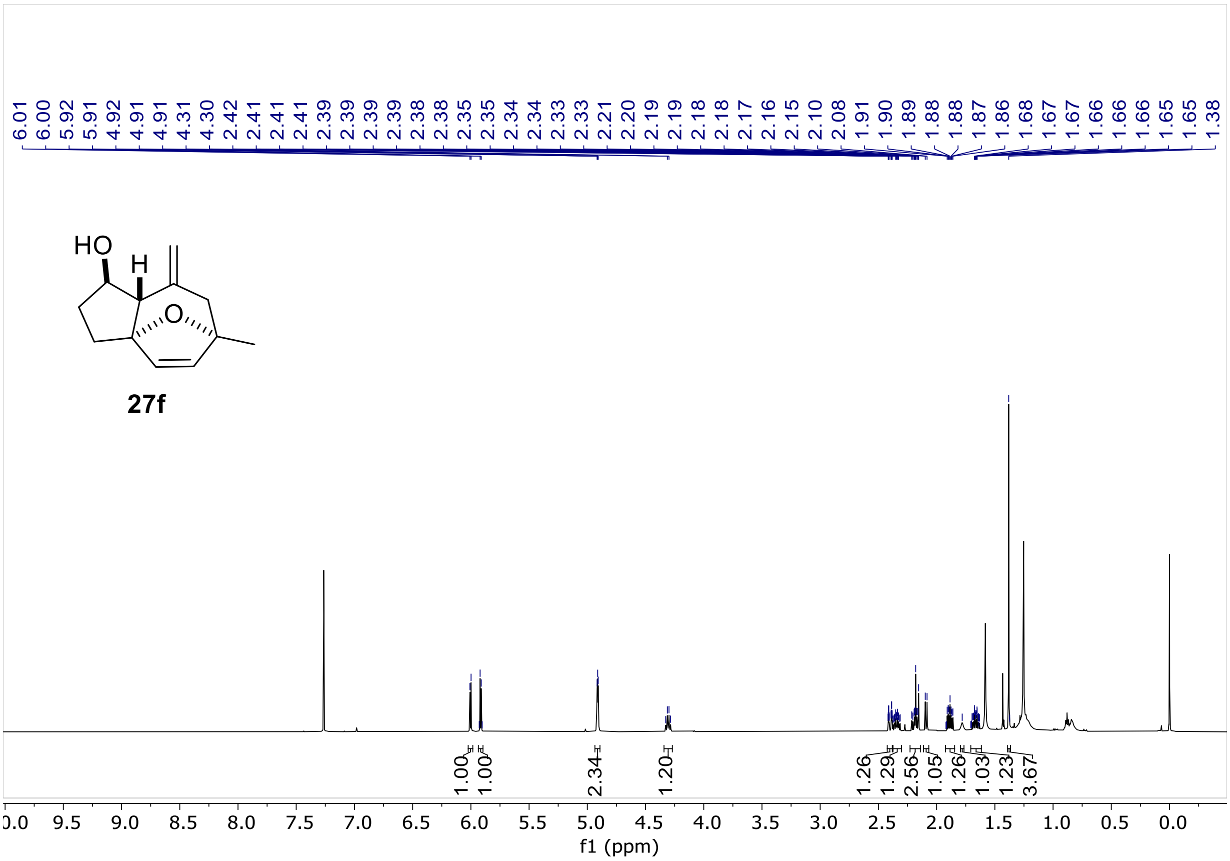

$^{13}C\{^{1}H\}$ NMR (150 MHz, $CDCl_3$): **27f**

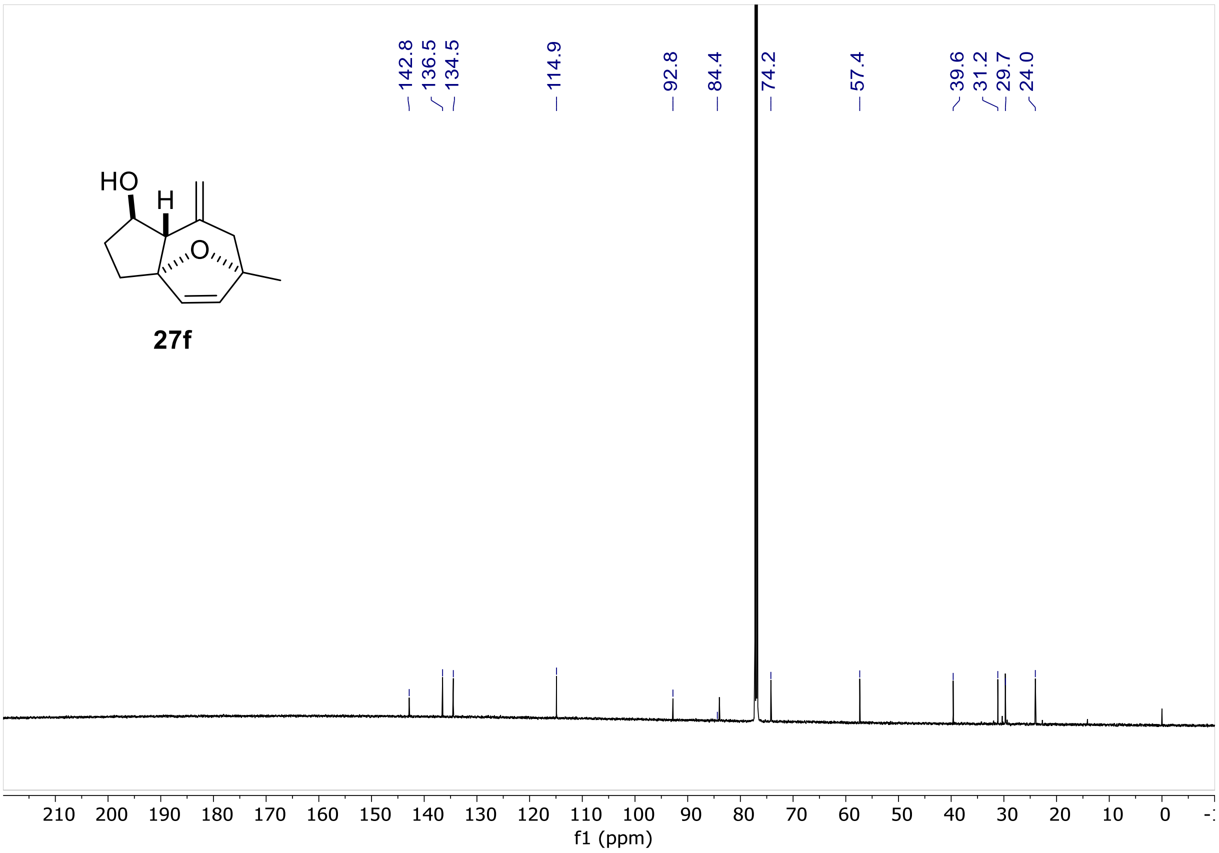

^{1}H NMR (600 MHz, $CDCl_3$): **26g**

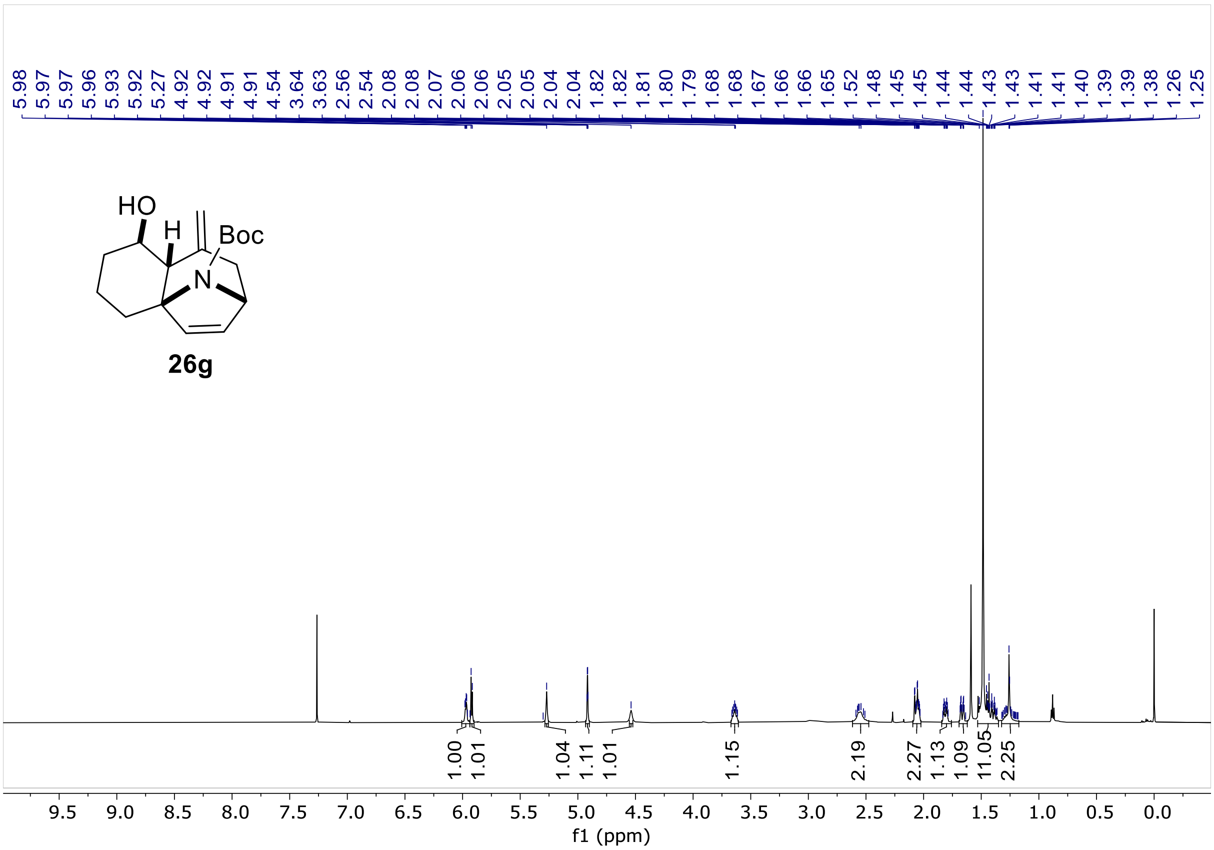

^{13}C{^{1}H} NMR (150 MHz, $CDCl_3$): **26g**

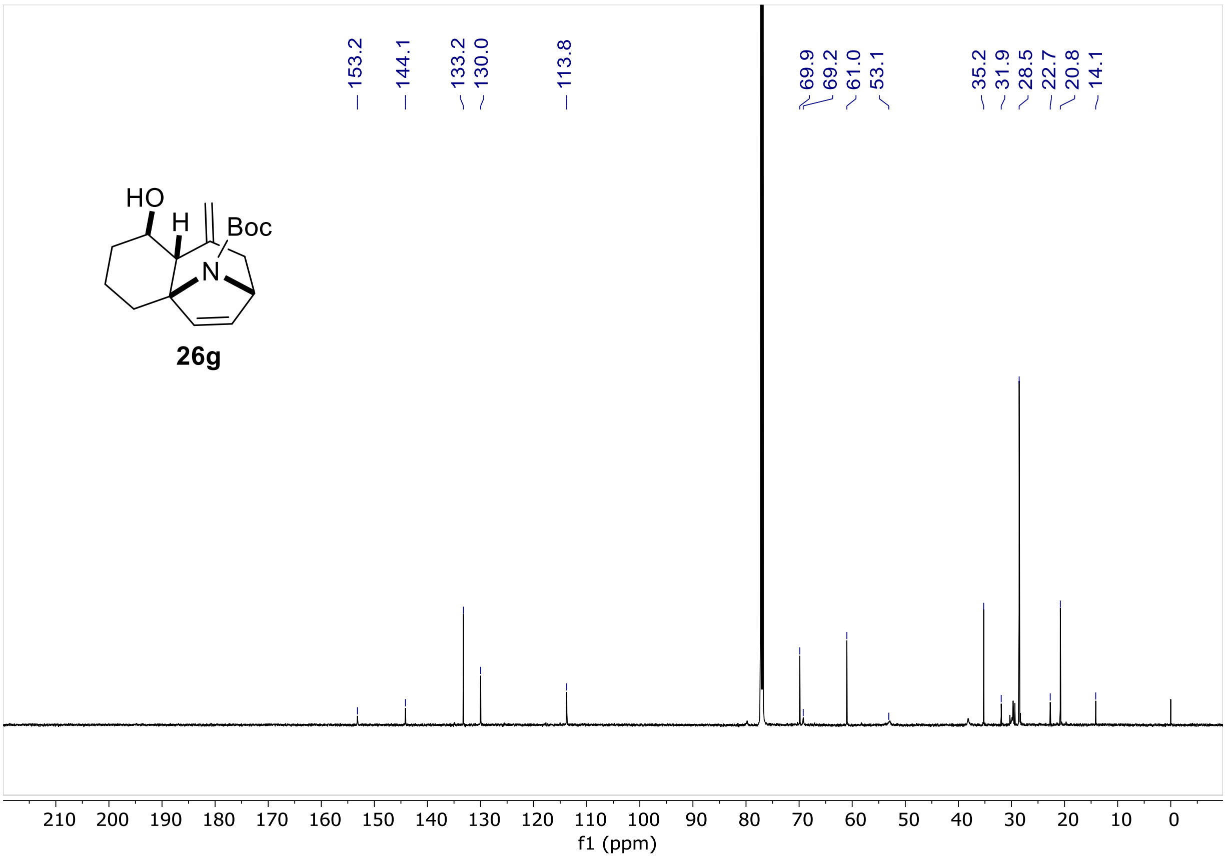

^{1}H NMR (600 MHz, $CDCl_3$): **27g**

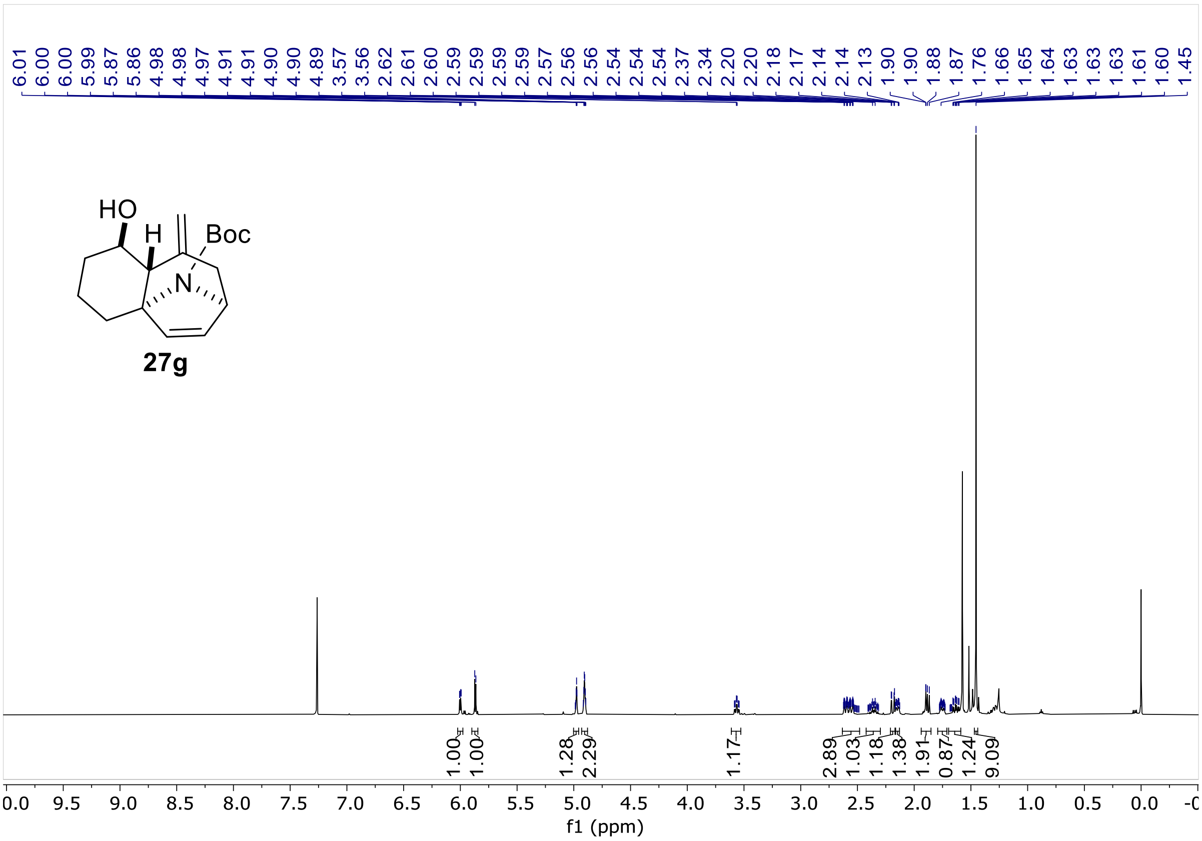

^{13}C{^{1}H} NMR (150 MHz, $CDCl_3$): **27g**

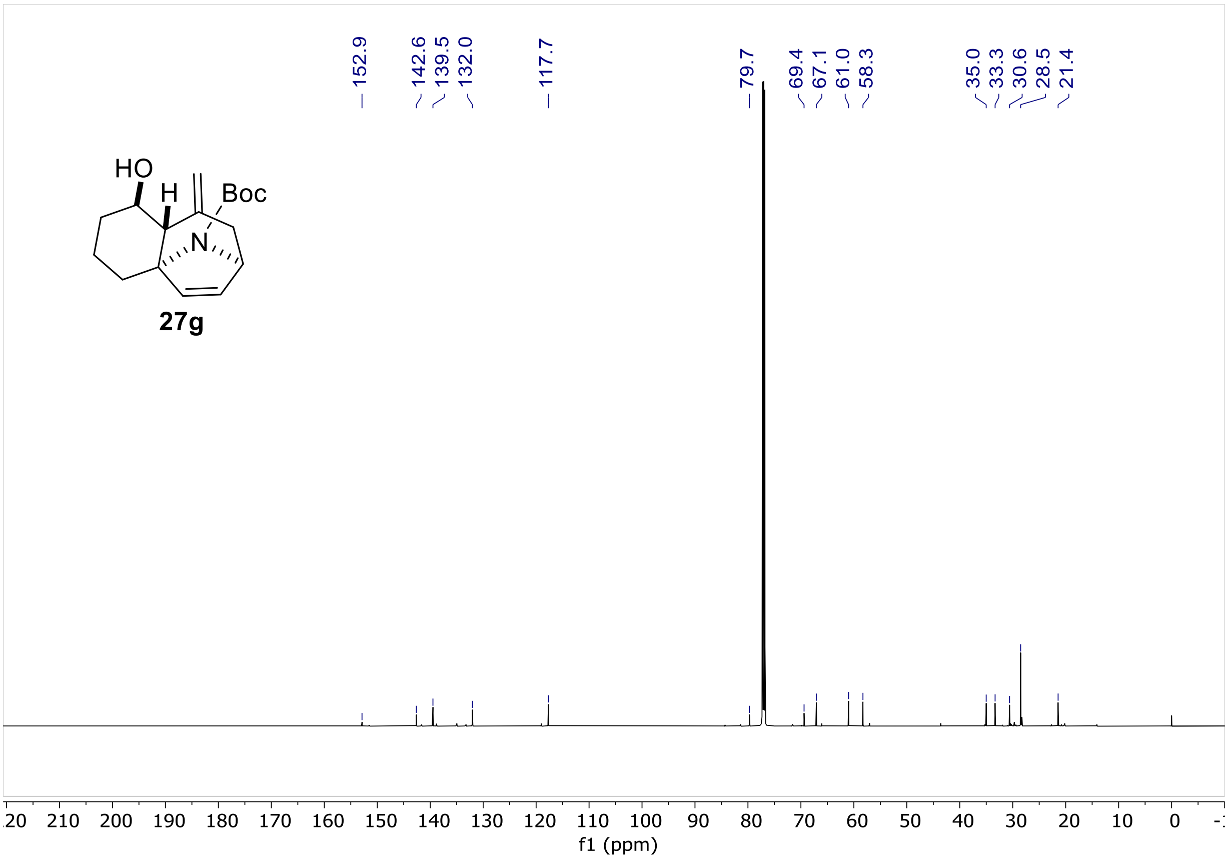

^{1}H NMR (600 MHz, $CDCl_3$): **26h**

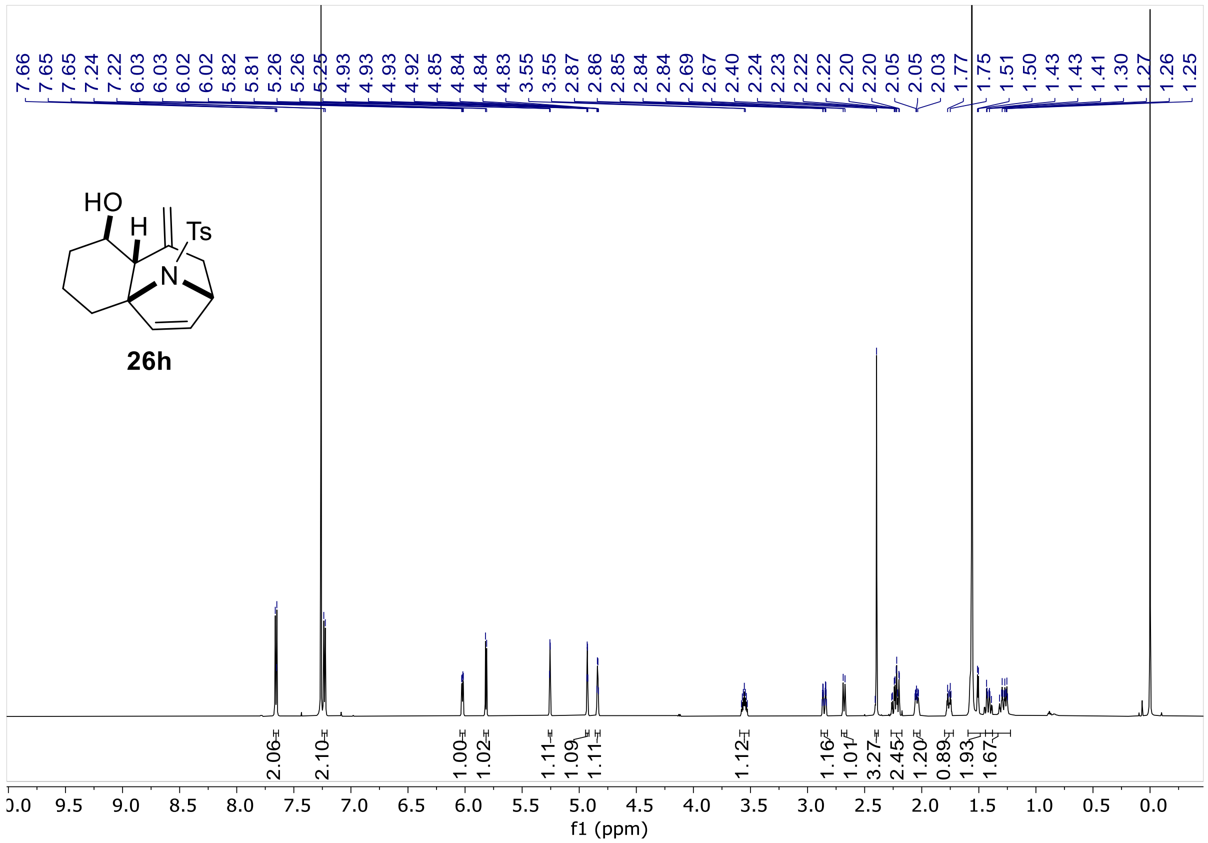

$^{13}C\{^{1}H\}$ NMR (150 MHz, $CDCl_3$): **26h**

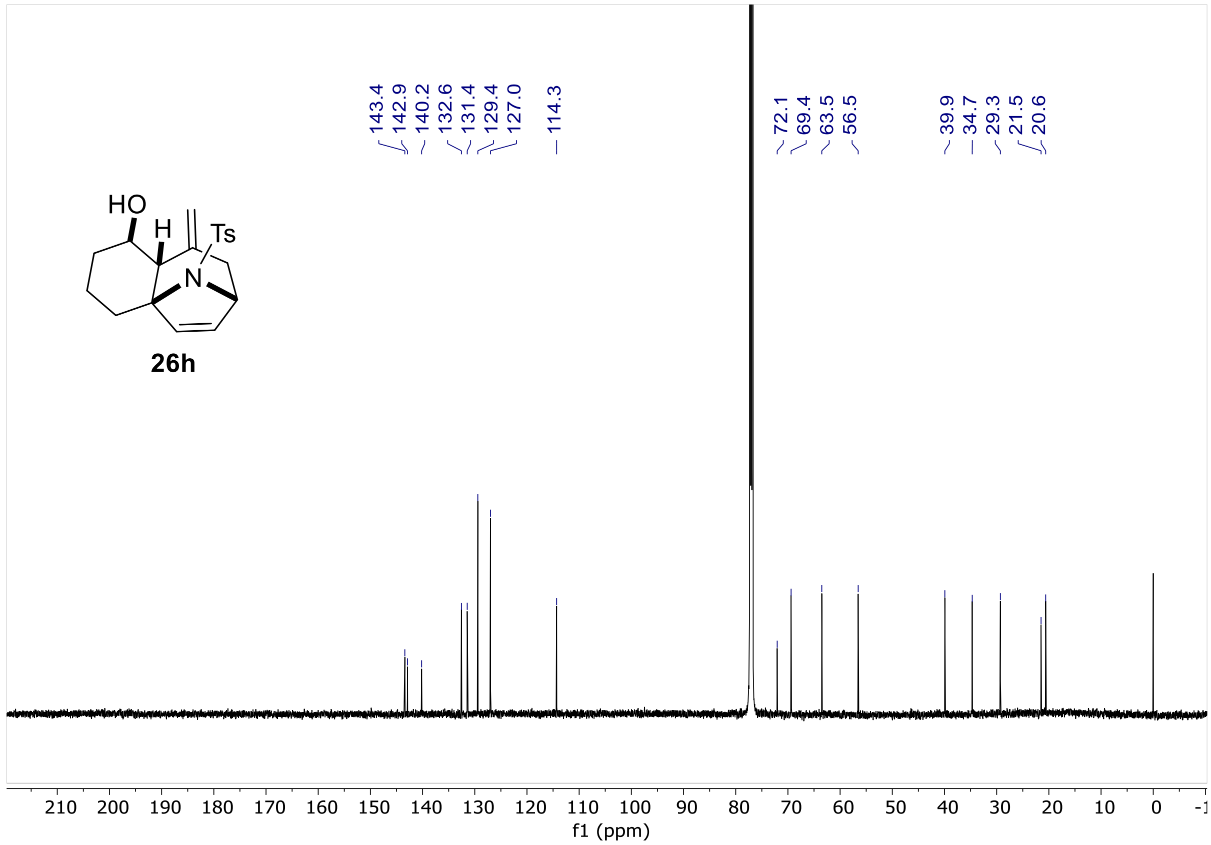

^{1}H NMR (600 MHz, $CDCl_3$): **27h**

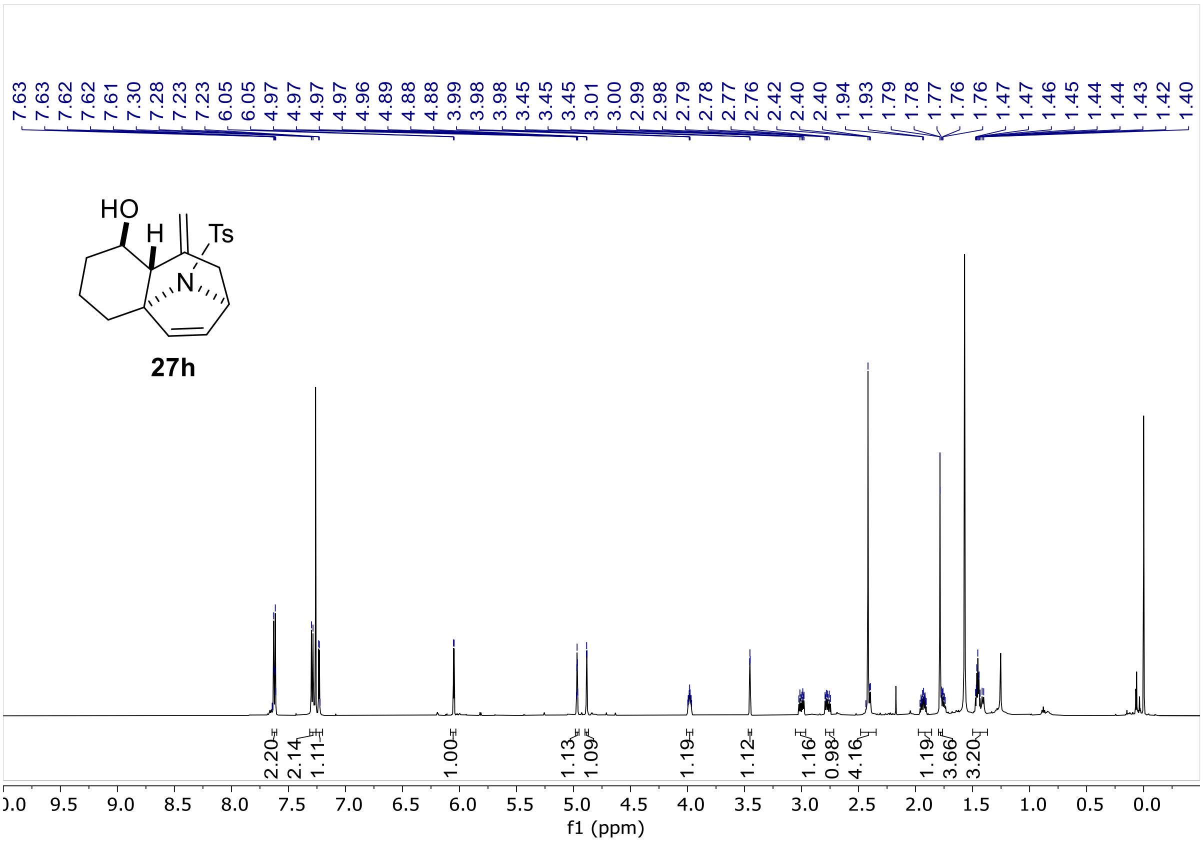

^{13}C{^{1}H} NMR (150 MHz, $CDCl_3$): **27h**

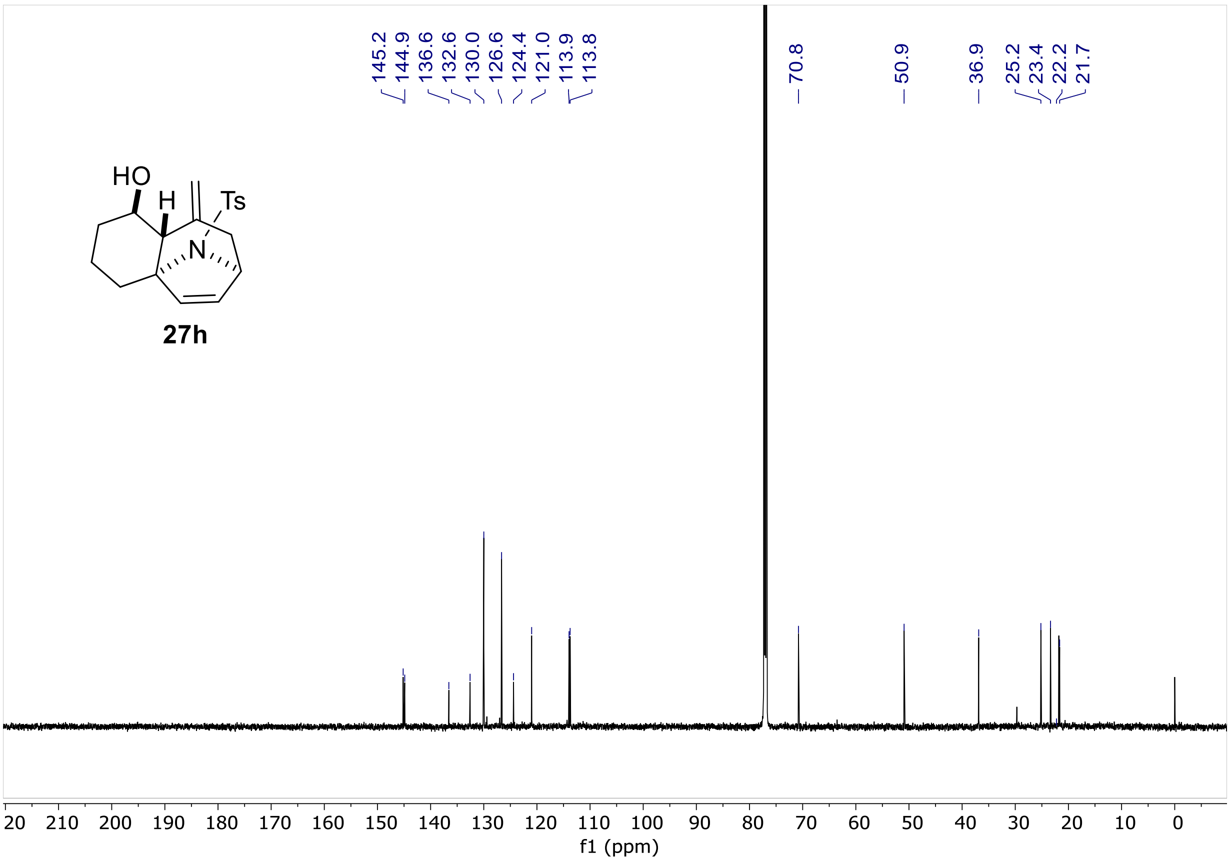

^{1}H NMR (600 MHz, $CDCl_3$): **26i**

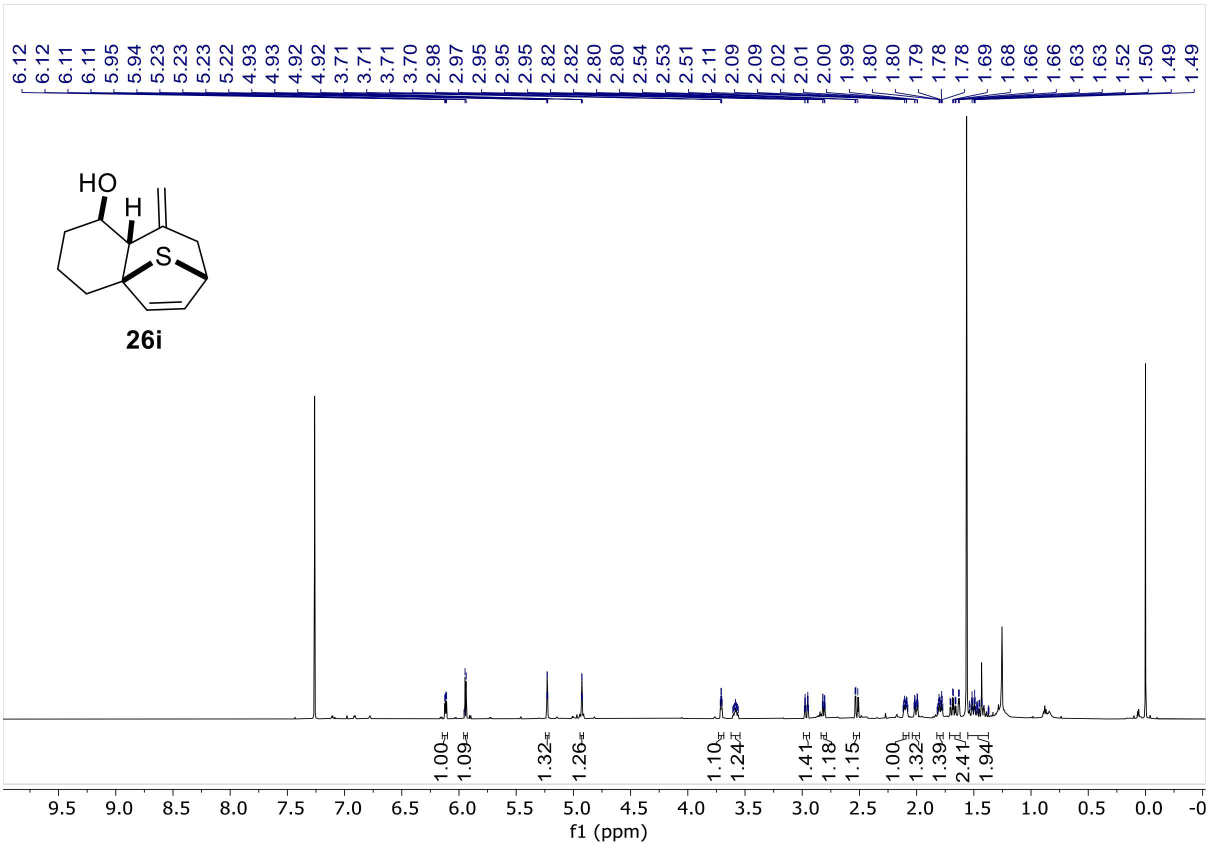

^{13}C{^{1}H} NMR (150 MHz, $CDCl_3$): **26i**

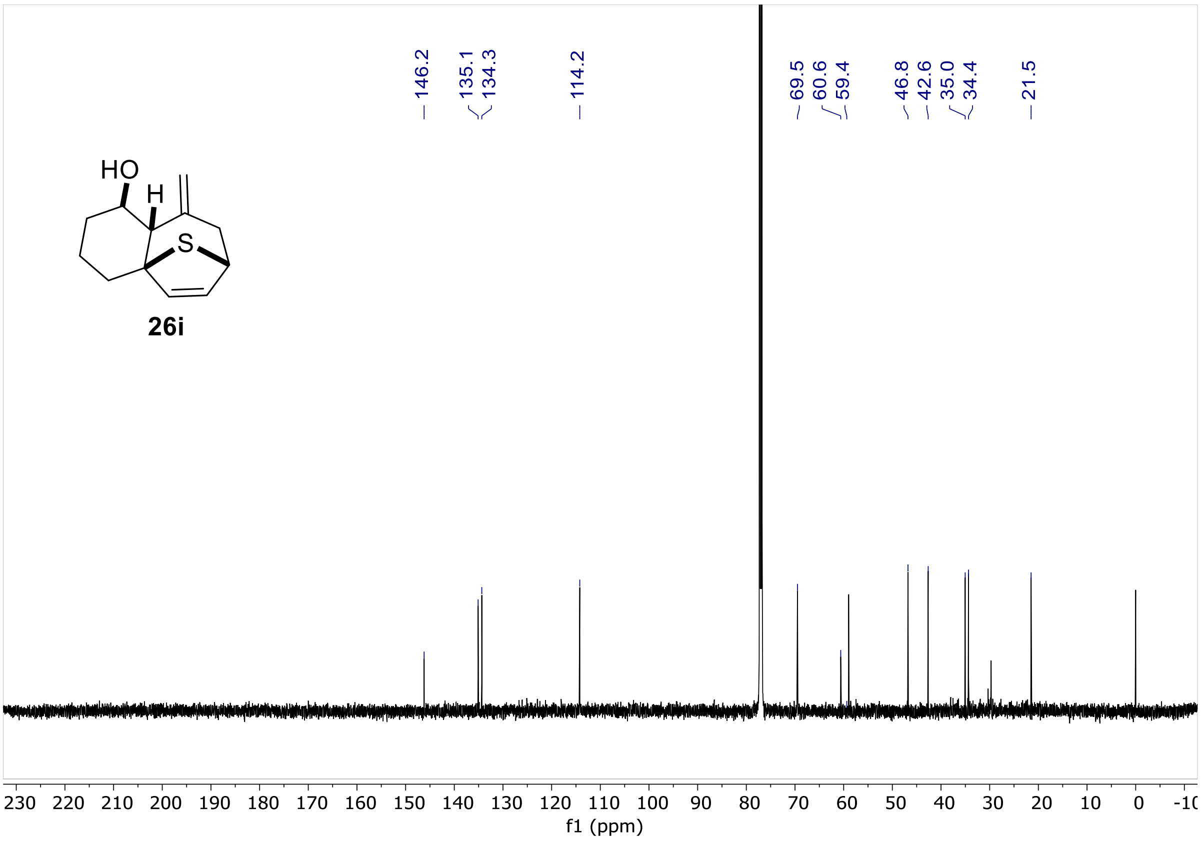

^{1}H NMR (600 MHz, $CDCl_3$): **27i**

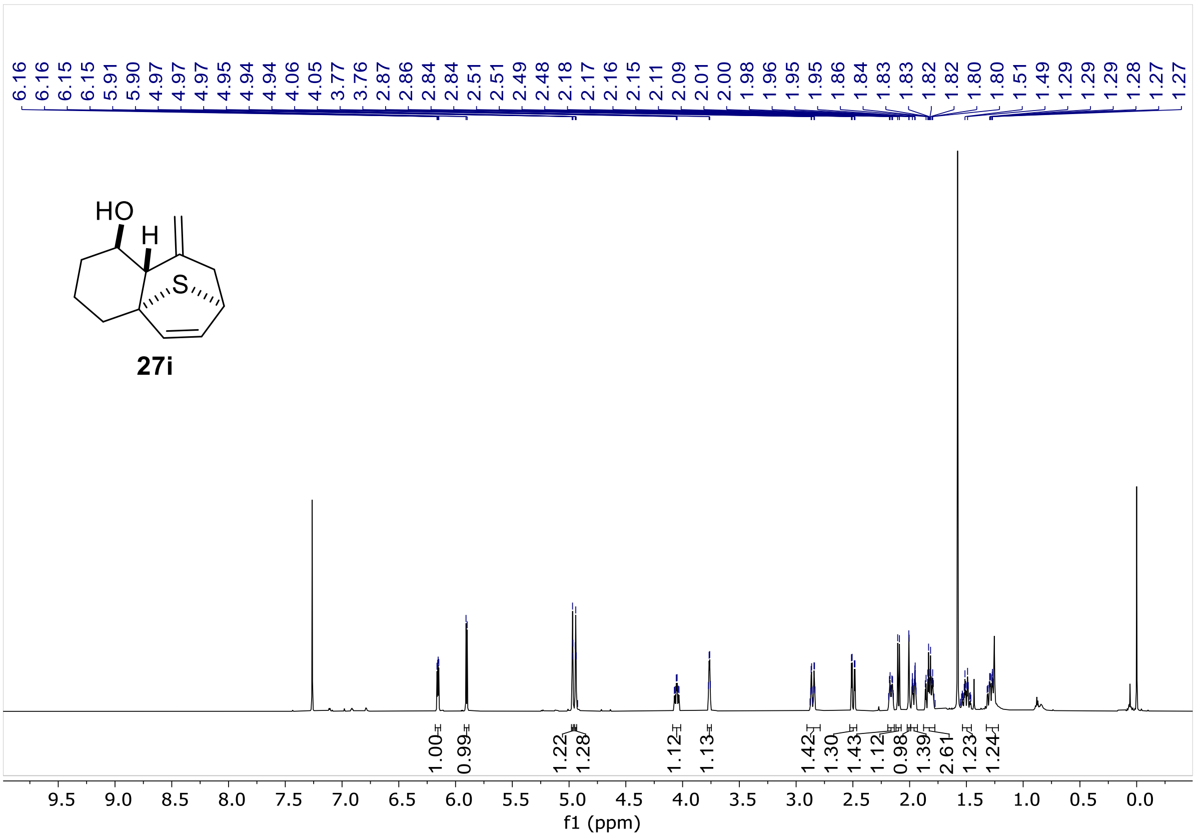

$^{13}C\{^{1}H\}$ NMR (150 MHz, $CDCl_3$): **27i**

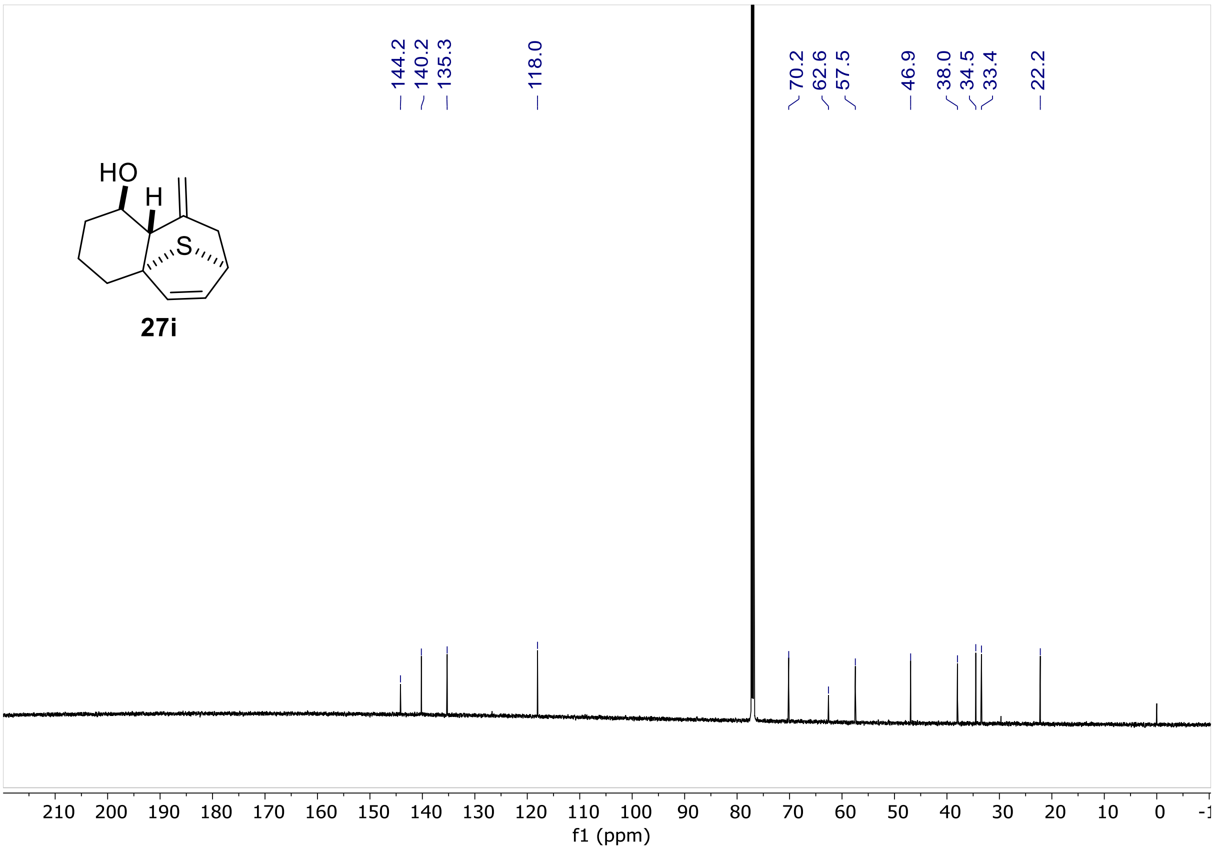

^{1}H NMR (600 MHz, $CDCl_3$): **27j**

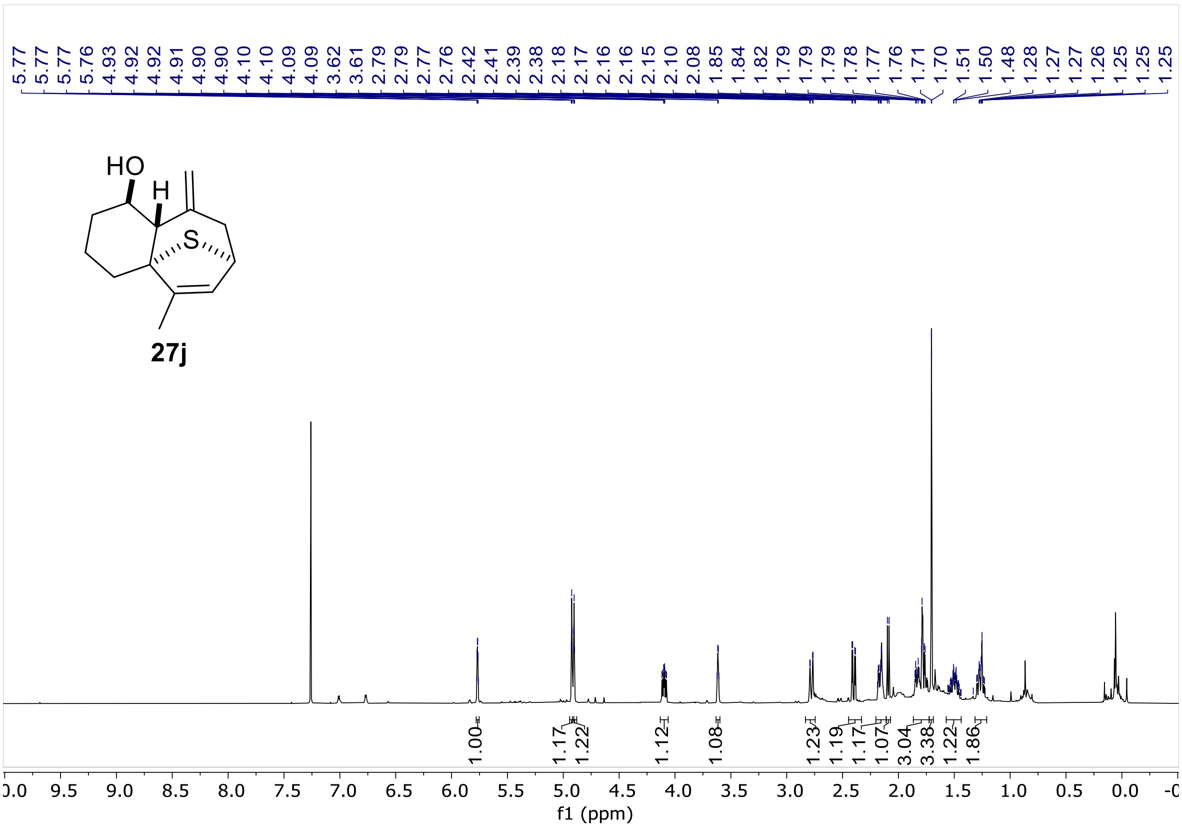

^{13}C{^{1}H} NMR (150 MHz, $CDCl_3$): **27j**

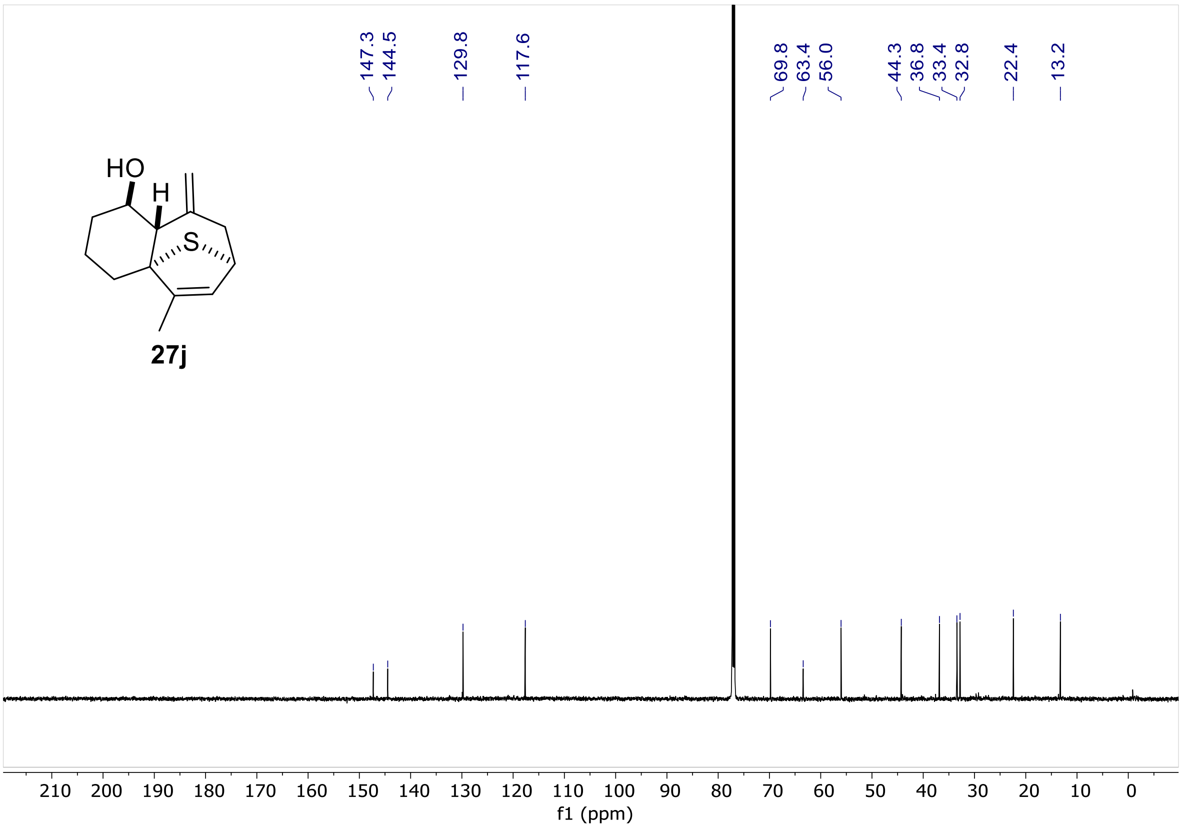

^{1}H NMR (600 MHz, $CDCl_3$): **27k**

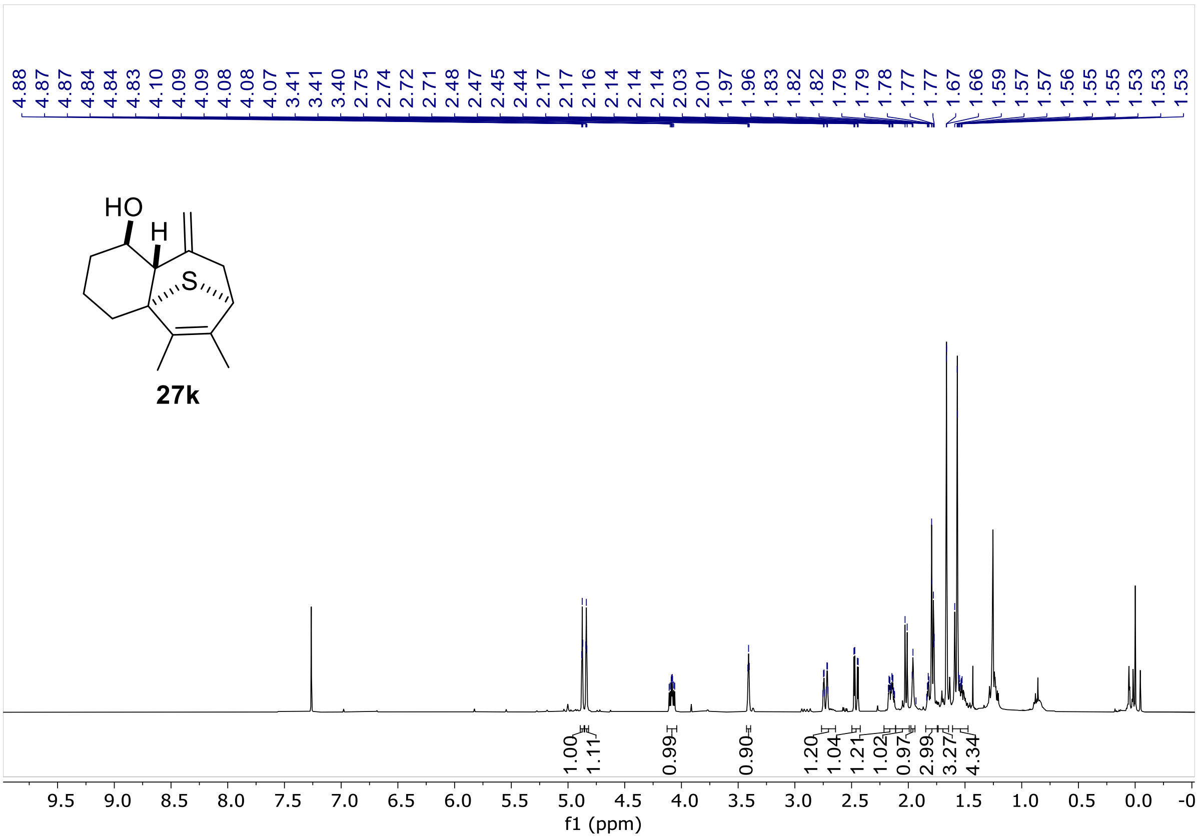

^{13}C{^{1}H} NMR (150 MHz, $CDCl_3$): **27k**

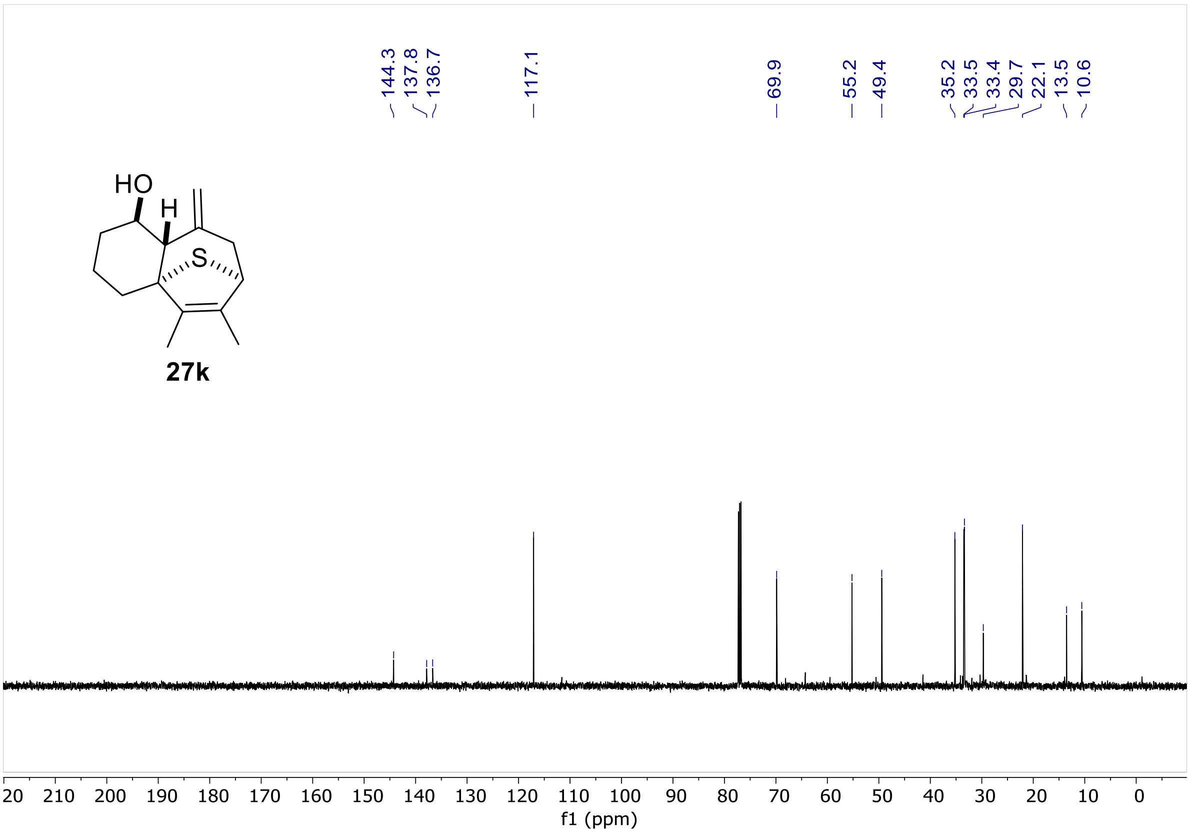

^{1}H NMR (400 MHz, $CDCl_3$): **30a**

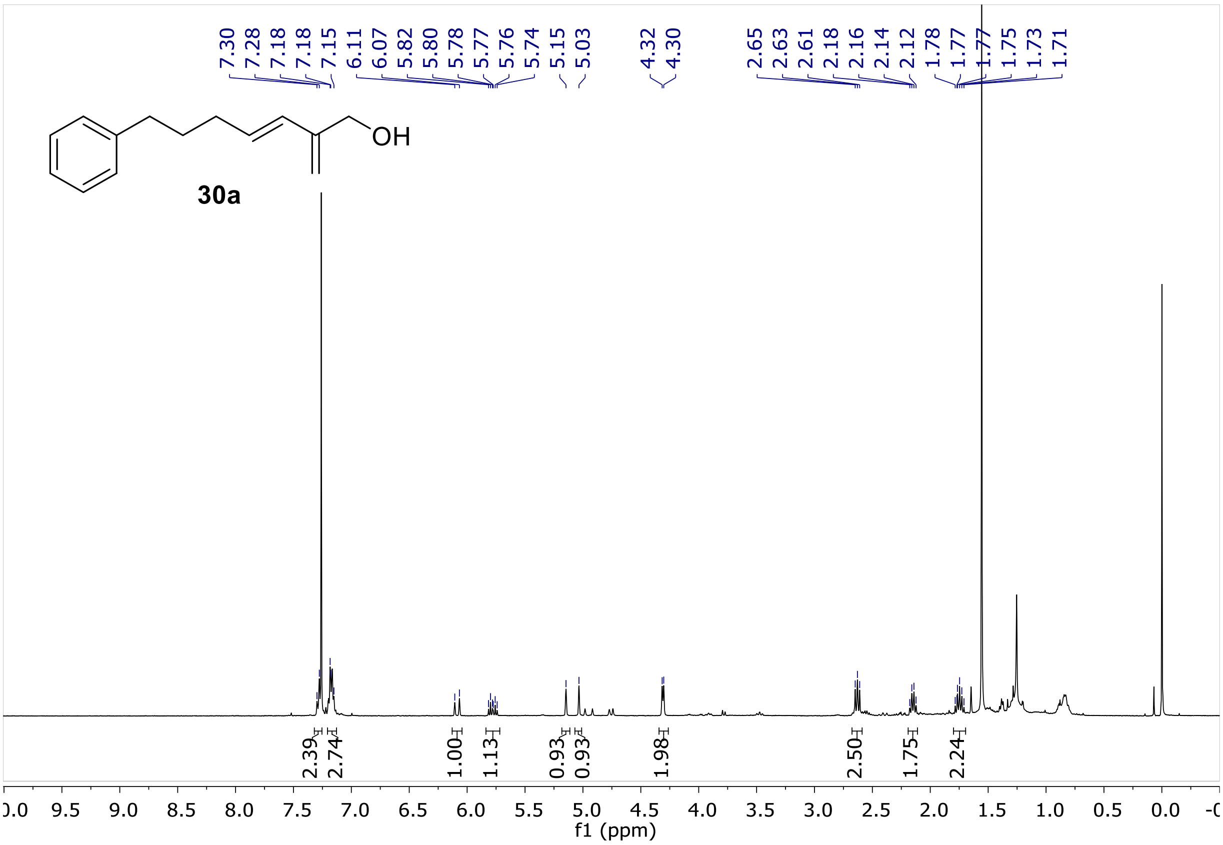

$^{13}C\{^{1}H\}$ NMR (150 MHz, $CDCl_3$): **30a**

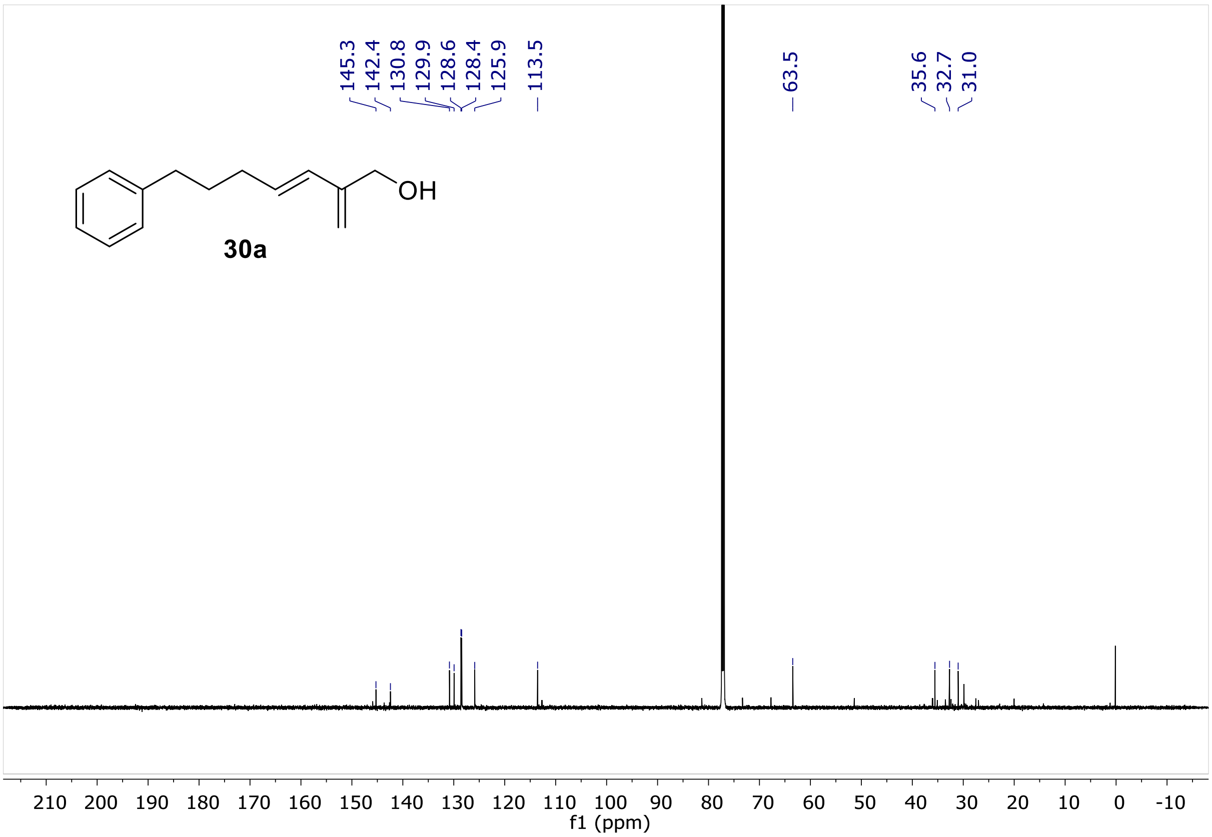

^{1}H NMR (600 MHz, $CDCl_3$): **30b**

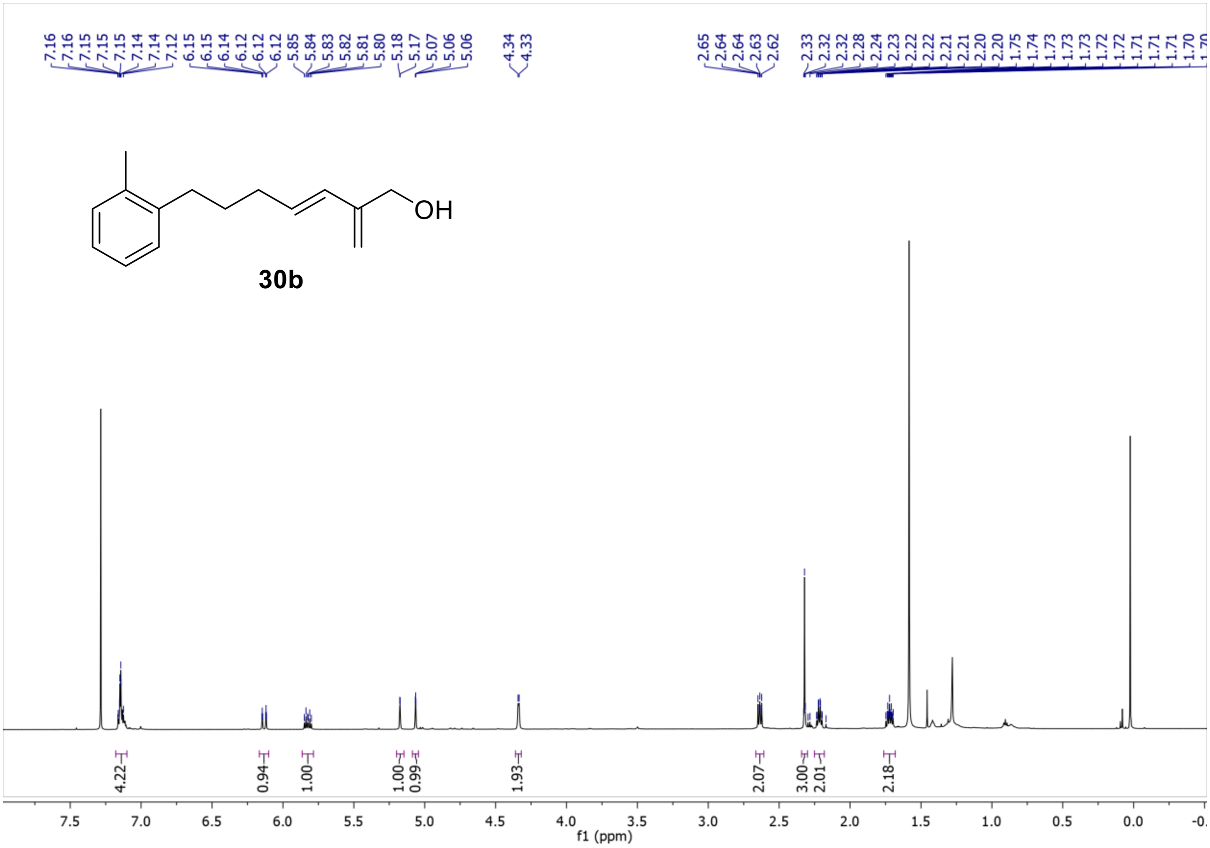

^{13}C{^{1}H} NMR (150 MHz, $CDCl_3$): **30b**

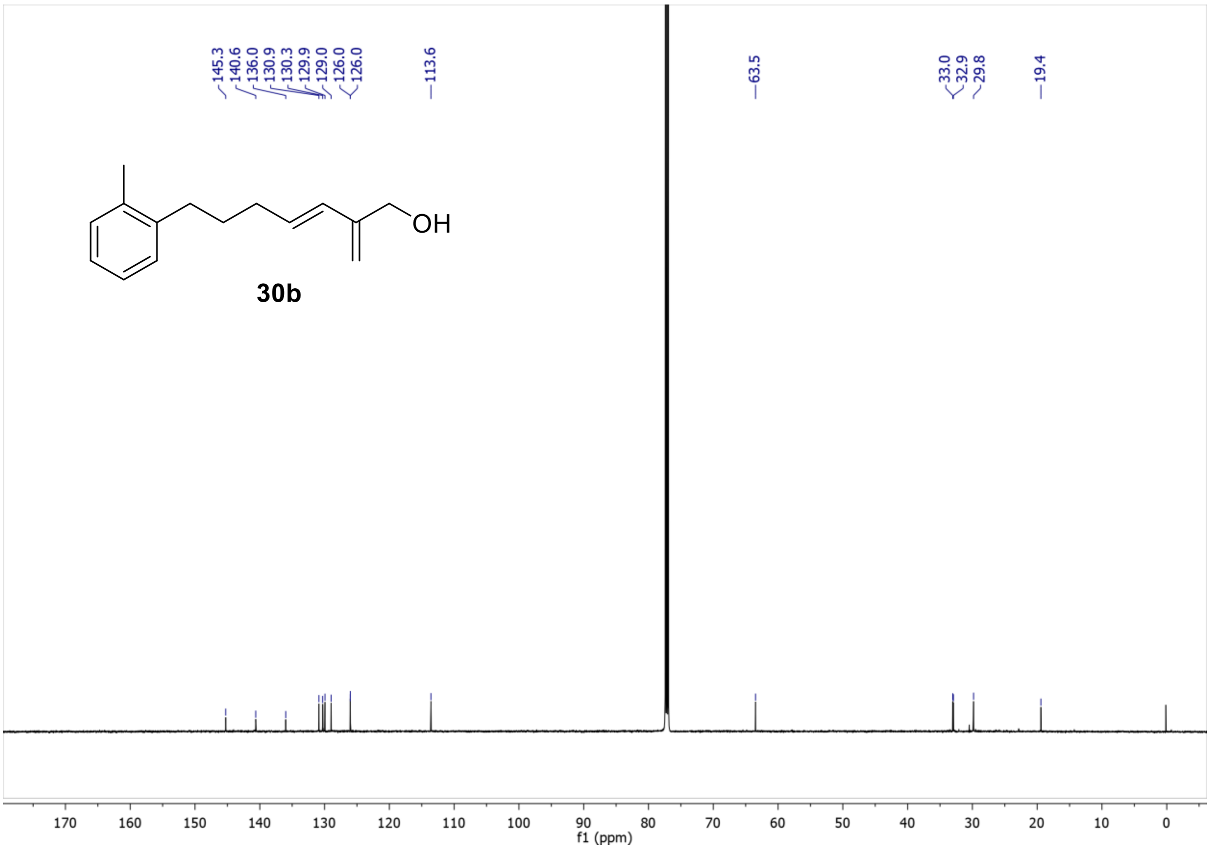

^{1}H NMR (600 MHz, $CDCl_3$): **30c**

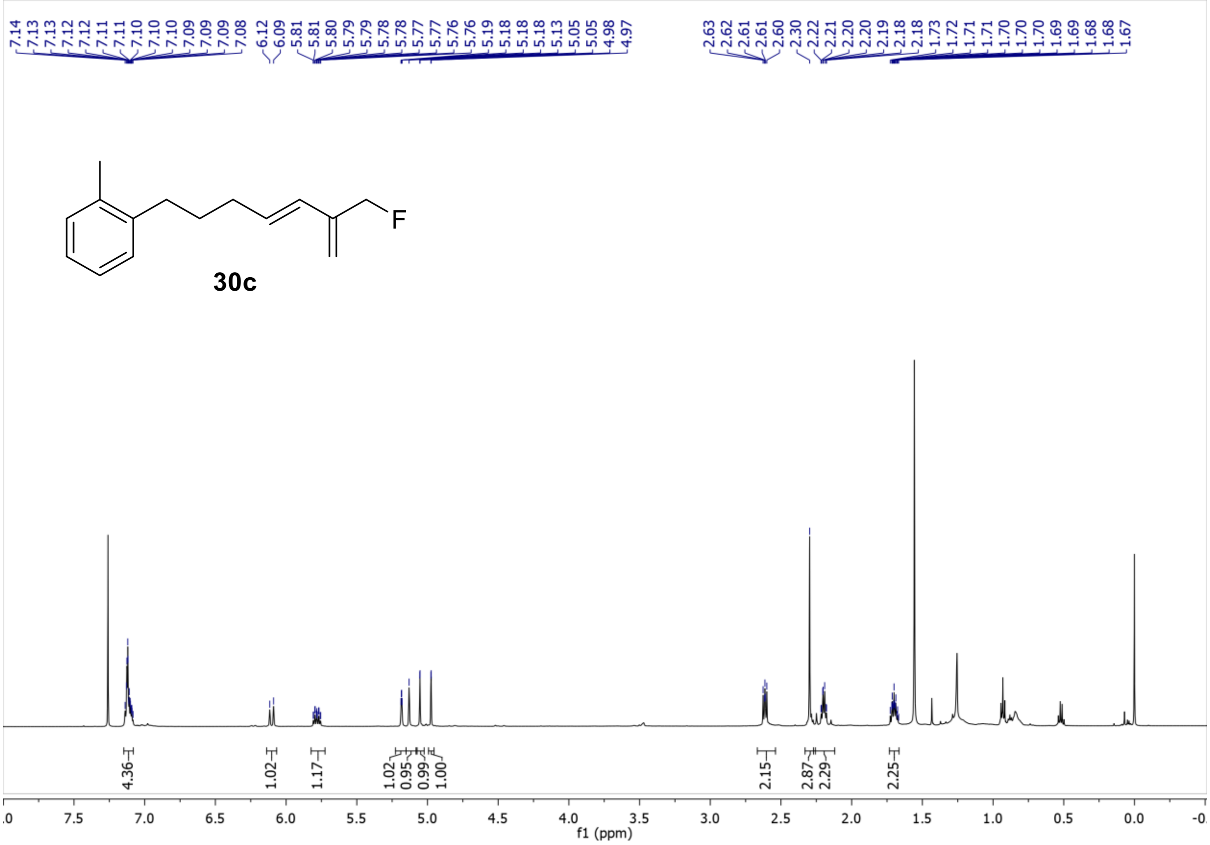

^{13}C{^{1}H} NMR (150 MHz, $CDCl_3$): **30c**

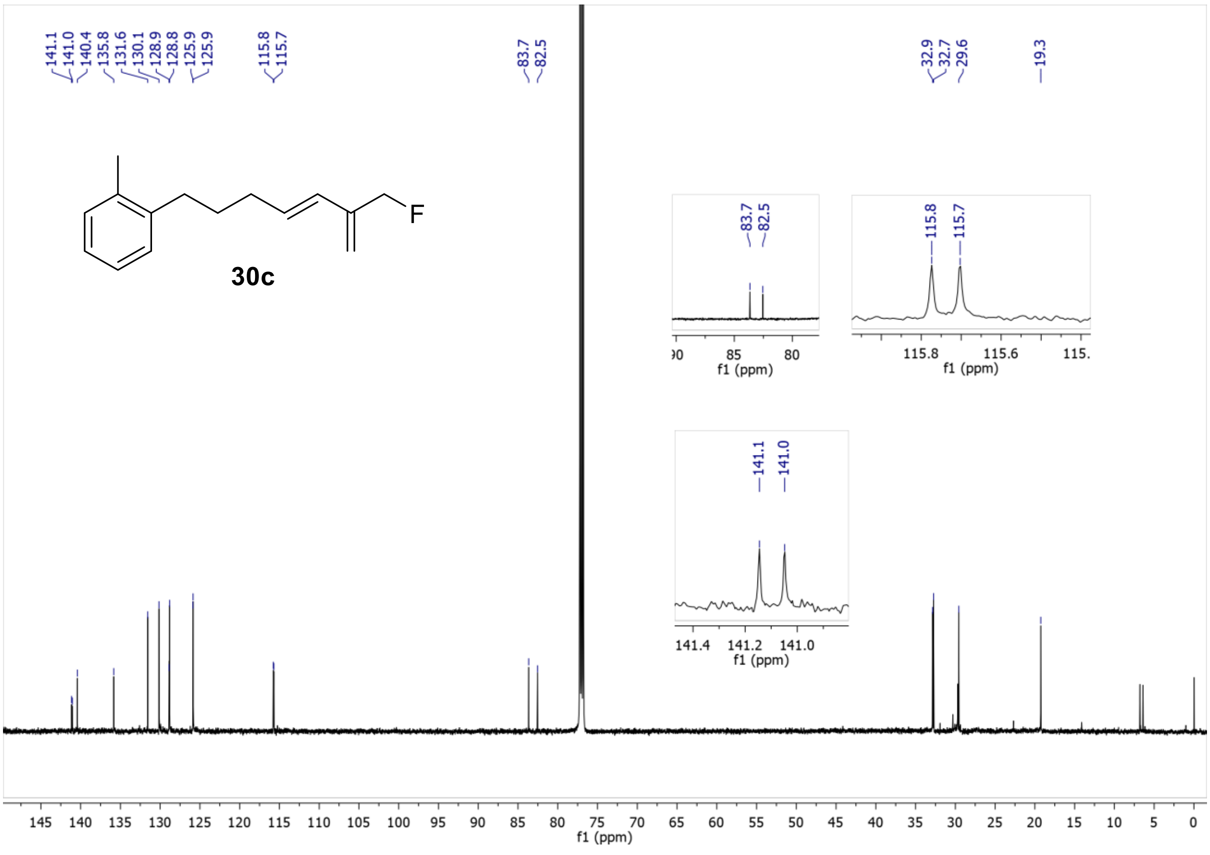

^{19}F NMR (564 MHz, $CDCl_3$): **30c**

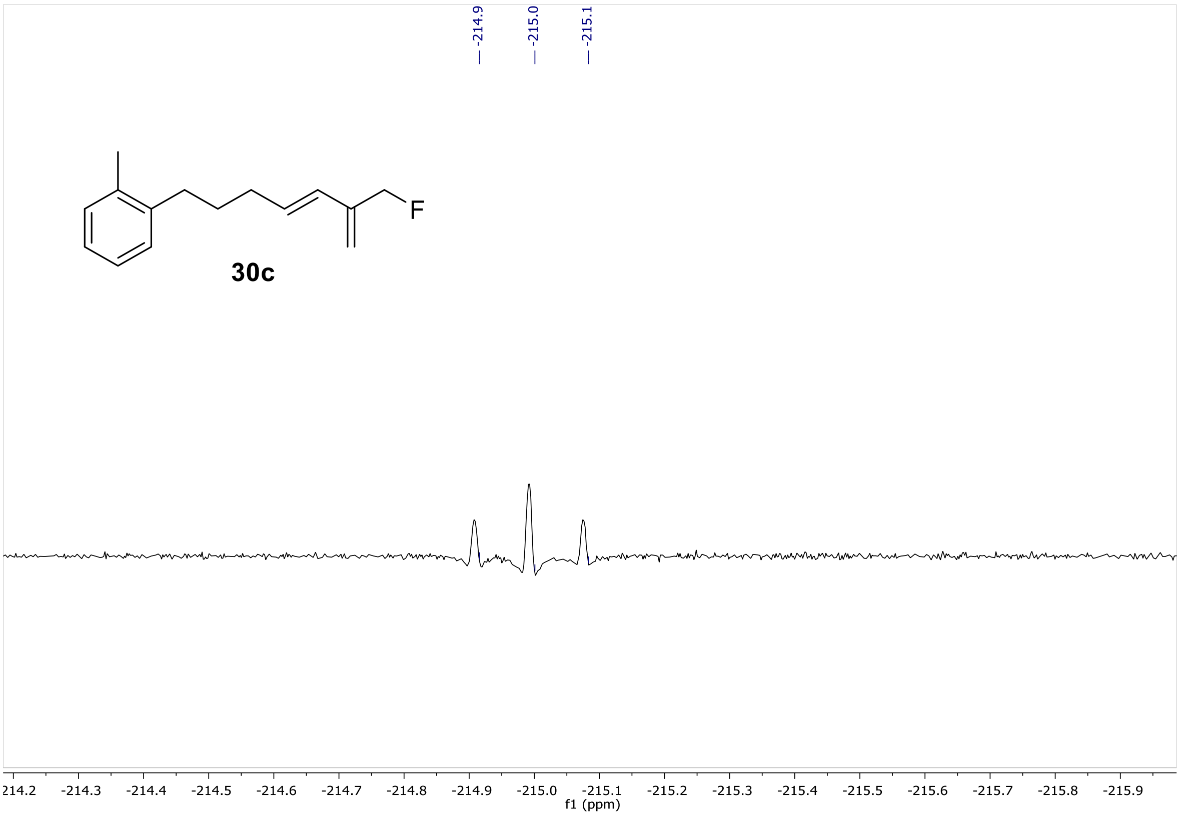

^{1}H NMR (600 MHz, $CDCl_3$): **26o**

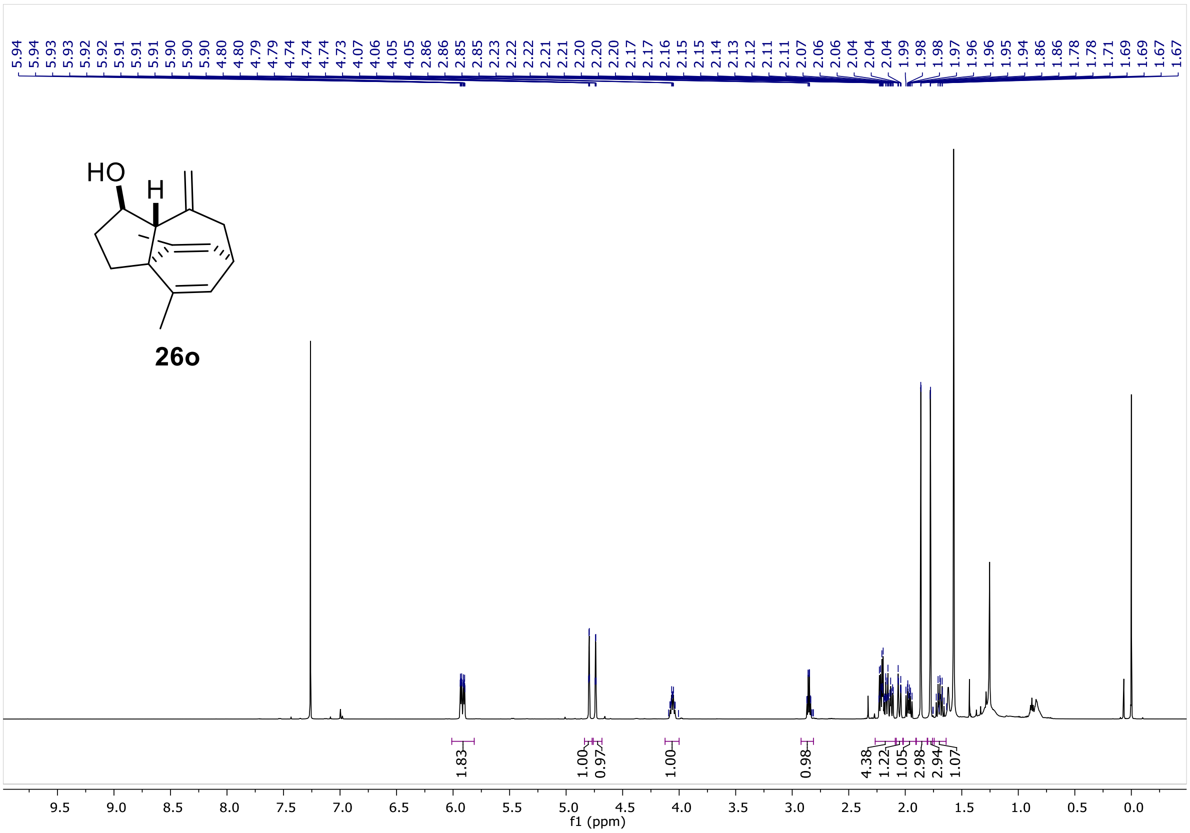

$^{13}C\{^{1}H\}$ NMR (150 MHz, $CDCl_3$): **26o**

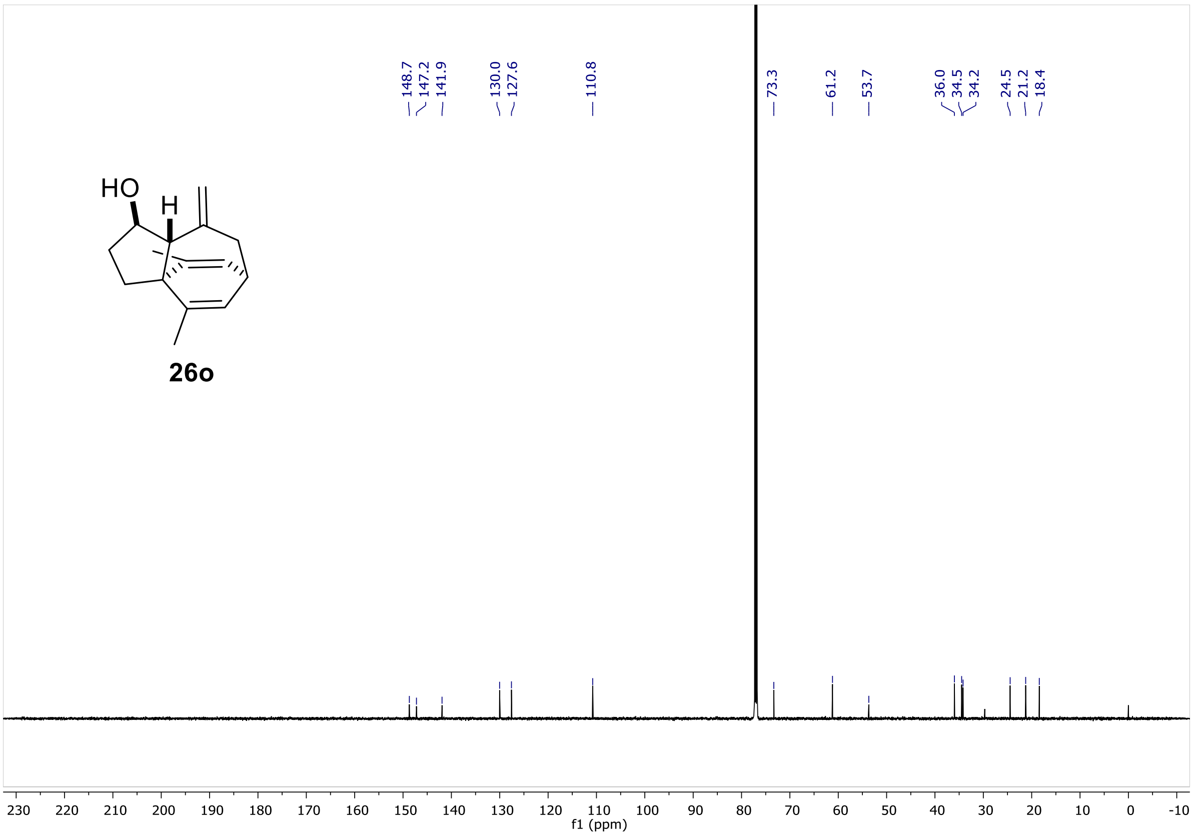

^{1}H NMR (400 MHz, $CDCl_3$): **30d**

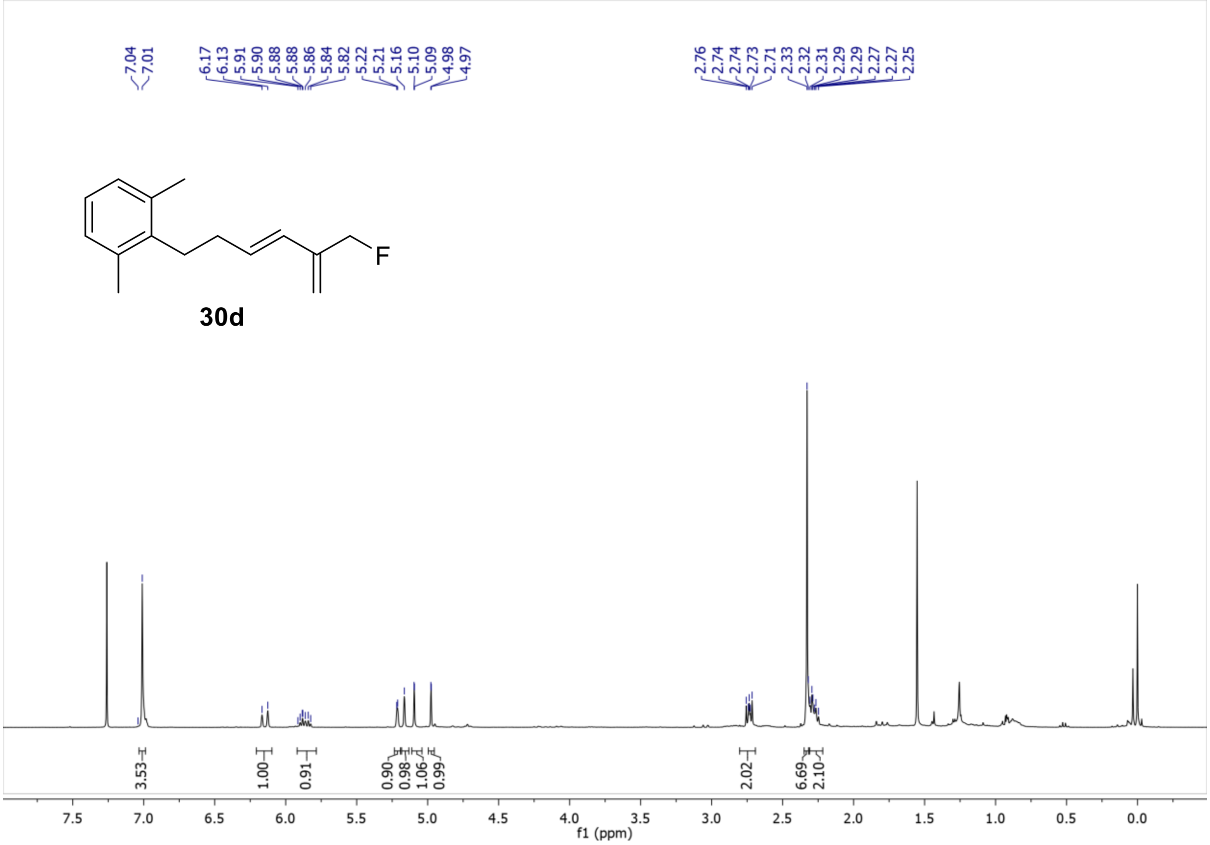

$^{13}C\{^{1}H\}$ NMR (150 MHz, $CDCl_3$): **30d**

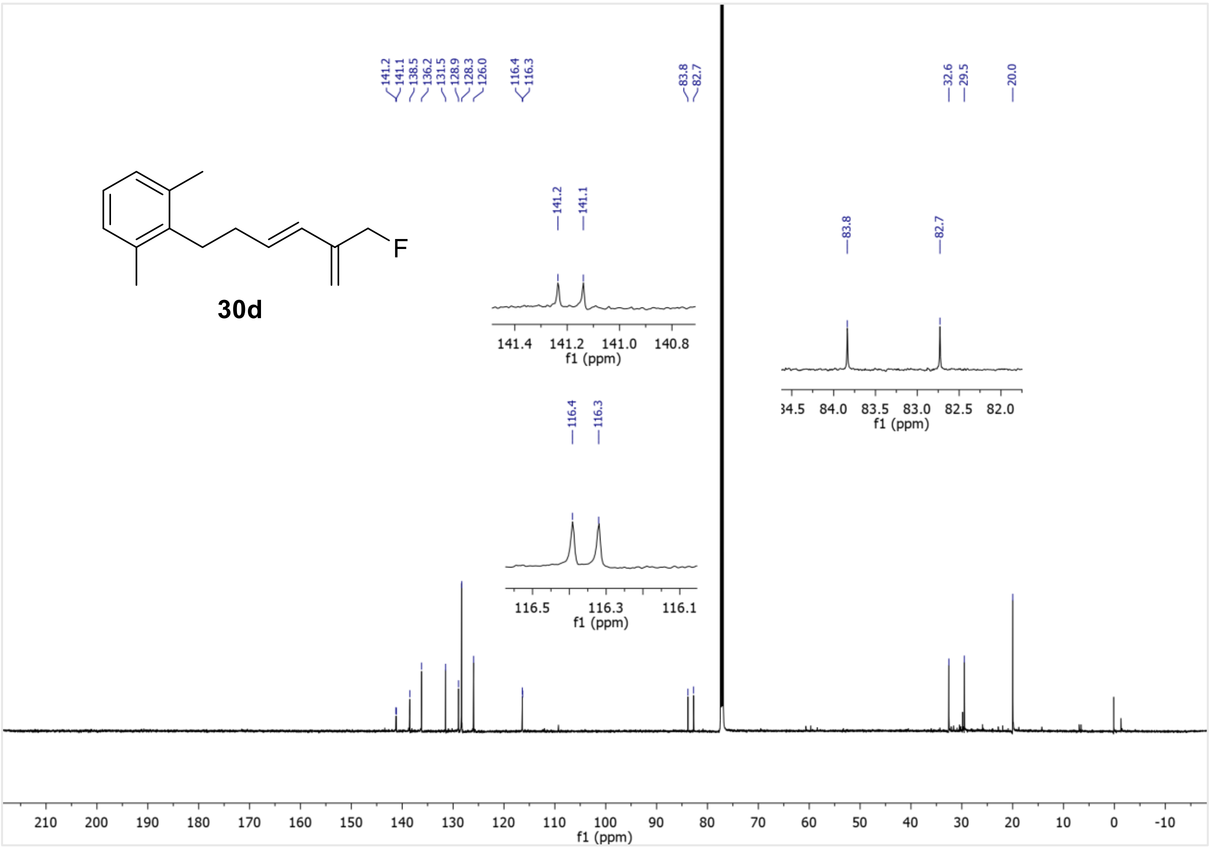

^{19}F NMR (376 MHz, $CDCl_3$): **30d**

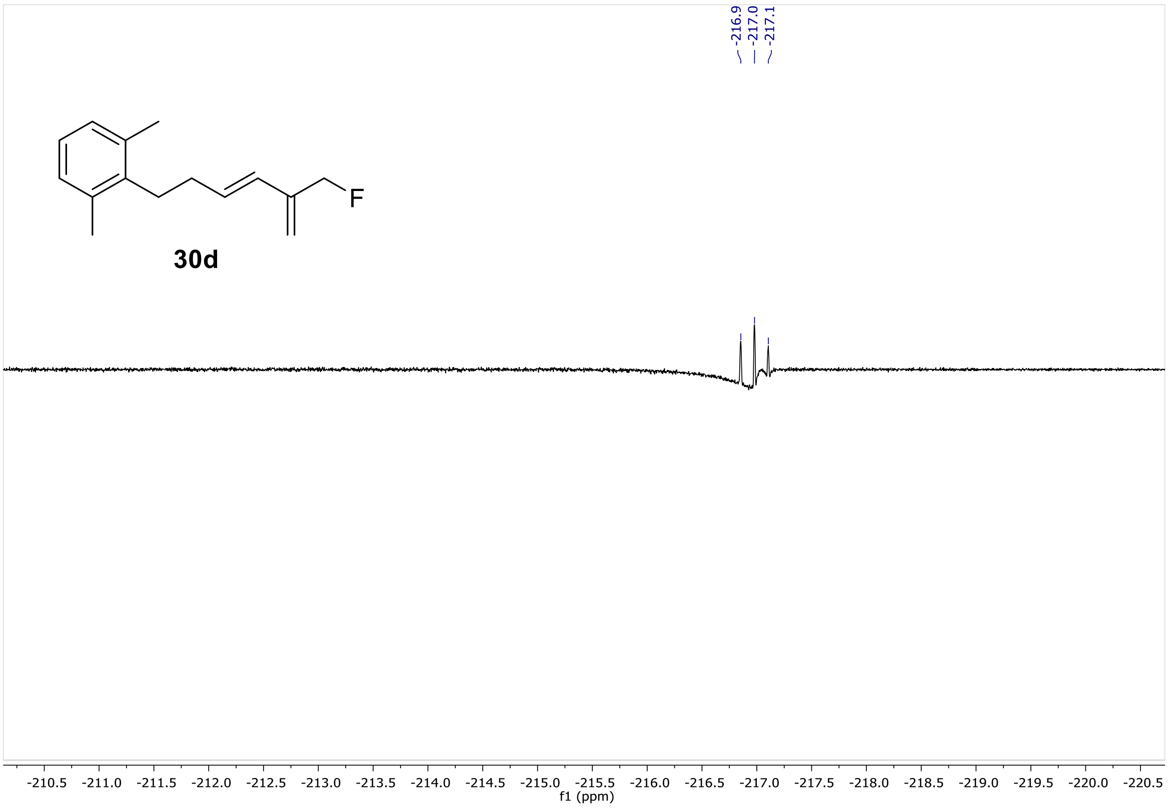

^{1}H NMR (600 MHz, $CDCl_3$): **30e**

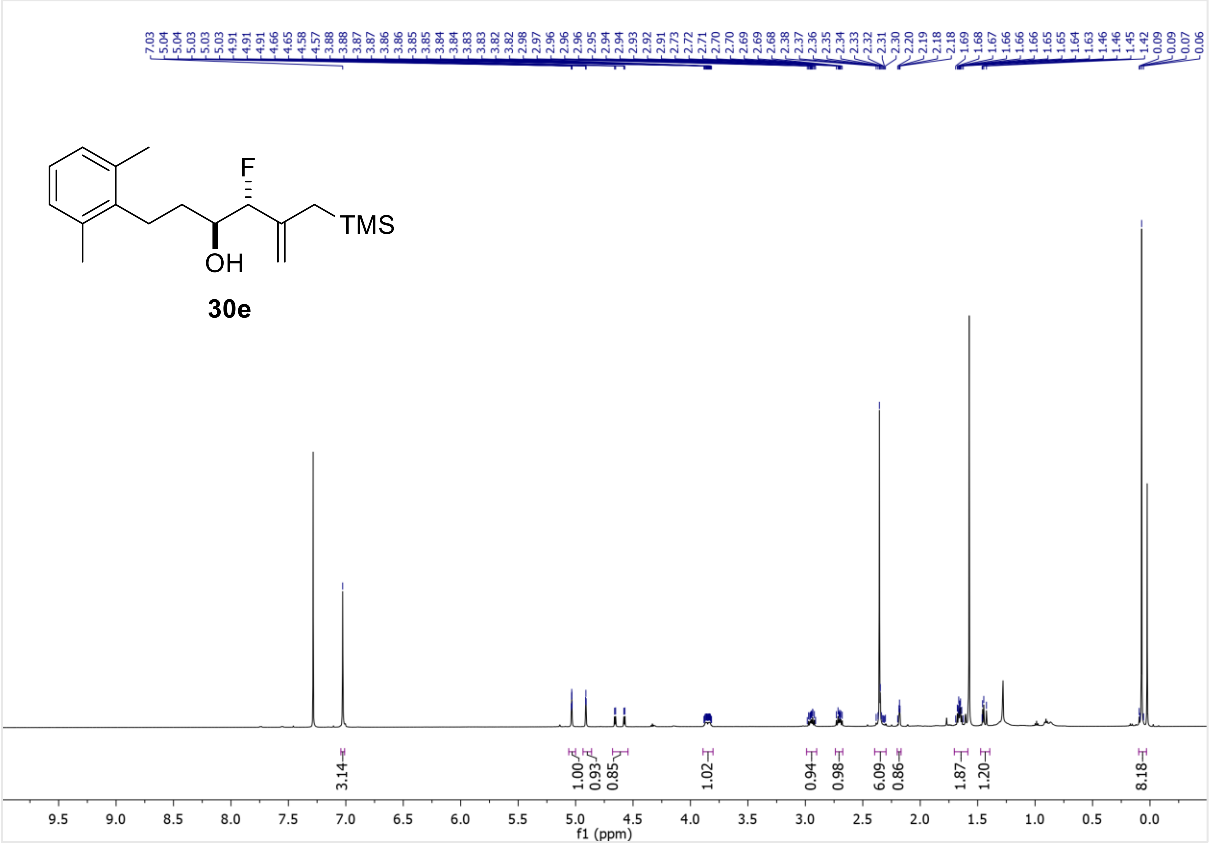

$^{13}C\{^{1}H\}$ NMR (150 MHz, $CDCl_3$): **30e**

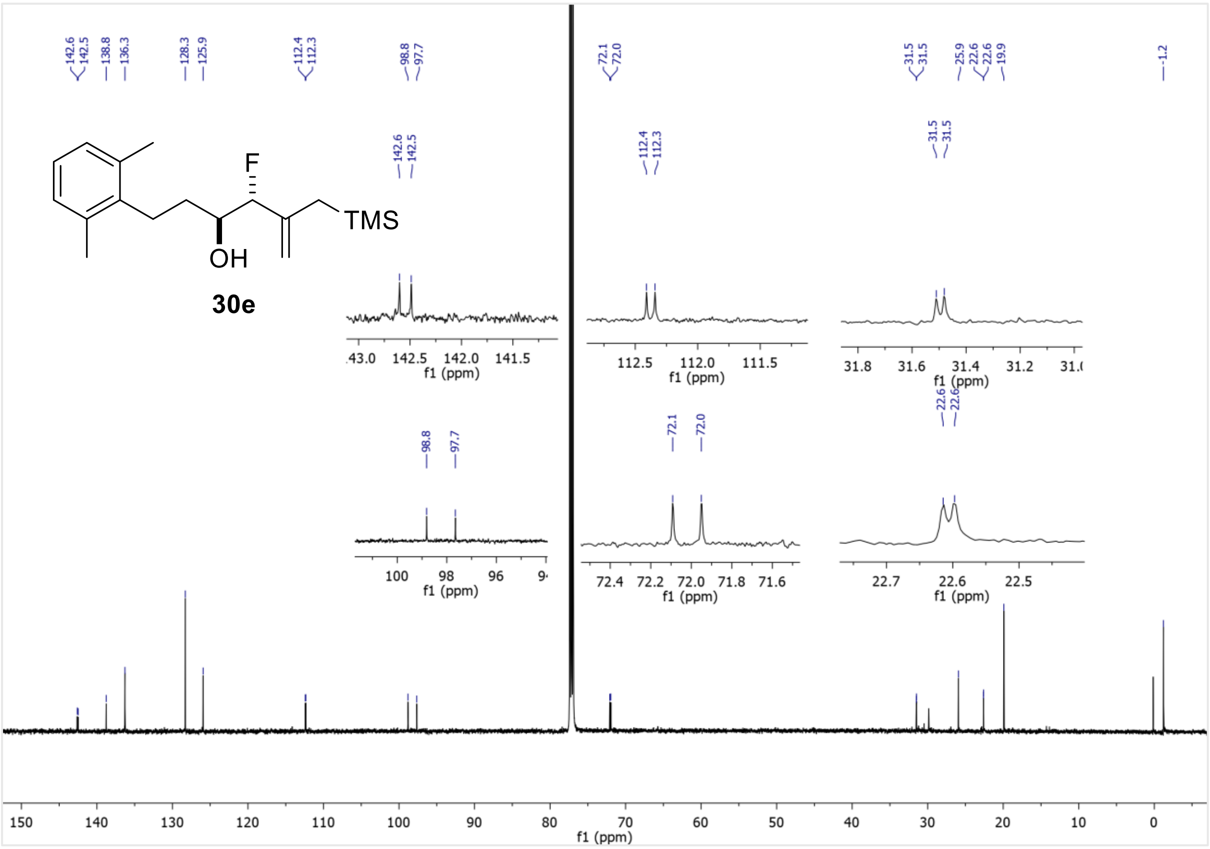

^{19}F NMR (470 MHz, $CDCl_3$): **30e**

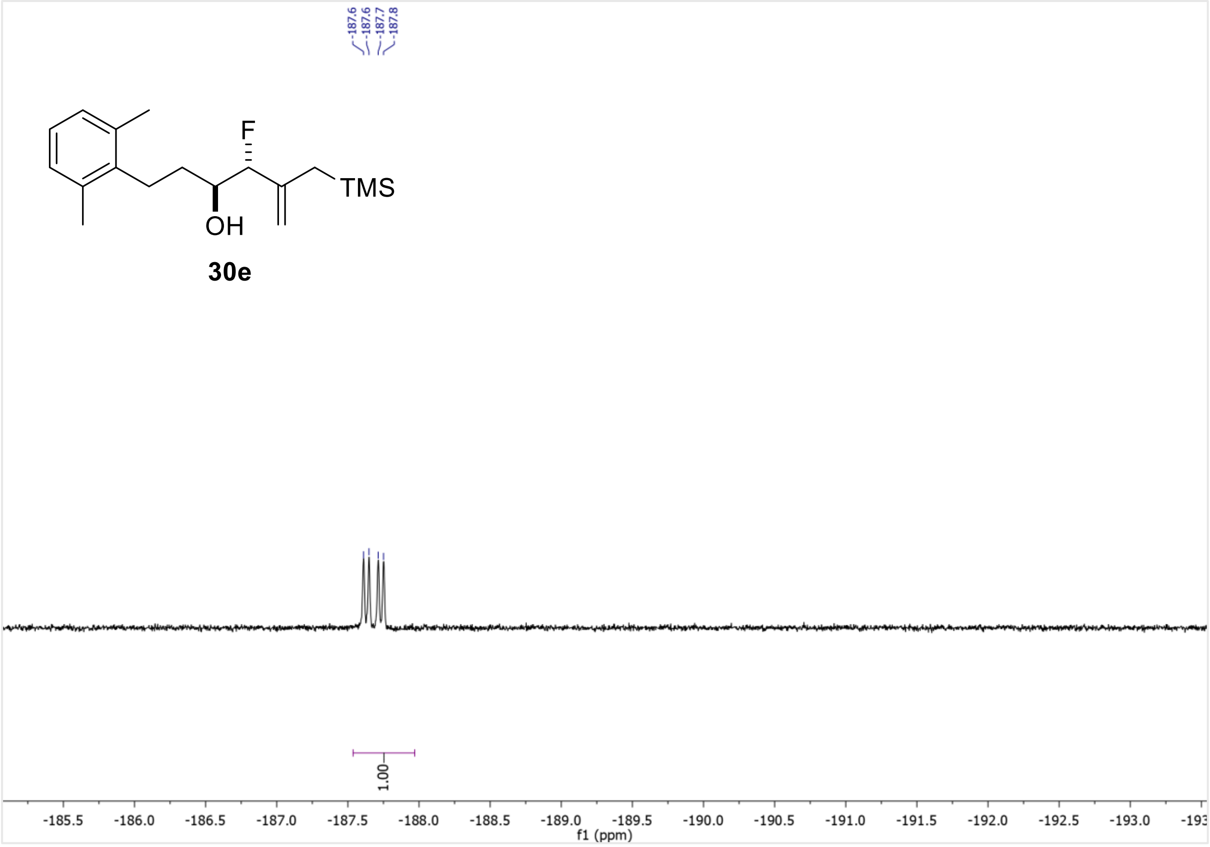

^{1}H NMR (600 MHz, $CDCl_3$): **26p**

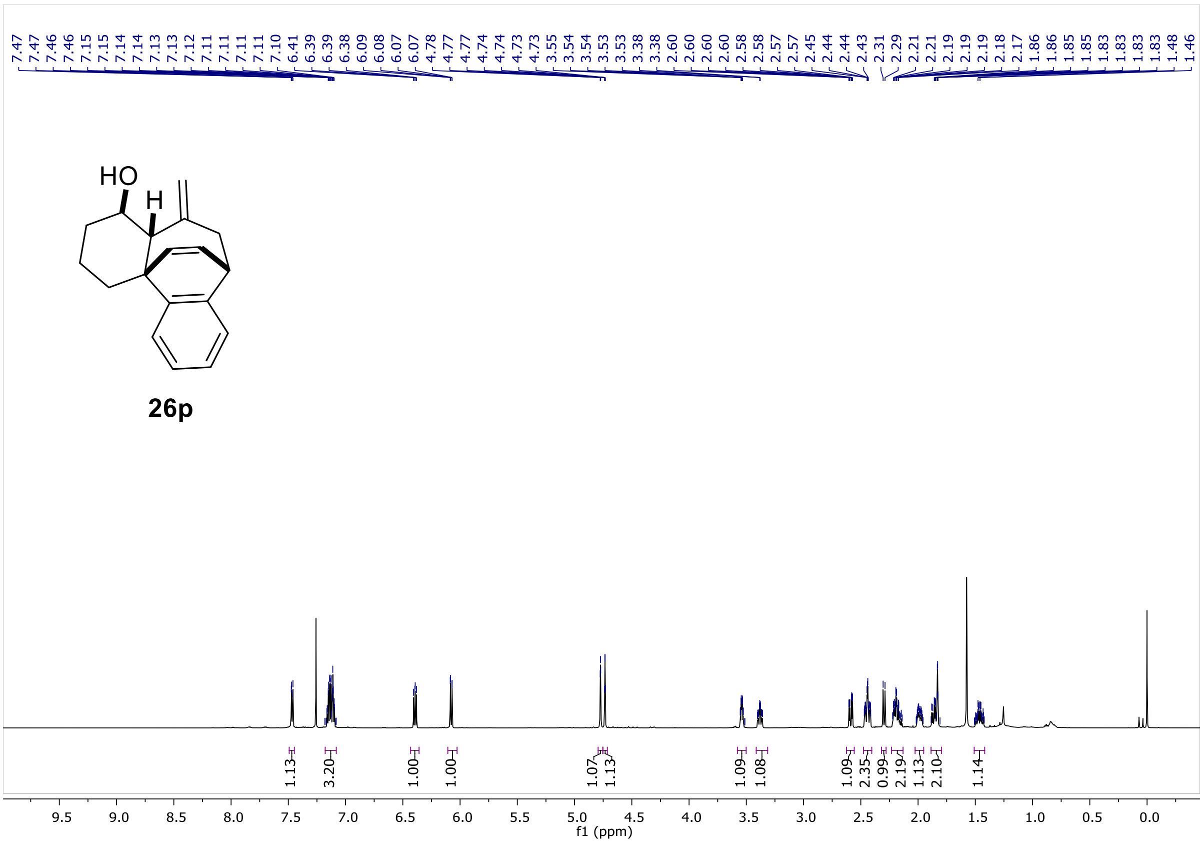

$^{13}C\{^{1}H\}$ NMR (150 MHz, $CDCl_3$): **26p**

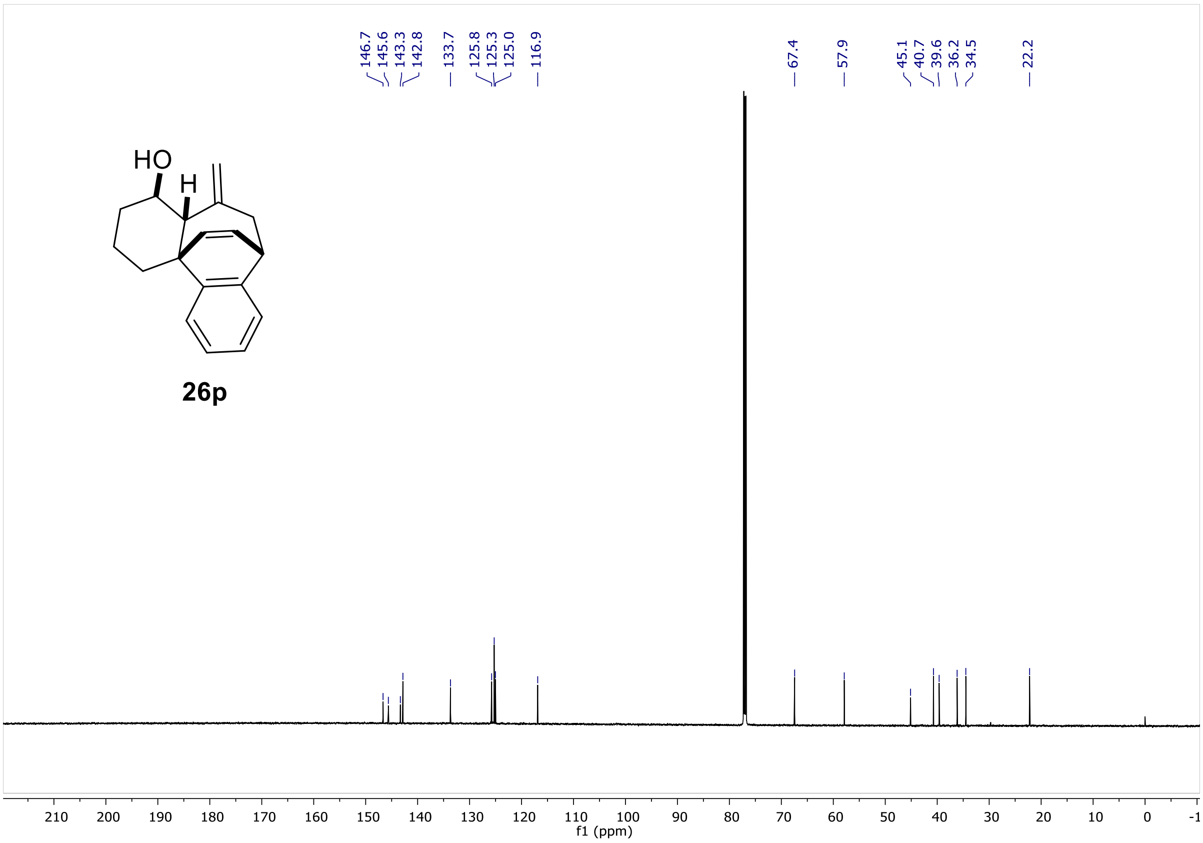

^{1}H NMR (600 MHz, $CDCl_3$): **26q**

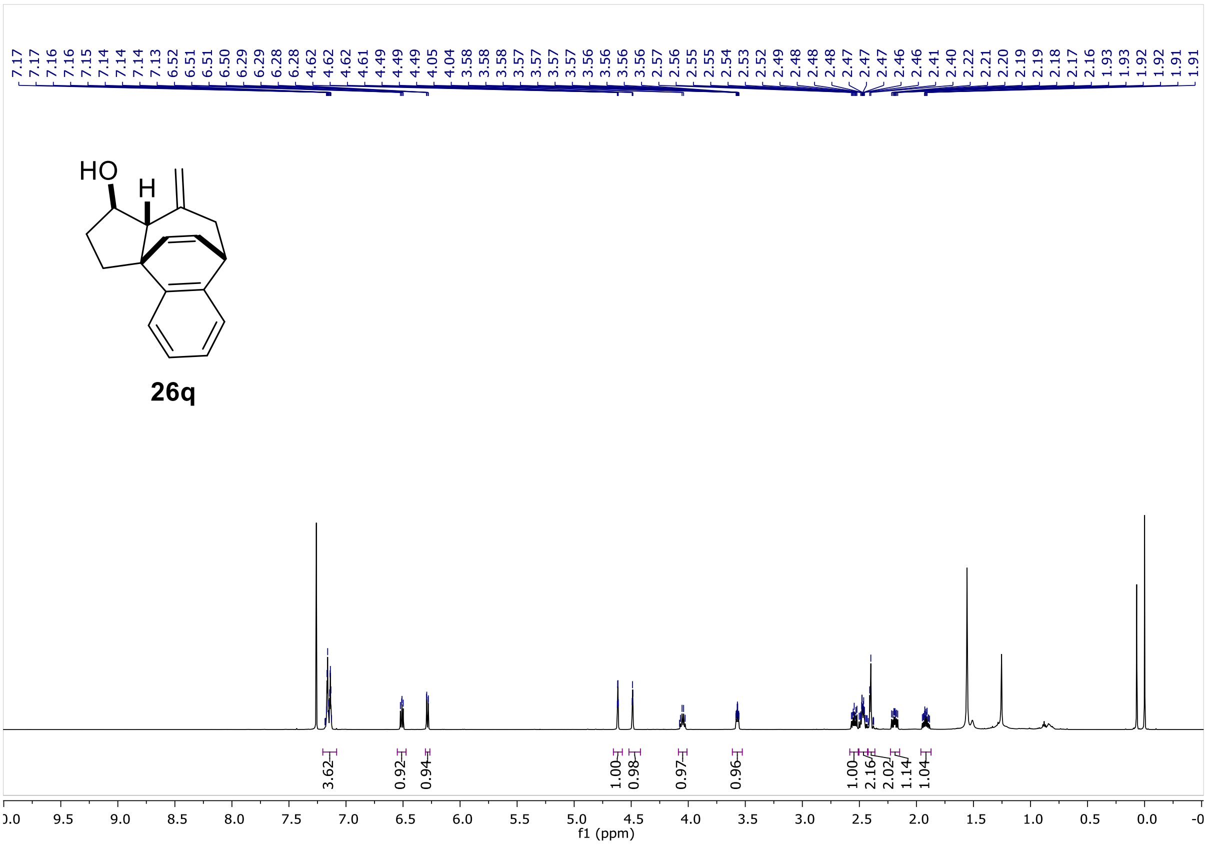

^{13}C{^{1}H} NMR (150 MHz, $CDCl_3$): **26q**

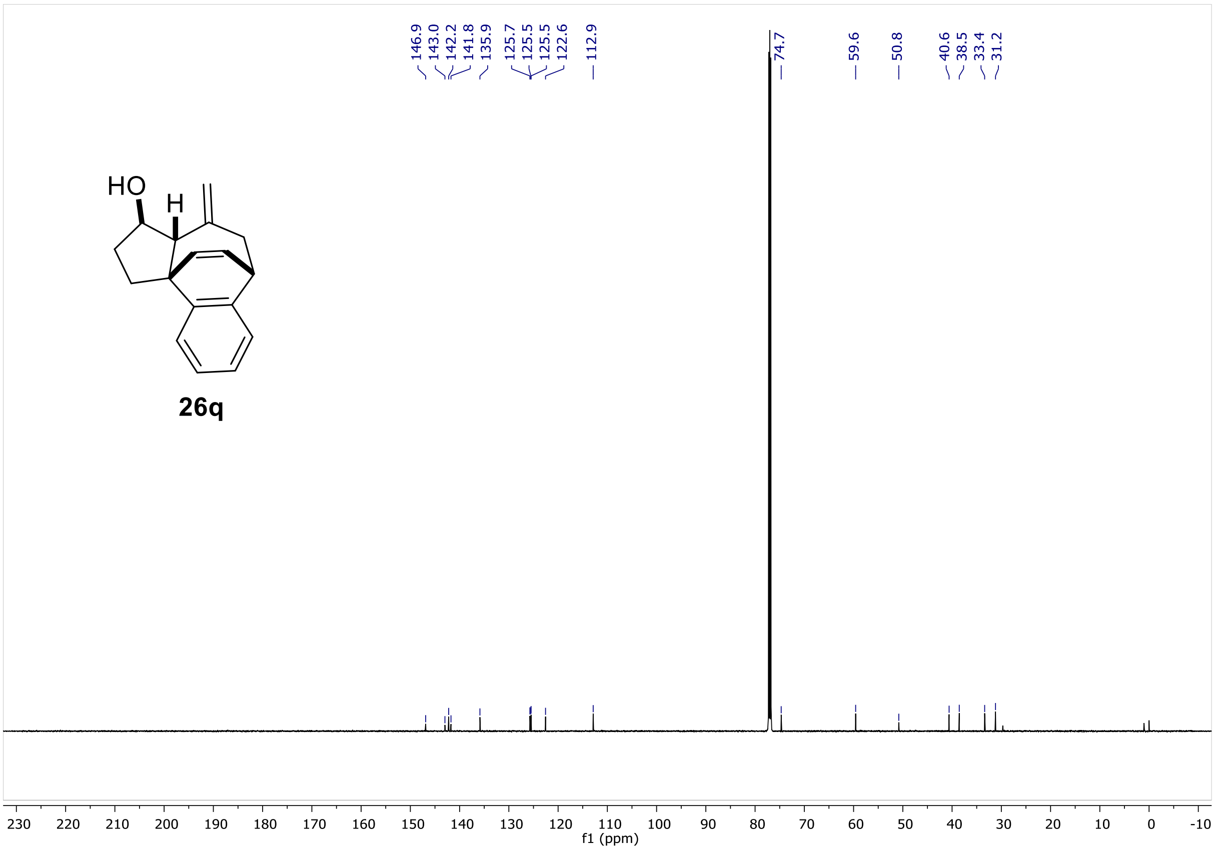

^{1}H NMR (500 MHz, $CDCl_3$): **27q**

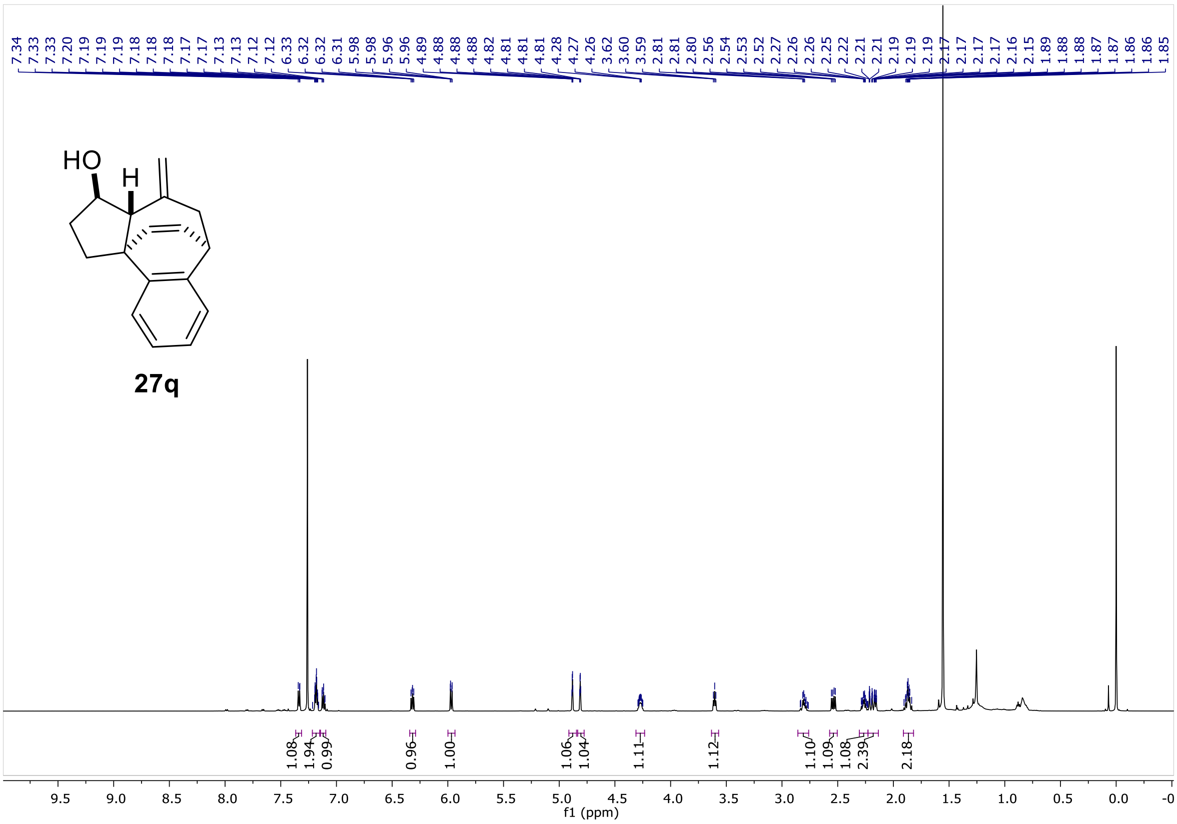

^{13}C{^{1}H} NMR (125 MHz, $CDCl_3$): **27q**

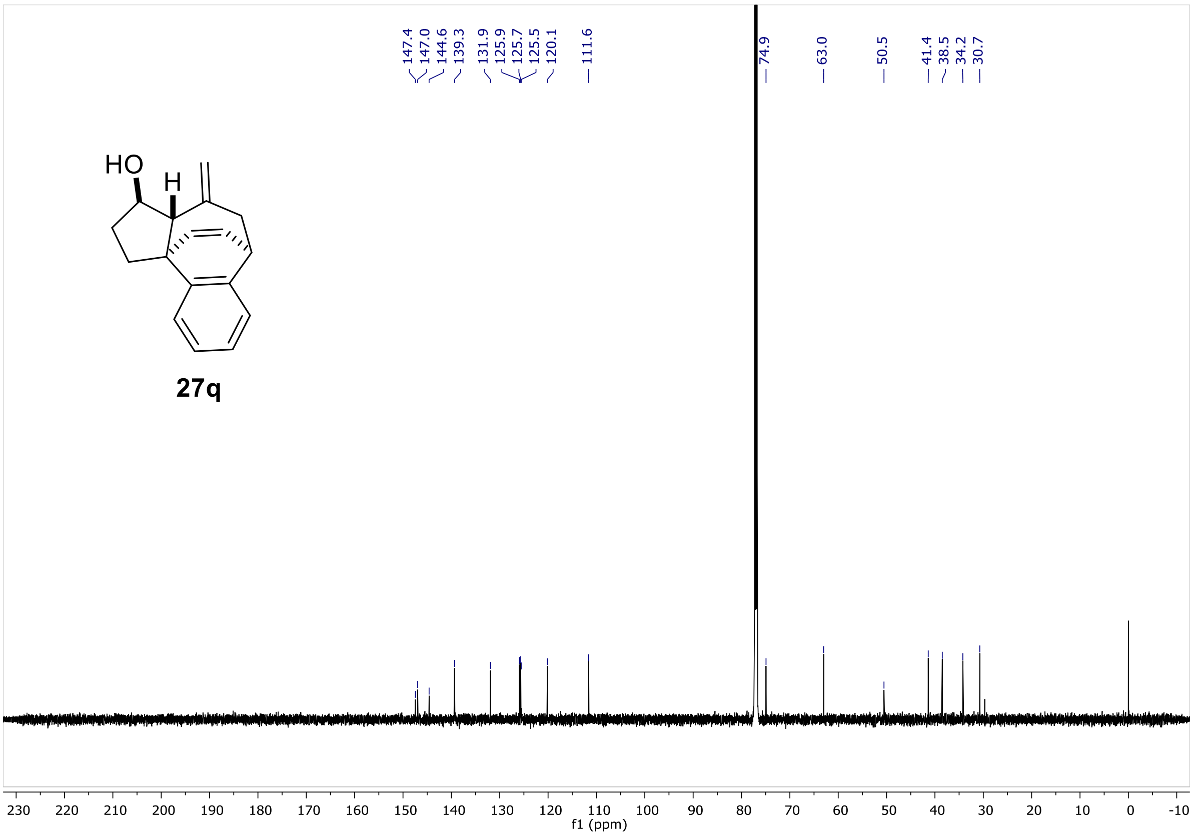

^{1}H NMR (600 MHz, $CDCl_3$): **26r**

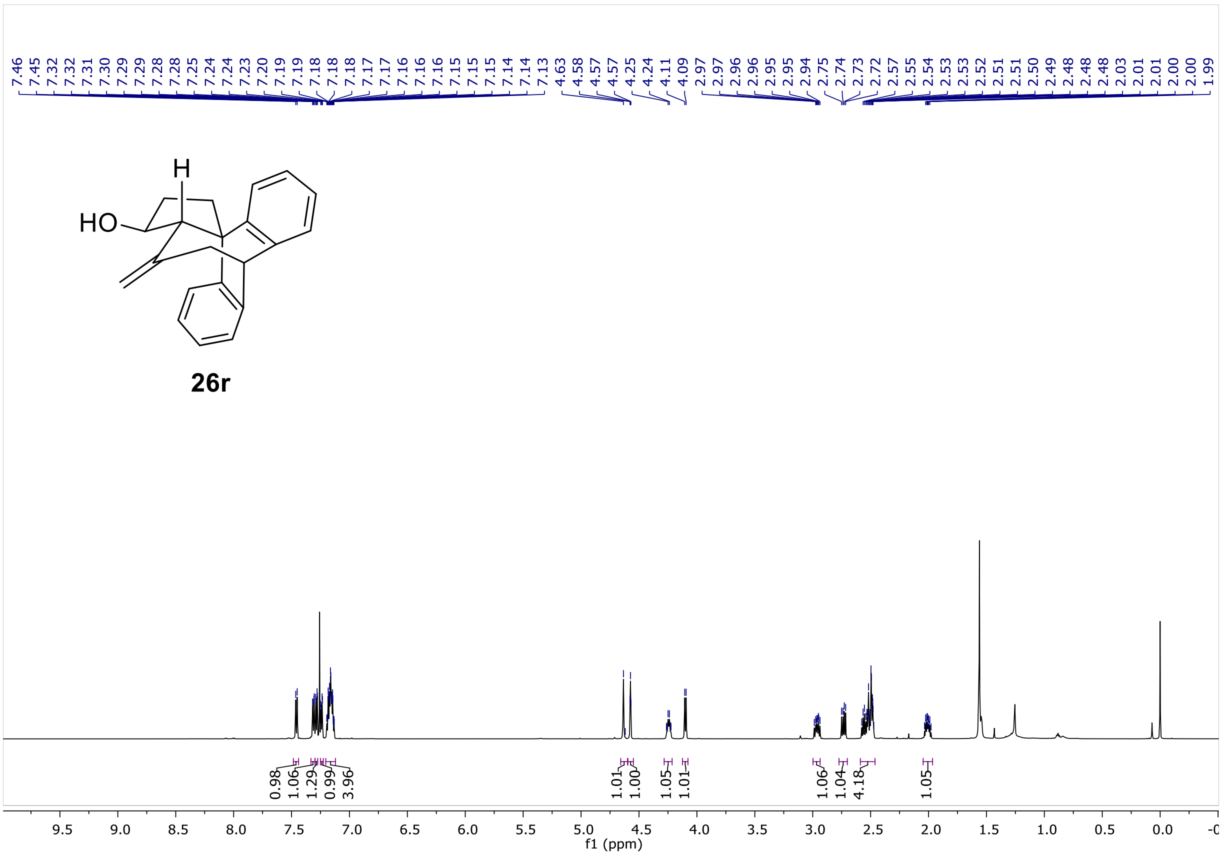

$^{13}C\{^{1}H\}$ NMR (150 MHz, $CDCl_3$): **26r**

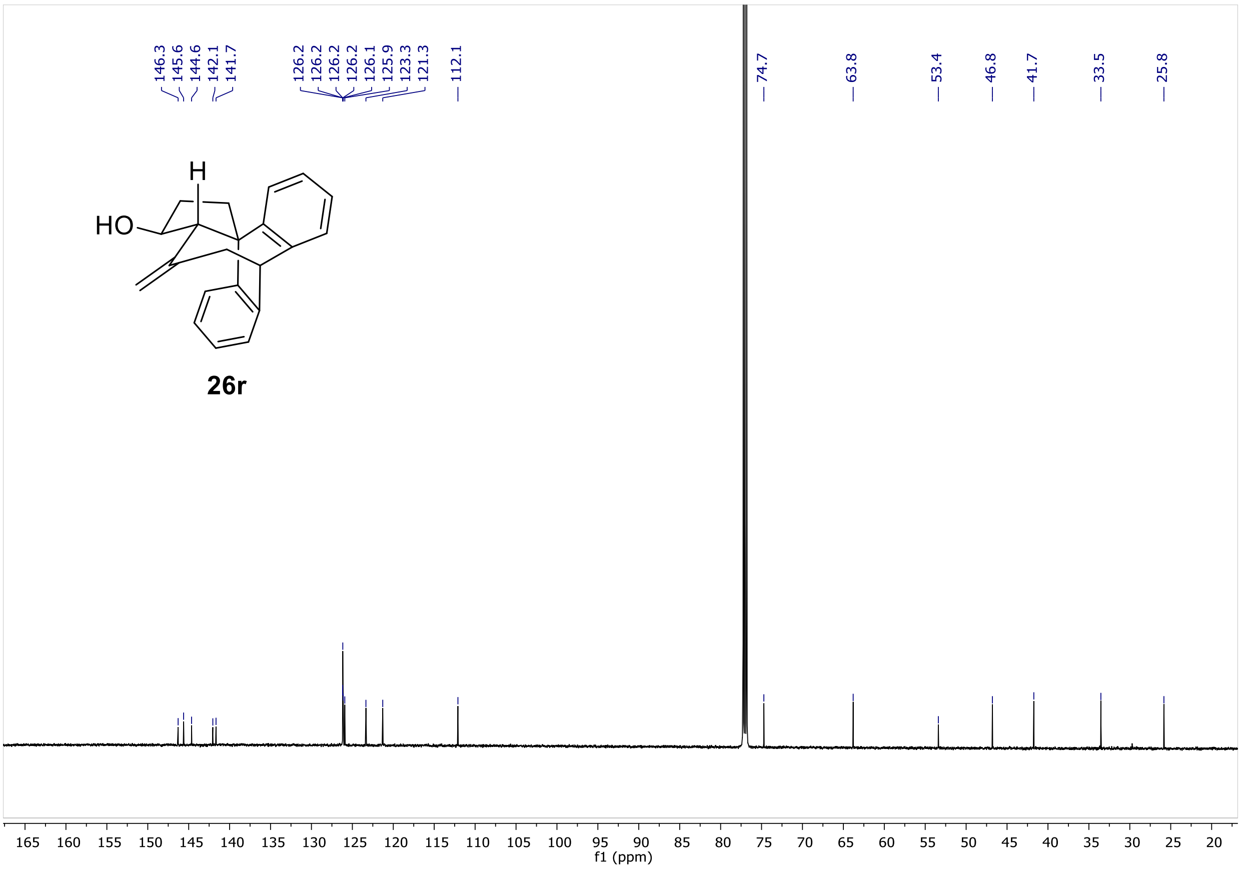

^{1}H NMR (500 MHz, $CDCl_3$): **48**

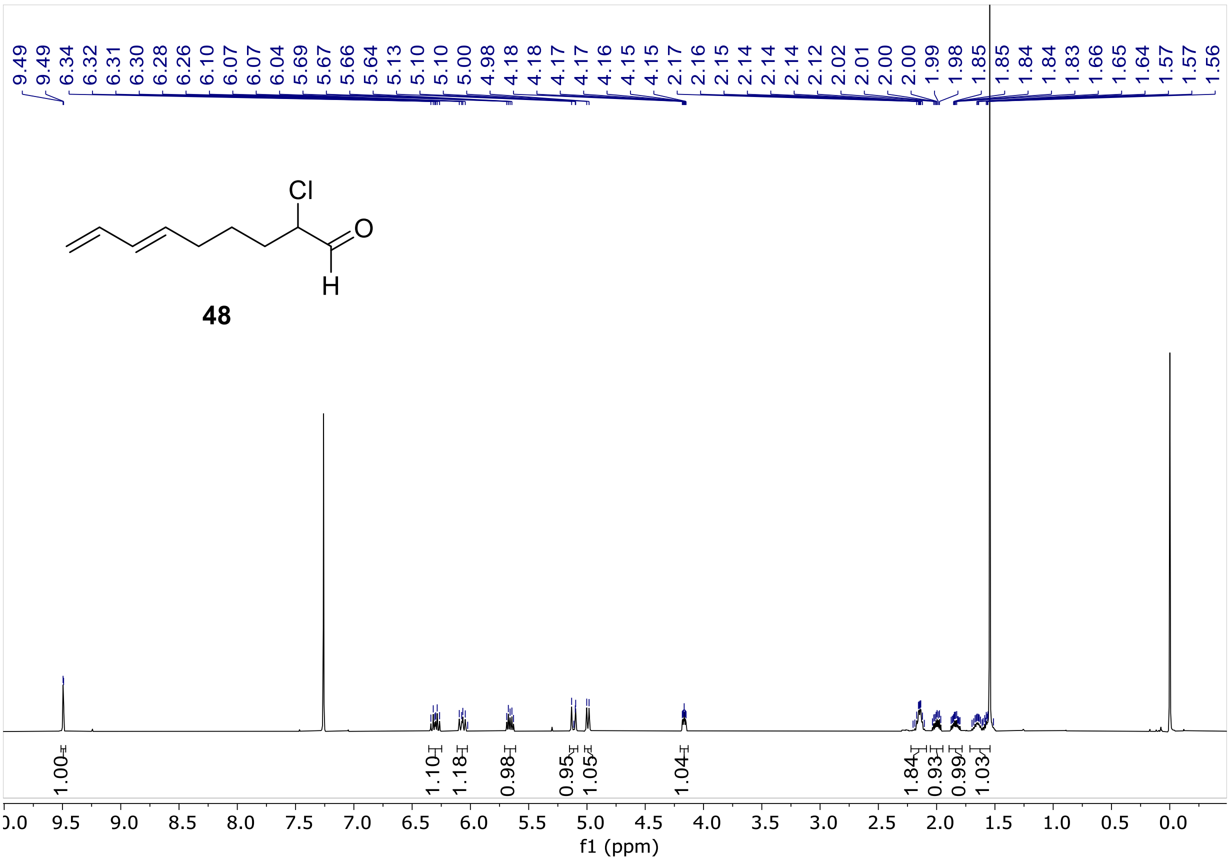

$^{13}C\{^{1}H\}$ NMR (100 MHz, $CDCl_3$): **48**

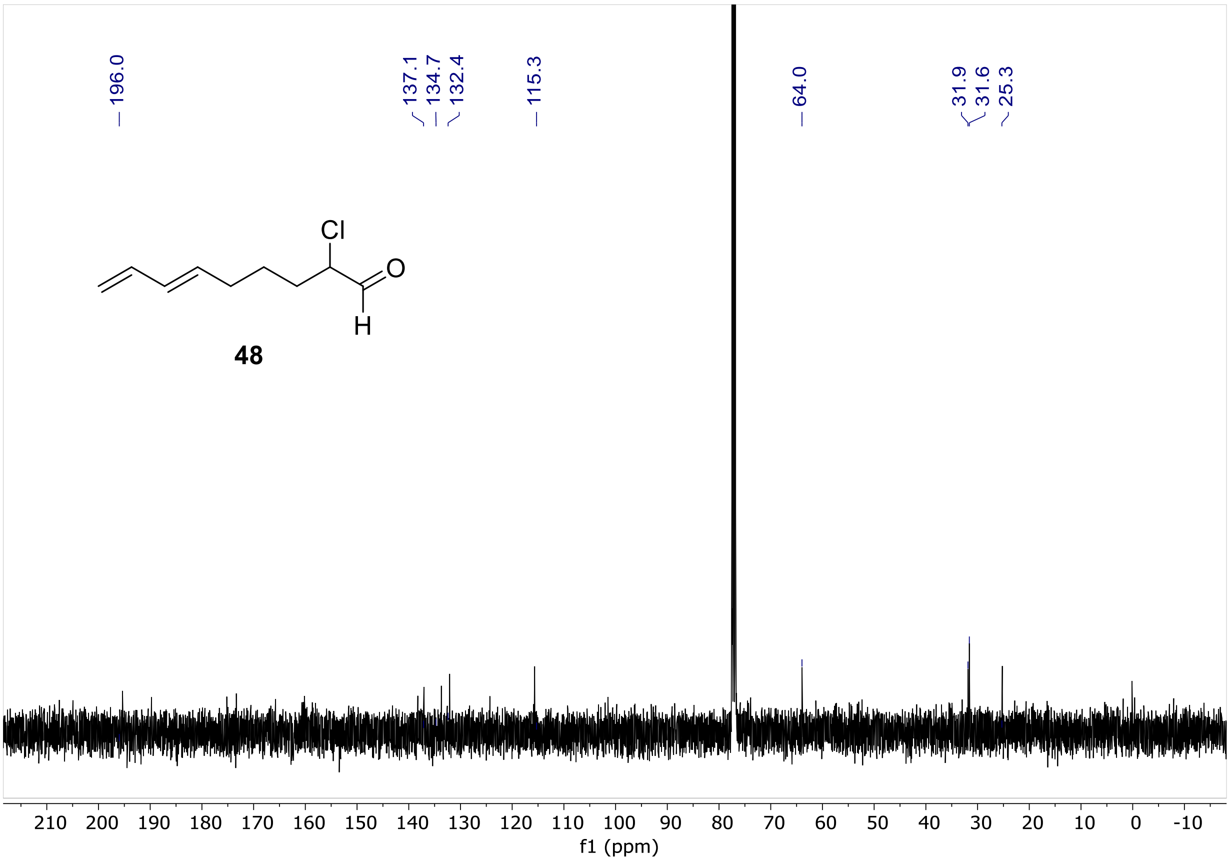

^{1}H NMR (400 MHz, $CDCl_3$): **25l**

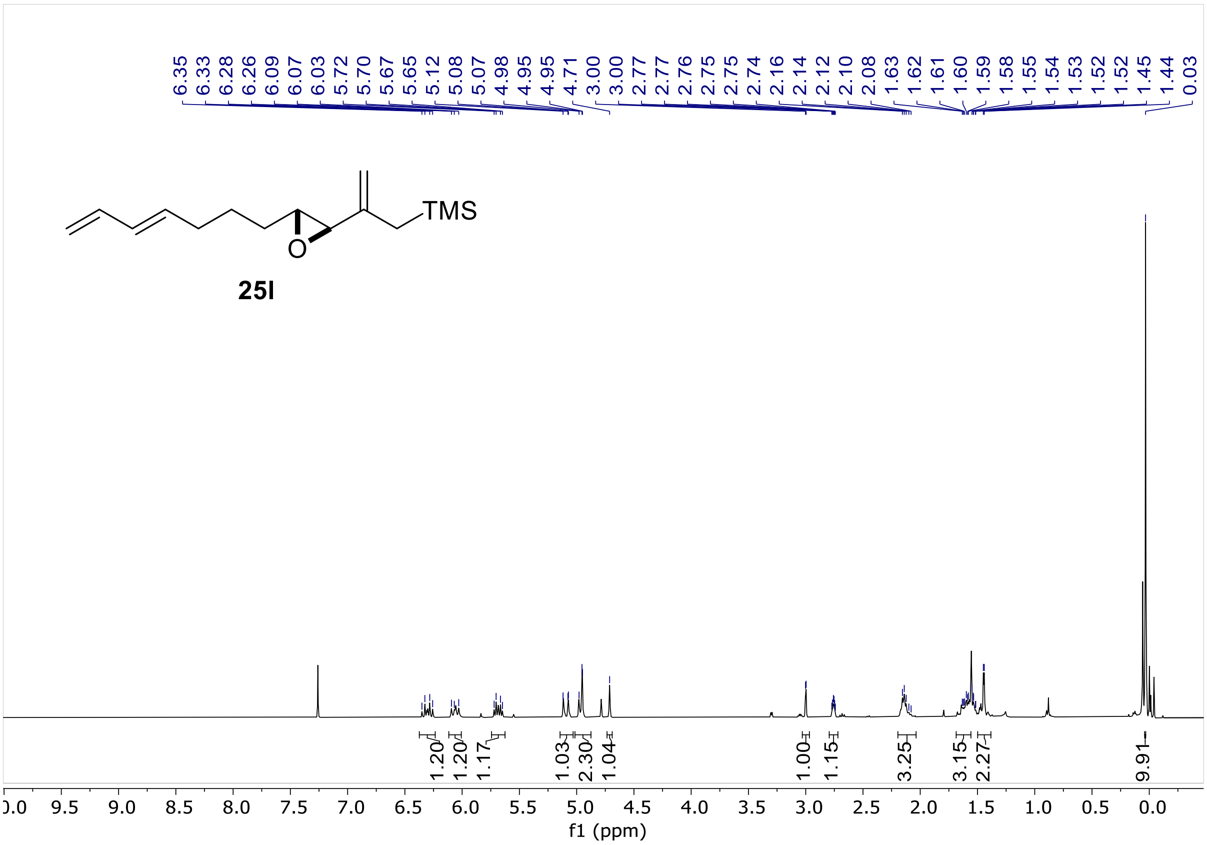

^{13}C{^{1}H} NMR (150 MHz, $CDCl_3$): **25l**

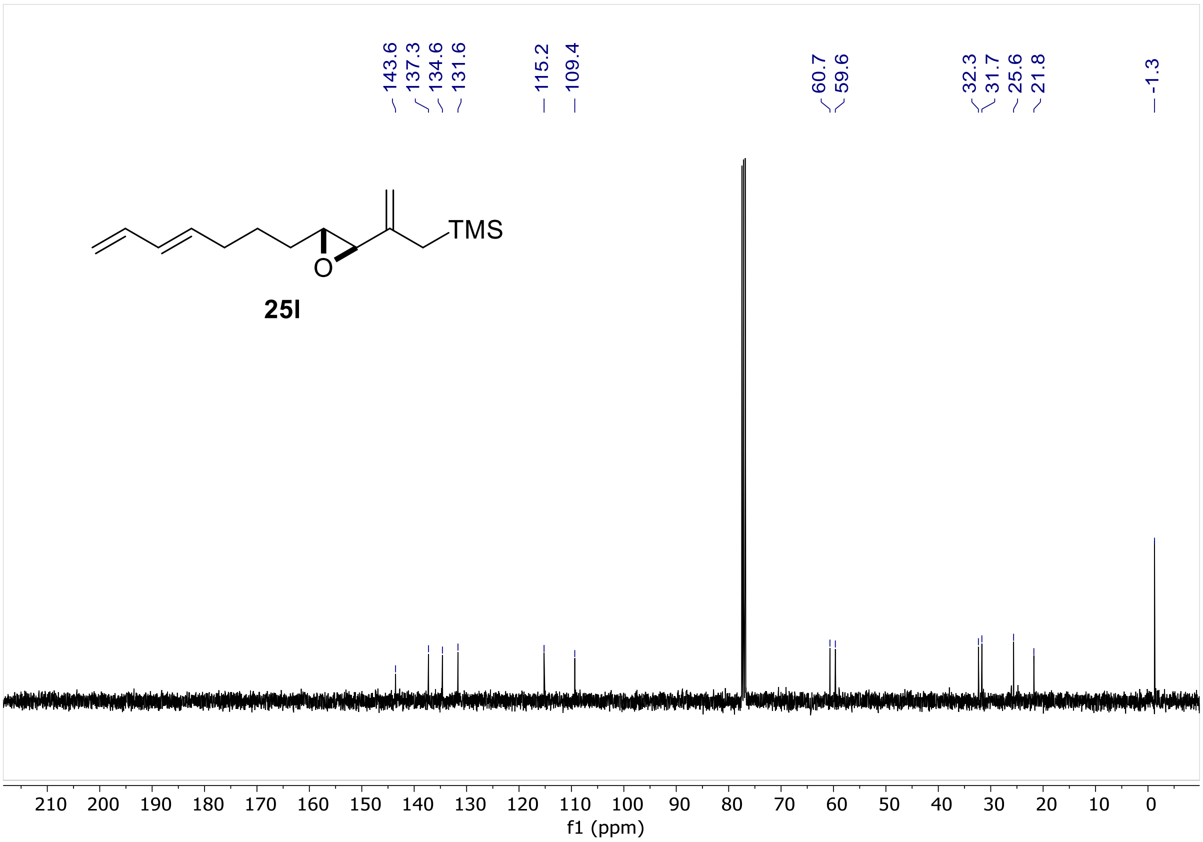

^{1}H NMR (600 MHz, $CDCl_3$): **27l**

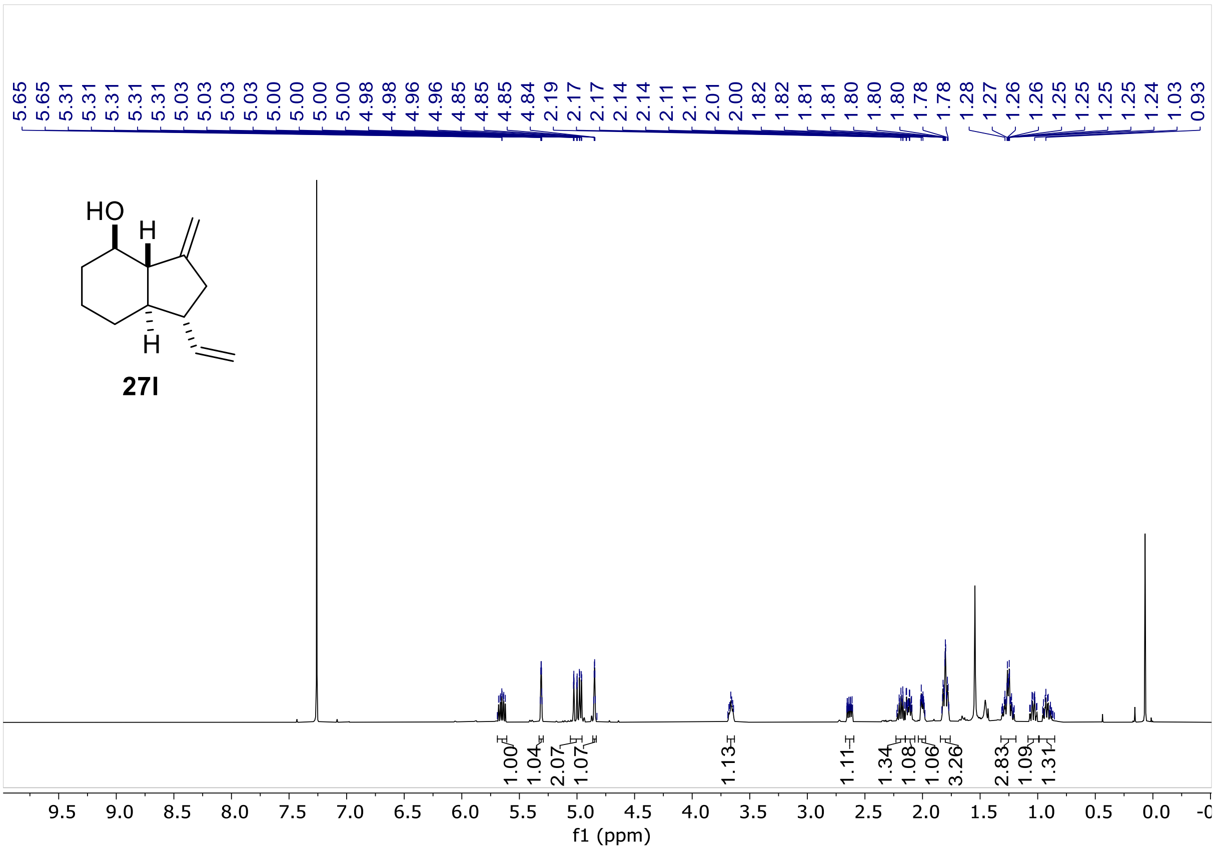

$^{13}C\{^1H\}$ NMR (125 MHz, $CDCl_3$): **27l**

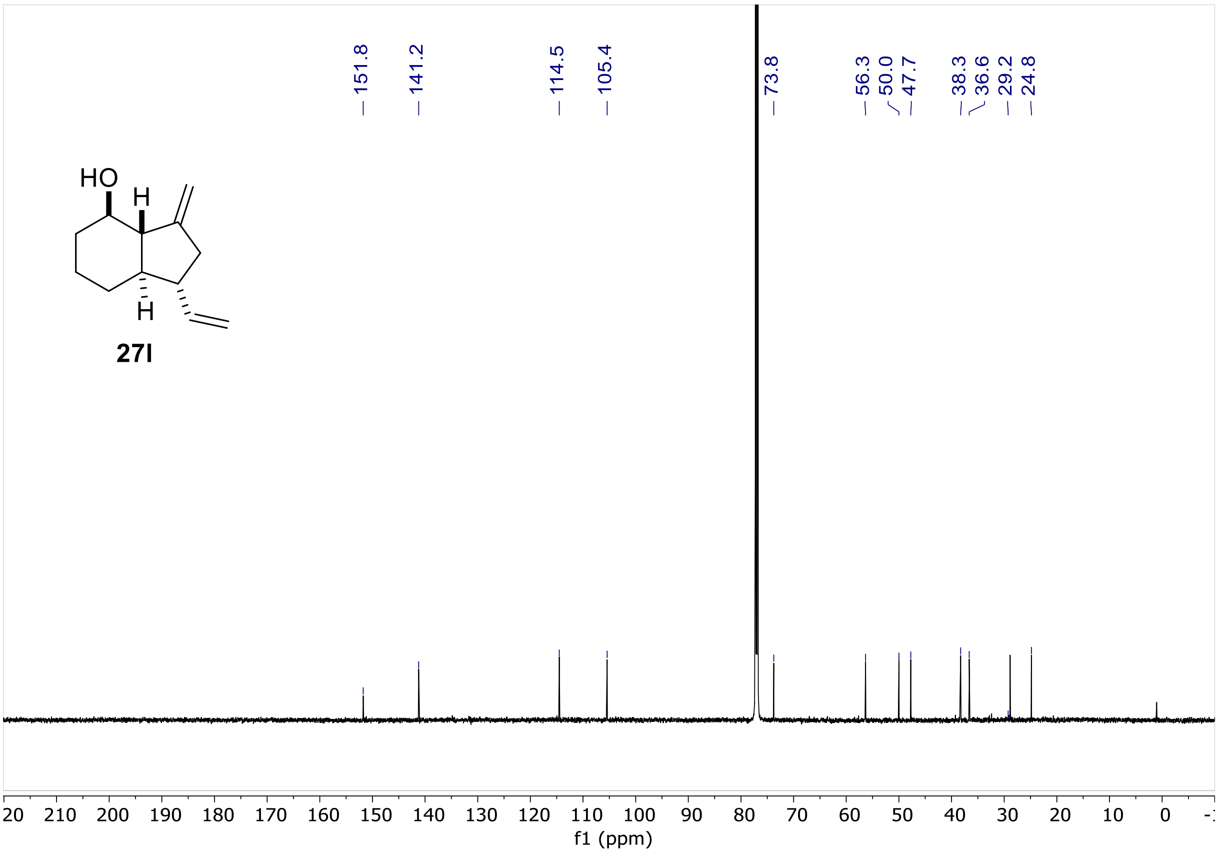

16. HPLC Chromatograms of (+)-23a, (+)-26a, and (–)-27a

Sample	**(+)-23a**
Instrument	Agilent 1260 Infinity II
Column	Daicel Chiralcel® OJ-3
Mobile Phase	Hexane/ iso-Propanol = 90/10 (v/v)
Flowrate	1mL/ min
Detection	UV 239nm

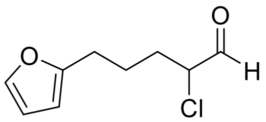

(±)-23a

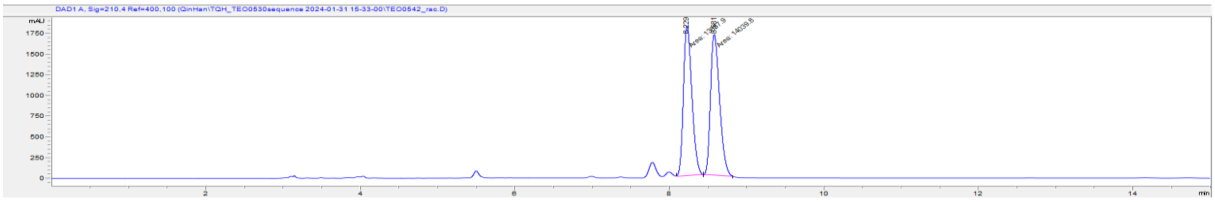

#	Time	Type	Area	Height	Width	Area%	Symmetry
1	8.229	MM	13887.9	1817.6	0.1273	49.728	0.715
2	8.581	MM	14039.8	1708.7	0.1369	50.272	0.664

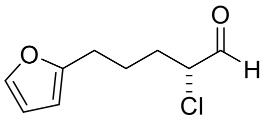

(+)-23a, 78 %*ee*

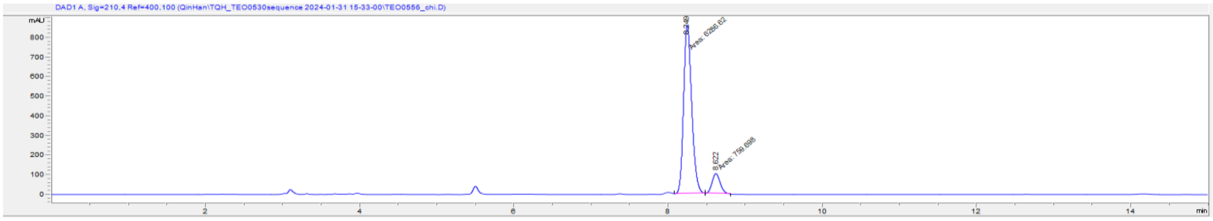

#	Time	Type	Area	Height	Width	Area%	Symmetry
1	8.249	MM	6286.6	858.9	0.122	89.219	0.777
2	8.622	MM	759.7	102.6	0.1234	10.781	0.858

Sample	**(+)-26a**
Instrument	Agilent 1260 Infinity II
Column	Daicel Chiralcel® OJ-3
Mobile Phase	Hexane/ iso-Propanol = 95/5 (v/v)
Flowrate	1mL/ min
Detection	UV 239nm

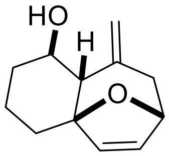

(±)-26a

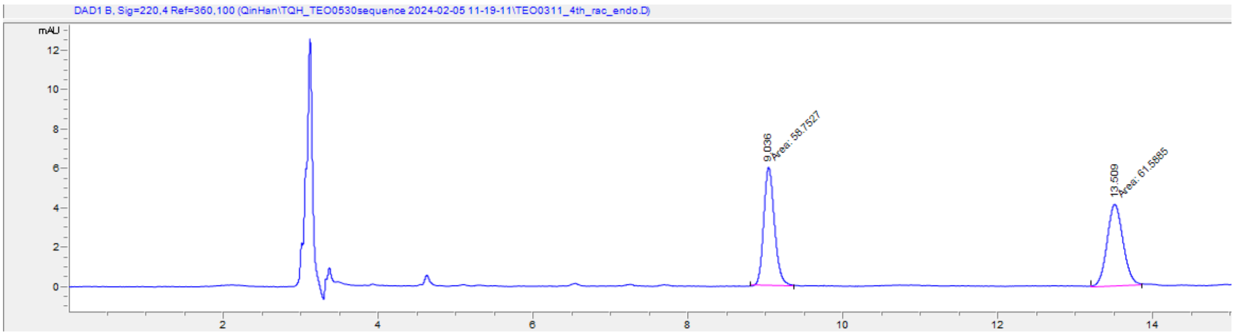

#	Time	Type	Area	Height	Width	Area%	Symmetry
1	9.036	MM	58.8	6	0.1622	48.822	0.807
2	13.509	MM	61.6	4.2	0.244	51.178	0.915

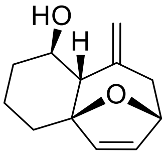

(+)-26a, 81 %*ee*

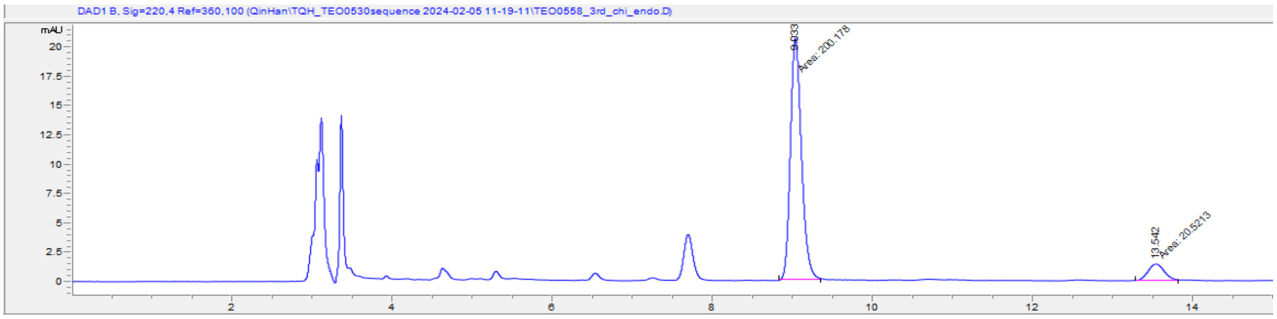

#	Time	Type	Area	Height	Width	Area%	Symmetry
1	9.033	MM	200.2	20.7	0.161	90.702	0.795
2	13.542	MM	20.5	1.4	0.2371	9.298	0.956

Sample	**(–)-27a**
Instrument	Agilent 1260 Infinity II
Column	Daicel Chiralcel® OJ-3
Mobile Phase	Hexane/ iso-Propanol = 95/5 (v/v)
Flowrate	1mL/ min
Detection	UV 239nm

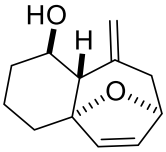

(±)-27a

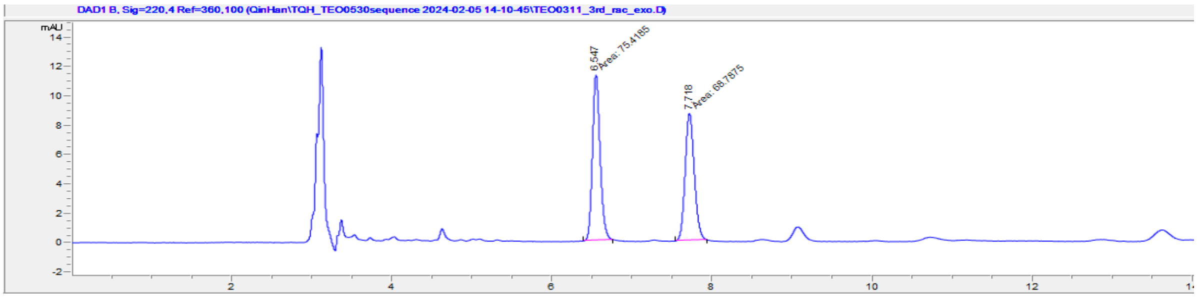

#	Time	Type	Area	Height	Width	Area%	Symmetry
1	6.547	MM	75.4	11.3	0.1115	52.299	0.799
2	7.718	MM	68.8	8.7	0.1325	47.701	0.866

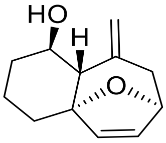

(–)-27a, 79 %*ee*

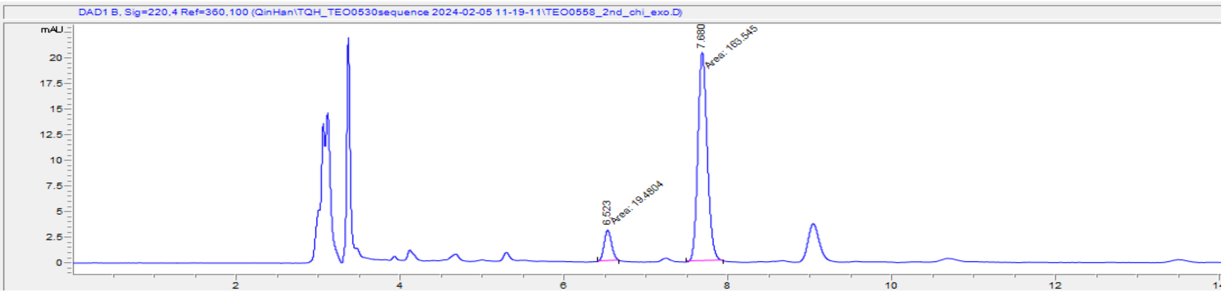

#	Time	Type	Area	Height	Width	Area%	Symmetry
1	6.523	MM	19.5	3.1	0.1063	10.644	0.809
2	7.68	MM	163.5	20.6	0.1326	89.356	0.83

17. Computations: Molecular Coordinates Summary

2,5-dimethylfuran

Charge = 0 Multiplicity = 1

C	-0.718874	1.300066	0.000231
C	0.718825	1.300085	-0.000385
C	1.111338	-0.004551	0.000263
O	0.000012	-0.814915	-0.000191
H	-1.374642	2.159005	0.000616
H	1.374549	2.159055	-0.000702
C	-1.111323	-0.004572	0.000211
C	2.434052	-0.683041	0.000085
H	2.560432	-1.319649	0.883167
H	2.560661	-1.318689	-0.883666
H	3.230230	0.064233	0.000635
C	-2.434030	-0.683064	-0.000083
H	-2.560876	-1.318188	-0.884181
H	-2.560157	-1.320189	0.882655
H	-3.230219	0.064200	0.001069

0 imaginary frequencies

E_{B3LYP}= -308.701411

G_{B3LYP}= -308.606458

E_{M06-2X}= -308.657739

G_{tot}= -308.559767

Furan

Charge = 0 Multiplicity = 1

C	-0.718684	0.959435	-0.000172
C	0.718397	0.959669	0.000096
C	1.098546	-0.347117	-0.000168
O	0.000159	-1.161461	0.000053
H	-1.373752	1.818290	0.000069
H	1.373191	1.818770	0.000437

C	-1.098421	-0.347399	0.000039
H	-2.054441	-0.846718	0.000225
H	2.054703	-0.846181	0.000080

0 imaginary frequencies

E_{B3LYP}= -230.041128

G_{B3LYP}= -229.996642

E_{M06-2X}= -230.024348

G_{tot}= -229.976843

Enolsilane B

Charge = 0 Multiplicity = 1

C	0.638524	0.262210	-1.609323
O	1.154892	1.090220	-0.489448
Si	0.396371	2.584144	0.202817
C	-1.442136	2.326077	0.239194
C	0.634809	-0.368732	-0.291905
C	1.686898	-1.295217	0.153167
C	1.433487	-2.121445	1.176828
O	2.815478	-1.272075	-0.601613
Si	4.389682	-0.902981	-0.040979
C	5.040983	-2.318680	1.002032
C	5.367147	-0.700572	-1.621733
C	4.291382	0.683111	0.952048
C	0.989067	3.876454	-1.000074
C	1.184292	2.646933	1.887968
O	-2.368474	-0.490891	-1.407281
S	-2.896602	-1.192434	-0.210320
C	-4.498604	-0.318116	0.132711
F	-5.093591	-0.832706	1.220011
O	-3.299237	-2.597006	-0.426831
O	-2.111226	-0.950969	1.034011
F	-5.336183	-0.445824	-0.908348
F	-4.295974	0.992998	0.347418
H	5.353470	-1.620859	-2.214045
H	4.957391	0.107498	-2.235734
H	3.596066	0.577292	1.790337

H	5.273621	0.942252	1.360787
H	3.949206	1.512998	0.326921
H	4.497121	-2.411082	1.947071
H	4.965527	-3.271098	0.467832
H	6.096584	-2.149807	1.242207
H	-0.305062	-0.387552	0.252914
H	1.438179	0.035564	-2.303909
H	0.647095	4.864724	-0.674766
H	0.591673	3.697033	-2.003389
H	2.081289	3.894182	-1.053183
H	0.779670	3.497589	2.446686
H	2.268521	2.763710	1.822020
H	0.964329	1.737805	2.455764
H	-1.725331	1.526163	0.926920
H	-1.857928	2.079311	-0.740160
H	-1.910215	3.255901	0.581865
H	0.481956	-2.061873	1.690950
H	2.145761	-2.874801	1.489351
H	6.411662	-0.459637	-1.398104
H	-0.314530	0.590761	-2.006852

0 imaginary frequencies

E_{B3LYP}= -2085.977871

G_{B3LYP}= -2085.702374

E_{M06-2X}= -2085.940791

G_{tot}= -2085.662275

Allylsilane A

Charge = 0 Multiplicity = 1

C	0.538554	0.436207	-1.723536
O	1.167059	1.157666	-0.593720
Si	0.418885	2.610451	0.182436
C	-1.409906	2.307894	0.322791
C	0.606362	-0.346148	-0.492138
C	1.600435	-1.380336	-0.182865
C	1.202203	-2.271141	0.744299
Si	4.443435	-0.921415	0.073708

C	4.696745	-2.126926	1.500868
C	5.912423	-0.979674	-1.107636
C	4.204400	0.818864	0.749625
C	0.897393	3.949022	-1.023196
C	1.271591	2.708349	1.833561
O	-2.416293	-0.460087	-1.401627
S	-2.901930	-1.194103	-0.206124
C	-4.483370	-0.321367	0.225226
F	-5.037543	-0.862862	1.321043
O	-3.321812	-2.589454	-0.448867
O	-2.068073	-0.994648	1.013461
F	-5.363195	-0.415271	-0.784183
F	-4.263396	0.982104	0.467788
H	6.043696	-1.982980	-1.526621
H	5.776043	-0.282276	-1.941111
H	3.390155	0.834139	1.478715
H	5.112206	1.168730	1.252425
H	3.957935	1.528574	-0.045951
H	3.844183	-2.113771	2.187313
H	4.823082	-3.152697	1.138683
H	5.590705	-1.860968	2.075192
H	-0.295516	-0.348394	0.111766
H	1.252802	0.277263	-2.522962
H	0.540624	4.916955	-0.655175
H	0.451221	3.779942	-2.007636
H	1.983693	4.007031	-1.136410
H	0.822530	3.522337	2.413256
H	2.339841	2.909688	1.730364
H	1.140185	1.782582	2.401527
H	-1.635700	1.478576	0.997150
H	-1.889818	2.091314	-0.633922
H	-1.868788	3.215386	0.731778
H	0.247574	-2.167451	1.249239
H	1.805806	-3.140838	0.982391
H	6.839476	-0.707982	-0.591666
H	-0.436472	0.811974	-2.012872
C	2.904232	-1.459905	-0.926606
H	2.882175	-0.865374	-1.844562

H	3.084553	-2.495188	-1.237117

0 imaginary frequencies

E_{B3LYP}= -2050.039386

G_{B3LYP}= -2049.740344

E_{M06-2X}= -2049.975991

G_{tot}= -2049.673930

Enolsilane D

Charge = 0 Multiplicity = 1

C	-4.601119	3.519900	1.506028
C	-3.862878	2.529796	0.926735
C	-4.920186	3.578152	-0.706759
C	-5.290168	4.200771	0.444404
C	-2.926690	1.497736	1.449890
C	-1.474099	1.725381	0.994704
C	-0.545369	0.624395	1.533320
C	0.816984	0.806919	0.942654
O	1.833050	-0.301786	1.076595
Si	1.919981	-1.972352	1.763564
C	0.251286	-2.541593	2.357405
C	1.227164	0.108662	-0.280010
C	2.229802	0.648135	-1.215387
C	2.296652	0.165287	-2.462301
O	2.977509	1.658462	-0.701817
Si	4.686324	1.728323	-0.650443
C	5.350470	2.256363	-2.322528
C	5.020299	3.018077	0.661575
C	5.318602	0.031542	-0.165786
C	3.139067	-1.698216	3.147156
C	2.565570	-2.940759	0.311714
O	-1.689160	-0.295908	-1.494719
S	-1.706788	-1.774010	-1.597536
C	-3.073763	-2.267895	-0.439958
F	-3.291801	-3.588513	-0.487411
O	-2.133138	-2.321068	-2.900135
O	-0.510555	-2.431312	-0.999067

F	-4.212203	-1.631924	-0.750146
F	-2.759197	-1.946636	0.835973
H	4.571607	3.979252	0.391429
H	4.609195	2.708615	1.627578
H	5.000117	-0.728702	-0.885361
H	6.412914	0.024888	-0.132652
H	4.947065	-0.252700	0.823038
H	5.202615	1.484238	-3.083796
H	4.864413	3.174558	-2.667270
H	6.426488	2.450866	-2.252686
H	0.565894	-0.664911	-0.654435
H	1.334834	1.736017	1.160661
H	3.302274	-2.639908	3.682342
H	2.760573	-0.964394	3.865023
H	4.104250	-1.349994	2.769659
H	2.784631	-3.965451	0.630580
H	3.484935	-2.505228	-0.087814
H	1.811575	-2.983054	-0.478811
H	-0.462149	-2.620299	1.535618
H	-0.158379	-1.897199	3.138965
H	0.385393	-3.541853	2.786610
H	-0.485158	0.677801	2.625594
H	-0.947695	-0.348771	1.253853
H	-1.132042	2.710319	1.331418
H	-1.445633	1.712603	-0.097343
H	-2.985657	1.507184	2.543098
H	-3.247377	0.504522	1.116973
H	-4.649826	3.737093	2.563342
H	-5.968040	5.037304	0.533063
H	1.647096	-0.648124	-2.760789
H	2.977715	0.575063	-3.197690
H	6.097316	3.172337	0.784927
O	-4.050449	2.557286	-0.433899
H	-5.167592	3.720832	-1.746802

0 imaginary frequencies

E_{B3LYP}= -2432.806960

G_{B3LYP}= -2432.404843

E_{M06-2X}= -2432.715018

G_{tot}= -2432.309882
Allylsilane C

Charge = 0 Multiplicity = 1

C	-4.799575	3.402165	1.400417
C	-4.006876	2.430959	0.862636
C	-5.121245	3.346732	-0.812088
C	-5.525719	3.996727	0.312015
C	-3.013274	1.477367	1.427213
C	-1.577264	1.763492	0.952884
C	-0.584812	0.748218	1.542544
C	0.758232	0.955660	0.910896
O	1.848952	-0.049289	1.142511
Si	1.998196	-1.659725	1.937311
C	0.334754	-2.374802	2.367616
C	1.173303	0.182487	-0.264065
C	2.066885	0.620230	-1.348161
C	2.037840	-0.154035	-2.447221
Si	4.736703	1.621451	-0.758944
C	5.510953	0.417832	-1.985154
C	5.545259	3.321074	-0.891403
C	4.939564	0.956852	0.993130
C	3.016428	-1.238668	3.440751
C	2.861040	-2.631891	0.601974
O	-1.652786	-0.344273	-1.481430
S	-1.638745	-1.822685	-1.580635
C	-2.968966	-2.339872	-0.390875
F	-3.146119	-3.667311	-0.411340
O	-2.083778	-2.382906	-2.871320
O	-0.414075	-2.454008	-1.010210
F	-4.132278	-1.745915	-0.693300
F	-2.644428	-1.985144	0.873351
H	5.438677	3.733174	-1.900399
H	5.091467	4.029208	-0.189652
H	4.666835	-0.099477	1.043384
H	5.983916	1.046616	1.311033
H	4.317251	1.500691	1.710119

H	5.008807	-0.554363	-1.950762
H	5.442803	0.794433	-3.011234
H	6.569792	0.257931	-1.755481
H	0.553991	-0.678328	-0.490068
H	1.197340	1.939328	1.050442
H	3.199871	-2.149079	4.021540
H	2.480679	-0.533171	4.083310
H	3.979969	-0.800652	3.171594
H	3.156399	-3.610649	0.994289
H	3.757878	-2.127018	0.233852
H	2.179480	-2.795663	-0.238024
H	-0.286928	-2.517073	1.481914
H	-0.206059	-1.776524	3.104449
H	0.519067	-3.361449	2.810096
H	-0.508017	0.874773	2.627902
H	-0.943178	-0.259581	1.334916
H	-1.291381	2.782873	1.235196
H	-1.551582	1.694911	-0.137256
H	-3.069112	1.533969	2.519173
H	-3.279484	0.453405	1.142091
H	-4.860389	3.661827	2.447474
H	-6.249577	4.796895	0.366083
H	1.433538	-1.053918	-2.485885
H	2.607076	0.105349	-3.333931
H	6.614856	3.263375	-0.663123
O	-4.195403	2.389139	-0.497449
H	-5.376347	3.430258	-1.856694
C	2.897228	1.866266	-1.227886
H	2.453771	2.582137	-0.528706
H	2.919547	2.369851	-2.201169

0 imaginary frequencies

E_{B3LYP}= -2396.868530

G_{B3LYP}= -2396.442274

$E_{M06\text{-}2X}$= -2396.750160

G_{tot}= -2396.320885

endo-TS allylsilane A and 2,5-dimethylfuran

Charge = 0 Multiplicity = 1

C	4.712337	-2.074767	-1.228755
C	3.376505	-2.568710	-1.411358
C	3.022423	-3.190030	-0.244928
O	4.060480	-3.101790	0.645772
C	1.107702	-0.192292	-1.219681
O	0.480738	0.699835	-0.264419
Si	-1.277658	0.592408	0.229659
C	-1.469283	-1.215696	0.677661
C	1.737906	-0.458443	0.069673
C	2.953712	0.159083	0.567599
Si	3.174245	2.960377	-0.240350
C	2.494130	3.350845	1.471558
C	3.326809	-0.215065	1.808351
C	4.724905	3.980186	-0.583438
C	1.894792	3.325546	-1.576539
C	-2.009396	1.194848	-1.380050
C	-1.194849	1.792070	1.654347
O	-3.930139	0.565218	1.131896
S	-5.229882	0.469975	0.409990
O	-6.406569	0.307421	1.287008
C	-5.100061	-1.170055	-0.452697
F	-4.888990	-2.158352	0.436400
O	-5.383356	1.432212	-0.704765
F	-4.074671	-1.177773	-1.325676
F	-6.223980	-1.447820	-1.128797
H	2.730232	-0.899239	2.401445
H	4.245262	0.151371	2.254302
H	5.484988	3.818451	0.188288
H	4.491398	5.049953	-0.607116
H	5.165670	3.713454	-1.550216
H	2.267532	3.045542	-2.567912
H	0.959837	2.790891	-1.395090
H	1.672251	4.397946	-1.598299
H	3.232111	3.128639	2.249681
H	2.228925	4.410246	1.552421

H	1.597919	2.760862	1.681097
H	1.265087	-1.209386	0.686897
H	1.663000	0.357145	-1.973940
H	-1.872189	0.451123	-2.171045
H	-1.496438	2.113397	-1.683394
H	-3.076462	1.402429	-1.288445
H	-0.453864	1.456890	2.387106
H	-0.899679	2.783762	1.298622
H	-2.167720	1.866233	2.141111
H	-1.206669	-1.879240	-0.152215
H	-0.839470	-1.466856	1.537173
H	-2.503404	-1.417403	0.953115
H	5.311054	-1.528152	-1.942246
H	2.768719	-2.502491	-2.302441
C	5.085910	-2.412784	0.036908
C	3.736335	1.121907	-0.275117
H	3.784353	0.778642	-1.313587
H	4.769547	1.126652	0.086725
H	0.448816	-0.953585	-1.629900
C	6.320249	-2.202805	0.836386
H	6.109358	-1.659875	1.763466
H	6.787818	-3.155119	1.110452
H	7.039022	-1.622697	0.254330
C	1.818651	-3.942298	0.197204
H	1.026866	-3.847582	-0.548687
H	2.038734	-5.007690	0.328661
H	1.440175	-3.569798	1.155469

1 imaginary frequency

E_{B3LYP}= -2358.752161

G_{B3LYP}= -2358.336641

E_{M06-2X}= -2358.636009

G_{tot}= -2358.217470

endo-INT allylsilane A and 2,5-dimethylfuran

Charge = 0 Multiplicity = 1

C	-4.743207	-2.568808	0.400965

C	-3.991397	-1.942992	1.338436
C	-2.550506	-2.121221	1.027585
O	-2.597601	-3.046758	-0.125490
C	-1.454632	0.155880	1.554548
O	-0.728075	1.230634	1.010221
Si	0.930911	1.173304	0.618170
C	1.147465	0.339048	-1.053349
C	-1.859143	-0.825100	0.440032
C	-2.703209	-0.197260	-0.652506
Si	-3.486013	2.634682	-0.423425
C	-2.130348	2.974399	-1.683920
C	-2.462431	-0.533195	-1.928420
C	-5.114850	3.381828	-1.029550
C	-3.073165	3.384165	1.255496
C	1.810126	0.266592	2.008667
C	1.416828	2.979276	0.525995
O	4.264133	1.392983	-0.396973
S	5.279108	0.380040	-0.016213
O	6.559205	0.473367	-0.753656
C	4.555248	-1.213427	-0.643401
F	4.239416	-1.116213	-1.947647
O	5.403055	0.140222	1.441500
F	3.436506	-1.546677	0.027609
F	5.431560	-2.222892	-0.502622
H	-1.648272	-1.197615	-2.198228
H	-3.060488	-0.131388	-2.740861
H	-5.387591	2.993804	-2.017067
H	-5.039667	4.472011	-1.106417
H	-5.935990	3.152668	-0.341133
H	-3.858307	3.164263	1.987489
H	-2.123220	2.994681	1.627894
H	-2.992261	4.473939	1.174128
H	-2.378673	2.550948	-2.662419
H	-1.985052	4.052818	-1.809762
H	-1.187169	2.534761	-1.354483
H	-0.939088	-1.211386	-0.005393
H	-2.343328	0.565963	2.046881
H	1.552732	-0.797505	2.031583

H	1.541656	0.702175	2.977654
H	2.893851	0.336810	1.886211
H	0.838029	3.507557	-0.238663
H	1.252966	3.483627	1.483742
H	2.477235	3.049614	0.268506
H	1.060206	-0.749659	-0.989439
H	0.399933	0.701641	-1.765447
H	2.145405	0.564746	-1.436685
H	-5.818815	-2.604794	0.312407
H	-4.346169	-1.379917	2.191294
C	-3.830444	-3.208644	-0.494082
C	-3.819072	0.740889	-0.280604
H	-4.186169	0.560516	0.735640
H	-4.668681	0.554510	-0.946197
H	-0.868707	-0.365886	2.317734
C	-4.121663	-4.018359	-1.689924
H	-3.204076	-4.354745	-2.169761
H	-4.733984	-4.878699	-1.398244
H	-4.718998	-3.423348	-2.387837
C	-1.738213	-2.813331	2.119816
H	-1.781231	-2.230299	3.040212
H	-2.150504	-3.804242	2.318601
H	-0.697283	-2.910009	1.805467

0 imaginary frequencies

E_{B3LYP}= -2358.793753

G_{B3LYP}= -2358.372289

E_{M06-2X}= -2358.680815

G_{tot}= -2358.256332

endo-TS2 allylsilane A and 2,5-dimethylfuran

Charge = 0 Multiplicity = 1

C	-5.073686	-1.940580	0.627650
C	-4.071744	-2.006884	1.519601
C	-2.811768	-2.322025	0.770157
O	-3.303159	-2.867620	-0.463995
C	-1.259787	-0.306469	1.428085

O	-0.624192	0.874361	0.988971
Si	1.026490	0.888732	0.548869
C	1.224798	-0.180717	-0.985139
C	-2.069530	-0.929461	0.287435
C	-3.142793	-0.085585	-0.304605
Si	-3.087783	2.757038	-0.232220
C	-1.972372	2.507045	-1.723857
C	-3.744827	-0.542525	-1.482486
C	-4.645596	3.675421	-0.762704
C	-2.249326	3.643611	1.191858
C	2.007392	0.250475	2.017342
C	1.389480	2.692029	0.205877
O	4.264668	1.216006	-0.728339
S	5.384973	0.417302	-0.172065
O	6.652977	0.511353	-0.929686
C	4.849317	-1.337193	-0.473906
F	4.539230	-1.524431	-1.770088
O	5.522546	0.479283	1.302767
F	3.765125	-1.656552	0.257298
F	5.829015	-2.198499	-0.151788
H	-3.149415	-1.091185	-2.202912
H	-4.577561	0.023854	-1.890696
H	-5.169066	3.142248	-1.563254
H	-4.386783	4.671985	-1.136964
H	-5.339716	3.799642	0.074914
H	-2.955411	3.816570	2.010674
H	-1.412680	3.049086	1.562601
H	-1.871556	4.616616	0.859488
H	-2.523530	2.065637	-2.560111
H	-1.574826	3.471557	-2.057136
H	-1.131444	1.855177	-1.483426
H	-1.389415	-1.279858	-0.491510
H	-1.908301	-0.063647	2.277560
H	1.826405	-0.812673	2.205888
H	1.730062	0.806833	2.919570
H	3.080392	0.375090	1.849769
H	0.772351	3.084192	-0.607595
H	1.208738	3.305550	1.094239

H	2.441182	2.786589	-0.078390
H	1.064506	-1.241375	-0.764288
H	0.512979	0.118198	-1.762256
H	2.236554	-0.070961	-1.381070
H	-6.102913	-1.651310	0.787064
H	-4.107242	-1.785284	2.577544
C	-4.503415	-2.276943	-0.687251
C	-3.740670	1.014849	0.434009
H	-3.541920	0.993803	1.505747
H	-4.817163	1.071390	0.251077
H	-0.533635	-1.042385	1.787005
C	-5.300654	-2.817427	-1.831526
H	-4.679705	-2.954797	-2.717094
H	-5.710224	-3.789107	-1.533972
H	-6.131698	-2.150476	-2.064093
C	-1.830884	-3.291020	1.401542
H	-1.528301	-2.940304	2.389169
H	-2.316141	-4.262201	1.516734
H	-0.942632	-3.407410	0.776637

1 imaginary frequency

E_{B3LYP}= -2358.780763

G_{B3LYP}= -2358.356824

E_{M06-2X}= -2358.665834

G_{tot}= -2358.238876

endo-Product allylsilane A and 2,5-dimethylfuran

Charge = 0 Multiplicity = 1

C	-4.996887	-2.118606	1.086212
C	-3.756245	-2.437010	1.452591
C	-2.860059	-2.321506	0.226637
O	-3.790765	-2.437378	-0.852420
C	-1.148995	-0.563552	1.121083
O	-0.558088	0.702824	0.873602
Si	1.110313	0.780085	0.496147
C	1.374085	-0.256982	-1.049958
C	-2.277837	-0.839851	0.126033

C	-3.450250	0.095870	0.228287
Si	-2.946954	2.711815	-0.307837
C	-1.988828	2.082857	-1.782748
C	-4.668876	-0.244537	-0.570136
C	-4.613005	3.393984	-0.822772
C	-1.978834	3.844945	0.817238
C	2.049385	0.143639	1.990284
C	1.452144	2.592597	0.180341
O	4.374045	1.195596	-0.662850
S	5.497525	0.416799	-0.084719
O	6.784377	0.554834	-0.802508
C	5.015559	-1.345327	-0.429191
F	4.752185	-1.519761	-1.737487
O	5.588909	0.459088	1.394297
F	3.916197	-1.701574	0.261161
F	6.005043	-2.187472	-0.087749
H	-4.470943	-0.093127	-1.637221
H	-5.517655	0.375453	-0.275660
H	-5.204458	2.661415	-1.378962
H	-4.444421	4.251979	-1.483494
H	-5.195821	3.736344	0.036640
H	-2.601363	4.203112	1.641604
H	-1.109766	3.324488	1.222019
H	-1.632096	4.709996	0.241602
H	-2.594722	1.449620	-2.436520
H	-1.673538	2.953532	-2.368832
H	-1.097873	1.536389	-1.477253
H	-1.878005	-0.775943	-0.893554
H	-1.535388	-0.594363	2.147762
H	1.851162	-0.917856	2.171887
H	1.742975	0.701347	2.882270
H	3.127978	0.262932	1.860213
H	0.834727	2.993033	-0.629455
H	1.278487	3.198582	1.075100
H	2.503512	2.691043	-0.104748
H	1.181624	-1.320214	-0.874155
H	0.715480	0.077429	-1.859047
H	2.407888	-0.152927	-1.385121

H	-5.876126	-2.028528	1.711580
H	-3.392995	-2.672019	2.445068
C	-4.974594	-1.774551	-0.396955
C	-3.454420	1.212304	1.058539
H	-2.656598	1.317917	1.786600
H	-4.428741	1.599631	1.354931
H	-0.413267	-1.368462	1.036393
C	-6.168820	-2.215313	-1.217523
H	-6.005340	-2.000176	-2.276739
H	-6.322319	-3.289947	-1.093958
H	-7.070418	-1.692170	-0.888934
C	-1.790097	-3.386916	0.072744
H	-1.134259	-3.423108	0.944570
H	-2.281080	-4.357742	-0.025533
H	-1.183269	-3.205777	-0.818429

0 imaginary frequencies

E_{B3LYP}= -2358.794792

G_{B3LYP}= -2358.369703

E_{M06-2X}= -2358.686547

G_{tot}= -2358.258439

exo-TS allylsilane A and 2,5-dimethylfuran

Charge = 0 Multiplicity = 1

C	-3.599770	-3.425303	-0.870541
C	-4.766142	-2.728020	-0.731732
O	-4.812293	-2.165450	0.521008
C	-3.652811	-2.495776	1.176190
C	-2.879087	-3.273565	0.357664
C	-1.021385	-0.175784	1.281078
C	-1.647811	-0.482608	-0.002995
C	-2.865595	0.109508	-0.526143
C	-3.221441	-0.293770	-1.763773
O	-0.402301	0.667393	0.278710
Si	1.367020	0.560011	-0.224477
C	1.222359	1.729475	-1.671165
C	1.536983	-1.260638	-0.620020

C	2.069020	1.214127	1.382420
Si	-3.138602	2.934520	0.156968
C	-1.830455	3.368946	1.444004
C	-2.502342	3.264217	-1.584434
C	-4.694458	3.947806	0.495771
F	4.127739	-1.213858	1.325669
C	5.250825	-0.622028	0.875947
F	5.457710	0.484842	1.610961
F	6.275135	-1.461537	1.084032
S	5.099921	-0.197225	-0.925877
O	3.951865	0.754996	-0.947400
O	6.402524	0.414801	-1.251924
O	4.820060	-1.504411	-1.561550
H	-2.607843	-0.977787	-2.337536
H	-4.133055	0.060924	-2.232163
H	-5.470259	3.744625	-0.250076
H	-4.475417	5.020591	0.470092
H	-5.108564	3.715339	1.482960
H	-2.178007	3.134549	2.456154
H	-0.898089	2.828722	1.265139
H	-1.611932	4.441840	1.409981
H	-3.252729	2.998508	-2.336595
H	-2.256657	4.323499	-1.714140
H	-1.601031	2.680291	-1.789596
H	-1.189121	-1.272696	-0.580351
H	-1.566461	0.411545	2.013601
H	1.980074	0.470656	2.180127
H	1.514061	2.108122	1.683898
H	3.121228	1.471358	1.271795
H	0.464925	1.361305	-2.370635
H	0.913115	2.720933	-1.326620
H	2.175459	1.816605	-2.192513
H	1.257042	-1.887982	0.232611
H	0.890944	-1.519311	-1.465650
H	2.561000	-1.512367	-0.897528
H	-3.289211	-3.970662	-1.749705
H	-1.916915	-3.694838	0.611719
C	-3.666486	1.090545	0.280876

H	-3.697110	0.806229	1.337862
H	-4.703049	1.055463	-0.069400
H	-0.363487	-0.922834	1.718317
C	-5.950405	-2.515062	-1.604006
H	-6.834747	-3.028440	-1.209371
H	-6.196172	-1.452250	-1.688969
H	-5.746570	-2.904462	-2.603318
C	-3.531142	-2.051884	2.589568
H	-3.708463	-0.976840	2.693822
H	-4.260213	-2.562142	3.229243
H	-2.532569	-2.281110	2.967040

1 imaginary frequency

E_{B3LYP}= -2358.752718

G_{B3LYP}= -2358.337414

E_{M06-2X}= -2358.636554

G_{tot}= -2358.218231

exo-INT allylsilane A and 2,5-dimethylfuran

Charge = 0 Multiplicity = 1

C	-4.082734	-2.945997	-0.748641
C	-4.589202	-1.654225	-1.276772
O	-5.449057	-1.185768	-0.160440
C	-5.336986	-2.004230	0.837350
C	-4.517184	-3.133600	0.517919
C	-1.200468	-0.418926	1.733993
C	-1.886422	-0.813412	0.452843
C	-2.876503	-0.112540	-0.123541
C	-3.461955	-0.584285	-1.438323
O	-0.327325	0.699275	1.556964
Si	1.270245	0.513307	1.025330
C	1.859089	2.257757	0.682010
C	1.328986	-0.551585	-0.525412
C	2.276690	-0.288004	2.396856
Si	-2.672247	2.756874	-0.311920
C	-1.819819	3.704701	1.073457
C	-1.435177	2.314917	-1.664115

C	-4.070165	3.791905	-1.052047
F	4.258794	-1.892043	0.208804
C	5.479385	-1.484929	-0.192048
F	6.346756	-1.752491	0.799189
F	5.826916	-2.222356	-1.260752
S	5.481912	0.325999	-0.604330
O	5.078463	0.960887	0.674454
O	6.883730	0.575594	-1.011298
O	4.485485	0.425305	-1.698360
H	-2.702435	-1.033172	-2.083126
H	-3.905644	0.250657	-1.986308
H	-4.578768	3.250849	-1.857834
H	-3.687524	4.729842	-1.468989
H	-4.819591	4.043833	-0.293639
H	-2.540717	4.023308	1.834569
H	-1.076219	3.058923	1.548484
H	-1.314629	4.598560	0.692128
H	-1.914422	1.813970	-2.511840
H	-0.944360	3.216926	-2.044924
H	-0.664793	1.649515	-1.269099
H	-1.503382	-1.695100	-0.056477
H	-1.942017	-0.124337	2.485946
H	1.908269	-1.289988	2.642966
H	2.245820	0.317747	3.308853
H	3.317959	-0.375304	2.075911
H	1.321342	2.704396	-0.159963
H	1.712100	2.901451	1.555650
H	2.927449	2.232378	0.447133
H	1.066692	-1.592759	-0.308525
H	0.636399	-0.183241	-1.288500
H	2.340537	-0.534314	-0.941233
H	-3.433612	-3.595088	-1.322113
H	-4.311278	-3.952520	1.190961
C	-3.450684	1.157036	0.432530
H	-3.304378	1.212759	1.515619
H	-4.529943	1.194468	0.249438
H	-0.656149	-1.280023	2.142869
C	-5.463446	-1.776509	-2.520999

H	-6.251079	-2.518055	-2.376593
H	-5.910180	-0.806960	-2.747772
H	-4.836853	-2.082467	-3.361269
C	-6.009724	-1.679270	2.105078
H	-6.737199	-0.879654	1.972357
H	-6.477651	-2.574147	2.522265
H	-5.240951	-1.354131	2.818045

0 imaginary frequencies

E_{B3LYP}= -2358.793806

G_{B3LYP}= -2358.376082

E_{M06-2X}= -2358.680941

G_{tot}= -2358.260198

exo-TS2 allylsilane A and 2,5-dimethylfuran

Charge = 0 Multiplicity = 1

C	3.720323	-2.993570	1.362167
C	4.620051	-1.836608	1.041283
O	4.670536	-1.874911	-0.397437
C	3.426681	-2.324568	-0.784979
C	2.996394	-3.269434	0.268449
C	1.376774	-0.411954	-1.613927
C	2.159865	-0.849102	-0.389920
C	3.167129	-0.009513	0.161145
C	3.820935	-0.458638	1.415066
O	0.583911	0.722212	-1.301479
Si	-1.050287	0.588613	-0.836142
C	-1.581494	2.354506	-0.524339
C	-1.166783	-0.461457	0.719138
C	-2.010739	-0.190178	-2.248620
Si	2.955019	2.785539	0.185239
C	2.111025	3.629019	-1.258539
C	1.770921	2.400947	1.591394
C	4.427516	3.768330	0.819408
F	-4.064635	-1.746822	-0.142250
C	-5.288641	-1.304531	0.208922
F	-6.122647	-1.549588	-0.815824

F	-5.699144	-2.028413	1.264057
S	-5.254679	0.506862	0.618186
O	-4.785842	1.126681	-0.645905
O	-6.661828	0.799431	0.973198
O	-4.295400	0.579647	1.746974
H	3.108263	-0.679821	2.208931
H	4.547126	0.271293	1.773332
H	4.945220	3.239268	1.626011
H	4.087767	4.732001	1.214378
H	5.148726	3.964990	0.019990
H	2.831427	3.878057	-2.044055
H	1.343946	2.970708	-1.672343
H	1.635507	4.557219	-0.923828
H	2.285513	1.964707	2.452897
H	1.292604	3.327794	1.925552
H	0.986572	1.714321	1.268796
H	1.538579	-1.345752	0.347340
H	2.044955	-0.140494	-2.436401
H	-1.694395	-1.219223	-2.449521
H	-1.886871	0.390233	-3.168991
H	-3.072169	-0.203456	-1.988295
H	-1.046293	2.796519	0.321389
H	-1.401077	2.980075	-1.404609
H	-2.653849	2.362093	-0.307808
H	-0.910100	-1.508549	0.523548
H	-0.498725	-0.087332	1.502278
H	-2.190071	-0.428761	1.103575
H	3.620917	-3.417402	2.352794
H	2.173359	-3.962709	0.158855
C	3.744141	1.102682	-0.544359
H	3.524666	1.132467	-1.611056
H	4.812845	1.216195	-0.348789
H	0.762424	-1.249951	-1.963679
C	6.018580	-1.833728	1.617246
H	6.532782	-2.762002	1.359047
H	6.587043	-0.987369	1.224769
H	5.969637	-1.748554	2.705445
C	3.335060	-2.622586	-2.249897

H	3.654472	-1.769153	-2.849253
H	4.005663	-3.461513	-2.462230
H	2.322531	-2.912605	-2.530876

1 imaginary frequency

E_{B3LYP}= -2358.774765

G_{B3LYP}= -2358.351754

E_{M06-2X}= -2358.659432

G_{tot}= -2358.233402

exo-Product allylsilane A and 2,5-dimethylfuran

Charge = 0 Multiplicity = 1

C	-4.369283	-2.310639	-1.621977
C	-4.966602	-1.822196	-0.312240
O	-4.075842	-2.386534	0.669815
C	-2.775466	-2.324173	0.053420
C	-3.089242	-2.593930	-1.409906
C	-1.139902	-0.598995	1.211623
C	-2.263616	-0.824901	0.185591
C	-3.460036	0.072310	0.405355
C	-4.786821	-0.275963	-0.211677
O	-0.545576	0.675633	1.018419
Si	1.116494	0.775012	0.619025
C	1.437617	2.603023	0.381411
C	1.355834	-0.189545	-0.976648
C	2.083203	0.074654	2.066588
Si	-3.018022	2.711880	-0.239874
C	-2.081454	3.936337	0.814784
C	-2.046489	2.074457	-1.700996
C	-4.726738	3.295670	-0.732789
F	3.904068	-1.700117	0.135421
C	5.161974	-1.223365	0.187842
F	5.486850	-1.063743	1.483929
F	5.975965	-2.156607	-0.333248
S	5.321041	0.382832	-0.734669
O	4.495001	1.319616	0.066980
O	6.776761	0.646441	-0.695678

O	4.771544	0.056891	-2.072630
H	-4.842542	0.172242	-1.212085
H	-5.582350	0.182533	0.381382
H	-5.301949	2.525331	-1.253249
H	-4.613941	4.142131	-1.419735
H	-5.301355	3.635322	0.133284
H	-2.684720	4.265545	1.665118
H	-1.154628	3.493628	1.183953
H	-1.830345	4.813018	0.207945
H	-2.599489	1.337243	-2.289882
H	-1.831114	2.925402	-2.356827
H	-1.097432	1.641243	-1.385255
H	-1.838685	-0.542008	-0.786709
H	-1.534756	-0.670652	2.232132
H	1.925787	-1.002123	2.184471
H	1.776157	0.567592	2.995392
H	3.151031	0.245423	1.916498
H	0.826231	3.025658	-0.421818
H	1.239679	3.169130	1.297352
H	2.491577	2.730826	0.119337
H	1.124163	-1.252367	-0.848793
H	0.702618	0.203711	-1.763620
H	2.388664	-0.116804	-1.326172
H	-4.913088	-2.319629	-2.558623
H	-2.336619	-2.889601	-2.130607
C	-3.374378	1.232475	1.174283
H	-2.493879	1.368309	1.795064
H	-4.310142	1.617783	1.577626
H	-0.403421	-1.397421	1.095306
C	-6.388073	-2.256748	-0.019335
H	-6.469433	-3.343180	-0.100939
H	-6.677917	-1.953744	0.990385
H	-7.078367	-1.797515	-0.732493
C	-1.886692	-3.369136	0.705324
H	-1.771593	-3.187878	1.776271
H	-2.354747	-4.346532	0.568336
H	-0.898715	-3.392600	0.239443

0 imaginary frequencies

E_{B3LYP}= -2358.783805
G_{B3LYP}= -2358.358206
E_{M06-2X}= -2358.676011
G_{tot}= -2358.247393

endo-TS allylsilane A and furan

Charge = 0 Multiplicity = 1

C	-5.327134	2.629207	-0.488482
C	-4.163123	2.787159	-1.306926
C	-3.220167	3.387210	-0.520678
O	-3.724388	3.618048	0.726895
C	-1.334848	0.549285	-1.177117
O	-0.754919	-0.400455	-0.249001
Si	1.020592	-0.434006	0.218610
C	1.322617	1.338452	0.742496
C	-1.931455	0.813806	0.129454
C	-3.178360	0.261070	0.622525
Si	-3.590977	-2.502424	-0.230646
C	-2.933758	-2.963180	1.472808
C	-3.527847	0.633848	1.871399
C	-5.205417	-3.414025	-0.583572
C	-2.339854	-2.928422	-1.575495
C	1.671060	-1.001870	-1.438716
C	0.854490	-1.694353	1.583469
O	3.622291	-0.637349	1.057955
S	4.930368	-0.608099	0.343533
O	6.109117	-0.591080	1.232062
C	4.937093	1.082079	-0.426554
F	4.803556	2.033198	0.516372
O	5.011783	-1.516963	-0.821943
F	3.918256	1.219786	-1.296052
F	6.081979	1.304695	-1.087300
H	-2.888123	1.266395	2.476772
H	-4.465632	0.314346	2.312476
H	-5.951331	-3.214970	0.193236

H	-5.041543	-4.496189	-0.623436
H	-5.630741	-3.105459	-1.544793
H	-2.700602	-2.620228	-2.562911
H	-1.376493	-2.446867	-1.394014
H	-2.176495	-4.011189	-1.606495
H	-3.653513	-2.704905	2.256838
H	-2.738176	-4.038756	1.536776
H	-2.000296	-2.436347	1.687880
H	-1.414890	1.527561	0.757506
H	-1.929479	0.053272	-1.938442
H	1.578771	-0.205338	-2.183666
H	1.079539	-1.857525	-1.780461
H	2.719321	-1.298360	-1.384910
H	0.137705	-1.342925	2.332530
H	0.487619	-2.642677	1.179424
H	1.817554	-1.863787	2.065041
H	1.068370	2.050681	-0.049158
H	0.729981	1.580093	1.630859
H	2.372611	1.478721	0.993987
H	-6.269669	2.184191	-0.769984
H	-4.044423	2.499956	-2.341445
C	-5.004567	3.138951	0.734054
H	-5.542666	3.247035	1.662015
C	-4.026972	-0.629887	-0.235466
H	-4.050745	-0.269146	-1.269071
H	-5.058015	-0.567773	0.127295
H	-0.635495	1.280355	-1.575355
H	-2.219387	3.746726	-0.700631

1 imaginary frequency

E_{B3LYP}= -2280.089084

G_{B3LYP}= -2279.725772

E_{M06-2X}= -2280.001991

G_{tot}= -2279.635660

endo-INT-S_N2’ allylsilane A and furan

Charge = 0 Multiplicity = 1

C	-5.631834	-2.047150	-0.076807
C	-5.218404	-2.578037	1.109563
C	-4.046188	-3.315756	0.814208
O	-3.766717	-3.343816	-0.454787
C	-1.396337	-0.481435	1.662307
O	-0.719145	0.746336	1.400154
Si	0.883432	0.783244	0.835618
C	1.038412	-0.299766	-0.695569
C	-2.036805	-1.038180	0.417518
C	-3.136408	-0.513457	-0.168860
Si	-3.428527	2.340720	-0.419700
C	-2.194415	2.078987	-1.818679
C	-3.667137	-1.137814	-1.426763
C	-5.011745	3.110233	-1.102202
C	-2.688464	3.424283	0.927050
C	1.995158	0.191199	2.230412
C	1.180191	2.588291	0.433043
O	4.078833	1.208740	-0.665245
S	5.249615	0.419404	-0.208899
O	6.451820	0.538662	-1.064617
C	4.717198	-1.340286	-0.485611
F	4.300324	-1.520002	-1.752708
O	5.504031	0.466405	1.250844
F	3.706198	-1.686397	0.332389
F	5.735316	-2.188126	-0.259411
H	-2.877439	-1.540208	-2.062913
H	-4.267972	-0.440675	-2.014792
H	-5.457012	2.478238	-1.878584
H	-4.809880	4.091649	-1.544735
H	-5.757416	3.246332	-0.311368
H	-3.421887	3.631751	1.713800
H	-1.832501	2.911771	1.373953
H	-2.350135	4.382595	0.518674
H	-2.612401	1.475070	-2.630759
H	-1.895100	3.043188	-2.243330
H	-1.296829	1.577889	-1.449968
H	-1.520373	-1.852241	-0.083544

H	-2.160406	-0.272912	2.419926
H	1.798938	-0.856001	2.485209
H	1.821636	0.793983	3.128740
H	3.050211	0.273433	1.957000
H	0.509720	2.935941	-0.359104
H	1.022319	3.222558	1.311370
H	2.212715	2.709417	0.093455
H	0.967689	-1.364299	-0.450660
H	0.256797	-0.066586	-1.425343
H	2.012633	-0.127362	-1.158639
H	-6.501575	-1.430716	-0.258514
H	-5.663757	-2.479209	2.087842
C	-4.622930	-2.363519	-1.105111
H	-4.992305	-2.795718	-2.035254
C	-3.910266	0.639725	0.372772
H	-3.778345	0.741524	1.453435
H	-4.982874	0.503075	0.184357
H	-0.714809	-1.227240	2.090438
H	-3.409446	-3.895006	1.471994

0 imaginary frequencies

E_{B3LYP}= -2280.125940

G_{B3LYP}= -2279.757098

E_{M06-2X}= -2280.040499

G_{tot}= -2279.668638

endo-INT-S_N2 allylsilane A and furan

Charge = 0 Multiplicity = 1

C	-4.768767	-3.185119	0.312929
C	-4.062206	-2.495980	1.256507
C	-2.630371	-2.554564	0.913780
O	-2.583885	-3.502288	-0.191586
C	-1.666483	-0.279090	1.523302
O	-0.989994	0.860189	1.060553
Si	0.673515	0.924550	0.679899
C	0.953415	0.113457	-0.993808

C	-2.008692	-1.210720	0.348348
C	-2.903110	-0.624238	-0.717160
Si	-3.845620	2.141168	-0.401704
C	-2.487272	2.578246	-1.628121
C	-2.662407	-0.937774	-2.001064
C	-5.502592	2.812551	-1.015452
C	-3.494406	2.855177	1.305388
C	1.604946	0.077021	2.072673
C	1.021898	2.761757	0.603873
O	3.959701	1.465719	-0.424841
S	5.056950	0.572602	0.023035
O	6.346731	0.772981	-0.674908
C	4.518093	-1.103585	-0.574257
F	4.233356	-1.074667	-1.888736
O	5.152682	0.391027	1.491414
F	3.418852	-1.530745	0.075359
F	5.487531	-2.013595	-0.378490
H	-1.808333	-1.541913	-2.288465
H	-3.298250	-0.569993	-2.800545
H	-5.738623	2.434570	-2.016110
H	-5.484322	3.906696	-1.065390
H	-6.319950	2.523093	-0.345710
H	-4.279732	2.577003	2.016922
H	-2.532804	2.499235	1.682077
H	-3.462341	3.949438	1.258062
H	-2.697930	2.169130	-2.621389
H	-2.392008	3.665267	-1.723563
H	-1.529437	2.176303	-1.292263
H	-1.070245	-1.535885	-0.105184
H	-2.579703	0.054925	2.027890
H	1.416937	-1.001515	2.094157
H	1.301995	0.494191	3.039512
H	2.682827	0.218690	1.957650
H	0.414350	3.250373	-0.164707
H	0.809448	3.245370	1.562726
H	2.076987	2.911483	0.359101
H	0.954551	-0.979123	-0.931963
H	0.178657	0.417041	-1.704822

H	1.929408	0.419658	-1.377878
H	-5.837755	-3.303058	0.221816
H	-4.455512	-1.960679	2.109889
C	-3.804865	-3.746286	-0.554730
H	-3.941617	-4.366514	-1.432703
C	-4.073031	0.224210	-0.313146
H	-4.416238	-0.002945	0.702687
H	-4.914843	0.006817	-0.978641
H	-1.070068	-0.836083	2.258656
H	-1.981608	-2.943610	1.702265

0 imaginary frequencies

E_{B3LYP}= -2280.124382

G_{B3LYP}= -2279.755326

$E_{M06\text{-}2X}$= -2280.039494

G_{tot}= -2279.667419

endo-TS2-S_N2’ allylsilane A and furan

Charge = 0 Multiplicity = 1

C	-5.391004	-2.340947	0.299529
C	-4.514072	-2.553968	1.301374
C	-3.250953	-2.918188	0.667117
O	-3.494458	-3.273269	-0.608786
C	-1.438574	-0.673721	1.455086
O	-0.829562	0.582743	1.202247
Si	0.803005	0.704725	0.718148
C	1.034240	-0.315295	-0.845240
C	-2.171825	-1.162924	0.225499
C	-3.304324	-0.504152	-0.275381
Si	-3.475295	2.312328	-0.358999
C	-2.275770	2.057497	-1.782859
C	-3.991501	-1.086843	-1.454156
C	-5.082679	3.064925	-0.985384
C	-2.723708	3.325930	1.027697
C	1.859329	0.093548	2.144703
C	1.043447	2.534457	0.406991
O	3.987294	1.275593	-0.625562

S	5.172489	0.530625	-0.132522
O	6.406612	0.729450	-0.925152
C	4.737439	-1.243286	-0.478904
F	4.396445	-1.407447	-1.770557
O	5.354326	0.551066	1.338647
F	3.700592	-1.654839	0.274418
F	5.779437	-2.050499	-0.218026
H	-3.313190	-1.344653	-2.267771
H	-4.798826	-0.454966	-1.825680
H	-5.540592	2.445056	-1.763200
H	-4.891626	4.054175	-1.415338
H	-5.807222	3.183663	-0.173366
H	-3.454484	3.507228	1.822439
H	-1.868284	2.792657	1.448766
H	-2.382605	4.295663	0.649986
H	-2.725557	1.482768	-2.598589
H	-1.973147	3.028789	-2.188272
H	-1.377086	1.536246	-1.447940
H	-1.573399	-1.715926	-0.492421
H	-2.136066	-0.552780	2.291510
H	1.714265	-0.975995	2.330052
H	1.594437	0.633866	3.060250
H	2.921798	0.253134	1.943678
H	0.391355	2.897964	-0.392908
H	0.832903	3.119907	1.307728
H	2.082916	2.704208	0.112859
H	0.951786	-1.389158	-0.647350
H	0.289431	-0.049123	-1.602575
H	2.028075	-0.127046	-1.256774
H	-6.417854	-2.010561	0.369130
H	-4.657748	-2.434856	2.365400
C	-4.637487	-2.492821	-0.981500
H	-5.145331	-2.953105	-1.826056
C	-3.973909	0.570531	0.433723
H	-3.697068	0.645096	1.485819
H	-5.061282	0.516287	0.323340
H	-0.705101	-1.431160	1.758652
H	-2.422120	-3.424160	1.147459

1 imaginary frequency
E_{B3LYP}= -2280.121520
G_{B3LYP}= -2279.750108
$E_{M06\text{-}2X}$= -2280.034715
G_{tot}= -2279.660284

endo-TS2-S_N2 allylsilane A and furan

Charge = 0 Multiplicity = 1

C	-5.080250	-2.634313	0.411501
C	-4.144987	-2.536872	1.381175
C	-2.818074	-2.704758	0.726895
O	-3.125350	-3.361878	-0.504944
C	-1.535684	-0.568756	1.439147
O	-0.926885	0.633376	1.032873
Si	0.726795	0.753582	0.621357
C	1.016991	-0.215838	-0.964019
C	-2.183363	-1.271680	0.242978
C	-3.287605	-0.568268	-0.463713
Si	-3.581047	2.274356	-0.326559
C	-2.344352	2.208552	-1.739813
C	-3.708648	-1.090189	-1.683549
C	-5.187026	3.060375	-0.921700
C	-2.921923	3.170778	1.183871
C	1.720035	0.096435	2.072557
C	0.980365	2.590566	0.372263
O	3.957691	1.349391	-0.572665
S	5.113065	0.573792	-0.056567
O	6.381253	0.783775	-0.790193
C	4.673729	-1.182398	-0.480122
F	4.380303	-1.298914	-1.788380
O	5.233270	0.543405	1.420758
F	3.605167	-1.607604	0.219583
F	5.696916	-2.009724	-0.208092
H	-3.013772	-1.630194	-2.315988
H	-4.566869	-0.646884	-2.180451
H	-5.599741	2.519375	-1.779708

H	-5.009876	4.096204	-1.231061
H	-5.942963	3.070072	-0.129603
H	-3.683052	3.223863	1.969306
H	-2.043574	2.652070	1.573299
H	-2.637138	4.195074	0.919737
H	-2.773154	1.723325	-2.622226
H	-2.048446	3.223553	-2.025431
H	-1.446838	1.660761	-1.448283
H	-1.407893	-1.576601	-0.461230
H	-2.282381	-0.337928	2.206758
H	1.595491	-0.983484	2.202761
H	1.400315	0.589075	2.997459
H	2.786770	0.287954	1.929368
H	0.353600	2.978463	-0.436357
H	0.740956	3.147862	1.283565
H	2.028364	2.767942	0.115664
H	0.964849	-1.297030	-0.799162
H	0.278510	0.051816	-1.727338
H	2.012916	0.012739	-1.349762
H	-6.145581	-2.469776	0.478991
H	-4.288010	-2.282112	2.421762
C	-4.361848	-2.940263	-0.814508
H	-4.783921	-3.331106	-1.731518
C	-4.088639	0.448063	0.212192
H	-4.012605	0.413531	1.300303
H	-5.138566	0.376794	-0.084645
H	-0.816399	-1.261483	1.895163
H	-2.044154	-3.252661	1.260957

1 imaginary frequency

E_{B3LYP}= -2280.118341

G_{B3LYP}= -2279.747180

E_{M06-2X}= -2280.030861

G_{tot}= -2279.656681

endo-Product allylsilane A and furan

Charge = 0 Multiplicity = 1

C	5.137400	-2.705310	-0.880751
C	3.857532	-2.973290	-1.145150
C	3.064974	-2.664786	0.113063
O	4.036209	-2.774115	1.148180
C	1.403702	-0.961467	-0.865206
O	0.857528	0.339469	-0.730478
Si	-0.819745	0.539552	-0.454708
C	-1.214422	-0.314535	1.172146
C	2.564303	-1.167640	0.107681
C	3.772344	-0.303003	-0.130637
Si	3.385648	2.349386	0.287912
C	2.392581	1.841744	1.784812
C	5.023602	-0.663902	0.614044
C	5.099158	2.939672	0.753631
C	2.478426	3.486531	-0.882890
C	-1.722549	-0.195016	-1.925742
C	-1.066109	2.390852	-0.341501
O	-4.107200	1.304720	0.532964
S	-5.239855	0.558402	-0.069888
O	-6.564198	0.852767	0.521544
C	-4.924612	-1.194425	0.462802
F	-4.768992	-1.265697	1.797557
O	-5.212880	0.471536	-1.549740
F	-3.810464	-1.690963	-0.106659
F	-5.950520	-1.990988	0.119637
H	4.914005	-0.403309	1.672909
H	5.888655	-0.134716	0.209711
H	5.648904	2.196146	1.337143
H	4.995477	3.836051	1.375603
H	5.691501	3.204001	-0.126522
H	3.103878	3.750885	-1.739639
H	1.561202	3.014696	-1.238711
H	2.212614	4.405774	-0.349488
H	2.951606	1.187398	2.459286
H	2.141485	2.751572	2.341597
H	1.464884	1.350646	1.492428
H	2.210317	-0.985367	1.129734
H	1.746323	-1.112162	-1.897041

H	-1.583840	-1.279562	-1.985998
H	-1.337922	0.246345	-2.852005
H	-2.796363	0.001498	-1.868554
H	-0.461334	2.837238	0.454009
H	-0.816487	2.889611	-1.283415
H	-2.121034	2.577271	-0.120641
H	-1.068303	-1.398178	1.118000
H	-0.575757	0.074122	1.973105
H	-2.255825	-0.126811	1.441075
H	5.980606	-2.732880	-1.558396
H	3.418601	-3.274731	-2.087112
C	5.208224	-2.215910	0.555075
H	6.087551	-2.516004	1.125511
C	3.769234	0.758345	-1.026222
H	2.926599	0.858154	-1.703481
H	4.735102	1.097143	-1.399084
H	0.658687	-1.740655	-0.660758
H	2.225300	-3.327123	0.328570

0 imaginary frequencies

E_{B3LYP}= -2280.135576

G_{B3LYP}= -2279.762801

E_{M06-2X}= -2280.055933

G_{tot}= -2279.680139

exo-TS allylsilane A and furan

Charge = 0 Multiplicity = 1

C	-3.613834	3.970725	0.716193
C	-4.810478	3.321132	0.635858
O	-4.912895	2.660424	-0.555048
C	-3.748421	2.876031	-1.232309
C	-2.919339	3.680557	-0.502121
C	-1.330932	0.415075	-1.284532
C	-1.927273	0.779159	-0.002177
C	-3.193444	0.298205	0.519414
C	-3.522840	0.748529	1.748314
O	-0.784356	-0.479977	-0.282923

Si	0.988285	-0.517678	0.199504
C	0.796302	-1.711674	1.619276
C	1.308737	1.270972	0.641870
C	1.642647	-1.179417	-1.423466
Si	-3.683690	-2.497891	-0.159847
C	-2.435270	-3.041485	-1.464696
C	-3.045784	-2.866884	1.573070
C	-5.318889	-3.389096	-0.466619
F	3.863755	1.098125	-1.333146
C	4.955449	0.424848	-0.921653
F	5.076967	-0.673087	-1.688528
F	6.026796	1.203422	-1.130996
S	4.813196	-0.036785	0.871770
O	3.614735	-0.923221	0.891935
O	6.086792	-0.726297	1.156886
O	4.618605	1.268006	1.543836
H	-2.856249	1.384582	2.318815
H	-4.467754	0.484773	2.211129
H	-6.064014	-3.122046	0.290297
H	-5.183210	-4.475479	-0.436850
H	-5.730300	-3.131046	-1.448564
H	-2.775776	-2.779704	-2.472469
H	-1.457896	-2.582663	-1.299793
H	-2.308111	-4.129020	-1.431081
H	-3.762484	-2.543826	2.335557
H	-2.875504	-3.940601	1.704771
H	-2.101262	-2.348957	1.759992
H	-1.400663	1.527737	0.573635
H	-1.928785	-0.121141	-2.015573
H	1.619854	-0.409946	-2.200740
H	1.022911	-2.019636	-1.752231
H	2.671074	-1.520314	-1.312598
H	0.097706	-1.305297	2.357606
H	0.397607	-2.665920	1.262022
H	1.758520	-1.887196	2.100667
H	1.062803	1.943189	-0.186871
H	0.702212	1.554271	1.508649
H	2.355116	1.432810	0.903415

H	-3.264871	4.572809	1.541840
H	-1.939599	4.026269	-0.797922
H	-5.662280	3.244844	1.292275
C	-4.069777	-0.618629	-0.284590
H	-4.084595	-0.326241	-1.339947
H	-5.098380	-0.506420	0.072911
H	-0.610326	1.100895	-1.723134
H	-3.683041	2.433876	-2.213775

1 imaginary frequency

E_{B3LYP}= -2280.089676

G_{B3LYP}= -2279.726541

$E_{M06\text{-}2X}$= -2280.002751

G_{tot}= -2279.636597

exo-INT-S_N2' allylsilane A and furan

Charge = 0 Multiplicity = 1

C	-5.469622	-2.734693	0.906269
C	-4.448524	-3.706399	0.769603
C	-1.382558	-0.364849	1.728637
O	-0.670428	0.841332	1.445676
Si	0.920942	0.830024	0.858738
C	1.046727	-0.299411	-0.642032
C	-2.031298	-0.941006	0.497735
C	-3.133189	-0.442211	-0.083950
Si	-3.389851	2.408914	-0.434487
C	-2.156012	2.086754	-1.823361
C	-3.654437	-1.081116	-1.357630
C	-4.952522	3.197017	-1.147583
C	-2.625091	3.530776	0.869096
C	2.047147	0.261031	2.252787
C	1.249507	2.615496	0.394766
O	4.119521	1.151003	-0.699178
S	5.282161	0.349900	-0.243089
O	6.470107	0.421689	-1.123840
C	4.709853	-1.405294	-0.462562
F	4.265515	-1.610285	-1.716550

O	5.565176	0.429770	1.209894
F	3.708167	-1.709349	0.382925
F	5.715352	-2.267385	-0.232817
H	-2.842921	-1.426992	-2.003358
H	-4.258403	-0.378888	-1.935834
H	-5.410773	2.551857	-1.905466
H	-4.727501	4.159662	-1.619410
H	-5.697802	3.374470	-0.364447
H	-3.351238	3.780295	1.650654
H	-1.777565	3.018299	1.332594
H	-2.269220	4.467751	0.427363
H	-2.587714	1.479786	-2.626100
H	-1.822902	3.032104	-2.264909
H	-1.277811	1.564306	-1.437704
H	-1.521919	-1.778073	0.024024
H	-2.143372	-0.115176	2.477112
H	1.837214	-0.774605	2.541944
H	1.899098	0.892693	3.135756
H	3.098820	0.317016	1.960032
H	0.573367	2.950822	-0.397993
H	1.117964	3.281050	1.254124
H	2.278777	2.705062	0.035847
H	0.958714	-1.355025	-0.366201
H	0.264164	-0.073534	-1.372888
H	2.020936	-0.157091	-1.115257
H	-6.157044	-2.569530	1.728774
C	-4.543158	-2.321769	-1.088686
H	-5.078884	-2.608190	-2.000840
C	-3.897288	0.749643	0.410504
H	-3.751162	0.889770	1.485967
H	-4.970864	0.605125	0.246826
H	-0.720256	-1.113233	2.182906
H	-4.199685	-4.458607	1.502652
C	-3.886199	-3.478954	-0.448884
H	-3.073512	-4.025167	-0.909930
O	-5.584335	-1.954595	-0.115876

0 imaginary frequencies

E_{B3LYP}= -2280.121833

G_{B3LYP}= -2279.754322
E_{M06-2X}= -2280.039270
G_{tot}= -2279.668740

exo-INT-S_N2 allylsilane A and furan

Charge = 0 Multiplicity = 1

C	-5.148515	-1.870723	-1.748052
C	-4.972595	-2.895664	-0.789426
O	-3.772737	-2.958833	-0.313235
C	-2.978906	-1.886336	-0.928926
C	-3.937724	-1.256228	-1.855442
C	-1.333305	-1.834434	0.991128
C	-2.315710	-0.987652	0.148949
C	-3.261778	-0.178833	1.025521
C	-4.253499	-0.750955	1.721316
O	-0.604989	-0.997859	1.854705
Si	0.968234	-0.457057	1.478747
C	1.402408	0.683094	2.896203
C	0.903932	0.470723	-0.155775
C	2.087221	-1.963271	1.408578
Si	-3.472356	2.418149	-0.326013
C	-3.308616	4.195460	0.280809
C	-2.336402	2.206824	-1.823129
C	-5.267882	2.051223	-0.775146
F	3.450774	-1.016923	-1.312392
C	4.738836	-0.701946	-1.079445
F	5.287771	-1.711106	-0.378915
F	5.364292	-0.631051	-2.266733
S	4.891353	0.903357	-0.154433
O	4.303153	0.590266	1.171304
O	6.351354	1.145858	-0.147585
O	4.097501	1.843115	-0.982489
H	-4.884798	-0.154101	2.371812
H	-4.449596	-1.815685	1.707951
H	-5.425195	0.998047	-1.024896
H	-5.592122	2.653754	-1.629841

H	-5.924662	2.284814	0.069742
H	-3.961914	4.382884	1.139295
H	-2.279429	4.410822	0.587083
H	-3.578060	4.904671	-0.509044
H	-2.302529	1.193339	-2.232181
H	-2.661925	2.871269	-2.630960
H	-1.308800	2.481641	-1.563740
H	-1.711155	-0.297625	-0.446208
H	-1.901846	-2.553981	1.590293
H	1.863339	-2.605198	0.550606
H	1.977395	-2.560863	2.319878
H	3.127456	-1.641837	1.324619
H	0.704587	1.524740	2.956351
H	1.382981	0.154517	3.854742
H	2.410496	1.075439	2.735664
H	0.609390	-0.181544	-0.985421
H	0.183769	1.293688	-0.096559
H	1.880657	0.893909	-0.403110
H	-6.066394	-1.655662	-2.273813
H	-3.678540	-0.433569	-2.507567
H	-5.686616	-3.623074	-0.419252
C	-2.957908	1.289758	1.127761
H	-1.881912	1.426657	1.292193
H	-3.463353	1.700919	2.009250
H	-0.678634	-2.405484	0.315656
H	-2.183658	-2.406418	-1.478690

0 imaginary frequencies

E_{B3LYP}= -2280.118811

G_{B3LYP}= -2279.750913

E_{M06-2X}= -2280.036293

G_{tot}= -2279.665376

exo-TS2-S_N2’ allylsilane A and furan

Charge = 0 Multiplicity = 1

C	5.709418	-0.320657	0.196429
C	5.260411	-1.543548	-0.525215

O	4.584775	-2.302512	0.489224
C	4.096793	-1.387452	1.334512
C	4.950206	-0.211980	1.306779
C	1.754247	-2.398346	0.148640
C	2.304419	-0.978139	0.146503
C	3.029014	-0.412098	-0.904590
C	4.089582	-1.199425	-1.583322
O	0.558369	-2.439014	-0.603865
Si	-0.890922	-1.664223	-0.153976
C	-2.238386	-2.683769	-0.956683
C	-0.894047	0.092227	-0.826962
C	-1.032096	-1.659133	1.718225
Si	3.039228	2.525478	-0.064013
C	2.070679	3.869995	-0.957656
C	2.240033	2.191649	1.608038
C	4.831666	3.085538	0.091668
F	-6.252088	-0.393411	-0.951919
C	-5.201665	0.431481	-0.806693
F	-4.208771	-0.023284	-1.597723
F	-5.559045	1.646623	-1.255141
S	-4.658733	0.510077	0.967371
O	-4.314664	-0.901044	1.272706
O	-3.506395	1.442561	0.925864
O	-5.857568	1.026256	1.665902
H	4.536410	-0.660602	-2.417895
H	3.739914	-2.173723	-1.926995
H	5.424286	2.489893	0.787983
H	4.845443	4.120743	0.450091
H	5.329732	3.067043	-0.883149
H	2.476243	4.054324	-1.957863
H	1.015923	3.596710	-1.063910
H	2.121157	4.809416	-0.397398
H	2.701008	1.375589	2.169028
H	2.319272	3.096182	2.221089
H	1.174272	1.966609	1.496448
H	1.680943	-0.284475	0.704397
H	2.459559	-3.097953	-0.301588
H	-0.286596	-1.015746	2.197922

H	-0.927902	-2.667888	2.132301
H	-2.027210	-1.284882	1.978156
H	-2.115029	-2.722236	-2.043970
H	-2.236056	-3.710128	-0.575919
H	-3.208482	-2.232628	-0.733678
H	-0.164411	0.725277	-0.311937
H	-0.661754	0.103743	-1.897458
H	-1.877359	0.545334	-0.676219
H	6.430123	0.383998	-0.193129
H	4.923548	0.581090	2.038029
H	6.013208	-2.159455	-1.011614
C	2.849027	1.006139	-1.264661
H	1.786183	1.122766	-1.521119
H	3.426307	1.260545	-2.157387
H	1.576252	-2.719092	1.183138
H	3.535661	-1.763482	2.180898

1 imaginary frequency

E_{B3LYP}= -2280.106426

G_{B3LYP}= -2279.735826

E_{M06-2X}= -2280.018943

G_{tot}= -2279.645324

exo-TS2-S_N2 allylsilane A and furan

Charge = 0 Multiplicity = 1

C	-5.213857	-0.625323	-1.385998
C	-5.084099	-1.860314	-0.631338
O	-4.009730	-2.533273	-1.066495
C	-3.112700	-1.519511	-1.550833
C	-4.031549	-0.436554	-2.005848
C	-1.368780	-2.239954	0.185040
C	-2.266145	-1.063328	-0.261720
C	-3.199588	-0.581247	0.799711
C	-4.217479	-1.406442	1.266899
O	-0.596670	-1.865834	1.296532
Si	0.992036	-1.252577	1.180913
C	1.608653	-1.271386	2.946530

C	0.907641	0.503061	0.513111
C	1.980946	-2.401170	0.072817
Si	-3.271879	2.420481	0.194471
C	-2.882240	3.754712	1.464921
C	-1.998398	2.514047	-1.189900
C	-5.031826	2.656055	-0.435325
F	3.178284	0.023989	-1.590787
C	4.505292	0.081431	-1.370239
F	5.000923	-1.159941	-1.523061
F	5.044479	0.864779	-2.319638
S	4.887377	0.740514	0.325609
O	4.369991	-0.317681	1.228093
O	6.360915	0.876245	0.304236
O	4.134658	2.017510	0.367209
H	-4.931254	-0.997681	1.975398
H	-4.074539	-2.477995	1.317452
H	-5.192119	2.208227	-1.418166
H	-5.233273	3.728924	-0.525765
H	-5.767345	2.237881	0.258949
H	-3.599666	3.742855	2.291710
H	-1.878742	3.626249	1.883127
H	-2.927124	4.743093	0.995555
H	-2.116354	1.752373	-1.963941
H	-2.083345	3.491206	-1.678327
H	-0.983486	2.432545	-0.789705
H	-1.622469	-0.244101	-0.586271
H	-1.997062	-3.093371	0.458358
H	1.693371	-2.321191	-0.979920
H	1.853614	-3.442925	0.386209
H	3.039048	-2.140315	0.147995
H	0.985476	-0.645738	3.593867
H	1.615059	-2.286588	3.356124
H	2.630635	-0.882467	2.964345
H	0.566030	0.526871	-0.526857
H	0.219279	1.104695	1.117136
H	1.889867	0.981346	0.544225
H	-6.093128	0.000541	-1.387549
H	-3.736856	0.383976	-2.643010

H	-5.894658	-2.456575	-0.232633
C	-3.067777	0.804115	1.263215
H	-2.017170	0.921066	1.569027
H	-3.692731	0.976809	2.144223
H	-0.750004	-2.543278	-0.670088
H	-2.436331	-1.949642	-2.288050

1 imaginary frequency

E_{B3LYP}= -2280.108984

G_{B3LYP}= -2279.737588

$E_{M06\text{-}2X}$= -2280.021686

G_{tot}= -2279.647271

exo-Product allylsilane A and furan

Charge = 0 Multiplicity = 1

C	-5.153631	1.624125	1.434323
C	-5.057494	2.160794	0.016387
O	-3.887162	2.989711	0.058742
C	-3.001138	2.257103	0.915311
C	-3.931604	1.675419	1.965724
C	-1.341779	1.696674	-0.972961
C	-2.378450	1.123171	0.069273
C	-3.425350	0.366936	-0.672253
C	-4.742488	1.015066	-0.968388
O	-0.646944	0.669363	-1.612376
Si	0.912026	0.126432	-1.146068
C	0.934044	-1.665166	-1.687331
C	1.069439	0.293003	0.718970
C	2.152321	1.172467	-2.083429
Si	-3.569882	-2.286552	0.285026
C	-3.196229	-3.875524	-0.632137
C	-2.414569	-2.003092	1.727540
C	-5.374790	-2.113896	0.742881
F	3.975159	1.568966	0.526331
C	5.136541	0.902929	0.384189
F	5.655812	1.234636	-0.811841
F	5.977199	1.335848	1.338377

S	4.885458	-0.934798	0.506168
O	4.079491	-1.247548	-0.700138
O	6.267603	-1.463020	0.487303
O	4.165301	-1.091115	1.793062
H	-5.532439	0.260247	-1.026524
H	-4.646167	1.434567	-1.981457
H	-5.568578	-1.184742	1.284378
H	-5.652961	-2.947857	1.396639
H	-6.023571	-2.151627	-0.137112
H	-3.845449	-3.996480	-1.503834
H	-2.154724	-3.908482	-0.964032
H	-3.366945	-4.722701	0.041025
H	-2.640293	-1.083557	2.273237
H	-2.530418	-2.838407	2.427309
H	-1.367809	-1.982550	1.411258
H	-1.801986	0.439488	0.695521
H	-1.870239	2.307841	-1.709641
H	2.130467	2.214573	-1.748299
H	1.926842	1.154928	-3.155039
H	3.161578	0.784462	-1.932146
H	0.219352	-2.263945	-1.111922
H	0.687108	-1.765864	-2.749200
H	1.935206	-2.071281	-1.519995
H	0.995916	1.333493	1.051953
H	0.296309	-0.284235	1.236929
H	2.039927	-0.089188	1.045717
H	-6.056226	1.216447	1.870896
H	-3.607196	1.316582	2.934065
H	-5.906720	2.754934	-0.323084
C	-3.152951	-0.921106	-1.154565
H	-2.088880	-1.123491	-1.299569
H	-3.802194	-1.263010	-1.963234
H	-0.687297	2.358696	-0.391853
H	-2.231025	2.932024	1.294177

0 imaginary frequencies

E_{B3LYP}= -2280.136841

G_{B3LYP}= -2279.764320

E_{M06-2X}= -2280.053660

G_{tot}= -2279.678120

endo-TS enolsilane B and furan

Charge = 0 Multiplicity = 1

C	5.269446	2.387988	0.488543
C	4.087878	2.661220	1.245616
C	3.228621	3.305471	0.398433
O	3.806032	3.460332	-0.829188
C	1.222773	0.632797	1.186485
O	0.638603	-0.385842	0.336760
Si	-1.115735	-0.457777	-0.152574
C	-1.473128	1.285308	-0.734535
C	1.801062	0.837430	-0.137675
C	3.029097	0.202613	-0.570465
O	3.642973	-0.553974	0.368930
Si	4.047356	-2.213024	0.221708
C	5.603276	-2.380361	-0.812164
C	3.506182	0.450483	-1.805422
C	4.340549	-2.731702	1.993549
C	2.592014	-3.092876	-0.562061
C	-1.816315	-0.995046	1.493195
C	-0.942779	-1.745712	-1.489713
O	-3.795882	-0.729222	-1.065534
S	-5.098941	-0.696037	-0.345647
O	-6.285716	-0.701642	-1.224780
C	-5.114204	1.007548	0.394502
F	-4.981907	1.942667	-0.564739
O	-5.169104	-1.583817	0.837454
F	-4.098633	1.166263	1.264516
F	-6.261872	1.238149	1.048476
H	2.950178	1.074946	-2.492336
H	4.450651	0.040814	-2.138639
H	5.141668	-2.141048	2.449029
H	4.630350	-3.786487	2.042536
H	3.435132	-2.601072	2.594377
H	1.698412	-2.987904	0.059157

H	2.363909	-2.680979	-1.549879
H	2.808956	-4.159261	-0.683264
H	6.397817	-1.728977	-0.434336
H	5.968414	-3.412678	-0.775471
H	5.425900	-2.130923	-1.862858
H	1.294981	1.504748	-0.821551
H	1.861327	0.196320	1.948002
H	-1.765791	-0.179243	2.220970
H	-1.224520	-1.832502	1.876777
H	-2.857383	-1.309751	1.405069
H	-0.199606	-1.425894	-2.227031
H	-0.610587	-2.697288	-1.064699
H	-1.900587	-1.896559	-1.988759
H	-1.260781	2.032805	0.036462
H	-0.881921	1.524429	-1.624503
H	-2.525480	1.370294	-1.002786
H	6.159297	1.877861	0.825301
H	3.900301	2.413690	2.280000
C	5.042364	2.882033	-0.762645
H	5.629838	2.920537	-1.665814
H	0.520171	1.375927	1.555437
H	2.255157	3.752451	0.524026

1 imaginary frequency

E_{B3LYP}= -2316.024494

G_{B3LYP}= -2315.686155

E_{M06-2X}= -2315.963469

G_{tot}= -2315.622111

endo-INT-S_N2' enolsilane B and furan

Charge = 0 Multiplicity = 1

C	5.659499	1.342109	-0.412179
C	6.013894	1.721923	0.853767
C	5.229194	2.850749	1.151120
O	4.450821	3.220480	0.179417
C	1.312791	-0.070109	1.714482
O	0.545867	-1.158256	1.197142

Si	-1.072799	-1.010361	0.704334
C	-1.200909	0.131602	-0.786605
C	1.745494	0.891307	0.642528
C	2.820772	0.696540	-0.130975
O	3.706590	-0.328324	0.052390
Si	3.589285	-1.944754	-0.520980
C	5.276732	-2.288938	-1.264886
C	3.242829	1.625074	-1.229492
C	3.255644	-3.053791	0.946093
C	2.224230	-2.019540	-1.798860
C	-2.076153	-0.382182	2.163959
C	-1.546912	-2.760853	0.232597
O	-4.369622	-1.125825	-0.793425
S	-5.438018	-0.290196	-0.191818
O	-6.706762	-0.259058	-0.954311
C	-4.792141	1.442004	-0.389518
F	-4.444709	1.679435	-1.667614
O	-5.586957	-0.427527	1.276946
F	-3.707732	1.656889	0.377828
F	-5.726381	2.342451	-0.038478
H	2.521190	2.425915	-1.395891
H	3.371049	1.076419	-2.168444
H	4.014644	-2.920682	1.723792
H	3.270360	-4.105072	0.637805
H	2.271566	-2.828977	1.366030
H	1.271898	-1.745111	-1.340684
H	2.413813	-1.350110	-2.643982
H	2.134525	-3.037922	-2.191562
H	6.074379	-2.121189	-0.533363
H	5.343629	-3.331597	-1.593289
H	5.466882	-1.654027	-2.136827
H	1.126002	1.757395	0.436136
H	2.197093	-0.506937	2.188303
H	-1.814148	0.649406	2.422389
H	-1.893899	-1.007839	3.044795
H	-3.146195	-0.404305	1.940102
H	-0.912148	-3.138420	-0.576167
H	-1.455622	-3.444457	1.082838

H	-2.584808	-2.765496	-0.112600
H	-1.078134	1.183721	-0.514034
H	-0.442421	-0.116491	-1.535958
H	-2.190186	0.015265	-1.236323
H	6.070820	0.532281	-0.996882
H	6.732117	1.264948	1.516980
C	4.618144	2.263776	-0.917882
H	4.958689	2.851052	-1.776924
H	0.748573	0.467146	2.486693
H	5.199079	3.450912	2.053392

0 imaginary frequencies

E_{B3LYP}= -2316.068059

G_{B3LYP}= -2315.722876

E_{M06-2X}= -2316.007959

G_{tot}= -2315.659757

endo-INT-S_N2 enolsilane B and furan

Charge = 0 Multiplicity = 1

C	5.005744	2.695597	0.285868
C	4.202229	2.051837	1.184948
C	2.793220	2.395109	0.897005
O	2.895145	3.379321	-0.178235
C	1.474386	0.336648	1.547938
O	0.816373	-0.820719	1.103117
Si	-0.836408	-0.895746	0.687889
C	-1.109539	-0.024067	-0.954824
C	1.918324	1.214506	0.356902
C	2.706574	0.443591	-0.664659
O	3.712838	-0.255008	-0.078554
Si	4.238960	-1.853886	-0.409707
C	5.553944	-1.784048	-1.745369
C	2.493338	0.525872	-1.983122
C	4.956598	-2.400264	1.229434
C	2.753780	-2.872094	-0.916770
C	-1.810880	-0.122230	2.094977
C	-1.159824	-2.732301	0.529018

O	-4.119029	-1.456145	-0.510048
S	-5.234580	-0.602258	-0.031916
O	-6.514808	-0.790741	-0.750657
C	-4.718093	1.108185	-0.546377
F	-4.419226	1.143101	-1.857854
O	-5.346490	-0.490343	1.442224
F	-3.633060	1.523333	0.133650
F	-5.704715	1.992502	-0.321070
H	1.674297	1.121673	-2.364544
H	3.103888	-0.015492	-2.695739
H	5.748311	-1.719303	1.558273
H	5.387449	-3.403744	1.150085
H	4.182737	-2.423838	2.003137
H	1.961711	-2.761967	-0.171471
H	2.354854	-2.555250	-1.884387
H	3.027494	-3.929789	-0.990996
H	6.368744	-1.111034	-1.459435
H	5.981642	-2.779630	-1.907293
H	5.146402	-1.438104	-2.700407
H	1.045341	1.673203	-0.110039
H	2.356674	0.022104	2.114160
H	-1.641815	0.957421	2.165078
H	-1.520051	-0.573902	3.049928
H	-2.883847	-0.276117	1.952328
H	-0.534219	-3.178370	-0.251134
H	-0.957509	-3.256722	1.468448
H	-2.209046	-2.883673	0.260361
H	-1.074729	1.065849	-0.860122
H	-0.350552	-0.332389	-1.680300
H	-2.096848	-0.290741	-1.339113
H	6.078845	2.643176	0.184049
H	4.507648	1.387747	1.980426
C	4.143626	3.461345	-0.523656
H	4.376931	4.111201	-1.359192
H	0.843732	0.940576	2.214115
H	2.279675	2.890628	1.726676

0 imaginary frequencies

E_{B3LYP}= -2316.067381

G_{B3LYP}= -2315.722809
$E_{M06\text{-}2X}$= -2316.007835
G_{tot}= -2315.660244

endo-TS2-S_N2’ enolsilane B and furan

Charge = 0 Multiplicity = 1

C	-4.979583	-1.987463	-0.023597
C	-4.533999	-2.055079	1.252997
C	-3.253202	-2.718389	1.198216
O	-3.086263	-3.299616	0.007950
C	-1.437716	-0.259055	2.028748
O	-0.736338	0.933475	1.710317
Si	0.863308	0.957086	1.126918
C	0.902086	0.375635	-0.662789
C	-1.734529	-1.079405	0.793733
C	-2.557158	-0.570936	-0.199099
O	-3.297789	0.478513	0.092393
Si	-3.845256	1.862709	-0.797702
C	-5.446252	1.414582	-1.657536
C	-2.908889	-1.367634	-1.397967
C	-4.093816	3.124717	0.551184
C	-2.475089	2.314888	-1.985953
C	1.903800	-0.126226	2.254702
C	1.342480	2.763924	1.232865
O	4.074280	1.442138	-0.297300
S	5.135220	0.418632	-0.127518
O	6.344990	0.633364	-0.953182
C	4.386601	-1.107992	-0.879970
F	3.950513	-0.861107	-2.128849
O	5.390191	0.010913	1.274833
F	3.340548	-1.554068	-0.159810
F	5.290930	-2.100206	-0.944343
H	-2.051277	-1.872765	-1.840087
H	-3.430611	-0.785158	-2.155871
H	-4.849579	2.789967	1.268295
H	-4.425603	4.078761	0.128962

H	-3.158003	3.294999	1.091718
H	-1.559987	2.564857	-1.441008
H	-2.248267	1.506281	-2.687444
H	-2.768446	3.191183	-2.573577
H	-6.184213	1.040635	-0.941080
H	-5.866729	2.308934	-2.130293
H	-5.310829	0.662978	-2.441155
H	-1.036913	-1.857174	0.506448
H	-2.377852	0.037192	2.503314
H	1.634502	-1.183879	2.163165
H	1.763513	0.169186	3.300501
H	2.965204	-0.036014	2.009549
H	0.678008	3.385358	0.622778
H	1.301593	3.131591	2.263127
H	2.363753	2.879677	0.858979
H	0.784754	-0.707474	-0.755391
H	0.110776	0.856772	-1.246466
H	1.868240	0.643987	-1.097124
H	-5.885571	-1.526865	-0.390473
H	-4.987556	-1.657812	2.148900
C	-3.905941	-2.537170	-0.894862
H	-4.193215	-3.151164	-1.745361
H	-0.875278	-0.872155	2.743035
H	-2.679901	-3.125408	2.020731

1 imaginary frequency

E_{B3LYP}= -2316.062224

G_{B3LYP}= -2315.715471

E_{M06-2X}= -2315.999338

G_{tot}= -2315.649566

endo-TS2-S_N2 enolsilane B and furan

Charge = 0 Multiplicity = 1

C	-4.624131	-2.293302	0.114023
C	-4.088354	-2.048428	1.332151
C	-2.644740	-2.408355	1.261763
O	-2.560869	-3.258958	0.103101

C	-1.512552	-0.206271	2.051114
O	-0.810001	0.957631	1.689770
Si	0.812551	0.996800	1.169703
C	0.860825	0.675861	-0.683344
C	-1.730500	-1.137748	0.849995
C	-2.426986	-0.503450	-0.302763
O	-3.252074	0.455622	0.047927
Si	-4.060376	1.705702	-0.857262
C	-5.527857	0.938021	-1.728223
C	-2.450545	-1.118039	-1.542380
C	-4.571591	2.879238	0.497620
C	-2.789369	2.435167	-2.017496
C	1.766858	-0.277603	2.165978
C	1.370576	2.744665	1.538138
O	4.067054	1.517790	-0.137271
S	5.064892	0.421813	-0.064199
O	6.313465	0.665771	-0.820818
C	4.262960	-0.956193	-1.021078
F	3.875521	-0.531938	-2.237635
O	5.246658	-0.167583	1.284055
F	3.175494	-1.432390	-0.386006
F	5.117607	-1.979440	-1.190123
H	-1.635265	-1.772307	-1.821357
H	-3.047111	-0.708132	-2.348313
H	-5.246688	2.389970	1.206385
H	-5.091154	3.746720	0.078043
H	-3.696356	3.238254	1.047260
H	-1.931642	2.814079	-1.453267
H	-2.424511	1.710573	-2.750445
H	-3.229261	3.274339	-2.566459
H	-6.198251	0.458574	-1.008328
H	-6.096029	1.721354	-2.241599
H	-5.237809	0.195184	-2.476543
H	-0.788556	-1.583858	0.528803
H	-2.481340	0.102234	2.453725
H	1.466146	-1.302622	1.924483
H	1.602583	-0.121208	3.238087
H	2.838840	-0.197165	1.967528

H	0.746138	3.478549	1.017566
H	1.328800	2.960909	2.610413
H	2.402299	2.863739	1.195327
H	0.709986	-0.376532	-0.938645
H	0.094426	1.266486	-1.196348
H	1.841403	0.969465	-1.065745
H	-5.615664	-2.064685	-0.247196
H	-4.562617	-1.584500	2.185048
C	-3.566435	-2.870699	-0.683326
H	-3.653258	-3.369455	-1.639557
H	-0.985498	-0.772195	2.830630
H	-2.198748	-2.907260	2.120264

1 imaginary frequency

E_{B3LYP}= -2316.063118

G_{B3LYP}= -2315.715937

E_{M06-2X}= -2316.000811

G_{tot}= -2315.650611

endo-Product enolsilane B and furan

Charge = 0 Multiplicity = 1

C	4.665309	2.308092	-0.132222
C	4.104838	2.402431	1.075676
C	2.625446	2.667621	0.865820
O	2.573478	3.237493	-0.438265
C	1.658858	0.624280	2.158424
O	0.873109	-0.541726	2.134402
Si	-0.735791	-0.650983	1.597420
C	-0.705271	-1.018954	-0.248401
C	1.821456	1.307890	0.793528
C	2.491261	0.477994	-0.253994
O	2.758152	-0.719996	0.021969
Si	3.457884	-2.152522	-0.828933
C	5.282650	-1.787398	-0.921674
C	2.915581	1.126783	-1.529308
C	2.994038	-3.485880	0.378555
C	2.569451	-2.273089	-2.463371

C	-1.617436	0.948644	2.037101
C	-1.446777	-2.113612	2.522860
O	-3.963006	-1.329523	0.369004
S	-4.862659	-0.219319	-0.031631
O	-6.085789	-0.638366	-0.751915
C	-3.893152	0.669025	-1.346387
F	-3.498732	-0.184881	-2.308870
O	-5.061447	0.825031	1.001532
F	-2.790941	1.254089	-0.840147
F	-4.637428	1.628575	-1.921337
H	2.030689	1.327115	-2.142861
H	3.601386	0.492244	-2.089953
H	3.420368	-3.285570	1.365481
H	3.372669	-4.452337	0.030633
H	1.907405	-3.561701	0.478895
H	1.485795	-2.211779	-2.328126
H	2.876982	-1.506920	-3.178921
H	2.794810	-3.250720	-2.903697
H	5.677423	-1.530854	0.065555
H	5.806851	-2.682367	-1.273476
H	5.511981	-0.971648	-1.612567
H	0.841162	1.591704	0.391299
H	2.647892	0.351324	2.542353
H	-1.251640	1.807500	1.465002
H	-1.481667	1.171179	3.101524
H	-2.688866	0.860487	1.838619
H	-0.873807	-3.025711	2.324886
H	-1.449361	-1.940246	3.603699
H	-2.476632	-2.272608	2.190552
H	-0.385252	-0.169810	-0.859713
H	-0.032137	-1.860626	-0.443022
H	-1.708822	-1.298733	-0.575491
H	5.687135	2.050400	-0.377628
H	4.568228	2.246122	2.040660
C	3.564294	2.509404	-1.157273
H	3.835334	3.048957	-2.064341
H	1.235875	1.373658	2.841448
H	2.144096	3.336363	1.579845

0 imaginary frequencies

E_{B3LYP}= -2316.078306

G_{B3LYP}= -2315.728633

E_{M06-2X}= -2316.022110

G_{tot}= -2315.669418

exo-TS enolsilane B and furan

Charge = 0 Multiplicity = 1

C	3.656935	3.832080	-0.793332
C	4.824282	3.136868	-0.672582
O	4.871137	2.492765	0.531556
C	3.700878	2.763656	1.174668
C	2.921272	3.588491	0.410124
C	1.236007	0.459880	1.304916
C	1.816916	0.782342	0.005212
C	3.060561	0.215403	-0.473993
C	3.512175	0.556275	-1.696890
O	0.675699	-0.503151	0.378383
Si	-1.072001	-0.557302	-0.125752
C	-0.892627	-1.766691	-1.533032
C	-1.440129	1.209470	-0.614982
C	-1.790980	-1.193850	1.477990
O	3.706435	-0.585024	0.408861
Si	4.130390	-2.225468	0.147138
C	2.679610	-3.075502	-0.677447
C	5.674684	-2.303947	-0.914196
C	4.452131	-2.857046	1.876947
F	-4.036372	1.046430	1.303508
C	-5.130841	0.374565	0.896583
F	-5.263334	-0.710334	1.680609
F	-6.197466	1.164297	1.090773
S	-4.985319	-0.116657	-0.888882
O	-3.808037	-1.027891	-0.887495
O	-6.275367	-0.777573	-1.172198
O	-4.758730	1.175368	-1.577162
H	2.924597	1.199521	-2.337647

H	4.465651	0.201317	-2.066770
H	5.249676	-2.285852	2.362485
H	4.758233	-3.908060	1.851551
H	3.552041	-2.781426	2.495000
H	1.789549	-3.022192	-0.044807
H	2.437153	-2.608388	-1.636855
H	2.911931	-4.129049	-0.864938
H	6.469745	-1.675834	-0.499937
H	6.046888	-3.333574	-0.956459
H	5.482506	-1.980907	-1.942060
H	1.296425	1.489877	-0.623987
H	1.878438	-0.022292	2.035406
H	-1.787731	-0.416703	2.247872
H	-1.194614	-2.038659	1.836602
H	-2.819046	-1.522616	1.328395
H	-0.157246	-1.397307	-2.254780
H	-0.549844	-2.738660	-1.166635
H	-1.852411	-1.895681	-2.034956
H	-1.232464	1.912239	0.198654
H	-0.836172	1.494625	-1.482954
H	-2.489219	1.323574	-0.893131
H	3.352463	4.433279	-1.636950
H	1.950423	3.979336	0.677695
H	5.689259	3.017419	-1.304847
H	0.517223	1.158501	1.726192
H	3.594422	2.341362	2.161080

1 imaginary frequency

E_{B3LYP}= -2316.024610

G_{B3LYP}= -2315.686144

E_{M06-2X}= -2315.963498

G_{tot}= -2315.622013

exo-INT-S_N2’ enolsilane B and furan

Charge = 0 Multiplicity = 1

C	-5.068767	-0.233338	1.507863
C	-4.001503	-1.246326	1.658385

O	-4.499029	-2.381470	0.873992
C	-5.609219	-2.015644	0.308989
C	-6.031947	-0.722041	0.669149
C	-1.746034	-2.165860	-1.556100
C	-2.429716	-0.821697	-1.427310
C	-2.779775	-0.238716	-0.273499
C	-2.617121	-0.844994	1.087799
O	-0.637224	-2.363811	-0.688329
Si	0.806952	-1.468479	-0.801074
C	0.743283	-0.083043	0.465948
C	0.975362	-0.767768	-2.536045
C	2.164186	-2.704843	-0.422665
O	-3.461283	0.951355	-0.246074
Si	-2.766805	2.516897	-0.215618
C	-1.920634	2.785406	1.437964
C	-1.556237	2.662845	-1.638014
C	-4.247247	3.641694	-0.413646
F	3.644366	-0.683449	1.613147
C	4.826303	-0.255385	1.132364
F	5.489953	0.334463	2.141177
F	5.527793	-1.336850	0.747151
S	4.599107	0.921774	-0.288051
O	3.677103	1.943581	0.264534
O	5.976968	1.393929	-0.552317
O	4.017944	0.063720	-1.349828
H	-2.195764	-0.120333	1.789721
H	-1.974825	-1.724401	1.066595
H	-4.756492	3.459163	-1.364910
H	-3.935503	4.691107	-0.392206
H	-4.968144	3.488806	0.395905
H	-2.616731	2.634727	2.269628
H	-1.064467	2.119371	1.580017
H	-1.547475	3.813880	1.497546
H	-2.052918	2.493483	-2.598428
H	-1.120305	3.667574	-1.654655
H	-0.734981	1.946159	-1.548745
H	-2.652501	-0.284692	-2.344878
H	-2.464078	-2.965845	-1.332437

H	2.123721	-3.546351	-1.122351
H	2.071908	-3.102026	0.593439
H	3.139736	-2.222022	-0.513433
H	-0.098982	0.578102	0.248288
H	0.610676	-0.479328	1.478310
H	1.658083	0.514169	0.450524
H	0.975830	-1.555809	-3.296798
H	0.173230	-0.062844	-2.777856
H	1.930232	-0.236705	-2.591263
H	-5.055336	0.722860	2.010793
H	-6.933661	-0.239199	0.325040
H	-3.892554	-1.609247	2.684825
H	-1.443019	-2.296223	-2.601452
H	-6.083915	-2.738268	-0.344946

0 imaginary frequencies

E_{B3LYP}= -2316.067060

G_{B3LYP}= -2315.722025

E_{M06-2X}= -2316.006581

G_{tot}= -2315.658527

exo-INT-S_N2 enolsilane B and furan

Charge = 0 Multiplicity = 1

C	-5.386748	-0.976710	-1.407026
C	-5.397403	-2.030983	-0.472940
O	-4.268351	-2.668297	-0.393127
C	-3.300297	-1.961610	-1.231081
C	-4.115900	-0.933165	-1.909853
C	-1.481894	-2.503563	0.471225
C	-2.194053	-1.373481	-0.291499
C	-2.799242	-0.270387	0.536135
C	-3.194610	-0.401659	1.808474
O	-0.465980	-2.025744	1.314944
Si	0.908153	-1.158873	0.806410
C	2.167140	-1.459054	2.157329
C	0.506580	0.672855	0.694194
C	1.466321	-1.827953	-0.858757

O	-2.964431	0.829355	-0.242812
Si	-3.546455	2.397249	0.146252
C	-5.352047	2.275118	0.642593
C	-2.474336	3.116286	1.503258
C	-3.342092	3.312077	-1.470723
F	5.927933	-0.584208	-1.585570
C	5.416276	-0.346563	-0.365668
F	4.642416	-1.398483	-0.032122
F	6.433364	-0.290905	0.510694
S	4.439653	1.233174	-0.351155
O	3.383911	0.986053	-1.363271
O	3.960167	1.318748	1.049792
O	5.442053	2.254788	-0.731570
H	-3.651033	0.410092	2.359129
H	-3.004810	-1.321558	2.343737
H	-2.291612	3.337642	-1.776133
H	-3.691847	4.345160	-1.373955
H	-3.919670	2.834125	-2.268396
H	-5.960273	1.903874	-0.188015
H	-5.508104	1.613216	1.498650
H	-5.729021	3.267297	0.913296
H	-1.421669	3.104475	1.205486
H	-2.762947	4.155869	1.692292
H	-2.564931	2.566562	2.443940
H	-1.484906	-0.908226	-0.983067
H	-2.204226	-3.048577	1.086823
H	0.693813	-1.728730	-1.629367
H	1.744375	-2.885154	-0.793711
H	2.337151	-1.255757	-1.188785
H	3.045488	-0.833970	1.977029
H	1.752280	-1.195161	3.136093
H	2.480007	-2.507465	2.190752
H	-0.217718	0.904813	-0.092220
H	0.098391	1.037839	1.642275
H	1.434162	1.211827	0.481445
H	-6.228654	-0.346758	-1.649239
H	-3.725100	-0.264862	-2.663561
H	-6.207479	-2.393643	0.149156

H	-1.085419	-3.210304	-0.271470
H	-2.862995	-2.704792	-1.903605

0 imaginary frequencies

E_{B3LYP}= -2316.065808

G_{B3LYP}= -2315.722094

E_{M06-2X}= -2316.006508

G_{tot}= -2315.659775

exo-TS2-S_N2' enolsilane B and furan

Charge = 0 Multiplicity = 1

C	-5.454362	-0.758514	0.704724
C	-4.331846	-1.441188	1.404981
O	-4.099293	-2.616929	0.608648
C	-4.490906	-2.278829	-0.622247
C	-5.495246	-1.246210	-0.557430
C	-1.534760	-2.387685	-0.856906
C	-2.506115	-1.243919	-1.132432
C	-2.829871	-0.279073	-0.189446
C	-2.958265	-0.602585	1.251294
O	-0.565362	-2.046345	0.116545
Si	0.832419	-1.136004	-0.250940
C	1.223942	-0.280854	1.367511
C	0.454588	0.099681	-1.617501
C	2.153754	-2.338809	-0.824021
O	-3.342828	0.857521	-0.629536
Si	-3.311598	2.480770	-0.004014
C	-4.742510	2.709567	1.180624
C	-1.652679	2.736571	0.823054
C	-3.519138	3.501359	-1.550121
F	4.277026	-1.061239	1.183904
C	5.264010	-0.438829	0.513670
F	6.261979	-0.200320	1.381424
F	5.721265	-1.286989	-0.425339
S	4.670107	1.142805	-0.261430
O	4.055310	1.867077	0.877119
O	5.915425	1.741657	-0.792008

O	3.712833	0.676384	-1.295204
H	-3.038049	0.277476	1.886660
H	-2.154120	-1.250595	1.594041
H	-2.691793	3.330519	-2.245111
H	-3.539783	4.567096	-1.300106
H	-4.455280	3.254922	-2.060428
H	-5.698828	2.496808	0.693555
H	-4.658513	2.087223	2.076376
H	-4.764542	3.754168	1.510640
H	-0.827723	2.517157	0.139329
H	-1.564198	3.785218	1.127152
H	-1.524740	2.124141	1.719980
H	-2.620820	-0.928667	-2.165624
H	-2.053956	-3.277881	-0.490597
H	1.791302	-2.933070	-1.670518
H	2.440278	-3.027499	-0.023242
H	3.043553	-1.790992	-1.142608
H	0.366476	0.321697	1.685923
H	1.435899	-1.009921	2.156369
H	2.090794	0.377595	1.271319
H	0.152002	-0.392886	-2.547963
H	-0.332642	0.805341	-1.338684
H	1.372814	0.661099	-1.813813
H	-6.030340	0.053055	1.124366
H	-6.099758	-0.916921	-1.389364
H	-4.464407	-1.719928	2.447826
H	-1.073622	-2.658727	-1.814880
H	-4.347899	-3.020110	-1.397311

1 imaginary frequency

E_{B3LYP}= -2316.058296

G_{B3LYP}= -2315.710809

$E_{M06\text{-}2X}$= -2315.994374

G_{tot}= -2315.643868

exo-TS2-S_N2 enolsilane B and furan

Charge = 0 Multiplicity = 1

C	5.288245	-0.865725	1.298237
C	5.222367	-1.709505	0.130837
O	4.328654	-2.678923	0.307755
C	3.394435	-2.156144	1.275755
C	4.198240	-1.165510	2.042406
C	1.531276	-2.435841	-0.484565
C	2.276678	-1.413330	0.390709
C	2.976342	-0.318370	-0.343191
C	3.883036	-0.574139	-1.350623
O	0.596799	-1.811284	-1.327383
Si	-0.833847	-1.047446	-0.803213
C	-2.005461	-1.198593	-2.253914
C	-0.481478	0.761598	-0.442825
C	-1.466823	-1.928252	0.729677
O	2.866614	0.844055	0.267987
Si	3.353813	2.461149	-0.167045
C	5.203948	2.588977	0.086321
C	2.833255	2.771761	-1.936373
C	2.389016	3.484041	1.056311
F	-6.021425	-0.829263	1.323962
C	-5.423591	-0.422615	0.191134
F	-4.620944	-1.419863	-0.231290
F	-6.374153	-0.237949	-0.740403
S	-4.456629	1.137639	0.473685
O	-3.473750	0.739671	1.510511
O	-3.879982	1.422178	-0.863082
O	-5.487352	2.099828	0.926342
H	4.453535	0.230453	-1.796821
H	3.798071	-1.485896	-1.925555
H	1.313601	3.334942	0.921281
H	2.605899	4.548366	0.919538
H	2.647838	3.214330	2.084667
H	5.471748	2.378832	1.126077
H	5.766315	1.909982	-0.560976
H	5.530388	3.609346	-0.141993
H	1.762972	2.586150	-2.063923
H	3.022908	3.822195	-2.182596
H	3.376562	2.157345	-2.658660

H	1.586674	-0.957266	1.102953
H	2.245152	-2.979777	-1.109729
H	-0.747275	-1.896658	1.555372
H	-1.701759	-2.978003	0.524696
H	-2.376506	-1.423825	1.065769
H	-2.912419	-0.625115	-2.045860
H	-1.547139	-0.798497	-3.164686
H	-2.281963	-2.241124	-2.440637
H	0.196096	0.901056	0.404152
H	-0.034395	1.246946	-1.316961
H	-1.429624	1.258127	-0.218691
H	6.045617	-0.117874	1.477556
H	3.876935	-0.710406	2.968523
H	5.988720	-1.883425	-0.612617
H	1.054092	-3.166476	0.182345
H	2.938422	-2.978440	1.824582

1 imaginary frequency

E_{B3LYP}= -2316.063231

G_{B3LYP}= -2315.717861

E_{M06-2X}= -2315.999572

G_{tot}= -2315.651183

exo-Product enolsilane B and furan

Charge = 0 Multiplicity = 1

C	5.278248	1.716539	-1.354667
C	5.174835	1.621802	0.157573
O	4.287473	2.689643	0.493491
C	3.337155	2.681834	-0.577927
C	4.186366	2.346878	-1.791232
C	1.531347	1.781162	1.017876
C	2.343548	1.515679	-0.296820
C	3.118492	0.258872	-0.107373
C	4.455546	0.296778	0.558333
O	0.927821	0.611190	1.500895
Si	-0.684195	0.166128	1.143309
C	-0.882009	-1.475544	2.019668

C	-0.859629	0.020559	-0.721179
C	-1.808870	1.480972	1.870918
O	2.581080	-0.808909	-0.506699
Si	2.904127	-2.572608	-0.259522
C	4.441594	-2.959186	-1.241096
C	3.038984	-2.747001	1.591123
C	1.359206	-3.295574	-0.996356
F	-3.596706	1.552379	-0.791242
C	-4.818315	1.045862	-0.540219
F	-5.288400	1.640938	0.571282
F	-5.620294	1.382602	-1.564223
S	-4.759752	-0.799415	-0.323489
O	-3.977858	-0.968489	0.926467
O	-6.189182	-1.168364	-0.219560
O	-4.074534	-1.262820	-1.554279
H	5.043231	-0.588264	0.312167
H	4.284693	0.305987	1.642933
H	0.465099	-2.958990	-0.465957
H	1.399303	-4.388067	-0.935883
H	1.262266	-3.017584	-2.049882
H	4.353561	-2.581756	-2.264124
H	5.353439	-2.554734	-0.794948
H	4.552390	-4.047831	-1.294770
H	2.247198	-2.176374	2.085448
H	2.916954	-3.800667	1.862575
H	4.005486	-2.410960	1.975015
H	1.645991	1.364036	-1.122855
H	2.229716	2.159915	1.768864
H	-1.714820	2.439502	1.350425
H	-1.573330	1.641729	2.928340
H	-2.848598	1.156307	1.790953
H	-0.188446	-2.232110	1.640641
H	-0.707794	-1.367979	3.095363
H	-1.903829	-1.832710	1.866477
H	-0.806832	1.003348	-1.202481
H	-0.063065	-0.603747	-1.136114
H	-1.822964	-0.421346	-0.988588
H	6.067113	1.262411	-1.939813

H	3.880695	2.523746	-2.814106
H	6.105779	1.733612	0.713340
H	0.809542	2.575145	0.786991
H	2.815411	3.639666	-0.603798

0 imaginary frequencies

E_{B3LYP}= -2316.083728

G_{B3LYP}= -2315.734159

E_{M06-2X}= -2316.025484

G_{tot}= -2315.672896

endo-TS enolsilane D

Charge = 0 Multiplicity = 1

C	5.231344	1.855468	-0.355404
C	4.273781	2.219898	0.640853
C	3.260373	2.885056	-0.001260
O	3.548890	2.955225	-1.343165
C	2.072253	3.664892	0.466543
C	1.412071	3.125610	1.747371
C	0.497752	1.906850	1.534715
C	1.259770	0.684158	1.088600
O	0.503545	-0.452331	0.560590
Si	-1.235529	-0.698943	0.143441
C	-1.602312	0.501687	-1.243510
C	1.668251	0.532686	-0.304939
C	2.788985	-0.267782	-0.741695
O	3.448048	-0.905594	0.248212
Si	4.078829	-2.498100	0.219959
C	5.737087	-2.487684	-0.656218
C	3.129135	-0.254174	-2.046150
C	4.267182	-2.904481	2.034392
C	2.827659	-3.594916	-0.640872
C	-2.070073	-0.418173	1.790069
C	-1.107756	-2.476034	-0.408549
O	-4.054434	-1.399199	-0.675574
S	-5.338533	-0.926532	-0.092877

O	-6.541292	-1.287393	-0.872229
C	-5.231572	0.916804	-0.299199
F	-5.046525	1.240608	-1.591931
O	-5.448843	-1.101411	1.373922
F	-4.200561	1.426759	0.402831
F	-6.355443	1.514108	0.125294
H	2.521784	0.284648	-2.761434
H	4.006780	-0.769260	-2.413574
H	4.937683	-2.193894	2.528031
H	4.685971	-3.907918	2.163292
H	3.299382	-2.872523	2.544341
H	1.865844	-3.568812	-0.119813
H	2.660252	-3.283294	-1.676143
H	3.179405	-4.631819	-0.655500
H	6.391013	-1.714372	-0.240486
H	6.235563	-3.454655	-0.526854
H	5.638725	-2.308631	-1.731226
H	1.118042	1.086224	-1.054678
H	1.979335	0.312227	1.814655
H	-2.185335	0.646115	2.007744
H	-1.474895	-0.874840	2.587386
H	-3.065510	-0.867842	1.790778
H	-0.391840	-2.573779	-1.230510
H	-0.771659	-3.112310	0.415942
H	-2.086541	-2.822905	-0.745072
H	-1.612791	1.544593	-0.915739
H	-0.873771	0.393013	-2.053494
H	-2.589796	0.267639	-1.644052
H	6.148350	1.304174	-0.210188
H	4.332654	2.017413	1.699806
H	-0.269236	2.157105	0.795499
H	-0.014034	1.670375	2.471542
H	2.176836	2.896291	2.497600
H	0.797115	3.921840	2.175185
H	1.336451	3.715001	-0.344084
H	2.387887	4.700536	0.647769
C	4.740708	2.315971	-1.541250
H	5.094277	2.280095	-2.559061

1 imaginary frequency

E_{B3LYP}= -2432.799528

G_{B3LYP}= -2432.396705

E_{M06-2X}= -2432.707814

G_{tot}= -2432.301972

endo-INT-S_N2' enolsilane D

Charge = 0 Multiplicity = 1

C	-5.248497	-1.608618	-0.785774
C	-4.764188	-2.040316	0.404789
C	-3.536629	-2.725288	0.127911
O	-3.347744	-2.853905	-1.165153
C	-2.608396	-3.390793	1.068813
C	-2.168989	-2.521440	2.274623
C	-1.071013	-1.480662	2.005498
C	-1.509191	-0.233533	1.222992
O	-0.550408	0.817101	1.309261
Si	1.076186	0.844470	0.822594
C	1.264810	0.129512	-0.907687
C	-1.845917	-0.585239	-0.197564
C	-2.880071	-0.067327	-0.896254
Si	-3.510252	2.526696	-0.197665
C	-2.063356	3.007774	-1.283723
C	-3.343318	-0.675348	-2.180336
C	-5.129890	3.269850	-0.771802
C	-3.185939	2.900482	1.606089
C	2.123526	-0.068928	2.088843
C	1.493001	2.672160	0.833072
O	4.369924	1.464561	-0.551275
S	5.477480	0.558398	-0.158262
O	6.742653	0.765186	-0.898849
C	4.908986	-1.103731	-0.767129
F	4.578659	-1.046990	-2.070331
O	5.626461	0.350385	1.302100
F	3.830686	-1.538239	-0.089123
F	5.879883	-2.022091	-0.623667

H	-2.521860	-1.045837	-2.793872
H	-3.953662	0.019195	-2.758756
H	-5.321239	3.034886	-1.823559
H	-5.111534	4.359667	-0.665043
H	-5.967971	2.889391	-0.178787
H	-3.974305	2.490952	2.245906
H	-2.224167	2.483000	1.916532
H	-3.157255	3.984765	1.761942
H	-2.246163	2.763488	-2.334924
H	-1.879450	4.084946	-1.212835
H	-1.158472	2.488403	-0.958755
H	-1.209008	-1.300163	-0.706676
H	-2.415500	0.162616	1.694351
H	1.976703	-1.151988	2.042532
H	1.863095	0.264533	3.099537
H	3.186374	0.128721	1.923128
H	0.877378	3.229726	0.119936
H	1.341568	3.109426	1.825481
H	2.543245	2.793960	0.552553
H	1.168568	-0.960307	-0.923783
H	0.520548	0.551121	-1.590667
H	2.262229	0.380979	-1.277132
H	-6.145048	-1.032429	-0.966330
H	-0.227349	-1.960821	1.495209
H	-0.696870	-1.133768	2.973679
H	-3.045068	-2.035680	2.719277
H	-1.794940	-3.218267	3.028995
H	-1.746796	-3.768184	0.513596
H	-3.154531	-4.261579	1.458227
C	-4.251805	-1.932283	-1.832441
H	-4.621198	-2.399443	-2.744318
H	-5.179481	-1.891201	1.389908
O	-3.747435	0.831351	-0.377785

0 imaginary frequencies

E_{B3LYP}= -2432.846728

G_{B3LYP}= -2432.436203

E_{M06-2X}= -2432.755195

G_{tot}= -2432.341651

endo-INT-S_N2 enolsilane D

Charge = 0 Multiplicity = 1

C	-5.204086	-2.047609	-0.650009
C	-4.329679	-1.800470	0.368963
C	-2.965198	-2.213682	-0.037206
O	-3.193509	-2.797408	-1.373411
C	-2.300310	-3.298423	0.827657
C	-1.792606	-2.716967	2.150538
C	-0.823884	-1.556109	1.909362
C	-1.497926	-0.455454	1.090873
O	-0.701692	0.685515	0.870169
Si	0.941803	0.802914	0.437773
C	1.322368	-0.346241	-1.001702
C	-2.001296	-0.990960	-0.273076
C	-2.696287	0.075528	-1.073291
Si	-3.815819	2.359612	0.085239
C	-2.711624	3.414517	-0.996639
C	-2.464214	0.291549	-2.372629
C	-5.633349	2.738781	-0.165055
C	-3.335601	2.505234	1.889856
C	1.992123	0.454662	1.955928
C	1.123710	2.590080	-0.091466
O	4.205974	1.400142	-0.837564
S	5.391741	0.767263	-0.208343
O	6.652295	0.911508	-0.970887
C	5.030655	-1.051391	-0.345825
F	4.752696	-1.387577	-1.619162
O	5.508890	0.978520	1.254220
F	3.975831	-1.405993	0.411379
F	6.086232	-1.779780	0.055421
H	-1.703540	-0.276490	-2.893235
H	-3.006462	1.049462	-2.924622
H	-5.916052	2.641198	-1.218009
H	-5.859561	3.762056	0.153416
H	-6.261146	2.059456	0.420882

H	-3.944240	1.847366	2.518994
H	-2.282706	2.241824	2.024101
H	-3.483280	3.532912	2.239539
H	-3.019530	3.398988	-2.045831
H	-2.749424	4.452633	-0.648674
H	-1.674570	3.076035	-0.935696
H	-1.156774	-1.388016	-0.838251
H	-2.373783	-0.107320	1.650144
H	1.982235	-0.602770	2.233973
H	1.615252	1.032207	2.807426
H	3.032188	0.740555	1.777131
H	0.560836	2.794158	-1.007863
H	0.772840	3.277824	0.684682
H	2.180735	2.790162	-0.288881
H	1.286051	-1.405570	-0.729557
H	0.623344	-0.172469	-1.826281
H	2.331703	-0.128034	-1.358403
H	-6.262927	-1.843237	-0.692906
H	0.063806	-1.923095	1.381524
H	-0.487519	-1.133248	2.859575
H	-2.636468	-2.380740	2.764640
H	-1.299157	-3.515240	2.711150
H	-1.462262	-3.693565	0.245664
H	-3.009257	-4.113726	0.990402
C	-4.439410	-2.627455	-1.682564
H	-4.751536	-2.963418	-2.665058
H	-4.561049	-1.370954	1.333051
O	-3.666022	0.713733	-0.362872

0 imaginary frequencies

E_{B3LYP}= -2432.840994

G_{B3LYP}= -2432.431815

E_{M06-2X}= -2432.750143

G_{tot}= -2432.337945

endo-TS2-S_N2’ enolsilane D

Charge = 0 Multiplicity = 1

C	-5.077862	-1.300782	-1.026959
C	-4.606624	-1.778988	0.138499
C	-3.309991	-2.413620	-0.147944
O	-3.247349	-2.610736	-1.494730
C	-2.669229	-3.472017	0.703422
C	-2.303176	-2.999497	2.119389
C	-1.199160	-1.934901	2.146534
C	-1.617860	-0.650116	1.429739
O	-0.688827	0.410958	1.576879
Si	0.924218	0.564237	1.066283
C	1.080860	0.208534	-0.775741
C	-1.907007	-0.932886	-0.031625
C	-2.618211	-0.000530	-0.802243
Si	-3.204398	2.686812	-0.142319
C	-1.839258	3.182727	-1.318332
C	-3.006220	-0.317672	-2.192094
C	-4.896884	3.254504	-0.688955
C	-2.824004	3.076590	1.639138
C	2.029733	-0.555076	2.093484
C	1.292853	2.365681	1.428192
O	4.133166	1.515996	-0.224230
S	5.280618	0.585353	-0.081959
O	6.498998	0.983333	-0.822570
C	4.717712	-0.932775	-0.996034
F	4.317102	-0.618535	-2.241693
O	5.511128	0.084982	1.294304
F	3.686725	-1.532948	-0.372596
F	5.714682	-1.828570	-1.093184
H	-2.177899	-0.676197	-2.802076
H	-3.517219	0.513163	-2.677396
H	-5.097385	2.970317	-1.726509
H	-4.971293	4.344679	-0.615366
H	-5.676872	2.820735	-0.055503
H	-3.651059	2.777928	2.290812
H	-1.925469	2.533430	1.945727
H	-2.654607	4.150009	1.773444
H	-2.044635	2.859899	-2.343502
H	-1.738747	4.273170	-1.325903

H	-0.878819	2.761836	-1.008012
H	-1.180273	-1.530473	-0.571427
H	-2.547209	-0.287653	1.884978
H	1.921077	-1.608527	1.819357
H	1.785298	-0.453640	3.156448
H	3.080305	-0.285497	1.951947
H	0.621972	3.034003	0.878873
H	1.184106	2.584067	2.495636
H	2.321996	2.582986	1.128668
H	1.029525	-0.860523	-1.002916
H	0.306487	0.720907	-1.355292
H	2.057013	0.571245	-1.107455
H	-5.980269	-0.731071	-1.199003
H	-0.289583	-2.335134	1.683999
H	-0.950048	-1.685184	3.181792
H	-3.198735	-2.617883	2.624738
H	-1.978504	-3.873556	2.689911
H	-1.785198	-3.848074	0.180407
H	-3.388189	-4.298621	0.762594
C	-4.033844	-1.563247	-2.063388
H	-4.363272	-1.842838	-3.062094
H	-5.040126	-1.688577	1.123950
O	-3.279273	0.946671	-0.193179

1 imaginary frequency

E_{B3LYP}= -2432.839995

G_{B3LYP}= -2432.428864

E_{M06-2X}= -2432.747403

G_{tot}= -2432.333253

endo-TS2-S_N2 enolsilane D

Charge = 0 Multiplicity = 1

C	-4.813666	-1.158511	-1.256107
C	-4.358098	-1.674822	-0.091963
C	-2.998309	-2.241593	-0.338160
O	-2.952513	-2.356414	-1.783629
C	-2.607568	-3.554816	0.321212

C	-2.338581	-3.367849	1.819796
C	-1.245579	-2.323155	2.069195
C	-1.626396	-0.969340	1.467112
O	-0.697207	0.051410	1.748076
Si	0.913862	0.310996	1.282853
C	1.038722	0.606224	-0.571570
C	-1.864172	-1.105046	-0.049418
C	-2.327624	0.141453	-0.714907
Si	-3.463644	2.612126	-0.049682
C	-1.962550	3.536054	-0.673139
C	-2.314280	0.262010	-2.090060
C	-4.946180	2.727305	-1.186154
C	-3.885883	3.023402	1.718973
C	2.000378	-1.120846	1.830457
C	1.356139	1.880269	2.205474
O	4.166253	1.550685	0.283838
S	5.264582	0.571812	0.088596
O	6.504069	1.136122	-0.491522
C	4.628839	-0.520625	-1.275100
F	4.239241	0.214271	-2.332795
O	5.465143	-0.373927	1.212436
F	3.573140	-1.252300	-0.872449
F	5.582782	-1.372126	-1.688539
H	-1.602252	-0.315681	-2.664008
H	-2.747798	1.129595	-2.572270
H	-4.718904	2.416042	-2.209753
H	-5.300245	3.763123	-1.224552
H	-5.767625	2.107664	-0.813248
H	-4.705430	2.395999	2.082608
H	-3.018559	2.866369	2.367249
H	-4.194399	4.069980	1.807915
H	-1.681709	3.246829	-1.689423
H	-2.168694	4.611584	-0.676645
H	-1.104758	3.360291	-0.016663
H	-0.974158	-1.495274	-0.545375
H	-2.570932	-0.644914	1.919085
H	1.855944	-2.008908	1.207485
H	1.783208	-1.393156	2.868692

H	3.055349	-0.840271	1.758568
H	0.694822	2.707097	1.924578
H	1.284446	1.742368	3.289201
H	2.383816	2.158193	1.954067
H	0.936892	-0.309007	-1.161860
H	0.288206	1.324597	-0.914364
H	2.029579	1.020855	-0.774462
H	-5.713362	-0.589298	-1.437180
H	-0.301355	-2.665394	1.632905
H	-1.076833	-2.189529	3.141427
H	-3.262374	-3.070682	2.331206
H	-2.045098	-4.330533	2.246540
H	-1.703337	-3.917103	-0.178858
H	-3.399699	-4.287768	0.148019
C	-3.804489	-1.446632	-2.245973
H	-3.879141	-1.349569	-3.320924
H	-4.828891	-1.621929	0.879262
O	-3.013716	0.937904	0.076414

1 imaginary frequency

E_{B3LYP}= -2432.836841

G_{B3LYP}= -2432.426651

E_{M06-2X}= -2432.742962

G_{tot}= -2432.329753

endo-Product enolsilane D

Charge = 0 Multiplicity = 1

C	5.103620	1.488378	-0.675489
C	4.386570	2.023777	0.314540
C	3.011470	2.368247	-0.239446
O	3.253381	2.460876	-1.644920
C	2.340530	3.627854	0.281083
C	1.822525	3.443393	1.713385
C	0.848346	2.263883	1.816522
C	1.499784	0.952051	1.368483
O	0.654694	-0.170004	1.485700
Si	-0.928802	-0.449441	0.950420

C	-1.152128	0.128935	-0.827507
C	2.028122	1.115697	-0.066602
C	2.758811	-0.056861	-0.613969
O	2.920312	-1.061570	0.129716
Si	3.543328	-2.742018	-0.030767
C	5.394570	-2.586678	-0.189632
C	3.412486	0.078886	-1.944619
C	2.977092	-3.448660	1.591193
C	2.655949	-3.433172	-1.516816
C	-2.134091	0.377948	2.128880
C	-1.076666	-2.315537	1.045361
O	-4.029841	-1.592297	-0.447585
S	-5.281053	-0.852935	-0.145826
O	-6.465945	-1.296793	-0.914057
C	-4.951534	0.842464	-0.833772
F	-4.569576	0.767471	-2.122111
O	-5.520989	-0.593096	1.293761
F	-3.975667	1.471882	-0.153272
F	-6.054587	1.607056	-0.770291
H	2.653500	0.172185	-2.726680
H	4.064770	-0.764668	-2.167665
H	3.440779	-2.921807	2.430152
H	3.253409	-4.505860	1.656736
H	1.890783	-3.370336	1.688921
H	1.573952	-3.308936	-1.412047
H	2.972231	-2.968320	-2.454114
H	2.864850	-4.505927	-1.588091
H	5.798859	-1.947685	0.600881
H	5.842067	-3.580576	-0.080688
H	5.706437	-2.184424	-1.157070
H	1.202333	1.335605	-0.751055
H	2.361404	0.754333	2.020037
H	-2.127026	1.466994	2.025494
H	-1.866828	0.135435	3.163387
H	-3.154914	0.031901	1.944979
H	-0.377401	-2.802441	0.356753
H	-0.868557	-2.681577	2.056231
H	-2.092921	-2.608650	0.767226

H	-1.131873	1.218707	-0.930423
H	-0.387845	-0.298119	-1.486140
H	-2.127360	-0.219440	-1.176473
H	6.100857	1.070774	-0.630074
H	4.674642	2.147448	1.350043
H	-0.030415	2.460176	1.189919
H	0.491871	2.143258	2.843201
H	2.665731	3.285535	2.397343
H	1.329204	4.363374	2.038607
H	1.507646	3.865620	-0.390266
H	3.057672	4.452007	0.223308
C	4.211211	1.443316	-1.900088
H	4.691199	1.614693	-2.863269

0 imaginary frequencies

E_{B3LYP}= -2432.851806

G_{B3LYP}= -2432.440230

E_{M06-2X}= -2432.764225

G_{tot}= -2432.349630

exo-TS enolsilane D

Charge = 0 Multiplicity = 1

C	-5.447630	-0.014852	0.682044
C	-5.170360	-0.869361	-0.429655
C	-4.575771	-2.001103	0.071103
O	-4.464922	-1.884129	1.433731
O	-0.442214	-1.160085	-1.179405
C	-2.070683	-0.729176	-0.701330
C	-2.224930	0.447769	0.137621
O	-2.477307	1.560155	-0.589302
Si	-2.530427	3.198901	-0.084372
C	-4.085281	3.479793	0.925930
C	-2.198801	0.357919	1.481724
C	-2.585267	4.122799	-1.707534
C	-0.974590	3.592486	0.887026
H	-2.000105	-0.585116	1.969153
H	-2.385872	1.210800	2.119052

H	-3.450784	3.817846	-2.303874
H	-2.659069	5.200960	-1.531326
H	-1.681178	3.937338	-2.295552
H	-0.078398	3.340384	0.312319
H	-0.921746	3.067065	1.844078
H	-0.945104	4.667188	1.097628
H	-4.977192	3.301509	0.317345
H	-4.118903	4.517837	1.274288
H	-4.139056	2.830302	1.804139
H	-5.903664	0.962922	0.659957
H	-5.402147	-0.687769	-1.469154
C	-4.987956	-0.668194	1.785832
H	-4.961286	-0.430196	2.836928
C	-1.334034	-1.939407	-0.334135
H	-0.987555	-1.985132	0.696931
Si	1.155317	-0.383532	-0.807766
C	1.242389	-0.266070	1.055108
C	2.251471	-1.656379	-1.622566
C	0.919145	1.222105	-1.728909
H	1.173089	-1.251543	1.526389
H	0.442286	0.365388	1.447132
H	2.199701	0.175033	1.332591
H	2.240226	-2.591552	-1.054306
H	1.877875	-1.863579	-2.630301
H	3.283678	-1.307692	-1.694416
H	0.946846	1.048020	-2.809049
H	1.717724	1.918696	-1.469710
H	-0.048666	1.665037	-1.481815
O	5.478916	-0.317034	-1.466785
S	5.097972	0.548133	-0.326328
O	3.703826	1.064050	-0.380724
O	6.108664	1.543385	0.087956
C	5.044308	-0.633319	1.106194
F	4.629045	-0.006785	2.222996
F	4.193871	-1.649419	0.866223
F	6.256687	-1.155091	1.345918
H	-2.547791	-0.711021	-1.671920
C	-4.257700	-3.331990	-0.526087

H	-5.028791	-4.038581	-0.192190
H	-4.370554	-3.244845	-1.611546
C	-1.695284	-3.283409	-0.909367
H	-0.811050	-3.923089	-0.823366
H	-1.912185	-3.168989	-1.976752
C	-2.883157	-3.939358	-0.188338
H	-2.907167	-4.994216	-0.475512
H	-2.726503	-3.918904	0.896063

1 imaginary frequency

E_{B3LYP}= -2432.799006

G_{B3LYP}= -2432.395316

$E_{M06\text{-}2X}$= -2432.705348

G_{tot}= -2432.298639

exo-INT-S_N2' enolsilane D

Charge = 0 Multiplicity = 1

C	5.820728	-0.359730	0.405662
C	5.307436	-0.896351	-0.876406
O	4.616534	-2.112896	-0.479639
C	4.434084	-2.066181	0.816728
C	5.233263	-1.045674	1.418574
C	3.564065	-3.081133	1.446636
C	2.483200	-3.679195	0.530810
C	1.282313	-2.776702	0.212337
C	1.542527	-1.560693	-0.696491
C	2.169684	-0.394780	0.024092
C	3.204252	0.359809	-0.398209
C	4.165168	0.004924	-1.489255
O	0.340826	-1.102091	-1.312987
Si	-1.178111	-0.745478	-0.647907
C	-1.873725	0.562000	-1.796593
C	-1.080747	-0.078616	1.107435
C	-2.242907	-2.293870	-0.667349
Si	3.075086	3.036517	0.101499
C	3.477235	3.549978	-1.657626
C	1.228114	3.092744	0.404700

C	4.043271	4.016191	1.364497
F	-5.045055	-0.492850	-1.413761
C	-5.960064	-0.041933	-0.532390
F	-6.636295	0.960030	-1.119605
F	-6.823356	-1.042894	-0.289535
S	-5.149104	0.531778	1.036658
O	-4.254723	1.620822	0.575840
O	-6.292889	0.970962	1.868998
O	-4.457503	-0.688533	1.519755
H	4.639820	0.898876	-1.894859
H	3.714168	-0.561917	-2.303277
H	3.829377	3.668631	2.379949
H	3.777960	5.077056	1.308219
H	5.120385	3.928123	1.191177
H	4.553209	3.507333	-1.854946
H	2.966770	2.915033	-2.389312
H	3.146483	4.580030	-1.829508
H	0.982886	2.774128	1.422459
H	0.851697	4.112334	0.269718
H	0.692715	2.443884	-0.295059
H	1.644950	-0.046616	0.908752
H	2.190340	-1.887946	-1.516287
H	-1.853970	-3.058162	0.013411
H	-2.297833	-2.729391	-1.670685
H	-3.254106	-2.034626	-0.341820
H	-1.219809	1.440446	-1.825833
H	-1.978655	0.183826	-2.818871
H	-2.856406	0.878964	-1.437803
H	-0.609530	-0.777074	1.806285
H	-0.547687	0.874577	1.159897
H	-2.110125	0.084975	1.441393
H	6.476820	0.496025	0.478457
H	0.810561	-2.446155	1.144934
H	0.547778	-3.396791	-0.309975
H	2.947893	-4.024832	-0.398051
H	2.104553	-4.571553	1.036316
H	3.139927	-2.644837	2.356447
H	4.239932	-3.880314	1.787809

H	6.048295	-1.148749	-1.634480
H	5.299255	-0.865284	2.481041
O	3.620922	1.429698	0.340644

0 imaginary frequencies

E_{B3LYP}= -2432.843355

G_{B3LYP}= -2432.434712

E_{M06-2X}= -2432.753788

G_{tot}= -2432.342126

exo-INT-S_N2 enolsilane D

Charge = 0 Multiplicity = 1

C	-5.186914	-0.404524	-1.247433
C	-5.318796	-1.183978	-0.075354
O	-4.343812	-2.023550	0.097244
C	-3.297758	-1.700327	-0.888245
C	-3.978907	-0.738326	-1.783532
C	-2.771159	-2.993678	-1.512578
C	-2.095129	-3.897704	-0.476220
C	-0.995304	-3.157842	0.291723
C	-1.549426	-1.914600	0.991950
C	-2.172679	-0.954134	-0.044520
C	-2.716035	0.336619	0.498039
C	-3.370184	0.454990	1.666479
O	-0.595950	-1.233628	1.769512
Si	0.936956	-0.608454	1.390739
C	1.252398	0.609584	2.778333
C	0.913291	0.287197	-0.263847
C	2.191782	-2.006236	1.423953
Si	-2.670198	3.024780	-0.235449
C	-2.009295	3.647885	-1.868445
C	-4.463204	3.518463	0.010436
C	-1.575886	3.506124	1.207220
F	3.586430	-1.017749	-1.244570
C	4.844708	-0.614891	-0.987316
F	5.439061	-1.569847	-0.249380
F	5.495035	-0.530383	-2.160605

S	4.872131	1.017977	-0.098883
O	4.279696	0.699076	1.223402
O	6.313235	1.355170	-0.071284
O	4.036589	1.885629	-0.963979
H	-3.792837	1.394554	1.996722
H	-3.424028	-0.382734	2.348198
H	-1.919566	3.067993	2.148165
H	-1.563765	4.594669	1.325927
H	-0.547160	3.174105	1.034867
H	-0.977233	3.318534	-2.022759
H	-2.614690	3.281524	-2.703543
H	-2.023674	4.742365	-1.895817
H	-4.877549	3.152228	0.953804
H	-4.550375	4.610433	0.010008
H	-5.083916	3.133749	-0.805273
H	-1.414425	-0.698501	-0.786253
H	-2.339607	-2.236462	1.681414
H	2.104295	-2.662648	0.553333
H	2.060112	-2.614823	2.325081
H	3.200614	-1.586524	1.430564
H	0.508576	1.413240	2.773776
H	1.219078	0.121119	3.757745
H	2.244764	1.048959	2.642722
H	0.828298	-0.399842	-1.112289
H	0.083421	0.998645	-0.312158
H	1.846987	0.841939	-0.392094
H	-5.908422	0.315066	-1.603254
H	-0.196328	-2.858230	-0.397692
H	-0.544517	-3.811413	1.043528
H	-2.848388	-4.273962	0.224846
H	-1.680124	-4.768567	-0.990924
H	-2.056266	-2.695470	-2.286402
H	-3.598284	-3.505589	-2.011041
H	-6.128906	-1.215213	0.643660
H	-3.522556	-0.340490	-2.679158
O	-2.573010	1.319707	-0.419957

0 imaginary frequencies

E_{B3LYP}= -2432.839299

G_{B3LYP}= -2432.430671
E_{M06-2X}= -2432.747903
G_{tot}= -2432.336256

exo-TS2-S_N2' enolsilane D

Charge = 0 Multiplicity = 1

C	-5.567027	-0.375803	-0.510962
C	-5.171411	-1.192926	0.678324
O	-4.310881	-2.196176	0.123663
C	-3.720570	-1.611421	-0.951125
C	-4.649809	-0.601689	-1.469300
C	-2.857115	-2.497246	-1.794207
C	-1.916568	-3.401641	-0.991342
C	-0.870040	-2.624317	-0.188477
C	-1.483505	-1.642632	0.812832
C	-2.245870	-0.518366	0.116764
C	-3.192418	0.261905	0.799930
C	-4.223276	-0.355780	1.667694
O	-0.528185	-1.049041	1.676850
Si	1.048877	-0.494206	1.363970
C	1.401564	0.634045	2.817131
C	1.113976	0.480775	-0.244769
C	2.228714	-1.955337	1.354214
Si	-2.705072	2.787434	-0.422924
C	-1.416203	3.450949	0.754452
C	-2.007178	2.191704	-2.054947
C	-4.137724	3.951311	-0.685539
F	3.723367	-0.961553	-1.249118
C	4.996012	-0.656948	-0.932926
F	5.490865	-1.669293	-0.198190
F	5.697254	-0.592046	-2.077637
S	5.102779	0.949128	-0.002503
O	4.434196	0.645302	1.286716
O	6.561605	1.181148	0.091200
O	4.366439	1.892193	-0.878607
H	-4.817051	0.407378	2.168832

H	-3.822506	-1.065170	2.389785
H	-4.907044	3.491053	-1.313068
H	-3.794690	4.863162	-1.185174
H	-4.593385	4.235987	0.267284
H	-1.889492	3.847146	1.657741
H	-0.699521	2.682117	1.054462
H	-0.856658	4.262792	0.278265
H	-2.731560	1.589665	-2.610723
H	-1.784215	3.076634	-2.662021
H	-1.079404	1.623574	-1.958027
H	-1.663425	0.004317	-0.635188
H	-2.175448	-2.199675	1.451866
H	2.144760	-2.551202	0.441278
H	2.032589	-2.609572	2.210524
H	3.255460	-1.587881	1.423247
H	0.702387	1.476265	2.851367
H	1.329339	0.095570	3.767823
H	2.417466	1.026770	2.717420
H	0.926372	-0.138689	-1.127278
H	0.383228	1.293753	-0.235219
H	2.103949	0.928112	-0.366488
H	-6.380496	0.335900	-0.519812
H	-0.208224	-2.082122	-0.873375
H	-0.247254	-3.327287	0.371284
H	-2.510278	-4.029625	-0.318927
H	-1.411126	-4.075276	-1.688361
H	-2.304067	-1.867477	-2.497528
H	-3.545106	-3.108853	-2.392932
H	-5.963306	-1.666322	1.255273
H	-4.551478	-0.123656	-2.433427
O	-3.486955	1.487560	0.454493

1 imaginary frequency

E_{B3LYP}= -2432.840872

G_{B3LYP}= -2432.429120

E_{M06-2X}= -2432.747020

G_{tot}= -2432.332249

exo-TS2-S_N2 enolsilane D

Charge = 0 Multiplicity = 1

C	-5.038696	-0.391102	-1.247662
C	-5.092974	-1.005997	0.056076
O	-4.290582	-2.062154	0.106448
C	-3.269242	-1.829517	-0.906768
C	-3.957376	-0.916172	-1.865990
C	-2.702710	-3.142291	-1.425475
C	-1.996415	-3.954063	-0.333441
C	-0.911368	-3.134974	0.375777
C	-1.507369	-1.870967	0.995602
C	-2.143736	-0.992267	-0.102512
C	-2.769270	0.267216	0.384945
C	-3.728433	0.278630	1.375154
O	-0.598731	-1.101702	1.747403
Si	0.948599	-0.504906	1.374476
C	1.242656	0.755330	2.728719
C	0.949584	0.332035	-0.310368
C	2.198023	-1.904009	1.478305
Si	-2.789247	3.002425	-0.229392
C	-2.123884	3.481140	1.451543
C	-1.776167	3.693351	-1.633890
C	-4.618632	3.336702	-0.444078
F	3.624406	-1.016340	-1.213645
C	4.880428	-0.616108	-0.941543
F	5.446533	-1.546834	-0.152215
F	5.559858	-0.584460	-2.100818
S	4.903830	1.050501	-0.118051
O	4.274060	0.791142	1.200035
O	6.347491	1.373477	-0.066457
O	4.100067	1.891405	-1.037819
H	-4.239847	1.195430	1.639498
H	-3.748196	-0.516944	2.106887
H	-4.967191	2.966817	-1.413264
H	-4.795912	4.417385	-0.417173
H	-5.231334	2.881163	0.338732
H	-2.675588	3.014678	2.271934

H	-1.071651	3.192781	1.539068
H	-2.187379	4.566999	1.578102
H	-2.128689	3.311474	-2.596848
H	-1.848477	4.785564	-1.655235
H	-0.721351	3.424708	-1.522551
H	-1.389483	-0.729396	-0.844050
H	-2.299938	-2.177981	1.688213
H	2.135063	-2.583227	0.623561
H	2.042575	-2.487954	2.391806
H	3.206170	-1.482944	1.500265
H	0.494029	1.554099	2.696642
H	1.202677	0.293675	3.720806
H	2.233524	1.197005	2.590880
H	0.869294	-0.385194	-1.133898
H	0.122742	1.044144	-0.392641
H	1.886939	0.877774	-0.449120
H	-5.713333	0.376305	-1.596829
H	-0.125463	-2.857264	-0.337377
H	-0.438118	-3.724738	1.165661
H	-2.735300	-4.297491	0.399419
H	-1.560193	-4.849226	-0.784918
H	-1.997923	-2.887984	-2.224589
H	-3.515741	-3.714262	-1.880722
H	-5.899024	-0.969905	0.776958
H	-3.562565	-0.666275	-2.840916
O	-2.521830	1.290906	-0.410473

1 imaginary frequency

E_{B3LYP}= -2432.837560

G_{B3LYP}= -2432.426909

E_{M06-2X}= -2432.743050

G_{tot}= -2432.329380

exo-Product enolsilane D

Charge = 0 Multiplicity = 1

C	5.151617	0.783711	-1.530857
C	5.141851	0.834870	-0.014085

O	4.406910	2.023428	0.269235
C	3.382314	2.049931	-0.742684
C	4.106118	1.492215	-1.958962
C	2.790542	3.443194	-0.872044
C	2.056340	3.887088	0.398745
C	0.997451	2.863420	0.827962
C	1.631854	1.492847	1.065745
C	2.299733	1.012676	-0.277724
C	2.942527	-0.301311	-0.047679
C	4.310311	-0.362338	0.548061
O	0.775911	0.503333	1.569034
Si	-0.833273	0.111438	1.166135
C	-1.101450	-1.495703	2.090840
C	-0.991785	-0.117419	-0.692574
C	-1.991538	1.435641	1.824500
O	2.284658	-1.329496	-0.370550
Si	2.481159	-3.110633	-0.150345
C	3.925215	-3.600387	-1.224123
C	2.718903	-3.338932	1.683896
C	0.843785	-3.701938	-0.800245
F	-3.708095	1.440248	-0.843853
C	-4.947381	0.965659	-0.618186
F	-5.443541	1.606333	0.455386
F	-5.706771	1.282632	-1.680580
S	-4.934249	-0.872537	-0.340536
O	-4.208670	-1.017370	0.945744
O	-6.374005	-1.210911	-0.285584
O	-4.208268	-1.388032	-1.526504
H	4.784084	-1.324588	0.352838
H	4.212600	-0.239885	1.634222
H	0.010521	-3.282820	-0.230713
H	0.790379	-4.793158	-0.728997
H	0.718556	-3.423560	-1.850675
H	3.814172	-3.191984	-2.232890
H	4.890088	-3.281812	-0.821670
H	3.942643	-4.692770	-1.305149
H	1.958997	-2.788551	2.246114
H	2.612271	-4.401619	1.926122

H	3.706754	-3.015929	2.021994
H	1.516576	0.889487	-1.027576
H	2.442311	1.614491	1.792372
H	-1.972337	2.348288	1.222974
H	-1.728858	1.697119	2.855117
H	-3.012791	1.046224	1.813448
H	-0.403362	-2.275827	1.773398
H	-0.975284	-1.351388	3.169062
H	-2.121742	-1.842202	1.904926
H	-0.898262	0.831658	-1.231204
H	-0.224363	-0.800649	-1.067051
H	-1.972372	-0.534271	-0.938048
H	5.842675	0.190515	-2.115538
H	0.217569	2.782934	0.061199
H	0.511436	3.178948	1.755831
H	2.782045	4.020353	1.208786
H	1.586773	4.860054	0.229059
H	2.096816	3.432245	-1.720871
H	3.599320	4.136060	-1.122656
H	3.749990	1.610589	-2.974342
H	6.115318	0.881503	0.474345

0 imaginary frequencies

E_{B3LYP}= -2432.857948

G_{B3LYP}= -2432.445517

E_{M06-2X}= -2432.769136

G_{tot}= -2432.353686

endo-TS allylsilane C

Charge = 0 Multiplicity = 1

C	5.374876	2.096877	-0.443109
C	4.470452	2.324967	0.642191
C	3.372166	2.957569	0.120574
O	3.552659	3.132715	-1.229574
C	2.173296	3.618636	0.722451
C	1.588188	2.906942	1.955028
C	0.679189	1.709732	1.629685

C	1.433755	0.536798	1.055559
O	0.668263	-0.512013	0.374664
Si	-1.090028	-0.671329	-0.042011
C	-1.463592	0.731841	-1.221262
C	1.854721	0.513477	-0.344619
C	2.991997	-0.197720	-0.893267
Si	3.415842	-2.842846	0.287020
C	2.659265	-3.536164	-1.291961
C	3.197265	-0.023799	-2.215820
C	5.024245	-3.743924	0.691784
C	2.237673	-3.012680	1.748900
C	-1.863962	-0.642476	1.656744
C	-1.020137	-2.343003	-0.863948
O	-3.935790	-1.174856	-0.871581
S	-5.186069	-0.762600	-0.178957
O	-6.423036	-0.972497	-0.959735
C	-5.029262	1.087601	-0.112915
F	-4.869829	1.596026	-1.347887
O	-5.254842	-1.152535	1.248368
F	-3.964460	1.459926	0.624964
F	-6.122403	1.643351	0.431215
H	2.514364	0.556679	-2.826532
H	4.055665	-0.460006	-2.714829
H	5.729457	-3.692079	-0.144520
H	4.835346	-4.800976	0.907435
H	5.511455	-3.306540	1.570075
H	2.684298	-2.598351	2.659575
H	1.291102	-2.499420	1.568301
H	2.022194	-4.069946	1.937987
H	3.362418	-3.459011	-2.128185
H	2.397371	-4.592281	-1.169106
H	1.751322	-2.993602	-1.567269
H	1.302348	1.160317	-1.015630
H	2.112073	0.065973	1.764238
H	-1.952125	0.375170	2.043433
H	-1.250936	-1.227509	2.349934
H	-2.867161	-1.073223	1.622334
H	-0.445183	-2.294373	-1.793522

H	-0.554358	-3.084243	-0.208573
H	-2.037004	-2.663312	-1.099105
H	-1.504226	1.707967	-0.731973
H	-0.724437	0.770859	-2.027752
H	-2.441695	0.542533	-1.666663
H	6.336802	1.608128	-0.400519
H	4.623917	2.068392	1.680099
H	-0.095510	2.030851	0.928205
H	0.177331	1.377317	2.542347
H	2.391827	2.600450	2.633757
H	0.982769	3.628902	2.509536
H	1.403538	3.726103	-0.049962
H	2.451233	4.640494	1.011361
C	4.767753	2.596346	-1.555786
H	5.044209	2.651203	-2.596344
C	3.903452	-1.012056	-0.023471
H	4.066443	-0.519038	0.939913
H	4.885008	-1.051397	-0.507193

1 imaginary frequency

E_{B3LYP}= -2396.863860

G_{B3LYP}= -2396.436050

E_{M06-2X}= -2396.746139

G_{tot}= -2396.315310

endo-INT-S_N2’ allylsilane C

Charge = 0 Multiplicity = 1

C	-5.215040	-1.868787	-0.916289
C	-4.739287	-2.408645	0.232645
C	-3.418931	-2.890909	-0.053881
O	-3.149417	-2.790192	-1.333644
C	-2.466322	-3.588639	0.838193
C	-2.102478	-2.826243	2.140907
C	-1.040619	-1.721990	2.010662
C	-1.533362	-0.395887	1.413130
O	-0.566865	0.640726	1.549362
Si	1.019953	0.750483	0.959396

C	1.161145	0.043078	-0.778866
C	-1.952706	-0.581257	-0.016873
C	-3.026228	-0.023764	-0.623499
Si	-3.401454	2.770969	-0.207956
C	-2.166856	2.881742	-1.627947
C	-3.370883	-0.487319	-2.007908
C	-4.936183	3.799518	-0.586245
C	-2.598032	3.322552	1.399866
C	2.166478	-0.116971	2.169829
C	1.342066	2.597087	0.936169
O	4.224890	1.474729	-0.507168
S	5.389097	0.603769	-0.210943
O	6.594382	0.877532	-1.026003
C	4.843142	-1.063863	-0.825169
F	4.426397	-0.988823	-2.102710
O	5.641490	0.365987	1.230390
F	3.827826	-1.554352	-0.090517
F	5.853688	-1.948265	-0.769032
H	-2.491878	-0.663098	-2.630051
H	-4.042768	0.197144	-2.529201
H	-5.403544	3.482361	-1.524636
H	-4.680688	4.860498	-0.679418
H	-5.681045	3.703062	0.210985
H	-3.323003	3.321815	2.221138
H	-1.776627	2.648296	1.660315
H	-2.196280	4.337219	1.305264
H	-2.594367	2.530788	-2.573100
H	-1.847746	3.919428	-1.772287
H	-1.275837	2.283034	-1.416618
H	-1.300333	-1.207612	-0.617428
H	-2.397758	-0.066024	2.001358
H	2.034443	-1.203168	2.146434
H	1.958717	0.225191	3.189816
H	3.213756	0.096566	1.939761
H	0.651497	3.107570	0.256688
H	1.221174	3.036954	1.931544
H	2.365305	2.777400	0.594478
H	1.079793	-1.048090	-0.798873

H	0.393940	0.458240	-1.439910
H	2.143608	0.311463	-1.175263
H	-6.177191	-1.403643	-1.080464
H	-0.191781	-2.096330	1.425050
H	-0.658129	-1.501585	3.012229
H	-3.012376	-2.422605	2.599842
H	-1.722652	-3.581439	2.833439
H	-1.570987	-3.849202	0.269622
H	-2.962202	-4.526405	1.124192
C	-4.126168	-1.888519	-1.919011
H	-4.376302	-2.243403	-2.917876
C	-3.973314	0.934308	0.012893
H	-4.075654	0.764823	1.088442
H	-4.970818	0.844145	-0.431379
H	-5.222380	-2.474265	1.196046

0 imaginary frequencies

E_{B3LYP}= -2396.906705

G_{B3LYP}= -2396.472673

E_{M06-2X}= -2396.789008

G_{tot}= -2396.351957

endo-INT-S_N2 allylsilane C

Charge = 0 Multiplicity = 1

C	-5.173214	-2.282733	-0.744402
C	-4.395261	-2.077400	0.356953
C	-2.981257	-2.354038	0.022274
O	-3.078539	-2.891526	-1.345124
C	-2.278734	-3.407808	0.889447
C	-1.882801	-2.819696	2.245130
C	-0.955056	-1.619900	2.055040
C	-1.618895	-0.515870	1.230156
O	-0.787729	0.602509	1.025540
Si	0.858344	0.732470	0.612285
C	1.177314	-0.170342	-1.005879
C	-2.098922	-1.033424	-0.147150
C	-2.830536	-0.015521	-0.991267

Si	-3.457750	2.711441	-0.052797
C	-2.266001	3.313763	-1.380232
C	-2.582958	0.021128	-2.310814
C	-5.102762	3.629093	-0.215495
C	-2.785951	2.985548	1.683386
C	1.934564	0.110149	2.023515
C	1.103304	2.579283	0.419661
O	4.116770	1.464285	-0.649737
S	5.292087	0.709887	-0.148057
O	6.548021	0.945377	-0.895561
C	4.882496	-1.053280	-0.572465
F	4.580076	-1.171681	-1.878558
O	5.430335	0.679195	1.327557
F	3.828494	-1.501601	0.134541
F	5.923911	-1.861766	-0.311836
H	-1.833403	-0.617542	-2.766287
H	-3.107319	0.709874	-2.965720
H	-5.525394	3.517129	-1.219787
H	-4.970404	4.699600	-0.024293
H	-5.838773	3.251982	0.503239
H	-3.515883	2.662491	2.434284
H	-1.860318	2.426608	1.833481
H	-2.587497	4.050176	1.849815
H	-2.740750	3.306894	-2.367006
H	-1.940211	4.338155	-1.171097
H	-1.381362	2.675248	-1.431142
H	-1.218522	-1.385211	-0.689316
H	-2.489203	-0.150110	1.790683
H	1.957341	-0.980008	2.096147
H	1.569338	0.507719	2.976947
H	2.964158	0.450525	1.879942
H	0.510634	2.991958	-0.400833
H	0.826263	3.109820	1.336557
H	2.160502	2.765890	0.210417
H	1.147038	-1.259093	-0.895396
H	0.438419	0.123914	-1.758229
H	2.171543	0.099661	-1.369605
H	-6.236928	-2.137410	-0.856061

H	-0.043324	-1.951875	1.546276
H	-0.653264	-1.201810	3.018798
H	-2.775009	-2.529313	2.812817
H	-1.383507	-3.597077	2.829515
H	-1.384798	-3.726037	0.344722
H	-2.933013	-4.276199	0.996662
C	-4.297617	-2.744542	-1.753114
H	-4.511771	-3.021976	-2.778816
C	-3.881645	0.856481	-0.361709
H	-4.229455	0.457481	0.597082
H	-4.758106	0.881232	-1.018175
H	-4.728330	-1.754289	1.333511

0 imaginary frequencies

E_{B3LYP}= -2396.896015

G_{B3LYP}= -2396.463619

$E_{M06\text{-}2X}$= -2396.779422

G_{tot}= -2396.344007

endo-TS2-S_N2' allylsilane C

Charge = 0 Multiplicity = 1

C	-5.320004	-1.758546	-0.614264
C	-4.588164	-2.208659	0.417360
C	-3.241737	-2.522105	-0.109784
O	-3.365367	-2.610205	-1.466771
C	-2.321713	-3.537151	0.509091
C	-1.782218	-3.134413	1.886518
C	-0.839544	-1.929542	1.822112
C	-1.547007	-0.659925	1.345098
O	-0.717449	0.494092	1.355856
Si	0.901147	0.655954	0.846933
C	1.109135	-0.078219	-0.873930
C	-2.142265	-0.848840	-0.040945
C	-3.137050	0.015626	-0.551305
Si	-3.136908	2.802379	-0.103844
C	-1.906002	2.741464	-1.522323
C	-3.663180	-0.272758	-1.906784

C	-4.634856	3.834507	-0.580272
C	-2.370191	3.380904	1.504650
C	2.043679	-0.133765	2.112854
C	1.176115	2.509376	0.828885
O	4.089430	1.461174	-0.558991
S	5.281597	0.654856	-0.196314
O	6.496438	0.950051	-0.989047
C	4.825028	-1.057461	-0.757720
F	4.454297	-1.051984	-2.051402
O	5.501854	0.486373	1.259894
F	3.801745	-1.557284	-0.040274
F	5.865972	-1.897042	-0.626604
H	-2.881863	-0.410302	-2.654806
H	-4.382244	0.473222	-2.246810
H	-5.104764	3.459317	-1.495052
H	-4.332439	4.872165	-0.758567
H	-5.385846	3.833380	0.216135
H	-3.135378	3.490621	2.279901
H	-1.624777	2.657463	1.842035
H	-1.881181	4.350967	1.366969
H	-2.383223	2.434580	-2.458163
H	-1.476998	3.736621	-1.679925
H	-1.084886	2.052569	-1.313714
H	-1.444741	-1.242200	-0.775978
H	-2.360098	-0.455228	2.052261
H	2.024882	-1.225941	2.075915
H	1.751070	0.177604	3.121847
H	3.075262	0.185188	1.939067
H	0.509512	3.023085	0.131251
H	1.021656	2.940685	1.823183
H	2.209795	2.696633	0.523617
H	1.055605	-1.171475	-0.874697
H	0.351532	0.302315	-1.566693
H	2.093400	0.206493	-1.253543
H	-6.338373	-1.395514	-0.603457
H	-0.004096	-2.160784	1.151204
H	-0.414904	-1.733806	2.810228
H	-2.614883	-2.924675	2.569077

H	-1.248653	-3.991019	2.306414
H	-1.497798	-3.723020	-0.186231
H	-2.898636	-4.466776	0.587025
C	-4.409347	-1.697830	-1.802806
H	-4.826154	-1.948848	-2.776266
C	-3.849177	0.984501	0.249924
H	-3.747887	0.845117	1.325911
H	-4.901926	1.056985	-0.037888
H	-4.872266	-2.297843	1.456364

1 imaginary frequency

E_{B3LYP}= -2396.898672

G_{B3LYP}= -2396.462567

E_{M06-2X}= -2396.782200

G_{tot}= -2396.343076

endo-TS2-S_N2 allylsilane C

Charge = 0 Multiplicity = 1

C	-5.206834	-1.822382	-0.703494
C	-4.478720	-2.095199	0.399515
C	-3.084263	-2.401114	-0.037690
O	-3.239703	-2.702660	-1.434356
C	-2.321309	-3.510359	0.666930
C	-1.844215	-3.070642	2.052692
C	-0.920315	-1.856772	1.947165
C	-1.624619	-0.658158	1.308440
O	-0.807575	0.491456	1.217371
Si	0.832757	0.651735	0.781949
C	1.085869	-0.056819	-0.942692
C	-2.165392	-1.009947	-0.090102
C	-3.012724	-0.002126	-0.776059
Si	-3.128440	2.790540	-0.066403
C	-1.868986	2.944982	-1.453864
C	-3.248637	-0.178163	-2.136873
C	-4.629429	3.861507	-0.457386
C	-2.450710	3.225150	1.627463
C	1.944637	-0.152346	2.067206

C	1.118713	2.502950	0.823050
O	4.055992	1.474903	-0.538875
S	5.234359	0.661376	-0.148169
O	6.473128	0.959659	-0.901676
C	4.790247	-1.044240	-0.739268
F	4.458662	-1.025391	-2.043342
O	5.410356	0.479262	1.312449
F	3.744502	-1.548115	-0.057811
F	5.824560	-1.888064	-0.584835
H	-2.517881	-0.684326	-2.757000
H	-3.927885	0.497398	-2.648815
H	-5.053383	3.612427	-1.435763
H	-4.346563	4.919709	-0.472453
H	-5.413921	3.734880	0.296101
H	-3.253721	3.214872	2.372257
H	-1.681428	2.511506	1.926185
H	-2.015115	4.230021	1.614600
H	-2.336004	2.760567	-2.426659
H	-1.450608	3.956827	-1.471693
H	-1.044053	2.239745	-1.336164
H	-1.334893	-1.314334	-0.728630
H	-2.467650	-0.386361	1.955798
H	1.966959	-1.242350	1.998398
H	1.610506	0.120280	3.074389
H	2.970146	0.205431	1.936390
H	0.466500	3.050360	0.139088
H	0.957879	2.897648	1.831560
H	2.158317	2.689435	0.537805
H	1.046852	-1.150898	-0.951170
H	0.331897	0.319444	-1.642157
H	2.072271	0.243008	-1.304405
H	-6.229015	-1.479900	-0.772013
H	-0.044539	-2.126438	1.346303
H	-0.556045	-1.557614	2.933209
H	-2.703302	-2.838343	2.694171
H	-1.318063	-3.902289	2.528502
H	-1.461581	-3.772990	0.041854
H	-2.968542	-4.389361	0.727154

C	-4.309632	-1.999761	-1.833724
H	-4.594786	-2.112478	-2.872038
C	-3.824486	0.955768	-0.023913
H	-3.953041	0.690136	1.026626
H	-4.808613	1.061650	-0.489940
H	-4.793162	-2.032348	1.431764

1 imaginary frequency

E_{B3LYP}= -2396.890790

G_{B3LYP}= -2396.455000

E_{M06-2X}= -2396.772691

G_{tot}= -2396.333882

endo-Product allylsilane C

Charge = 0 Multiplicity = 1

C	-5.465890	-1.783621	-0.174023
C	-4.505236	-2.239072	0.631586
C	-3.218299	-2.315291	-0.175595
O	-3.689609	-2.364373	-1.517427
C	-2.304472	-3.491862	0.116428
C	-1.596668	-3.326089	1.462366
C	-0.736454	-2.060993	1.472164
C	-1.559113	-0.794724	1.213287
O	-0.781855	0.396243	1.160029
Si	0.864768	0.569226	0.735725
C	1.164030	-0.141870	-0.983157
C	-2.384681	-0.917888	-0.077888
C	-3.418844	0.126632	-0.316760
Si	-2.915136	2.844578	-0.098697
C	-1.778569	2.518481	-1.548337
C	-4.236547	-0.033904	-1.552469
C	-4.398225	3.851503	-0.646612
C	-2.097570	3.517394	1.439826
C	1.967645	-0.206917	2.045099
C	1.167725	2.419056	0.738928
O	4.122529	1.442786	-0.606153
S	5.291799	0.655361	-0.140646

O	6.558215	0.945232	-0.850033
C	4.891528	-1.073050	-0.696079
F	4.608195	-1.097279	-2.011005
O	5.407289	0.521541	1.331244
F	3.827982	-1.570899	-0.038105
F	5.930238	-1.897111	-0.478122
H	-3.614243	0.023518	-2.449367
H	-5.036952	0.706026	-1.610559
H	-4.939004	3.370114	-1.466879
H	-4.048879	4.826027	-1.006056
H	-5.095278	4.022827	0.178612
H	-2.852152	3.764032	2.192496
H	-1.405879	2.781621	1.851983
H	-1.539598	4.427336	1.196038
H	-2.340357	2.203636	-2.432204
H	-1.262078	3.450457	-1.802160
H	-1.023072	1.766692	-1.324437
H	-1.711179	-0.978710	-0.938566
H	-2.252379	-0.683288	2.057588
H	2.013206	-1.296090	1.979343
H	1.606413	0.061879	3.044095
H	2.988186	0.171819	1.934641
H	0.539798	2.963250	0.029996
H	1.011969	2.849056	1.733148
H	2.214216	2.570976	0.456685
H	1.161592	-1.235455	-0.998566
H	0.420372	0.208523	-1.706420
H	2.148601	0.192679	-1.320553
H	-6.493837	-1.552915	0.072639
H	-4.575948	-2.470855	1.685909
H	0.037217	-2.157845	0.703498
H	-0.228521	-1.951076	2.432895
H	-2.333006	-3.288222	2.274579
H	-0.967918	-4.199509	1.655140
H	-1.566746	-3.562935	-0.690704
H	-2.910705	-4.402465	0.093263
C	-4.819897	-1.503896	-1.515878
H	-5.426950	-1.681275	-2.403460

C	-3.764061	1.110220	0.618672
H	-3.342382	1.024367	1.615531
H	-4.806857	1.428289	0.588250

0 imaginary frequencies

E_{B3LYP}= -2396.904337

G_{B3LYP}= -2396.466636

E_{M06-2X}= -2396.792649

G_{tot}= -2396.351929

exo-TS allylsilane C

Charge = 0 Multiplicity = 1

C	4.524454	2.712541	-1.811577
C	5.087792	2.236496	-0.667365
O	4.268423	2.478292	0.401132
C	3.152214	3.132559	-0.075641
C	3.270182	3.289863	-1.428913
C	2.205719	3.701771	0.928160
C	1.705759	2.761545	2.037286
C	0.740978	1.657588	1.572246
C	1.468173	0.489710	0.955107
C	1.855750	0.462679	-0.458497
C	2.983784	-0.256444	-1.003488
C	3.106020	-0.210009	-2.347802
O	0.682415	-0.593094	0.373957
Si	-1.073317	-0.748774	0.001123
C	-1.043396	-2.452856	-0.754633
C	-1.470801	0.606581	-1.223597
C	-1.846030	-0.649636	1.700838
Si	3.559021	-2.752284	0.416572
C	2.471101	-2.809204	1.955192
C	2.726027	-3.616380	-1.033939
C	5.217333	-3.563034	0.808189
F	-3.935424	1.443429	0.560559
C	-5.094248	0.754704	0.556568
F	-5.220824	0.155482	1.753337
F	-6.096261	1.637233	0.421973

S	-5.125155	-0.497041	-0.816237
O	-4.024417	-1.425735	-0.446697
O	-6.482069	-1.074737	-0.720502
O	-4.859212	0.319244	-2.024116
H	2.372552	0.294444	-2.967242
H	3.948970	-0.667488	-2.854600
H	5.869898	-3.577278	-0.071177
H	5.076762	-4.597286	1.139873
H	5.740281	-3.025034	1.606380
H	2.932683	-2.265320	2.786711
H	1.482855	-2.383016	1.769124
H	2.337178	-3.848229	2.275619
H	3.358730	-3.586084	-1.927398
H	2.526696	-4.667104	-0.798713
H	1.773476	-3.139951	-1.280358
H	1.247465	1.050285	-1.133928
H	2.191553	0.049874	1.639543
H	-1.982198	0.383111	2.028500
H	-1.222565	-1.173829	2.431707
H	-2.828749	-1.122948	1.665949
H	-0.455954	-2.456459	-1.677856
H	-0.606169	-3.180697	-0.064933
H	-2.066369	-2.754441	-0.988868
H	-1.410431	1.606638	-0.786137
H	-0.789868	0.556990	-2.079784
H	-2.488267	0.473326	-1.599124
H	4.942565	2.651557	-2.805346
H	0.007720	2.070124	0.873255
H	0.191344	1.276438	2.437146
H	2.560231	2.316881	2.559985
H	1.180786	3.374512	2.775052
H	1.352584	4.111514	0.377637
H	2.697424	4.556021	1.412587
H	2.550278	3.776467	-2.070970
H	5.998572	1.701978	-0.450264
C	3.972664	-0.951308	-0.112596
H	4.182178	-0.350277	0.778072
H	4.921054	-1.027316	-0.654630

1 imaginary frequency
E_{B3LYP}= -2396.862669
G_{B3LYP}= -2396.435370
E_{M06-2X}= -2396.742930
G_{tot}= -2396.312612

exo-INT-S_N2' allylsilane C

Charge = 0 Multiplicity = 1

C	5.963593	-0.627804	0.464702
C	5.336897	-0.930706	-0.842136
O	4.569063	-2.134363	-0.570658
C	4.447254	-2.251090	0.726044
C	5.357551	-1.385144	1.412602
C	3.529468	-3.275981	1.263731
C	2.432631	-3.740572	0.294140
C	1.278860	-2.757319	0.049795
C	1.609186	-1.455374	-0.703465
C	2.244882	-0.396631	0.164168
C	3.306137	0.395025	-0.112432
C	4.246722	0.137293	-1.256111
O	0.437353	-0.878232	-1.284814
Si	-1.113630	-0.632805	-0.644721
C	-1.829079	0.720530	-1.726864
C	-1.077309	-0.095406	1.156272
C	-2.138361	-2.201275	-0.810023
Si	2.921620	3.208392	0.102313
C	3.570555	3.553154	-1.635704
C	1.048002	3.045245	0.057313
C	3.463938	4.573353	1.283522
F	-5.020712	-0.443924	-1.465384
C	-5.939862	-0.058951	-0.557000
F	-6.584382	1.010415	-1.054054
F	-6.828538	-1.058349	-0.424521
S	-5.141455	0.342638	1.070943
O	-4.214199	1.447119	0.726006
O	-6.287794	0.733051	1.923964

O	-4.487767	-0.934023	1.447772
H	4.791383	1.038764	-1.539407
H	3.755568	-0.259808	-2.145046
H	3.109912	4.377723	2.301086
H	3.061040	5.541804	0.968528
H	4.555347	4.658146	1.315795
H	4.665053	3.584553	-1.663938
H	3.231000	2.798258	-2.352301
H	3.202914	4.524308	-1.984529
H	0.636588	2.886897	1.059307
H	0.583664	3.945596	-0.358220
H	0.756448	2.194372	-0.564895
H	1.670392	-0.155591	1.056173
H	2.263365	-1.706157	-1.543933
H	-1.777994	-2.997839	-0.151571
H	-2.127329	-2.578758	-1.838181
H	-3.171681	-1.977723	-0.530473
H	-1.234450	1.637128	-1.660833
H	-1.865281	0.412893	-2.777239
H	-2.844686	0.948716	-1.393359
H	-0.649409	-0.857358	1.815913
H	-0.517951	0.833919	1.294602
H	-2.112539	0.076432	1.465149
H	6.711490	0.139649	0.609418
H	0.793786	-2.515040	1.003007
H	0.537198	-3.285353	-0.556032
H	2.890522	-4.023262	-0.659067
H	2.007218	-4.655688	0.714619
H	3.118287	-2.898021	2.205673
H	4.167261	-4.128566	1.543497
H	6.011113	-1.129863	-1.674466
C	3.651200	1.558383	0.763220
H	4.737629	1.692045	0.846299
H	3.258978	1.417974	1.776097
H	5.492418	-1.362642	2.483766

0 imaginary frequencies

E_{B3LYP}= -2396.905786

G_{B3LYP}= -2396.472935

$E_{M06\text{-}2X}$= -2396.790119
G_{tot}= -2396.354249

exo-INT-S_N2 allylsilane C

Charge = 0 Multiplicity = 1

C	-5.018736	-1.321595	-1.403917
C	-5.065748	-1.980007	-0.151792
O	-3.938465	-2.511319	0.192599
C	-2.917637	-2.091647	-0.785436
C	-3.726772	-1.419765	-1.823254
C	-2.141712	-3.332093	-1.245972
C	-1.362657	-3.979149	-0.099293
C	-0.419607	-2.972801	0.562176
C	-1.186729	-1.763681	1.101380
C	-1.993804	-1.050737	-0.012541
C	-2.784926	0.156428	0.457629
C	-3.518357	0.109931	1.578534
O	-0.365277	-0.813756	1.738363
Si	1.143612	-0.172867	1.295196
C	1.252289	1.399952	2.306436
C	1.195684	0.235417	-0.541794
C	2.490718	-1.385281	1.789057
Si	-3.892339	2.828898	-0.257468
C	-3.498250	4.000232	-1.683085
C	-5.674900	2.224026	-0.411720
C	-3.622103	3.737789	1.375427
F	4.052268	-1.058906	-0.950720
C	5.252082	-0.488815	-0.732477
F	5.842703	-1.149039	0.279827
F	5.996844	-0.668018	-1.836954
S	5.086059	1.319873	-0.334732
O	4.403951	1.320544	0.982841
O	6.491221	1.784535	-0.309711
O	4.269151	1.835846	-1.459799
H	-4.088664	0.964010	1.920961
H	-3.539503	-0.764137	2.219202

H	-3.918162	3.149298	2.248504
H	-4.201782	4.667245	1.390063
H	-2.565971	4.002564	1.495743
H	-2.462590	4.352372	-1.628838
H	-3.635130	3.508068	-2.651912
H	-4.151632	4.878798	-1.658194
H	-5.926619	1.482938	0.352319
H	-6.374725	3.060592	-0.312544
H	-5.842334	1.767475	-1.393145
H	-1.283034	-0.728074	-0.777589
H	-1.894174	-2.125532	1.857367
H	2.529001	-2.255175	1.127345
H	2.325723	-1.740737	2.811914
H	3.460608	-0.883947	1.745577
H	0.453159	2.098630	2.036672
H	1.175242	1.193438	3.378967
H	2.216821	1.878337	2.113703
H	1.136021	-0.653598	-1.177833
H	0.378000	0.909035	-0.818459
H	2.136503	0.740563	-0.777418
H	-5.857488	-0.851276	-1.893692
H	0.327160	-2.637368	-0.166880
H	0.123124	-3.438433	1.389395
H	-2.063602	-4.381603	0.640379
H	-0.798457	-4.827834	-0.495576
H	-1.457109	-2.995858	-2.031206
H	-2.844720	-4.033417	-1.702611
H	-5.905456	-2.125079	0.518302
C	-2.683387	1.367225	-0.436030
H	-2.739847	1.043457	-1.487211
H	-1.663569	1.770679	-0.343613
H	-3.305218	-1.043543	-2.745854

0 imaginary frequencies

E_{B3LYP}= -2396.891943

G_{B3LYP}= -2396.460981

E_{M06-2X}= -2396.777596

G_{tot}= -2396.343615

exo-TS2-S_N2' allylsilane C

Charge = 0 Multiplicity = 1

C	-5.438079	-0.247755	-0.227021
C	-4.979995	-1.268165	0.763934
O	-4.191856	-2.164277	-0.026305
C	-3.645961	-1.400365	-1.006271
C	-4.585116	-0.295328	-1.263218
C	-2.892210	-2.121505	-2.080725
C	-1.968886	-3.230104	-1.569010
C	-0.853092	-2.704323	-0.662870
C	-1.387052	-1.952511	0.555808
C	-2.074986	-0.638042	0.161405
C	-2.878491	0.081412	1.061500
C	-3.949695	-0.624779	1.810075
O	-0.380259	-1.636000	1.500888
Si	1.176815	-0.984048	1.292060
C	1.679676	-0.527620	3.037202
C	1.109351	0.556964	0.212808
C	2.328310	-2.293493	0.592286
Si	-3.032312	2.816132	-0.275961
C	-2.239474	4.374002	0.422240
C	-2.108750	2.286173	-1.827912
C	-4.856283	3.157726	-0.595800
F	3.606590	-0.296453	-1.500089
C	4.907915	-0.124681	-1.200765
F	5.447679	-1.340263	-0.999942
F	5.504788	0.423827	-2.272755
S	5.127459	0.952250	0.298962
O	4.580730	0.122741	1.400955
O	6.591927	1.162152	0.344308
O	4.325762	2.154978	-0.032664
H	-4.495644	0.042817	2.476140
H	-3.563581	-1.469150	2.383957
H	-5.332756	2.418544	-1.241558
H	-4.947416	4.133093	-1.086415
H	-5.416815	3.206222	0.343164

H	-2.715866	4.678096	1.359956
H	-1.171842	4.227657	0.614710
H	-2.343591	5.199872	-0.289236
H	-2.479631	1.358766	-2.269813
H	-2.211753	3.072492	-2.583916
H	-1.040350	2.162860	-1.624845
H	-1.445133	-0.021920	-0.475059
H	-2.105608	-2.593034	1.074431
H	2.169438	-2.466390	-0.475330
H	2.190844	-3.244206	1.118539
H	3.361442	-1.964698	0.729697
H	0.999852	0.215497	3.466806
H	1.686165	-1.403454	3.694300
H	2.688702	-0.106253	3.011043
H	0.817851	0.338742	-0.819692
H	0.400958	1.282043	0.627583
H	2.092789	1.033247	0.179775
H	-6.238923	0.455653	-0.048993
H	-0.188805	-2.045088	-1.235146
H	-0.245770	-3.540322	-0.305739
H	-2.564909	-3.974252	-1.030246
H	-1.531963	-3.739123	-2.432139
H	-2.337375	-1.380098	-2.664092
H	-3.652990	-2.541508	-2.752345
H	-5.740469	-1.827326	1.305111
C	-2.758415	1.537808	1.174066
H	-1.699947	1.753114	1.377469
H	-3.347478	1.921410	2.010985
H	-4.546347	0.338276	-2.136660

1 imaginary frequency

E_{B3LYP}= -2396.885940

G_{B3LYP}= -2396.450523

E_{M06-2X}= -2396.768675

G_{tot}= -2396.330239

exo-TS2-S_N2 allylsilane C

Charge = 0 Multiplicity = 1

C	-5.062707	-0.716239	-0.985694
C	-4.956592	-1.486019	0.244926
O	-4.000787	-2.411424	0.103481
C	-3.068268	-1.859856	-0.862543
C	-3.940624	-0.968439	-1.685758
C	-2.317585	-2.966884	-1.584468
C	-1.450732	-3.802092	-0.639690
C	-0.461924	-2.917554	0.124523
C	-1.192786	-1.846778	0.932679
C	-2.081346	-0.945225	0.024598
C	-2.845969	0.057009	0.829005
C	-3.853077	-0.358724	1.695274
O	-0.351823	-1.011694	1.691824
Si	1.170915	-0.345524	1.326934
C	1.346718	1.016669	2.599768
C	1.187139	0.378089	-0.411355
C	2.487998	-1.663029	1.562703
Si	-3.737154	2.693873	-0.351048
C	-2.944021	4.367733	-0.015202
C	-3.744076	2.388729	-2.209910
C	-5.459999	2.632700	0.402989
F	3.978225	-0.926832	-1.139159
C	5.196872	-0.443677	-0.832692
F	5.752991	-1.272106	0.069659
F	5.944671	-0.481991	-1.948696
S	5.095634	1.288771	-0.165783
O	4.398060	1.116740	1.132340
O	6.517159	1.687674	-0.061987
O	4.311793	2.000983	-1.203669
H	-4.438706	0.397904	2.208554
H	-3.783800	-1.312277	2.201616
H	-5.910504	1.637943	0.369096
H	-6.124147	3.316862	-0.135967
H	-5.430496	2.952136	1.449750
H	-2.896192	4.582278	1.057018
H	-1.926212	4.410965	-0.416364
H	-3.526275	5.162380	-0.493597

H	-4.490983	1.656272	-2.520981
H	-3.975523	3.331080	-2.718268
H	-2.763353	2.055301	-2.563417
H	-1.429882	-0.427903	-0.682161
H	-1.860455	-2.354336	1.639241
H	2.496232	-2.394671	0.750154
H	2.324330	-2.198285	2.504213
H	3.470776	-1.186774	1.597522
H	0.563836	1.773082	2.481090
H	1.286278	0.621449	3.619078
H	2.320744	1.496710	2.470526
H	1.056283	-0.378751	-1.191008
H	0.403135	1.132629	-0.535044
H	2.146845	0.869326	-0.592306
H	-5.884457	-0.060568	-1.231469
H	0.228808	-2.442164	-0.581261
H	0.140177	-3.516848	0.813342
H	-2.092582	-4.340275	0.066919
H	-0.912747	-4.557618	-1.218535
H	-1.692704	-2.487545	-2.346032
H	-3.048969	-3.587450	-2.109534
H	-5.768276	-1.731141	0.918071
C	-2.567754	1.483053	0.627466
H	-1.583475	1.625921	0.176356
H	-2.571450	1.979963	1.605253
H	-3.634845	-0.549499	-2.633605

1 imaginary frequency

E_{B3LYP}= -2396.882737

G_{B3LYP}= -2396.449019

$E_{M06\text{-}2X}$= -2396.765041

G_{tot}= -2396.328304

exo-Product allylsilane C

Charge = 0 Multiplicity = 1

C	-5.309734	-0.617051	-1.045983
C	-5.159816	-1.150808	0.367493

O	-4.239663	-2.235087	0.208134
C	-3.310618	-1.758940	-0.787317
C	-4.209573	-0.956837	-1.717461
C	-2.593246	-2.933633	-1.429080
C	-1.738585	-3.698826	-0.417987
C	-0.740458	-2.762320	0.265058
C	-1.434894	-1.613802	1.001450
C	-2.338234	-0.795568	-0.030260
C	-3.092187	0.227358	0.737948
C	-4.454779	-0.103719	1.257645
O	-0.565505	-0.751580	1.678521
Si	1.027122	-0.252883	1.318073
C	1.309145	1.118831	2.561605
C	1.085944	0.431164	-0.433833
C	2.219697	-1.673169	1.600966
Si	-2.912277	2.811235	-0.431700
C	-1.959088	4.291727	0.205671
C	-2.238127	2.173881	-2.054668
C	-4.760601	3.094018	-0.462979
F	3.759165	-1.129086	-1.103590
C	5.012870	-0.736773	-0.807103
F	5.506803	-1.592546	0.105337
F	5.750867	-0.847260	-1.924475
S	5.045857	1.007418	-0.164293
O	4.340161	0.905962	1.137216
O	6.493272	1.299713	-0.067631
O	4.314798	1.762636	-1.210389
H	-5.042399	0.806303	1.406184
H	-4.297196	-0.538199	2.256691
H	-5.303299	2.218865	-0.827512
H	-4.974373	3.926245	-1.142825
H	-5.146494	3.363870	0.524427
H	-2.320187	4.608829	1.188006
H	-0.889803	4.073846	0.279345
H	-2.089357	5.126234	-0.491865
H	-2.801801	1.316804	-2.427676
H	-2.323084	2.977109	-2.795323
H	-1.182081	1.899440	-1.987957

H	-1.641861	-0.309019	-0.717837
H	-2.120722	-2.041529	1.740207
H	2.203010	-2.405602	0.790280
H	1.982193	-2.188503	2.537680
H	3.233418	-1.270996	1.669448
H	0.598764	1.941911	2.429781
H	1.213153	0.750224	3.588028
H	2.321461	1.509073	2.424950
H	0.845985	-0.317808	-1.194995
H	0.383245	1.263807	-0.542970
H	2.084851	0.816467	-0.653435
H	-6.143320	-0.017282	-1.388106
H	-0.048622	-2.353484	-0.480241
H	-0.142036	-3.307347	1.000757
H	-2.388150	-4.160380	0.333787
H	-1.202619	-4.509512	-0.919687
H	-1.962156	-2.545278	-2.237437
H	-3.346466	-3.582176	-1.886279
H	-3.943157	-0.703391	-2.735639
H	-6.074559	-1.517691	0.834725
C	-2.513105	1.470054	1.035767
H	-1.424163	1.477757	1.006075
H	-2.943417	1.989154	1.895017

0 imaginary frequencies

E_{B3LYP}= -2396.910481

G_{B3LYP}= -2396.474223

E_{M06-2X}= -2396.797929

G_{tot}= -2396.358652
